# Selective and clean synthesis of aminoalkyl-*H*-phosphinic acids from hypophosphorous acid by phospha-Mannich reaction†

**DOI:** 10.1039/d0ra03075a

**Published:** 2020-06-04

**Authors:** Peter Urbanovský, Jan Kotek, Ivana Císařová, Petr Hermann

**Affiliations:** Department of Inorganic Chemistry, Faculty of Science, Universita Karlova (Charles University) Hlavova 8/2030, 12843 Prague 2 Czech Republic petrh@natur.cuni.cz +420-22195-1253 +420-22195-1263

## Abstract

Aminoalkyl-*H*-phosphinic acids, also called aminoalkylphosphonous acids, are investigated as biologically active analogues of carboxylic amino acids and/or as valuable intermediates for synthesis of other aminoalkylphosphorus acids. Their synthesis has been mostly accomplished by phospha-Mannich reaction of a P–H precursor, an aldehyde and an amine. The reaction is rarely clean and high-yielding. Here, reaction of H_3_PO_2_ with secondary amines and formaldehyde in wet AcOH led to aminomethyl-*H*-phosphinic acids in nearly quantitative yields and with almost no by-products. Surprisingly, the reaction outcome depended on the basicity of the amines. Amines with p*K*_a_ > 7–8 gave the desired products. For less basic amines, reductive *N*-methylation coupled with oxidation of H_3_PO_2_ to H_3_PO_3_ became a relevant side reaction. Primary amines reacted less clearly and amino-bis(methyl-*H*-phosphinic acids) were obtained only for very basic amines. Reaction yields with higher aldehydes were lower. Unique carboxylic–phosphinic–phosphonic acids as well as poly(*H*-phosphinic acids) derived from polyamines were obtained. Synthetic usefulness of the aminoalkyl-*H*-phosphinic was illustrated in P–H bond oxidation and its addition to double bonds, and in selective amine deprotection. Compounds with an ethylene-diamine fragment, *e.g.* most common polyazamacrocycles, are not suitable substrates. The X-ray solid-state structures of seventeen aminoalkyl-phosphinic acids were determined. In the reaction mechanism, *N*-hydroxyalkyl species R_2_NCH_2_OH and [R_2_N(CH_2_OH)_2_]^+^, probably stabilized as acetate esters, are suggested as the reactive intermediates. This mechanism is an alternative one to the known phospha-Mannich reaction mechanisms. The conditions can be utilized in syntheses of various aminoalkylphosphorus compounds.

## Abstract 2

Acetic acid was used as a new solvent for phospha-Mannich reaction leading to clear reaction mixtures and high yields of the aminoalkylphosphonous acids (AHPA), and hydroxymethylated species were suggested as key intermediates in the reaction.

## Graphical abstract

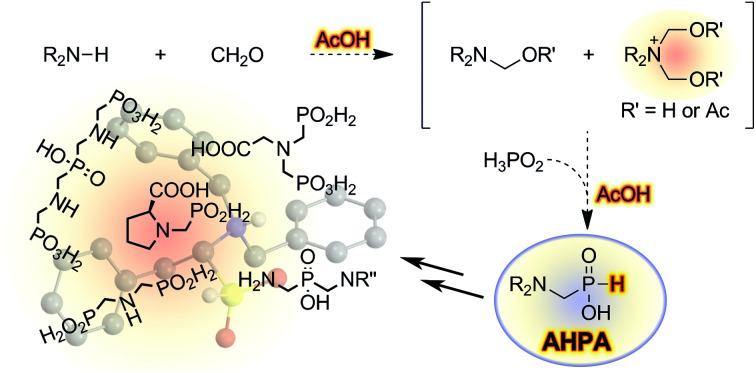

## Introduction

Phosphorus acid analogues of common amino acids have been studied for a long time.^1–4^ Within the compound family, the aminoalkylphosphonic acids are more frequently investigated than the others and some of them, *e.g.* glyphosate, are well known. Aminoalkylphosphinic acids have been less studied and they can be divided into two groups: (i) those containing two P–C bonds, *i.e.* bis(aminoalkyl)-phosphinic acids, and (ii) those with one P–C and one P–H bonds which are called aminoalkyl-*H*-phosphinic or aminoalkylphosphonous acids. Syntheses of the latter compounds have been the least studied among all kinds of aminoalkylphosphorus acids^5^ although they are the most suitable precursors in syntheses of the (unsymmetrical) phosphinic acids through further substitution of the P–H bond^6–10^ or can be also used for synthesis of phosphonic acids by oxidation of the P–H bond to P–OH bond.^11–14^

Phosphinic acid are analogues of carboxylic acids and, formally, they mimic tetrahedral intermediates in reactions involving carboxylic acid derivatives in biological systems, *e.g.* peptide bond hydrolysis. The acids are naturally occurring and their biosyntheses have been studied.^15^ Aminoalkylphosphonic and aminoalkylphosphinic acids are biologically active compounds and there are a number of their applications in biology and medicine as peptidomimetics, enzyme inhibitors, antiviral or antibacterial agents, herbicides, *etc.*^2,4–10^

Aminoalkylphosphorus acids are usually prepared by reaction of a precursor with a P(O)–H bond, an aldehyde and a primary/secondary amine.^3,5,16,17^ The most common P–H reagents for synthesis of the aminoalkyl-*H*-phosphinic acids are hypophosphorous acid, its esters or trivalent phosphines derived from the acid. The esters of hypophosphorous acids are generally not very stable, they are often prepared *in situ* and can be used only under very mild conditions.^18–20^ Their addition to imines leads to esters of 1-aminoalkyl-*H*-phosphinic acids.^21^ However due to their instability, the H_3_PO_2_ esters cannot be considered as reagents of choice, unlike diesters of H_3_PO_3_ (*i.e.* dialkyl/diaryl phosphites) which are the most common precursors for synthesis of aminoalkylphosphonic acids. Dialkoxyphosphines of general formula H–P(OR)_2_ are highly unstable pyrophoric compounds and only trimethylsilyl derivative, H–P(OSiMe_3_)_2_, is widely used as it can be very easily generated *in situ*.^22^ Its addition to imines gives (after hydrolysis of the trimethylsilyl groups) directly the desired 1-aminoalkyl-*H*-phosphinic acids.^23^ Phosphites derived from H_3_PO_2_ with one P–H bond protected have been also used in addition reactions to the imine double bond;^24^ however, the phosphites have to be prepared by special procedures and there is necessary a deprotection step which might be problematic. The cheapest and the most easily accessible reagent, H_3_PO_2_, has been frequently used as a nucleophile in addition to imines derived from primary amines.^5,16^ This approach has been used to obtain many *H*-phosphinic acid analogues of common amino acids but in variable, and mostly only moderate yields.^12,25–30^ The most simple one-pot reaction of an amine, an aldehyde and H_3_PO_2_ has been used in the syntheses less frequently.^31^ Generally, all these reactions lead to complicated reaction mixtures which are hard to purify and the desired amino-*H*-phosphinic acid (AHPA) might be only a minor product in the mixtures. The most of the above reaction procedures have been used for reactions of primary amines and, surprisingly, syntheses of AHPA derived from secondary amines are much less explored. As given above, the AHPA's are of an interest themselves and they are valuable intermediates in syntheses of wide range of other aminoalkylphosphorus acids. Therefore, any improvement of their synthesis, mainly from a view of clean reaction, is valuable.

For a long time, we have been involved in investigation of complexing properties of polyazamacrocycles modified with phosphonic/phosphinic acid pendant arms. The ligands can serve as carriers of metal ions for utilizations in biology or medicine. The phosphorus substituents in the pendant arms are used to finely tune various properties of the ligands as *e.g.* MRI related parameters,^32,33^ complexation rate,^34^ ligand bifunctionality^35,36^ or targeting properties.^35–37^ To further explore possibilities offered by *P*-aminoalkyl substituents on the phosphinic acid pendant arms (*e.g.* tuning basicity of amino group or its bifunctionality), the AHPA's would be the most valuable precursors. However, missing general procedure for their synthesis is a limiting factor for those purposes. Recently, we have found that acetic acid was a suitable solvent for phospha-Mannich reaction of H_3_PO_2_.^33^ Therefore, we decided to investigate in more details these conditions of phospha-Mannich reaction of H_3_PO_2_ (sometimes called Moedritzer–Irani–Redmore reaction). Scope of the reaction and investigation of the reaction mechanism are described in this paper.

## Results

We have recently successfully used acetic acid as a solvent (at 40 °C) for reaction of H_3_PO_2_, paraformaldehyde and Bn_2_NH to get a gram amount of *N*,*N*-dibenzyl-aminomethyl-*H*-phosphinic acid 1.^33^ Under these conditions, no formation of the most expected by-products, *i.e.* Bn_2_N–Me, HOCH_2_–P(O)(OH)–CH_2_NBn_2_, H_3_PO_3_ or Bn_2_NCH_2_–PO_3_H_2_, was observed. As the reaction led to almost pure product, purification of the reaction mixture could be carried out by a simple chromatography on strong cation exchanger. Such a clear synthesis was rather surprising and, with our best knowledge, AcOH as a solvent has not been used for this kind of phospha-Mannich reaction before. The most traditional solvent for the reaction is water. Thus, influence of water content in the reaction mixture was tested in the reaction with Bn_2_NH (Table S1 and Fig. S1†). Under the aforementioned conditions, a small amount of water was always present due to utilization of commercial 50% aq. H_3_PO_2_. Utilization of crystalline H_3_PO_2_ (*i.e.* under fully anhydrous conditions) did not improve conversion to the product or shorten reaction time. Therefore, small water content (up to ∼5% w/w) does not alter the reaction outcome. Increased amount of water in acetic acid progressively slowed down the reaction and lowered the yield. Oxidation of H_3_PO_2_ to H_3_PO_3_ was not detectable in wet AcOH and, thus, reductive *N*-methylation is efficiently suppressed under these reaction conditions. The aqueous phospha-Mannich reaction with H_3_PO_3_ is commonly carried out in 1 : 1 aq. HCl (*i.e.* in ∼18% aq. HCl).^38^ Here, addition of only one equiv. of HCl (as Bn_2_NH·HCl) led to much lower conversion and observation of by-products and, with more HCl, almost no conversion was observed (Table S2 and Fig. S2†). In the reaction with 1 equiv. of HCl, bis-substituted H_3_PO_2_ (*i.e.* (Bn_2_NCH_2_)_2_PO_2_H_2_) and the *N*-methylated amine (*i.e.* Bn_2_N–Me) were clearly detected after the reaction (Fig. S3†). If mixture with ten equiv. of HCl was heated to 60 °C, a complicated reaction mixture was obtained where HOCH_2_PO_2_H_2_ and AcOCH_2_PO_2_H_2_ were major components; the desired compound 1 was only a minor product (∼7%). Without HCl in solution and in the presence of all three components, formation of the HOCH_2_PO_2_H_2_ was observed only after the complete consumption of the amine and if an excesses of formaldehyde and H_3_PO_2_ over the amine were used, and after long reaction times.

Reactivity of H_3_PO_3_ as H–P precursor was tested as well. Some small conversion was observed for Bn_2_NH and (C_6_H_11_)_2_NH (*i.e.* Cy_2_NH) but the reactions were slow (H_3_PO_3_ consumption was not complete even after several days). The desired aminomethylphosphonic acids (APON's) were formed together with a significant amount of H_3_PO_4_ and it was connected with extended reductive *N*-methylation of the used amines (Fig. S4†). Elevated temperature (60 °C) accelerated consumption of H_3_PO_3_ but mainly due to its oxidation. The pure product, Bn_2_NCH_2_PO_3_H_2_ (A) and Cy_2_NCH_2_PO_3_H_2_ (B), were isolated in a zwitter-ionic form, albeit in a low yields (∼25%).

Similarly to H_3_PO_3_, the P–H bond in *H*-phosphinic acids is much less reactive than that in H_3_PO_2_. Anyway, some *H*-phosphinic acids were tested in reaction with Bn_2_NH (40 °C, 1 d). The Ph-PO_2_H_2_ and PhtNCH_2_–PO_2_H_2_ gave the corresponding bis-substituted phosphinic acids, (Ph)(Bn_2_NCH_2_)PO_2_H (C) and (PhtNCH_2_)(Bn_2_NCH_2_)PO_2_H (D), and the phosphonic acids, Ph-PO_3_H_2_ and PhtNCH_2_–PO_3_H_2_, in molar ratios ∼1 : 8 and ∼3 : 2, respectively. Thus, reductive *N*-methylation of Bn_2_NH (with simultaneous oxidation of the *H*-phosphinic acids) was significant. Despite complex reaction mixtures, these bis-substituted phosphinic acids (C) and (D) were purified and characterized. With HO_2_CCH_2_CH_2_PO_2_H_2_, the corresponding phosphonic acid was almost exclusively formed and only a small amount of the desired bis-substituted phosphinic acid (∼5%) was detected in the reaction mixture. In addition, AHPA prepared in this work were also tested. Thus, 1 was reacted with an equiv. of Bn_2_NH and formaldehyde at 40 °C and (Bn_2_NCH_2_)_2_PO_2_H (ref. 39) was obtained together with the corresponding “redox” products, Bn_2_NCH_2_PO_3_H_2_ and Bn_2_N–Me (Fig. S5†). At higher temperature (60 °C), the starting materials were consumed faster but more extensive oxidation (∼60%) and even *P*-hydroxymethylation (∼10%) of 1 were observed. Reaction of Cy_2_NCH_2_PO_2_H_2_ (5, see below) with Bn_2_NH and formaldehyde led to the (Cy_2_NCH_2_)(Bn_2_NCH_2_)PO_2_H (E) and no phosphonic acid, Cy_2_NCH_2_PO_3_H_2_, was observed. However, the reaction at 40 °C was very slow and a full conversion of 5 could not be achieved even after heating at 60–80 °C up to four days and, at the temperatures, (HOCH_2_)(Cy_2_NCH_2_)PO_2_H was also formed in a significant amount.

### Reaction of secondary amines, formaldehyde and H_3_PO_2_

**Scheme 1:**
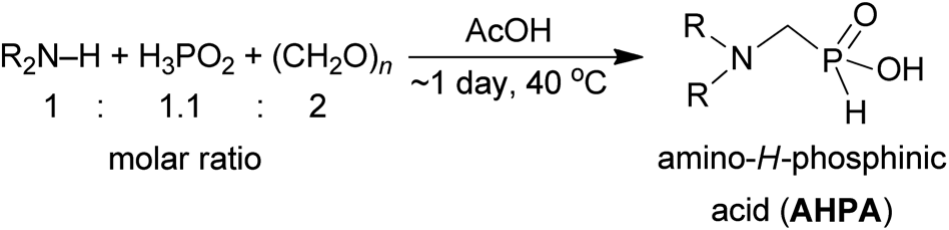
Reaction of secondary amines, paraformaldehyde and H_3_PO_2_.

As our goal was to get an access to a small library of *N*-substituted (1-aminomethyl)-*H*-phosphinic acids giving us a possibility to tune properties of pendant arm(s) in polyaza-macrocyclic ligands with the macrocycle–CH_2_PO_2_H–CH_2_NR_2_ fragment, a range of amines in the reaction was investigated. First, reactions of H_3_PO_2_, formaldehyde and various secondary monoamines were tested (Scheme 1). Acetic acid as a solvent has one practical advantage – it is a good solvent for even very hydrophobic amines which are not soluble in water or diluted aq. HCl which have been used as solvents earlier. The secondary amine, H_3_PO_2_ (1.1 equiv.) and paraformaldehyde (2 equiv.) were mixed in acetic acid and the suspension-to-solution (paraformaldehyde slowly dissolved during the reaction course) was heated at 40 °C in oil bath till ^31^P NMR spectroscopy showed no changes in composition of the reaction mixture. Conversions were estimated from ^31^P NMR spectra of the reaction mixtures (large P–H doublet of triplets for AHPA with ^1^*J*_PH_ ∼ 520–570 Hz, non-split triplet of H_3_PO_2_ with ^1^*J*_PH_ ∼ 530 Hz, or non-split doublet of H_3_PO_3_ with ^1^*J*_PH_ ∼ 650 Hz). Completion of the reactions required several hours up to 1–2 days. Most of the AHPA's were isolated as solids or thick oils after a simple ion exchange on strong cation exchanger (Dowex 50). The results are summarized in Table 1. Higher reaction temperature led to some reductive *N*-methylation of the starting amines (and concomitant oxidation of H_3_PO_2_ acid to H_3_PO_3_), to formation of hydroxymethyl-*H*-phosphinic acid which can be acetylated (*i.e.* formation of AcOCH_2_PO_2_H_2_). Therefore, the temperature was kept at 40 °C despite longer reaction times were necessary to finish the reactions. Slight excess of H_3_PO_2_ somewhat reduced risk of the “redox” reaction of the desired AHPA (*i.e.* formation of the corresponding aminoalkylphosphonic acids, APON's) as H_3_PO_2_ is preferentially oxidized over the AHPA. Despite utilization of an only small excess of H_3_PO_2_ and larger excess of formaldehyde, no *P*-hydroxymethylation of AHPA was observed. Under the used conditions (wet AcOH, 40 °C), the *P*-hydroxymethylation occurred only on H_3_PO_2_ and only after complete consumption of the starting amine.

**Table 1:** Reaction of secondary amines (1.0 mmol), H_3_PO_2_ (as 50% aq. solution) and paraformaldehyde in molar ratio 1 : 1.1 : 2, respectively, in AcOH at 40 °C followed by purification on Dowex 50, if not stated otherwise

Entry	Amine	Product	Conversion^a^ (after 24 h, %)	Isolated yield (%)	log *K*_a_ of the starting amine^b^
1	Bn_2_NH	Bn_2_NCH_2_PO_2_H_2_ (1)	95	78	8.5
2	Me_2_NH^c^	Me_2_NCH_2_PO_2_H_2_ (2)^d^	88	>85^e^	10.8
3	Et_2_NH	Et_2_NCH_2_PO_2_H_2_ (3)^d^	92	>85^e^	11.0
4	iPr_2_NH	iPr_2_NCH_2_PO_2_H_2_ (4)	89	>85^e^	11.1
5	Cy_2_NH	Cy_2_NCH_2_PO_2_H_2_ (5)	98	78	11.3
6	Bn(Me)NH	Bn(Me)NCH_2_PO_2_H_2_ (6)	98	>85^e^	9.6^f^
7	Piperidine	C_5_H_10_NCH_2_PO_2_H_2_ (7)^d^	92	>85^e^	11.0
8	Morpholine	O(CH_2_CH_2_)_2_NCH_2_PO_2_H_2_ (8)^d^	92	>85^e^	8.6
9	(CF_3_CH_2_)(Bn)NH	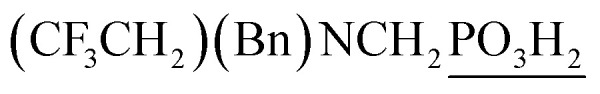 (9)	0^g^	(5)^h^^,^^l^	5.4
10	(CF_3_CH_2_)_2_NH	—	0^g^	—	1.2
11	HO_2_CCH_2_(Me)NH (sarcosine)	(HO_2_CCH_2_)(Me)NCH_2_PO_2_H_2_ (10)	90	69	10.0
12	HO_2_CCH_2_(Bn)NH (*N*-Bn-glycine)	(HO_2_CCH_2_)(Bn)NCH_2_PO_2_H_2_ (11)	75	57	9.2
13	(HO_2_CCH_2_)_2_NH (H_2_ida)	(HO_2_CCH_2_)_2_NCH_2_PO_2_H_2_ (12)^i^	— j	89	9.3
14	l-Proline	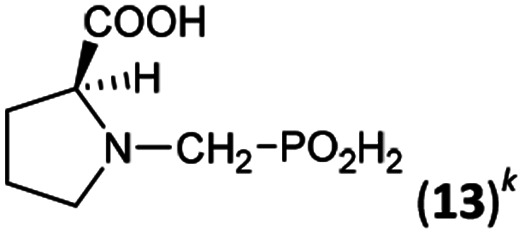	88	73	10.4
15	HOCH_2_CH_2_(Me)NH	HOCH_2_CH_2_N(Me)CH_2_PO_2_H_2_ (14a)^l^	53 (14a)	33 (14a)^e^	9.9
		[HOCH_2_CH_2_N(Me)CH_2_]_2_PO_2_H (14b)^l^	40 (14b)	30 (14b)^e^	
16	(HOCH_2_CH_2_)_2_NH	(HOCH_2_CH_2_)_2_NCH_2_PO_2_H_2_ (15a)	6 (15a)	— (15a)	8.9
		[(HOCH_2_CH_2_)_2_NCH_2_]_2_PO_2_H (15b)^l^	70 (15b)	46 (15b)^e^	
17	*N*-Me-piperazine	MeN(CH_2_CH_2_)_2_NCH_2_PO_2_H_2_ (16)	25^g^	20^e^	9.0 and 4.8
18	(PhtNCH_2_CH_2_)_2_NH	(PhtNCH_2_CH_2_)_2_NCH_2_PO_2_H_2_ (17)	70	63	8.5
19	Ph(Me)NH	—	Mixture^g^	—	4.9
20	Imidazole	—	0	—	7.0

a Determined by ^31^P NMR spectroscopy, based on amine.

b Basicities of the amines were taken from databases^40^ or predicted.^41^

c 40% aq. solution of Me_2_NH was used.

d Ref. 31*a*.

e Isolated as a thick oil.

f Ref. 42.

g Significant oxidation of H_3_PO_2_ accompanied by *N*-methylation was observed.

h Isolated yield of the corresponding phosphonic acid.

i Ref. 34*b* and 43.

j Product precipitated during the reaction.

k Ref. 44.

l Special purification procedure was used, see ESI.

Dialkylamines (Entries 1–6, Table 1) gave the high/quantitative conversion to the corresponding APHS's 16 as well as good isolated yields, despite an increasing steric hindrance brought by the alkyl substituents. Simple cyclic amines (Entries 7 and 8) reacted similarly to give the desired products 7 and 8. Introducing a strongly electron withdrawing 2,2,2-trifluoroethyl group on the amine nitrogen atom (Entries 9 and 10) resulted in dominant oxidation of H_3_PO_2_ together with *N*-methylation of amines (Fig. S6†) and the corresponding *N*-methylated amines were isolated. In the case of (benzyl)(2,2,2-trifluoroethyl)amine (Entry 9), only a small amount of the corresponding phosphonic acid 9 was isolated in a reasonable purity to identify it and the isolated 9 was contaminated with a small amount of the corresponding *H*-phosphinic acid (see ESI,† characterization spectra). Thus under the conditions, any formed AHPA was probably quickly oxidized to phosphonic acid. Amino acids (*N*-Me-Gly, *N*-Bn-Gly, H_2_ida or l-proline; Entries 11–14) gave the corresponding *H*-phosphinic acids 10–13 with great conversions and in high isolated yields. Reaction with amines containing 2-hydroxyethyl group(s) (Entries 15 and 16) surprisingly led mainly to bis(aminomethyl)phosphinic acids 14b and 15b even in molar ratio of the reactants 1 : 1 : 1. In the case of (HOCH_2_CH_2_)(Me)NH, monosubstituted product 14a was obtained after ion-exchange column chromatography purification in a low yield. With diethanolamine, the desired *P*-monosubstituted amino acid 15a was formed only as a very minor component of the reaction mixture and could not be isolated. The main product was bis(aminomethyl)phosphinic acid derivative 15b and, in the crude reaction mixture, it is partially present as an intramolecular ester and, thus, an esterification of the phosphinic acid group probably took place. To simplify the mixture, these impurities were hydrolysed with hot azeotropic aq. HCl and, afterwards, pure bis-substituted phosphinic acids 14b and 15b were isolated.

Reaction of *N*-methyl-piperazine (Entry 17) led to a small yield of 16 (25% conversion) and a significant *N*-methylation was observed; the *N*,*N*′-dimethyl-piperazine was identified as a main product. Thus, fragment N–C–C–NH seems to be not suitable for the reaction (see also below). However if one amine of the N–C–C–N fragment is fully protected as in (Pht-NCH_2_CH_2_)_2_NH (Entry 18), the reaction underwent smoothly and the desired amino acid (17) was isolated in a good yield and no *N*-methylation was observed.

Utilization of a simple aromatic amine (*N*-Me-aniline, Entry 19) led to a complicated reaction mixture which was not possible to purify. A significant oxidation of H_3_PO_2_ was clearly observed. Imidazole, an example of a simple heterocyclic amine (Entry 20), did not react at all (even no oxidation of H_3_PO_2_ was observed). Simple amides with different electron donating effect and bulkiness of the *N*-alkyl groups were also tested. None of *N*-Me-formamide, *N*-Me-acetamide, *N*-Et-acetamide, *N-t*-Bu-acetamide, and *N*-Cy-acetamide reacted under the used conditions and, thus, even the electronically rich secondary amides did not produce *N*-acyl-AHPA.

### Reaction of secondary amines, higher aldehydes and H_3_PO_2_

**Scheme 2:**
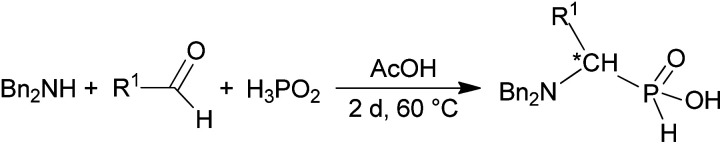
Reaction of Bn_2_NH, aldehydes and H_3_PO_2_.

In the reactions shown in Scheme 1, only formaldehyde was used as the carbonyl component. To determine scope of the reaction while utilizing other aldehydes, the reaction was carried out with several aliphatic and aromatic aldehydes (Scheme 2 and Table 2) and with a model secondary amine, *N*,*N*-dibenzylamine. Utilization of the aldehydes generates a chiral centre and, thus, the AHPA's were obtained as racemic mixtures.

**Table 2:** Reaction of Bn_2_NH (1.0 mmol), H_3_PO_2_ (as 50% aq. solution) and aldehydes (molar ratio 1 : 1.1 : 2; AcOH, 60 °C, 2 d) followed by purification on Dowex 50, if not stated otherwise

Entry	Aldehyde	Product	Conversion^a^ (after 48 h, %)	Isolated yield (%)
21	Me-CHO	Bn_2_NCH(CH_3_)PO_2_H_2_ (18)	85	69^b^
22	Paraldehyde (acetaldehyde trimer)	Bn_2_NCH(CH_3_)PO_2_H_2_ (18)	88	71^b^
23	*n*-Pr-CHO	Bn_2_NCH(CH_2_CH_2_CH_3_)PO_2_H_2_ (19)	55	42^b^
24	PhCH_2_-CHO^c^^,^^d^	Bn_2_NCH(CH_2_Ph)PO_2_H_2_ (20)	33	16
25	*t*Bu-CHO	—	3	—
26	CF_3_-CHO^d^^,^^e^	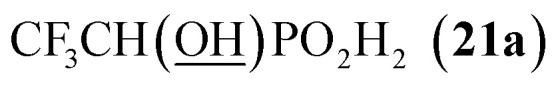	52^f^	(24)^b^
		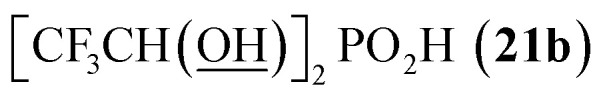	<5^f^	—g

a Determined by ^31^P NMR spectroscopy, based on amine.

b Isolated as a thick oil.

c Freshly distilled aldehyde was used.

d At 80 °C, 3 d.

e Used as a monohydrate (fluoral hydrate).

f Conversion based on H_3_PO_2_.

g Prepared and characterized after reaction with an excess of fluoral hydrate (see ESI).

All reactions had to be performed at higher temperature (60 °C) than with formaldehyde as, otherwise, the reactions were too slow. Even under these conditions, no significant *P*-hydroxyalkylation of H_3_PO_2_ or the formed AHPA was observed, as well as no oxidation of H_3_PO_2_ or the AHPA (Fig. S7†). Reaction with acetaldehyde (Entry 21) afforded the desired AHPA 18 with a high conversion and in a good yield. Use of its cyclic trimer, paraldehyde, did not change the outcome of reaction (Entry 22). Paraldehyde is not stable under the acidic conditions and slowly depolymerizes.^45^ Use of longer carbon-chain aldehyde, *n*-butyraldehyde (Entry 23) gave the desired amino acid 19 with a lower conversion and isolated yield, and it might be explained by a lower reactivity of the higher aldehydes. Freshly distilled phenylacetaldehyde (Entry 24) required more harsh conditions (80 °C, three days) and, anyway, the conversion to the desired AHPA 20 and the isolated yield were low. The lower conversion may be contributed to a preferential polymeration of the aldehyde under the given conditions.^46^ Addition of more aldehyde into the reaction mixture during the reaction time improved the conversion only slightly. Surprisingly, commercial phenylacetaldehyde stabilized with citric acid (only 0.01%) did not react at all. Sterically hindered pivalaldehyde, *t*Bu-CHO, (Entry 25) did not afford any desired amino acid even at higher temperature (80 °C) and on prolonged reaction time (three days). Only H_3_PO_2_*P*-hydroxyalkylation and the hydroxyacid acetylation (*i.e.* formation of *t*BuCH(OAc)–PO_2_H_2_) were observed. Reaction of other secondary amines, Cy_2_NH, piperidine, or Me_2_NH, with pivalaldehyde did not lead to any desired AHPA. Aromatic aldehyde, benzaldehyde, afforded the desired product with only a small conversion (∼10%) and PhCH(OAc)–PO_2_H_2_ and PhCH(OH)–PO_2_H_2_ were observed as major components of the reaction mixture. If higher temperature (80 °C) and longer reaction time (three days) were used, conversion to the desired amino acid reached ∼30% but together with many side products (Fig. S8†). Other secondary amines (Cy_2_NH, piperidine and Me_2_NH) were also tested but no improvement in the conversion or composition of the reaction mixture were observed (max. ∼20% of AHPA, 3 d, 80 °C) and a significant oxidation of H_3_PO_2_ to H_3_PO_3_ was always observed. More importantly, the phospha-Mannich products, (R_2_N)(phenyl)methyl-*H*-phosphinic acids, decomposed during purification of these reaction mixtures. Utilization of aromatic aldehyde with more electron withdrawing group, *p*-nitrobenzaldehyde, led to no observable change in ^31^P NMR spectra during reaction time. Trifluoroacetaldehyde (Entry 26) was tested as the most electron-poor aldehyde. At 60 °C after one day, no reaction was observed in ^31^P NMR spectrum. At 80 °C after one day, *P*-hydroxyalkylation took place giving the compound 21a, CF_3_CH(OH)–PO_2_H_2_, as a main product together with a small amount of compound 21b, [CF_3_CH(OH)]_2_PO_2_H (Fig. S9†). If the reaction was carried out without presence of amine, molar ratio of 21a and 21b was ∼2 : 1 (Fig. S9†). With high excess of fluoral hydrate, 21b was isolated in a high yield (see ESI†). Interestingly, no significant oxidation to H_3_PO_3_ was observed in these reactions. Finally, reaction with the simplest ketone, acetone, was tested. No change in ^31^P NMR spectrum of the reaction mixture was observed even after heating at 80 °C for three days.

### Reaction of primary amines, formaldehyde and H_3_PO_2_

**Scheme 3:**
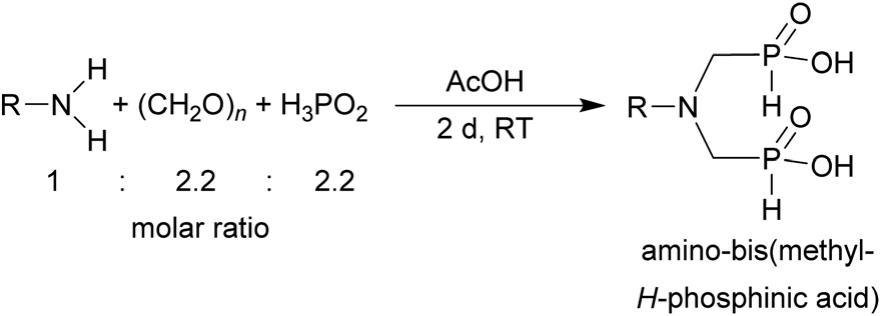
Reaction of primary amines, paraformaldehyde and H_3_PO_2_.

In the next step, reactivity of primary amines was tested (Table 3). Aliphatic amines were used and reaction conditions (various ratio of reactants, temperature *etc.*) were widely altered. Reactions with methylamine always led to mixtures which were hard to purify. For higher amines as BnNH_2_ (Entry 27), PhCH_2_CH_2_NH_2_ (PhenNH_2_, Entry 28), CyNH_2_ (Entry 29), *t*BuNH_2_ (Entry 30) or AdNH_2_ (Entry 31), the expected *N*,*N*-bis(methyl-*H*-phosphinic acids) 22–26 (Scheme 3) could be obtained if the reactions were carried out with slight excesses of H_3_PO_2_ (2.2 equiv.) and paraformaldehyde (2.2 equiv.). The conversions were only moderate (26–46%, Table 3, *e.g.* Fig. S10†) as well as isolated yields (∼35%). Utilization of higher excesses of H_3_PO_2_ or paraformaldehyde led to a higher extent of side reactions (*e.g. P*-hydroxymethylations) and the mixtures were hardly separable. Pure monosubstituted amino acids, R–NHCH_2_PO_2_H_2_, could not be obtained during these attempts. Generally, purification of the alkylamine-bis(methyl-*H*-phosphinic acids) was problematic as these compounds are not retained on Dowex 50; the AHPA's, the simple phosphorus acids and *P*-hydroxymethyl phosphinic acids were all eluted together with water and they had to be separated by chromatography on silica, leading to low isolated yields.

**Table 3:** Reaction of primary amines (0.5 mmol), H_3_PO_2_ (as 50% aq. solution), paraformaldehyde in molar ratio 1 : 2.2 : 2.2 (AcOH, 2 d, room temperature) followed by purification on Dowex 50 and/or silica, if not stated otherwise

Entry	Amine	Product	Conversion^a^ (after 48 h, %)	Isolated yield^b^ (%)	log *K*_a_ of starting amine^c^
27	Bn-NH_2_	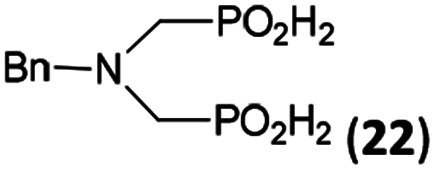	44	34	9.3
28	Phen-NH_2_	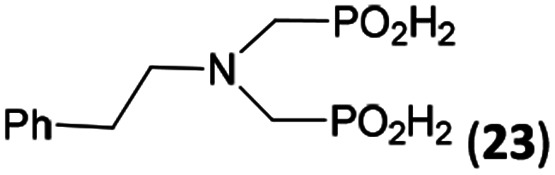	44	32	9.8
29	Cy-NH_2_	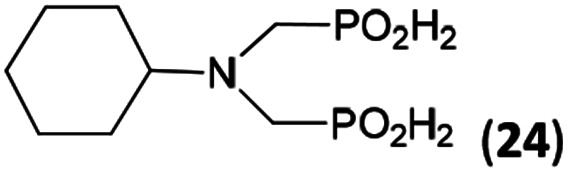	48	33	10.6
30	*t*Bu-NH_2_	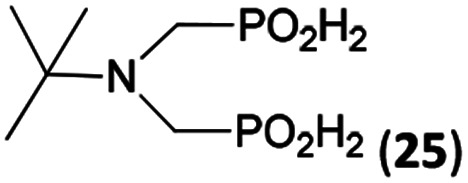	26	20	10.5
31	Ad-NH_2_	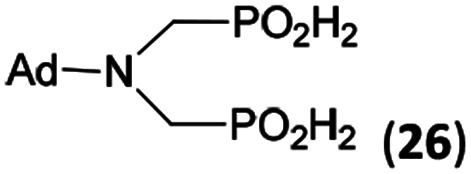	44	31	10.5
32	H_2_O_3_P–CH_2_–NH_2_^d^	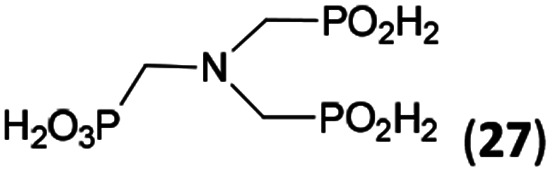	94	∼70^e^	10.0

a Determined by ^31^P NMR spectroscopy, based on amine.

b Isolated as thick oils.

c Basicities of the amine groups were taken from database^40^ or predicted.^41^

d 2 equiv. of anhydrous AcONa was added to dissolve the amino acid in AcOH; molar ratio of amino acid, aq. H_3_PO_2_, and paraformaldehyde was 1 : 4 : 2.5.

e Yield of not fully purified product (∼85% purity).

The NH_2_CH_2_PO_3_H_2_ was tested as amino acid with basic primary amine group (Entry 32). Surprisingly, it reacted smoothly with some excess of H_3_PO_2_ (4 equiv.) and paraformaldehyde (2.5 equiv.) to give the desired bis(*H*-phosphinic acid) 27 with an excellent conversion (94%); isolated yield of a crude product was ∼70% (the amine containing by-products could not be fully removed). As the aminomethylphosphonic acid is not soluble in AcOH, sodium acetate (2 equiv.) was added to dissolve it. Surprisingly, reactions with glycine afforded rich mixtures with a significant oxidation of H_3_PO_2_ to H_3_PO_3_.

### Reaction of *N*-alkyl-aminomethylphosphorus acids, formaldehyde and H_3_PO_2_

**Scheme 4:**
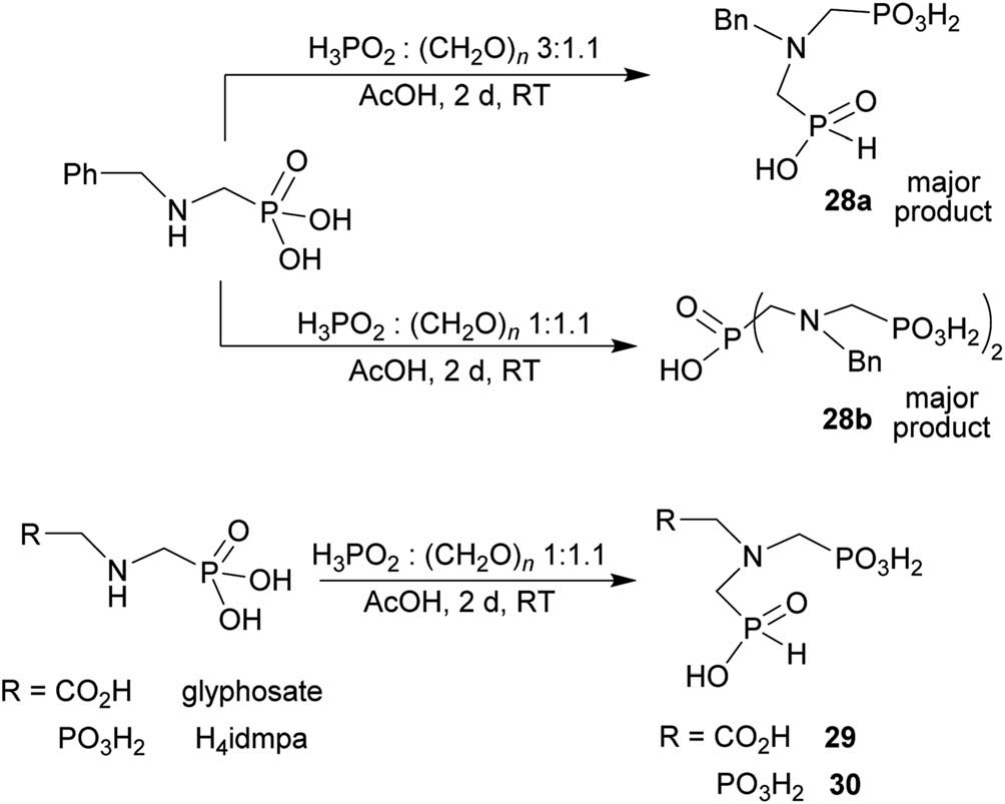
Synthesis of phosphorus amino acids containing *N*-methylphosphonic-*N*-methyl-*H*-phosphinic acid pendant group.

Other amino acids with a secondary amine group were tested as well (Scheme 4 and Table 4). A simple amino-methyl-*H*-phosphinic acid, BnNH–CH_2_PO_2_H_2_ (for an improved synthesis, see ESI†), was reacted under the above conditions and a non-separable mixture of products was obtained. The starting amino acid is, in principle, intermediate in the reaction of BnNH_2_ discussed above. If aminophosphonic acid BnNH–CH_2_PO_3_H_2_ was used (Entry 33), the reaction led to a mixture where the desired AHPA 28a and *P*-disubstituted phosphinic acid 28b as a by-product (Fig. S11†) were present in molar ratio ∼3 : 1; both compounds were isolated. Thus similarly to the (2-hydroxyethyl)-amines (Entries 15 and 16), the <svg xmlns="http://www.w3.org/2000/svg" version="1.0" width="10.400000pt" height="16.000000pt" viewBox="0 0 10.400000 16.000000" preserveAspectRatio="xMidYMid meet"><metadata> Created by potrace 1.16, written by Peter Selinger 2001-2019 </metadata><g transform="translate(1.000000,15.000000) scale(0.011667,-0.011667)" fill="currentColor" stroke="none"><path d="M80 1160 l0 -40 40 0 40 0 0 -40 0 -40 40 0 40 0 0 -40 0 -40 40 0 40 0 0 -40 0 -40 40 0 40 0 0 -40 0 -40 40 0 40 0 0 -40 0 -40 40 0 40 0 0 -40 0 -40 40 0 40 0 0 80 0 80 -40 0 -40 0 0 40 0 40 -40 0 -40 0 0 40 0 40 -40 0 -40 0 0 40 0 40 -40 0 -40 0 0 40 0 40 -40 0 -40 0 0 40 0 40 -80 0 -80 0 0 -40z M560 520 l0 -40 -40 0 -40 0 0 -40 0 -40 -40 0 -40 0 0 -40 0 -40 -40 0 -40 0 0 -40 0 -40 -40 0 -40 0 0 -40 0 -40 -40 0 -40 0 0 -40 0 -40 -40 0 -40 0 0 -40 0 -40 80 0 80 0 0 40 0 40 40 0 40 0 0 40 0 40 40 0 40 0 0 40 0 40 40 0 40 0 0 40 0 40 40 0 40 0 0 40 0 40 40 0 40 0 0 80 0 80 -40 0 -40 0 0 -40z"/></g></svg> N–CH_2_PO_3_H_2_ fragment accelerates the double substitution of H_3_PO_2_ and the reaction was clearly preferred even if amino acid-to-H_3_PO_2_ molar ratio was 1 : 1. However if a higher excess of H_3_PO_2_ (3 equiv., see Table 4) was used, conversion to the desired AHPA 28a was improved and the compound was isolated in a moderate yield. In the crude reaction mixtures, almost no *N*-methylation was detected (<5%). Similarly, *N*-phosphonomethyl-glycine (Entry 34) gave a derivative 29 where *H*-phosphinic, phosphonic and carboxylic acid functions are attached to the same nitrogen atom. Despite the high conversion, isolated yield was low due to problematic separation of the highly polar and acidic components of the reaction mixture. Purification on the strong cation exchange resin separated only by-products derived from H_3_PO_2_ (*e.g.* HOCH_2_PO_2_H_2_) and the amine-containing components could not be fully separated. Highly basic diphosphonic acid, H_4_idmpa, reacted smoothly (Entry 35) to give diphosphonic-*H*-phosphinic acid product 30 in ∼70% isolated yield. In the Entries 34 and 35 where starting zwitter-ionic amino-methylphosphonic acids insoluble in AcOH were used, sodium acetate was added and the starting amino acids slowly dissolved and reacted.

**Table 4:** Reaction of phosphorylated secondary amines (1.0 mmol), H_3_PO_2_ (as 50% aq. solution) and paraformaldehyde in molar ratio 1 : 3 : 1.1 (AcOH, 2 d, room temperature)

Entry	Amine	Product	Conversion^a^ (after 48 h, %)	Isolated yield (%)	log *K*_a_ of starting amine^b^
33	H_2_O_3_PCH_2_–NH–Bn	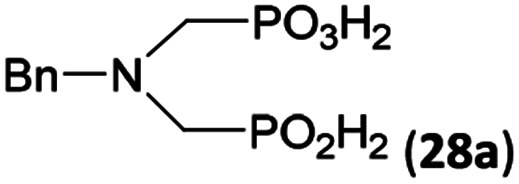	93	49^c^	10.0
		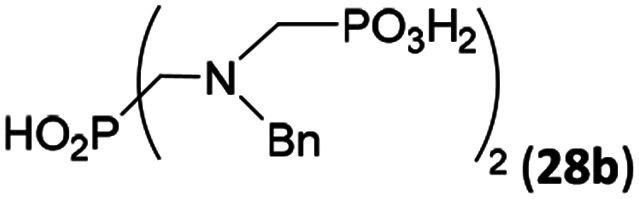	<5	—d	
34	H_2_O_3_PCH_2_–NH–CH_2_CO_2_H (glyphosate)^e^	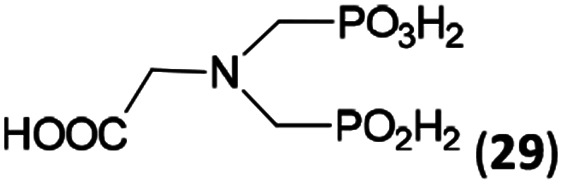	92 (<5)^f^	∼60^g^	10.0
35	(H_2_O_3_PCH_2_)_2_NH (H_4_idmpa)^e^	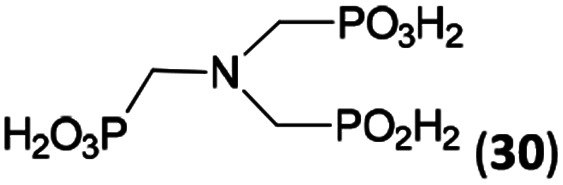	85 (<5)^f^	∼70^g^	11.5^h^

a Determined by ^31^P NMR spectroscopy, based on amine.

b Basicities of the amines were taken from database^40^ or predicted.^41^

c Isolated by using C18 silica column chromatography.

d Prepared and isolated under different conditions, see ESI.

e Two equiv. of anhydrous AcONa per phosphonate group were added to dissolve the amino acid in AcOH.

f Conversion to AHPA; conversion to bis-substituted phosphinic acid is in parenthesis.

g Phosphorus acids were partially co-eluted with the product and repeated chromatographic purification was necessary. The yields of not fully purified product (purity ∼85% and ∼90% for 29 and 30, respectively) are given.

h Ref. 47.

### Reaction of linear secondary polyamines, formaldehyde and H_3_PO_2_

**Scheme 5:**
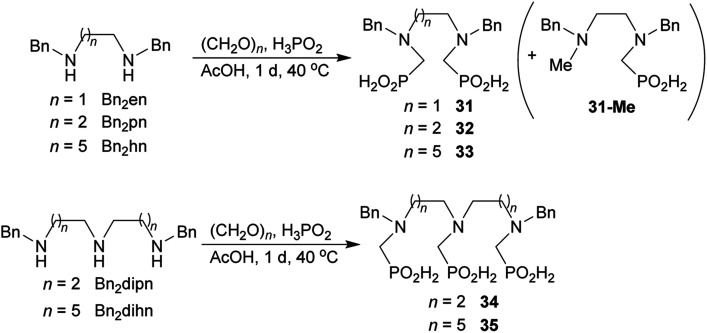
Reaction of linear secondary polyamines, formaldehyde and H_3_PO_2_.

Reactions with linear secondary polyamines were also tested (Table 5) as the reaction can lead to interesting polydentate ligands. Simple linear secondary diamine, *N*,*N*′-dibenzyl-ethylene-diamine (Bn_2_en, Entry 36) afforded two products (Scheme 5), the desired *N*,*N*′-bis-substituted (31) and *N*-methylated mono-substituted (31-Me) amino acids in molar ratio ∼2.5 : 1 (Fig. S12†). The products were separated by C18 reverse-phase silica column chromatography as both compounds are strongly stuck to the cation exchange resin. When spacer between the amines was longer as in *N*,*N*′-dibenzyl-propylene-diamine (Bn_2_pn, Entry 37), the reaction afforded the expected amino-bis(*H*-phosphinic acid) 32 as a major product and no reductive *N*-methylation and H_3_PO_3_ were detected in the reaction mixture. The product 32 was easily isolated on ion-exchange resin. Further extension of the spacer between the secondary amines in *N*,*N*′-dibenzyl-hexylene-diamine (Bn_2_hn, Entry 38) favoured formation of the α,ω-bis(*H*-phosphinic acid) leading to almost quantitative conversion and a very good isolated yield of 33. Triamines were used as well (Scheme 5). Reaction of the *N*,*N*′′-dibenzyl-diethylene-triamine (Bn_2_dien, Entry 39) led to an intractable reaction mixture of various products (Fig. S13†). Reaction of *N*,*N*′′-dibenzyl-dipropylene-triamine (Bn_2_dipn, Entry 40) or *N*,*N*′′-dibenzyl-dihexylene-triamine (Bn_2_dihn, Entry 41) afforded the desired amino acids 34 and 35 (Fig. S13†), respectively, in good yields.

**Table 5:** Reaction of secondary polyamines (0.25 mmol), H_3_PO_2_ (as 50% aq. solution) and paraformaldehyde in molar ratio 1 : 1.1*x* : 2*x* (*x* is a number of secondary amino groups) in AcOH (2 mL) at 40 °C, 1 d, followed by purification on Dowex 50, if not stated otherwise

Entry	Amine	Product	Conversion^a^ (after 24 h, %)	Isolated yield (%)	log *K*_a_ of amine group^b^
36	Bn–NH(CH_2_)_2_NH–Bn (Bn_2_en)	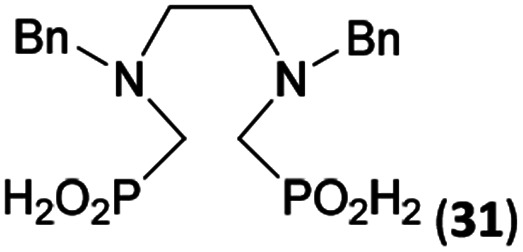	58	51^c^	8.9 and 6.0
		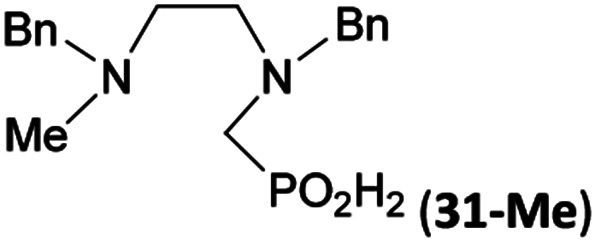	11	10^c^	
37	Bn–NH(CH_2_)_3_NH–Bn (Bn_2_pn)	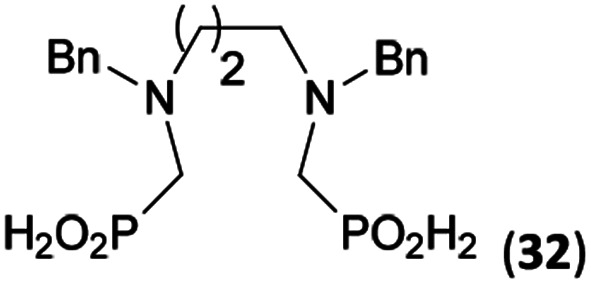	82	68^d^	9.7^e^
38	Bn–NH(CH_2_)_6_NH–Bn (Bn_2_hn)	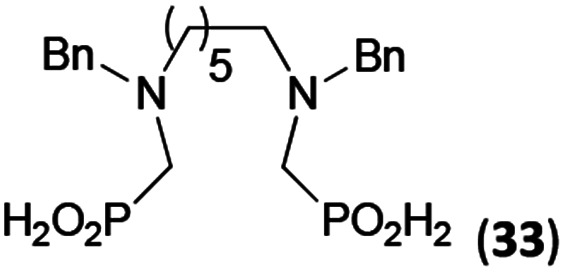	88	80^d^	10.1^e^
39	Bn–NH(CH_2_)_2_NH(CH_2_)_2_NH–Bn (Bn_2_dien)	—	Mixture	—	9.4^e^
40	Bn–NH(CH_2_)_3_NH(CH_2_)_3_NH–Bn (Bn_2_dipn)	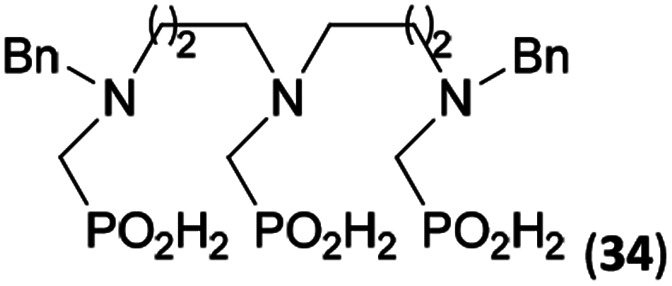	91	85^d^	10.1^e^
41	Bn–NH(CH_2_)_6_NH(CH_2_)_6_NH–Bn (Bn_2_dihn)	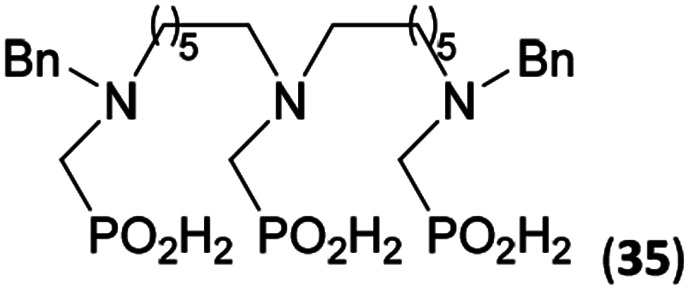	93	82^d^	10.8^e^

a Determined by ^31^P NMR spectroscopy, based on amine.

b Basicities of the amines were taken from database^40^ or predicted.^41^

c A special purification procedure, see ESI.

d Isolated as thick oils.

e Only the first log *K*_a_ could be predicted;^41^ basicities of the other amine group(s) are several orders of magnitude lower than (ethylene) or rather similar to (propylene, hexamethylene) the value in the table.

### Reaction of cyclic secondary polyamines, formaldehyde and H_3_PO_2_

**Fig. 1:**
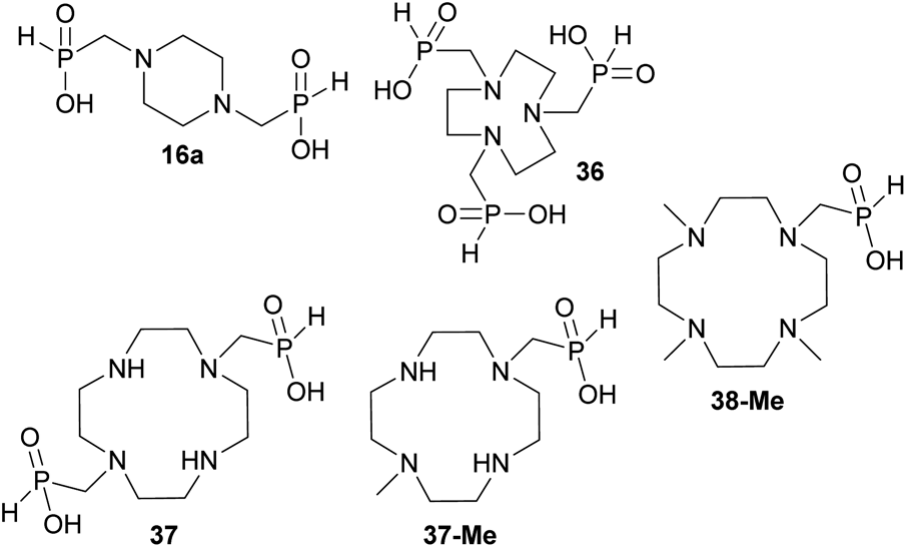
Structures of the isolated amino-*H*-phosphinic acid derived from cyclic secondary polyamines discussed in the text.

The simplest cyclic secondary diamine, piperazine, gave two AHPA products which were separated using strong anion exchanger. The desired bis(*H*-phosphinic) acid (16a, Fig. 1)^31^ was isolated in a moderate yield (37%) and other product was *H*-phosphinic acid with the other amine *N*-methylated, 16 (22%). Presence of a closely located secondary amine probably triggered unwanted “redox” process with H_3_PO_2_ (Fig. S14†), thus, piperazine-*N*-methyl-*H*-phosphinic acid was more prone to further *N*-methylation (*i.e.* forming product 16). Surprisingly if piperazine was used, preparation of *N*′-methylated *H*-phosphinic acid 16 proceeded with a similar yield as reaction where *N*-Me-piperazine was the starting amine (Entry 17, Table 1).

Reaction with 1,4,7-triazacyclononane (tacn) was dependent on the reactant molar ratio. The tacn was reacted with 2.2 equiv. of formaldehyde per amino group and various molar amount of H_3_PO_2_. If H_3_PO_2_ acid was equimolar to tacn, formation of a compound with *δ*_P_ < 0 ppm was observed (*i.e.* with a P–O–P moiety) and no *H*-phosphinic acid was detected (Fig. S15†). With more H_3_PO_2_ (3–5 equiv., based on tacn) as well as formaldehyde, no P–O–P compound was observed and major products were methyl-*H*-phosphinic acids. In these mixtures, 1,4,7-triazacyclononane-1,4,7-tris(methyl-*H*-phosphinic acid) 36 (Fig. 1) was a major product (∼70% conversion for 5/5 equiv. of H_3_PO_2_/formaldehyde) and it was isolated in a moderate yield (∼50%). Reactions with twelve-membered tetraazamacrocycle, cyclen, led to very complicated reaction mixtures which cannot be purified and a significant oxidation to H_3_PO_3_ was always detected. If paraformaldehyde was added gradually, some *H*-phosphinic acids were observed but only after addition of several equiv. of (CH_2_O)_*n*_ (Fig. S16†). However during the time, H_3_PO_2_ was continuously oxidized to H_3_PO_3_ and *N*-methyl derivatives of cyclen were formed. Reaction of cyclen with excess of paraformaldehyde and H_3_PO_2_ (6 equiv. each) did not improve the conversion to any amino-*H*-phosphinic acid even at 40 °C for three days. Reaction of 1,7-bis(benzyloxycarbonyl)-cyclen with paraformaldehyde (3 equiv.) and H_3_PO_2_ (3 equiv.) gave a rich mixture (40 °C, two days). The mixture could be partially separated on C18-silica to get the desired bis(*H*-phosphinic acid) derivative and a *N*-methylated by-product which were directly deprotected in aq. HCl to give cyclen-1,7-bis(methyl-*H*-phosphinic acid) 37 (Fig. 1) and 7-methyl-cyclen-1-(methyl-*H*-phosphinic acid) 37-Me (Fig. 1) in only a small overall yield (∼5 and ∼15%, see ESI†). Reaction of 1,7-dimethyl-cyclen proceeded with *N*-methylation of the remaining amine groups and only 4,7,10-trimethyl-cyclen-1-(methyl-*H*-phosphinic acid) 38-Me (Fig. 1) was detected in the reaction mixture with a low conversion (∼20%) and it was isolated in a low yield (∼15%, see ESI†). Fourteen-membered tetraazamacrocycles, cyclam, produced bis(formaldehyde)-diaminal^48^ as a single product under these conditions. This bis-aminal is probably formed immediately by reaction of cyclam with formaldehyde and it does not react with H_3_PO_2_ at all. Under the used conditions, only signal of H_3_PO_2_ was detected in ^31^P NMR spectra with no change with time even at 40 °C. Excess of paraformaldehyde and H_3_PO_2_ (6 equiv. each) did not lead to the conversion to any AHPA's. The 1,4,8-trimethyl-cyclam^34*c*^ did not reacted under our conditions and only unchanged H_3_PO_2_ was observed in the ^31^P NMR spectra.

### Reaction mechanism investigations

**Fig. 2:**
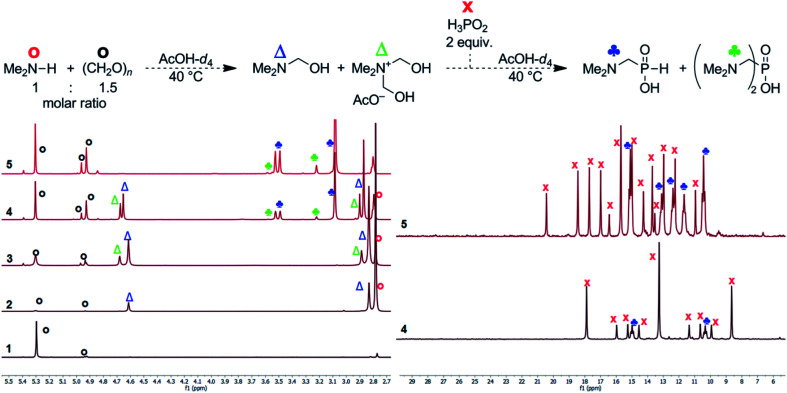
Reaction of H_3_PO_2_, Me_2_NH, paraformaldehyde followed by ^1^H (left) and ^31^P (right) NMR (0.2 mmol of amine, final molar ratio 2 : 1 : 1.5, respectively; 40 °C, AcOH-*d*_4_ (0.4 mL)). (1) 1.5 equiv. (CH_2_O)_*n*_ in AcOH-*d*_4_, 15 min at 40 °C; (2) 40% aq. Me_2_NH added (1 equiv.), reaction time 90 min at 40 °C; (3) reaction time 960 min at 40 °C; (4) addition of 50% aq. H_3_PO_2_ (2 equiv.), reaction time 5 min at room temperature; (5) reaction time 150 min at 40 °C. The ^31^P NMR spectra were not referenced and they show complicated ^31^P–^2^D couplings (non-binomial multiplets) due to utilization of AcOH-*d*_4_.

To get more information about the reaction, mechanism of the reaction was investigated with model secondary amine, Me_2_NH. Thus, changes in mixture of H_3_PO_2_, Me_2_NH (1 equiv.) and formaldehyde with time were followed in more details by NMR spectroscopy in AcOH-*d*_4_ (Fig. 2). If only paraformaldehyde (1.5 equiv.) was added to the amine solution, ^1^H NMR signal of the methyl groups of the (CH_3_)_2_N fragment (∼2.79 ppm, the starting amine) was slowly transformed to signals at ∼2.82 and ∼2.86 ppm, and two new signals assigned to a methylene group at ∼4.60 and ∼4.66 ppm appeared (their methyl-to-methylene intensity ratios were 6 : 2 and 6 : 4, respectively; Fig. 2, traces 2 and 3). The ^1^H NMR spectrum slowly evolved to an equilibrium state (during several hours) but intensity ratios of each signal pair was not changed. In a separate experiment, paraformaldehyde was gradually added to (Me)_2_NH solution (Fig. S17†) and intensity of 2.82/4.60 ppm pair *vs.* 2.86/4.66 ppm pair increased with more formaldehyde added. Only explanation of the results is a gradual formation of Me_2_NCH_2_OH and [Me_2_N(CH_2_OH)_2_]^+^ intermediates (with methyl-to-methylene intensity ratios 6 : 2 and 6 : 4, respectively). As the reaction was carried out in AcOH-*d*_4_, both compounds might be stabilized by acetylation of the alcohol group and, therefore, Me_2_NCH_2_OAc and [Me_2_N(CH_2_OAc)_2_]^+^ could be also considered as products of the reaction of Me_2_NH with formaldehyde. After addition of the last reactant, aq. H_3_PO_2_ (2 equiv.), into the amine and formaldehyde mixture (Fig. 2), the desired Me_2_NCH_2_PO_2_H_2_ started to be formed immediately and the reaction mixture did not change after ∼150 min with a complete conversion of the starting amine. Only a small amount of product of bis-substitution of H_3_PO_2_ (*i.e.* (Me_2_NCH_2_)_2_PO_2_H) was observed. The ^1^H and ^31^P NMR spectra mutually correspond (Fig. 2). If analogous experiment was carried out in D_2_O, a quick formation of the amine-formaldehyde intermediates was also observed but their reaction with H_3_PO_2_ was very slow (Fig. S18†).

Reaction of H_3_PO_2_ with the presumed intermediates, Me_2_NCH_2_OR and [Me_2_N(CH_2_OR)_2_]^+^, was further investigated. If H_3_PO_2_ (1 equiv.) was added to the already prepared (at 40 °C for 1 d) mixture of Me_2_NH (1 equiv.) and paraformaldehyde (2 equiv.), the reaction did not change after 5 h at 40 °C and two *H*-phosphinic acids were formed in molar ratio ∼8.5 : 1 (Fig. S19†). These *H*-phosphinic acids were identified as Me_2_NCH_2_PO_2_H_2_2 and [Me_2_N(CH_2_OR)(CH_2_PO_2_H_2_)]^+^ (R = H or Ac, a minor product) on the basis of 2D NMR spectra (Fig. S20†). This cationic derivative could be formed from the intermediate [Me_2_N(CH_2_OR)_2_]^+^ cation. Thus, H_3_PO_2_ reacts with both Me_2_NCH_2_OR and [Me_2_N(CH_2_OR)_2_]^+^ to form *H*-phosphinic acids. The formation rate of these two *H*-phosphinic acids was evaluated in time (Fig. S21†). Under the used conditions (40 °C, AcOH), [Me_2_N(CH_2_OR)(CH_2_PO_2_H_2_)]^+^ (R = H or Ac) cation was stable and did not decompose, even at elevated temperature (80 °C, 5 h) and after addition of an excess of conc. aq. HCl (Fig. S22†). However, this quarternary ammonium cation was easily decomposed to Me_2_NCH_2_PO_2_H_2_ (compound 2) after addition of a water excess (Fig. S22†). Thus, the [Me_2_N(CH_2_OR)(CH_2_PO_2_H_2_)]^+^ cation might be also considered as an reaction intermediate together with Me_2_NCH_2_OR and [Me_2_N(CH_2_OR)_2_]^+^ cation.

Imines (=Schiff bases) have been commonly suggested as intermediates in the phospha-Mannich reaction. To check this alternative, commercial (Me_2_N=CH_2_)^+^Cl^−^ was dissolved in AcOH-*d*_4_. The ^1^H NMR spectrum recorded just after dissolution showed a different pattern of the signals (Fig. S23†) than that in Fig. 2. This experiment provided chemical shifts of the iminium cation (3.74/∼8.0 ppm, intensity ratio 6 : 2). The ^1^H spectrum slowly evolved due to instability of the cation in a protic solvent. The main signals under equilibrium were assigned to Me_2_NH, Me_2_NCH_2_OR and [Me_2_N(CH_2_OR)_2_]^+^ (R = H or Ac), and only the minor one to the (Me_2_N=CH_2_)^+^ cation (3.74 and ∼8.0 ppm). Closer inspection of the Me_2_NH/paraformaldehyde mixture in AcOH-*d*_4_ discussed above showed that only a small amount of the iminium cation was present in the equilibrated mixture. All three compounds might be in equilibrium and could be considered as reaction intermediates. Addition of aq. H_3_PO_2_ (1 equiv.) to the solution of (Me_2_N=CH_2_)^+^Cl^−^ in AcOH-*d*_4_ at 40 °C resulted in a quick formation of the desired product 2 but together with the bis-substituted phosphinic acid (Me_2_NCH_2_)_2_PO_2_H giving the final molar ratio of the acids ∼2.5 : 1, respectively (Fig. S24†). Under these reaction conditions, the iminium cation may participate in the reactions directly or after its hydrolysis to hydroxymethyl derivative. In addition, the P–H bond of already formed 2 further reacts to give undesired (Me_2_NCH_2_)_2_PO_2_H. After one day at 40 °C, even some *P*-hydroxymethylated species were detected and it can be caused by the presence of HCl. If analogous experiment was carried out with the solid anhydrous H_3_PO_2_ (1 equiv.), reaction was instantaneous and both mono- and bis-substituted phosphinic acids were formed in molar ratio ∼1 : 1 (Fig. S25†). The iminium cation was completely consumed and ∼40% H_3_PO_2_ remained unreacted because of total consumption of the iminium cation. Therefore, reaction of the iminium cation in the absence of water together with the presence of HCl leads to a higher conversion to undesired bis-substituted phosphinic acids.

Another experiments were done with a commercial aminal, (Me_2_N)_2_CH_2_. In AcOH-*d*_4_, the aminal immediately reacted and decomposed to mixture of Me_2_NH, (Me_2_N=CH_2_)^+^ cation and presumably Me_2_NCH_2_OH/Me_2_NCH_2_OAc in molar ratio ∼6 : 5 : 1 (Fig. S26†). Composition of the reaction mixture remained unchanged after 60 min at 40 °C. After addition of anhydrous H_3_PO_2_, signals of the mono- and bis-substituted phosphinic acids slowly appeared and signal intensity of the iminium cation decreased; however, the reaction was considerably slower than that in presence of water (see above). An addition of D_2_O (4 equiv.; molar amount approx. equal to water content in the 50% aq. H_3_PO_2_ if added) resulted in a complete transformation of the iminium cation to the (acetylated) *N*-hydroxymethylated amine (Fig. S27†). After addition of H_3_PO_2_ to this solution, conversion to the bis-substituted phosphinic acid was suppressed as consequence of hydrolysis of the iminium cation. Thus, some bis-substitution of H_3_PO_2_ is possible even in absence of HCl (see above) due to a high reactivity of the iminium cation (if present in the equilibrium).

To probe reasons for *N*-methylation of the ethylene-diamine fragment in polyamines, model polyamines were reacted with paraformaldehyde in AcOH-*d*_4_ at 40 °C. In the case of piperazine and *N*,*N*′-Bn_2_-ethylene-diamine, the corresponding cyclic aminals were formed quickly. If only one equiv. of H_3_PO_2_ was added to these solutions, both aminals did not react to give monosubstituted AHPA and complex reaction mixtures were obtained. If two equiv. of H_3_PO_2_ were added, both aminals reacted to produce the corresponding *N*,*N*′-bis(substituted) AHPA's together with several by-products, mainly *N*′-methylated AHPA's. Hence, the *N*-monosubstituted AHPA's cannot be prepared under conditions used in this work and only *N*,*N*′-bis(substituted) AHPA's could be isolated with excess of H_3_PO_2_.

Reaction of the model primary amine, BnNH_2_, with paraformaldehyde was also investigated in AcOH-*d*_4_ (40 °C). After 3 hours, BnNH_2_ was partially converted to/into its cyclic triazine trimer (*i.e.* 1,3,5-tribenzyl-1,3,5-triazacyclohexane). Further heating (40 °C, additional 4 hours) led to gradual decomposition of the trimer into a rich mixture.

### Further reactions of the prepared aminoalkyl-*H*-phosphinic acids

**Scheme 6:**
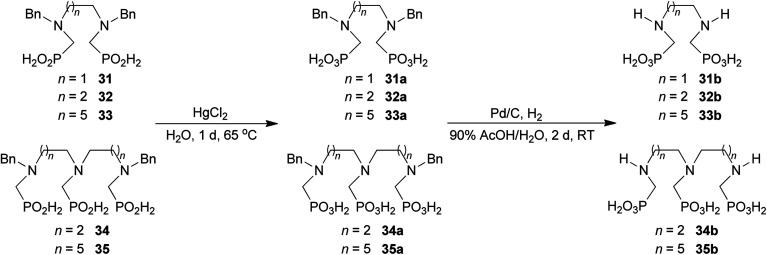
Synthesis of polyamino-polyphosphonic acids.

To further utilize the prepared AHPA's, we decided to prepare a few examples of (aminomethyl)phosphonates with secondary amine groups which are hardly accessible by other methods (Scheme 6 and Table 6). The *N*-benzyl-(poly)amino-(methyl-*H*-phosphinic acids) were chosen as model substrates (Entries 42–46). The *N*-benzyl protection of the amines could not be removed by hydrogenation on Pd/C as P–H bond would poison the catalyst. Thus, the P–H bonds were first oxidized to the corresponding phosphonic acids by divalent mercury.^11,12^ Conversions were practically quantitative. Isolation consisted only from two filtrations (removal of Hg_2_Cl_2_ and HgS) and several evaporations. Complete oxidation was carried out with 1.5 equiv. of HgCl_2_ per *H*-phosphinic acid group and it may be carried out in pure water instead of diluted aq. HCl (the original procedure). Reaction temperature had to be at least 65 °C (no reaction was observed at 50 °C). The procedure afforded pure *N*-benzyl-(poly)amino-(poly)phosphonic acids 31a–35a. The *N*,*N*′-Bn_2_-ethylene-diamine-*N*,*N*′-bis(methyl-*H*-phosphinic acid) 31a had to be oxidized as its ammonium salt due to its low solubility in water. The zwitter-ionic form of phosphonic acid 31a was obtained after removal of mercury(i,ii) ions and simple acidification of the reaction mixture.

**Table 6:** Oxidation of amino-methyl-*H*-phosphinic acid to amino-phosphonic acid (1.5 equiv. of HgCl_2_ per P–H bond, 1 d, 65 °C) and *N*-benzyl group removal (1 atm H_2_, Pd/C, 90% aq. AcOH, 2 d, room temperature)

Entry	*H*-Phosphinic acid	Oxidation	Hydrogenolysis	Isolated yield (over two steps, %)
42	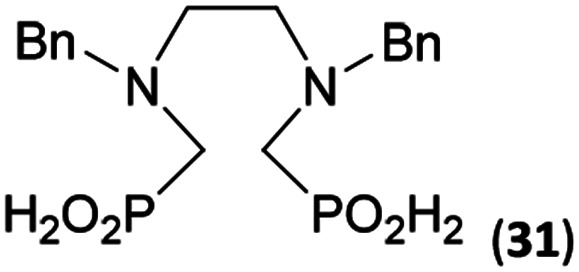	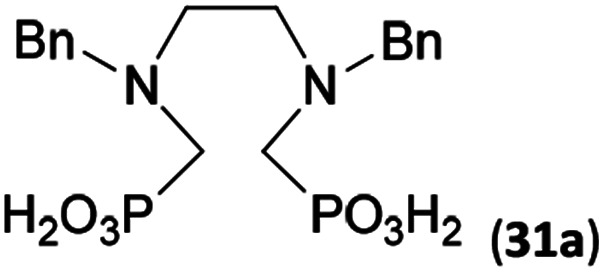	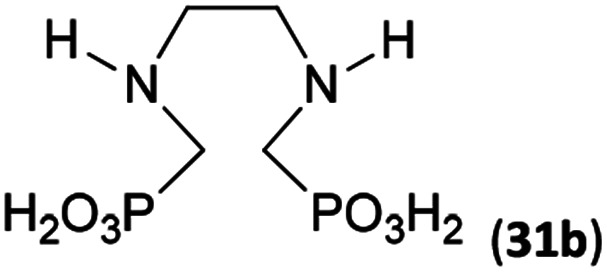	72
43	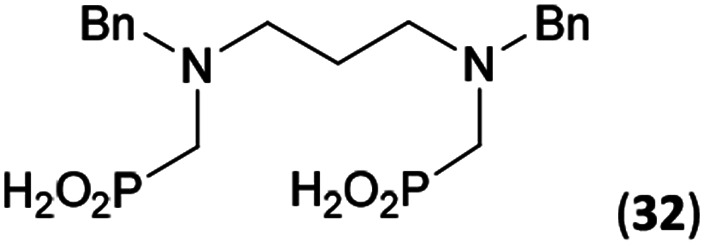	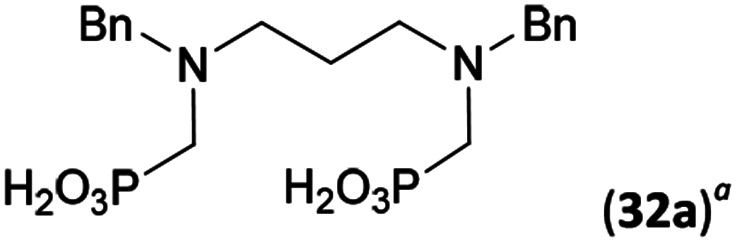	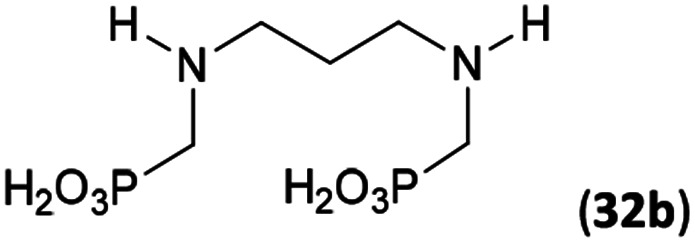	80
44	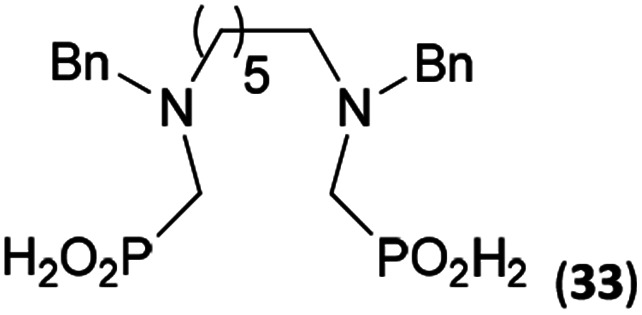	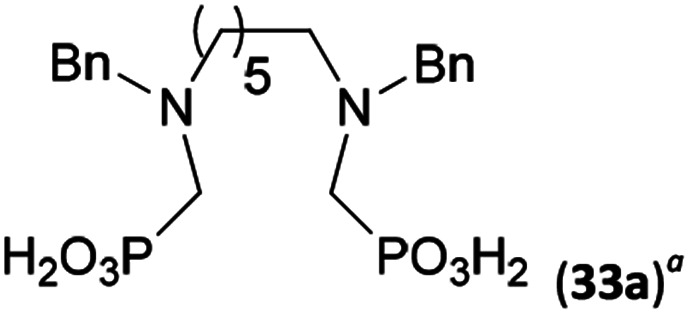	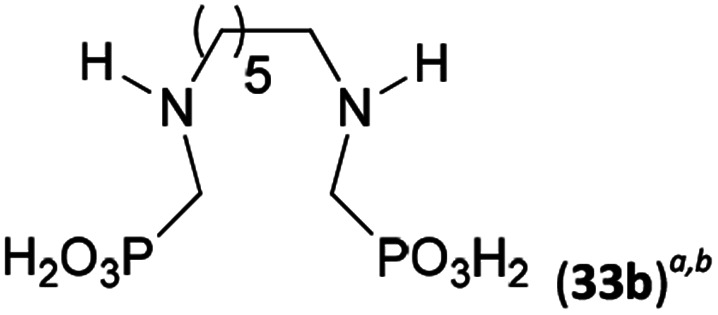	70
45	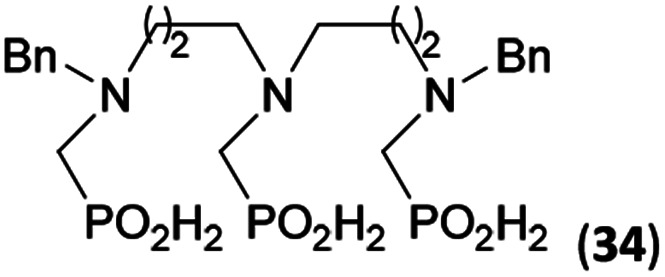	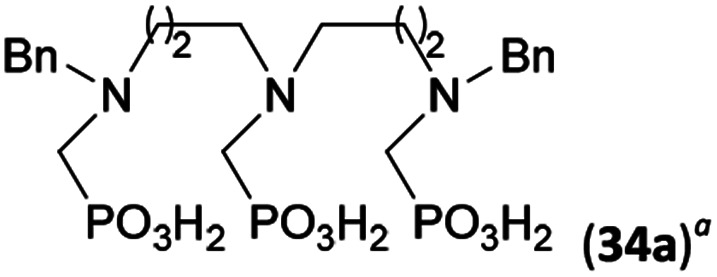	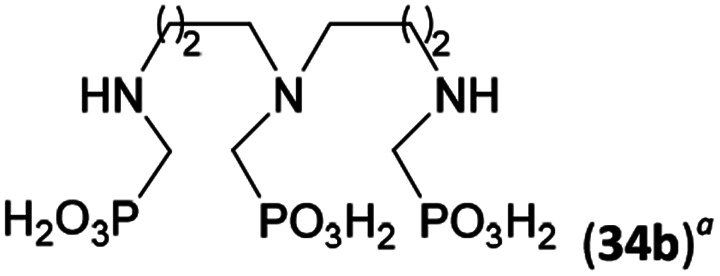	84
46	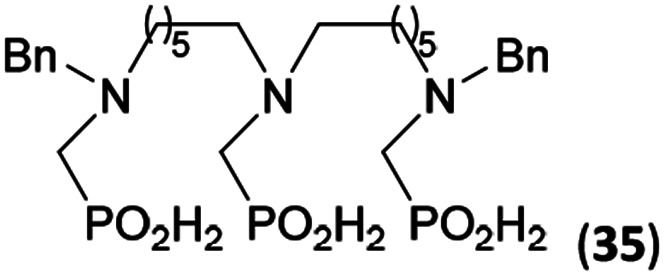	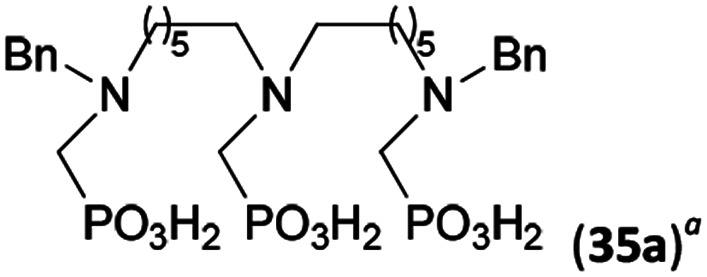	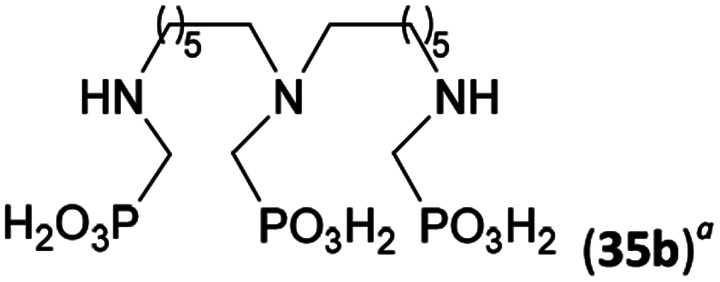	82

a Isolated as a thick oil.

b Hydrogenolysis required 75 °C.

Next, the *N*-benzyl groups were removed by hydrogenation on Pd/C in aq. AcOH as a solvent. The desired poly(amino)-poly(methylphosphonic acids) 31b–35b were prepared in almost quantitative yields after catalyst removal and the solvent evaporation. The ethylene-diamine-*N*,*N*′-bis(methylphosphonic acid) 31b and, partially, propylene-diamine-*N*,*N*′-bis(methylphosphonic acid) 32b precipitated during the reaction. For these two compounds, the catalyst on the filter was washed with water and 5% aq. NH_3_ to dissolve the amino acids. These products were re-precipitated in their zwitter-ionic form by acidification of the alkaline solutions by aq. HCl.

**Scheme 7:**
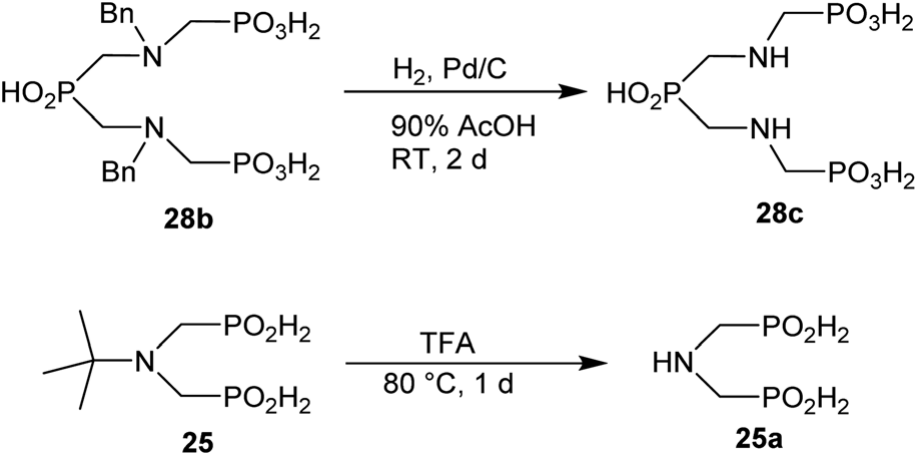
Examples of the amine group deprotection.

**Scheme 8:**
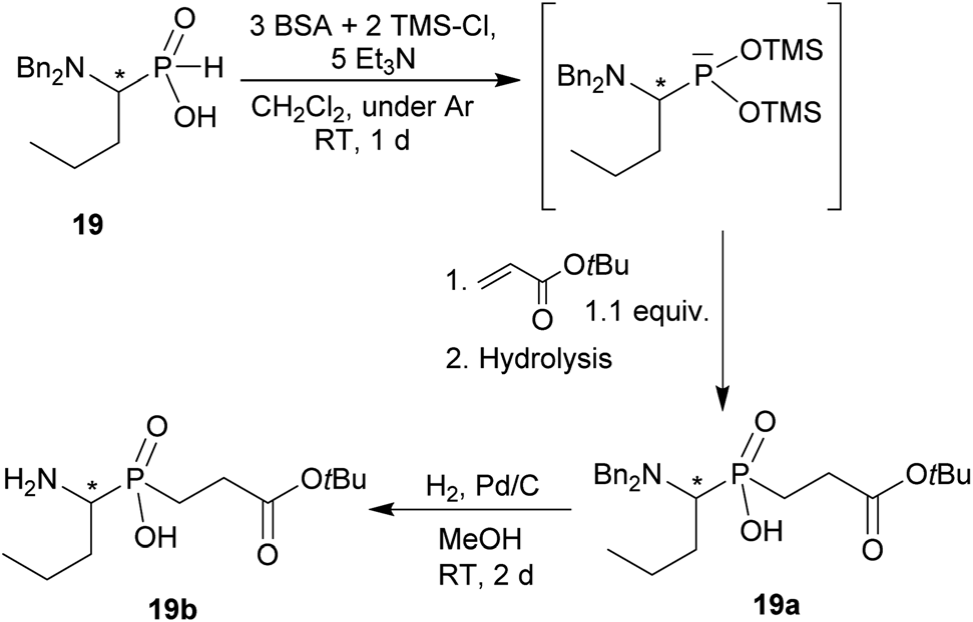
Example of further reaction of P–H bond of AHPA.

**Scheme 9:**
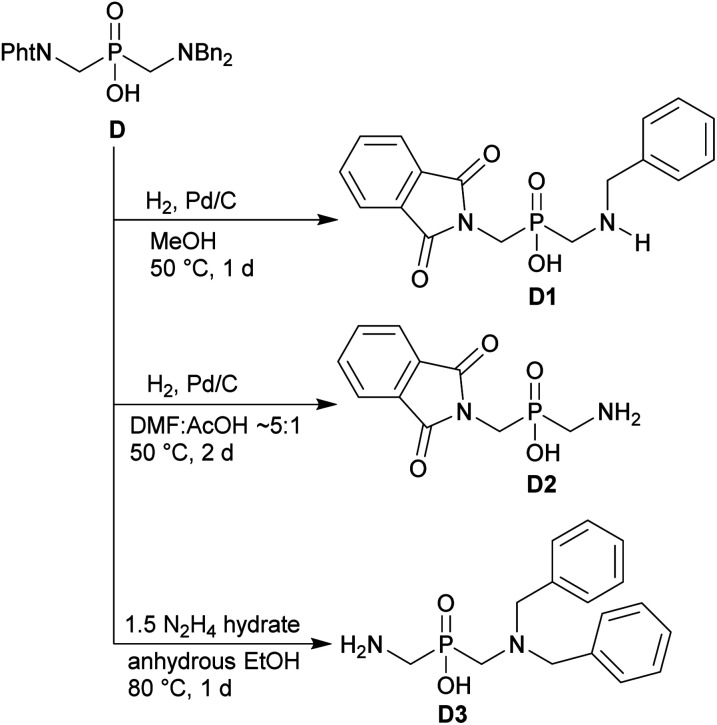
Orthogonal protection of the amine groups in (NH_2_CH_2_)_2_PO_2_H.

The phthaloyl, benzyl or *t*-butyl groups are amine protective groups and the prepared compounds can be used as precursors for synthesis of phosphinic acid derivatives with free primary or secondary amine groups. To illustrate such possibilities, the groups were removed by common methods (Schemes 7–9). Hydrogenolysis of *N*-benzylated derivative 28b in aq. AcOH led to a bis(phosphonomethyl-aminomethyl)phosphinic acid 28c in a quantitative yield (Scheme 7). Removal of *t*-Bu group from compound 25 in hot trifluoroacetic acid gave *H*-phosphinic acid analogue of H_2_ida 25a (Scheme 7). A mixture of triethylamine, trimethylsilylchloride and *N*,*O*-bis(trimethylsilyl)acetamide converted *H*-phosphinic acid 19 to trivalent phosphorus intermediate (Scheme 8). The intermediate reacted with *t*-butyl acrylate to give derivative 19a in a moderate yield. The carboxylic ester protected compound 19b with free amino group was prepared by removal of the *N*-benzyl groups of 19a in a quantitative yield. Orthogonally *N*-protected compound D was used to prepare compounds D2 and D3 by Pd-catalyzed hydrogenolysis and hydrazine-mediated phthaloyl removal, respectively (Scheme 9). Surprisingly, hydrogenation of the compound D in common solvents (MeOH, EtOH, AcOH or their mixtures with water) led to *N*-monobenzylated compound D1 as it is not soluble in the solvents.

### Solid-state structures of 1-aminoalkyl-*H*-phosphinic acids

Single crystals of fifteen 1-aminoalkyl-*H*-phosphinic acids and two bis-substituted phosphinic acids were obtained and their solid-state structures were determined (experimental crystallographic data, refinement results and CCDC reference numbers are given in Table S5,† and figures of their molecular structures are shown in the in ESI† together with characterization data of the compounds). Except one, all structures are zwitter-ionic and, always, the phosphinic acid groups are deprotonated and the amine groups are protonated. The exception is adamantylammonium salt of 18 where the phosphinate group is deprotonated and the proton is bound to amino group of adamantylamine. Two phosphinic acid groups are present in 22·H_2_O and 25 and only one of them is deprotonated. Crystal structure of the C·PhPO_3_H_2_ adduct points to a high acidity of the phosphinic acid group as phenylphosphonic acid is fully protonated and serves as a template for hydrogen bond network. If carboxylic groups are present (compounds 10, 11, 12 and 13·0.25H_2_O) they are always protonated.

**Fig. 3:**
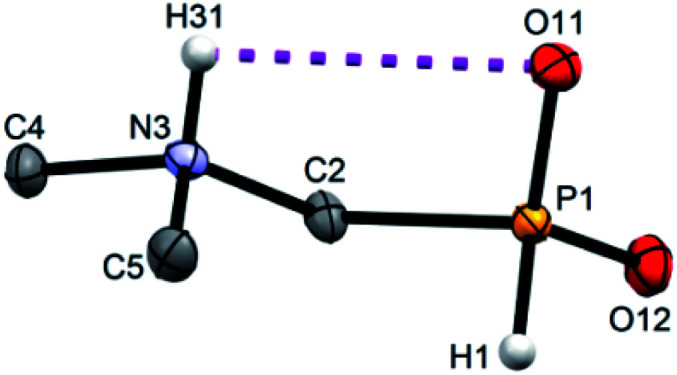
Molecular structure of 2 found in its crystal structure. Magenta dashed line shows intramolecular hydrogen bond. Carbon-bound hydrogen atoms are omitted for sake of clarity.

**Fig. 4:**
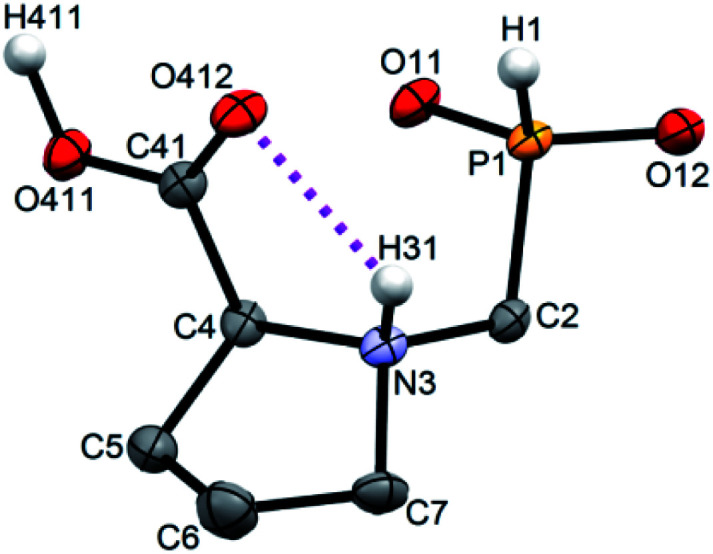
Molecular structure of 13 found in the crystal structure of 13·0.25H_2_O. Magenta dashed line shows intramolecular hydrogen bond. Carbon-bound hydrogen atoms are omitted for sake of clarity.

**Fig. 5:**
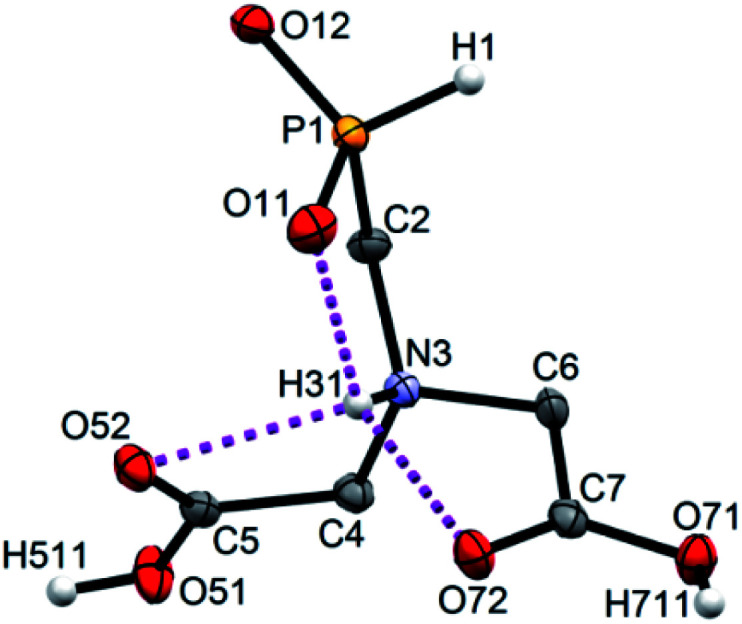
Molecular structure of 12 found in its crystal structure. Magenta dashed lines show intramolecular hydrogen bonds. Carbon-bound hydrogen atoms are omitted for sake of clarity.

In the most of cases, deprotonated phosphinate group is turned in such a way which enables a close intramolecular ^+^N–H31⋯O11^−^–P interaction (see Fig. 3 as an example) with N3⋯O11 distances ranging in 2.80–3.21 Å (Table S6†). However, the ⋯H–N–C–P–O⋯ ring is very sterically demanding and the N–H⋯O angles are far from optimum ones (range 79–120°, Table S6†). In the cases of 10, 11 and 13·0.25H_2_O (see Fig. 4 as an example), where one carboxylic acid moiety is present, the phosphinate group is not involved in the intramolecular interaction with the protonated amino group. In these cases, somewhat surprisingly, the carbonyl oxygen atoms of the protonated carboxylate groups interact with the protonated amine instead, probably due to a shorter possible distance (N3⋯O11 distances in a range 2.70–2.76 Å with N–H⋯O angles 94–113°; Table S6†). In the case of 12, oxygen atoms of phosphinate as well as both carboxylic groups are involved in intramolecular hydrogen bonding (Fig. 5). The hydrogen-bond system has a longer N3⋯O(phosphinate) distance but with a more convenient N–H⋯O angle if compared to those of carboxylic acid oxygen atom (2.82 Å *vs.* 2.70–2.73 Å and 120° *vs.* 104–110°, respectively; Table S6†).

In few cases, such orientation of substituents on the phosphinate group is not found or the interaction is very weak (*e.g.* in 1, 17·2H_2_O or BnNHCH_2_PO_2_H_2_) as a result of the phosphinate group involvement in the intermolecular hydrogen bond system. In almost all cases, the protonated amino group is involved in a short intermolecular hydrogen bond interaction with phosphinate oxygen atom of neighbouring molecule with *d*(N3⋯O(phosphinate)^#^) = 2.65–2.75 Å (Table S7†). Such interaction was not found only for 12 and C·PhPO_3_H_2_. In the structure of 12, protonated amino group is fully wrapped by three oxygen atoms from the pendant acid moieties (one phosphinate and two carboxylic acid groups, see Fig. 5) and, thus, cannot participate in intermolecular bonding. In the structure of C·PhPO_3_H_2_, phenylphosphonic acid serves as an acceptor of the hydrogen atom from compound C. Beside these interactions, further more or less complicated system of hydrogen bonds is formed involving also water molecules of crystallization or other molecules present in the crystal structures (Table S7†). Compounds 13·0.25H_2_O, (AdNH_3_)^+^(18)^−^·H_2_O and 20·MeOH contain a carbon chirality centre and crystallize in their racemic form as it is required by centrosymmetric space groups *P*2_1_/*n* and *P*1̄, respectively. Surprisingly, non-chiral compounds 10 and 22·H_2_O crystallize (as the only ones among the crystallographically characterized compounds) in the chiral groups *P*2_1_2_1_2_1_ and *P*2_1_, respectively. In these cases, the chirality is induced by four different substituent bound to the protonated amino group (in the case of 22·H_2_O, the methyl-*H*-phosphinic acid groups are dissimilar as one of them is protonated and the other one not). Polarity of the whole crystal is caused by an oriented chain of hydrogen bonds.

## Discussion

**Scheme 10:**
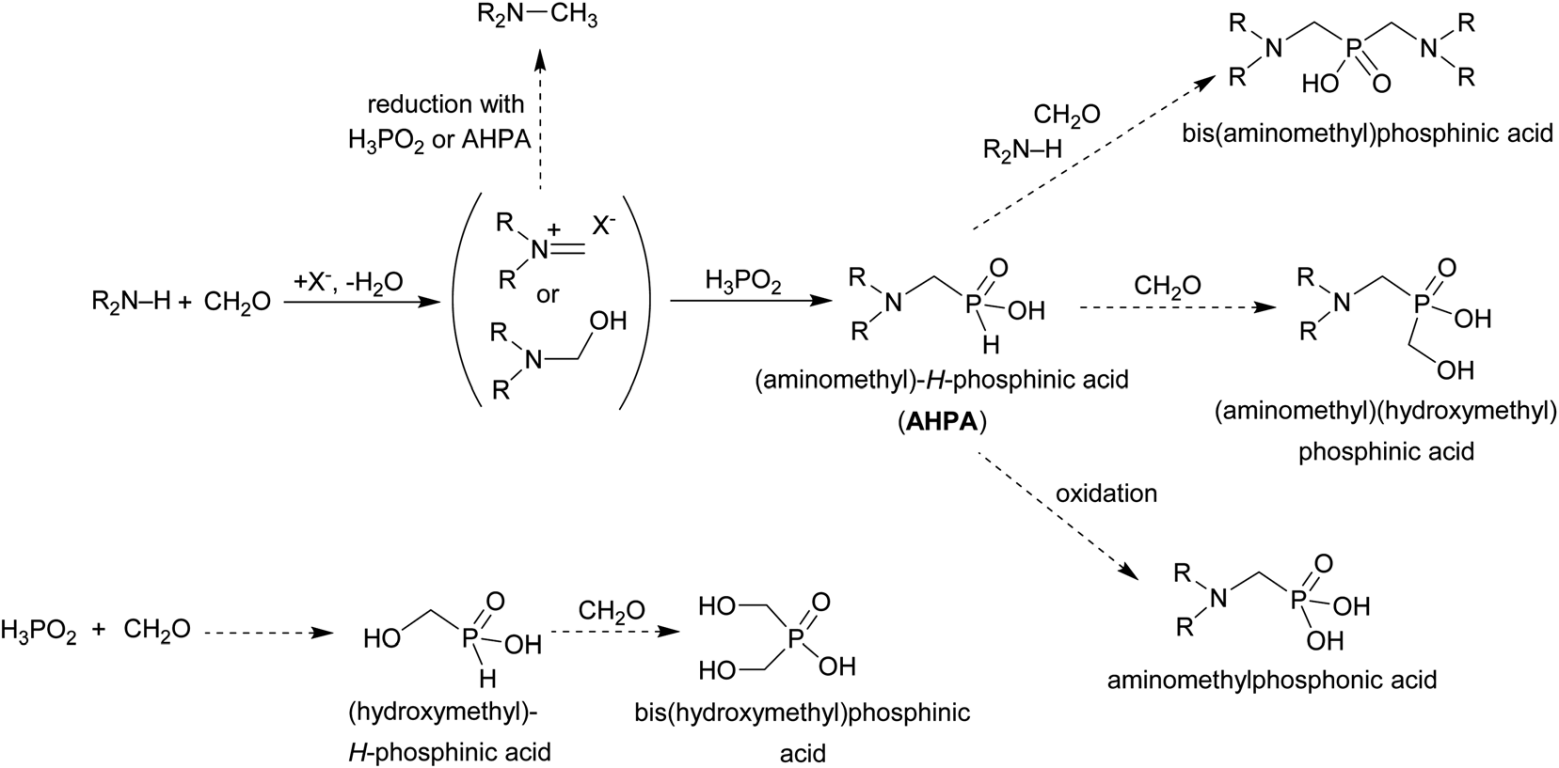
Phospha-Mannich reaction of an amine, formaldehyde and hypophosphorous acid. Non-desired side reactions are shown with dashed arrows.

Hypophosphorous acid has two reactive P–H bonds which greatly differ in reactivity. The phospha-Mannich reaction of a secondary amine, an aldehyde and H_3_PO_2_ with molar ratio 1 : 1 : 1 mostly takes place according to Scheme 10. The (1-aminoalkyl)-*H*-phosphinic acids (AHPA's) are desired products of the reaction. The main by-products observed in the reactions are (i) *N*-alkylated amines (product of reductive alkylation of the amine by the aldehyde connected with oxidation of H_3_PO_2_ or any *H*-phosphinic acid), (ii) (1-hydroxyalkyl)phosphinic acids (product of addition of H_3_PO_2_ or any *H*-phosphinic acid on the aldehyde) and/or (iii) phosphonic acids (products of oxidation of P–H bond in H_3_PO_2_ or any *H*-phosphinic acid). If an excess of the amine and the aldehyde is used under forced conditions, the second P–H bond can also react and (iv) the reaction leads to bis(1-aminoalkyl)phosphinic acids. Products with the C–P–C group can be formed even with equimolar amounts of reactants. Generally, the desired (1-aminoalkyl)-*H*-phosphinic or bis(1-aminoalkyl)phosphinic acids (depending on molar ratio of reactants) might be a minor component of such reaction mixtures and the mixtures are hardly separable. So, the main task is to find out reaction conditions which the final reaction mixtures will contain as a low number and amount of the by-products as possible. It would facilitate purification of the target compound. There is no general method which can easily lead to the AHPA's with a good purity and in high yields. Mostly, their syntheses are accompanied with a number of by-products as shown in Scheme 10.

Here, we describe simple preparations of the *H*-phosphinic acids if they are derived from secondary amines. Hypophosphorous acid is used as a cheap reagent and the reaction in acetic acid is easy to run and gives, generally, more clear reaction mixtures than preparations in the other solvents. A small amount of water (introduced by utilization of commercial aqueous solution of H_3_PO_2_) does not disturb the reaction. However, presence of higher amount of water slows down the reaction and changes its outcome to more rich mixtures. The H_3_PO_3_ and alkyl/aryl-*H*-phosphinic acids are significantly less reactive than H_3_PO_2_. There is generally accepted that trivalent tautomers of pentavalent compounds with H–P bond are reactive forms in most of phosphorus-centered reactions of the compounds. Then, the highest reactivity of H_3_PO_2_ and the observed changes in reactivity of the *H*-phosphinic acids might be a consequence of different stabilization of these reactive trivalent phosphorus tautomers, as it is changed with different phosphorus substituents.^49^ This “P–H bond” reactivity seems to be better distinguished in acetic acid than in the other solvents. The reactivity of the “second” P–H bond is decreased and, therefore, formation of compounds with C–P–C is efficiently suppressed. Compounds as (HOCH_2_)(R_2_NCH_2_)PO_2_H and (R_2_NCH_2_)_2_PO_2_H are common by-products in phospha-Mannich reactions of H_3_PO_2_ (Scheme 10). Formation of these by-products is promoted by a presence of a strong acid (*e.g.* HCl), by a higher temperature and/or a long reaction time. It is also supported by a formation of entropically not favoured cyclic aminophosphinic acids with a [–N(R)–CH_2_–PO_2_H–CH_2_–]_2_ eight-membered ring in reaction of primary amines, formaldehyde and H_3_PO_2_ in azeotropic aqueous HCl.^50^ Addition of excess of formaldehyde and/or excess of H_3_PO_2_ under the used conditions did not alter high yields and purity of the desired 1-aminoalkyl-*H*-phosphinic acids.

The most common and the most problematic side reaction in phospha-Mannich reactions with formaldehyde is formation of *N*-methylated by-product(s).^51^ We observed that this side reaction was completely suppressed for some reactants and, somewhat surprisingly, extent of the reaction seems to depend on the amine basicity. More basic (*i.e.* more nucleophilic) amines reacted to the desired aminomethyl-*H*-phosphinic acids with no *N*-methylation. With the less nucleophilic amines, the reductive *N*-methylation and simultaneous oxidation of H_3_PO_2_ to H_3_PO_3_ was the preferred reaction. Basicity/acidity of the amines where the reaction direction seems to be changed can be estimated close to a value of the amine protonation constant log *K*_a_ about 7–8 (Tables 1 and 3–5). The dependence on amine log *K*_a_ is more easily understandable for the secondary amines as it is given simply by electronic properties of the amine substituents. Reactivity of the primary amines could be explained in more intricate way. Phosphinic acid group is an electron-withdrawing group and decreases basicity of the *α*-amine groups by about 1.5–2.5 orders of magnitude.^52^ After the first substitution on the primary amines, basicity of the secondary amines in the “mono-substituted” RNH–CH_2_–PO_2_H_2_ is significantly decreased and reductive *N*-methylation becomes a more important reaction. Only strongly electron-donating groups as alkyl substituents (*e.g.* cyclohexyl) or methylphosphonate group (it also increases basicity of the *α*-amine group)^52*b*^ are able to off-set the basicity decrease caused by the methyl-*H*-phosphinic acid group. Therefore, only basic primary amines (log *K*_a_ > ∼10) gave expected bis(methyl-*H*-phosphinic acids). Such behaviour is in accord with a mechanism which we can suggest for the phospha-Mannich reaction under conditions used in this work (see below). Therefore, utilization of AcOH as a solvent is not generally suitable for synthesis of AHPA's derived from primary amines. The bis(AHPA's) were obtained only for amines those basicity was increased by electron-donating groups as alkyls or methylphosphonate groups. The Bn–N(CH_2_PO_2_H_2_)_2_ (compound 22) has been easily obtained in a moderate yield in reaction of BnNH_2_ with a high excess of H_3_PO_2_ and formaldehyde in water at slightly increased temperature^31*c*^ and, therefore, such reaction conditions may be also suitable for synthesis of bis(AHPA's) derived from other primary amines.

**Scheme 11:**
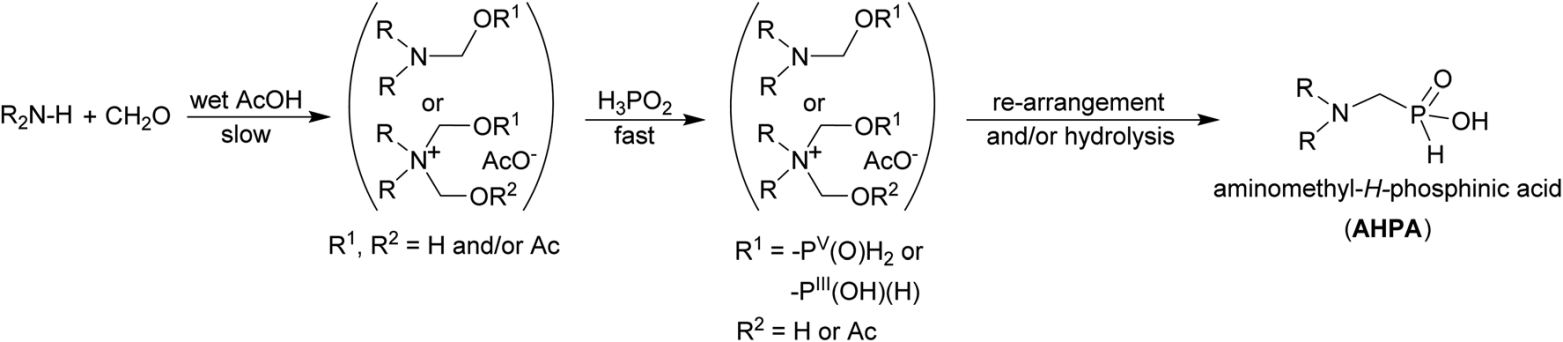
Suggested reaction mechanism for reaction of secondary amine, H_3_PO_2_ and formaldehyde in wet acetic acid.

To elucidate mechanism of the reaction in the acetic acid, a model secondary amine, Me_2_NH, was used. It relatively quickly react with formaldehyde and the solution is slowly evolving into mixtures of several products: Me_2_N–CH_2_OH/Me_2_N–CH_2_OAc, (Me_2_N=CH_2_)^+^, and [Me_2_N(CH_2_OH)_2_]^+^/[Me_2_N(CH_2_OAc)_2_]^+^. Under the used conditions, the N–(CH_2_OH)_1,2_ fragments should be probably acetylated as such esters are relatively stable and even their isolation was described.^53^ They were also used in Arbuzov reaction to get compounds with N–C–P fragment.^54^ As the Me_2_N–CH_2_OH and (Me_2_N=CH_2_)^+^ species have been suggested as intermediates in Kabachnik–Fields (K–F) reaction in organic solvents,^55–58^ the reaction in acetic acid follows a generally accepted mechanism of the K–F reaction. In presence of even a small amount of water, the iminium cation is not stable and hydrolyses to the Me_2_N–CH_2_OR species. The formation of *N*-methylated by-products is probably suppressed under conditions where the (Me_2_N=CH_2_)^+^ cation is not present in the reaction mixture. If pure aminal (Me_2_N)_2_CH_2_ was dissolved in AcOH, it quickly dissociated to Me_2_NH and the iminium cation, (Me_2_N=CH_2_)^+^. In the presence of a small amount of water, the cation further reacted to the (acetylated) *N*-hydroxymethylated species. Reaction of primary amines with formaldehyde gave their cyclic triazine trimers which are further decomposed and, thus, it may also contribute to less clear reactions of primary amines. The ammonium [Me_2_N(CH_2_OR)_2_]^+^ cation seems to be the most stable species with an excess of formaldehyde. We can speculate that it is, probably stabilized as the acetyl ester, the most important reaction intermediate. The ammonium cations will be more stable for the more basic (=nucleophilic) amines and, once formed, the cations would be also less prone to the reduction to methyl group. Presence and reactivity of the cation was proven by observation (Fig. S19–S21†) of its *H*-phosphinic acids derivative, [Me_2_N(CH_2_OR)(CH_2_PO_2_H_2_)]^+^. The species might be considered as another reaction intermediate. Some amount of the species remained in the solution even after several hours but, anyway, it was completely hydrolysed to 2 after addition of excess of water (Fig. S22†). Thus generally, conversions to the final AHPA's were almost quantitative. During the reaction, the P–C bond is probably formed by re-arrangement of transient esters/phosphites formally derived from reaction of H_2_P(O)(OH) or H–P(OH)_2_, respectively, with any of the *N*-hydroxymethylated amine species. However despite the discussion above, the iminium cation cannot be fully excluded as an intermediate. The differences in reactivity between H_3_PO_2_ on one side, and H_3_PO_3_ or AHPA on the other side, can be then explained by the most easy formation of the esters/phosphites derived from H_3_PO_2_/H–P(OH)_2_, respectively. In addition, a small amount of water in AcOH may also help to stabilize the tautomeric P(iii) form of H_3_PO_2_, H–P(OH)_2_;^52^ such trivalent phosphorus tautomers are generally supposed to be reactive phosphorus intermediates in phospha-Mannich reaction. The suggested mechanism is shown in the Scheme 11.

The reductive *N*-methylation takes place mainly if the iminium cations/Schiff bases are present in the mixture after reaction of the amines with formaldehyde. It happens in the presence of a strong acid as HCl and also for less basic amines, *e.g.* for primary amines after attachment of the first methyl-*H*-phosphinic acid group. The low basicity of some amine groups in the polyamines could be also suggested as a cause of the easy polyamine *N*-methylation. Basicity of the second/third amine groups of the ethylene-diamine/triethylene-diamine derivatives, respectively, is significantly decreased (down to log *K*_a_ 5–7) in comparison with the first one(s).^40^ Linear polyamines with propylene or hexylene chains, and thus more distant secondary amine groups, are more basic and reacted as desired. An alternative explanation of the extensive *N*-methylation of polyamines with ethylene-diamine fragment is an easy formation of five-membered cyclic aminals. The methylene group in the aminals can be more easily reduced to methyl group. Such aminals, derived from tacn and cyclam, are formed very quickly. The cyclam bis(aminal) is so stable that it is fully unreactive under conditions used in this study.^48,59^ The tacn aminal reacts, in presence of the excess of formaldehyde, with an excess of H_3_PO_2_ to give a 1,4,7-tris(methyl-*H*-phosphinic acid) tacn derivative as a major component of the mixture. The compound was isolated in about twice higher yield than from reaction of tacn, formaldehyde and H_3_PO_2_ in 1 : 1 aq. HCl.^31*e*^ It should be noticed that a similar reaction of ethyl ethylphosphinate with 1,4,7-tris(methoxymethyl)-tacn derivative in benzene produced a 1,4,7-tris[methyl(ethyl)phosphinic acid] derivative of tacn in a low yield.^60^

Arylamines are probably not enough basic to give AHPA's with a high conversion and, in addition, the corresponding AHPA's are not stable. Similarly, amides and heterocyclic amines do not react under conditions used in this work. Probably, the ammonium intermediate cannot be formed with these amines or amides. It is somewhat surprising for amides as acetylated aminals derived from aromatic aldehydes (*e.g.* PhCH(NHAc)_2_) can react with H_3_PO_2_ in acetic acid with formation of *N*-acetylated AHPA's.^61^ Partially *N*-substituted or -protected cyclic amines also do not react clearly under the used conditions. The reason might be preferred conformation of the macrocycles in solution and/or the presence of intramolecular hydrogen bond system. Presence of β-hydroxy group(s) in the amines (*e.g.* in diethanolamine) leads to bis-substitution on the phosphorus atom. It could be a consequence of a formation of intramolecular cyclic ester. It changes reactivity of the phosphorus-containing moiety and reaction of the “second” P–H bond is easier. Thus, presence of some substituents (hydroxy, amine and/or amide groups) in the β-position to the amine group can cause that the reaction does not proceed as expected.

Reactivity of different aldehydes probably depends on a local electron density and their bulkiness. Formaldehyde as the simplest aldehyde afforded AHPA's in high conversions and yields. Slightly lower conversion and yield were achieved with acetaldehyde (compound 18) and both were more decreased with *n*-butyraldehyde (compound 19). Bulky pivaldehyde did not react at all. Electronically poor aldehydes as benzaldehyde and trifluoro-acetaldehyde prefer *P*-hydroxyalkylation to give 1-hydroxyalkyl-*H*-phosphinic acids. In this case, originally formed AHPA's might be decomposed with regeneration of the amine and formation of 1-hydroxyalkyl-*H*-phosphinic acids. Thus, the reaction conditions are not generally suitable for sterically hindered and electronically poor aldehydes.

Carboxylic or phosphonic amino acids contain a relatively basic amine group. Their reaction with paraformaldehyde and H_3_PO_2_ gave good yields of multi-acidic derivatives 10–12 and 27–30 where some uncommon combinations of the acidic groups (carboxylic/phosphonic/phosphinic acid groups) are present. The reactivity of these amino acids also points to significance of basicity of the amine group as the reactions were relatively clean and extent of the *N*-methylation was small. Compound 12 has been prepared before by reaction of H_3_PO_2_ with H_2_ida and formaldehyde in EtOH or in water but the isolated yields were 70% or 35%, respectively.^34*b*,43^

As some amine substituents can serve as amine protection, usefulness of the synthesized amino-*H*-phosphinic acids was exemplified on preparations of some new compounds with secondary or primary amine groups. Phosphonic acid complexonates with secondary amines 31b–35b were prepared after oxidation of 31–35 and hydrogenation of the intermediate *N*-benzylated phosphonic acid derivatives 31a–35a. Easy *N*-debenzylation of compound 28b gave phosphinic-bis(amino-phosphonic acid) derivative 28c. Unknown *H*-phosphinic acid H_2_ida analogue 25a was obtained by acidic removal of *t*-butyl group in 25. The hydrogenative debenzylation was even possible for Bn_2_N– group leading to phtaloyl-monoprotected bis(aminomethyl)phosphinic acid D2. This *N*-debenzylation is probably feasible due to the closely located electron-withdrawing phosphinic acid group. However, due to the solubility issues, *N*-monobenzylated bis(aminomethyl)phosphinic aid D1 precipitated from the solution if common solvents for the hydrogenation reaction were used. Alternatively, the phthaloyl group was conventionally removed by hydrazine hydrate to get derivative D3 of the same amino acid monoprotected by *N*-dibenzyl moiety. Pentavalent phosphorus of *H*-phosphinic acid 19 was converted to P(iii) with a mixture of silylating agents (Me_3_SiCl and BSA) and Et_3_N, and the silylated intermediate was reacted with *t*-butyl acrylate to obtain phosphinic acid 19a. This acid was easily *N*-debenzylated to yield compound 19b with free primary amine and protected carboxylate group. The compounds with the free amine group are examples of amino phosphinic acid building blocks which can be utilized in syntheses of phosphinic acid oligopeptides.^6–10^

The structures of the largest set of amino-alkylphosphinic acids show that, in the solid state, the most common structural motif is intramolecular hydrogen bond formation between protonated amino group and the phosphinate oxygen atom. When carboxymethyl substituent is bound to the central nitrogen atom, somewhat unexpected hydrogen bond interaction between protonated carboxyl group(s) and the central amino group was found. Such interaction is preferred over phosphinate interaction due to a shorter distances if compared to distances between nitrogen and phosphinate oxygen atoms; however, the cycles with the N–H⋯O fragment formed by intramolecular interactions are strained due to non-optimal hydrogen-bond angle (N3–H31⋯O11) ranging in interval of 79–120°. Intermolecular hydrogen bonds are also important to stabilize the structures as it is clearly seen from short distances between amino groups and oxygen atoms from neighbouring molecules (Table S7†). In general, the structural data confirm necessity of hydrogen bonds to stabilize solid-state structures of amino acids derived from phosphoric acid. Protonation scheme in the compounds containing both phosphinic acid and carboxylic group also agrees with solution thermodynamic data, *i.e.* acidity of the phosphinic acids is higher than that of carboxylic acids.

## Experimental section

### General

The commercially available (Fluka, Aldrich, CheMatech, Strem, Fluorochem) chemicals and solvents (Lachner or Penta, CZ) had synthetic purity and were used as received, if not stated otherwise. Deuterated solvents were bought from Armar or Sigma. The compounds (PhtNCH_2_CH_2_)_2_NH,^62^ PhtNCH_2_PO_2_H_2_,^63^ HO_2_CCH_2_CH_2_PO_2_H_2_,^35*a*^ BnNHCH_2_PO_3_H_2_ (ref. 64) and 1,3,5-tribenzyl-1,3,5-triazacyclohexane^65^ were obtained by literature methods. Hydrochlorides of *trans*-Cbz_2_cyclen,^66^*trans*-Me_2_cyclen^67^ and 1,4,8-Me_3_cyclam,^68^ were prepared as previously reported and the free bases were obtained after participation between dichloromethane and aq. NaOH (pH > 12). The BnNHCH_2_PO_2_H_2_,^69^*N*,*N*′-dibenzyl-alkylene-diamines^70^ and *N*,*N*′′-dibenzyl-dialkylene-triamines were prepared by an improved literature procedures (see ESI, Tables S1 and S2†). Commercial phenylacetaldehyde stabilized with 0.01% citric acid was redistilled at reduced pressure (*T*_b_ ∼ 82 °C, *p* ∼ 10 torr). Strong cation exchanger resin Dowex 50 was always used in H^+^-form, if not stated otherwise. Deionized water (Millipore) was used throughout the work. The 1D/2D NMR experiments (chemical shift in ppm, coupling constants in Hz) were performed on Bruker Avance III with cryo probe (14.3 T, 600 MHz; ^1^H and ^13^C{^1^H}), Varian VNMRS300 (7.0 T, 300 MHz; ^1^H, ^19^F, ^31^P and ^31^P{^1^H}) or on Bruker Avance III HD (9.4 T, 400 MHz; ^1^H, ^13^C{^1^H}, ^19^F, ^31^P and ^31^P{^1^H}) spectrometers using 5 mm sample tubes. All NMR spectra were collected at 25.0 °C unless stated otherwise. The ^31^P and ^19^F NMR spectra were referenced to external 85% aq. H_3_PO_4_ (*δ*_P_ 0.0 ppm) and to 0.1 M TFA in D_2_O (*δ*_F_ −75.51 ppm), respectively, in NMR coaxial insert tubes. The ^1^H and ^13^C{^1^H} NMR spectra were referenced to external or internal *t*-BuOH (*δ*_H_ 1.25 ppm, *δ*_C_ 30.3 ppm), CDCl_3_ (*δ*_H_ 7.26 ppm, *δ*_C_ 77.0 ppm), AcOH-*d*_4_ (*δ*_H_ 2.05 ppm, *δ*_C_ 20.0 ppm), MeOH-*d*_4_ (*δ*_H_ 3.33 ppm, *δ*_C_ 49.0 ppm), or DMSO-*d*_6_ (*δ*_H_ 2.50 ppm, *δ*_C_ 39.5 ppm). The pD values were measured by an electrode system calibrated with standard buffers, and the read pH values were corrected according to pD = pH + 0.4. The pD was adjusted with DCl or NaOD solutions in D_2_O. The ESI-MS spectra were recorded on Bruker Esquire 3000 spectrometer with ion-trap detection in negative or positive modes. The HR-MS were acquired on LC-MS system consisted from Acquity UPLC (Waters) and Velos Pro Orbitrap Elite with a HESI probe (Thermo Scientific). Thin-layer chromatography (TLC) was performed on silica ^60^F_254_ TLC sheets (Merck) with UV detection (254 nm) or by spraying with 0.1% ninhydrin solution in EtOH coupled with mild heating. Flash reversed-phase column chromatography (C18) with UV detector was carried out on Sepachore Flash System X50 apparatus (Büchi). Elemental analyses were performed at the Institute of Organic Chemistry and Biochemistry of the Czech Academy of Science (Prague, Czech Republic) and are presented in the format: found (calculated). Complete characterization data (^1^H, ^13^C{^1^H}, ^19^F, and ^31^P NMR; MS, HR-MS, TLC, elemental analyses) of the synthesized compounds are given in ESI.†

The diffraction data were collected at 120 K for [H_3_(*N*,*N*′′-dibenzyl)-diethylene-triamine]Cl_3_, 1, 12, 13·0.25H_2_O, 17·2H_2_O, BnNHCH_2_PO_2_H_2_, C·PhPO_3_H_2_ and D, or at 150 K (all other structures) on Nonius KappaCCD diffractometer equipped a cooling system (Cryostream Cooler, Oxford Cryosystem). The Bruker APEX-II CCD detector with monochromatized Mo-Kα radiation (*λ* 0.71073 Å) was used for 2, 5, 10, 11, (AdNH_3_)^+^(18)^−^·H_2_O, 22·H_2_O, BnNHCH_2_PO_2_H_2_ and C·PhPO_3_H_2_. The Bruker D8 VENTURE Kappa Duo PHOTON100 diffractometer with IμS micro-focus sealed tube was used for 12, 13·0.25H_2_O, 17·2H_2_O, D with Cu-Kα (*λ* 1.54178 Å) radiation or for [H_3_(*N*,*N*′′-dibenzyl)-diethylene-triamine]Cl_3_, 1, 4·2H_2_O, 8·H_2_O, 20·MeOH, 25 with Mo-Kα (*λ* 0.71073 Å) radiation. Data were analysed using the SAINT (Bruker AXS Inc.) software package. Data were corrected for absorption effects using the multi-scan method (SADABS). All structures were solved by direct methods (SHELXT2014)^71^ and refined using full-matrix least-squares techniques (SHELXL2014).^72^ All non-hydrogen atoms were refined anisotropically. All hydrogen atoms were found in the difference density map. However, the appropriate numbers of hydrogen atoms bound to carbon atoms were fixed in theoretical positions using *U*_eq_(H) = 1.2 *U*_eq_(C) to keep a number of parameters low, and only hydrogen atoms bound to heteroatoms (N, O, P) were fully refined. The ESI† brings detailed information on refinement of individual structures (Table S5†) and figures of molecular structures of all structurally characterized compounds.

It has to be noticed that the syntheses of most of the compounds prepared in this work were scaled up to a gram scale in repeated experiments with no significant decrease of isolated yields and, sometimes, the yields were even higher than those described in the tables and in the text due to a lower relative loss of the materials.

### NMR experiments

The conversions were determined by integration of ^31^P NMR signals and their standard errors are estimated to be ∼5%. The quantification is valid only if no ^31^P-containing precipitate was formed during the reactions. The estimated values were reproducible.

#### Mechanistic investigations

The experiment can be exemplified as follows: in NMR tube, 50% aq. H_3_PO_2_ (25 μL, 0.19 mmol, 1 equiv.), paraformaldehyde (8.5 mg, 0.28 mmol, 1.5 equiv.) and 40% aq. Me_2_NH (24 μL, 0.19 mmol, 1 equiv.) were mixed with AcOH-*d*_4_ (0.40 mL). The mixture was heated at 40 °C and NMR spectra were acquired periodically. Analogous experiments were done with gradual addition of the starting materials in various orders. More specific instructions are given in appropriate figure captions (see ESI†). Other examples of NMR experiments: (i) (Me_2_N=CH_2_)^+^Cl^−^ (23 mg, 0.19 mmol, 1 equiv.) or (Me_2_N)_2_CH_2_ (25 μL, 0.19 mmol, 1 equiv.), solid H_3_PO_2_ (12.5 mg, 0.19 mmol, 1 equiv.) and D_2_O (18 μL, 0.90 mmol, 4 equiv.) were dissolved in AcOH-*d*_4_ (0.40 mL); (ii) Bn_2_en (29 μL, 0.12 mmol, 1 equiv.) or piperazine hexahydrate (24 mg, 0.12 mmol, 1 equiv.), paraformaldehyde (15 mg, 0.50 mmol, 4 equiv.) or benzylamine (13 μL, 0.12 mmol, 1 equiv.) and 50% aq. H_3_PO_2_ (16 mg, 0.12 mmol, 1 equiv.) were used. The solutions were heated at 40 °C if not stated otherwise. Standard ^1^H, ^13^C{^1^H} and ^31^P NMR spectra were acquired at 25 °C, if not stated otherwise. Details on the NMR experiments are given in captions of the appropriate figures in ESI.†

### General procedure for syntheses with secondary amines (Table 1, compounds 1–8, 10, 11, 13, 16, and 17)

In 4 mL vial, starting amine or amide (1.0 mmol, 1 equiv.), paraformaldehyde (60 mg, 2.0 mmol, 2 equiv.), and weighted 50% aq. H_3_PO_2_ (145 mg, 1.1 mmol, 1.1 equiv.) were mixed with glacial AcOH (2 mL). The suspension was stirred and heated up to 40 °C for 1 day and conversion was determined by ^31^P NMR. In most cases, reaction was finished after several hours (∼5 h) and no more changes were observed at 24 h time point. Then, solvents were removed on rotary evaporator and the oily residue was purified on Dowex 50 (3 × 10 cm bed). The column was washed with water. Non-aminic compounds were eluted off first. Sometimes, a part of products were eluted off already with water with only small delay behind the solvent front (2–4 column volumes were used) (Procedure A). Products were generally eluted off with 10% aq. pyridine (Procedure B). Fractions containing pure product (TLC and/or ^31^P NMR) were combined and solvents were evaporated in vacuum giving a pure oily product. Some oils solidified upon standing or after a trituration with a proper solvent (see ESI†). Compounds 9, 12, 14a, 14b, and 15b were prepared by modified procedures, and these special preparation, purification and isolation procedures are given in ESI.†

### General procedure for syntheses with higher aldehydes (Table 2, compounds 18–20)

In 4 mL vial, Bn_2_NH (192 μL, 1.0 mmol, 1 equiv.), aldehyde (2.0 mmol, 2 equiv.), and weighted 50% aq. H_3_PO_2_ (145 mg, 1.1 mmol, 1.1 equiv.) were dissolved in glacial AcOH (2 mL). The solutions were stirred and heated up to 60 °C for 2 d and reaction progress was followed by ^31^P NMR. To get higher conversion for compound 20, heating up to 80 °C for 3 d was used. Then, solvents were removed on rotary evaporator and the oily residue was purified on Dowex 50 (3 × 10 cm bed). The column was washed with water (50 mL), EtOH (100 mL) and the products were eluted with mixture 10% aq. pyridine : EtOH ∼ 3 : 1 (v/v). Combined fractions containing pure compounds were evaporated to dryness to get oily products (product 20 crystallized from hot MeOH solution upon cooling, see ESI†). Compounds 21a and 21b were prepared by modified procedures, and these special preparation, purification and isolation procedures are given in ESI.†

### General procedure for syntheses with primary amines (Table 3, compounds 22–27)

In 4 mL vial, primary amine (0.5 mmol, 1 equiv.), paraformaldehyde (33 mg, 1.3 mmol, 2.2 equiv.), and weighted 50% aq. H_3_PO_2_ (144 mg, 1.3 mmol, 2.2 equiv.) were mixed in glacial AcOH (2 mL). The suspensions were stirred at room temperature for 2 d and conversion was determined by ^31^P NMR. Then, the solutions were concentrated *in vacuo*. The oily residue was purified on Dowex 50 (3 × 10 cm bed). The products and simple phosphorus acids were eluted off with water (amines with one *H*-phosphinic acid group were retained on the column). After concentrating *in vacuo*, the oily residue was further purified on silica (50 g, 5 × 10 cm) using conc. aq. NH_3_ : EtOH 1 : 10 (v/v) as an eluent. Fractions (10 mL) containing pure product were combined and concentrated *in vacuo*. To get zwitter-ionic forms of the amino acids, ammonia form the oily residue was removed on Dowex 50 (3 × 10 cm bed) with water elution. The combined fractions were evaporated to dryness to get the pure products. Compound 27 was prepared by a modified procedure, see ESI.†

### General procedure for syntheses with phosphonomethylated secondary amines (Table 4, compounds 29 and 30)

In 25 mL flask, a secondary amine (1.0 mmol, 1 equiv.), paraformaldehyde (33 mg, 1.1 mmol, 1.1 equiv.), weighted 50% aq. H_3_PO_2_ (396 mg, 3.0 mmol, 3 equiv.) and anhydrous sodium acetate (164 mg or 328 mg, 2.0 or 4.0 mmol, for the glyphosate or H_4_idmpa, respectively; *i.e.* 2 equiv. per phosphonic group in the starting amine) were mixed in glacial AcOH (10 mL). The suspensions were stirred at room temperature for 2 d and then conversion was determined by ^31^P NMR. Then, the solids were filtered off and filtrates were concentrated *in vacuo*. The oily residue was triturated in MeOH (10 mL) using ultrasound. The solids were filtered and washed with Et_2_O (2 × 2 mL). The crude products were dissolved in water (5 mL) and the solution was loaded on Dowex 50 column (3 × 10 cm bed). The products were eluted off with water. Solvents were removed and the oily residue was re-chromatographed on Dowex 50 (3 × 10 cm bed) with water elution and 1–3 mL fractions were collected. Fractions containing product were combined, concentrated *in vacuo* and repeatedly purified on Dowex 50 as stated above (2–3 times). Finally, fractions with almost pure products were combined and concentrated *in vacuo*. For a special preparation, purification and isolation procedure of 28a and 28b, see ESI.†

### General procedure for synthesis with polyamines (Table 5, compounds 32–35)

In 25 mL flask, a secondary polyamine (0.25 mmol, 1 equiv.), paraformaldehyde (1.0 mmol/4 equiv. or 1.5 mmol/6 equiv. for diamines or triamines, respectively), and weighted 50% aq. H_3_PO_2_ (0.55 mmol/2.2 equiv. or 0.83 mmol/3.3 equiv. for diamines or triamines, respectively) were mixed in glacial AcOH (10 mL). The suspensions were stirred and heated up to 40 °C for 1–2 d and then conversion was determined by ^31^P NMR. Then, solvents were removed on rotary evaporator and the oily residue was purified on Dowex 50 (3 × 10 cm bed). The column was washed with water. Pure products were eluted off with mixture of 10% aq. pyridine with EtOH (∼3 : 1, v/v). Fractions containing pure product was combined and concentrated *in vacuo*. For a special purification procedure of 31 and 31-Me, see ESI.†

### General procedure for synthesis with oxidation of *H*-phosphinic acids and hydrogenation reactions (Table 6, compounds 31a–35a and 31b–35b)

In 25 mL flask, amino-*H*-phosphinic acid (Procedure C) or their ammonium salts (Procedure D) (0.25 mmol, 1 equiv.) were dissolved in water (10 mL) and the solutions were heated up to 65 °C. Then, hot aqueous solution of HgCl_2_ (102 mg, 0.4 mmol, 1.5 equiv. per phosphinic acid group, ∼10 mL) was added. The solutions were stirred at 65 °C for 1 d and completion of reaction was determined by ^31^P NMR. After cooling, the suspensions were filtered through 0.22 μm PVDF microfilter. For 31a, the microfilter was washed with 5% aq. NH_3_ (2 × 3 mL), the filtrate was concentrated *in vacuo* and the residue was re-dissolved in water. The solutions were saturated with H_2_S and precipitated HgS were filtered off on 0.22 μm PVDF microfilters. The clear filtrates were concentrated *in vacuo* to get pure amino phosphonic acids 31a–35a. After characterization, the oils were dissolved in 90% aq. AcOH, transferred to 25 mL flask and Pd/C (∼10 mg, 10% w/w) was added. The flask was flushed with hydrogen, connected to a hydrogen balloon and the suspensions were vigorously stirred at room temperature (75 °C for 33b) under (1 atm) H_2_ for 2 d. The suspensions were filtered through 0.22 μm PVDF microfilters, filtrates were evaporated *in vacuo* to thick oils. The oily residues were co-evaporated with toluene (2 × 5 mL) to remove acetic acid and then with water (5 mL) to remove toluene to give pure products. For compounds 31b and 32b (Procedure E), the microfilter was washed with 5% aq. NH_3_ (2 × 3 mL) and the solvent was removed in vacuum. This ammonium salts of 31b and 32b were converted to zwitter-ionic forms by dissolution in water (5 mL) and acidification of the solution with 3% aq. HCl to pH 1–2. After 1 d at 4 °C, the solid products were filtered off, and washed with acetone (2 mL), Et_2_O (2 × 3 mL) and dried on air to get white powders. For a special preparation, purification and isolation procedures of 28c, see ESI.†

## Conclusions

We introduced a novel protocol for synthesis of 1-aminoalkyl-*H*-phosphinic acids under mild conditions using wet acetic acid as a solvent and utilizing cheap H_3_PO_2_. Reactions are clean and almost no reductive methylation (coupled with P–H bond oxidation), *P*-hydroxymethylation or further reaction of the remaining P–H bond were observed. The proposed reaction conditions are usable for basic secondary amines with log *K*_a_ > 7–8, it means for most of dialkylamines. The *N*-methylation is the preferred reaction for polyamines with the ethylene-diamine fragment. Therefore, the reaction conditions are not suitable for modification of the common polyazamacrocycles. Utilization of primary amines is more restricted. Introduction of *N*-(alkyl-*H*-phosphinic acids) in the first step significantly decreased basicity of the formed secondary amine and *N*-methylation then becomes a strongly competing reaction for the second phospha-Mannich step. Only primary amines with a strong electron-donating group (*i.e.* adamantyl, cyclohexyl, phosphonomethyl) produced expected *N*,*N*-bis(methyl-*H*-phosphinic acids). The reaction cannot be controlled to get the *N*-mono(methyl-*H*-phosphinic acids) in a pure form. The reaction conditions are convenient for further modification of basic amino-methylphosphonic acids and several uncommon products with both *H*-phosphinic and phosphonic acid groups were obtained. Similarly to previous data on phospha-Mannich reaction, higher aldehydes are much less reactive and less useful. The *H*-phosphinic acids other than H_3_PO_2_ are much less reactive and their reactivity is more distinguished in AcOH than in other solvents. Utilization of acetic acid also solves problems with a solubility of hydrophobic amines which are not soluble in aqueous media. We also showed that prepared AHPA's can be used as intermediates for synthesis of less available or more elaborate phosphorus acid derivatives. Synthetic applicability of several AHPA's was tested. Oxidation of the P–H bond or its further reaction of electrophiles, and/or selective removal of the benzyl/*t*-butyl/phthaloyl amine protecting groups gave compounds which are hardly accessible by other ways. Orthogonally protected compounds can be utilized in a preparation of aminophosphinic acid peptides. In addition, we determine a number of single-crystal structures and this set is the largest collection of the solid-state structures of 1-aminoalkyl-*H*-phosphinic acids published till now. The dominant feature of the structures is presence of intramolecular hydrogen bonds between protonated amine group and the acidic groups.

Detailed mechanistic study of the reaction mixtures showed equilibria of all previously suggested amine-containing intermediates in phospha-Mannich reactions. The high basicity of the amines, excess of formaldehyde and presence of a small amount of water stabilize N(CH_2_OAc) and [N(CH_2_OAc)_2_]^+^ fragments which are relatively resistant to reduction to *N*-methyl group and react with H_3_PO_2_ to the desired products. Presence of strong acids stabilizes (N=CH_2_)^+^ intermediate which is more susceptible to a reduction to methyl group. These reaction conditions are a good alternative to those previously used for phospha-Mannich (Kabachnik–Fields and Moedritzer–Irani–Redmore) reaction and they are useable for preparation of other compounds with N–CH(R)–P fragment.

## Conflicts of interest

There are no conflicts to declare.

## Supplementary Material

RA-010-D0RA03075A-s001

RA-010-D0RA03075A-s002

RA-010-D0RA03075A-s003
