# Supplementary material for: Selective and clean synthesis of aminoalkyl-H-phosphinic acids from hypophosphorous acid by phospha-Mannich reaction
Source: RSC Adv. 2020 Jun 4;10(36):21329–49. doi: 10.1039/d0ra03075a (PMC9059144; doi:10.1039/d0ra03075a)
Supplement: RA-010-D0RA03075A-s001 [file RA-010-D0RA03075A-s001.pdf]

Electronic Supplementary Material (ESI) for RSC Advances.
This journal is © The Royal Society of Chemistry 2020

Electronic Supplementary Information for:

Selective and clean synthesis of amino H-phosphinic acids from hypophosphorus acid by phospha-Mannich reaction

Peter Urbanovský, Jan Kotek, Ivana Císařová and Petr Hermann*

Department of Inorganic Chemistry, Faculty of Science, Universita Karlova (Charles University), Hlavova 8/2030, 12843 Prague 2, Czech Republic. Tel.: +420-22195-1263, fax: +420-22195-1253, e-mail: petrh@natur.cuni.cz

Content:

1. Reaction Scope Investigations

Dependence of reaction on H_2O and HCl (**Tables S1–S2, Figures S1–S3**) ... 2–4

Reaction of *H*-phosphinic acids (**Figures S4–S5**) ... 5

Reaction of 2,2,2-trifluoroethylamines (**Figure S6**) ... 6–7

Reaction of higher aldehydes (**Figures S7–S9**) ... 8–9

Reaction of primary amine (**Figure S10**) and amino-phosphonic acid (**Figure S11**) ... 10

Reaction of linear secondary polyamines (**Figures S12–S13**) ... 11–12

Reaction of cyclic secondary polyamines (**Figures S14–S16**) ... 13–15

2. Mechanistic Studies

N-hydroxymethylation of secondary amine (**Figures S17–S18**) ... 16–19

Investigation of *N*-hydroxymethylated aminomethyl-*H*-phosphinic acid (**Figures S19–S22**) ... 20–25

Investigation of iminium species (**Figures S23–S25**) ... 26–28

Investigation of aminal species (**Figures S26–S27**) ... 29–30

3. Syntheses and Characterization Data

Starting materials (**Tables S3–S4**) ... 31–33

Compounds (**1–17**) in Table 1 ... 34–47

Compounds (**18–21b**) in Table 2 ... 48–52

Compounds (**22–27**) in Table 3 ... 53–59

Compounds (**28a–30**) in Table 4 ... 60–62

Compounds (**31–35**) in Table 5 ... 63–64

Compounds (**31a–35b**) in Table 6 ... 65–69

Compounds (**16a, 36, 37, 37-Me, 38-Me**) based on cyclic amines ... 70–73

Additional compounds (**28c, 25a, 19a, 19b, A–E, D1–D3**) ... 74–83

4. X-ray Diffraction Experimental and Data ... 84

Experimental and fitting details (**Table S5**) ... 85–88

Structural parameters (**Tables S6–S7**) ... 89–91

Supplementary Figures and Tables

1. Reaction Scope Investigations

Table S1, Figure S1

Influence of water content on reaction of Bn_2NH, paraformaldehyde and H_3PO_2 in acetic acid (0.25 mmol of amine and molar ratio 1:2:1.1, respectively; AcOH (2 ml); 40 °C; after 2, 24, and 48 h; conversion determined by ^{31}P NMR).

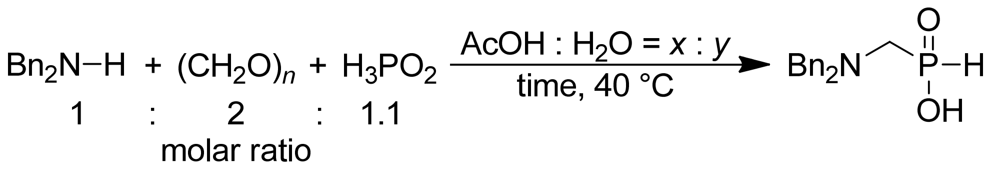

Water content	Conversion, %		
	5 h	24 h	48 h
< 1%	88	90	91
25 %	44	86	91
50 %	22	71	84
75 %	6	46	63
100 %	0	24	42

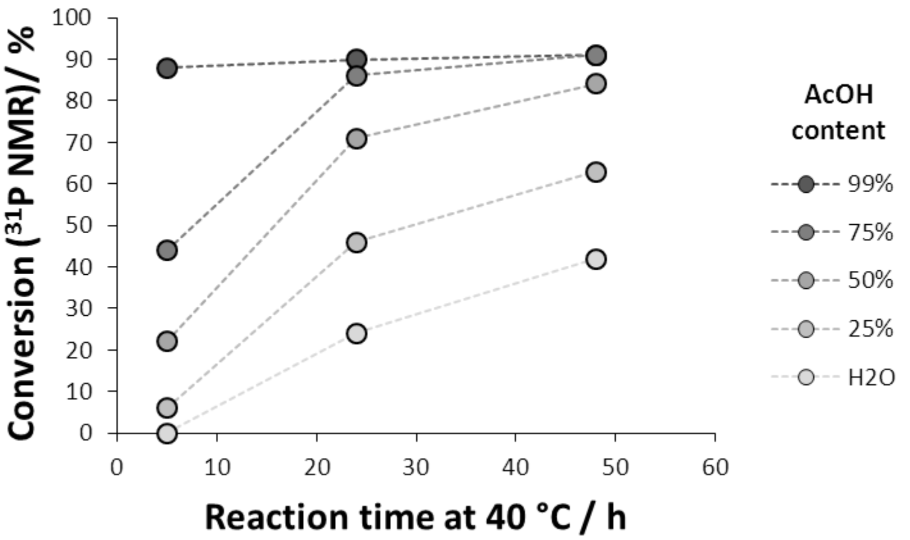

Table S2, Figure S2

Influence of HCl content on reaction of Bn_2NH, paraformaldehyde and H_3PO_2 in acetic acid (0.5 mmol of amine and molar ratio 1:2:1.1, respectively; AcOH (2 ml); 40 °C, 24 h, conversion determined by ^{31}P NMR).

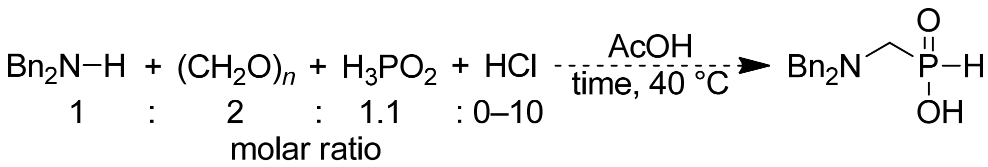

HCl equiv.	Conversion, %
0	90
1	33
~ 10*	< 5

*By addition of conc. HCl, water content in reaction was < 1 %

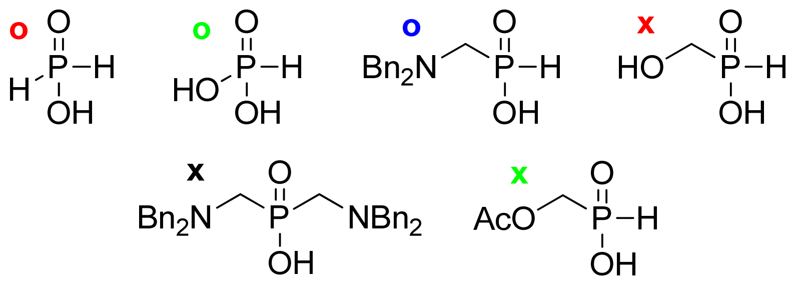

^{31}P NMR spectra:

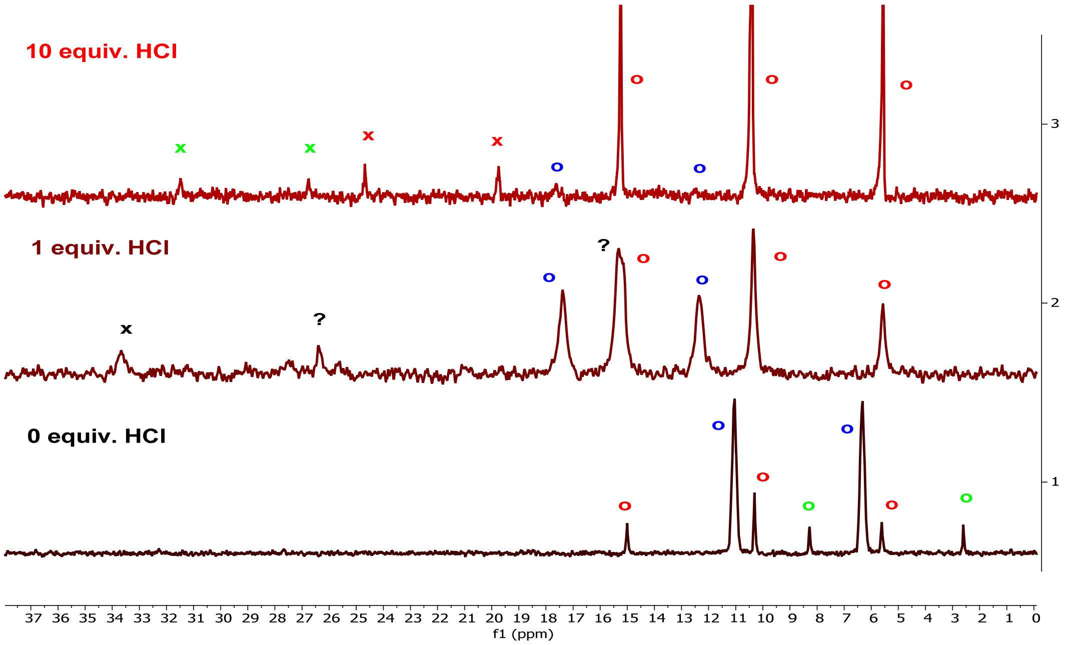

Figure S3

^{1}H (top) and ^{31}P (bottom) NMR spectra of the aq. ammonia fraction after chromatography on Dowex 50 column. Elution was done with 10% aq. pyridine (removal of compounds with no amine groups and (*N*,*N*-dibenzyl-amino)-methyl-*H*-phosphinic acid**1**) followed by conc. aq. NH_3:EtOH = 1:5 (elution of all other amines). The spectra (not referenced) show mixture of *N*-methylated *N*,*N*-dibenzyl-amine and bis(*N*,*N*-dibenzyl-aminomethyl)phosphinic acid in ratio ~8:1. Characterisation data are identical to published.[1] The sample originated from reaction of (Bn_2NH:CH_2O:aq. H_3PO_2 = 1:2:1.1 with 1 equiv. HCl present as hydrochloride salt of starting amine, AcOH as solvent, 24 hours, 40 °C).

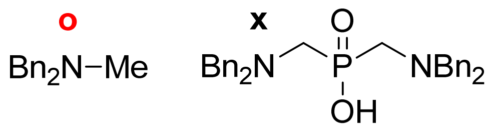

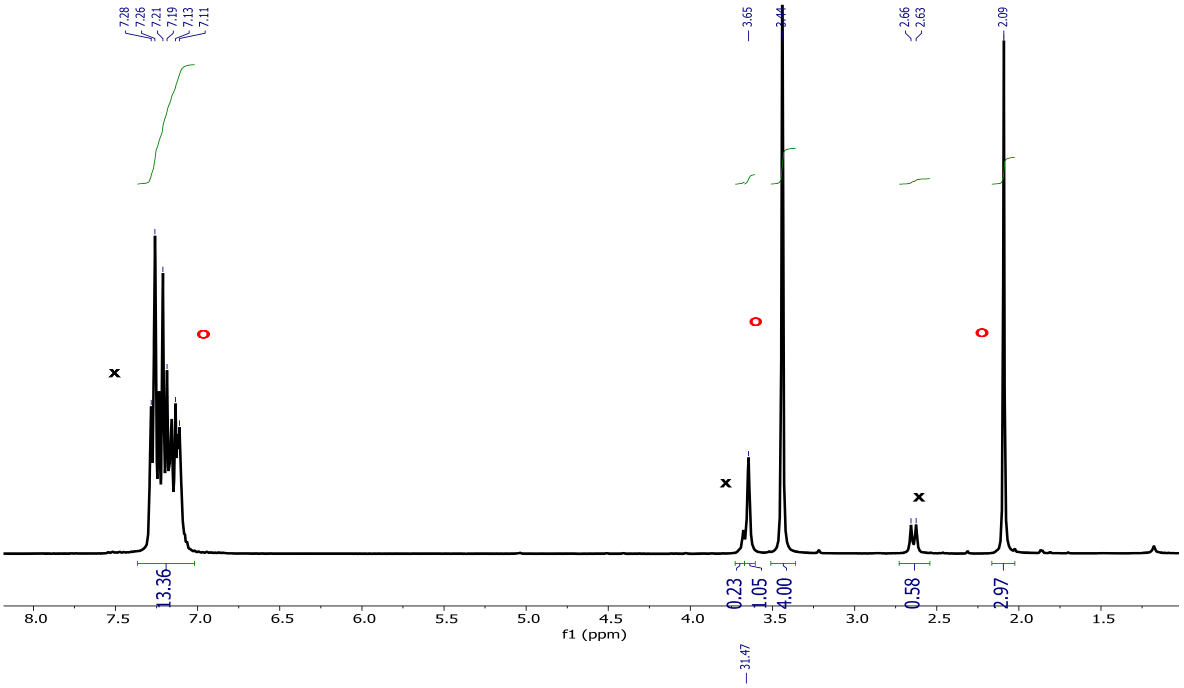

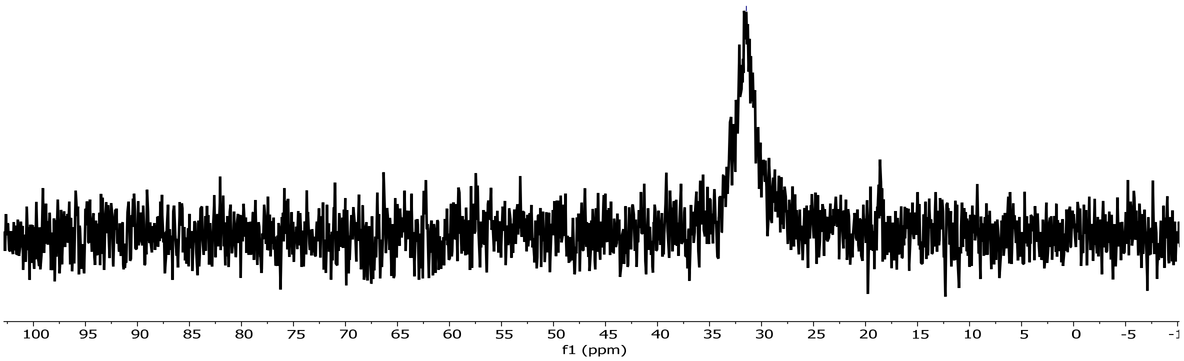

[1] G. Tircso, A. Benyei, R. Kiraly, I. Lazar, R. Pal, and E. Brucher, *Eur. J. Inorg. Chem.* **2007**, 701–713.

Figure S4

^{31}P NMR spectrum of reaction mixture of *N*,*N*-dibenzyl-amine, paraformaldehyde and H_3PO_3 (0.25 mmol of amine, molar ratio 1:2:1.1, respectively; AcOH (2 ml); 36 h at 40 °C).

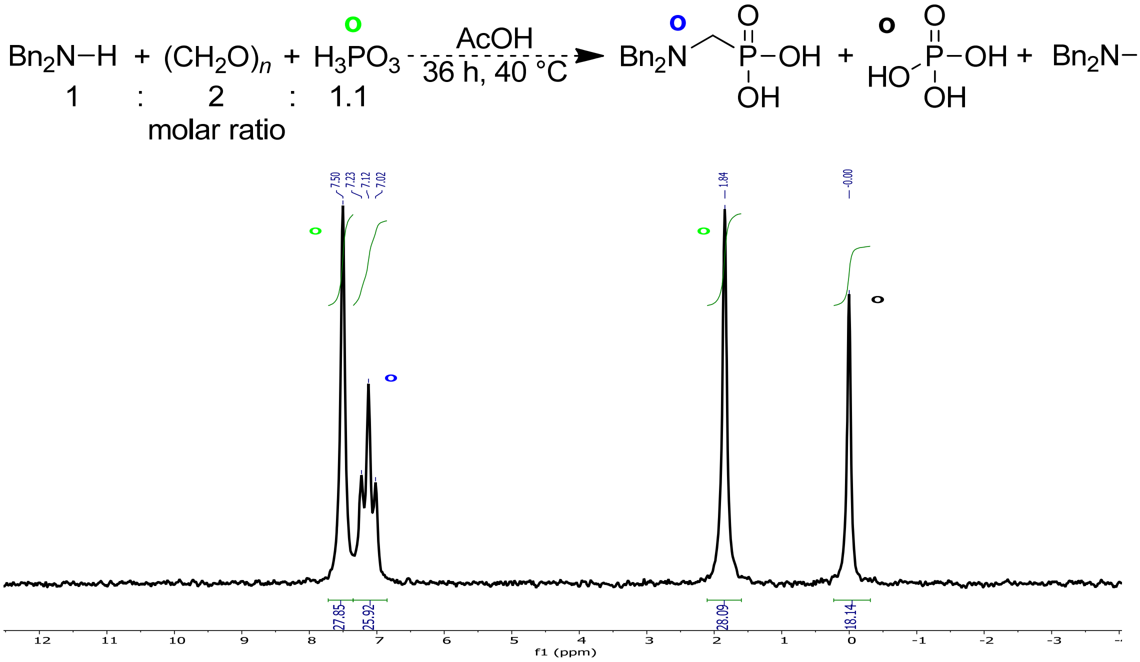

$Bn_2NCH_2PO_3H_2$ was identified at δ_P ~7 ppm (triplet), H_3PO_3 corresponds to dublet at ~2 + 7.5 ppm and H_3PO_4 singlet at ~0 ppm (used as an internal reference).

Figure S5

^{31}P NMR spectrum (not referenced) of reaction mixture of *N*,*N*-dibenzyl-amine, paraformaldehyde and (*N*,*N*-dibenzyl-amino)-methyl-*H*-phosphinic acid (0.5 mmol of phosphinic acid, molar ratio 1.1:2:1, respectively; AcOH (2 ml); 36 h at 40 °C). Reaction was not in equilibrium but molar ratio of products (C–P–C and R–PO_3H_2) remained the same in approx. 1:1.1 molar ratio, respectively.

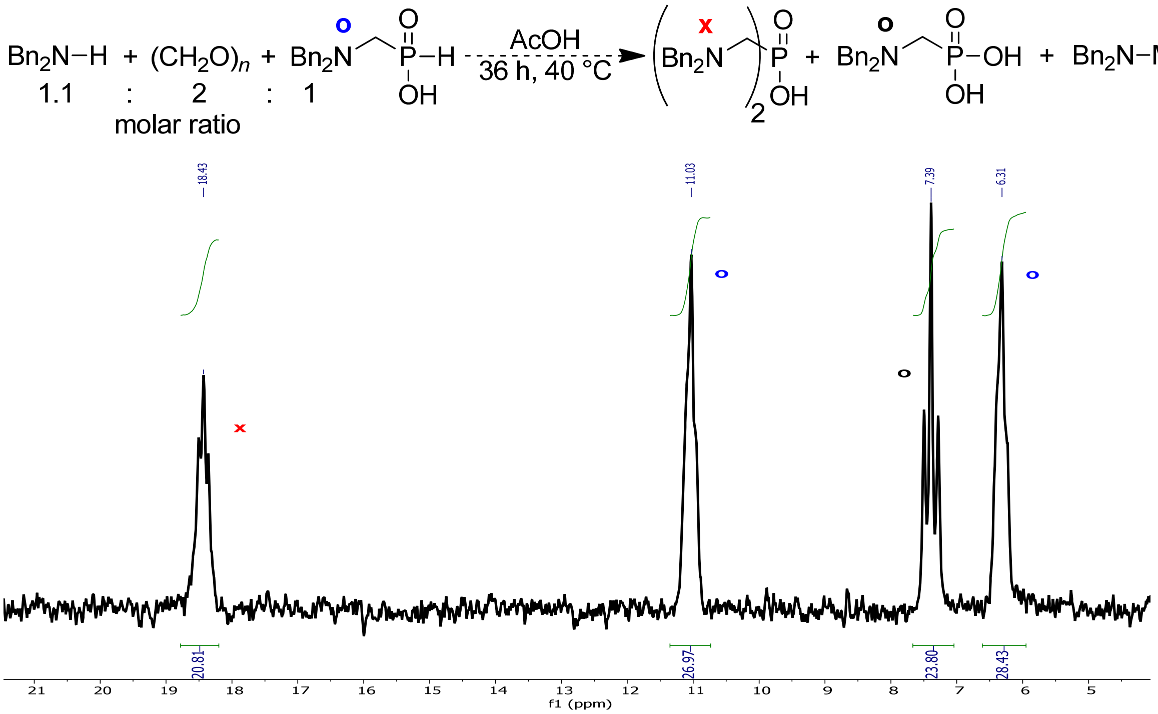

Figure S6

^{1}H, ^{31}P and ^{19}F NMR spectra of reaction mixture with (*N*-benzyl)-2,2,2-trifluoroethylamine (*a*) and bis(2,2,2-trifluoroethyl)amine (*b*), paraformaldehyde and 50% aq. H_3PO_2 in molar ratio 1:2:1.1, respectively (0.5 mmol of amine, AcOH (2 ml), 1 d, 40 °C). Spectra were not referenced.

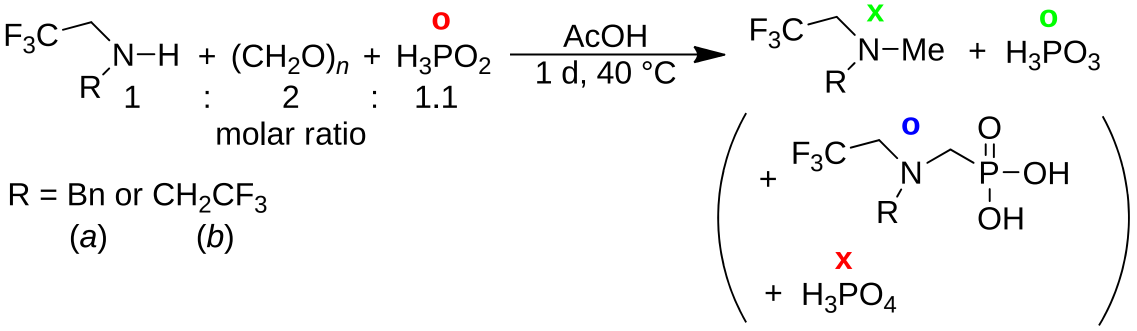

The integrated signals in ^{1}H NMR spectra correspond to *N*-methylated amines. ^{31}P NMR spectra show majority (> 60 %) of H_3PO_3 present and small amount of amino phosphonic acid (~5 %). The ^{19}F NMR spectra show *N*-methylated amines ($^{3}J_{FH}$ ~ 9.0 Hz (*a*) and ~ 9.5 Hz (*b*), which are similar to published values[2]) and amino-phosphonic acid ($^{3}J_{FH}$ ~ 9.2 Hz (*a*) and ~ 8.2 Hz (*b*)).

^{1}H (*a*)

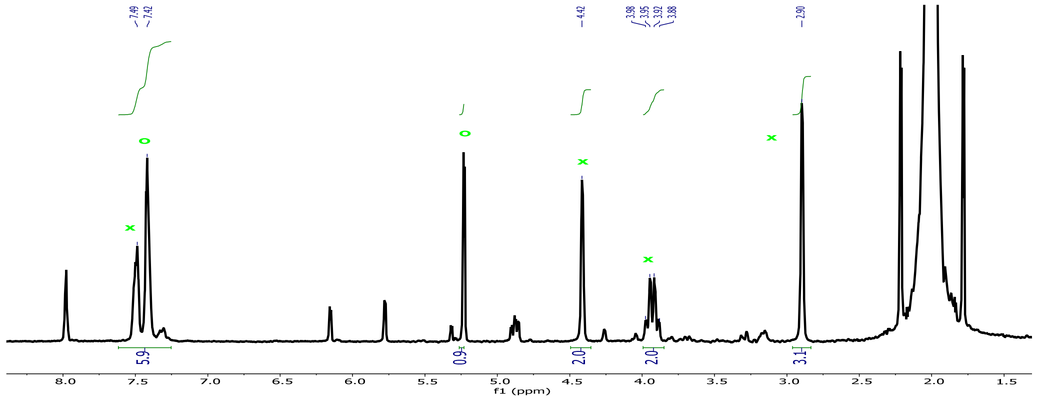

^{1}H (*b*)

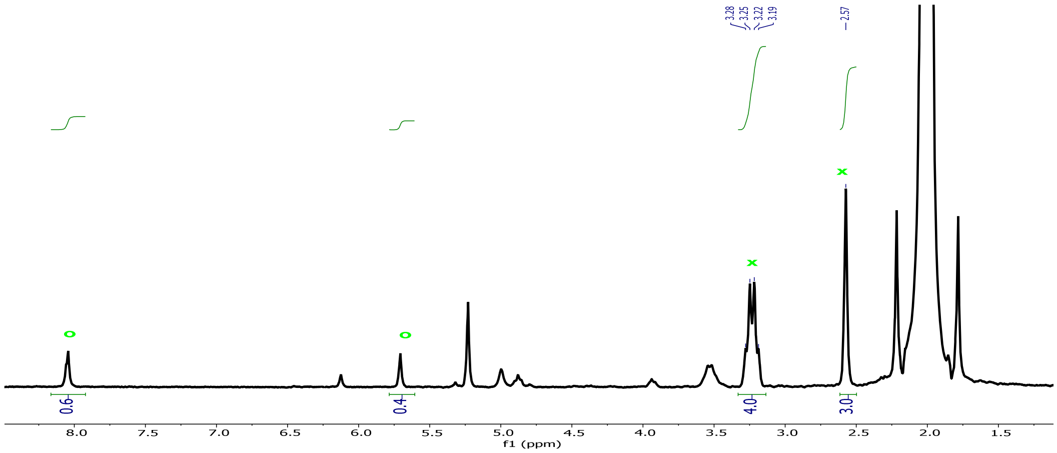

[2] (a) H. Mimura *et al.*, *J. Fluorine Chem.* **2010**, *131*, 477–486. (b) H. Burger *et al.*, *J. Fluorine Chem.* **1989**, *44*, 147–153.

^{31}P (*a*)

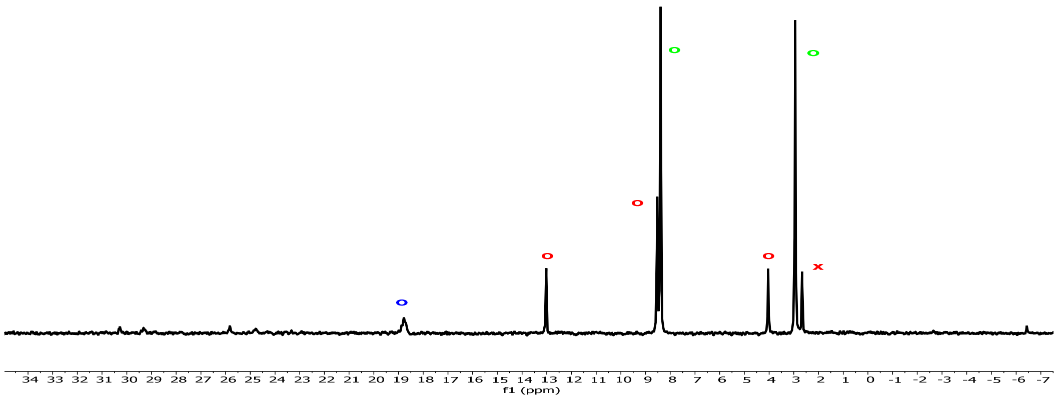

^{31}P (*b*)

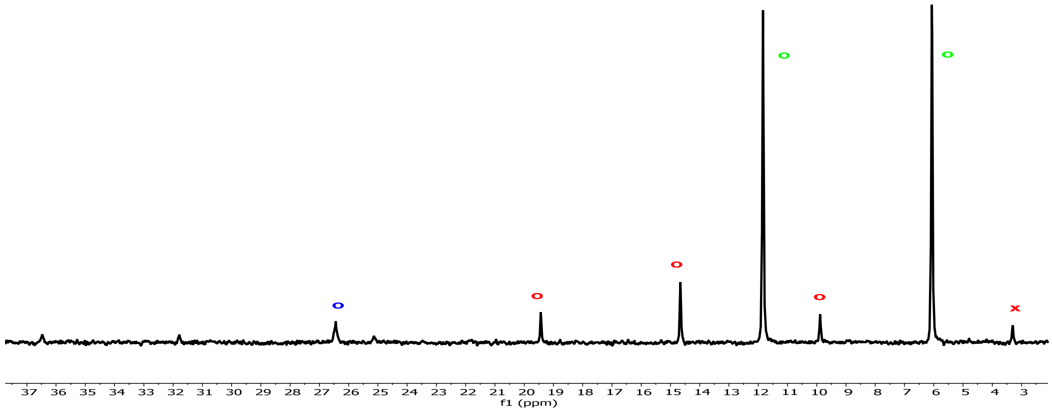

^{19}F (*a*)

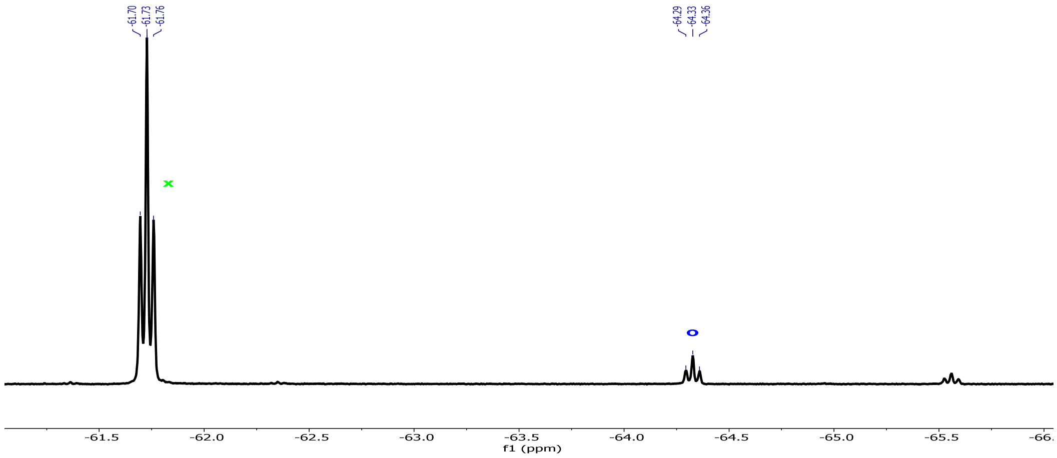

^{19}F (*b*)

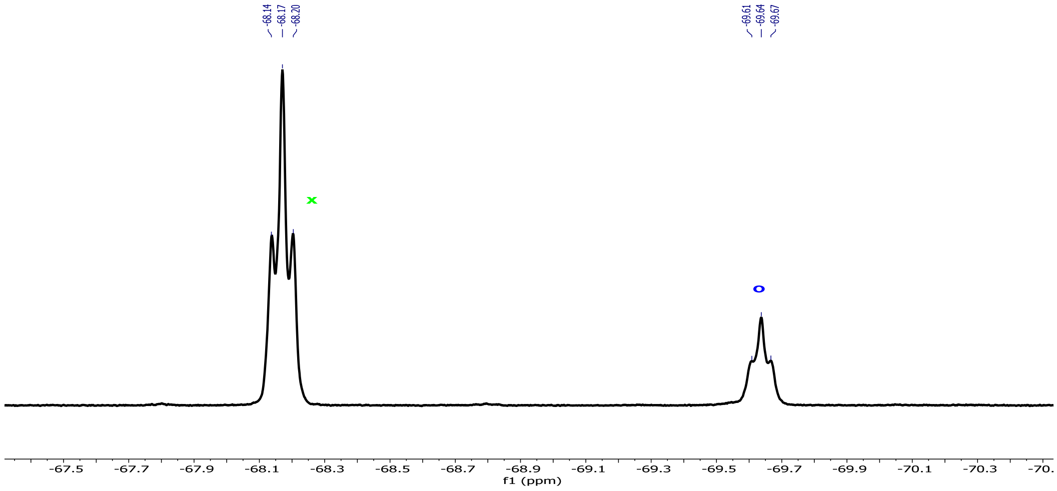

Figure S7

^{31}P NMR spectrum of *N*,*N*-dibenzyl-amine, acetaldehyde and 50% aq. H_3PO_2 (0.5 mmol of amine, in molar ratio 1:2:1.1, respectively; AcOH (2 ml), 2 d, 60 °C). Spectrum was not referenced.

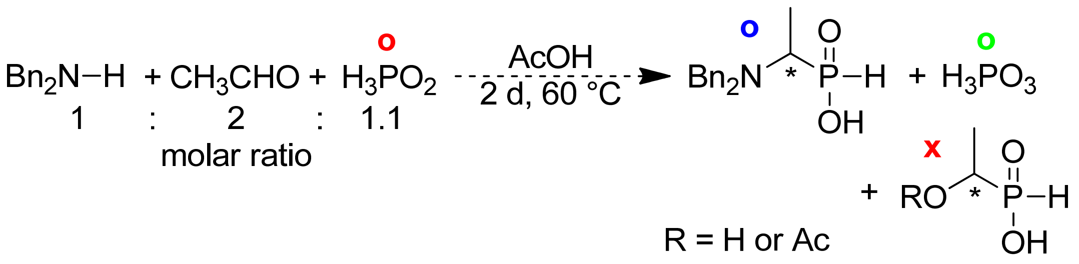

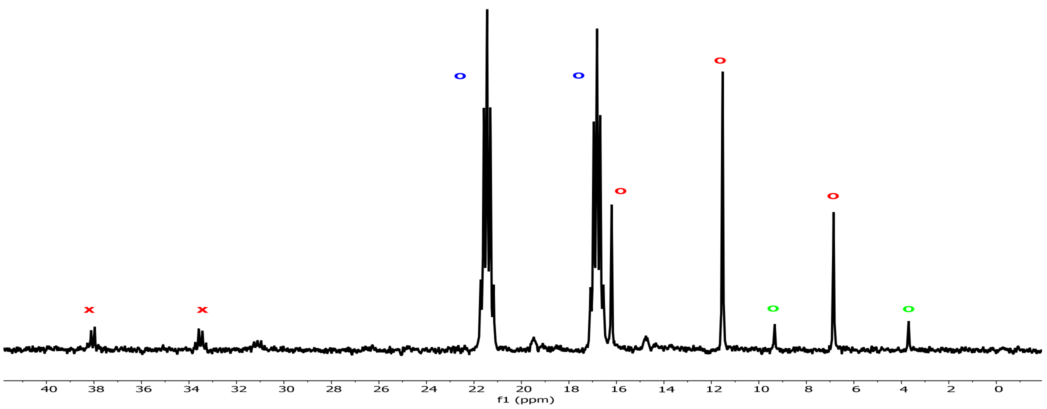

Figure S8

^{31}P NMR spectra of *N*,*N*-dibenzyl-amine, benzaldehyde and 50% aq. H_3PO_2 (0.5 mmol of amine, in molar ratio 1:2:1.1, respectively; AcOH (2 ml), 2 d at 60 °C (bottom) and 3 d at 80 °C (top)). Spectra were not referenced.

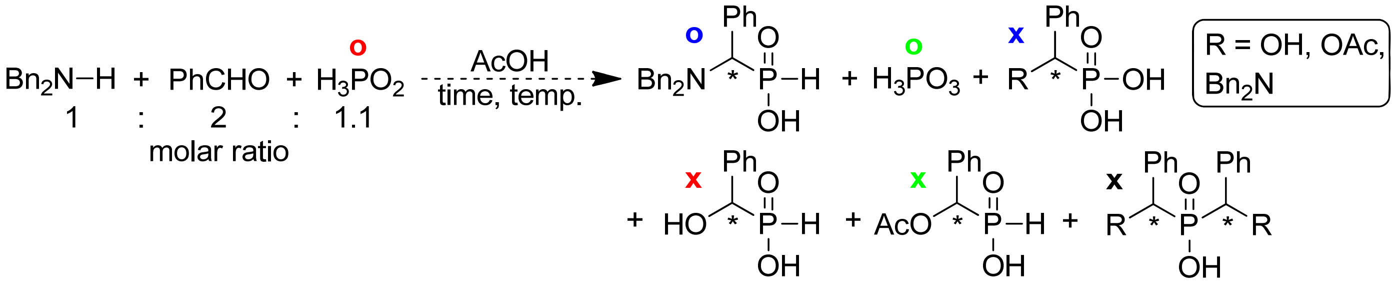

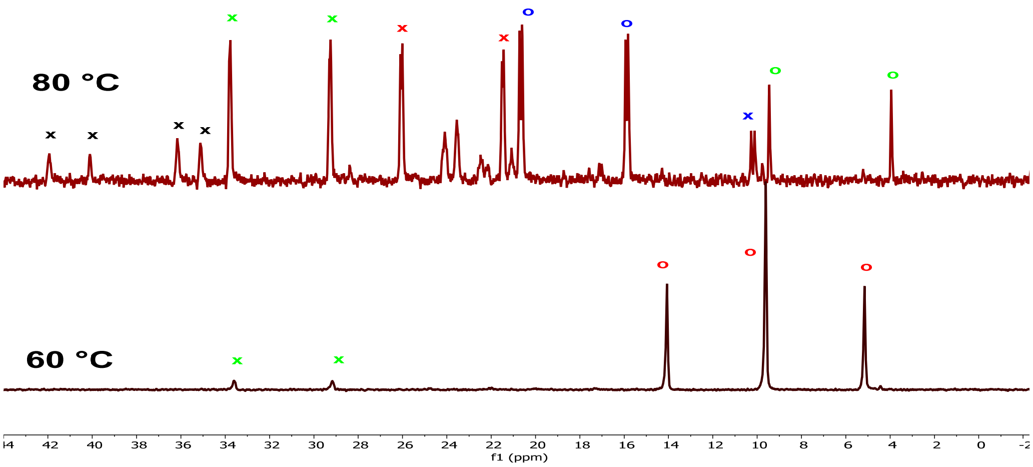

Figure S9

^{31}P NMR spectra of *N*,*N*-dibenzyl-amine, trifluoroacetaldehyde monohydrate and 50% aq. H_3PO_2 (0.5 mmol of amine, in molar ratio 1:2:1.1(bottom), respectively, or 0:2:1.1 (top), respectively; AcOH (2 ml), 2 days at 80 °C). Spectra were not referenced.

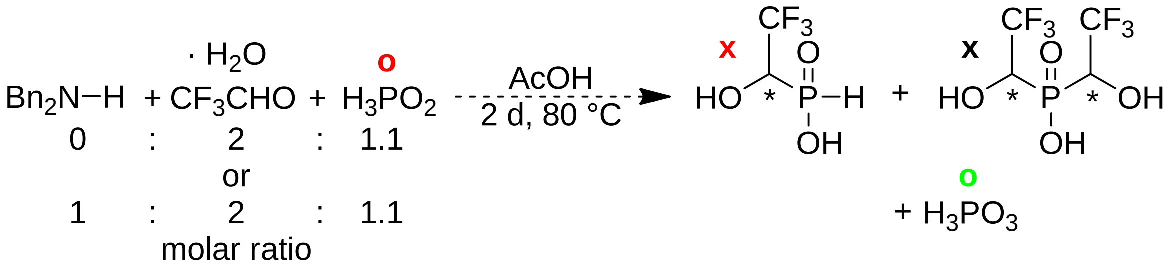

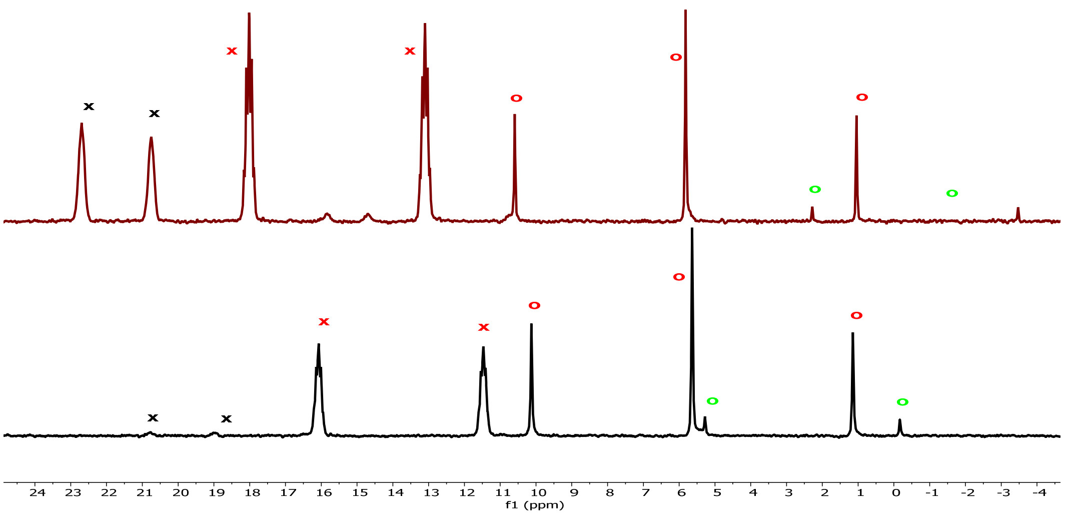

Figure S10

^{31}P NMR spectrum of *N*-cyclohexylamine, paraformaldehyde and 50% aq. H_3PO_2 (0.5 mmol of amine, in molar ratio 1:2.2:2.2, respectively; AcOH (2 ml), 1 d, RT). Spectrum was not referenced.

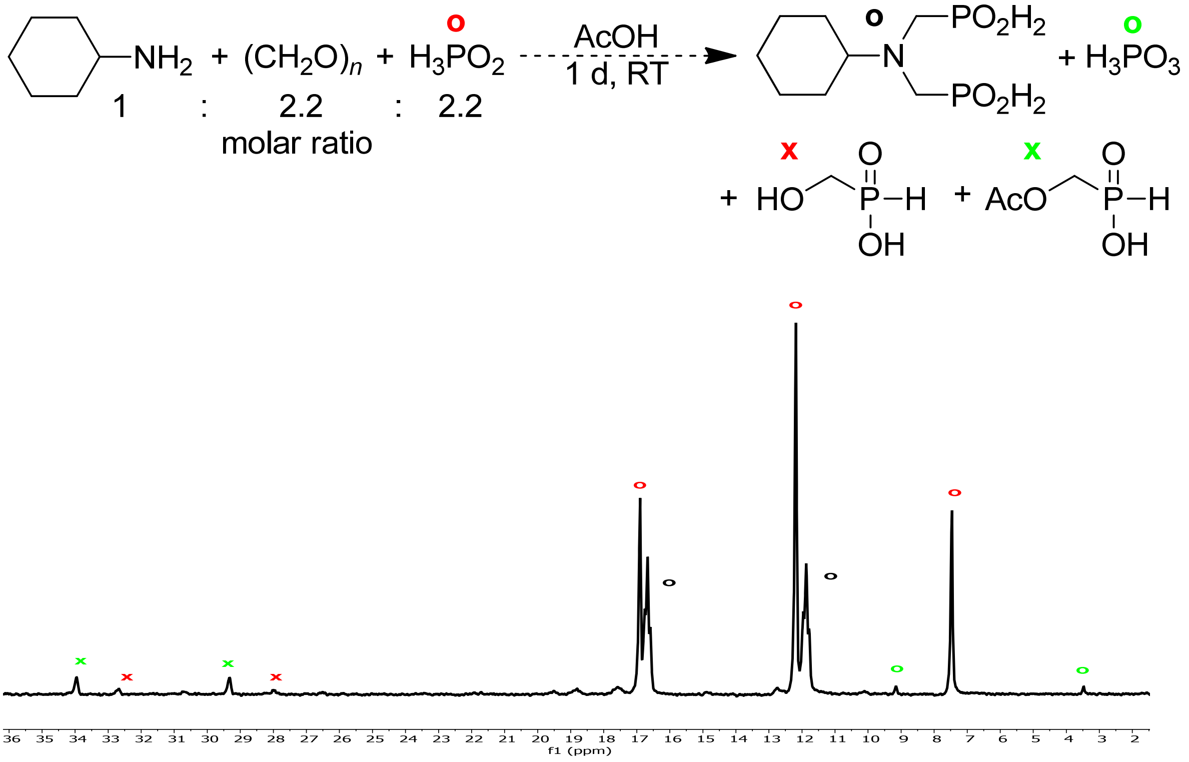

Figure S11

^{31}P NMR spectrum of (*N*-benzyl)-aminomethylphosphonic acid, paraformaldehyde and 50% aq. H_3PO_2 (0.5 mmol of amine, in molar ratio 1:2:1.1, respectively; AcOH (2 ml), 1 d, 40 °C). Spectrum was not referenced. Molar ratio of mono- and bis-substituted phosphinic acids is ~3:1 (*i.e.* blue-to-green in the Figure label).

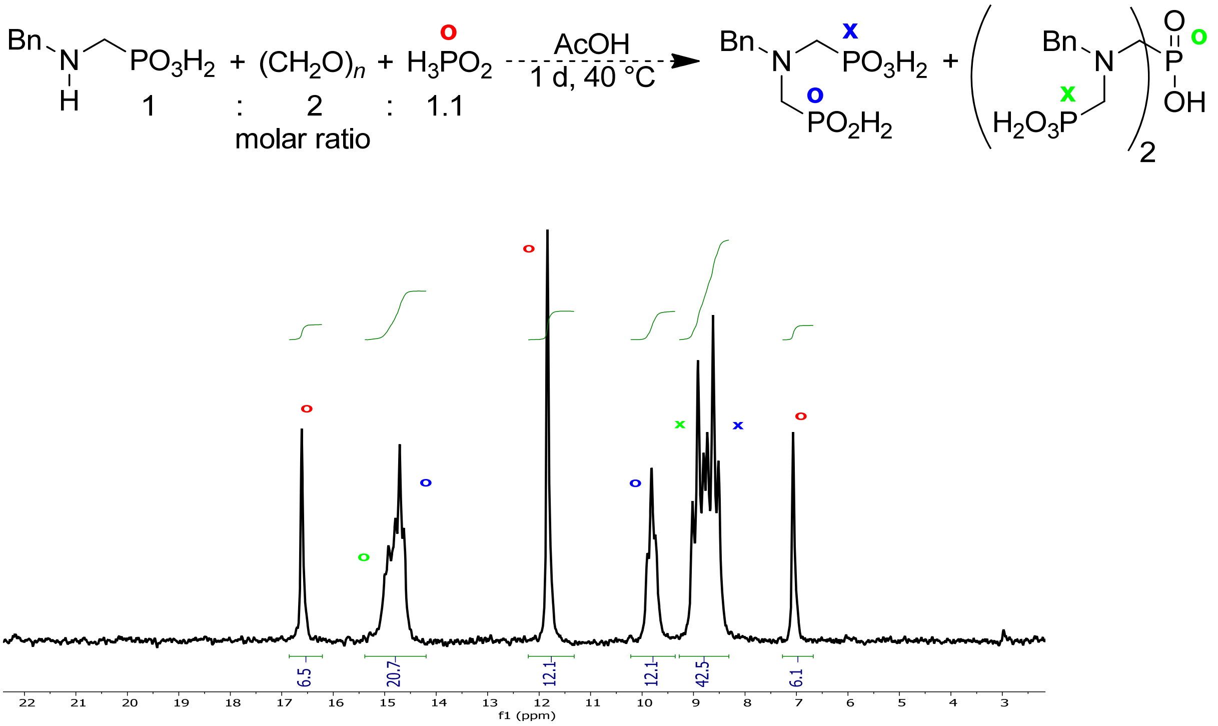

Figure S12

^{31}P NMR spectrum of (*N*,*N'*-dibenzyl)-ethylenediamine, paraformaldehyde and 50% aq. H_3PO_2 (0.5 mmol of amine, in molar ratio 1:4:2.2, respectively; AcOH (10 ml), 1 d, 40 °C). Spectrum is not referenced. Molar ratio of *N*-methylated mono-phosphinic acid to H_3PO_3 is ~1:1.

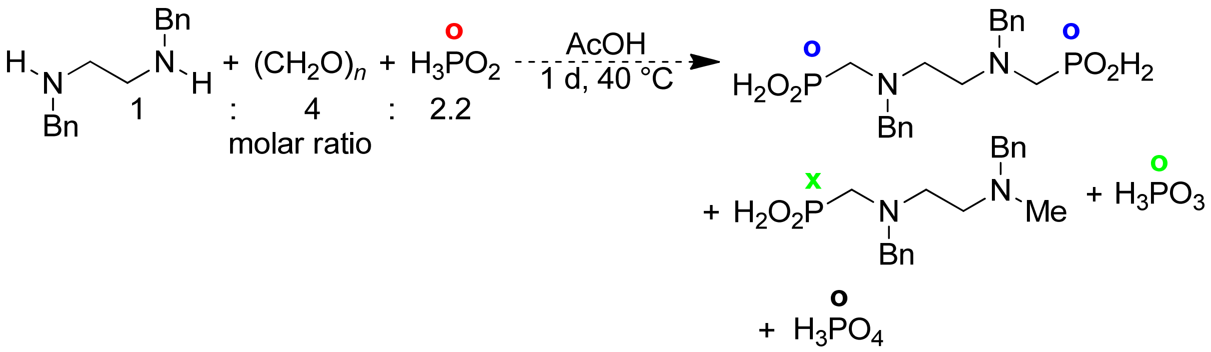

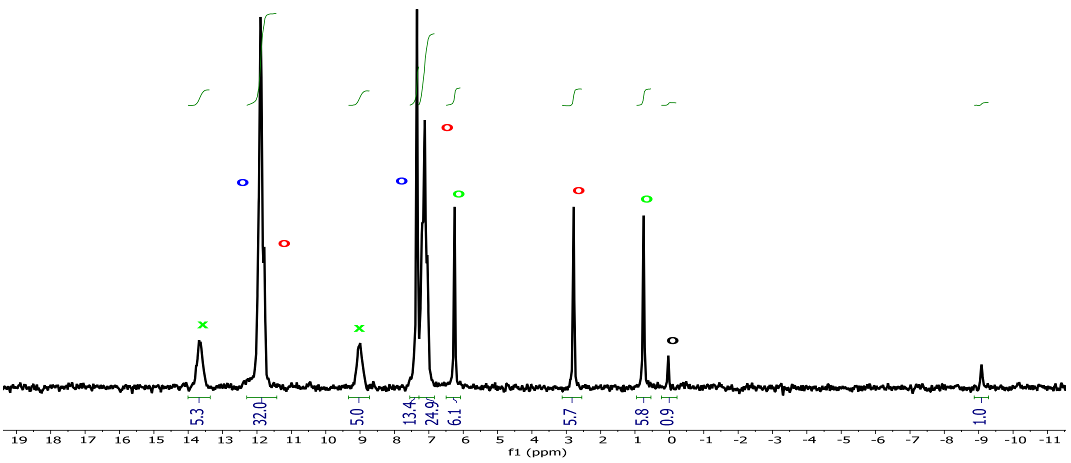

Figure S13

^{31}P NMR spectra of (*N*,*N″*-dibenzyl)-diethylenetriamine (*a*) or (*N*,*N″*-dibenzyl)-dipropylenetriamine (*b*) or (*N*,*N″*-dibenzyl)-dihexylenetriamine (*c*) with paraformaldehyde, and 50% aq. H_3PO_2 (0.25 mmol of amine, in molar ratio 1:6:3.3, respectively; AcOH (2 ml), 1 d, 40 °C). Spectra were not referenced.

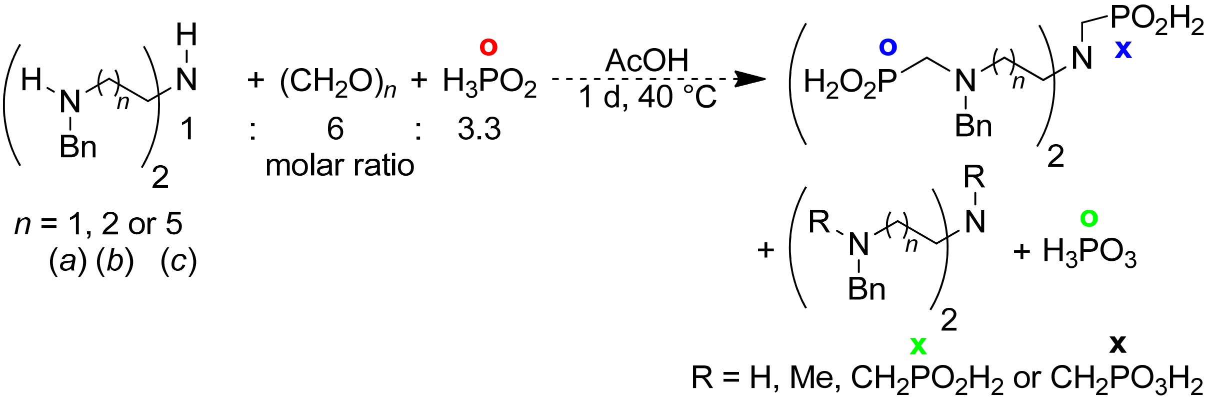

(*a*)

(*b*)

(*c*)

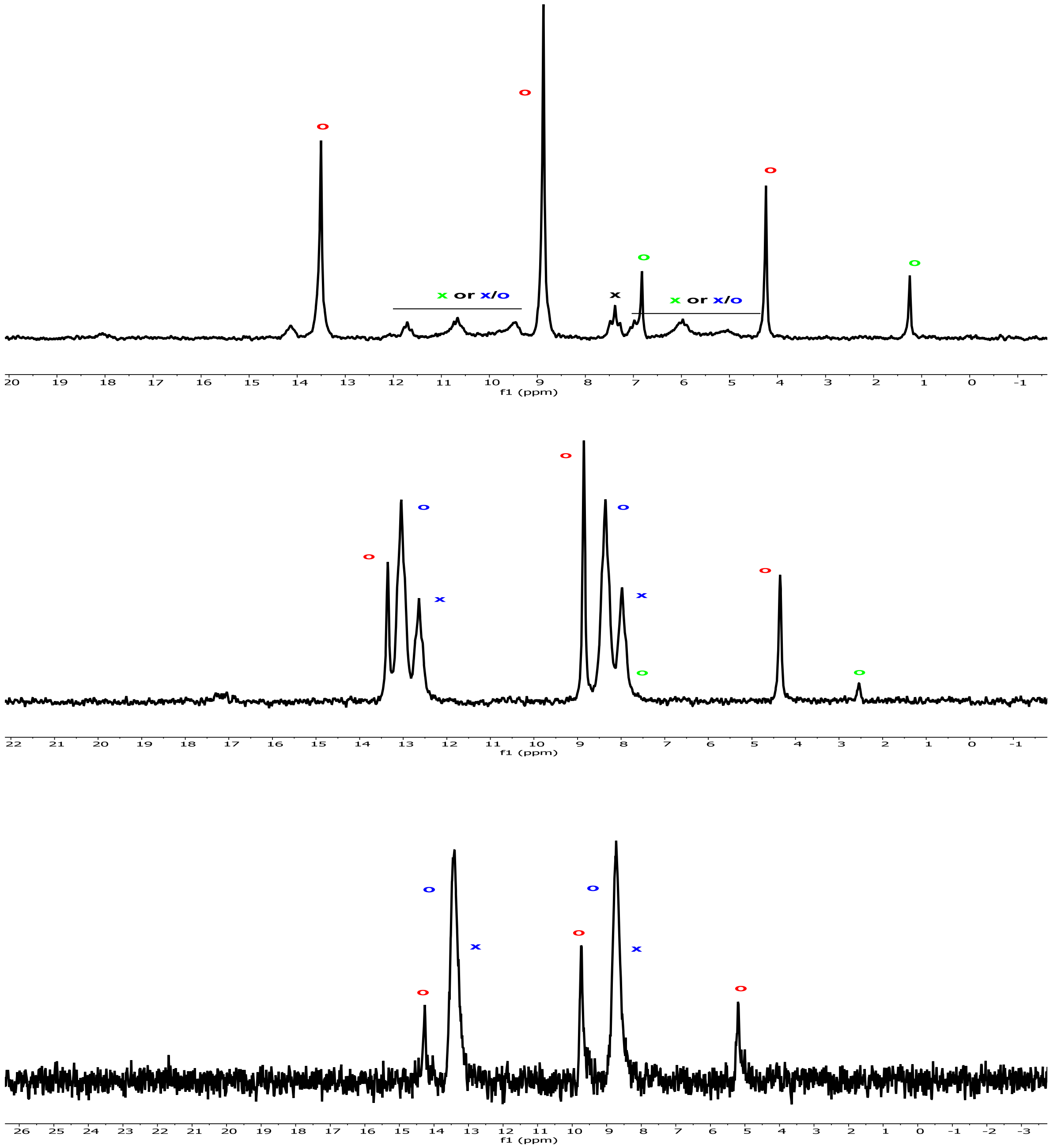

Figure S14

^{31}P NMR spectrum of piperazine, paraformaldehyde and 50% aq. H_3PO_2 (0.5 mmol of amine, in molar ratio 1:4:2.2, respectively; AcOH (2 ml), 1 d, 40 °C). Spectrum was not referenced.

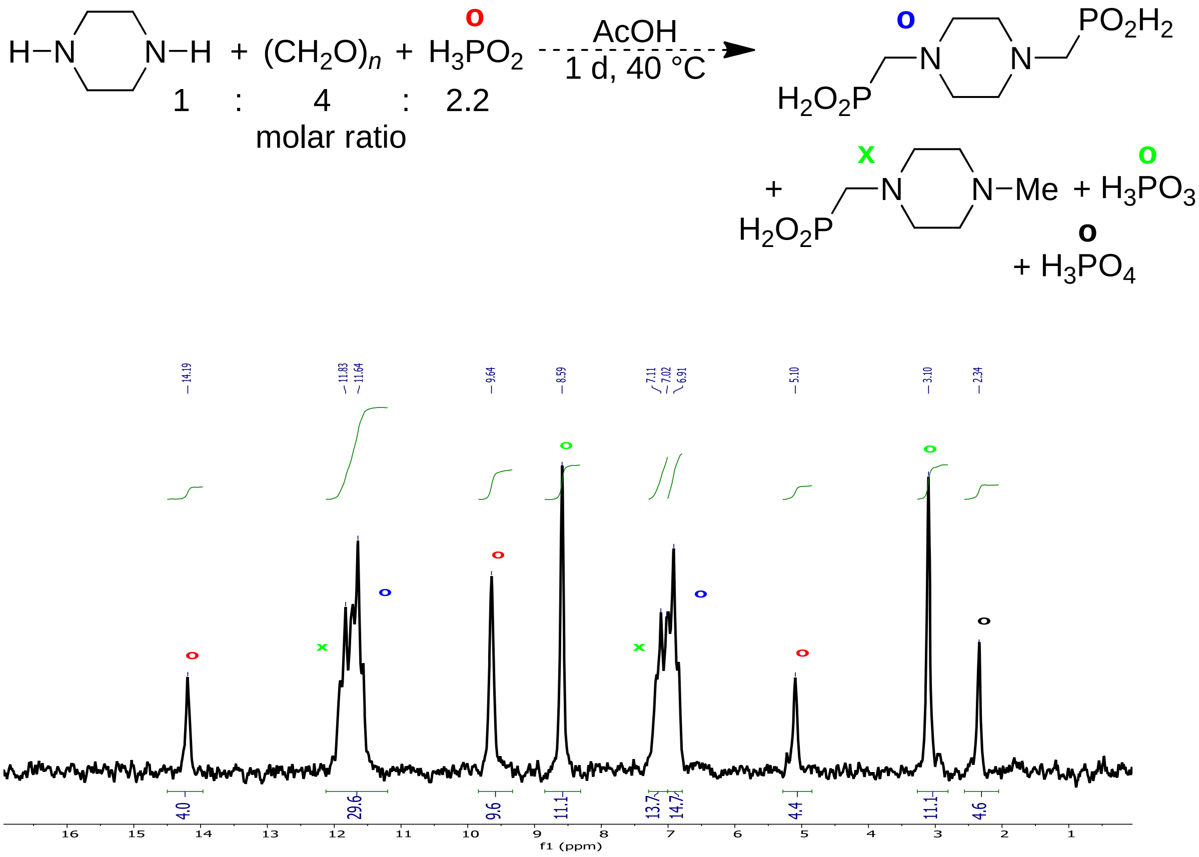

Figure S15

^{31}P NMR spectra of tacn, paraformaldehyde and 50% aq. H_3PO_2 (0.25 mmol of amine, in molar ratio 1:2.2:1 (top) and 1:2.2:3 (bottom), respectively; AcOH (2 ml), 1 d, 40 °C). Spectra were referenced to $\delta_P(H_3PO_2)$ = 6.0 ppm.

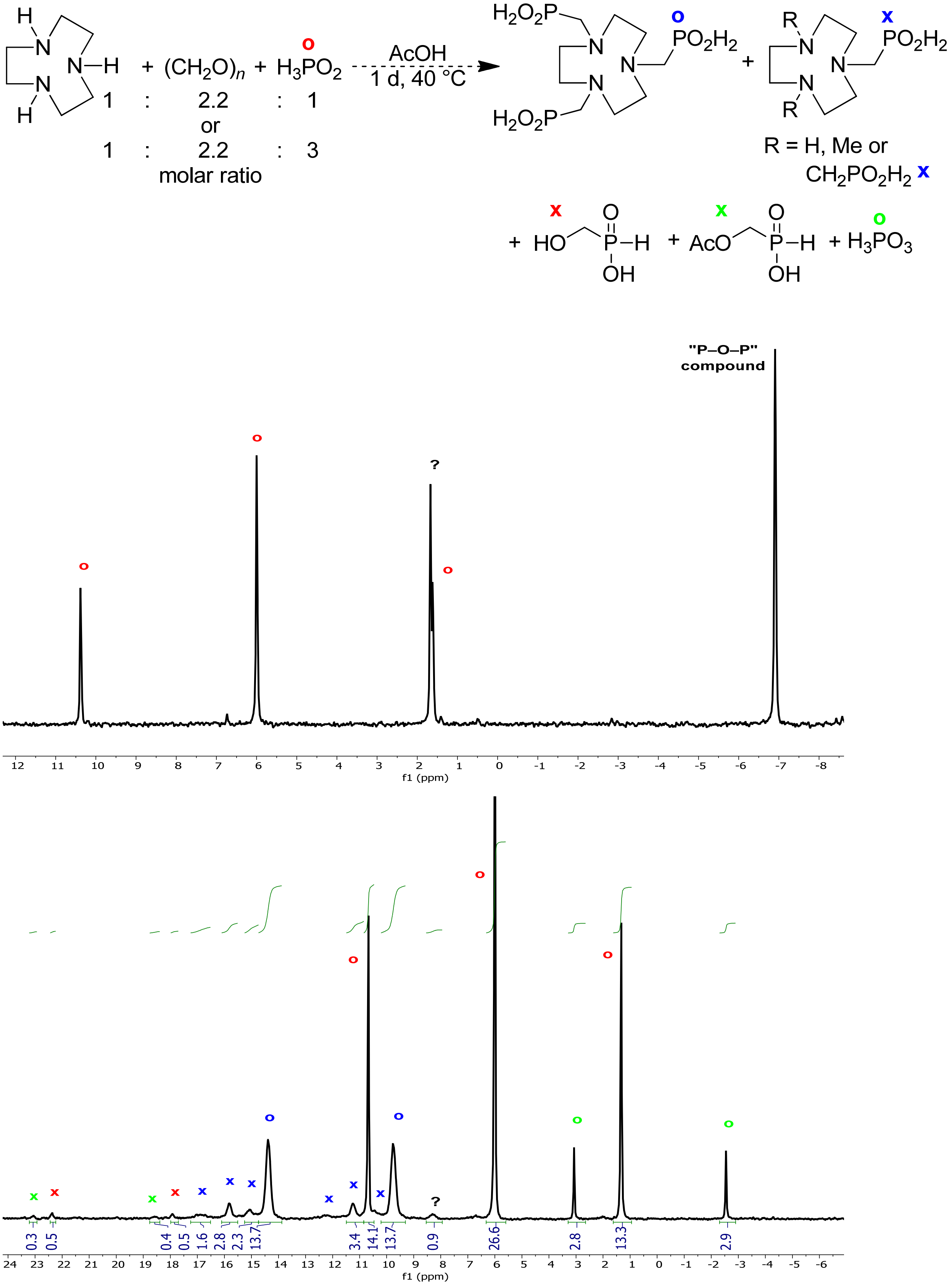

Figure S16

^{31}P NMR spectra of cyclen, paraformaldehyde and 50% aq. H_3PO_2 (0.25 mmol of amine, in molar ratio from 1:1:4 (bottom) to 1:4:4 (top), respectively; AcOH (2 ml), after 4 h each, 40 °C). Spectra were referenced to $\delta_P(H_3PO_2)$ ~9 ppm. Some amino-*H*-phosphinic acid signals (blue circle) are visible only in the spectrum (***4***) at ~16 ppm as a broad dublet.

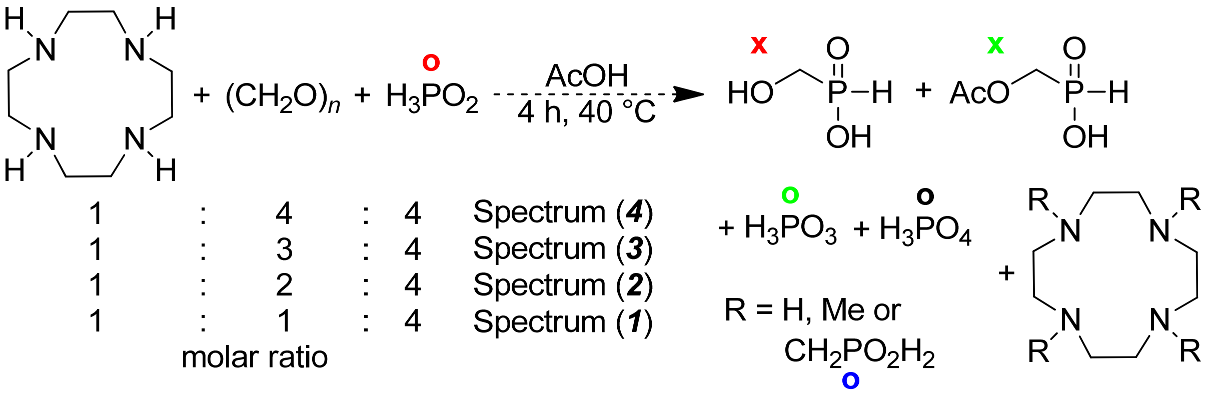

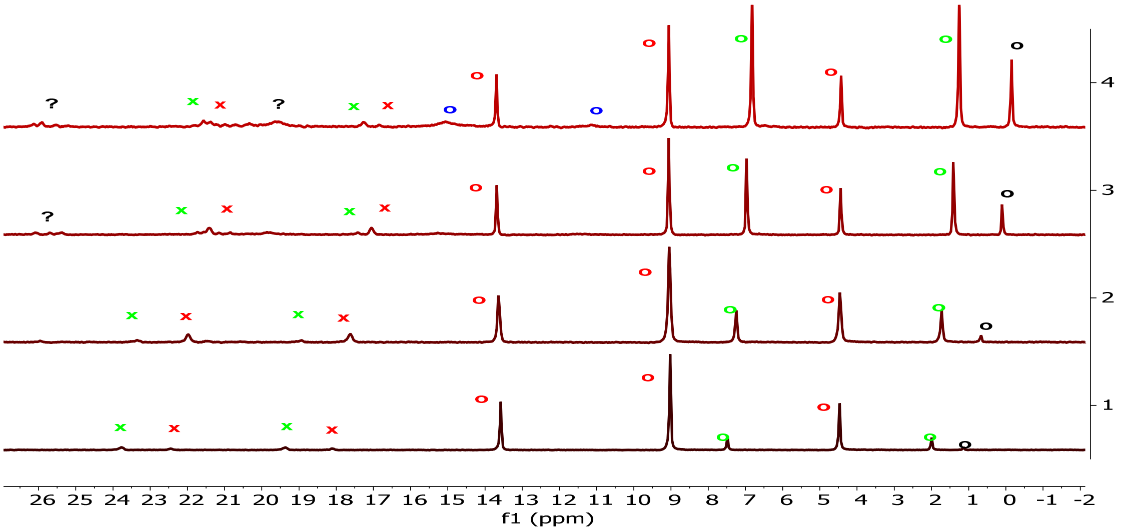

2. Mechanistic Studies

Figure S17

^{1}H and ^{13}C{^{1}H} NMR spectra of paraformaldehyde (*a*) and its mixtures with Me_2NH (as 40% aq. solution) after gradual addition of paraformaldehyde (in molar ratio 1:1 (*b*), 2:1 (*c*) and 3:1 (*d*), respectively; next addition of paraformaldehyde always after 1 d, 40 °C). Solutions were heated up to 40 °C in AcOH and measured with a D_2O + *t*BuOH in insert tube. Spectra show formation of two intermediates, $(CH_3)_2NCH_2OR$ and $[(CH_3)_2N(CH_2OR)_2]^+$ (where R = H or Ac).

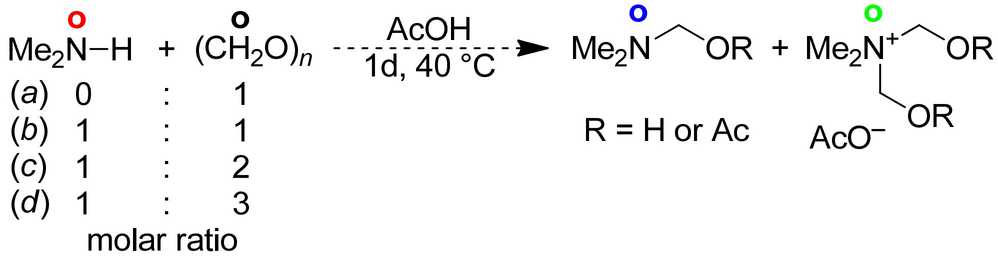

^{1}H

(*a*)

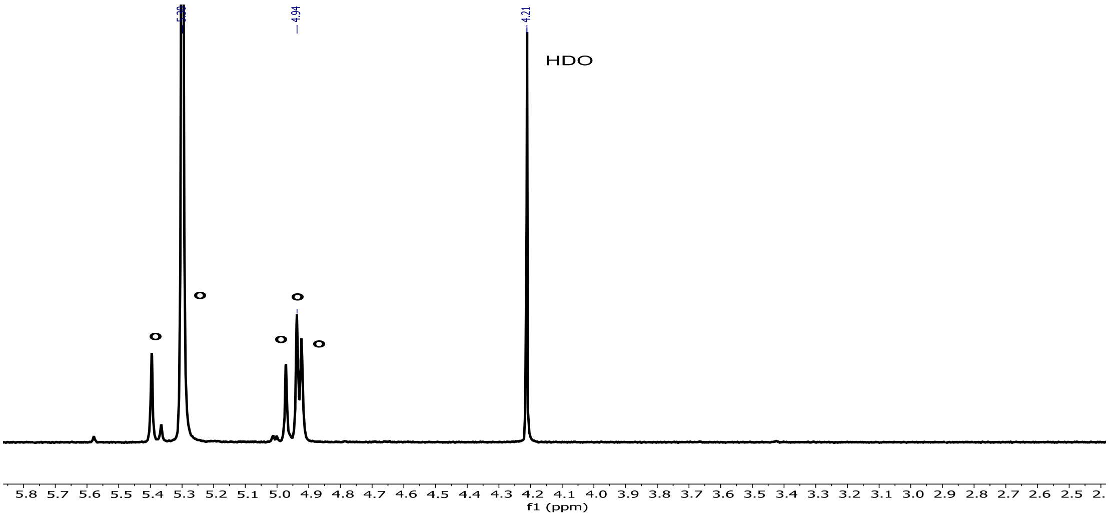

^{1}H

(*b*)

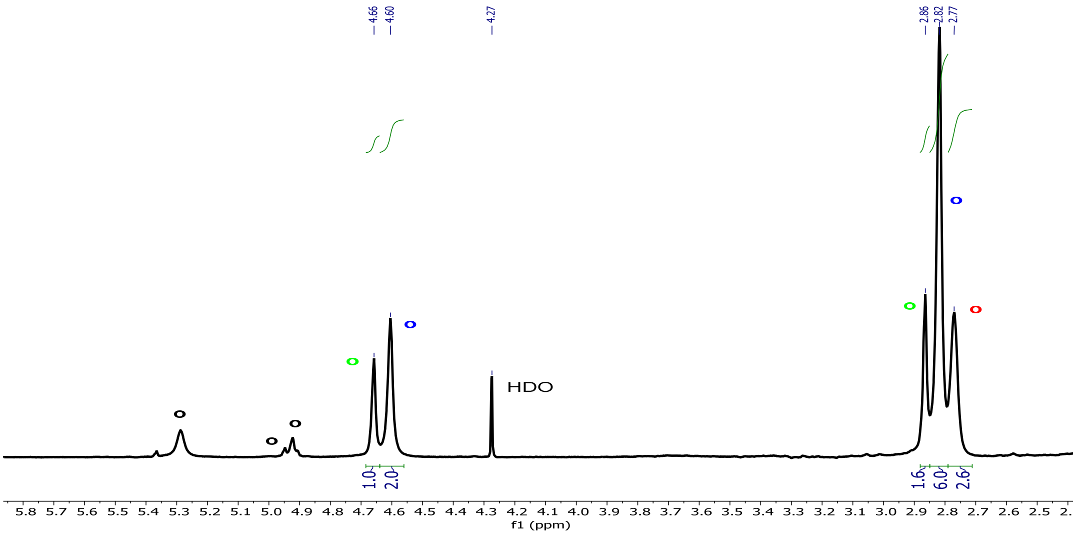

^{1}H

(*c*)

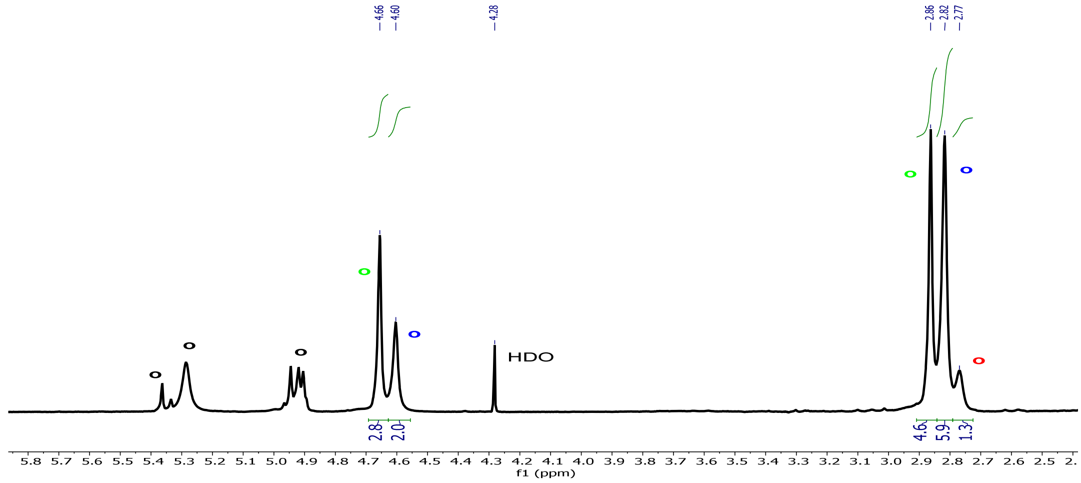

^{1}H

(*d*)

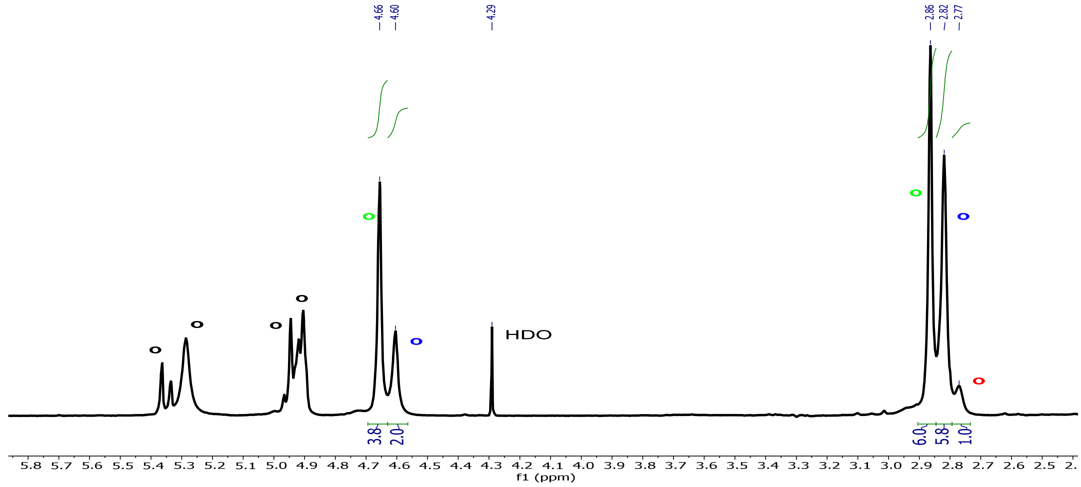

$^{13}C\{^{1}H\}$

(*a*)

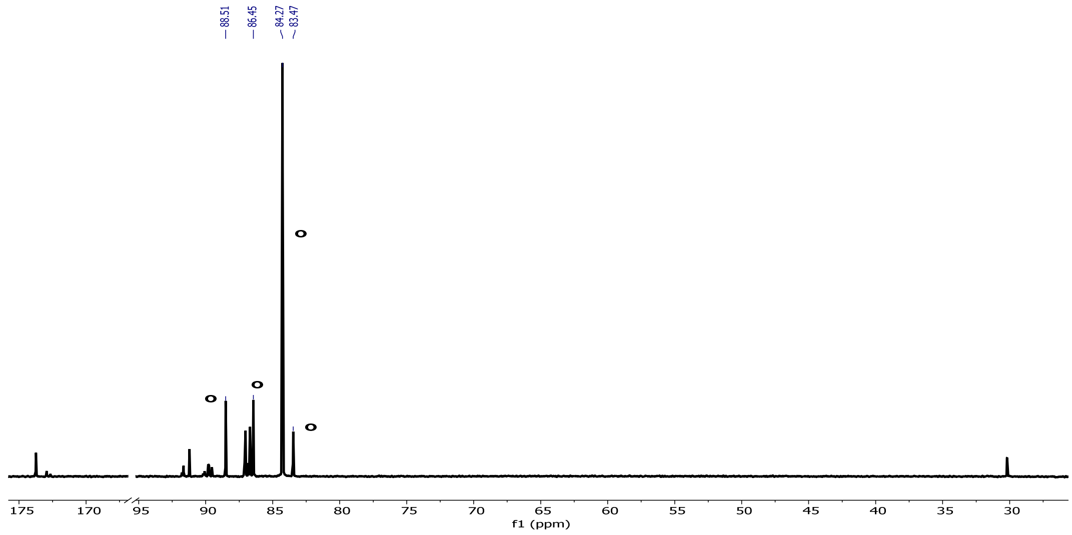

$^{13}C\{^{1}H\}$

(*b*)

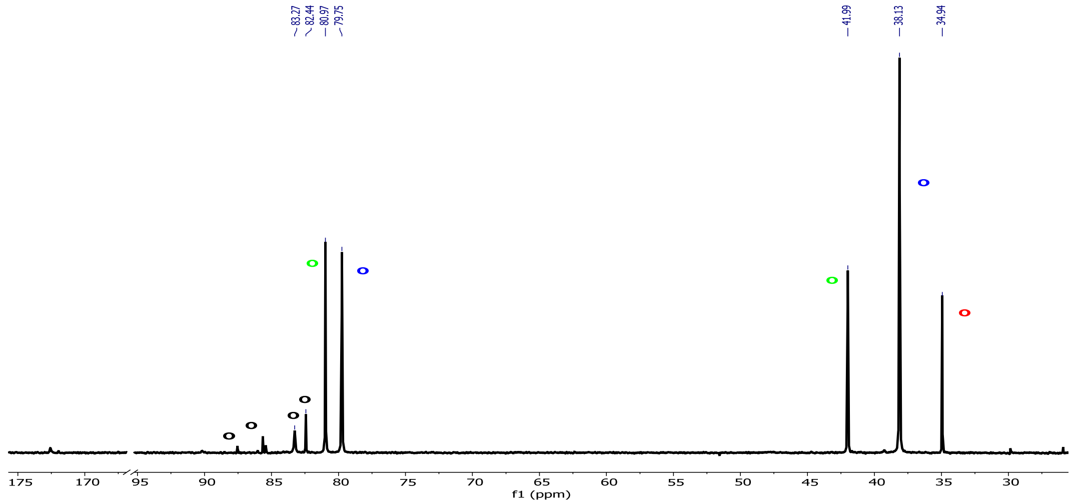
83.27
82.44
80.97
79.75
41.99
38.13
34.94
175 170 95 90 85 80 75 70 65 60 55 50 45 40 35 30
f1 (ppm)

Figure S18

Stacked ^{1}H (*a*) and ^{31}P (*b*) NMR spectra of reaction of Me_2NH (as 40% aq. solution), paraformaldehyde and H_3PO_2 in the final molar ratio 1:1.5:2 in D_2O (referenced to $\delta_H(HDO) = 4.70$ ppm and $\delta_P(H_3PO_2) = 10.0$ ppm). Due to different pH's, ^{1}H NMR signals of the same compounds appear with different chemical shifts in spectrum (***1***) than in spectrum (***2***) etc. The ^{31}P NMR spectra show difficult splitting due to ^{1}H-to-[2]D exchange.

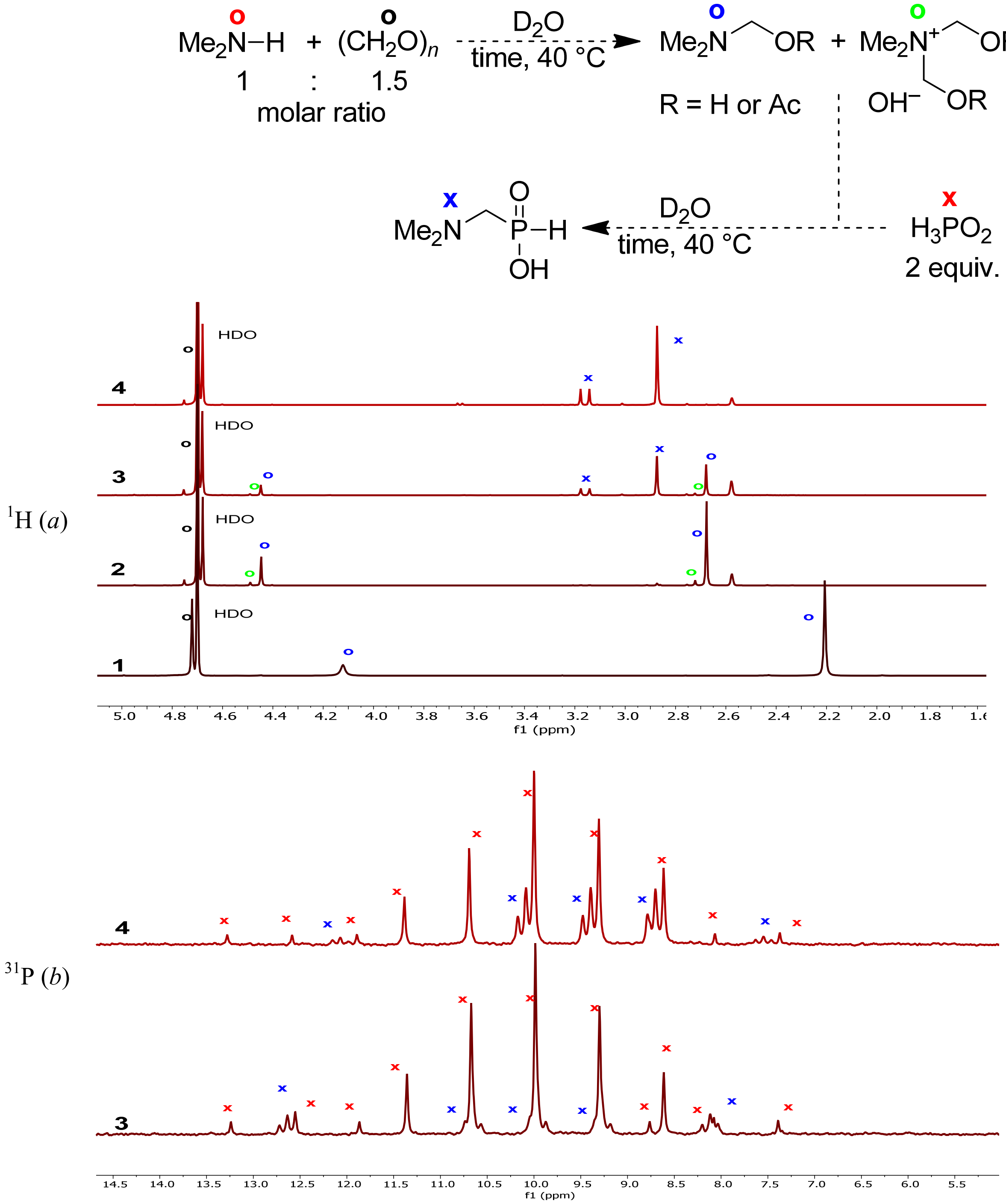

(***1***) 1 equiv. 40% aq. Me_2NH and 1.5 equiv. $(CH_2O)_n$ in D_2O at 180 min and 40 °C. (***2***) Addition of 1 equiv. 50% aq. H_3PO_2 to mixture in (***1***), 10 min at RT. (***3***) 240 min at 40 °C. (***4***) 4 d at 40 °C.

Figure S19

^{31}P NMR spectrum after addition of H_3PO_2 (1 equiv.) to a pre-mixed mixture of Me_2NH (as 40% aq. solution) and paraformaldehyde (in molar ratio 1:2; after 1 d at 40 °C in AcOH).The spectrum was measured after reaction time of 5 h at 40 °C. It showed formation of two *H*-phosphinic acids, $(CH_3)_2NCH_2PO_2H_2$ and $[(CH_3)_2N(CH_2OR)(CH_2PO_2H_2)]^+$ (where R = H or Ac). Spectrum was not referenced.

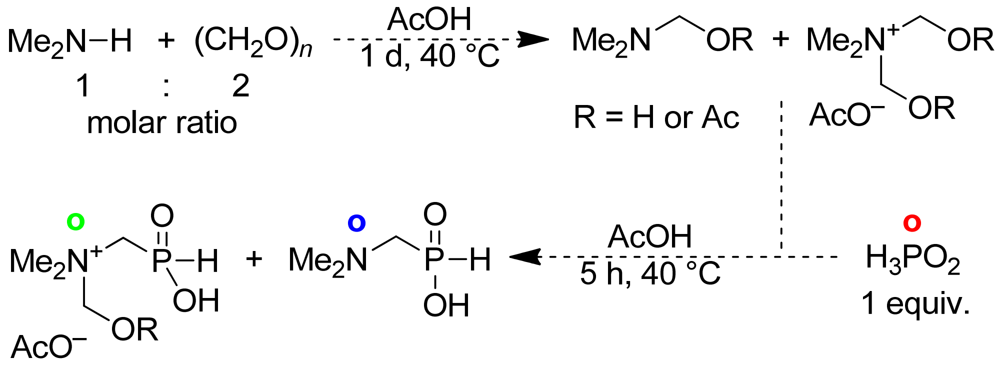

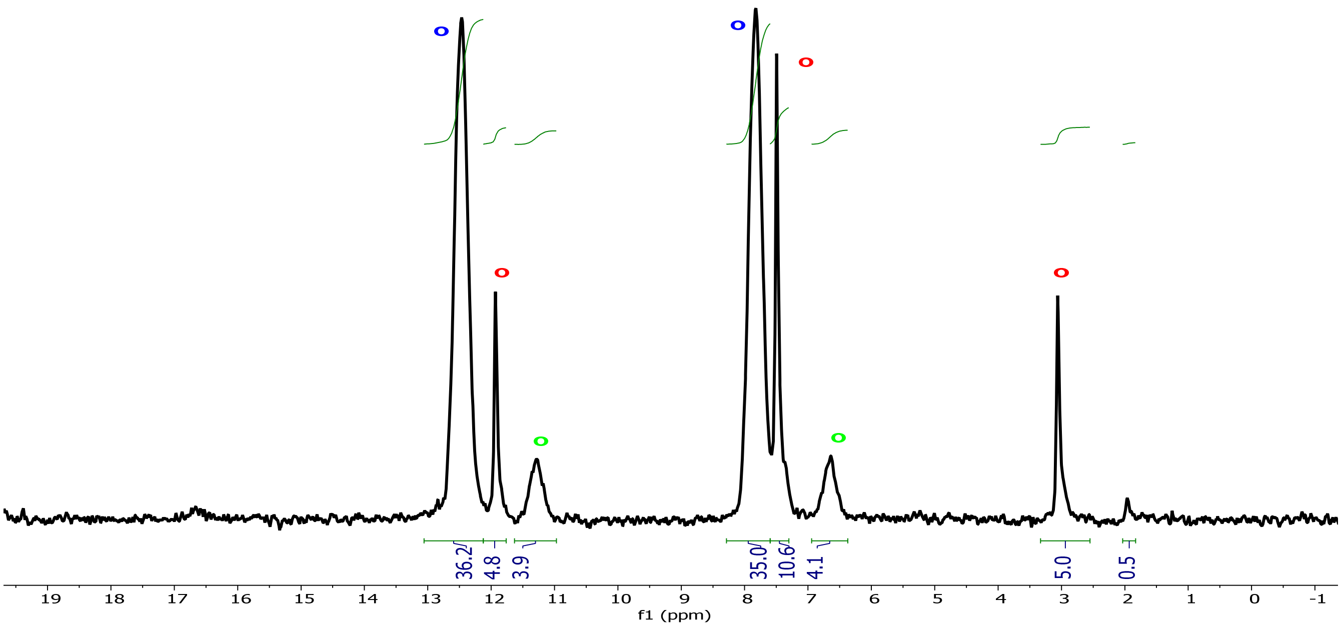

Figure S20

The ^{1}H, $^{13}C\{^{1}H\}$, ^{31}P and 2D (HSQC and HMBC) NMR spectra of mixtures after addition of H_3PO_2 (2.5 equiv.) to a mixture of Me_2NH (as 40% aq. solution) and paraformaldehyde in AcOH, see Figure S19. The spectra were measured after reaction time of 30 min and 40 °C. The spectra show formation of two *H*-phosphinic acids, $(CH_3)_2NCH_2PO_2H_2$ and $[(CH_3)_2N(CH_2OR)(CH_2PO_2H_2)]^+$ (where R = H or Ac). The ^{31}P NMR spectrum was not referenced. For a clear comparison of the reactions composition, overlays (denoted as (*x*)) of ^{1}H and $^{13}C\{^{1}H\}$ NMR spectra of pure paraformaldehyde (after 1 d at 40 °C) in AcOH with the spectra of the intermediate are shown.

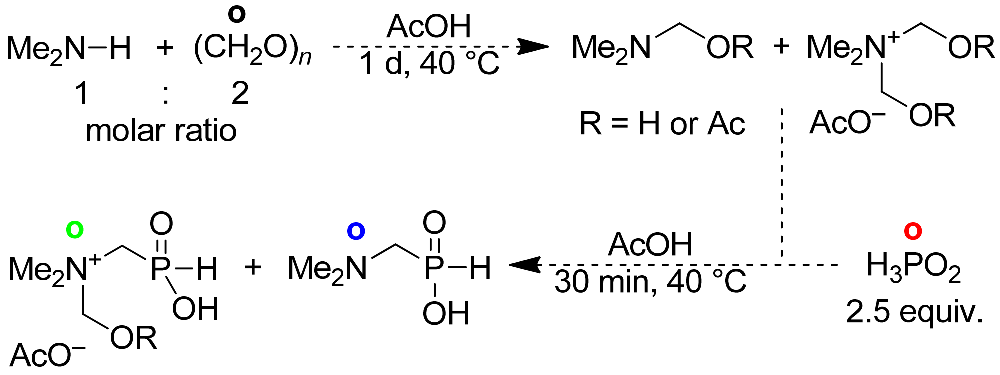

^{31}P

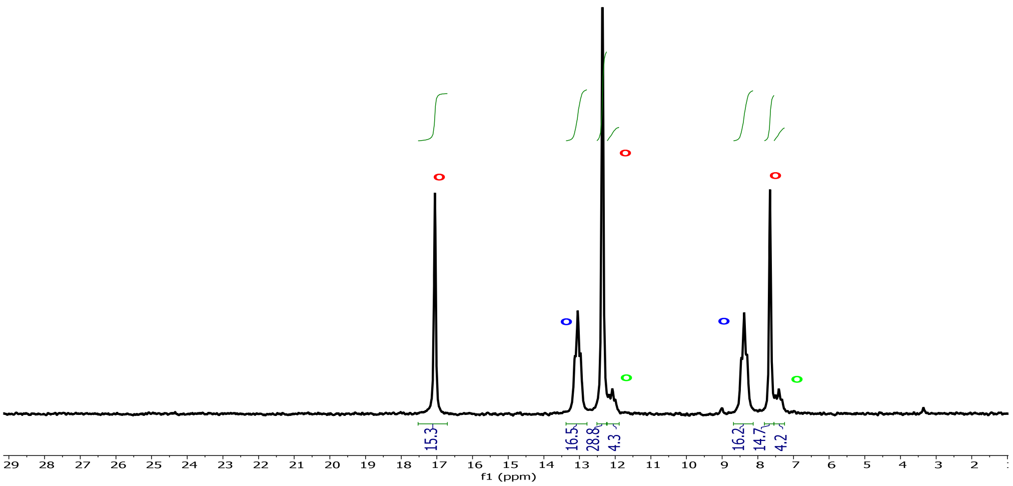

^{1}H

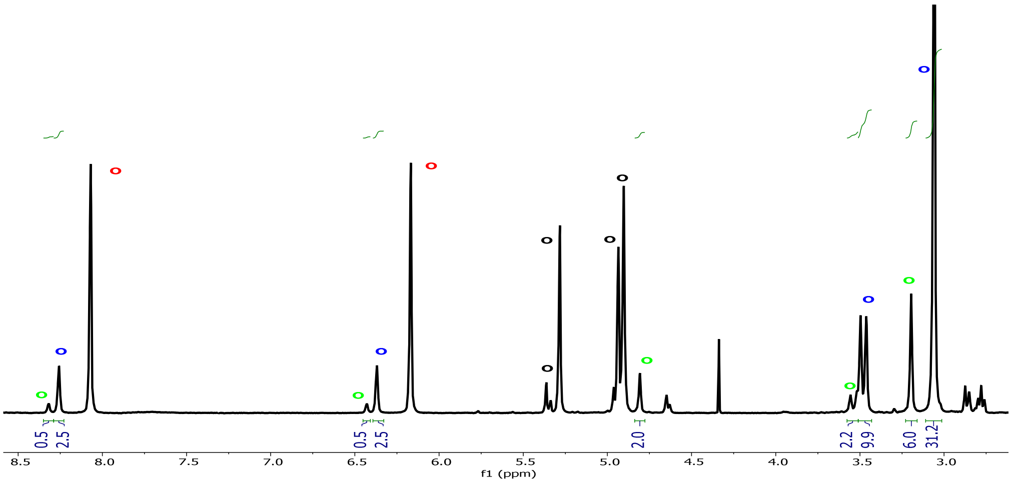

$^{13}C\{^{1}H\}$

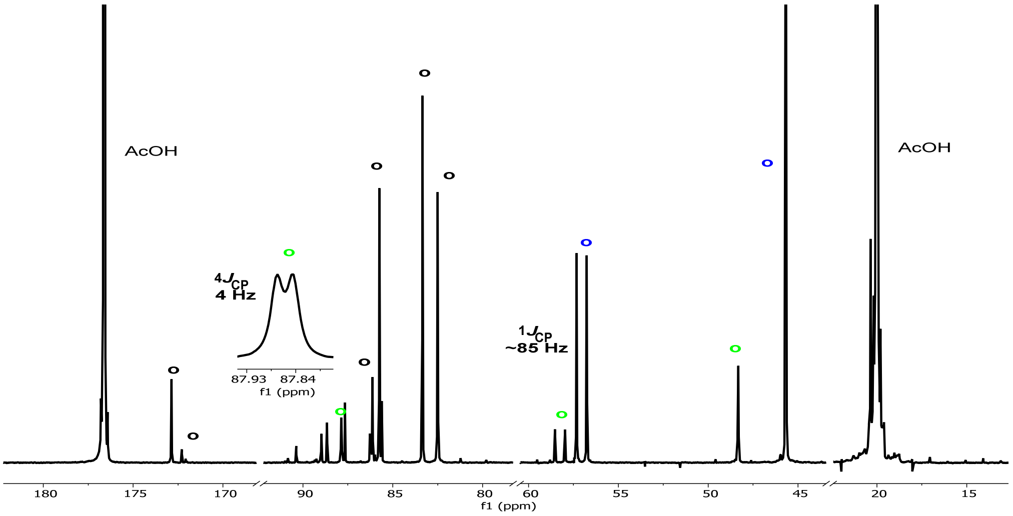

HSQC

(^{1}H–^{13}C)

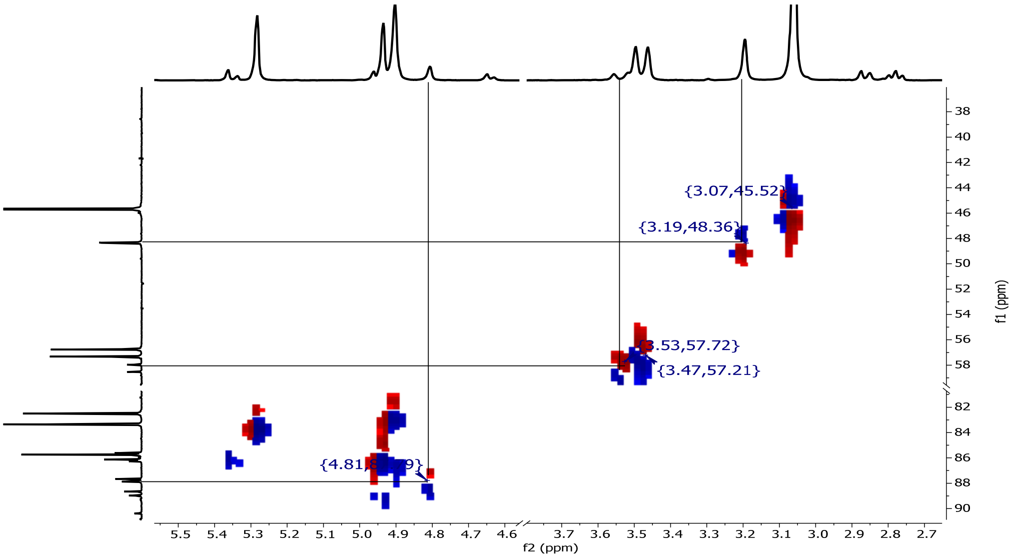

HMBC

(^{1}H–^{13}C)

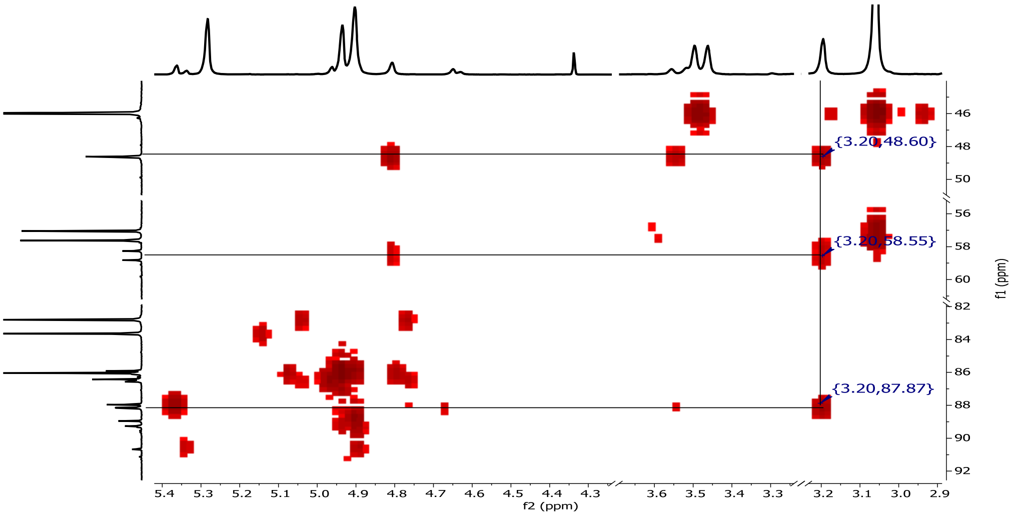

^{1}H (*x*)

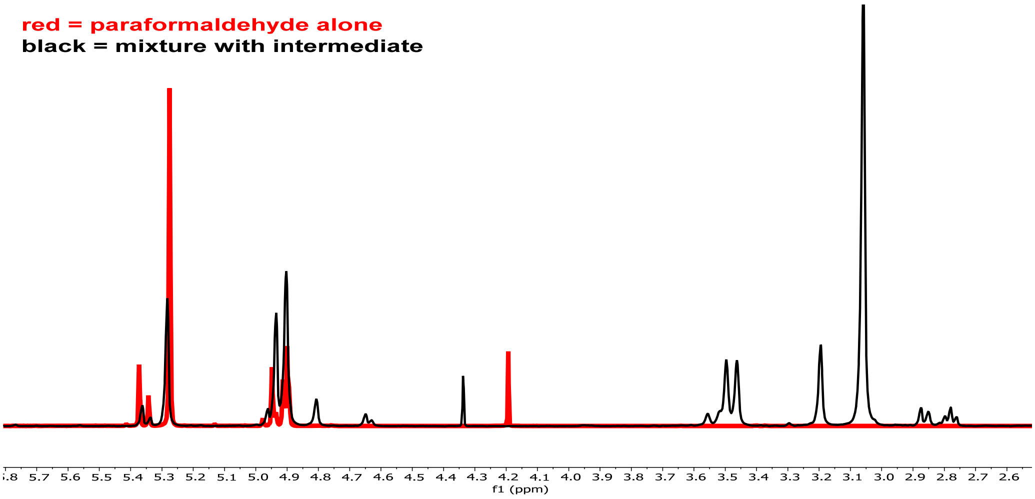
red = paraformaldehyde alone
black = mixture with intermediate
f1 (ppm)

^{13}C{^{1}H} (*x*)

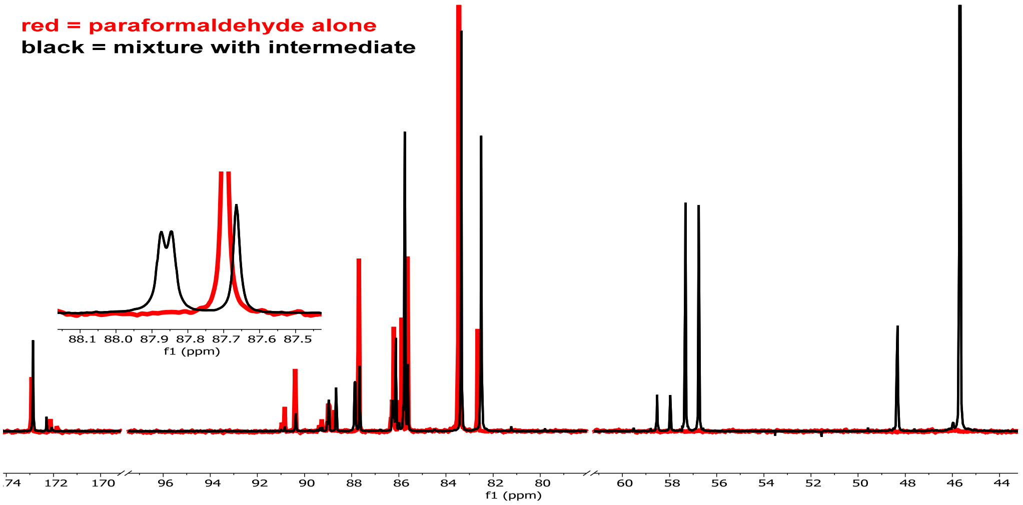
red = paraformaldehyde alone
black = mixture with intermediate
f1 (ppm)

Figure S21

Formation of two *H*-phosphinic acids after addition of H_3PO_2 (2.5 equiv.) to pre-mixed mixture of Me_2NH (as 40% aq. solution) and paraformaldehyde (in molar ratio 1:2, respectively; 1 d at 40 °C). The ^{31}P NMR spectra were measured regularly (reaction at 40 °C). Both *H*-phosphinic acids, $(CH_3)_2NCH_2PO_2H_2$ and $[(CH_3)_2N(CH_2OR)(CH_2PO_2H_2)]^+$ (where R = H or Ac), reach an equilibrium after ~30 min, and no change in ^{31}P NMR spectra was observed later.

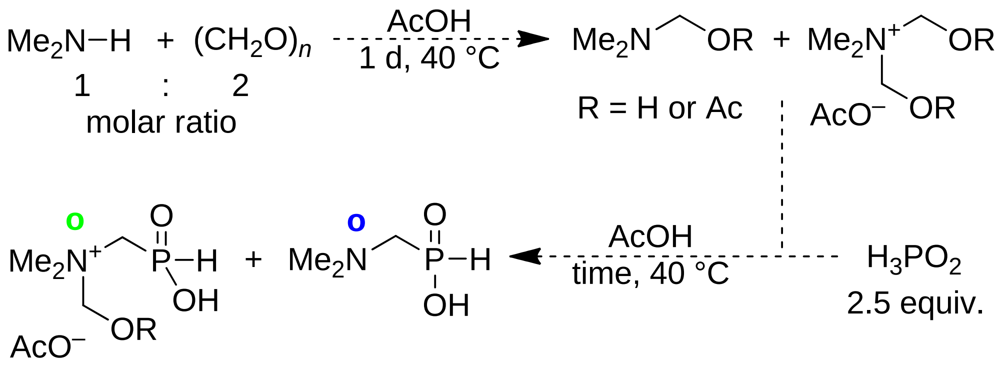

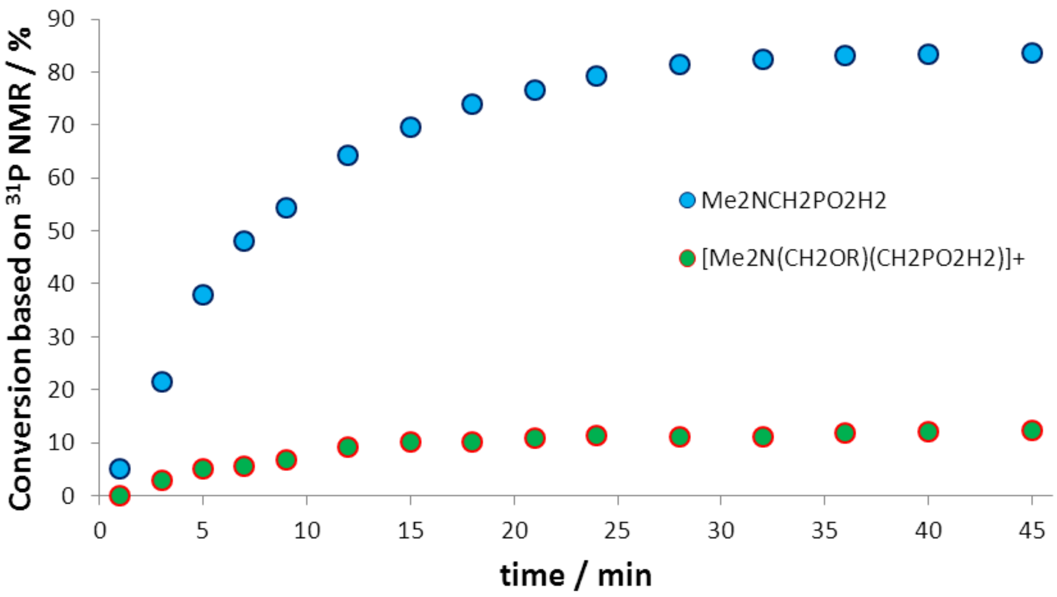

Figure S22

The ^{31}P NMR spectra (in AcOH) of a mixture prepared by reaction of H_3PO_2 (1 equiv.), Me_2NH (1 equiv.) and paraformaldehyde (2 equiv.) similarly as in Figures S18–21 (1 d at 40 °C, Mixture **A**). (***1***) Temperature of mixture **A** was elevated to 80 °C for 3.5 h. (***2***) Addition of conc. aq. HCl (~10–20 equiv.) to mixture (***1***); reaction time 2 h at 40 °C. (***3***) Addition of water excess (to get 20% v/v) to mixture (***2***); reaction time 20 min at 40 °C. (***4***) Addition of more water (another ~10% v/v) to mixture (***3***); reaction time 15 min at 40 °C. The ^{31}P NMR spectra were not referenced. Spectra of mixture (***1***) showed very slow hydrolysis of $[(CH_3)_2N(CH_2OR)(CH_2PO_2H_2)]^+$ (where R = H or Ac) to $(CH_3)_2NCH_2PO_2H_2$ at 80 °C.The addition of HCl did not alter significantly the composition of the reaction mixture (see mixture (***2***)). Addition of water quickly hydrolysed the cationic intermediate to the desired *H*-phosphinic acid (mixtures (***3***) and (***4***)). If analogous experiment was done with no HCl added, the intermediate was hydrolysed to *H*-phosphinic acid easily with water excess and mild heating (40 °C).

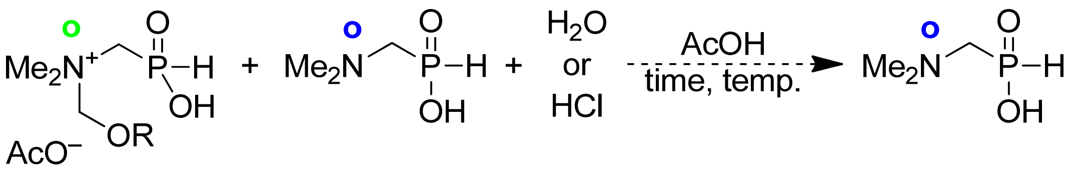

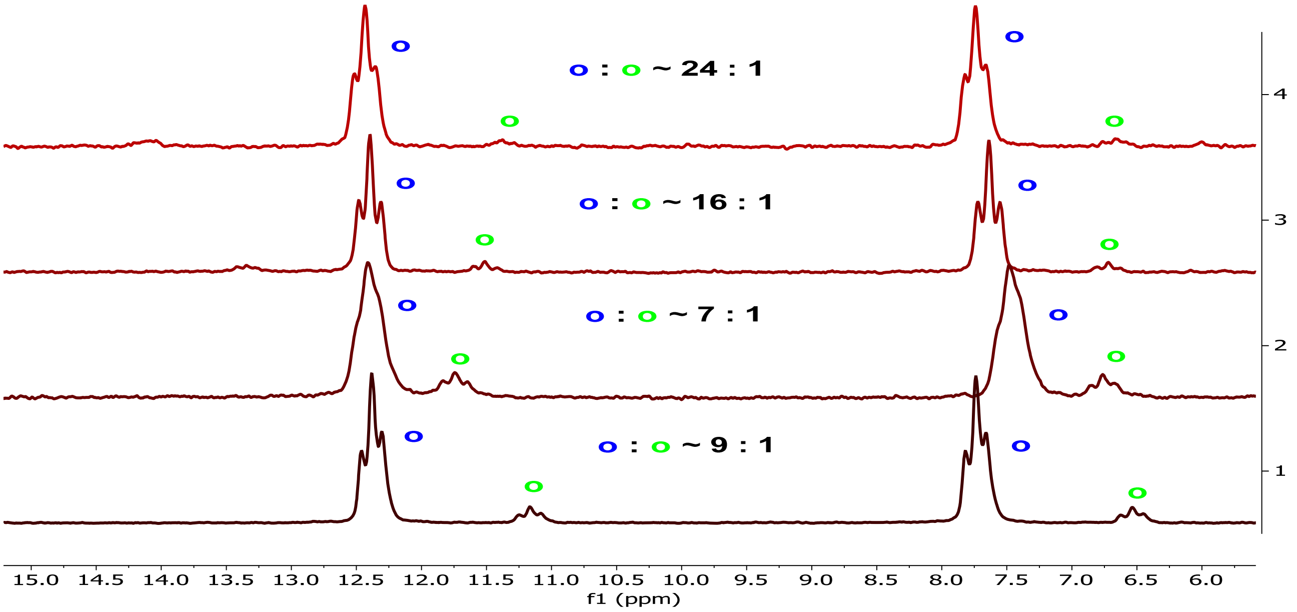

Figure S23

The ^{1}H NMR spectrum of $(Me_2N{=}CH_2)^{+}Cl^{-}$ immediately after dissolution in AcOH-d_4.

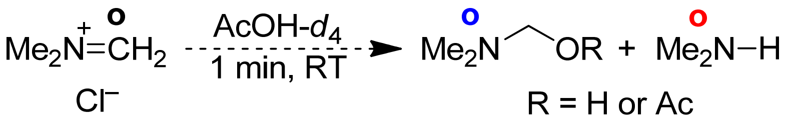

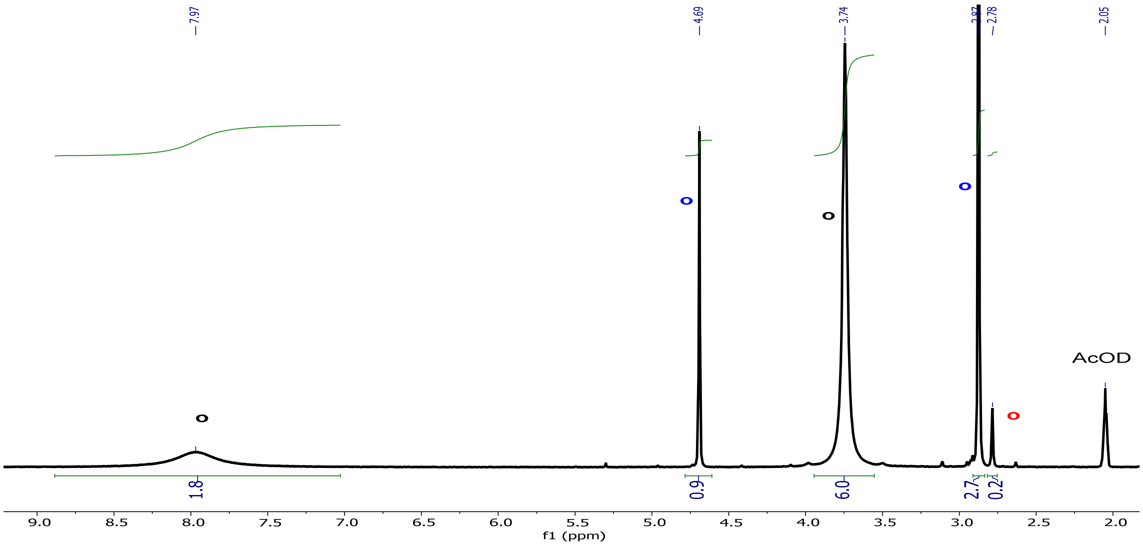

Figure S24

The ^{1}H and ^{31}P NMR spectra of reaction mixture of iminium salt $(Me_2N{=}CH_2)^{+}Cl^{-}$ and aq. H_3PO_2 (0.25 mmol of the iminium salt, molar ratio 1:1, AcOH-d_4 (0.4 ml), 40 °C). The ^{31}P NMR spectra are referenced to $\delta_P(H_3PO_2)$ = 10.0 ppm.

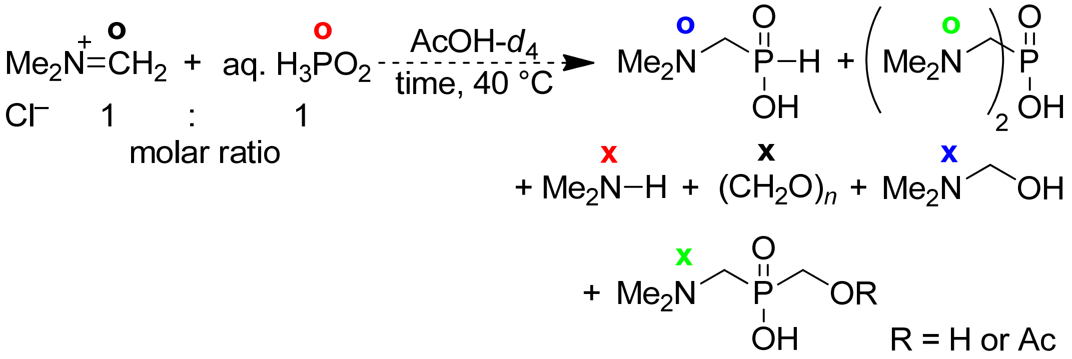

In the spectrum (**2**) and on-wards, δ_H of the same compounds are different than in (**1**) due to addition of aq. H_3PO_2. Complicated splitting of ^{31}P NMR signals is caused by ^{1}H–^{2}D exchange; thus, H_3PO_2 signals split by deuterium are not marked in the ^{31}P NMR spectra.

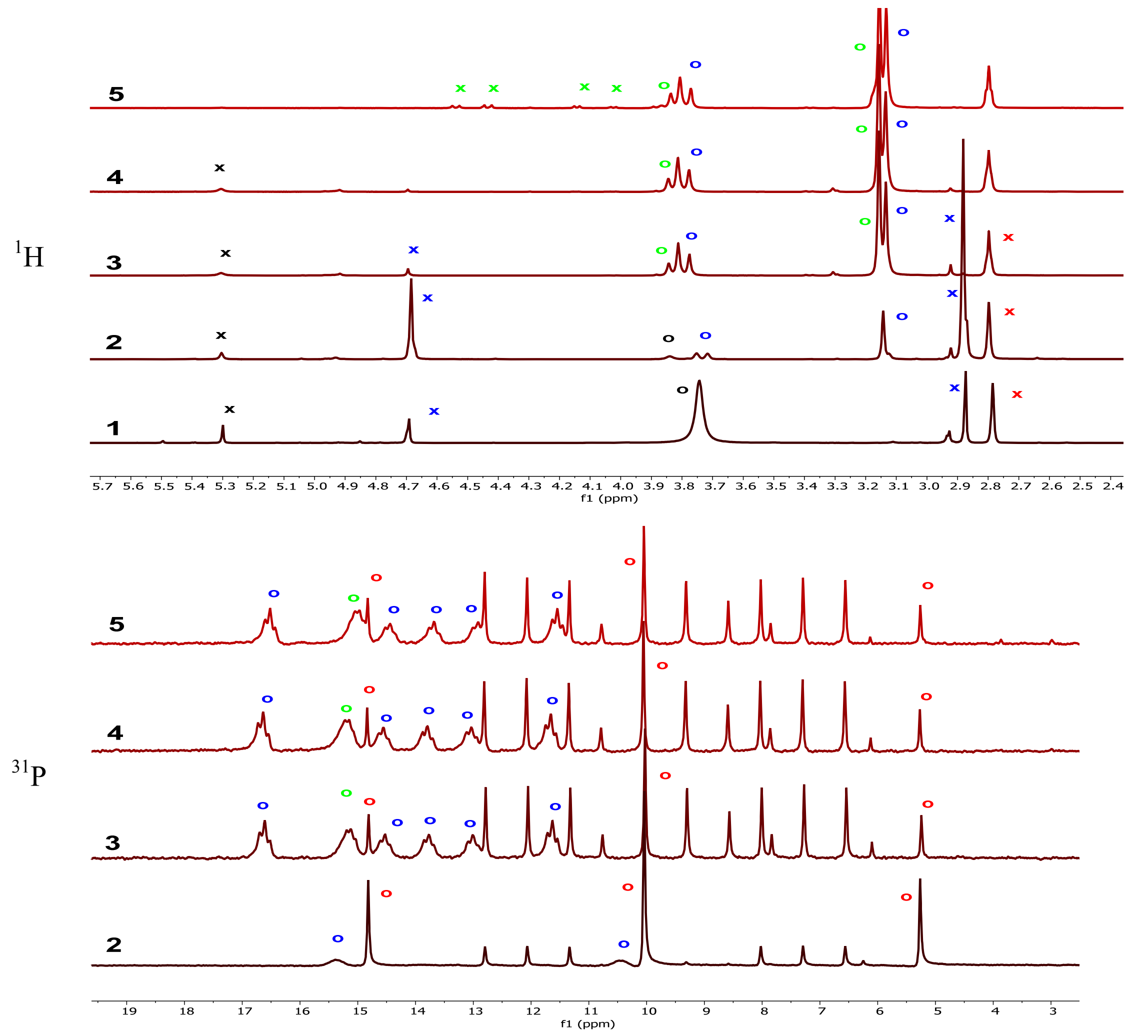

(**1**) $(Me_2N{=}CH_2)^+Cl^-$ (1 equiv.) in AcOH-d_4, 90 min, 40 °C. (**2**) Measured immediately after addition of 50% aq. H_3PO_2 (1 equiv.) to solution in (**1**). (**3**) Additional 15 min at 40 °C. (**4**) Additional 85 min at 40 °C. (**5**) After 1 d at 40 °C.

Figure S25

The ^{1}H and ^{31}P NMR spectra of reaction mixture containing iminium salt $(Me_2N{=}CH_2)^{+}Cl^{-}$ and anhydrous H_3PO_2 (0.25 mmol of the iminium salt, molar ratio 1:1, AcOH-d_4 (0.4 ml), 40 °C). Referenced to $\delta_P(H_3PO_2) = 10.0$ ppm. The ^{31}P NMR spectra contain complicated ^{31}P–^{2}D signal splitting.

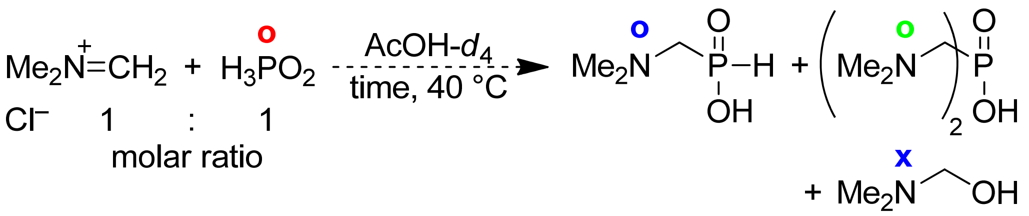

^{1}H

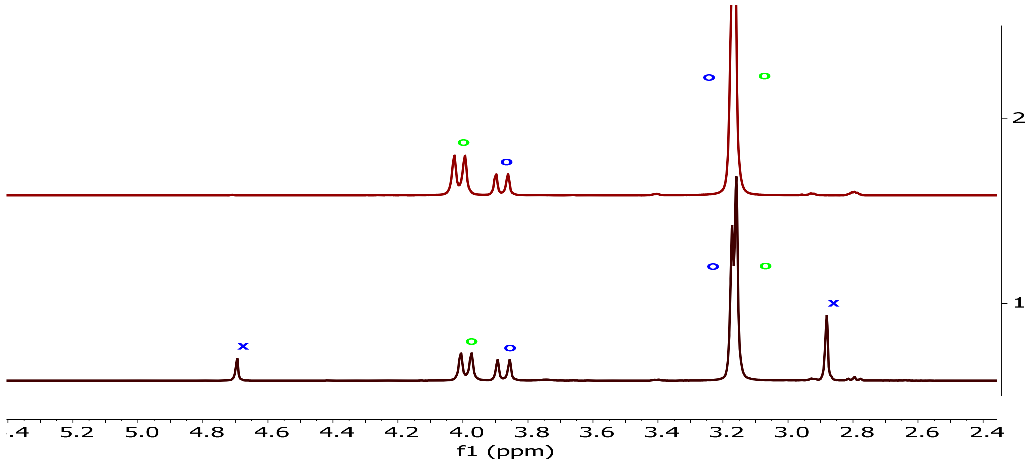

^{31}P

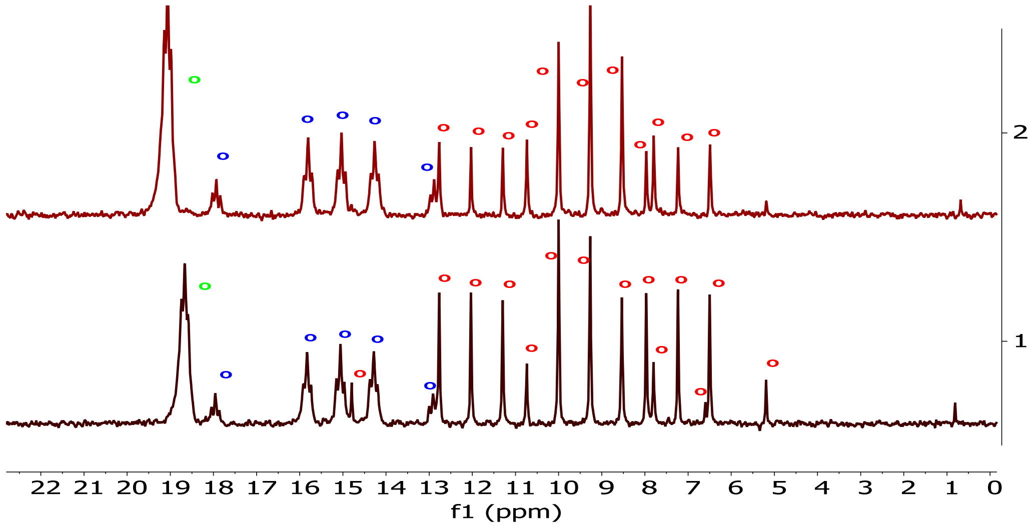

(***1***) Measured immediately after mixing and dissolution of $(Me_2N{=}CH_2)^{+}Cl^{-}$ and solid H_3PO_2 in AcOH-d_4.

(***2***) After 60 min at 40 °C.

Figure S26

The ^{1}H and ^{31}P NMR spectra of a mixture of $(Me_2N)_2CH_2$, anhydrous H_3PO_2 and D_2O (0.25 mmol of amine, molar ratio 1:1:4, AcOH-d_4 (0.4 ml), 40 °C); see description of spectra for more details. The ^{31}P NMR spectra were referenced to $\delta_P(H_3PO_2)$ = 10.0 ppm and they show complicated ^{31}P–^{2}D signal splitting. Overall final conversion to *H*-phosphinic and bis-substituted phosphinic acids (*i.e.* C–P–C compounds) was ~50 %; thus, 0.5 equiv. of Me_2NH remained unreacted as no more “formaldehyde” was available.

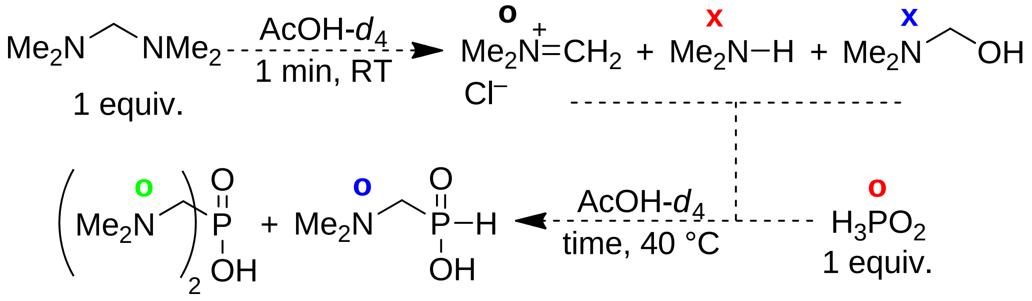

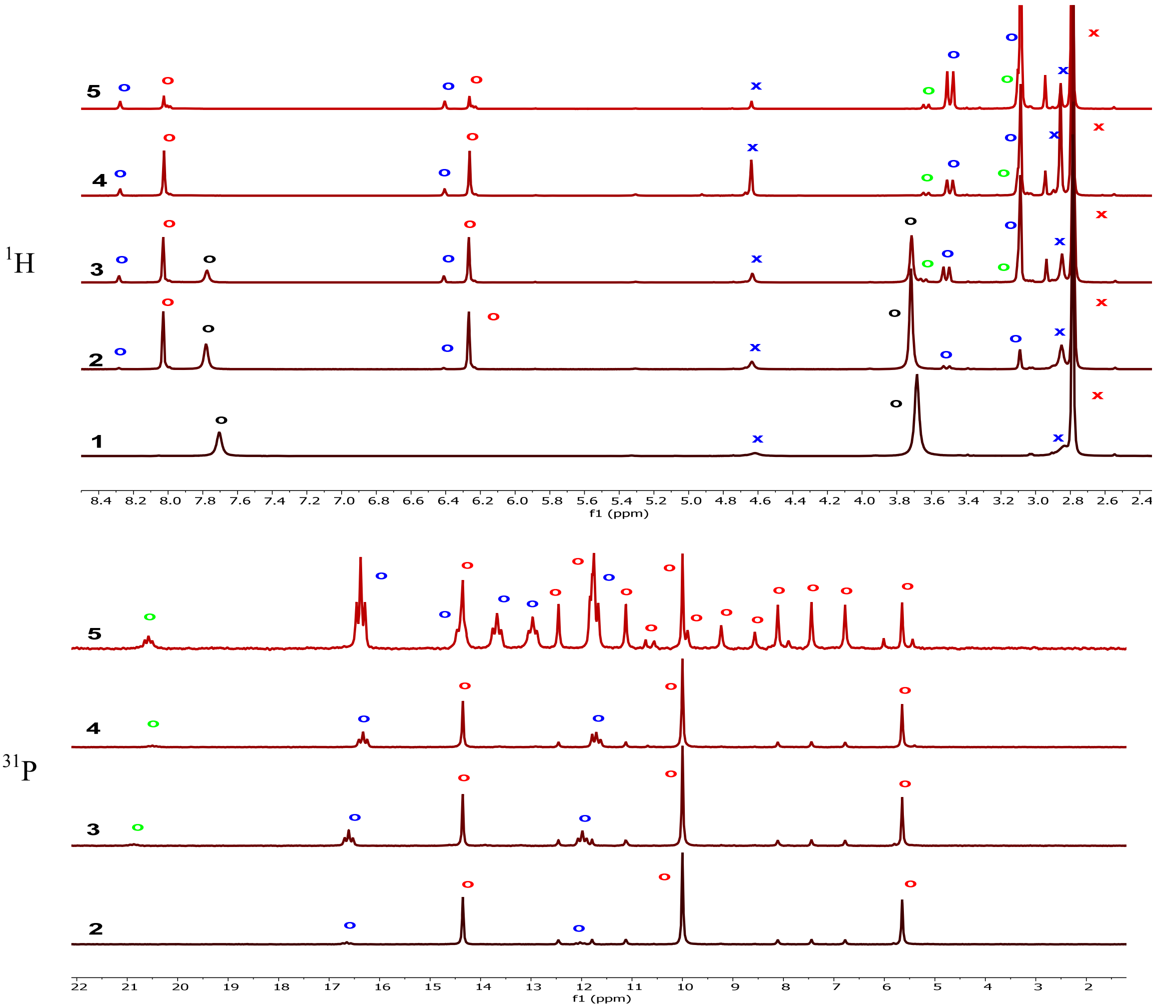

(***1***) Recorded immediately after dissolution of $(Me_2N)_2CH_2$ with AcOH-d_4. (***2***) Recorded after addition anhydrous H_3PO_2 (1 equiv.) to mixture (***1***). (***3***) After 120 min at 40 °C. (***4***) Measured immediately after addition of D_2O (4 equiv.) into mixture (***3***). (***5***) After 1 day at 40 °C.

Figure S27

Overlay of ^{1}H NMR spectra of mixture of decomposed $(Me_2N)_2CH_2$ (*i.e.* mainly to $[Me_2N{=}CH_2]^+$ and Me_2NH are present) and anhydrous H_3PO_2 (in molar ratio 1:1, in AcOH-d_4) before (**black**) and immediately after (**red**) addition of D_2O (4 equiv.). The signal intensity of iminium cation was changed due to its hydrolysis with D_2O and simultaneous formation of Me_2NCH_2OR (R = H or Ac). For more signals assignment, see Figure S26.

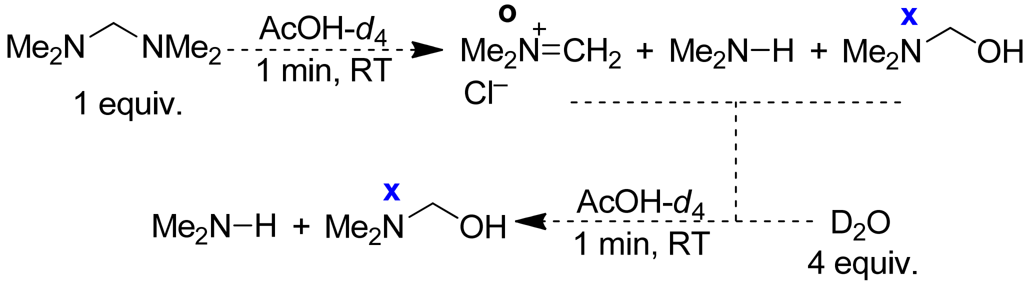

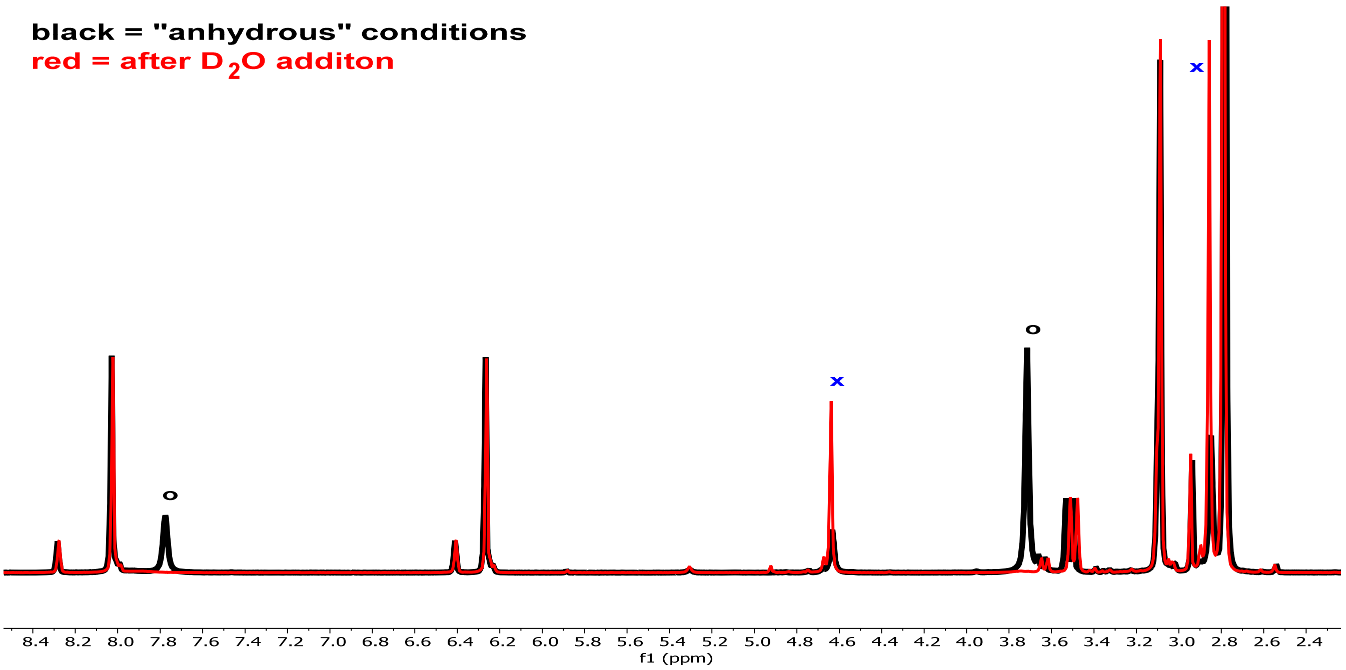

3. Synthesis of Starting Materials

BnNH–(CH_2)$_n$–NHBn and **BnNH–(CH_2)$_n$–NH–(CH_2)$_n$–NHBn** (n = 2 for Bn_2en and Bn_2dien-triamine, n = 3 for Bn_2prop-diamine and Bn_2diprop-triamine, and n = 6 for Bn_2hex-diamine and Bn_2dihex-triamine).

General procedure was reproduced from literature[3] with few applied changes. Mixture of the corresponding amine (1 equiv.), PhCHO (2.2 equiv.) and triethylamine (3 or 4 equiv., see Table S3) in MeOH (~120 mL) was left to react at room temperature for 6 h. Then, the mixture was cooled to 0 °C in ice bath and $NaBH_4$ (3 equiv.) was gradually added to an open reaction vessel. The reaction mixture was stirred at room temperture for 3 h and then was quenched with 1:1 aq. HCl (~2 mL). The solvents were evaporated to give an oily residue. Conc. aq. HCl (~20 mL) was added and the products were solidified after sonification. The product hydrochlorides were filtered off, washed twice with 1:1 aq. HCl (~10 mL), thrice with Et_2O (~10 mL) and dried in an oven (30 min., 100 °C). Yields are given in Table S3 and elementary analyses are given in Table S4. A single crystal of Bn_2dien trihydrochloride was obtained by acetone vapour diffusion into aqueous solution of the Bn_2dien hydrochloride.

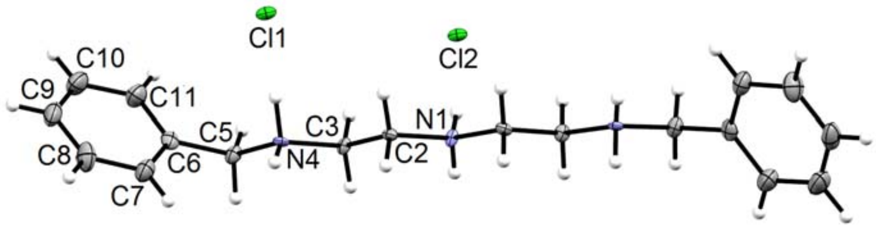

The amines in their acetate form were obtained on Dowex 1 in OH^--form (50 ml, 4×8 bed). The hydrochloride salts were dissolved in 10% AcOH (~20 ml), the solutions were applied on the column and the column was eluted with 20% AcOH (~150 ml). The eluates were evaporated to dryness *in vacuo* and the oily residues were directly used in the phospha-Mannich reaction.

Table S3

Reductive amination of alkyldiamines or dialkyltriamines with benzaldehyde, triethylamine and sodium boronhydride.

Starting amine	Mass of amine (g)	Volume of TEA (mL) and its equiv. in parenthesis	Volume of PhCHO (mL)	Mass of $NaBH_4$ (g)	Yield of HCl salt (%)
$H_2N–(CH_2)_3–NH_2$	0.70	3.9 (3)	2.2	1.0	60
$H_2N–(CH_2)_6–NH_2$	0.60	2.2 (3)	1.2	0.6	77
$[H_2N–(CH_2)_2–]_2NH$	1.00	5.4 (4)	2.3	1.1	89
$[H_2N–(CH_2)_3–]_2NH$	0.70	3.0 (4)	1.3	0.6	62
$[H_2N–(CH_2)_6–]_2NH$	1.50	3.9 (4)	1.6	0.8	84

[3] T. Pirali, G. Callipari, E. Ercolano, A. A. Genazzani, G. B. Giovenzana, and G. C. Tron, *Org. Lett.* **2008**, *10*, 4199–4202.

Table S4

Elementary analyses of prepared *N*-benzylated secondary polyamines.

Compound	C (*calc*)	H (*calc*)	N (*calc*)	Cl (*calc*)
BnHN–$(CH_2)_3$–NHBn·2HCl	61.87 (*62.39*)	7.13 (*7.39*)	8.56 (*8.56*)	21.99 (*21.66*)
BnHN–$(CH_2)_6$–NHBn·2HCl	64.81 (*65.03*)	7.79 (*8.19*)	7.52 (*7.58*)	19.92 (*19.19*)
[BnHN–$(CH_2)_2$–]$_2$NH·3HCl·3/2NaCl	45.51 (*45.00*)	5.91 (*5.87*)	8.86 (*8.75*)	31.85 (*33.20*)
[BnHN–$(CH_2)_3$–]$_2$NH·3HCl	56.85 (*57.08*)	7.44 (*7.66*)	9.91 (*9.98*)	25.57 (*25.27*)
[BnHN–$(CH_2)_6$–]$_2$NH·3HCl·3/2H_2O	58.70 (*58.70*)	8.25 (*8.90*)	7.77 (*7.90*)	21.76 (*19.99*)

1,3,5-tribenzyl-1,3,5-triazacyclohexane

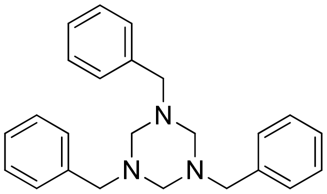

Synthesis was reproduced by published procedure.[4] Final product was recrystallized from hot toluene to remove traces of water.

*(*N*-Benzyl)-aminomethyl-*H*-phosphinic acid.*

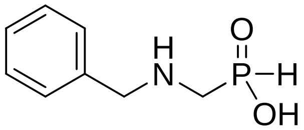

In 250-ml round-bottom flask, solid ("anhydrous") H_3PO_2 (12.5 g, 0.19 mol, 1 equiv.) was dissolved in toluene (~150 ml) and Me_3SiOEt (60 ml, 0.38 mol, 2 equiv.) was slowly added. Mixture was stirred at room temperature for 1 h and then *s*-triazine (*i.e.* 1,3,5-tribenzyl-1,3,5-triazacyclohexane; 18.0 g, 0.05 mol, 0.37 equiv.) was added. Suspension was stirred vigorously and heated at 50 °C (at the end of the reaction, the suspension dissolved) for 18 h. Then, 5% aq. NH_3 (25 ml) was added and mixture was stirred at 50 °C for another 30 min. The aqueous phase of biphasic mixture was collected and the organic phase was re-extracted with 5% aq. NH_3 (2 × 25 ml). The combined aqueous phases were then washed with toluene (25 ml). The aqueous phase was concentrated *in vacuo*. An oily residue was purified on strong cation exchanger (Dowex 50, 5×20-ml bed). Column was washed with water and product was eluted off with 10% aq. pyridine. Fractions containing pure product were combined and evaporated to dryness. An oily residue was dissolved on hot MeOH and left to crystallize on slow cooling of the solution in fridge. For faster crystallization, MeOH solution of the product was overlaid with Et_2O. Final product was obtained in a form of white polycrystalline powder. Total yield was 8.96 g, 25 % (for **M · ¼H_2O**). A single crystal was prepared by a slow cooling of boiling MeOH solution of the product.

[4]A. Makhloufi, W. Frank, and C. Ganter, *Organometallics* **2012**, *31*, 2001–2008.

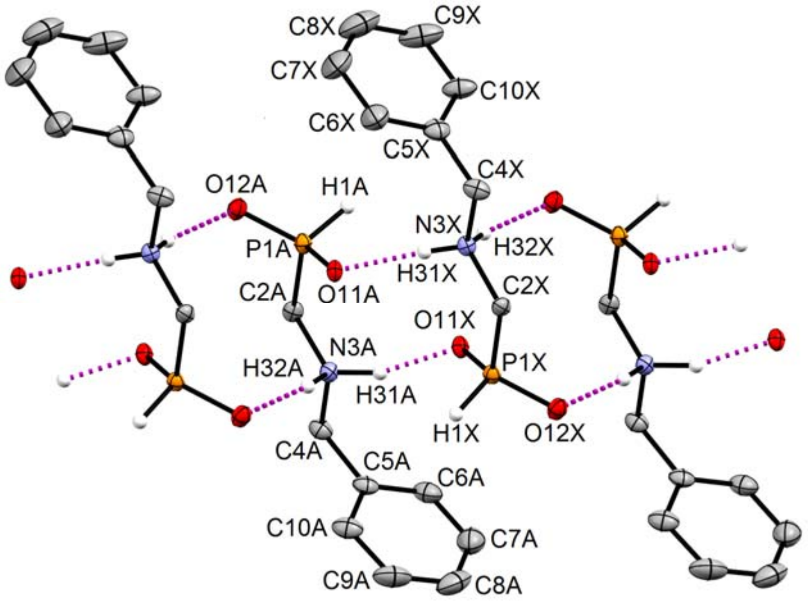

^{1}H NMR (D_2O + *t*BuOH, pD = 4.9 + 0.4): 3.14 (P–C<u>H</u>$_2$–N, d, $^{2}J_{HP}$ 10.9, $^{3}J_{HH}$ 1.9, 2H), 4.34 (Ph–C<u>H</u>$_2$–N, s, 2H), 7.15 (<u>H</u>–P, d, $^{1}J_{HP}$ 545.2, $^{3}J_{HH}$ 1.9, 1H), 7.45–7.56 (Ph, m, 5H)
^{13}C{^{1}H} NMR (D_2O + *t*BuOH, pD = 4.9 + 0.4): 46.6 (P–<u>C</u>H$_2$–N, d, $^{1}J_{CP}$ 86.0), 53.4 (Ph–C<u>H</u>$_2$–N, d, $^{3}J_{CP}$ 6.6), 130.0 (*o*-Ph), 130.5 (*p*-Ph), 130.7 (*m*-Ph), 131.0 (*i*-Ph)
^{31}P NMR (D_2O + *t*BuOH / 85% aq H_3PO_4, pD = 4.9 + 0.4): 11.6 (dt, $^{1}J_{PH}$ 545.1, $^{2}J_{PH}$ 10.9)
MS(+): 208 (208, $[M+Na]^+$), 371 (371, $[2M+H]^+$), 393 (393, $[2M+Na]^+$), 556 (556, $[3M+H]^+$)
MS(–): 739 (739, $[4M–H]^–$)
HRMS(+) (found (*calc*)): 186.0679 (*186.0678*, $C_8H_{13}NO_2P$), 371.1255 (*371.1290*, $C_{16}H_{25}N_2O_4P_2$)
TLC (conc. aq. NH_3 : EtOH = 1:{*x*}): 0.74 {5}, 0.68 {10}, 0.63 {20}, 0.55 {35}
EA (found (*calc* M · ¼H_2O)): C 50.96 (*50.66*), H 6.05 (*6.64*), N 7.35 (*7.39*), P 16.37 (*16.33*)

General procedure for secondary amines in Table 1 in the paper text.

*(*N,N-*Dibenzyl)-aminomethyl-*H-*phosphinic acid* ***1***.

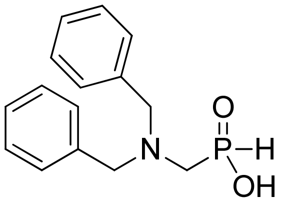

Procedure **B**.

From 192 µl (1.0 mmol) of Bn_2NH. Product crystallized after dissolving in boiling acetone and then was filtered off, washed twice with Et_2O and dried on air. White powder of **1** (214 mg, 78 %).

A single crystal was prepared by a slow cooling of hot acetone solution of **1**.

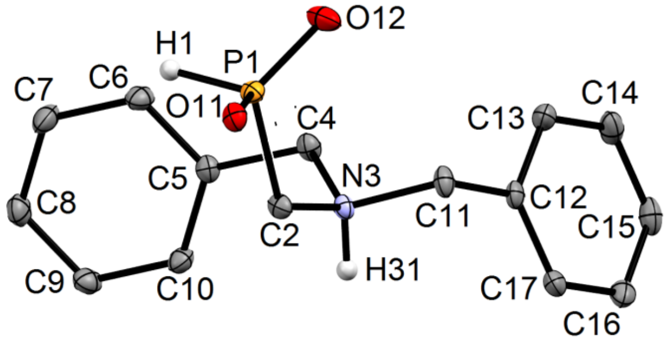

Characterization data were identical as published.[5]

[5] J. Kotek, P. Lebdušková, P. Hermann, L. Vander Elst, R. N. Muller, C. F. G. C. Geraldes, T. Maschmeyer, I. Lukeš, and J. A. Peters, *Chem. Eur. J*, **2003**, 9, 5899–5915.

*(*N,N-*Dimethyl)-aminomethyl*-H-*phosphinic acid* ***2***.

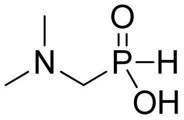

<u>Procedure **A**</u>.

From 113 μl (1.0 mmol) of 40% aq. Me_2NH. Product partially crystallized upon standing at room temperature.

Hygroscopic oil with a few crystals (117 mg, 95 %).

A single crystal was obtained on standing the oil of **2** for several weeks.

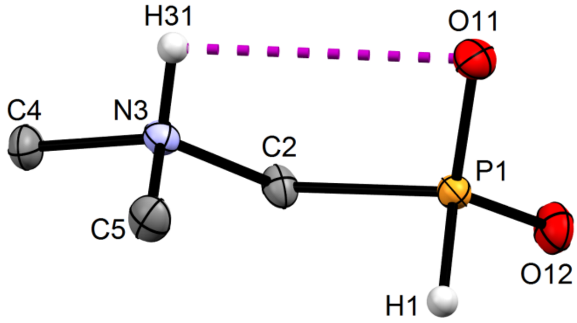

^{1}H NMR (D_2O + *t*BuOH, pD = 2.8 + 0.4): 3.02 (N–C<u>H</u>$_3$, d, $^{4}J_{HP}$ 0.9, 6H), 3.30 (P–C<u>H</u>$_2$–N, dd, $^{2}J_{HP}$ 10.4, $^{3}J_{HH}$ 1.7, 2H), 7.23 (<u>H</u>–P, dt, $^{1}J_{HP}$ 547.5, $^{3}J_{HH}$ 1.7, 1H)
^{13}C{^{1}H} NMR (D_2O + *t*BuOH, pD = 2.8 + 0.4): 45.8 (N–<u>C</u>H$_3$, d, $^{3}J_{CP}$ 4.8), 84.5 (P–<u>C</u>H$_2$–N, d, $^{1}J_{CP}$ 84.5)
^{31}P NMR (D_2O + *t*BuOH / 85% aq H_3PO_4, pD = 2.8 + 0.4): 9.7 (dt, $^{1}J_{PH}$ 548.1, $^{2}J_{PH}$ 10.4)
MS(+): 124 (124, $[M+H]^+$), 247 (247, $[2M+H]^+$)
MS(–): 122 (122, $[M-H]^-$), 245 (245, $[2M-H]^-$)
HRMS(+) (found (*calc*)): 124.0506 (*124.0527*,$C_3H_{11}NO_2P$), 247.0941 (*247.0977*, $C_6H_{21}N_2O_4P_2$)
TLC (conc. aq. NH_3 : EtOH = 1:{*x*}): 0.69 {5}, 0.48 {10}, 0.31 {20}, 0.28 {35}

*(*N,N-*Diethyl)-aminomethyl*-H-*phosphinic acid* ***3***.

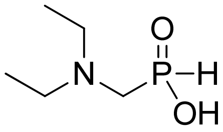

<u>Procedure **A**</u>.

From 103 μl (1.0 mmol) of Et_2NH.Viscous oil (140 mg, 93 %).

^{1}H NMR (D_2O + *t*BuOH, pD = 1.7 + 0.4): 1.33 (N–CH$_2$–C<u>H</u>$_3$, t, $^{3}J_{HH}$ 7.3, 6H), 3.26 (P–C<u>H</u>$_2$–N, dd, $^{2}J_{HP}$ 10.8, $^{3}J_{HH}$ 1.8, 2H), 3.28–3.47 (N–C<u>H</u>$_2$–CH$_3$, m, 4H), 7.25 (<u>H</u>–P, dt, $^{1}J_{HP}$ 547.1, $^{3}J_{HH}$ 1.6, 1H)
^{13}C{^{1}H} NMR (D_2O + *t*BuOH, pD = 1.7 + 0.4): 8.9 (N–CH$_2$–<u>C</u>H$_3$), 50.5 (N–<u>C</u>H$_2$–CH$_3$, d, $^{3}J_{CP}$ 4.2), 51.7 (P–<u>C</u>H$_2$–N, d, $^{1}J_{CP}$ 84.9)
^{31}P NMR (D_2O + *t*BuOH / 85% aq H_3PO_4, pD = 1.7 + 0.4): 10.4 (dt, $^{1}J_{PH}$ 547.7, $^{2}J_{PH}$ 10.8)
MS(+): 152 (152, $[M+H]^+$), 303 (303, $[2M+H]^+$)
MS(–): 150 (150, $[M-H]^-$), 301 (301, $[2M-H]^-$)
HRMS(+) (found (*calc*)): 152.0815 (*152.0840*,$C_5H_{15}NO_2P$), 303.1558 (*303.1603*, $C_{10}H_{29}N_2O_4P_2$)
TLC (conc. aq. NH_3 : EtOH = 1:{*x*}): 0.79 {5}, 0.57 {10}, 0.45 {20}, 0.42 {35}

*(*N,N-*Diisopropyl)-aminomethyl*-H-*phosphinic acid* **4**.

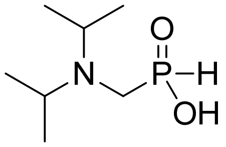

<u>Procedure **A**</u>.

From 140 µl (1.0 mmol) of *i*Pr_2NH. Product partially crystallized upon standing at room temperature. Viscous oil with a few crystals (168 mg, 94 %).

A single crystal was prepared on standing the oil of **4** for several weeks.

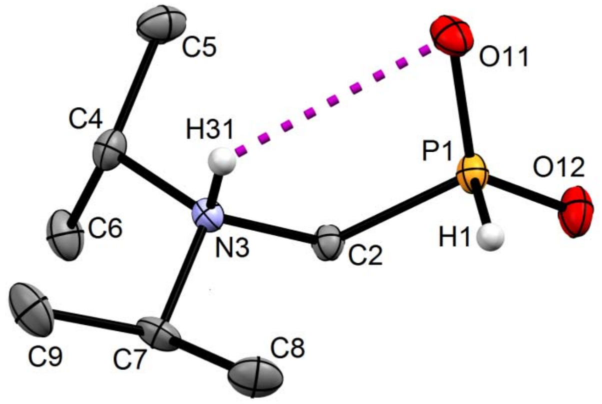

^{1}H NMR (D_2O + *t*BuOH, pD = 1.8 + 0.4): 1.31–1.46 (N–CH(–C<u>H</u>$_3$)$_2$, m, 12H), 3.81 (N–C<u>H</u>(–CH$_3$)$_2$, sept, $^{3}J_{HH}$ 6.4, 2H), 3.22 (P–C<u>H</u>$_2$–N, dd, $^{2}J_{HP}$ 11.2, $^{3}J_{HH}$ 1.8, 2H), 7.22 (<u>H</u>–P, dt, $^{1}J_{HP}$ 552.6, $^{2}J_{HH}$ 1.4, 1H)

^{13}C{^{1}H} NMR (D_2O + *t*BuOH, pD = 1.8 + 0.4): 16.9 + 18.7 (N–CH(–<u>C</u>H$_3$)$_2$), 47.4 (P–<u>C</u>H$_2$–N, d, $^{1}J_{CP}$ 83.0), 57.6 (N–<u>C</u>H(–CH$_3$)$_2$, d, $^{3}J_{CP}$ 2.7)

^{31}P NMR (D_2O + *t*BuOH / 85% aq H_3PO_4, pD = 1.8 + 0.4): 13.1 (dt, $^{1}J_{PH}$ 552.8, $^{2}J_{PH}$ 11.2)

MS(+): 180 (180, $[M+H]^+$), 360 (360, $[2M+H]^+$)

MS(–): 178 (178, $[M–H]^–$), 358 (358, $[2M–H]^–$)

HRMS(+) (found (*calc*)): 180.1114 (*180.1153*,$C_7H_{19}NO_2P$), 359.2173 (*359.2229*, $C_{14}H_{37}N_2O_4P_2$)

TLC (conc. aq. NH_3 : EtOH = 1:{*x*}): 0.79 {5}, 0.72 {10}, 0.57 {20}, 0.55 {35}

*(*N,N-*Dicyclohexyl)-aminomethyl-*H-*phosphinic acid* **5**.

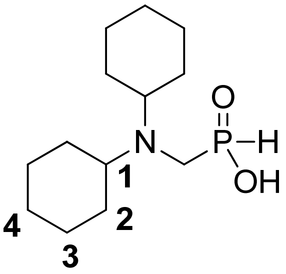

<u>Procedure **B**</u>.

From 199 µl (1.0 mmol) of Cy_2NH. Product crystallized after dissolving in boiling acetone and was filtered off, washed twice with Et_2O and dried on air. White polycrystalline powder, **5**·1/6H_2O (206 mg, 78 %).

A single crystal was obtained by a slow cooling of hot acetone solution of **5**.

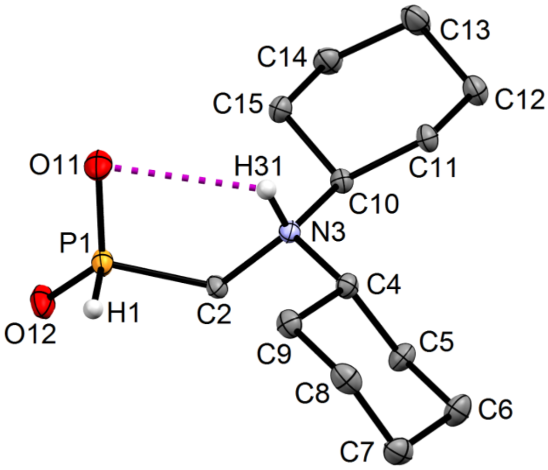

^{1}H NMR (D_2O + *t*BuOH, pD = 3.4 + 0.4): 1.10–1.27 (**4**, m, 2H), 1.28–1.43 (**3**, m, 4H), 1.43–1.62 (**2**, m, 4H), 1.63–1.72 (**4**, m, 2H), 1.85–1.96 (**3**, m, 4H), 2.00–2.10 (**2**, m, 4H), 3.29 (P–C<u>H</u>$_2$–N, dd, $^{2}J_{HP}$ 11.0, $^{3}J_{HH}$ 1.5, 2H), 3.43–3.55 (**1**, m, 2H), 7.20 (<u>H</u>–P, dt, $^{1}J_{HP}$ 552.5, $^{2}J_{HH}$ 1.5, 1H)

^{13}C{^{1}H} NMR (D_2O + *t*BuOH, pD = 3.4 + 0.4): 25.0 (**4**), 25.1 + 25.3 (**3**), 27.4 +29.1 (**2**), 48.4 (P–<u>C</u>H$_2$–N, d, $^{1}J_{CP}$ 83.2), 64.5 (**1**, d, $^{3}J_{CP}$ 2.6)

^{31}P NMR (D_2O + *t*BuOH / 85% aq H_3PO_4, pD = 3.4 + 0.4): 13.3 (dt, $^{1}J_{PH}$ 552.3, $^{2}J_{PH}$ 11.0)

MS(+): 260 (260, $[M+H]^+$), 519 (519, $[2M+H]^+$)

MS(–): 258 (258, $[M-H]^-$), 517 (517, $[2M-H]^-$)

HRMS(+) (found (*calc*)): 260.1741 (*260.1779*,$C_{13}H_{27}NO_2P$), 519.3419 (*519.3481*, $C_{26}H_{53}N_2O_4P_2$)

TLC (conc. aq. NH_3 : EtOH = 1:{*x*}): 0.90 {5}, 0.83 {10}, 0.76 {20}, 0.75 {35}

EA (found (*calc* M · 1/6H_2O)): C 59.63 (*59.52*), H 9.81 (*10.12*), N 5.21 (*5.34*), P 11.91 (*11.81*)

(N-*Benzyl)-(*N-*methyl)-aminomethyl*-H-*phosphinic acid* ***6***.

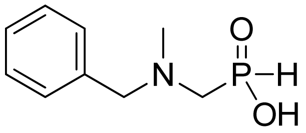

Procedure **A**.

From 120 mg (1.0 mmol) of Bn(Me)NH. Product was isolated as off-white powder after evaporation. Slightly hygroscopic powder, **6**·1/6H_2O (193 mg, 95 %).

^{1}H NMR (D_2O + *t*BuOH, pD = 5.9 + 0.4): 2.93 (C<u>H</u>$_3$–N, s, 3H), 3.38 (N–C<u>H</u>$_2$–P, d, $^2J_{HP}$ 10.5, 2H), 4.46 (N–C<u>H</u>$_2$–Ph, s, 2H), 7.17 (H–P, dt, $^1J_{HP}$ 549.2, $^3J_{HH}$ 1.7, 1H), 7.50–7.60 (Ph, m, 5H)
^{13}C{^{1}H} NMR (D_2O + *t*BuOH, pD = 5.9 + 0.4): 42.5 (<u>C</u>H$_3$–N, d, $^3J_{CP}$ 4.3), 55.3 (N–<u>C</u>H$_2$–P, d, $^1J_{CP}$ 83.9), 62.5 (N–<u>C</u>H$_2$–Ph, d, $^3J_{CP}$ 4.6), 129.5 (*i*-Ph), 130.0 (*m*-Ph), 131.0 (*p*-Ph), 131.9 (*o*-Ph)
^{31}P NMR (D_2O + *t*BuOH / 85% aq H_3PO_4, pD = 5.9 + 0.4): 10.0 (dt, $^1J_{PH}$ 549.0, $^2J_{PH}$ 10.6)
MS(+): 222 (222, [M+Na]$^+$), 421 (421, [2M+Na]$^+$), 620 (620, [3M+Na]$^+$)
MS(–): 198 (198, [M–H]$^-$), 397 (397, [2M–H]$^-$)
HRMS(+) (found (*calc*)): 222.0655 (*222.0654*,$C_9H_{14}NO_2PNa$)
TLC (conc. aq. NH_3 : EtOH = 1:{*x*}): 0.74 {5}, 0.71 {10}, 0.68 {20}, 0.64 {35}
EA (found (*calc* M · 1/6H_2O)): C 53.39 (*53.46*), H 6.90 (*7.15*), N 6.92 (*6.93*), P 15.24 (*15.32*)

1-(piperidinyl)methyl-H-*phosphinic acid* ***7***.

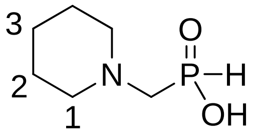

Procedure **A**.

From 99 µl (1.0 mmol) of piperidine. Product was isolated as viscous oil (158 mg, 97 %).

^{1}H NMR (D_2O + *t*BuOH, pD = 2.3 + 0.4): 1.44–1.56 (**3**, m, 1H), 1.70–1.80 (**3**, m, 1H), 1.74–1.86 (**2**, m, 2H), 1.90–2.01 (**2**, m, 2H), 3.05–3.16 (**1**, m, 2H), 3.14 (P–C<u>H</u>$_2$–N, d, $^2J_{HP}$ 10.7, $^3J_{HH}$ 1.7, 2H), 3.62–3.72 (**1**, m, 2H), 7.25 (<u>H</u>–P, dt, $^1J_{HP}$ 547.4, $^2J_{HH}$ 1.5, 1H)
^{13}C{^{1}H} NMR (D_2O + *t*BuOH, pD = 2.3 + 0.4): 21.4 (**3**), 23.4 (**2**), 56.3 (**1**), 56.7 (P–<u>C</u>H$_2$–N, d, $^1J_{CP}$ 90.2)
^{31}P NMR (D_2O + *t*BuOH / 85% aq H_3PO_4, pD = 2.3 + 0.4): 9.7 (dt, $^1J_{PH}$ 547.7, $^2J_{PH}$ 10.7)
MS(+): 164 (164, [M+H]$^+$), 327 (327, [2M+H]$^+$), 349 (349, [2M+Na]$^+$)
MS(–): 162 (162, [M–H]$^-$), 325 (325, [2M–H]$^-$)
HRMS(+) (found (*calc*)): 164.0816 (*164.0840*, $C_6H_{15}NO_2P$), 327.1571 (*327.1603*, $C_{12}H_{29}N_2O_4P_2$)
TLC (conc. aq. NH_3 : EtOH = 1:{*x*}): 0.76 {5}, 0.62 {10}, 0.48 {20}, 0.47 {35}

*(1-Morpholino)methyl-*H*-phosphinic acid* ***8***.

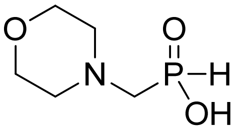

<u>Procedure **A**</u>.

From 87 µl (1.0 mmol) of morpholine. Product partially crystallized upon standing at room temperature. Viscous oil with a few crystals (152 mg, 92 %).

A single crystal was prepared on standing the oil of **8** for several weeks.

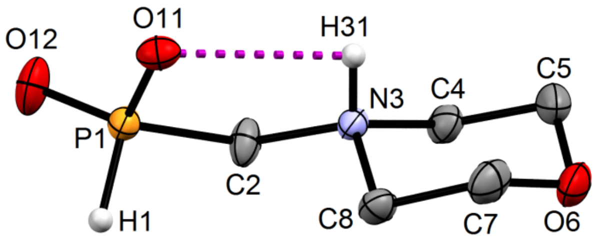

^{1}H NMR (D_2O + *t*BuOH, pD = 1.8 + 0.4): 3.33 (P–C$\underline{H}_2$–N, dd, $^{2}J_{HP}$ 10.6, $^{3}J_{HH}$ 1.8, 2H), 3.28–3.47 (N–C$\underline{H}_2$–CH_2, m, 2H), 3.59–3.78 (N–C$\underline{H}_2$–CH_2, m, 2H), 3.80–4.00 (O–C$\underline{H}_2$–CH_2, m, 2H), 4.00–4.22 (O–C$\underline{H}_2$–CH_2, m, 2H), 7.27 ($\underline{H}$–P, dt, $^{1}J_{HP}$ 549.7, $^{3}J_{HH}$ 1.7, 1H)
^{13}C{^{1}H} NMR (D_2O + *t*BuOH, pD = 1.8 + 0.4): 54.5 (N–$\underline{C}H_2$–CH_2, d, $^{3}J_{CP}$ 4.9), 56.8 (P–$\underline{C}H_2$–N, d, $^{1}J_{CP}$ 83.1), 64.4 (O–$\underline{C}H_2$–CH_2, d, $^{4}J_{CP}$ 0.4)
^{31}P NMR (D_2O + *t*BuOH / 85% aq H_3PO_4, pD = 1.8 + 0.4): 8.9 (dt, $^{1}J_{PH}$ 549.9, $^{2}J_{PH}$ 10.6)
MS(+): 166 (166, $[M+H]^+$), 331 (331, $[2M+H]^+$)
MS(–): 164 (164, $[M–H]^–$), 329 (329, $[2M–H]^–$)
HRMS(+) (found (*calc*)): 166.0609 (*166.0633*, $C_5H_{13}NO_3P$), 331.1155 (*331.1188*, $C_{10}H_{25}N_2O_6P_2$)
TLC (conc. aq. NH_3 : EtOH = 1:{*x*}): 0.70 {5}, 0.60 {10}, 0.45 {20}, 0.44 {35}

*(*N*-Benzyl)-*N*-(2,2,2-trifluoromethyl)-aminomethylphosphonic acid* **9**.

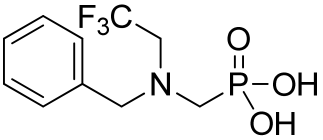

In 4-ml vial, (*N*-benzyl)-2,2,2-trifluoroethylamine (47 mg, 0.25 mmol, 1 equiv.), paraformaldehyde (15 mg, 2.0 mmol, 2 equiv.), and H_3PO_2 (as 50% aq. solution, 36 mg, 1.1 mmol, 1.1 equiv.) were mixed with glacial AcOH (2 ml). The suspension heated up to 40 °C for 1 day and conversion was determined by ^{31}P NMR. Then, solvents were removed by rotary evaporator and oily residue was purified by strong cation exchanger (Dowex 50, 3×10-cm bed) and the column was washed with water. Crude product was eluted off with 3% aq. HCl and the fraction was evaporated *in vacuo* to get viscous oil (4 mg, 5 %).

^{1}H NMR (D_2O + *t*BuOH, pD = 1.4 + 0.4): 3.43 (P–C<u>H</u>$_2$–N, d, $^2J_{HP}$ 12.2, 2H), 4.17 (CF_3–C<u>H</u>$_2$–N, q, $^3J_{HF}$ 8.9, 2H), 4.64 (Ph–C<u>H</u>$_2$–N, s, 2H)
^{13}C{^{1}H} NMR (D_2O + *t*BuOH, pD = 1.4 + 0.4): 50.6 (P–<u>C</u>H$_2$–N, d, $^1J_{CP}$ 137.3), 52.8 (CF_3–<u>C</u>H$_2$–N, dd, $^2J_{CF}$ 33.8, $^3J_{CP}$ 4.0), 60.8 (Ph–<u>C</u>H$_2$–N), 123.1 (<u>C</u>F$_3$–CH$_2$–N, q, $^1J_{CF}$ 278.7), 129.5 (*i*-Ph), 129.6 (*m*-Ph), 130.6 (*p*-Ph), 131.7 (*o*-Ph)
^{31}P NMR (D_2O + *t*BuOH / 85% aq H_3PO_4, pD = 1.4 + 0.4): 9.3 (t, $^2J_{PH}$ 12.2)
^{19}F NMR (D_2O + *t*BuOH / 0.1 M TFA in D_2O,[6] pD = 1.4 + 0.4): –65.20 (t, $^3J_{FH}$ 8.9)
MS(–): 282 (282, [M–H]$^-$), 565 (565, [355–H]$^-$)
HRMS(+) (found (*calc*)): 284.0664 (*284.0658*, $C_6H_{16}N_2O_2P$)
TLC (conc. aq. NH_3 : EtOH = 1:{*x*}): 0.76 {1}, 0.56 {5}, 0.33 {10}, 0.23 {20}, 0.03 {35}

[6] C. P. Rosenau, B. J. Jelier, A. D. Gossert, and A. Togni, *Angew. Chem. Int. Ed.* **2018**, *57*, 9528–9533. δ_F (0.1 M TFA in D_2O) = −75.51 ppm.

(N-*Methyl)-(*N-*carboxymethyl)-aminomethyl*-H-*phosphinic acid* ***10***.

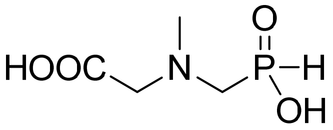

Procedure **A**.

From 89 mg (1.0 mmol) of sarcosine (*i.e.* *N*-Me-glycine). A residue obtained after solvent evaporation was triturated in boiling MeOH and the suspension was left to cool in fridge. Product was filtered off, washed twice with Et_2O and dried on air. White powder (115 mg, 69 %).

A single crystal was obtained by a slow cooling of boiling MeOH solution of **10**.

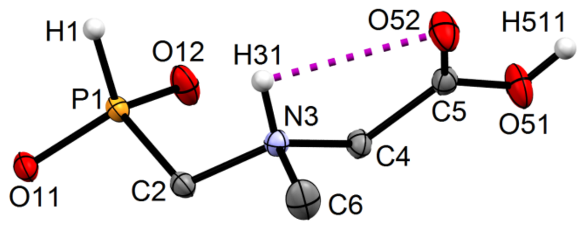

^{1}H NMR (D_2O + *t*BuOH, pD = 1.3 + 0.4): 3.13 (C<u>H</u>$_3$–N, s, 3H), 3.39 (P–C<u>H</u>$_2$–N, dd, $^{2}J_{HP}$ 10.6, $^{3}J_{HH}$ 1.7, 2H), 4.20 (HOOC–C<u>H</u>$_2$–N, s, 2H), 7.27 (<u>H</u>–P, dt, $^{1}J_{HP}$ 551.5, $^{3}J_{HH}$ 1.7, 1H)
^{13}C{^{1}H} NMR (D_2O + *t*BuOH, pD = 1.3 + 0.4): 44.4 (<u>C</u>H$_3$–N, d, $^{3}J_{CP}$ 3.7), 56.3 (P–<u>C</u>H$_2$–N, d, $^{1}J_{CP}$ 86.8), 58.6 (HOOC–<u>C</u>H$_2$–N, d, $^{3}J_{CP}$ 4.9), 168.9 (HOO<u>C</u>–CH$_2$)
^{31}P NMR (D_2O + *t*BuOH / 85% aq H_3PO_4, pD = 1.3 + 0.4): 9.6 (dt, $^{1}J_{PH}$ 551.5, $^{2}J_{PH}$ 10.6)
MS(+): 168 (168, $[M+H]^+$), 335 (335, $[2M+H]^+$)
MS(–): 166 (166, $[M–H]^-$), 333 (333, $[2M+H]^+$)
HRMS(+) (found (*calc*)): 168.0399 (*168.0426*, $C_4H_{11}NO_4P$), 335.0734 (*335.0773*, $C_8H_{21}N_2O_8P_2$)
TLC (conc. aq. NH_3 : EtOH = 1:{*x*}): 0.39 {5}, 0.18 {10}, 0.14 {20}, 0.09 {35}
EA(found (*calc* M)): C 28.47 (*28.75*), H 5.77 (*6.03*), N 8.15 (*8.38*), P 18.05 (*18.54*)

*(*N*-Benzyl)-(*N*-carboxymethyl)-aminomethyl-*H*-phosphinic acid* **11**.

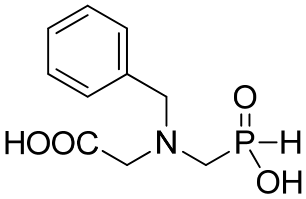

Procedure **A**.

From 165 mg (1.0 mmol) of *N*-benzyl-glycine. Crude product was dissolved in EtOH and precipitated with addition of acetone. The product was filtered off, washed twice with Et_2O and dried on air. White powder (138 mg, 57 %).

A single crystal was prepared by a slow diffusion of acetone vapours into aqueous solution of **11**.

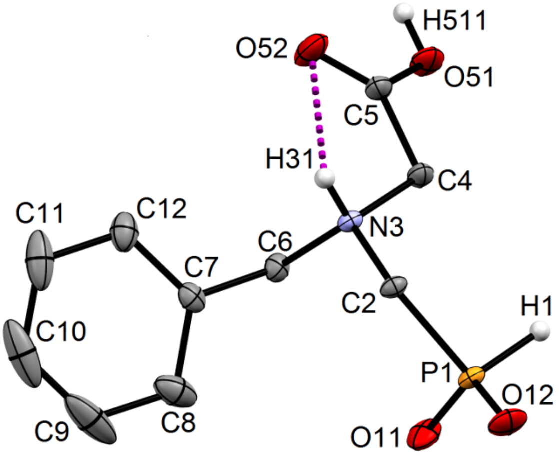

^{1}H NMR (D_2O + *t*BuOH, pD = 1.5 + 0.4): 3.38 (P–C$\underline{H}_2$–N, dd, $^{2}J_{HP}$ 10.5, $^{3}J_{HH}$ 1.5, 2H), 4.17 (HOOC–C$\underline{H}_2$–N, s, 2H), 4.62 (N–C$\underline{H}_2$–Ph, s, 2H), 7.18 ($\underline{H}$–P, dt, $^{1}J_{HP}$ 553.7, $^{3}J_{HH}$ 1.7, 1H)
^{13}C{^{1}H} NMR (D_2O + *t*BuOH, pD = 1.5 + 0.4): 53.5 (P–$\underline{C}H_2$–N, d, $^{1}J_{CP}$ 83.0), 55.5 (HOOC–$\underline{C}H_2$–N, d, $^{3}J_{CP}$ 4.0), 61.3 (Ph–$\underline{C}H_2$–N, d, $^{3}J_{CP}$ 3.8), 128.9 (*i*-Ph), 130.0 (*m*-Ph), 131.2 (*p*-Ph), 132.2 (*o*-Ph), 169.2 (HOO$\underline{C}$–CH_2)
^{31}P NMR (D_2O + *t*BuOH / 85% aq H_3PO_4, pD = 1.5 + 0.4): 9.9 (dt, $^{1}J_{PH}$ 553.9, $^{2}J_{PH}$ 10.5)
MS(+): 266 (266, $[M+Na]^{+}$)
MS(–): 242 (242, $[M–H]^{-}$), 485 (485, $[2M–H]^{-}$)
HRMS(+) (found (*calc*)): 244.0708 (*244.0739*, $C_{10}H_{15}NO_4P$), 487.1352 (*487.1399*, $C_{20}H_{29}N_2O_8P_2$)
TLC (conc. aq. NH_3 : EtOH = 1:{*x*}): 0.57 {5}, 0.29 {10}, 0.29 {20}, 0.21 {35}
EA(found (*calc* M)): C 49.33 (*49.39*), H 5.61 (*5.61*), N 5.65 (*5.76*), P 12.38 (*12.74*)

*[*N,N-*Bis(carboxymethyl)]-aminomethyl-*H-*phosphinic acid* ***12***.

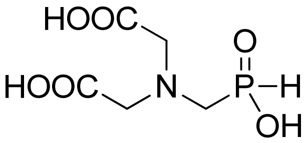

In 4-ml vial, imino-diacetic acid (133 mg, 1.0 mmol, 1 equiv.), paraformaldehyde (60 mg, 2.0 mmol, 2 equiv.) and H_3PO_2 (as 50% aq. solution, 145 mg, 1.1 mmol, 1.1 equiv.) were mixed with glacial AcOH (2 ml). The suspension was heated at 40 °C for 1 day during (product precipitated during the reaction time). Solids were filtered off, washed with AcOH (~2 ml), thrice with Et_2O (~5 ml) and dried on air. White powder of **12**·0.25H_2O (192 mg, 89 %).[7]

A single crystal was obtained by slow acetone vapour diffusion into aqueous solution of **12**.

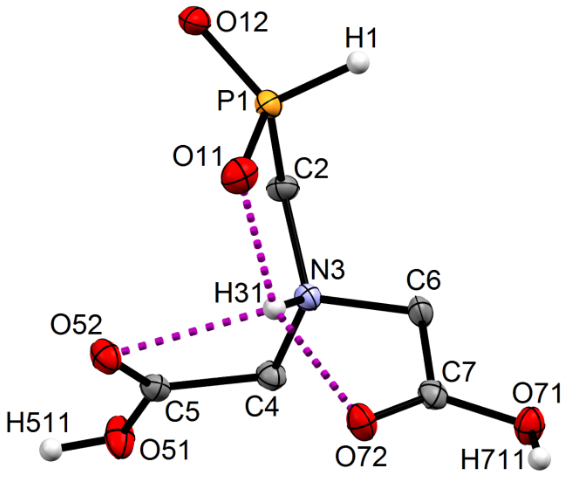

Characterization data were the same as published.[8]

---

[7]**EA (found (*calc* 12·0.25H$_2$O)):** C 27.89 (*27.85*), H 4.42 (*4.91*), N 6.54 (*6.50*), P 14.54 (*14.37*)

[8]M. Paurová, T. David, I. Císařová, P. Lubal, P. Hermann and J. Kotek, *New J. Chem.* **2018**, *42*, 11908–11929.

*[1-(Methyl-*H*-phosphinic acid]-*L*-proline-**13**.*

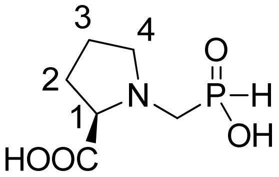

Procedure **A**.

From 115 mg (1.0 mmol) of *L*-proline. Crude product was dissolved in MeOH:EtOH ~9:1 and precipitated with addition of acetone. Product was filtered off, washed twice with Et_2O and dried on air. White powder (141 mg, 73 %). To get single crystals, aqueous solution of **13** was overlayered with acetone and left to stand for several days.

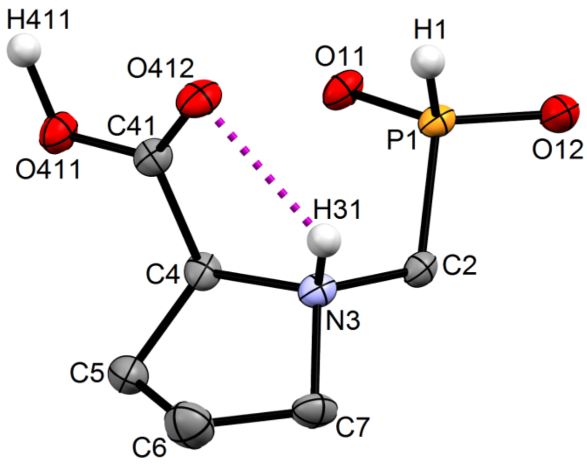

^{1}H NMR (D_2O + *t*BuOH, pD = 1.1 + 0.4): 1.97–2.14 (**3**, m, 1H), 2.15–2.29 (**2** + **3**, m, 2H), 2.52–2.65 (**3**, m, 1H), 3.30–3.39 (**4**, m, 1H), 3.32–3.54 (P–C<u>H</u>$_2$–N, m, 2H), 3.92–4.05 (**4**, m, 1H), 4.35–4.47 (**1**, m, 1H), 7.22 (<u>H</u>–P, dt, $^{1}J_{HP}$ 548.1, $^{3}J_{HH}$ 1.7, 1H)

^{13}C{^{1}H} NMR (D_2O + *t*BuOH, pD = 1.1 + 0.4): 23.3 (**3**), 28.6 (**2**), 54.9 (P–<u>C</u>H$_2$–N, d, $^{1}J_{CP}$ 84.4), 57.8 (**4**, d, $^{3}J_{CP}$ 3.7), 69.9 (**1**, d, $^{3}J_{CP}$ 4.6), 172.2 (HOO<u>C</u>–CH)

^{31}P NMR (D_2O + *t*BuOH / 85% aq H_3PO_4, pD = 1.1 + 0.4): 10.7 (dt, $^{1}J_{PH}$ 549.2, $^{2}J_{PH}$ 10.8)

MS(+): 194 (194, $[M+H]^+$), 387 (387, $[2M+H]^+$)

MS(–): 192 (192, $[M-H]^-$), 385 (385, $[2M-H]^-$)

HRMS(+) (found (*calc*)): 194.0558 (*194.0582*, $C_6H_{13}NO_4P$), 387.1092 (*387.1086*, $C_{12}H_{25}N_2O_8P_2$)

TLC (conc. aq. NH_3 : EtOH = 1:{*x*}): 0.41 {5}, 0.21 {10}, 0.18 {20}, 0.12 {35}

EA(found (*calc* M)): C 36.82 (*37.13*), H 5.99 (*6.26*), N 7.09 (*7.25*), P 15.39 (*16.04*)

*(*N*-methyl)-[*N*-(2-hydroxyethyl)]-aminomethyl-*H*-phosphinic acid* ***14a****.*

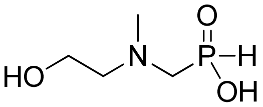

In 4-ml vial, (*N*-methyl)-ethanolamine (80 μl, 75 mg, 1.0 mmol, 1 equiv.), paraformaldehyde (60 mg, 2.0 mmol, 2 equiv.) and H_3PO_2 (as 50% aq. solution, 145 mg, 1.1 mmol, 1.1 equiv.) were mixed with glacial AcOH (2 ml). The suspension was heated at 40 °C for 1 day. Then, solvents were removed by rotary evaporator and an oily residue was purified on strong cation exchanger (Dowex 50, 3×10-cm bed). Product was eluted off with water after a delay. Fractions with pure product were combined and solvents were evaporated *in vacuo* to give product as viscous oil (51 mg, 33 %).

^{1}H NMR (D_2O + *t*BuOH, pD = 3.5 + 0.4): 3.08 (C<u>H</u>$_3$–N, s, 3H), 3.25–3.62 (P–C<u>H</u>$_2$–N + HO–CH$_2$–C<u>H</u>$_2$–N, m, 4H), 3.95 (HO–C<u>H</u>2–CH2–N, t, $^3J_{HH}$ 5.2, 2H), 7.27 (<u>H</u>–P, dt, $^1J_{HP}$ 548.9, $^3J_{HP}$ 1.7, 1H)
^{13}C{^{1}H} NMR (D_2O + *t*BuOH, pD = 3.5 + 0.4): 43.1 (<u>C</u>H$_3$–N, d, $^3J_{CP}$ 4.2), 55.8 (P–<u>C</u>H$_2$–N, d, $^1J_{CP}$ 84.1), 55.8 (HO–<u>C</u>H$_2$–CH$_2$–N), 59.8 (HO–CH$_2$–<u>C</u>H$_2$–N, d, $^3J_{CP}$ 4.4)
^{31}P NMR (D_2O + *t*BuOH / 85% aq H_3PO_4, pD = 3.5 + 0.4): 9.7 (dt, $^1J_{PH}$ 549.2, $^2J_{PH}$ 10.4)
TLC (conc. aq. NH_3 : EtOH = 1:{*x*}): 0.58 {5}, 0.44 {10}, 0.38 {20}, 0.33 {35}

*Bis{(*N*-methyl)-[*N*-(2-hydroxyethyl)]-aminomethyl}phosphinic acid* ***14b****.*

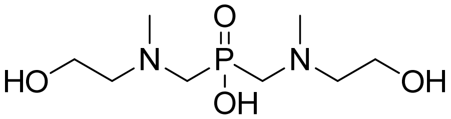

In 4-ml vial, (*N*-methyl)-ethanolamine (80 μl, 75 mg, 1.0 mmol, 1 equiv.), paraformaldehyde (30 mg, 1.0 mmol, 1 equiv.) and H_3PO_2 (as 50% aq. solution, 145 mg, 1.1 mmol, 1.1 equiv.) were mixed with glacial AcOH (2 ml). The suspension was heated at 40 °C for 1 day. Then, solvents were removed by rotary evaporator and an oily residue was purified on strong cation exchanger (Dowex 50, 3×10-cm bed). The column was washed with water. Product was eluted off with 10% aq. pyridine and the solution was concentrated *in vacuo*. An oily residue was dissolved in 1:1 aq. HCl and the solution was stirred at 90 °C for 1 day. Solvents were removed on rotary evaporator and an oily residue was purified on strong cation exchanger (Dowex 50, 3×10-cm bed). The column was washed with water and the product was eluted off with 10% aq. pyridine, and the solvents were evaporated *in vacuo* to give product as a viscous oil (72 mg, 30 %, yield based on starting amine).

^{1}H NMR (D_2O + *t*BuOH, pD = 5.6 + 0.4): 3.07 (C<u>H</u>$_3$–N, s, 6H), 3.42–3.48 (CH$_2$–C<u>H</u>$_2$–N, m, 4H), 3.49 (P–C<u>H</u>$_2$–N, d, $^2J_{HP}$ 9.2, 4H), 3.95 (HO–C<u>H</u>$_2$–CH$_2$–N, t, $^3J_{HH}$ 5.2, 4H)
^{13}C{^{1}H} NMR (D_2O + *t*BuOH, pD = 5.6 + 0.4): 43.7 (<u>C</u>H$_3$–N, d, $^3J_{CP}$ 3.8), 55.6 (P–<u>C</u>H$_2$–N, d, $^1J_{CP}$ 95.0), 56.1 (HO–<u>C</u>H$_2$–CH$_2$), 60.2 (CH$_2$–<u>C</u>H$_2$–N, d, $^3J_{CP}$ 4.6)
^{31}P NMR (D_2O + *t*BuOH / 85% aq H_3PO_4, pD = 5.6 + 0.4): 16.7 (p, $^2J_{PH}$ 10.3)
MS(+): 241 (241, $[M+H]^+$), 481 (481, $[2M+H]^+$)
MS(–): 239 (239, $[M-H]^-$), 479 (479, $[2M-H]^-$)
HRMS(+) (found (*calc*)): 241.1316 (*241.1312*, $C_8H_{22}N_2O_4P$)
TLC (conc. aq. NH_3 : EtOH = 1:{*x*}): 0.50 {5}, 0.32 {10}, 0.30 {20}, 0.20 {35}

*Bis{[*N,N-*bis(2-hydroxyethyl)]-aminomethyl}phosphinic acid* ***15b***.

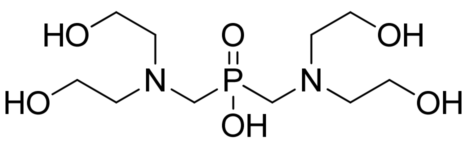

In 4-ml vial, diethanolamine (96 µl, 105 mg, 1.0 mmol, 1 equiv.), paraformaldehyde (60 mg, 2.0 mmol, 2 equiv.) and H_3PO_2 (as 50% aq. solution, 145 mg, 1.1 mmol, 1.1 equiv.) were mixed with glacial AcOH (2 ml). The suspension was heated at 40 °C for 1 day. Then, solvents were removed on rotary evaporator and an oily residue was purified on strong cation exchanger (Dowex 50, 3×10-cm bed). The column was washed with water. The product was eluted off with 10% aq. pyridine and the solution was concentrated *in vacuo*. An oily residue was dissolved in 1:1 aq. HCl and stirred at 90 °C for 1 day. Solvents were removed on rotary evaporator and an oily residue was purified on strong cation exchanger (Dowex 50, 3×10-cm bed). The column was washed with water and the product was eluted off with 10% aq. pyridine and the solution was concentrated *in vacuo* to give the product as a viscous oil (135 mg, 45 %, yield based on amine).

^{1}H NMR (D_2O + *t*BuOH, pD = 3.4 + 0.4): 3.60–3.65 (CH_2–C<u>H</u>$_2$–N, m, 8H), 3.65 (P–C<u>H</u>$_2$–N, d, $^2J_{HP}$ 8.9, 4H), 3.97–4.02 (HO–C<u>H</u>$_2$–CH_2, m, 8H)
^{13}C{^{1}H} NMR (D_2O + *t*BuOH, pD = 3.4 + 0.4): 53.2 (P–<u>C</u>H$_2$–N, d, $^1J_{CP}$ 94.3), 55.9 (HO–<u>C</u>H$_2$–CH_2), 58.0 (CH_2–<u>C</u>H$_2$–N, d, $^3J_{CP}$ 3.2)
^{31}P NMR (D_2O + *t*BuOH / 85% aq H_3PO_4, pD = 3.4 + 0.4): 16.5 (p, $^2J_{PH}$ 6.7)
MS(+): 301 (301, $[M+H]^+$), 601 (601, $[2M+H]^+$)
MS(–): 299 (299, $[M–H]^–$), 599 (599, $[2M–H]^–$)
HRMS(+) (found (*calc*)): 323.1292 (*323.1348*, $C_{10}H_{25}N_2O_6PNa$), 623.2683 (*623.2798*, $C_{20}H_{49}N_4O_{12}P_2Na$)
TLC (conc. aq. NH_3 : EtOH = 1:{*x*}): 0.45 {5}, 0.32 {10}, 0.26 {20}, 0.23 {35}

*Piperazine-(*N-*methyl)-(*N′-*methyl-*H-*phosphinic acid)* ***16***.

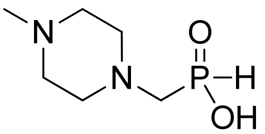

<u>Procedure **B**</u>.
From 111 µl (100 mg, 1.0 mmol) of (*N*-methyl)-piperazine. Viscous oil (36 mg, 20 %).

^{1}H NMR (D_2O + *t*BuOH, pD = 4.9 + 0.4): 2.72 (P–C<u>H</u>$_2$–N, dt, $^2J_{HP}$ 10.8, $^3J_{HH}$ 2.1, 2H), 2.90 (C<u>H</u>$_3$–N, s, 3H), 2.95–3.63 (N–C<u>H</u>$_2$–C<u>H</u>$_2$–N, m, 8H), 7.07 (<u>H</u>–P, dt, $^1J_{HP}$ 519.0, $^3J_{HH}$ 2.1, 1H)
^{13}C{^{1}H} NMR (D_2O + *t*BuOH, pD = 4.9 + 0.4): 43.4 (<u>C</u>H$_3$–N), 51.9 (CH_2–<u>C</u>H$_2$–N–CH_2, d, $^3J_{CP}$ 8.6), 53.5 (CH_3–N–<u>C</u>H$_2$), 58.1 (P–<u>C</u>H$_2$–N, d, $^1J_{CP}$ 101.3)
^{31}P NMR (D_2O + *t*BuOH / 85% aq H_3PO_4, pD = 4.9 + 0.4): 20.2 (dt, $^1J_{PH}$ 518.4, $^2J_{PH}$ 10.8)
MS(+): 179 (179, $[M+H]^+$), 357 (357, $[2M+H]^+$)
MS(–): 177 (177, $[M–H]^–$), 355 (355, $[355–H]^–$)
HRMS(+) (found (*calc*)): 179.0915 (*179.0949*, $C_6H_{16}N_2O_2P$), 357.1772 (*357.1821*, $C_{12}H_{31}N_4O_4P_2$)
TLC (conc. aq. NH_3 : EtOH = 1:{*x*}): 0.64 {5}, 0.36 {10}, 0.21 {20}, 0.18 {35}

[N,N-Bis(2-phthalimido-ethyl)]-aminomethyl-H-phosphinic acid **17**.

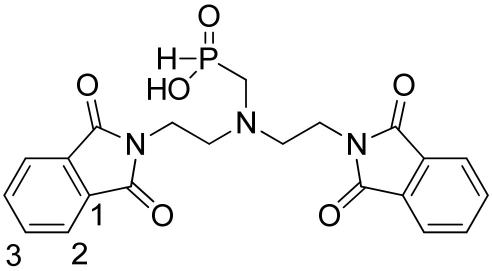

<u>Procedure **B**</u>.

From 115 mg (1.0 mmol) of *N*,*N*-bis(2-phtalimido-ethyl)amine. Product was crystallized from boiling water, filtered off, washed twice with Et_2O and dried on air. White powder (278 mg, 63 %).

A single crystal was prepared by slow cooling of a hot aqueous solution of **17**.

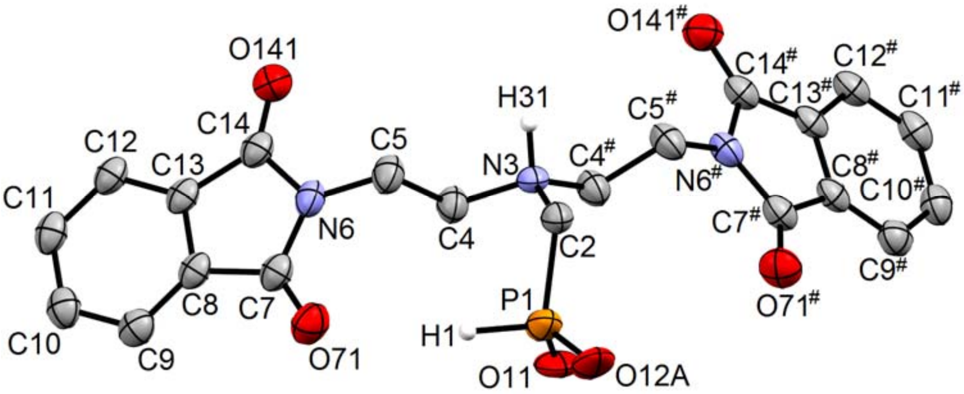

^{1}H NMR (DMSO-d_6): 2.86 (H–P–C<u>H</u>$_2$–N, dd, $^2J_{HP}$ 9.2, $^3J_{HH}$ 2.2, 2H), 2.91 (PhtN–CH$_2$–C<u>H</u>$_2$–N, t, $^3J_{HH}$ 6.2, 4H), 3.62 (PhtN–C<u>H</u>$_2$–CH$_2$–N, t, $^3J_{HH}$ 6.2, 4H), 6.75 (<u>H</u>–P, dt, $^1J_{HP}$ 529.6, $^3J_{HH}$ 2.2, 1H), 7.68–7.81 (Phth, m, 8H)

^{13}C{^{1}H} NMR (DMSO-d_6): 35.1 (PhtN–<u>C</u>H$_2$–CH$_2$–N), 52.4 (H–P–<u>C</u>H$_2$–N, d, $^1J_{CP}$ 105.6), 52.7 (PhtN–CH$_2$–<u>C</u>H$_2$–N, d, $^3J_{CP}$ 6.6), 122.9 (**2**), 131.7 (**1**), 134.2 (**3**), 167.8 (N–<u>C</u>=O)

^{31}P NMR (DMSO-d_6 / 85% aq H_3PO_4): 26.8 (dt, $^1J_{PH}$ 529.8, $^2J_{PH}$ 9.6)

MS(+): 464 (464, [M+Na]$^+$)

MS(–): 440 (440, [M–H]$^-$)

HRMS(+) (found (*calc*)): 442.1173 (*442.1162*, $C_{21}H_{21}N_3O_6P$)

TLC (conc. aq. NH$_3$: EtOH = 1:{*x*}): 0.84 {1.5}, 0.39 {5}, 0.13 {10}, 0.09 {20}

EA(found (*calc* M · 3/2H$_2$O)): C 53.80 (*53.85*), H 4.85 (*4.95*), N 8.77 (*8.97*), P 6.38 (*6.61*)

General procedure for reaction of various aldehydes (Table 2) in the paper text

*1-[(*N,N*-Dibenzyl)-amino]-ethyl-*H*-phosphinic acid* ***18***.

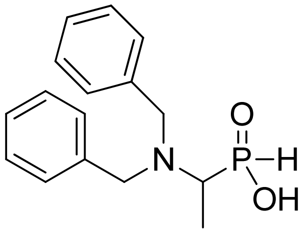

Either from 112 µl (2.0 mmol) of acetaldehyde, or 92 µl (0.7 mmol) of paraldehyde. Viscous oil (202 mg, ~70 %).
A single crystal was prepared by mixing of 1-adamantylamine (~1–2 equiv.) with a hot aqueous solution of **18**. After slow cooling, the product crystallized as 1-adamantylammonium salt. Anion of **18** is shown below.

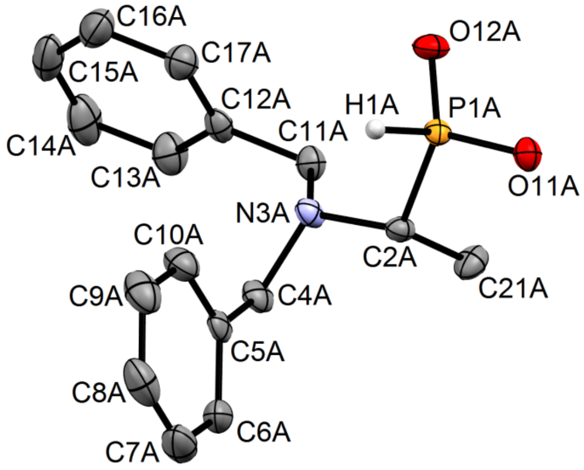

^{1}H NMR (D_2O + *t*BuOH, pD = 4.1 + 0.4): 1.59 (C<u>H</u>$_3$–CH, dd, $^{3}J_{HP}$ 15.9, $^{3}J_{HH}$ 7.3, 3H), 3.36 (P–C<u>H</u>–N, dqd, $^{2}J_{HP}$ 12.9, $^{3}J_{HH}$ 7.3, $^{3}J_{HH}$ 1.5, 1H), 4.24–4.83 (Ph–C<u>H</u>$_2$–N, m, 4H), 7.06 (<u>H</u>–P, dd, $^{1}J_{HP}$ 544.3, $^{3}J_{HH}$ 1.5, 1H), 7.41–7.56 (Ph, m, 10H)
^{13}C{^{1}H} NMR (D_2O + *t*BuOH, pD = 4.1 + 0.4): 8.2 (<u>C</u>H$_3$–CH), 56.7 (P–<u>C</u>H–N, d, $^{1}J_{CP}$ 86.8), 129.8 (*i*-Ph), 130.1 (*m*-Ph), 130.9 (*p*-Ph), 131.8 (*o*-Ph)
^{31}P NMR (D_2O + *t*BuOH / 85% aq H_3PO_4, pD = 4.1 + 0.4): 18.7 (dqd, $^{1}J_{PH}$ 544.3, $^{3}J_{PH}$ 15.6, $^{2}J_{PH}$ 13.2)
MS(+): 312 (312, [M+Na]$^+$), 579 (579, [2M+H]$^+$), 601 (601, [2M+Na]$^+$)
MS(–): 288 (288, [M–H]$^-$), 577 (577, [2M–H]$^-$)
HRMS(+) (found (*calc*)): 290.1268 (*290.1310*, $C_{16}H_{20}NO_2P$)
TLC: 0.63 (*i*PrOH:conc. aq. NH_3:H_2O = 10:1:2), 0.62 (MeOH:*i*PrOH = 1:1), 0.54 (EtOH)

*{1-[(*N,N*-Dibenzyl)-amino]-but-1-yl}*-H-*phosphinic acid* ***19***.

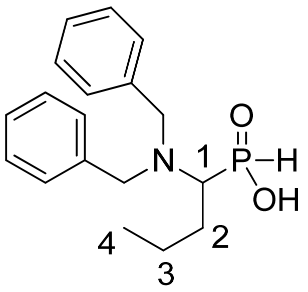

From 180 µl (2.0 mmol) of *n*-butyraldehyde. Viscous oil (133 mg, 42 %).

^{1}H NMR (CD_3OD): 1.01 (**4**, t, $^{3}J_{HH}$ 7.4, 3H), 1.29–1.46 (**3**, m, 1H), 1.62–1.75 (**3**, m, 1H), 1.92–2.14 (**2**, m, 2H), 2.93–3.02 (**1**, m, 1H), 4.30–4.70 (Ph–C$\underline{H}_2$–N, m, 4H), 7.15 ($\underline{H}$–P, dd, $^{1}J_{HP}$ 537.0, $^{3}J_{HH}$ 1.2, 1H), 7.37–7.65 (Ph, m, 10H)
^{13}C{^{1}H} NMR (CD_3OD): 14.4 (**4**), 21.1 (**3**, d, $^{3}J_{CP}$ 2.4), 26.5 (**2**, d, $^{2}J_{CP}$ 1.5), 57.3 (Ph–$\underline{C}H_2$–N), 62.0 (P–$\underline{C}H_2$–N, d, $^{1}J_{CP}$ 83.3), 130.7 (Ph), 131.2 (*p*-Ph), 131.9 (Ph), 132.0 (*i*-Ph)
^{31}P NMR (CD_3OD / 85% aq H_3PO_4): 14.3–15.1 and 18.8–19.5 (dm, $^{1}J_{PH}$ 536.9)
MS(+): 318 (318, $[M+H]^+$), 340 (340, $[M+Na]^+$), 356 (356, $[M+K]^+$), 657 (657, $[2M+Na]^+$)
MS(–): 316 (316, $[M–H]^-$), 633 (633, $[2M–H]^-$)
HRMS(+) (found (*calc*)): 318.1608 (*318.1623*, $C_{18}H_{25}NO_2P$), 635.3137 (*635.3168*, $C_{36}H_{49}N_2O_4P_2$)
TLC: 0.65 (*i*PrOH:conc. aq. NH_3:H_2O = 10:1:2), 0.65 (MeOH:*i*PrOH = 1:1), 0.54 (EtOH)

{1-[(N,N-Dibenzyl)-amino]-(2-phenyl)-eth-1-yl}-H-phosphinic acid ***20***.

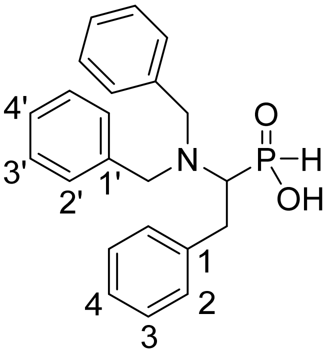

From 222 μl (240 mg, 2.0 mmol) of freshly distilled 1-phenyl-acetaldehyde. A viscous oil of a crude product was dissolved in CH_2Cl_2 (10 ml) and the solution was washed twice with water (5 ml). The organic phase was dried with anhydrous $MgSO_4$ and concentrated *in vacuo*. An oily residue was dissolved in MeOH (5 ml) and the product crystallized in the fridge. Product was filtered off, washed with Et_2O (2×1 ml) and dried on air. Crystalline powder, **20**·MeOH·3/2H_2O (68 mg, 16 %).

A single crystal was prepared by slow cooling of hot MeOH solution of **20** in fridge.

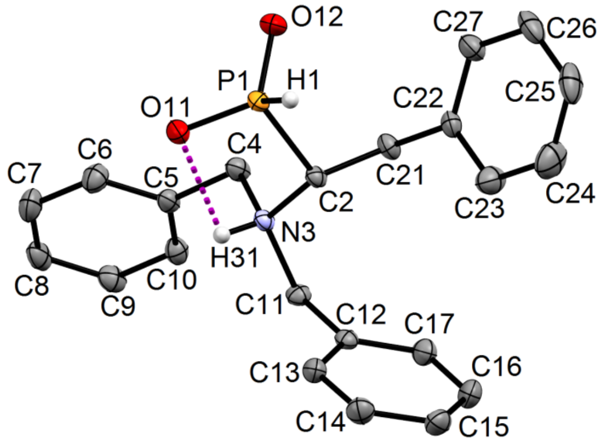

^{1}H NMR ($(CD_3)_2SO$): 2.45–2.70 (CH–C<u>H</u>$_2$–Ph + P–<u>C</u>H–N, m, 3H), 2.72 (<u>Me</u>OH, s, 3H), 4.17–4.55 (Ph–C<u>H</u>$_2$–N, m, 4H), 6.60 (<u>H</u>–P, d, $^1J_{HP}$ 522.8, 1H), 6.69–6.90 (Ph, m, 10H)
^{13}C{^{1}H} NMR ($(CD_3)_2SO$): 29.9 (CH–C<u>H</u>$_2$–Ph, d, $^2J_{CP}$ 8.1), 48.7 (<u>Me</u>OH), 54.1 (N–<u>C</u>H$_2$–Ph, d, $^3J_{CP}$ 5.7), 59.2 (P–<u>C</u>H–N, d, $^1J_{CP}$ 101.8), 126.2 (**4** or **4'**), 127.0 (**3**), 128.2 (**3'**), 128.3 (**4** or **4'**), 128.5 (**2'**), 129.4 (**2**), 139.2 (**1'**), 139.5 (**1**, d, $^3J_{CP}$ 12.6)
^{31}P NMR ($(CD_3)_2SO$ / 85% aq H_3PO_4): 29.1–29.4 and 32.3–32.7 (dm, $^1J_{PH}$ 522.6)
MS(+): 388 (388, $[M+Na]^+$), 404 (404, $[M+K]^+$)
MS(–): 364 (364, $[M–H]^-$)
HRMS(+) (found (*calc*)): 366.1628 (*366.1623*, $C_{22}H_{25}NO_2P$), 763.2892 (*763.3430*, $C_{44}H_{49}N_2O_4P_2$ + CH_3OH)
TLC: 0.74 (*i*PrOH:conc. aq. NH_3:H_2O = 10:1:2), 0.62 (MeOH:*i*PrOH = 1:1), 0.54 (EtOH)
EA(found (*calc* M · MeOH · 3/2H_2O)): C 65.13 (*65.08*), H 6.86 (*7.36*), N 3.26 (*3.30*), P 7.15 (*7.30*)

{1-Hydroxy-2,2,2-trifluoro-eth-1-yl}-H-*phosphinic acid* ***21a***.

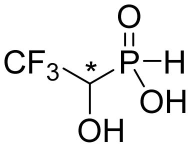

In 4-ml vial, (*N*,*N*-dibenzyl)-amine (192 µl, 1.0 mmol, 1 equiv.), trifluoroacetaldehyde monohydrate (161 µl, 2.0 mmol, 2 equiv.) and H_3PO_2 (50% aq., 145 mg, 1.1 mmol, 1.1 equiv.) were mixed with glacial AcOH (2 ml). Solution was heated at 80 °C for 3 days and conversion was determined by ^{31}P NMR. Then, solvents were removed *in vacuo* and an oily residue was purified on strong cation exchanger chromatography (Dowex 50, 3×10-cm bed) and it was eluted off with water. After concentration *in vacuo*, an oily residue was further purified by silica column chromatography (50 g, V_M ~35 ml). Column was washed with *i*PrOH (~200 ml) to elute off **21a** and **21b** was then eluted with *i*PrOH:conc. aq. NH_3:water ~20:1:2 (~7.5-ml fractions). Fractions containing pure product were combined and the solution was evaporated to dryness. To regenerate free acid form of the compound, the oil was applied on strong cation exchanger (Dowex 50, 3×5-cm bed) and product was eluted off with water. Fractions containing pure product were evaporated to dryness. Viscous oil (43 mg, 24 %).

^{1}H NMR (D_2O + *t*BuOH, pD = 0.5 + 0.4): 4.18 (P–C<u>H</u>–CF_3, dq, $^2J_{HP}$ 10.8, $^3J_{HF}$ 9.0, 1H), 7.01 (<u>H</u>–P, dqd, $^1J_{HP}$ 558.3, $^4J_{HF}$ 2.4, $^2J_{HH}$ 1.3, 1H)

^{13}C{^{1}H} NMR (D_2O + *t*BuOH, pD = 0.5 + 0.4): 69.3 (P–<u>C</u>H–CF_3, dq, $^1J_{CP}$ 102.2, $^2J_{CF}$ 30.5), 124.9 (P–CH–<u>C</u>F_3, qd, $^1J_{CF}$ 281.0, $^3J_{CP}$ 5.0)

^{31}P NMR (D_2O / 85% aq H_3PO_4, pD = 0.5 + 0.4): 19.0 (dq, $^1J_{PH}$ 558.6, $^3J_{PF}$ 6.6)

^{19}F NMR (D_2O / 0.1M TFA in D_2O, pD = 0.5 + 0.4): –71.67 (ddd, $^3J_{FH}$ 9.5, $^3J_{FP}$ 6.8, $^4J_{FH}$ 2.8)

MS(+): 165 (165, $[M+H]^+$), 329 (329, $[2M+H]^+$)

MS(–): 163 (163, $[M–H]^-$), 327 (327, $[2M–H]^-$), 491 (491, $[3M–H]^-$)

HRMS(–) (found (*calc*)): 162.9780 (*162.9777*, $C_2H_3F_3O_3P$)

TLC: 0.26 (*i*PrOH:conc. aq. NH_3:H_2O = 20:1:2), 0.50 (*i*PrOH:conc. aq. NH_3:H_2O = 10:1:2), 0.23 (*i*PrOH)

Bis(1-hydroxy-2,2,2-trifluoro-eth-1-yl)phosphinic acid ***21b***

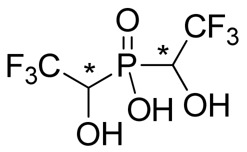

In 4-ml vial, trifluoroacetaldehyde monohydrate (322 μl, 4.0 mmol, 4 equiv.), and H_3PO_2 (50% aq., 132 mg, 1.0 mmol, 1.0 equiv.) were mixed with glacial AcOH (2 ml). Solution was heated at 80 °C for 3 days and conversion was determined by ^{31}P NMR. Then, solvents were removed on rotary evaporator. An oily residue was purified on silica column chromatography (50 g, V_M ~35 ml) with elution of *i*PrOH (7.5-ml fractions). Fractions containing pure product were combined, the solution was evaporated to dryness and once co-evaporated with toluene (~5 ml). Viscous oil (236 mg, 90 %).

Mixture of diastereoisomers:
^{1}H NMR (D_2O + *t*BuOH, pD = 0.3 + 0.4): 4.41 (P–C<u>H</u>–CF_3, p, $^3J_{HF}$ 9.0, 1H), 4.49 (P–C<u>H</u>–CF_3, p, $^3J_{HF}$ ~ $^2J_{HP}$ 9.1, 1H)
^{13}C{^{1}H} NMR (D_2O + *t*BuOH, pD = 0.3 + 0.4): 2× 67.2 (P–<u>C</u>H–CF_3, dq, $^1J_{CP}$ 101.0, $^3J_{CF}$ 30.6), 2× 125.0 (P–CH–<u>C</u>F_3, qd, $^1J_{CF}$ 281.2, $^2J_{CP}$ 4.2)
^{31}P NMR (D_2O / 85% aq H_3PO_4, pD = 0.3 + 0.4): 22.3 (thept, $^2J_{PH}$ 8.9, $^3J_{PF}$ 4.7), 25.0 (thept, $^2J_{PH}$ 9.6, $^3J_{PF}$ 4.9)
^{19}F NMR (D_2O / 0.1M TFA in D_2O, pD = 0.3 + 0.4): –70.51 (dd, $^3J_{FH}$ 9.2, $^4J_{FP}$ 4.8, 1F), –71.26 (dd, $^3J_{FH}$ 9.1, $^4J_{FP}$ 4.6, 1F)
MS(+): 263 (263, $[M+H]^+$), 525 (525, $[2M+H]^+$), 547 (547, $[2M+Na]^+$)
MS(–): 261 (261, $[M–H]^-$), 523 (523, $[2M–H]^-$)
HRMS(–) (found (*calc*)): 260.9757 (*260.9751*, $C_4H_4F_6O_4P$)
TLC: 0.32 (*i*PrOH:conc. aq. NH_3:H_2O = 20:1:2), 0.50 (*i*PrOH:conc. aq. NH_3:H_2O = 10:1:2), 0.55 (*i*PrOH)

General procedure for reaction of primary amines (Table 3) in the paper text

(N-*Benzyl)-amino*-N,N-*bis(methyl*-H-*phosphinic acid)* **22**.

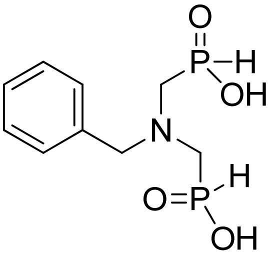

From 54 µl (54 mg, 1.0 mmol) of *N*-benzyl-amine. Viscous oil (45 mg, 34 %) which solidified upon standing.

Characterization data were the same as published.[9]

A single crystal was prepared by slow evaporation of aqueous solution of **22**.

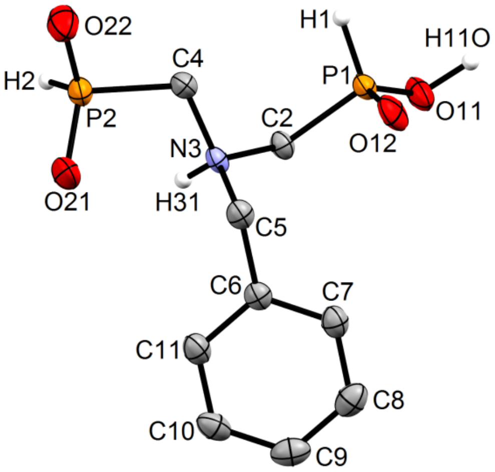

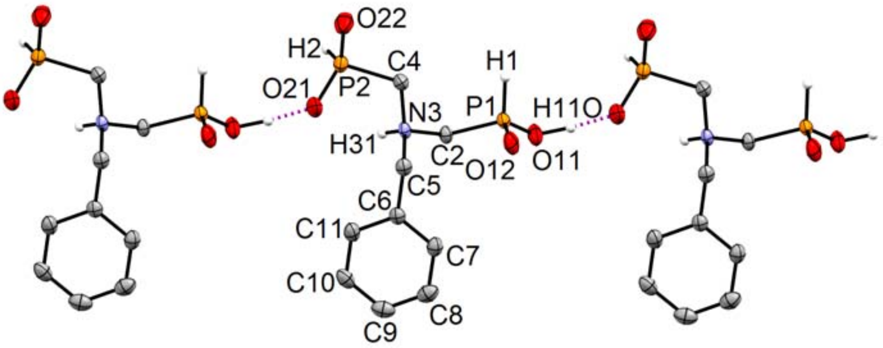

^{1}H NMR (D_2O + *t*BuOH, pD = 0.8 + 0.4): 3.46 (P–C$\underline{H}_2$–N, d, $^{2}J_{HP}$ 10.6, 4H), 4.69 (N–C$\underline{H}_2$–Ph, s, 2H), 7.17 ($\underline{H}$–P, dt, $^{1}J_{HP}$ 555.6, $^{3}J_{HH}$ 1.7, 2H), 7.52–7.64 (Ph, m, 5H)

^{13}C{^{1}H} NMR (D_2O + *t*BuOH, pD = 0.8 + 0.4): 54.1 (P–$\underline{C}H_2$–N, dd, $^{1}J_{CP}$ 83.6, $^{3}J_{CP}$ 4.0), 61.9 (N–C$\underline{H}_2$–Ph, t, $^{3}J_{CP}$ 3.5), 129.2 (*i*-Ph), 130.1 (*m*-Ph), 131.2 (*p*-Ph), 132.3 (*o*-Ph)

^{31}P NMR (D_2O + *t*BuOH / 85% aq H_3PO_4, pD = 0.8 + 0.4): 10.9 (dt, $^{1}J_{HP}$ 555.6, $^{2}J_{PH}$ 10.6)

MS(+): 527 (527, $[2M+H]^+$), 549 (549, $[2M+Na]^+$)

MS(–): 262 (262, $[M–H]^–$), 547 (547, $[2M+Na–2H]^–$)

HRMS(–) (found (*calc*)): 262.0404 (*262.0398*, $C_9H_{14}NO_4P_2$)

TLC (conc. aq. NH_3 : EtOH = 1:{*x*}): 0.83 {5}, 0.69 {10}, 0.64 {20}, 0.57 {35}.

---

[9] B. Dhawam, D. Redmore, *J. Chem. Res. (S)* **1988**, 34–35.

[N-(2-Phenyl-ethyl)]-amino-N,N-bis(methyl-H-phosphinic acid) ***23***.

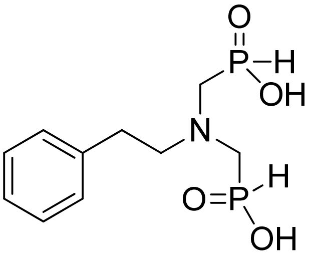

From 63 µl (31 mg, 1.0 mmol) of (2-phenyl-ethyl)amine. Viscous oil (44 mg, 32 %).

^{1}H NMR (D_2O + *t*BuOH, pD = 0.7 + 0.4): 3.12–3.19 ($N–CH_2–C\underline{H}_2–Ph$, m, 2H), 3.55 ($P–C\underline{H}_2–N$, d, $^2J_{HP}$ 10.5, 4H), 3.72–3.79 ($N–C\underline{H}_2–CH_2–Ph$, m, 2H), 7.29 ($\underline{H}–P$, dt, $^1J_{HP}$ 553.4, $^3J_{HH}$ 1.6, 2H), 7.33–7.46 (Ph, m, 5H)
^{13}C{^{1}H} NMR (D_2O + *t*BuOH, pD = 0.7 + 0.4): 30.4 ($N–CH_2–\underline{C}H_2–Ph$), 54.7 ($P–\underline{C}H_2–N$, d, $^1J_{CP}$ 84.2), 58.9 ($N–\underline{C}H_2–CH_2–Ph$, t, $^3J_{CP}$ 3.6), 128.2 (*p*-Ph), 129.6 (*o*-Ph), 129.8 (*m*-Ph), 136.4 (*i*-Ph)
^{31}P NMR (D_2O + *t*BuOH / 85% aq H_3PO_4, pD = 0.7 + 0.4): 10.5 (dt, $^1J_{HP}$ 553.3, $^2J_{PH}$ 10.5)
MS(+): 278 (278, $[M+H]^+$), 555 (555, $[2M+H]^+$)
MS(–): 276 (276, $[M–H]^–$), 553 (553, $[2M–H]^–$)
HRMS(–) (found (*calc*)): 276.0560 (*276.0555*, $C_{10}H_{16}NO_4P_2$)
TLC (conc. aq. NH_3 : EtOH = 1:{*x*}): 0.78 {5}, 0.68 {10}, 0.64 {20}, 0.53 {35}

(N-*Cyclohexyl)-amino*-N,N-*bis(methyl*-H-*phosphinic acid)* ***24***.

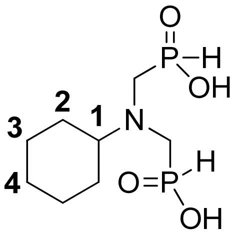

From 57 µl (50 mg, 1.0 mmol) of cyclohexylamine. Viscous oil (43 mg, 33 %).
^{1}H NMR (D_2O + *t*BuOH, pD = 0.8 + 0.4): 1.14–1.24 (**4**, m, 1H), 1.30–1.44 (**3**, m, 2H), 1.45–1.59 (**2**, m, 2H), 1.64–1.74 (**4**, m, 1H), 1.88–1.98 (**3**, m, 2H), 2.03–2.14 (**2**, m, 2H), 3.45 (P–C<u>H</u>$_2$–N, d, $^2J_{HP}$ 10.9, 4H), 3.61–3.71 (**1**, m, 1H), 7.28 (<u>H</u>–P, d, $^1J_{HP}$ 555.8, 2H)
^{13}C{^{1}H} NMR (D_2O + *t*BuOH, pD = 0.8 + 0.4): 2× 25.1 (**4** + **3**), 26.9 (**2**), 51.9 (P–<u>C</u>H$_2$–N, dd, $^1J_{CP}$ 83.9, $^3J_{CP}$ 3.9), 68.0 (**1**, t, $^3J_{CP}$ 3.7)
^{31}P NMR (D_2O + *t*BuOH / 85% aq H_3PO_4, pD = 0.8 + 0.4): 11.8 (dt, $^1J_{HP}$ 555.8, $^2J_{PH}$ 10.8)
MS(+): 256 (256, $[M+H]^+$), 511 (511, $[2M+H]^+$)
MS(–): 254 (254, $[M-H]^-$), 509 (509, $[2M-H]^-$)
HRMS(–) (found (*calc*)): 254.0717 (*254.0711*, $C_8H_{18}NO_4P_2$)
TLC (conc. aq. NH_3 : EtOH = 1:{*x*}): 0.57 {5}, 0.52 {10}, 0.48 {20}, 0.43 {35}

(N-*t-Butyl)-amino*-N,N-*bis(methyl*-H-*phosphinic acid)* ***25***.

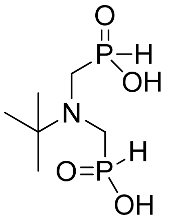

From 52 µl (36 mg, 1.0 mmol) of *t*-butylamine. Viscous oil which solidified upon standing, **25**·H_2O (25 mg, 20 %).

A single crystal was obtained on standing the oil of **25** for several weeks.

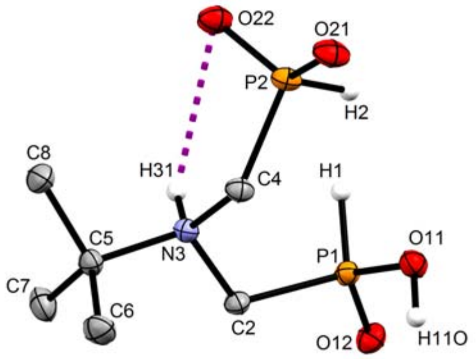

^{1}H NMR (D_2O + *t*BuOH, pD = 0.6 + 0.4): 1.48 (C<u>H</u>$_3$–C–P, s, 9H), 3.48 (P–C<u>H</u>$_2$–N, dd, $^2J_{HP}$ 11.2, $^3J_{HH}$ 1.5, 4H), 7.30 (<u>H</u>–P, dt, $^1J_{HP}$ 559.1, $^3J_{HH}$ 1.5, 2H)
^{13}C{^{1}H} NMR (D_2O + *t*BuOH, pD = 0.6 + 0.4): 24.7 (<u>C</u>H$_3$–C–P), 52.3 (P–<u>C</u>H$_2$–N, dd, $^1J_{CP}$ 82.3, $^3J_{CP}$ 2.6), 69.0 (CH$_3$–<u>C</u>–P, t, $^3J_{CP}$ 3.3)
^{31}P NMR (D_2O + *t*BuOH / 85% aq H_3PO_4, pD = 0.6 + 0.4): 13.0 (dt, $^1J_{HP}$ 559.2, $^2J_{PH}$ 11.2)
MS(+): 230 (230, $[M+H]^+$), 252 (252, $[M+Na]^+$), 459 (459, $[2M+H]^+$), 481 (481, $[2M+Na]^+$)
MS(–): 228 (228, $[M-H]^-$), 457 (457, $[2M-H]^-$)
HRMS(–) (found (*calc*)): 228.0566 (*228.0560*, $C_6H_{16}NO_4P_2$)
TLC (conc. aq. NH_3 : EtOH = 1:{*x*}): 0.59 {5}, 0.54 {10}, 0.39 {20}, 0.28 {35}
EA(found (*calc* M · H_2O)): C 29.10 (*29.16*), H 6.90 (*7.75*), N 5.39 (*5.67*), P 25.88 (*25.06*)

(N-*Adamantyl)-amino*-N,N-*bis(methyl*-H-*phosphinic acid)* ***26***.

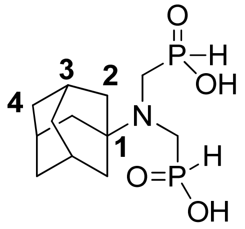

From 75 mg (1.0 mmol) of 1-adamantylamine. Viscous oil (47 mg, 31 %).

^{1}H NMR (D_2O + *t*BuOH, pD = 0.8 + 0.4): 1.60–1.82 (**4**, m, $^{3}J_{HH}$ 12.7, 6H), 1.93–2.11 (**2**, m, 6H), 2.24–2.37 (**3**, m, 3H), 3.52 (P–C$\underline{H}_2$–N, d, $^{2}J_{HP}$ 10.4, 4H), 7.31 ($\underline{H}$–P, dt, $^{1}J_{HP}$ 560.0, $^{3}J_{HH}$ 1.5, 2H)
^{13}C{^{1}H} NMR (D_2O + *t*BuOH, pD = 0.8 + 0.4): 30.3 (**4**), 35.2 (**3**), 37.0 (**2**), 50.1 (P–$\underline{C}H_2$–N, dd, $^{1}J_{CP}$ 83.6, $^{3}J_{CP}$ 3.6), 69.9 (**1**, t, $^{3}J_{CP}$ 2.9)
^{31}P NMR (D_2O + *t*BuOH / 85% aq H_3PO_4, pD = 0.8 + 0.4): 13.4 (dt, $^{1}J_{HP}$ 560.5, $^{2}J_{PH}$ 11.0)
MS(+): 308 (308, $[M+H]^+$), 615 (615, $[2M+H]^+$)
MS(–): 306 (306, $[M–H]^–$), 613 (613, $[2M–H]^–$)
HRMS(+) (found (*calc*)): 306.1028 (*306.1030*, $C_{12}H_{22}NO_4P_2$)
TLC (conc. aq. NH_3 : EtOH = 1:{*x*}): 0.73 {5}, 0.64 {10}, 0.54 {20}, 0.43 {35}

*Amino-(*N*-methylphosphonic acid)-*N,N*-bis(methyl-*H*-phosphinic acid)* ***27***.

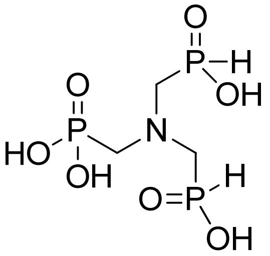

In 25-ml flask, aminomethylphosphonic acid (111 mg, 1.0 mmol, 1 equiv), paraformaldehyde (33 mg, 1.1 mmol, 1.1 equiv.), 50% aq. H_3PO_2 (396 mg, 3.0 mmol, 3 equiv.) and anhydrous sodium acetate (164 mg, 2.0 mmol, 2 equiv.) were mixed with glacial AcOH (10 ml). Solution was stirred at room temperature for 2 days and conversion was determined by ^{31}P NMR. Then, the solids were filtered off and the filtrate was concentrated *in vacuo*. An oily residue was triturated in MeOH (10 ml) using ultrasound. The solids were filtered off and washed with Et_2O (2× 2 ml). A crude powdered product was dissolved in water (5 ml) and was purified on strong cation exchanger (Dowex 50, 3×10-cm bed). Product was eluted off with water. After concentrating *in vacuo*, an oily residue was re-purified on strong cation exchanger (Dowex 50, 3×10-cm bed) and 1–3-ml fractions were collected. Fractions containing product were combined, solutions was concentrated *in vacuo* and the residue repeatedly purified as stated above (~2–4 cycles). Finally, fractions with pure product were combined and concentrated *in vacuo* to give product as a viscous oil (192 mg, 72 %).

^{1}H NMR (D_2O + *t*BuOH, pD = –0.1 + 0.4): 3.71 (H–P–C<u>H</u>$_2$–N, d, $^2J_{HP}$ 10.5, 4H), 3.78 (HO–P–C<u>H</u>$_2$–N, d, $^2J_{HP}$ 12.6, 2H), 7.32 (<u>H</u>–P, dt, $^1J_{HP}$ 560.9, $^3J_{HH}$ 1.8, 2H)
^{13}C{^{1}H} NMR (D_2O + *t*BuOH, pD = –0.1 + 0.4): 54.0 (HO–P–<u>C</u>H$_2$–N, dt, $^1J_{CP}$ 138.4, $^3J_{CP}$ 3.9), 56.2 (H–P–<u>C</u>H$_2$–N, dp, $^1J_{CP}$ 85.6, $^3J_{CP}$ 3.3)
^{31}P NMR (D_2O + *t*BuOH / 85% aq H_3PO_4, pD = –0.1 + 0.4): 9.0 (HO–<u>P</u>, t, $^2J_{PH}$ 12.6, 1P), 12.0 (H–<u>P</u>, dt, $^1J_{PH}$ 560.9, $^2J_{PH}$ 10.5, 2P)
MS(+): 335 (335, $[M+Na]^+$)
MS(–): 311 (311, $[M-H]^-$), 623 (623, $[2M-H]^-$), 644 (644, $[2M-2H+Na]^-$)
HRMS(–) (found (*calc*)): 265.9757 (*265.9754*, $C_3H_{11}NO_7P_3$)
TLC (conc. aq. NH_3 : MeOH = 1:{*x*}): 0.67 {1}, 0.43 {2}, 0.22 {5}

(N-*benzyl)-(*N-*methylphosphonic acid)-aminomethyl*-H-*phosphinic acid* ***28a***.

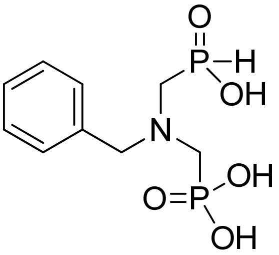

In 25-ml flask, (*N*-benzyl)-aminomethylphosphonic acid (201 mg, 1.0 mmol, 1 equiv.), paraformaldehyde (33 mg, 1.1 mmol, 1.1 equiv.), and H_3PO_2 (as 50% aq. solution, 396 mg, 3.0 mmol, 3 equiv.) were mixed with glacial AcOH (20 ml). The suspension was stirred at room tepmperature for 2 days and conversion was determined by ^{31}P NMR. Then, solvents were removed on rotary evaporator and an oily residue was co-evaporated with toluene (2×5 ml) and once with water (5 ml). Oily residue was dissolved in water (1 ml) and purified by C18 silica column chromatography (product was eluted by pure water after small delay). Fractions containing products were combined and evaporated to dryness. Then, the solidified product was triturated in MeOH (5 ml), filtered off and washed with Et_2O (2×5 ml). White powder, **28a**·0.5H_2O (151 mg, 49 %).

^{1}H NMR (D_2O + *t*BuOH, pD = 0.4 + 0.4): 3.44 (H–P–C<u>H</u>$_2$–N, d, $^2J_{HP}$ 10.5, 2H), 3.52 (HO–P–C<u>H</u>$_2$–N, d, $^3J_{HH}$ 12.6, 2H), 4.72 (Ph–C<u>H</u>$_2$–N, s, 2H), 7.16 (<u>H</u>–P, dt, $^1J_{HP}$ 557.7, $^3J_{HH}$ 1.7, 1H), 7.49–7.66 (Ph, m, 5H)
^{13}C{^{1}H} NMR (D_2O + *t*BuOH, pD = 0.4 + 0.4): 51.7 (HO–P–<u>C</u>H$_2$–N, dd, $^1J_{CP}$ 136.4, $^3J_{CP}$ 4.1), 53.8 (H–P–<u>C</u>H$_2$–N, dd, $^1J_{CP}$ 83.6, $^3J_{CP}$ 4.2), 61.3 (Ph–<u>C</u>H$_2$–N, t, $^3J_{HP}$ 3.5), 129.4 (*i*-Ph), 130.1 (*m*-Ph), 131.2 (*p*-Ph), 132.2 (*o*-Ph)
^{31}P NMR (D_2O + *t*BuOH / 85% aq H_3PO_4, pD = 0.4 + 0.4): 8.0 (HO–<u>P</u>, t, $^2J_{PH}$ 12.7, 1P), 10.9 (H–<u>P</u>, dt, $^1J_{PH}$ 556.4, $^2J_{PH}$ 10.5, 1P)
MS(+): 280 (280, $[M+H]^+$), 559 (559, $[2M+H]^+$)
MS(–): 278 (278, $[M–H]^-$), 557 (557, $[2M–H]^-$)
HRMS(+) (found (*calc*)): 280.0506 (*280.0498*, $C_9H_{16}NO_5P_2$)
TLC (conc. aq. NH_3 : EtOH = 1:{*x*}): 0.59 {1}, 0.25 {1.5}
EA(found (*calc* M · 0.5 H_2O)): C 37.79 (*37.51*), H 5.30 (*5.60*), N 4.90 (*4.86*), P 25.24 (*21.50*)

Bis{[(N-benzyl)-(N-methylphosphonic acid)-aminomethyl}phosphinic acid ***28b***.

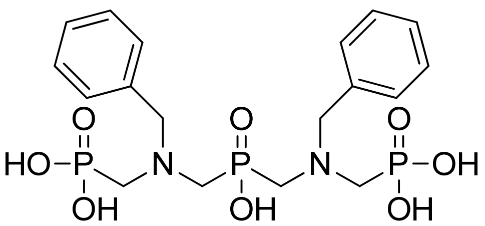

In 25-ml flask, (*N*-benzyl)-aminomethylphosphonic acid (100 mg, 0.5 mmol, 1 equiv.), paraformaldehyde (33 mg, 0.6 mmol, 2.2 equiv.) and H_3PO_2 (as 50% aq. solution, 66 mg, 1.0 mmol, 1 equiv.) were mixed with glacial AcOH (10 ml). The suspension was stirred at room temperature for 2 days and conversion was determined by ^{31}P NMR. Then, solvents were removed on rotary evaporator and an oily residue was co-evaporated with toluene (2×5 ml) and once with water (5 ml). An oily residue was dissolved in water (1 ml) and purified by C18 silica column chromatography (elution with gradient: pure water to ACN:water:TFA = 9:1:0.01). Fractions containing pure products were combined and evaporated to dryness. The residue was dissolved in water (2 ml) and the solution was left to crystallize in fridge (1–2 days). Solidified product was filtered off and washed with acetone (2 ml) and with Et_2O (2×5 ml). White powder, **28b**·$0.5H_2O$ (56 mg, 22 %).

^{1}H NMR (D_2O + *t*BuOH, pD = 0.6 + 0.4): 3.47 (HO–P–C<u>H</u>$_2$–N, d, $^2J_{HP}$ 12.4, 4H), 3.61 (C<u>H</u>$_2$–P–C<u>H</u>$_2$, d, $^2J_{HP}$ 9.1, 4H), 4.68 (Ph–C<u>H</u>$_2$–N, s, 4H), 7.47–7.67 (Ph, m, 10H)
^{13}C{^{1}H} NMR (D_2O + *t*BuOH, pD = 0.6 + 0.4): 51.6 (HO–P–<u>C</u>H$_2$–N, d, $^1J_{CP}$ 135.4), 53.4 (<u>C</u>H$_2$–P–<u>C</u>H$_2$, d, $^1J_{CP}$ 94.1), 61.9 (Ph–<u>C</u>H$_2$–N), 129.1 (*i*-Ph), 130.1 (*m*-Ph), 131.2 (*p*-Ph), 132.4 (*o*-Ph)
^{31}P NMR (D_2O + *t*BuOH / 85% aq H_3PO_4, pD = 0.6 + 0.4): 5.9–9.5 (HO–<u>P</u>, m, 2P), 14.5–17.2 (CH_2–<u>P</u>–CH_2, m, 1P)
MS(+): 493 (493, $[M+H]^+$), 515 (515, $[M+Na]^+$), 531 (531, $[M+K]^+$)
MS(–): 491 (491, $[M–H]^-$)
HRMS(+) (found (*calc*)): 515.0880 (*515.0872*, $C_{18}H_{27}N_2O_8P_3Na$)
TLC (conc. aq. NH_3 : MeOH = 1:{*x*}): 0.41 {1}, 0.17 {2}, 0.09 {5}
EA(found (*calc* M · $0.5H_2O$)): C 43.19 (*43.12*), H 5.40 (*5.63*), N 5.49 (*5.59*)

General procedure for reaction of phosphonylmethylated secondary amines (Table 4) in the paper text

(N-*Carboxymethyl)-(*N-*methylphosphonic acid)-aminomethyl*-H-*phosphinic acid* ***29***.

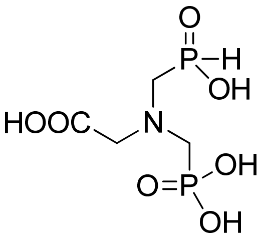

From 170 mg (1.0 mmol) of (*N*-acetic acid)-aminomethylphosphonic acid. White powder, **29** (143 mg, 58 %).

^{1}H NMR (D_2O + *t*BuOH, pD = –0.1 + 0.4): 3.64 (H–P–C<u>H</u>$_2$–N, d, $^2J_{HP}$ 10.5, 2H), 3.69 (HO–P–C<u>H</u>$_2$–N, d, $^2J_{HP}$ 12.5, 2H), 4.47 (N–C<u>H</u>$_2$–COOH, s, 2H), 7.30 (<u>H</u>–P, d, $^1J_{HP}$ 557.8, 1H)
^{13}C{^{1}H} NMR (D_2O + *t*BuOH, pD = –0.1 + 0.4): 53.2 (HO–P–<u>C</u>H$_2$–N, dd, $^1J_{CP}$ 136.7, $^3J_{CP}$ 3.9), 55.7 (H–P–<u>C</u>H$_2$–N, dd, $^1J_{CP}$ 83.2, $^3J_{CP}$ 3.4), 56.9 (HOOC–<u>C</u>H$_2$–N, t, $^3J_{CP}$ 3.9), 168.7 (N–CH$_2$–<u>C</u>OOH)
^{31}P NMR (D_2O + *t*BuOH / 85% aq H_3PO_4, pD = –0.1 + 0.4): 7.1 (HO–<u>P</u>, t, $^2J_{PH}$ 12.3, 1P), 10.2 (H–<u>P</u>, dt, $^1J_{PH}$ 557.8, $^2J_{PH}$ 10.5, 1P)
MS(+): 248 (248, $[M+H]^+$), 495 (495, $[2M+H]^+$), 517 (517, $[2M+Na]^+$)
MS(–): 246 (246, $[M-H]^-$), 493 (493, $[2M-H]^-$)
HRMS(+) (found (*calc*)): 269.9905 (*269.9903*, $C_4H_{11}NO_7P_2Na$)
TLC (conc. aq. NH_3 : MeOH = 1:{*x*}): 0.63 {1}, 0.29 {2}, 0.11 {5}
EA (M): C 19.77 (*19.44*), H 4.68 (*4.46*), N 5.16 (*5.67*), P 22.63 (*25.07*)

N,N-*Bis(methylphosphonic acid)-aminomethyl*-H-*phosphinic acid* ***30***.

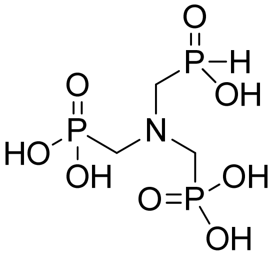

From 205 mg (1.0 mmol) of amino-*N,N*-bis(methylphosphonic acid). Viscous oil, **30** (195 mg, 69 %).

^{1}H NMR (D_2O + *t*BuOH, pD = –0.2 + 0.4): 3.73 (H–P–C<u>H</u>$_2$–N, d, $^2J_{HP}$ 10.5, 2H), 3.81 (HO–P–C<u>H</u>$_2$–N, d, $^3J_{HH}$ 12.7, 4H), 7.33 (<u>H</u>–P, dt, $^1J_{HP}$ 563.2, $^3J_{HH}$ 1.8, 1H)
^{13}C{^{1}H} NMR (D_2O + *t*BuOH, pD = –0.2 + 0.4): 53.9 (HO–P–<u>C</u>H$_2$–N, dt, $^1J_{CP}$ 138.0, $^3J_{CP}$ 3.9), 55.3–55.6 and 56.1–56.4 (H–P–<u>C</u>H$_2$–N, dm, $^1J_{CP}$ 85.2)
^{31}P NMR (D_2O + *t*BuOH / 85% aq H_3PO_4, pD = –0.2 + 0.4): 9.0 (HO–<u>P</u>, t, $^2J_{PH}$ 12.6, 2P), 12.1 (H–<u>P</u>, dt, $^1J_{PH}$ 563.5, $^2J_{PH}$ 10.5, 1P)
MS(+): 284 (284, $[M+H]^+$), 567 (567, $[2M+H]^+$)
MS(–): 282 (282, $[M-H]^-$), 565 (565, $[2M-H]^-$)
HRMS(–) (found (*calc*)): 281.9708 (*281.9703*, $C_3H_{11}NO_8P_3$)
TLC (conc. aq. NH_3 : MeOH = 1:{*x*}): 0.29 {1}, 0.13 {2}, 0.00 {5}

(N,N′-*Dibenzyl)-ethylenediamine*-N,N′-*bis(methyl*-H-*phosphinic acid)* ***31***.

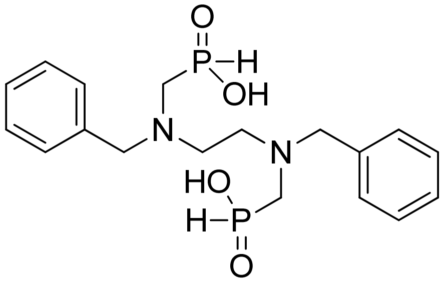

In 25-ml flask, *N,N'*-dibenzyl-ethylenediamine (240 µl, 1.0 mmol, 1 equiv.), paraformaldehyde (120 mg, 4.0 mmol, 4 equiv.), and H_3PO_2 (as 50% aq. solution, 290 mg, 2.2 mmol, 2.2 equiv.) were mixed with glacial AcOH (10 ml). The suspension was heated at 40 °C for 1 day and conversion was determined by ^{31}P NMR. Then, solvents were removed on rotary evaporator and the oily residue was co-evaporated with toluene (2×5 ml) and once with water (5 ml). Then, the oily residue was purified by flash silica column chromatography (C18, gradient from pure water to ACN:water:TFA = 9:1:0.01). Fractions containing pure product were combined and concentrated *in vacuo*. The residue was suspended / dissolved in water (10 ml) and left to finish crystallization in fridge. After 1 day, the solids were filtered off, washed with acetone (5 ml) and with Et_2O (2×5 ml). White powder, **31**·$2H_2O$ (220 mg, 51 %).

^{1}H NMR (D_2O + *t*BuOH, pD = 12.4 + 0.4): 2.66 (P–C$\underline{H}_2$–N, d, $^{2}J_{HP}$ 10.2, 4H), 2.76 (N–C$\underline{H}_2$–C$\underline{H}_2$–N, s, 4H), 3.76 (Ph–C$\underline{H}_2$–N, s, 4H), 7.00 ($\underline{H}$–P, d, $^{1}J_{HP}$ 510.6, 2H), 7.24–7.48 (Ph, m, 10H)
^{13}C{^{1}H} NMR (D_2O + *t*BuOH, pD = 12.4 + 0.4): 51.7 (N–$\underline{C}H_2$–$\underline{C}H_2$–N, d, $^{3}J_{CP}$ 8.6), 55.8 (P–$\underline{C}H_2$–N, d, $^{1}J_{CP}$ 102.5), 59.8 (Ph–$\underline{C}H_2$–N, d, $^{3}J_{CP}$ 6.1), 128.4 (*p*-Ph), 129.2 (Ph), 130.8 (Ph), 137.9 (*i*-Ph)
^{31}P NMR (D_2O + *t*BuOH / 85% aq H_3PO_4, pD = 12.4 + 0.4): 23.7 (dt, $^{1}J_{PH}$ 510.8, $^{2}J_{PH}$ 10.5)
MS(+): 419 (419, $[M+Na]^+$)
MS(–): 395 (395, $[M–H]^-$)
HRMS(+) (found (*calc*)): 397.1390 (*397.1446*, $C_{18}H_{27}N_2O_4P_2$)
TLC (conc. aq. NH_3 : EtOH = 1:{*x*}): 0.70 {5}, 0.55 {10}, 0.48 {20}, 0.44 {35}
EA(found (*calc* M · $2H_2O$)): C 49.58 (*49.48*), H 6.90 (*7.04*), N 6.21 (*6.41*), P 14.61 (*14.18*)

(N,N′-*Dibenzyl)-ethylenediamine-(*N-*methyl)-(*N′-*methyl*-H-*phosphinic acid)* ***31-Me***.

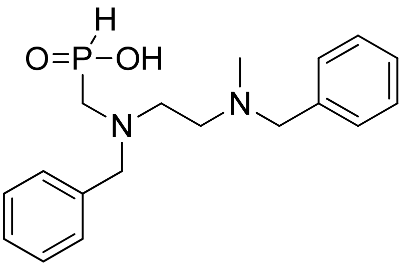

In 25-ml flask, *N,N'*-dibenzyl-ethylenediamine (240 µl, 1.0 mmol, 1 equiv.), paraformaldehyde (120 mg, 4.0 mmol, 4 equiv.) and H_3PO_2 (as 50% aq. solution, 290 mg, 2.2 mmol, 2.2 equiv.) were mixed with glacial AcOH (10 ml). The suspension was heated at 40 °C for 1 day and conversion was determined by ^{31}P NMR. Then, solvents were removed on rotary evaporator and the oily residue was co-evaporated with toluene (2×5 ml) and once with water (5 ml). Then, the oily residue was purified on flash silica column chromatography (C18, gradient from pure water to ACN:water:TFA = 9:1:0.01). Fractions containing pure product were combined and concentrated *in vacuo*. Viscous oil (33 mg, 10 %).

^{1}H NMR (D_2O + *t*BuOH, pD = 3.4 + 0.4): 2.68 (C<u>H</u>$_3$–N, s, 3H), 2.86 (P–C<u>H</u>$_2$–N, dd, $^2J_{HP}$ 9.0, $^3J_{HH}$ 1.8 Hz, 2H), 3.14 (N–C<u>H</u>$_2$–CH$_2$–N–CH$_3$, t, $^3J_{HH}$ 6.2, 2H), 3.28 (N–CH$_2$–C<u>H</u>$_2$–N–CH$_3$, t, $^3J_{HH}$ 6.1, 2H), 3.91 (Ph–C<u>H</u>$_2$–N–CH$_2$–P, s, 2H), 4.14 (Ph–C<u>H</u>$_2$–N–CH$_3$, s, 2H), 6.83 (<u>H</u>–P, dt, $^1J_{HP}$ 525.7, $^3J_{HH}$ 1.7, 1H), 7.34–7.59 (Ph, m, 10H)
^{13}C{^{1}H} NMR (D_2O + *t*BuOH, pD = 3.4 + 0.4): 40.4 (<u>C</u>H$_3$–N), 49.8 (N–<u>C</u>H$_2$–CH$_2$–N–CH$_3$, d, $^3J_{CP}$ 6.6), 52.8 (N–CH$_2$–<u>C</u>H$_2$–N–CH$_3$), 54.4 (P–<u>C</u>H$_2$–N, d, $^1J_{CP}$ 102.6), 59.6 (Ph–<u>C</u>H$_2$–N–CH$_3$), 61.9 (Ph–<u>C</u>H$_2$–N–CH$_2$–P, d, $^3J_{CP}$ 4.7), 129.3 (Ph), 2× 129.6 (Ph), 130.0 (Ph), 130.8 (2× Ph), 131.0 (Ph), 131.6 (Ph)
^{31}P NMR (D_2O + *t*BuOH / 85% aq H_3PO_4, pD = 3.4 + 0.4): 19.4–20.2 and 22.6–23.4 (dm, $^1J_{PH}$ 525.9)
MS(+): 333 (333, [M+H]$^+$), 354 (354, [M+Na]$^+$), 665 (665, [2M+H]$^+$)
MS(–): 331 (331, [M–H]$^-$), 663 (663, [2M–H]$^-$)
HRMS(+) (found (*calc*)): 333.1735 (*333.1726*, $C_{18}H_{26}N_2O_2P$)
TLC (conc. aq. NH_3 : EtOH = 1:{*x*}): 0.84 {5}, 0.72 {10}, 0.67 {20}, 0.61 {35}

General procedure for reaction of poly-secondary amines (Table 5) in the paper text

(N,N′-*Dibenzyl)-propylenediamine*-N,N′-*bis(methyl*-H-*phosphinic acid)* ***32***.

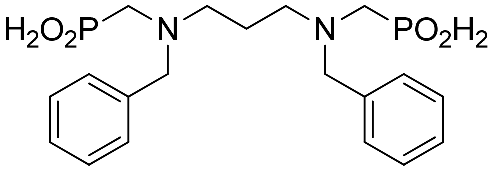

From 82 mg (0.25 mmol) of *N,N′*-dibenzyl-propylenediamine·2HCl converted to its acetate salt on Dowex 1 in OH^--form, elution off with 20% aq. AcOH. Viscous oil (70 mg, 68 %).

^{1}H NMR (D_2O + *t*BuOH, pD = 4.8 + 0.4): 2.15–2.32 (CH_2–$C\underline{H}_2$–CH_2, m, 2H), 3.27 (P–$C\underline{H}_2$–N, d, $^{3}J_{HP}$ 11.2, 4H), 3.28–3.34 ($C\underline{H}_2$–CH_2–$C\underline{H}_2$, m, 4H), 4.51 (Ph–$C\underline{H}_2$–N, s, 4H), 7.05 ($\underline{H}$–P, dt, $^{1}J_{HP}$ 551.4, $^{3}J_{HH}$ 1.5, 2H), 7.47–7.62 (Ph, m, 10H)
^{13}C{^{1}H} NMR (D_2O + *t*BuOH, pD = 4.8 + 0.4): 19.7 (CH_2–$\underline{C}H_2$–CH_2), 52.1 ($\underline{C}H_2$–CH_2–$\underline{C}H_2$, d, $^{3}J_{CP}$ 4.1 Hz), 52.7 (P–$\underline{C}H_2$–N, d, $^{1}J_{CP}$ 82.9), 60.4 (Ph–$\underline{C}H_2$–N, d, $^{3}J_{CP}$ 3.7), 129.3 (*i*-Ph), 130.2 (*m*-Ph), 131.2 (*p*-Ph), 131.9 (*o*-Ph)
^{31}P NMR (D_2O + *t*BuOH / 85% aq H_3PO_4, pD = 4.8 + 0.4): 10.6 (dt, $^{1}J_{PH}$ 551.4, $^{2}J_{PH}$ 10.5)
MS(+): 455 (455, $[M+2Na–H]^+$)
MS(–): 409 (409, $[M–H]^-$)
HRMS(+) (found (*calc*)): 411.1606 (*411.1597*, $C_{19}H_{29}N_2O_4P_2$)
TLC conc. aq. NH_3 : EtOH = 1:{*x*}): 0.72 {5}, 0.44 {10}, 0.41 {20}, 0.36 {35}

(N,N′-*Dibenzyl)-hexylenediamine*-N,N′-*bis(methyl*-H-*phosphinic acid)* ***33***.

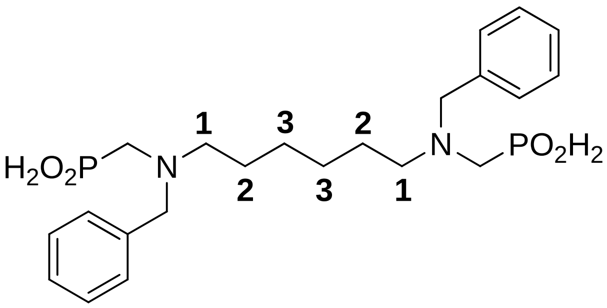

From 93 mg (0.25 mmol) of *N,N′*-dibenzyl-hexylenediamine·2HCl converted to its acetate salt on Dowex1 in OH^--form, elution off with 20% aq. AcOH. Viscous oil (91 mg, 80 %).

^{1}H NMR (D_2O + *t*BuOH, pD = 5.0 + 0.4): 1.30–1.40 (**3**, m, 4H), 1.66–1.86 (**2**, m, 4H), 3.21–3.27 (**1**, m, 4H), 3.26 (P–$C\underline{H}_2$–N, d, $^{2}J_{HP}$ 10.7, 4H), 4.50 (Ph–$C\underline{H}_2$–N, bs, 4H), 7.09 ($\underline{H}$–P, dt, $^{1}J_{HP}$ 549.6, $^{3}J_{HH}$ 1.6, 2H), 7.47–7.61 (Ph, m, 10H)
^{13}C{^{1}H} NMR (D_2O + *t*BuOH, pD = 5.0 + 0.4): 23.6 (**2**), 25.7 (**3**), 52.4 (P–$\underline{C}H_2$–N, d, $^{1}J_{CP}$ 83.6), 55.3 (**1**, d, $^{3}J_{CP}$ 4.0), 59.9 (Ph–$\underline{C}H_2$–N, d, $^{3}J_{CP}$ 3.6), 129.5 (*i*-Ph), 130.1 (*m*-Ph), 131.0 (*p*-Ph), 131.9 (*o*-Ph)
^{31}P NMR (D_2O + *t*BuOH / 85% aq H_3PO_4, pD = 5.0 + 0.4): 10.7 (dt, $^{1}J_{PH}$ 549.6, $^{2}J_{PH}$ 10.6)
MS(+): 453 (453, $[M+H]^+$), 475 (475, $[M+Na]^+$)
MS(–): 451 (451, $[M–H]^-$), 225 (225, $[M–2H]^{2–}$)
HRMS(+) (found (*calc*)): 453.2080 (*453.2067*, $C_{22}H_{35}N_2O_4P_2$)
TLC (conc. aq. NH_3 : EtOH = 1:{*x*}): 0.81 {5}, 0.66 {10}, 0.56 {20}, 0.52 {35}

(N,N″-*Dibenzyl)-propylenetriamine*-N,N′,N″-*tris(methyl*-H-*phosphinic acid)* ***34***.

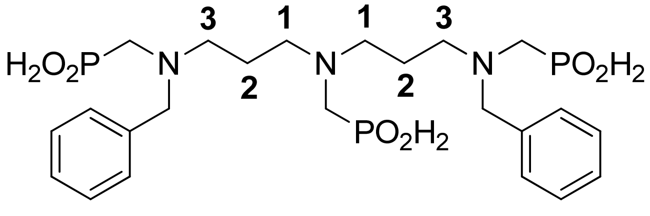

From 105 mg (0.25 mmol) of *N,N″*-dibenzyl-dipropylenetriamine·3HCl converted to its acetate salt on Dowex 1 in OH^--form, elution off with 20% aq. AcOH. Viscous oil (116 mg, 85 %).

^{1}H NMR (D_2O + *t*BuOH, pD = 4.7 + 0.4): 2.13–2.32 (**2**, m, 4H), 3.23 (P–CH$_2$–N, dt, $^{2}J_{HP}$ 10.2, $^{3}J_{HH}$ 1.5, 2H), 3.25–3.34 (**1**, m, 4H), 3.30 (2× P–CH$_2$–N, dt, $^{2}J_{HP}$ 10.4, $^{3}J_{HH}$ 1.5, 4H), 3.39 (**1**, t, $^{3}J_{HH}$ 8.0, 4H), 4.55 (Ph–CH$_2$–N, s, 4H), 7.06 (H–P, dt, $^{1}J_{HP}$ 551.8, $^{3}J_{HH}$ 1.5, 2H), 7.17 (H–P, dt, $^{1}J_{HP}$ 546.9, $^{3}J_{HH}$ 1.5, 1H), 7.48–7.64 (Ph, m, 10H)
^{13}C{^{1}H} NMR (D_2O + *t*BuOH, pD = 4.7 + 0.4): 19.9 (**2**), 52.3 (**3**, d, $^{3}J_{CP}$ 3.9), 52.6 (2× P–CH$_2$–N, d, $^{1}J_{CP}$ 83.0), 52.8 (**1**, d, $^{3}J_{CP}$ 4.3), 53.0 (P–CH$_2$–N, d, $^{1}J_{CP}$ 84.4), 60.6 (Ph–CH$_2$–N, d, $^{3}J_{CP}$ 3.7), 129.3 (*i*-Ph), 130.2 (*m*-Ph), 131.2 (*p*-Ph), 131.9 (*o*-Ph)
^{31}P NMR (D_2O + *t*BuOH / 85% aq H_3PO_4, pD = 4.7 + 0.4): 10.6 (dt, $^{1}J_{PH}$ 551.8, $^{2}J_{PH}$ 10.4, 2P), 11.1 (dt, $^{1}J_{PH}$ 546.3, $^{2}J_{PH}$ 7.1, 1P)
MS(–): 544 (544, $[M–H]^-$), 272 (272, $[M–2H]^{2-}$)
HRMS(+) (found (*calc*)): 568.1877 (*568.1866*, $C_{23}H_{38}N_3O_6P_3Na$)
TLC (conc. aq. NH_3 : EtOH = 1:{*x*}): 0.68 {5}, 0.33 {10}, 0.24 {20}, 0.21 {35}

(N,N″-*Dibenzyl)-hexylenetriamine*-N,N′,N′-*tris(methyl*-H-*phosphinic acid)* ***35***.

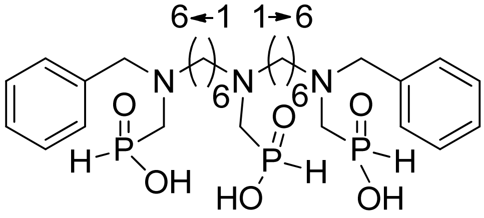

From 133 mg (0.25 mmol) of *N,N″*-dibenzyl-dipropylenetriamine·3HCl·3/2H_2O converted to its acetate salt on Dowex 1 in OH^--form, elution off with 20% aq. AcOH. Viscous oil (129 mg, 82 %).

^{1}H NMR (D_2O + *t*BuOH, pD = 2.7 + 0.4): 1.33–1.49 (**3** + **4**, m, 8H), 1.64–1.91 (**2** + **5**, m, 8H), 3.21–3.42 (**1** + **6** + 2× P–CH$_2$–N + P–CH$_2$–N, m, 14H), 4.40–4.64 (Ph–CH$_2$–N, m, 4H), 7.09 (H–P, dt, $^{1}J_{HP}$ 550.7, $^{3}J_{HH}$ 1.3, 2H), 7.24 (H–P, dt, $^{1}J_{HP}$ 545.8, $^{3}J_{HH}$ 1.2, 1H), 7.48–7.63 (Ph, m, 10H)
^{13}C{^{1}H} NMR (D_2O + *t*BuOH, pD = 2.7 + 0.4): 2× 23.7 (**2** + **5**), 2× 25.8 (**3** + **4**), 52.4 (2× P–CH$_2$–N, d, $^{1}J_{CP}$ 83.7), 52.9 (P–CH$_2$–N, d, $^{1}J_{CP}$ 84.3), 55.4 (**1**, d, $^{4}J_{CP}$ 3.9), 55.8 (**6**, d, $^{4}J_{CP}$ 3.8), 59.9 (Ph–CH$_2$–N, d, $^{3}J_{CP}$ 3.5), 129.5 (*i*-Ph), 130.1 (*m*-Ph), 131.0 (*p*-Ph), 131.9 (*o*-Ph)
^{31}P NMR (D_2O + *t*BuOH / 85% aq H_3PO_4, pD = 2.7 + 0.4): 10.0 (dt, $^{1}J_{PH}$ 546.5, $^{2}J_{PH}$ 11.0, 1P), 10.2 (dt, $^{1}J_{PH}$ 549.2, $^{2}J_{PH}$ 10.6, 2P)
MS(+): 630 (630, $[M+H]^+$)
MS(–): 628 (628, $[M–H]^-$)
HRMS(+) (found (*calc*)): 630.2997 (*630.2985*, $C_{29}H_{51}N_3O_6P_3$),
TLC (conc. aq. NH_3 : EtOH = 1:{*x*}): 0.71 {5}, 0.50 {10}, 0.39 {20}, 0.32 {35}

General procedure for oxidation of phosphinic acids to corresponding phosphonic acids and benzyl group removal (Table 6) in the paper text

(N,N′-*Dibenzyl)-ethylenediamine*-N,N′-*bis(methylphosphonic acid)* ***31a***.

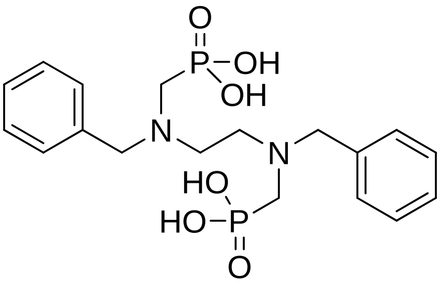

Procedure **D**.

From 110 mg (0.25 mmol) of **31**. White powder **31a**·0.25H_2O (110 mg, 95 %).

^{1}H NMR (D_2O + *t*BuOH, pD = 11.4 + 0.4): 2.67 (P–C<u>H</u>$_2$–N, d, $^2J_{HP}$ 11.5, 4H), 2.80 (N–C<u>H</u>$_2$–C<u>H</u>$_2$–N, s, 4H), 3.84 (Ph–C<u>H</u>$_2$–N, s, 4H), 7.27–7.44 (Ph, m, 10H)
^{13}C{^{1}H} NMR (D_2O + *t*BuOH, pD = 11.4 + 0.4): 51.3 (N–<u>C</u>H$_2$–<u>C</u>H$_2$–N, d, $^3J_{CP}$ 7.9), 53.9 (P–<u>C</u>H$_2$–N, d, $^1J_{CP}$ 137.4), 58.9 (Ph–<u>C</u>H$_2$–N, d, $^3J_{CP}$ 3.9), 128.2 (*p*-Ph), 129.1 (*m*-Ph), 131.0 (*o*-Ph), 138.1 (*i*-Ph)
^{31}P NMR (D_2O + *t*BuOH / 85% aq H_3PO_4, pD = 11.4 + 0.4): 15.1 (t, $^2J_{PH}$ 11.3)
MS(+): 473 (473, [M–H+2Na]$^+$), 495 (495, [M–2H+3Na]$^+$)
HRMS(+) (found (*calc*)): 451.1163 (*451.1158*, $C_{18}H_{26}N_2O_6P_2Na$)
TLC (conc. aq. NH_3 : MeOH = 1:{*x*}): 0.70 {1}, 0.33 {2}, 0.17 {5}
EA (found (*calc* M · 1/4H_2O)): C 50.01 (*49.95*), H 6.03 (*6.17*), N 6.61 (*6.47*), P 13.82 (*14.31*)

Ethylenediamine-N,N*'-bis(methylphosphonic acid)* ***31b***.

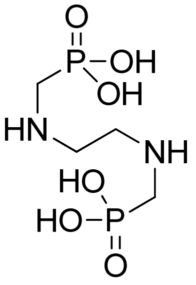

Procedure **E**.

From 87 mg (0.20 mmol) of **31a**. White powder **31b**·2H_2O (43 mg, 76 %).

^{1}H NMR (D_2O + *t*BuOH, pD = 6.9 + 0.4): 3.03 (P–C<u>H</u>$_2$–N, d, $^2J_{HP}$ 11.7, 4H), 3.53 (N–C<u>H</u>$_2$–C<u>H</u>$_2$–N, s, 4H)
^{13}C{^{1}H} NMR (D_2O + *t*BuOH, pD = 6.9 + 0.4): 46.0 (N–<u>C</u>H$_2$–<u>C</u>H$_2$–N, d, $^3J_{CP}$ 7.5), 46.4 (P–<u>C</u>H$_2$–N, d, $^1J_{CP}$ 130.0)
^{31}P NMR (D_2O + *t*BuOH / 85% aq H_3PO_4, pD = 6.9 + 0.4): 8.6 (t, $^2J_{PH}$ 11.9)
MS(+): 293 (293, [M–H+2Na]$^+$)
HRMS(+) (found (*calc*)): 271.0224 (*271.0219*, $C_4H_{14}N_2O_6P_2Na$)
TLC (conc. aq. NH_3 : MeOH = 1:{*x*}): 0.63 {1}, 0.07 {2}, 0.03 {5}
EA (found (*calc* M · 2H_2O)): C 17.31 (*16.91*), H 6.02 (*6.39*), N 9.52 (*9.86*), P 20.82 (*21.80*)

(N,N′*-Dibenzyl)-propylenediamine-*N,N′*-bis(methylphosphonic acid)* ***32a***.

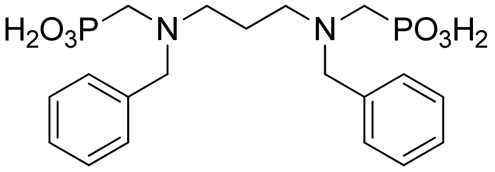

Procedure **C**.

From 103 mg (0.25 mmol) of **32**. Viscous oil (107 mg, 97 %).

^{1}H NMR (D_2O + *t*BuOH, pD = 1.0 + 0.4): 2.21–2.35 (CH_2–$C\underline{H}_2$–CH_2, m, 2H), 3.31 ($C\underline{H}_2$–CH_2–$C\underline{H}_2$, t, $^3J_{HH}$ 8.1, 4H), 3.36 (P–$C\underline{H}_2$–N, d, $^3J_{HP}$ 12.9, 4H), 4.56 (Ph–$C\underline{H}_2$–N, bs, 4H), 7.48–7.63 (Ph, m, 10H)
^{13}C{^{1}H} NMR (D_2O + *t*BuOH, pD = 1.0 + 0.4): 19.3 (CH_2–$\underline{C}H_2$–CH_2) , 49.6 (P–$\underline{C}H_2$–N, d, $^1J_{CP}$ 136.5), 51.2 ($\underline{C}H_2$–CH_2–$\underline{C}H_2$, d, $^3J_{CP}$ 4.1), 60.0 (Ph–$\underline{C}H_2$–N, d, $^3J_{CP}$ 4.1), 129.2 (*i*-Ph), 130.1 (*m*-Ph), 131.1 (*p*-Ph), 131.9 (*o*-Ph)
^{31}P NMR (D_2O + *t*BuOH / 85% aq H_3PO_4, pD = 1.0 + 0.4): 8.7 (t, $^2J_{PH}$ 12.8)
MS(+): 443 (443, $[M+H]^+$), 465 (465, $[M+Na]^+$)
MS(–): 441 (441, $[M–H]^-$)
HRMS(+) (found (*calc*)): 443.1508 (*443.1495*, $C_{19}H_{29}N_2O_6P_2$)
TLC (conc. aq. NH_3 : MeOH = 1:{*x*}): 0.74 {1}, 0.57 {2}, 0.40 {5}

*Propylenediamine-*N,N′*-bis(methylphosphonic acid)* ***32b***.

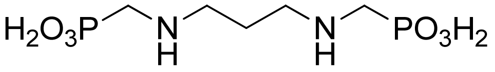

Procedure **E**.

From 88 mg (0.20 mmol) of **32a**. White powder, **32b**·1/2H_2O (43 mg, 81 %).

^{1}H NMR (D_2O + *t*BuOH, pD = 5.5 + 0.4): 2.13–2.27 (CH_2–$C\underline{H}_2$–CH_2, m, 2H), 3.06 (P–$C\underline{H}_2$–N, d, $^2J_{HP}$ 12.0, 4H), 3.28 ($C\underline{H}_2$–CH_2–$C\underline{H}_2$, t, $^3J_{HH}$ 7.8, 4H)
^{13}C{^{1}H} NMR (D_2O + *t*BuOH, pD = 5.5 + 0.4): 23.2 (CH_2–$\underline{C}H_2$–CH_2), 45.9 (P–$\underline{C}H_2$–N, d, $^1J_{CP}$ 131.1), 46.8 ($\underline{C}H_2$–CH_2–$\underline{C}H_2$, d, $^3J_{CP}$ 6.7)
^{31}P NMR (D_2O + NaOD + *t*BuOH / 85% aq H_3PO_4, pD = 5.5 + 0.4): 8.2 (t, $^2J_{PH}$ 12.3)
MS(+): 263 (263, $[M+H]^+$), 329 (329, $[M–2H+3Na]^+$)
HRMS(+) (found (*calc*)): 285.0383 (*285.0376*, $C_5H_{16}N_2O_6P_2Na$)
TLC (conc. aq. NH_3 : MeOH = 1:{*x*}): 0.56 {1}, 0.11 {2}, 0.03 {5}
EA(found (*calc* M · 1/2H_2O)): C 22.30 (*22.15*), H 5.93 (*6.32*), N 9.14 (*9.33*)

(N,N′*-Dibenzyl)-hexylenediamine-*N,N′*-bis(methylphosphonic acid)* ***33a***

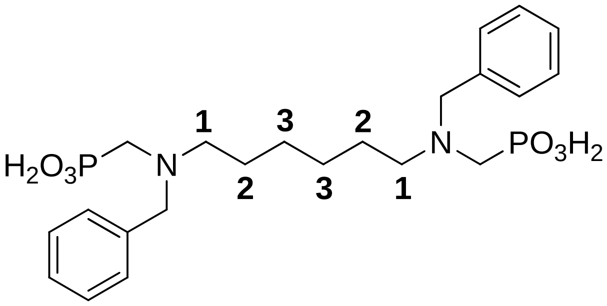

<u>Procedure **C**</u>.

From 113 mg (0.25 mmol) of **33**. Viscous oil (115 mg, 95 %).

^{1}H NMR (D_2O + *t*BuOH, pD = 1.3 + 0.4): 1.28–1.40 (**3**, m, 4H), 1.62–1.90 (**2**, m, 4H), 3.17–3.33 (**1**, m, 4H), 3.34 (P–C<u>H</u>$_2$–N, dt, $^{2}J_{HP}$ 12.9, $^{3}J_{HH}$ 1.4, 4H), 4.37–4.72 (Ph–C<u>H</u>$_2$–N, m, 4H), 7.47–7.64 (Ph, m, 10H)
^{13}C{^{1}H} NMR (D_2O + *t*BuOH, pD = 1.3 + 0.4): 23.4 (**2**), 25.7 (**3**), 49.5 (P–<u>C</u>H$_2$–N, d, $^{1}J_{CP}$ 137.6), 54.5 (**1**, d, $^{3}J_{CP}$ 4.2), 59.4 (Ph–<u>C</u>H$_2$–N, d, $^{3}J_{CP}$ 4.0), 129.6 (*i*-Ph), 130.0 (*m*-Ph), 130.9 (*p*-Ph), 131.9 (*o*-Ph)
^{31}P NMR (D_2O + *t*BuOH / 85% aq H_3PO_4, pD = 1.3 + 0.4): 7.9 (t, $^{2}J_{PH}$ 12.9)
MS(+): 485 (485, $[M+H]^+$), 507 (507, $[M+Na]^+$)
MS(–): 483 (483, $[M–H]^-$)
HRMS(+) (found (*calc*)): 507.1802 (*507.1784*, $C_{22}H_{34}N_2O_6P_2Na$)
TLC (conc. aq. NH_3 : MeOH = 1:{*x*}): 0.67 {1}, 0.36 {2}, 0.13 {5}

*Hexylenediamine-*N,N′*-bis(methylphosphonic acid)* ***33b***.

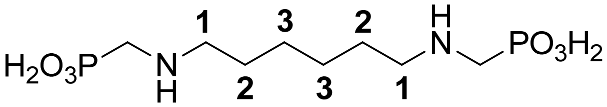

<u>Procedure **C**</u>.

From 97 mg (0.20 mmol) of **33a**. Viscous oil (45 mg, 74 %).

^{1}H NMR (D_2O + *t*BuOH, pD = 11.6 + 0.4): 1.33–1.46 (**3**, m, 4H), 1.55–1.68 (**2**, m, 4H), 2.76 (P–C<u>H</u>$_2$–N, d, $^{2}J_{HP}$ 12.1, 4H), (**1**, t, $^{3}J_{HH}$ 7.6, 4H)
^{13}C{^{1}H} NMR (D_2O + *t*BuOH, pD = 11.6 + 0.4): 26.4 (**3**), 27.5 (**2**), 47.5 (P–<u>C</u>H$_2$–N, d, $^{1}J_{CP}$ 131.9), 50.8 (**1**, d, $^{3}J_{CP}$ 8.9)
^{31}P NMR (D_2O + *t*BuOH / 85% aq H_3PO_4, pD = 11.6 + 0.4): 12.4 (t, $^{2}J_{PH}$ 11.6)
HRMS(+) (found (*calc*)): 327.0851 (*327.0845*, $C_8H_{22}N_2O_6P_2Na$)
TLC (conc. aq. NH_3 : MeOH = 1:{*x*}): 0.74 {1}, 0.11 {2}, 0.03 {5}

(N,N''-*Dibenzyl)-propylenetriamine*-N,N',N''-*tris(methylphosphonic acid)* ***34a***.

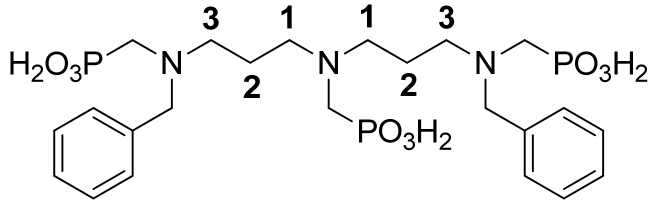

Procedure **C**.

From 136 mg (0.25 mmol) of **34**. Viscous oil(141 mg, 95 %).

^{1}H NMR (D_2O + *t*BuOH, pD = 1.2 + 0.4): 2.19–2.39 (**2**, m, 4H), 3.30–3.50 (**3** + **1**, m, 8H),3.38 **(**2× P–CH2–N + P–CH2–N, d, $^{2}J_{HP}$ 12.9, 4H), 4.59 (Ph–CH2–N, s, 4H), 7.47–7.63 (Ph, m, 10H)
^{13}C{^{1}H} NMR (D_2O + *t*BuOH, pD = 1.2 + 0.4): 19.4 (**2**), 49.3 (2× P–CH2–N, d, $^{1}J_{CP}$ 137.1), 50.1 (P–CH2–N, d, $^{1}J_{CP}$ 136.4), 51.3 (**3**, d, $^{3}J_{CP}$ 3.5), 52.5 (**1**, d, $^{3}J_{CP}$ 4.2), 60.3 (Ph–CH2–N, d, $^{3}J_{CP}$ 3.7), 129.3 (*i*-Ph), 130.1 (*m*-Ph), 131.1 (*p*-Ph), 132.0 (*o*-Ph)
^{31}P NMR (D_2O + *t*BuOH / 85% aq H_3PO_4, pD = 1.2 + 0.4): 7.5 (t, $^{2}J_{PH}$ 12.8, 1P), 7.9 (t, $^{2}J_{PH}$ 12.8, 2P)
MS(+): 594 (594, $[M+H]^+$), 615 (615, $[M+Na]^+$)
MS(–): 592 (592, $[M-H]^-$)
HRMS(+) (found (*calc*)): 594.1904 (*594.1894*, $C_{23}H_{39}N_3O_9P_3$)
TLC (conc. aq. NH_3 : MeOH = 1:{*x*}): 0.63 {1}, 0.20 {2}, 0.17 {5}

Propylenetriamine-N,N',N''-*tris(methylphosphonic acid)* ***34b***.

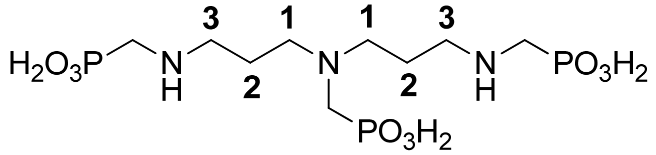

Procedure **C**.

From 119 mg (0.20 mmol) of **34a**.Viscous oil (73 mg, 88 %).

^{1}H NMR (D_2O + *t*BuOH, pD = 1.1 + 0.4): 2.18–2.34 (**2**, m, 4H), 3.26 (2× P–CH2–N, d, $^{2}J_{HP}$ 12.9, 4H), 3.29 (**3**, t, $^{3}J_{HH}$ 7.8, 4H), 3.44 (P–CH2–N, d, $^{2}J_{HP}$ 12.9, 2H), 3.44–3.55 (**1**, m, 4H)
^{13}C{^{1}H} NMR (D_2O + *t*BuOH, pD = 1.1 + 0.4): 21.1 (**2**), 44.6 (2× P–CH2–N, d, $^{1}J_{CP}$ 139.4), 46.5 (**3**, d, $^{3}J_{CP}$ 7.5), 49.9 (P–CH2–N, d, $^{1}J_{CP}$ 136.4), 52.7 (**1**, d, $^{3}J_{CP}$ 4.2)
^{31}P NMR (D_2O + *t*BuOH / 85% aq H_3PO_4, pD = 1.1 + 0.4): 7.7 (t, $^{2}J_{PH}$ 12.8, 1P), 9.7 (t, $^{2}J_{PH}$ 12.8, 2P)
MS(+): 414 (414, $[M+H]^+$)
MS(–): 412 (412, $[M-H]^-$)
HRMS(+) (found (*calc*)): 436.0777 (*436.0774*, $C_9H_{26}N_3O_9P_3Na$)
TLC (conc. aq. NH_3 : MeOH = 1:{*x*}): 0.44 {1}, 0.00 {2}

(N,N''-*Dibenzyl)-hexylenetriamine*-N,N',N''-*tris(methylphosphonic acid)* ***35a***.

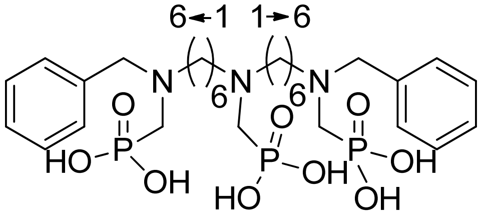

Procedure **C**.

From 157 mg (0.25 mmol) of **35**.Viscous oil (164 mg, 97 %).

^{1}H NMR (D_2O + *t*BuOH, pD = 0.4 + 0.4): 1.32–1.46 (**3** + **4**, m, 8H), 1.64–1.92 (**2** + **5**, m, 8H), 3.17–3.36 (**1** + **6**, m, 8H), 3.33 (1× P–C$\underline{H}_2$–N, d, $^2J_{HP}$ 13.1, 2H), 3.35 (2× P–C$\underline{H}_2$–N, d, $^2J_{HP}$ 12.9, 4H), 4.38–4.70 (Ph–C$\underline{H}_2$–N, m, 4H), 7.49–7.62 (Ph, m, 10H)
^{13}C{^{1}H} NMR (D_2O + *t*BuOH, pD = 0.4 + 0.4): 2× 23.5 (**2** + **5**), 2× 25.8 (**3** + **4**), 49.5 (2× P–$\underline{C}H_2$–N, d, $^1J_{CP}$ 137.5), 49.9 (1× P–$\underline{C}H_2$–N, d, $^1J_{CP}$ 137.7), 54.6 (**6**, d, $^3J_{CP}$ 3.9), 55.4 (**1**, d, $^3J_{CP}$ 3.6), 59.4 (Ph–$\underline{C}H_2$–N, d, $^3J_{CP}$ 4.1), 129.6 (*i*-Ph, d, $^4J_{CP}$ 0.3), 130.0 (*m*-Ph), 130.9 (*p*-Ph), 131.9 (*o*-Ph)
^{31}P NMR (D_2O + *t*BuOH / 85% aq H_3PO_4, pD = 0.4 + 0.4): 8.5 (t, $^2J_{PH}$ 13.0, 2P), 8.6 (t, $^2J_{PH}$ 13.2, 1P)
MS(+): 362 (362, $[M+2Na]^{2+}$), 678 (678, $[M+H]^+$), 700 (700, $[M+Na]^+$)
MS(–): 338 (338, $[M–2H]^{2–}$), 676 (676, $[M–H]^–$)
HRMS(+) (found (*calc*)): 700.2671 (*700.2652*, $C_{29}H_{50}N_3O_9P_3Na$)
TLC (conc. aq. NH_3 : MeOH = 1:{*x*}): 0.70 {1}, 0.47 {2}, 0.27 {5}

Hexylenetriamine-N,N',N''-*tris(methylphosphonic acid)* ***35b***.

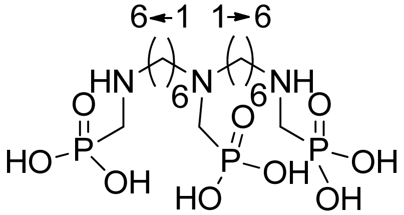

Procedure **C**.

From 135 mg (0.20 mmol) of **35a**.Viscous oil (85 mg, 85 %).

^{1}H NMR (D_2O + *t*BuOH, pD = 0.5 + 0.4): 1.36–1.51 (**3** + **4**, m, 8H), 1.67–1.83 (**2** + **5**, m, 8H), 3.13–3.21 (**6**, m, 4H), 3.21 (2× P–C$\underline{H}_2$–N, d, $^2J_{HP}$ 13.1, 4H), 3.25–3.42 (**1**, m, 4H), 3.35 (1× P–C$\underline{H}_2$–N, d, $^2J_{HP}$ 13.0, 2H)
^{13}C{^{1}H} NMR (D_2O + *t*BuOH, pD = 0.5 + 0.4): 23.5 (**2**), 25.7 (**5**), 2× 25.8 (**3** + **4**), 44.4 (2× P–$\underline{C}H_2$–N, d, $^1J_{CP}$ 139.6), 49.8 (**6**, d, $^4J_{CP}$ 6.8), 49.9 (1× P–$\underline{C}H_2$–N, d, $^1J_{CP}$ 137.7), 55.4 (**1**, d, $^4J_{CP}$ 4.3)
^{31}P NMR (D_2O + *t*BuOH / 85% aq H_3PO_4, pD = 0.5 + 0.4): 8.6 (t, $^2J_{PH}$ 13.0, 1P), 10.2 (t, $^2J_{PH}$ 12.8, 2P)
MS(+): 498 (498, $[M+H]^+$), 520 (520, $[M+Na]^+$)
MS(–): 496 (496, $[M–H]^–$), 993 (993, $[2M–H]^–$)
HRMS(+) (found (*calc*)): 520.1711 (*520.1719*, $C_{15}H_{38}N_3O_9P_3Na$)
TLC (conc. aq. NH_3 : MeOH = 1:{*x*}): 0.74 {1}, 0.11 {2}, 0.03 {5}

Reaction of cyclic polyamines

*Piperazine-*N,N′*-bis(methyl-*H*-phosphinic acid)* ***16a****.*

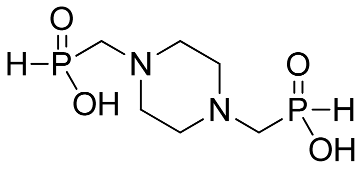

In 25-ml flask, piperazine hexahydrate (194 mg, 1.0 mmol, 1 equiv.), paraformaldehyde (60 mg, 2.0 mmol, 2 equiv.), and H_3PO_2 (as 50% aq. solution, 145 mg, 1.1 mmol, 1.1 equiv.) were mixed with glacial AcOH (10 ml). The suspension was heated at 40 °C for 1 day and conversion was determined by ^{31}P NMR. Then, solvents were removed on rotary evaporator and the oily residue was purified on strong anion exchanger (Dowex 1, 3×10-cm bed) in OH^--form. The column was washed with water and product was eluted off with 20% aq. AcOH. Eluate was concentrated *in vacuo* and the oily residue was re-purified on strong anion exchanger (Dowex 1, 3×10-cm bed) but in AcO^--form. The column was first washed with water which separated *N*-methylated mono substituted derivative **16**, and product was eluted off with 20% aq. AcOH. The eluate was concentrated *in vacuo* and the oily residue was triturated in EtOH using ultrasound. Solidified product was filtered off and washed with Et_2O (2× 5 ml). White powder, **16a**·$2H_2O$ (103 mg, 37 %).

^{1}H NMR (D_2O + *t*BuOH, pD = 1.7 + 0.4): 3.36 (P–C<u>H</u>$_2$–N, dd, $^2J_{HP}$ 10.5, $^3J_{HH}$ 1.8, 4H), 3.80 (N–C<u>H</u>$_2$–C<u>H</u>$_2$–N, s, 8H), 7.25 (<u>H</u>–P, dt, $^1J_{HP}$ 550.9, $^3J_{HH}$ 1.7, 2H)
^{13}C{^{1}H} NMR (D_2O + *t*BuOH, pD = 1.7 + 0.4): 51.4 (N–<u>C</u>H$_2$–<u>C</u>H$_2$–N, d, $^3J_{CP}$ 5.3), 56.3 (P–<u>C</u>H$_2$–N, d, $^1J_{CP}$ 83.2)
^{31}P NMR (D_2O + *t*BuOH / 85% aq H_3PO_4, pD = 1.7 + 0.4): 9.2 (dt, $^1J_{PH}$ 550.6, $^2J_{PH}$ 10.6)
MS(+): 265 (265, $[M+Na]^+$), 281 (281, $[M+K]^+$), 485 (485, $[2M+H]^+$)
MS(–): 241 (241, $[M-H]^-$), 483 (483, $[2M-H]^-$)
HRMS(+) (found (*calc*)): 243.0633 (*243.0664*, $C_6H_{17}N_2O_4P_2$), 485.1202 (*485.1249*, $C_{12}H_{33}N_4O_8P_4$)
TLC (conc. aq. NH_3 : EtOH = 1:{*x*}): 0.50 {5}, 0.23 {10}, 0.17 {20}, 0.12 {35}
EA(found (*calc* M · $2H_2O$)): C 25.95 (*25.91*), H 6.87 (*7.25*), N 9.91 (*10.07*), P 21.16 (*22.27*)

*1,4,7-Triazacyclononane-1,4,7-tris(methyl-*H*-phosphinic acid)* ***36***.

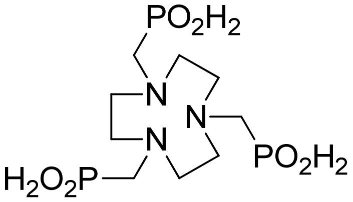

In 50-ml flask, 1,4,7-triazacyclononane (tacn; 129 mg, 1.0 mmol, 1 equiv.), paraformaldehyde (150 mg, 5.0 mmol, 5 equiv.) and H_3PO_2 (as 50% aq. solution, 660 mg, 5.0 mmol, 5 equiv.) was mixed in AcOH (~10 ml) and stirred for 1 day. Then, conversion was determined by ^{31}P NMR and the solution was concentrated *in vacuo*. The oily residue was purified on strong cation exchanger (Dowex 50, 3×5-ml bed) and product was eluted off with water. Solvents were removed *in vacuo* and the oily residue was re-purified on strong cation exchanger (Dowex 50, 3×10-ml bed). Product was eluted with water after a delay (~5-ml fractions). Fractions containing pure products were combined and evaporated to dryness to get pure product. Product solidified in its EtOH solution (~5 ml) by adding excess of Me_2CO (~15 ml) and using ultrasound. Solids were filtered off and washed with acetone (5 ml), Et_2O (2× 5ml) and dried in oven (15 min, 75 °C). White powder, **36**·3/2H_2O (183 mg, 47 %).

^{1}H NMR (D_2O + *t*BuOH, pD = 0.5 + 0.4): 3.36 (P–C$\underline{H}_2$–N, d, $^2J_{HP}$ 8.8, 6H), 3.59 (N–C$\underline{H}_2$–C$\underline{H}_2$–N, s, 12H), 7.29 ($\underline{H}$–P, dt, $^1J_{HP}$ 547.2, $^3J_{HH}$ 1.4, 3H)
^{13}C{^{1}H} NMR (D_2O + *t*BuOH, pD = 0.5 + 0.4): 52.4 (N–$\underline{C}H_2$–$\underline{C}H_2$–N, d, $^3J_{CP}$ 4.9), 56.6 (P–$\underline{C}H_2$–N, d, $^1J_{CP}$ 89.5)
^{31}P NMR (D_2O + *t*BuOH / 85% aq H_3PO_4, pD = 0.5 + 0.4): 16.4 (dt, $^1J_{HP}$ 547.2, $^2J_{PH}$ 8.7)
MS(+): 364 (364, $[M+H]^+$), 727 (727, $[2M+H]^+$)
MS(–): 362 (362, $[M-H]^-$), 725 (725, $[2M-H]^-$)
HRMS(+) (found (*calc*)): 364.0948 (*364.0951*, $C_9H_{25}N_3O_6P_3$), 727.1797 (*727.1829*, $C_{18}H_{49}N_6O_{12}P_6$)
TLC (conc. aq. NH_3 : EtOH = 1:{*x*}): 0.56 {1.5}, 0.47 {2}, 0.26 {5}, 0.11 {10}
EA(found (*calc* M · 3/2H_2O)): C 28.07 (*27.70*), H 6.09 (*6.97*), N 10.70 (*10.77*), P 23.56 (*23.81*)

*1,4,7,10-Tetraazacyclododecane-1,7-bis(methyl-*H*-phosphinic acid)* ***37*** and
*1,4,7,10-Tetraazacyclododecane-7-methyl-1-(methyl-*H*-phosphinic acid)* ***37-Me***.

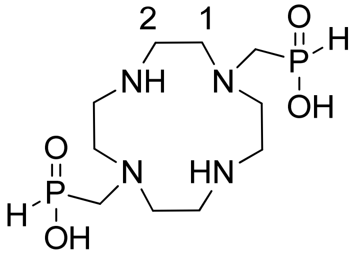

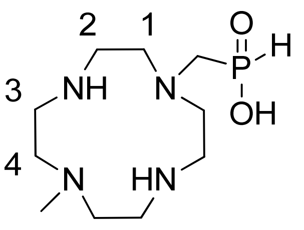

Firstly, *trans*-Cbz_2cyclen dihydrochloride (0.52 g, 1.0 mmol) was transferred into its "free-base form" by washing of its CH_2Cl_2 solution (20 ml) with 5% aq. NaOH (3×5 ml). Organic phase was dried with anhydrous sodium sulfate and evaporated to dryness. In 25-ml flask, the oily residue of *trans*-Cbz_2cyclen (1.0 mmol, 1 equiv.), paraformaldehyde (90 mg, 3.0 mmol, 3 equiv.) and H_3PO_2 (as 50% aq. solution, 396 mg, 3.0 mmol, 3 equiv.) were mixed with glacial

AcOH (20 ml). The suspension was stirred room temperature for 3 days. Conversion was not determined by ^{31}P NMR because product the signals were too broad. Then, solvents were removed on rotary evaporator and the residue was co-evaporated with toluene (2× 5 ml) and once with water (5 ml). The oily residue was further purified by silica column chromatography (C18, gradient from water to ACN:water:TFA ~9:1:0.01). Fractions containing pure products were combined and evaporated to dryness to get two oils, the protected precursors for **37** and **37-Me**, respectively. Each oily residue was dissolved in 1:1 aq. HCl (~10 ml) and heated at 100 °C for 2 days. Then, solvents were evaporated and each oily residue was purified on strong cation exchanger (Dowex 50, 3×10-cm bed). The column was firstly washed with water and then (*i*) product **37** was eluted off with 10% aq. pyridine and (*ii*) product **37-Me** was eluted off with 5% aq. NH_3. The each eluate was evaporated to dryness *in vacuo*. The residue containing **37** was dissolved in EtOH and left to crystallize in fridge for 1 day. The polycrystalline powder was filtered off, washed with acetone (5 ml) and with Et_2O (2× 5 ml) to get pure product. White polycrystalline powder **37**·$4H_2O$ (16 mg, 4 %). The oily residue containing **37-Me** was further purified on strong anion exchanger (Dowex 1, 3×5-cm bed). The column was washed with water and the product was eluted off with 20 % aq. AcOH. Solvents were evaporated *in vacuo*. The oily residue was loaded on strong cation exchanger (Dowex 50, 3×5-cm bed) in the **pyridine form**. After washing the column with water, product was eluted off with 5% aq. NH_3. Solvents were removed *in vacuo* and the pure product **37-Me** was obtained as viscous oil (40 mg, 15 %).

Characterization for compound **37**:

^{1}H NMR (D_2O + *t*BuOH, pD = 3.5 + 0.4): 2.83 (H–P–C<u>H</u>$_2$–N, dd, $^2J_{HP}$ 7.3, $^3J_{HH}$ 1.8, 4H), 2.96–3.17 (**1**, m, 8H), 3.17–3.40 (**2**, m, 8H), 7.15 (<u>H</u>–P, dt, $^1J_{HP}$ 509.6, $^3J_{HH}$ 1.6, 2H)
^{13}C{^{1}H} NMR (D_2O + *t*BuOH, pD = 3.5 + 0.4): 43.6 (**2**), 50.6 (**1**, d, $^3J_{CP}$ 6.0), 54.8 (N–<u>C</u>H$_2$–P, d, $^1J_{CP}$ 98.3)
^{31}P NMR (D_2O + *t*BuOH / 85% aq H_3PO_4, pD = 3.5 + 0.4): 21.1 (H–<u>P</u>, dt, $^1J_{PH}$ 509.6, $^2J_{PH}$ 7.4)
MS(+): 329 (329, $[M+H]^+$), 351 (351, $[M+Na]^+$), 679 (679, $[2M+Na]^+$)
MS(–): 327 (327, $[M-H]^-$), 655 (655, $[2M-H]^-$)
HRMS(+) (found (*calc*)): 329.1514 (*329.1502*, $C_{10}H_{27}N_4O_4P_2$)
TLC (conc. aq. NH_3 : EtOH = 1:{*x*}): 0.23 {1}, 0.18 {1.5}, 0.13 {5}
EA (found (*calc* M · $4H_2O$)): C 29.97 (*30.00*), H 8.07 (*8.56*), N 13.63 (*13.99*)

Characterization for compound **37-Me**:

^{1}H NMR (D_2O + *t*BuOH, pD = 9.7 + 0.4): 2.75 (H–P–C<u>H</u>$_2$–N, dd, $^2J_{HP}$ 7.8, $^3J_{HH}$ 1.6, 2H), 2.71–2.77 (**4**, m, 4H), 2.91–2.97 (**1**, m, 4H), 3.03–3.10 (**2** + **3**, m, 8H), 7.04 (<u>H</u>–P, dt, $^1J_{HP}$ 507.3, $^3J_{HH}$ 1.6, 1H)
^{13}C{^{1}H} NMR (D_2O + *t*BuOH, pD = 9.7 + 0.4): 42.9 (N–<u>C</u>H$_3$), 43.7 (**2**), 43.9 (**3**), 52.0 (**1**, d, $^3J_{CP}$ 6.5), 52.2 (**4**), 55.3 (N–<u>C</u>H$_2$–P, d, $^1J_{CP}$ 105.4)
^{31}P NMR (D_2O + *t*BuOH / 85% aq H_3PO_4, pD = 9.7 + 0.4): 22.8 (H–<u>P</u>, dt, $^1J_{PH}$ 507.3, $^2J_{PH}$ 7.9)
MS(+): 265 (265, $[M+H]^+$), 529 (529, $[2M+H]^+$)
MS(–): 263 (263, $[M-H]^-$), 527 (527, $[2M-H]^-$)
HRMS(+) (found (*calc*)): 265.1794 (*265.1788*, $C_{10}H_{26}N_4O_2P$)
TLC (conc. aq. NH_3 : EtOH = 1:{*x*}): 0.74 {1}, 0.68 {1.5}, 0.51 {2}, 0.32 {5}

*4,7,10-Trimethyl-1,4,7,10-tetraazacyclododecane-1-methyl-*H*-phosphinic acid* ***38-Me***

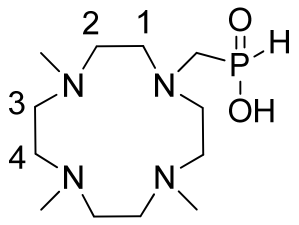

<u>Procedure **B**.</u>

From 346 mg (1.0 mmol) of 1,7-Me_2cyclen·4HCl which was used in its „free-base form" after washing its CH_2Cl_2 solution with 5% aq. NaOH thrice and solvent evaporation *in vacuo*. Product **38-Me** was isolated as viscous oil (44 mg, 15 %).

^{1}H NMR (D_2O + *t*BuOH, pD = 10.2 + 0.4): 2.40 (N–C<u>H</u>$_3$, s, 3H), 2.69 (2× N–C<u>H</u>$_3$, s, 6H), 2.80 (H–P–C<u>H</u>$_2$–N, dd, $^{2}J_{HP}$ 6.2, $^{3}J_{HH}$ 1.5, 2H), 2.75–2.80 (**4**, m, 4H), 2.89–2.94 (**1**, m, 4H), 2.96–3.03 (**2** + **3**, m, 8H), 7.11 (<u>H</u>–P, dt, $^{1}J_{HP}$ 506.0, $^{3}J_{HH}$ 1.3, 1H)
^{13}C{^{1}H} NMR (D_2O + *t*BuOH, pD = 10.2 + 0.4): 42.8 (2× N–<u>C</u>H$_3$), 43.3 (N–<u>C</u>H$_3$), 51.4 (**1**, d, $^{3}J_{CP}$ 6.7), 51.6 (**4**), 54.8 (**3**), 55.2 (**2**), 56.8 (H–P–<u>C</u>H$_2$–N, d, $^{1}J_{CP}$ 103.0)
^{31}P NMR (D_2O + *t*BuOH / 85% aq H_3PO_4, pD = 10.2 + 0.4): 22.4 (H–<u>P</u>, dt, $^{1}J_{PH}$ 506.4, $^{2}J_{PH}$ 6.4)
MS(+): 293 (293, $[M+H]^+$), 315 (315, $[M+Na]^+$), 585 (585, $[2M+H]^+$), 607 (607, $[2M+Na]^+$), 629 (629, $[2M+2Na–H]^+$)
MS(–): 291 (291, $[M–H]^-$), 583 (583, $[2M–H]^-$)
HRMS(+) (found (*calc*)): 293.2106 (*293.2101*, $C_{12}H_{30}N_4O_2P$)
TLC (conc. aq. NH_3 : EtOH = 1:{*x*}): 0.71 {5}, 0.27 {10}, 0.15 {20}, 0.09 {35}

Additional compounds

*Bis[(*N*-methylphosphonic acid)-aminomethyl]phosphinic acid* ***28c****.*

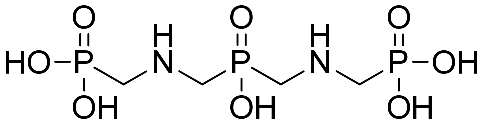

In 25-ml flask, **28b** (20 mg, 0.04 mmol) was dissolved in 90% aq. AcOH and Pd/C (2 mg, 10% w/w) was added. Flask was flushed with hydrogen. Suspension was vigorously stirred at room temperature for 2 days under hydrogen atmosphere from balloon. Then, suspension was filtered through 0.22 μm PVDF microfilter and the filtrate was concentrated *in vacuo*. The oily residue was co-evaporated with toluene (2×5 ml) to remove acetic acid and triturated with EtOH (3 ml) using ultrasound. The solid material was filtered off, washed with acetone (2 ml) and with Et_2O (2×3 ml). White powder, **28c**·3/2H_2O (13 mg, 98 %).

^{1}H NMR (D_2O + *t*BuOH, pD = 2.0 + 0.4): 3.33 (HO–P–C$\underline{H}_2$–N, d, $^2J_{HP}$ 12.4, 4H), 3.49 (C$\underline{H}_2$–P–C$\underline{H}_2$, d, $^2J_{HP}$ 9.8, 4H)
^{13}C{^{1}H} NMR (D_2O + *t*BuOH, pD = 2.0 + 0.4): 46.5 (HO–P–$\underline{C}H_2$–N, dd, $^1J_{CP}$ 136.7, $^3J_{CP}$ 4.7), 48.0 ($\underline{C}H_2$–P–$\underline{C}H_2$, dd, $^1J_{CP}$ 99.1, $^3J_{CP}$ 5.7)
^{31}P NMR (D_2O + *t*BuOH / 85% aq H_3PO_4, pD = 2.0 + 0.4): 8.4–10.2 (HO–$\underline{P}$, m, 2P), 16.7–17.8 (CH_2–$\underline{P}$–CH_2, m, 1P)
MS(+): 335 (335, $[M+Na]^+$)
MS(–): 311 (311, $[M–H]^-$), 623 (623, $[2M–H]^-$), 644 (644, $[2M–2H+Na]^-$)
HRMS(–) (found (*calc*)): 310.9970 (*310.9968*, $C_4H_{14}N_2O_8P_3$)
TLC (conc. aq. NH_3 : MeOH = 1:{*x*}): 0.74 {1}, 0.11 {2}, 0.03 {5}
EA(found (*calc* M · 3/2H_2O)): C 14.05 (*14.17*), H 5.63 (*5.35*), N 7.77 (*8.26*)

*Imino-bis(methyl-*H*-phosphinic acid)* ***25a****.*

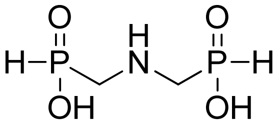

In 100-ml flask, **25** (0.92 g, 4.0 mmol) was dissolved TFA (~30 ml) and solution was gently refluxed (oil bath, 80 °C) for 1 day. Then, the solution was concentrated *in vacuo*. The oily residue was co-evaporated with toluene (2×10 ml) to remove trifluoroacetic acid and once with water (~5 ml). The oily residue was triturated with MeOH (~20 ml) using ultrasound. The solids were filtered off, washed with acetone (10 ml) and with Et_2O (2× 10 ml). White powder, **25a**·0.25MeOH (0.55 g, 76 %).

^{1}H NMR (D_2O + *t*BuOH, pD = 1.0 + 0.4): 3.26 (P–C$\underline{H}_2$–N, dd, $^2J_{HP}$ 10.8, $^3J_{HH}$ 1.9, 4H), 7.21 ($\underline{H}$–P, dt, $^1J_{HP}$ 548.5, $^3J_{HH}$ 1.9, 2H)
^{13}C{^{1}H} NMR (D_2O + *t*BuOH, pD = 1.0 + 0.4): 49.0 (P–$\underline{C}H_2$–N, dd, $^1J_{CP}$ 85.8, $^3J_{CP}$ 6.0)
^{31}P NMR (D_2O + *t*BuOH / 85% aq H_3PO_4, pD = 1.0 + 0.4): 12.0 (dt, $^1J_{HP}$ 548.6, $^2J_{PH}$ 10.7)
MS(+): 174 (174, $[M+H]^+$), 347 (347, $[2M+H]^+$)
MS(–): 172 (172, $[M–H]^-$), 345 (345, $[2M–H]^-$)
HRMS(+) (found (*calc*)): 174.0089 (*174.0080*, $C_2H_{10}NO_4P_2$), 347.0104 (*347.0086*, $C_4H_{19}N_2O_8P_4$)
TLC (conc. aq. NH_3 : EtOH = 1:{*x*}): 0.75 {1}, 0.39 {5}, 0.28 {10}
EA(found (*calc* M · 1/4MeOH)): C 14.95 (*14.93*), H 5.10 (*5.57*), N 7.69 (*7.74*), P 32.91 (*34.21*)

*{1-[(*N,N*-Dibenzyl)-amino]butyl}[(2-*t*-butoxycarbonyl)ethyl]phosphinic acid* ***19a***.

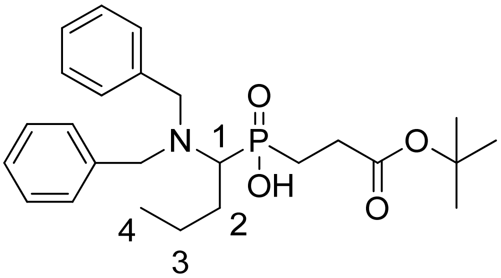

In 50-ml three-neck flask, *H*-phosphinic acid **19** (254 mg, 0.8 mmol, 1 equiv.) was dissolved in dry CH_2Cl_2 (~10 ml) under argon atmosphere. Next, dry Et_3N (555 µl, 4.0 mmol, 5 equiv.) was added followed by Me_3SiCl (202 µl, 1.6 mmol, 2 equiv.) and *N,O*-bis(trimethylsilyl)acetamide (590 µl, 2.4 mmol, 3 equiv.). The mixture was stirred at room temperature under argon atmosphere for 1 day. The complete conversion to P(III)N intermediate was checked by ^{31}P NMR and, then, *t*-butyl acrylate (130 µl, 0.9 mmol, 1.1 equiv.) was added. The mixture was stirred under argon atmosphere for 1 day. Then, EtOH (~0.5 ml, excess) was slowly added and, after 1 h, the mixture was concentrated *in vacuo*. The oily residue was dissolved in MeOH (~2 ml) and purified by C18 silica column chromatography (elution with gradient of pure water to ACN:water:TFA = 9:1:0.01). Fractions containing pure product were combined and evaporated to dryness. Viscous oil (160 mg, 45 %).

^{1}H NMR ($CDCl_3$): 0.97 (**4**, t, $^3J_{HH}$ 7.2, 3H), 1.26–1.50 (**3**, m, 1H), 1.40 (($C\underline{H}_3)_3$–C, s, 9H), 1.55–1.69 (**3**, m, 1H), 1.69–1.85 (**2** + (P–$C\underline{H}_2$–CH_2–CO), m, 2H), 1.85–1.97 (P–$C\underline{H}_2$–CH_2–CO, m, 1H), 2.04–2.25 (**2** + (P–CH_2–$C\underline{H}_2$–CO), m, 2H), 2.25–2.41 (P–CH_2–$C\underline{H}_2$–CO, m, 1H), 3.08 (**1**, ddd, $^2J_{HP}$ 10.1, $^3J_{HH}$ 8.1, $^3J_{HH}$ 4.5, 1H), 3.81–3.91 and 4.46–4.66 (N–$C\underline{H}_2$–Ph, m, 4H), 7.29–7.52 (Ph, m, 10H)
^{13}C{^{1}H} NMR ($CDCl_3$): 14.1 (**4**), 20.7 (**3**, d, $^3J_{CP}$ 3.2), 25.6 (**2**), 25.8 (P–$\underline{C}H_2$–CH_2–CO, d, $^1J_{CP}$ 98.2), 27.5 (P–CH_2–$\underline{C}H_2$–CO, d, $^2J_{CP}$ 3.5), 28.0 (($\underline{C}H_3)_3$–C), 56.1 (N–$\underline{C}H_2$–Ph, d, $^3J_{CP}$ 3.5), 58.6 (**1**, d, $^1J_{CP}$ 85.9), 80.8 (($CH_3)_3$–$\underline{C}$), 129.5 (*m*-Ph), 129.9 (*p*-Ph), 130.0 (*o*-Ph), 131.0 (*i*-Ph), 171.9 (CH_2–$\underline{C}$O–O*t*Bu, d, $^3J_{CP}$ 16.6)
^{31}P NMR ($CDCl_3$ / 85% aq H_3PO_4): 34.8–37.5 (m)
MS(+): 446 (446, $[M+H]^+$), 484 (484, $[M+K]^+$)
MS(–): 444 (444, $[M–H]^-$)
HRMS(+) (found (*calc*)): 446.2465 (*446.2455*, $C_{25}H_{37}NO_4P$)
TLC: 0.75 (*i*PrOH:conc. aq. NH_3:H_2O = 10:1:2), 0.60 (MeOH:*i*PrOH = 1:1), 0.58 (EtOH)

*(1-Aminobutyl)-[(2-*t*-butoxycarbonyl)ethyl]phosphinic acid* ***19b***.

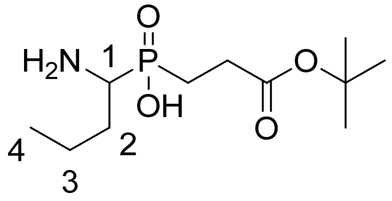

In 50-ml flask, phosphinic acid **19a** (147 mg, 0.33 mmol, 1 equiv.) and Pd/C (15 mg, 10% w/w) was suspended in MeOH (~10 ml) and flushed with hydrogen. Mixture was stirred at room temperature under hydrogen atmosphere from balloon for 1 day. Then, solids were filtered off using 0.22 µm PVDF filter, solvents were evaporated *in vacuo* and once co-evaporated with Et_2O (~5 ml) to obtain pure product. Viscous oil (88 mg, 100 %).

^{1}H NMR ($CDCl_3$): 0.98 (**4**, t, $^3J_{HH}$ 7.2, 3H), 1.43 (($C\underline{H}_3)_3$–C, s, 9H), 1.45–1.57 (**3**, m, 1H), 1.62–1.73 (**3**, m, 1H), 1.73–1.91 (**2**, m, 2H), 1.93–2.13 (P–$C\underline{H}_2$–CH_2–CO, m, 1H), 2.52 (P–CH_2–$C\underline{H}_2$–CO, dt, $^3J_{HP}$ 10.8, $^3J_{HH}$ 8.0), 3.54–3.67 (**1**, m, 1H), 8.28 ($\underline{H}_3N^+$–CH_2–P, bs, 3H)
^{13}C{^{1}H} NMR ($CDCl_3$): 13.7 (**4**), 19.8 (**3**, d, $^3J_{CP}$ 8.9), 23.0 (P–$\underline{C}H_2$–CH_2–CO, $^1J_{CP}$ 99.8), 27.3 (P–CH_2–$\underline{C}H_2$–CO, d, $^2J_{CP}$ 3.9), 28.0 (($\underline{C}H_3)_3$–C), 30.7 (**2**), 49.8 (**1**, d, $^1J_{CP}$ 90.4), 81.1 (($CH_3)_3$–$\underline{C}$)
^{31}P NMR ($CDCl_3$ / 85% aq H_3PO_4): 38.9–41.2 (m)
MS(+): 266 (266, $[M+H]^+$), 531 (531, $[2M+H]^+$), 796 (796, $[3M+H]^+$)
MS(–): 264 (264, $[M–H]^-$), 529 (529, $[M–H]^-$)
HRMS(+) (found (*calc*)): 266.1543 (*266.1516*, $C_{11}H_{25}NO_4P$), 531.2969 (*531.2959*, $C_{22}H_{49}N_2O_8P_2$)
TLC: 0.60 (*i*PrOH:conc. aq. NH_3:H_2O = 10:1:2), 0.23 (MeOH:*i*PrOH = 1:1), 0.18 (EtOH)

(N,N-Dibenzyl)-aminomethylphosphonic acid ***A***.

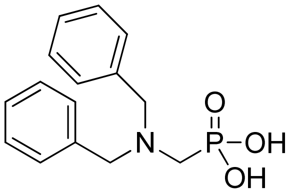

In 4-ml vial, *N,N*-dibenzyl-amine (192 µl, 1.0 mmol, 1 equiv.), paraformaldehyde (60 mg, 2.0 mmol, 2 equiv.) and H_3PO_3 (90 mg, 1.1 mmol, 1.1 equiv.) were mixed with glacial AcOH (2 ml). The suspension was heated at 40 °C for 36 h followed by heating at 60 °C for 2 days. Conversion was determined by ^{31}P NMR. Then, solvents were removed on rotary evaporator and the oily residue was purified on strong cation exchanger (Dowex 50, 3×10-cm bed). The column was washed with water and product was eluted off with 10% aq. pyridine. The pyridine eluate was evaporated to dryness. The solid residue was triturated in acetone using ultrasound, filtered off, washed with acetone (5 ml) and with Et_2O (2×5 ml) to get pure product. White powder **A**·4/3H_2O (76 mg, 24 %).[10]
Characterization data were the same as published.[11]

(N,N-Dicyclohexyl)-aminomethylphosphonic acid ***B***.

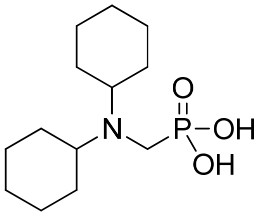

In 4-ml vial, *N,N*-dicyclohexyl-amine (201 µl, 181 mg, 1.0 mmol, 1 equiv.), paraformaldehyde (60 mg, 2.0 mmol, 2 equiv.), and H_3PO_3 (90 mg, 1.1 mmol, 1.1 equiv.) were mixed with glacial AcOH (2 ml). The suspension was heated at 40 °C for 2 days, and to 60 °C for 3 days during which time conversion was followed by ^{31}P NMR. Then, solvents were removed on rotary evaporator and the oily residue was purified on strong cation exchanger (Dowex 50, 3×10 cm bed). The column was washed with water and product was eluted off with 10% aq. pyridine. The pyridine eluate was evaporated to dryness. The solid residue was triturated in acetone using ultrasound, filtered off, washed with acetone (5 ml) and with Et_2O (2× 5 ml) to get pure product. Off-white powder **B**·H_2O (75 mg, 27 %).

^{1}H NMR (D_2O + *t*BuOH, pD = 5.4 + 0.4): 1.12–1.25 (**4**, m, 2H), 1.27–1.53 (**3**, m, 4H), 1.50–1.64 (**2**, m, 4H), 1.63–1.72 (**4**, m, 2H), 1.84–1.96 (**3**, m, 4H), 2.00–2.12 (**2**, m, 4H), 3.34 (P–C<u>H</u>$_2$–N, d, $^2J_{HP}$ 13.5, 2H), 3.51–3.61 (**1**, m, 2H)
^{13}C{^{1}H} NMR (D_2O + *t*BuOH, pD = 5.4 + 0.4): 25.1 (**4**), 25.1 + 25.4 (**3**), 27.9 + 29.0 (**2**), 45.7 (P–<u>C</u>H_2–N, d, $^1J_{CP}$ 135.9), 64.8 (**1**, d, $^3J_{CP}$ 3.4)
^{31}P NMR (D_2O + *t*BuOH / 85% aq H_3PO_4, pD = 5.4 + 0.4): 9.9 (t, $^2J_{PH}$ 13.4)
MS(+): 314 (314, $[M+Na]^+$), 605 (605, $[2M+Na]^+$)
MS(–): 290 (290, $[M–H]^–$), 581 (581, $[2M–H]^–$)
HRMS(+) (found (*calc*)): 276.1728 (*276.7123*, $C_{13}H_{27}NO_3P$)
TLC (conc. aq. NH_3 : EtOH = 1:{*x*}): 0.74 {1}, 0.61 {1.5}
EA (found (*calc* M · H_2O)): C 53.14 (*53.60*), H 8.68 (*9.00*), N 4.62 (*4.81*), P 9.68 (*10.63*)

---

[10]**EA (found (*calc* M · 4/3H_2O)):** C 57.05 (*57.41*), H 6.05 (*6.58*), N 4.50 (*4.46*), P 9.87 (*9.92*)
[11] W. Szczepaniak and K. Kuczynski, *Phosporus Sulfur Relat. Elem.* **1979**, *7*, 333–337.

[(N,N-Dibenzyl)-aminomethyl](phenyl)phosphinic acid **C**.

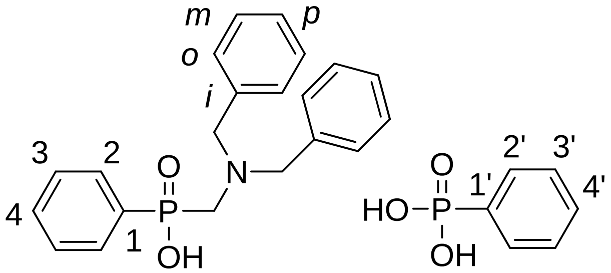

In 4-ml vial, *N*,*N*-dibenzyl-amine (106 µl, 0.55 mmol, 1.1 equiv.), paraformaldehyde (30 mg, 1.0 mmol, 2 equiv.), and phenyl-*H*-phosphinic acid (71 mg, 0.5 mmol, 1 equiv.) were mixed with glacial AcOH (2 ml). The suspension was heated at 40 °C for 1 day and conversion was followed by ^{31}P NMR. Then, solvents were removed on rotary evaporator. The oily residue was dissolved in EtOH and purified on strong cation exchanger (Dowex 50, 3×10-cm bed). Column was washed with aq. EtOH (1:1, ~100 ml) and product was eluted off with 10% pyridine in water : EtOH (~3:1) mixture. Pyridine eluate was evaporated to dryness and the oily residue was dissolved in water (~2 ml) and left to crystallize in fridge. After standing for 3 days, crystalline product was isolated (7 mg, 4 %). It was identified as a adduct of **C** with phenylphosphonic acid. A single crystal was taken from the bulk.

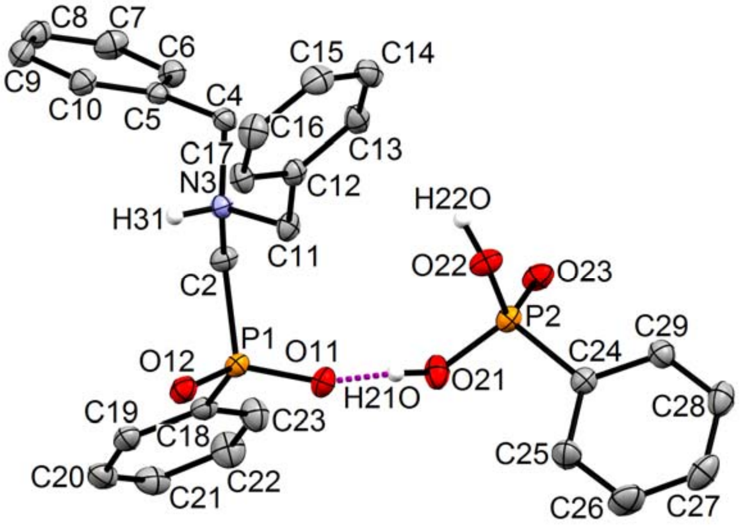

^{1}H NMR (DMSO-d_6): 2.77 (P–C<u>H</u>$_2$–N, d, $^2J_{HP}$ 10.0, 2H), 3.69 (N–C<u>H</u>$_2$–Ph, s, 4H), 7.06–7.15 (*o*-Ph, m, 4H), 7.15–7.26 (*m*-Ph + *p*-Ph, m, 6H), 7.40–7.54 (**2'** + **4'** + **2**, m, 5H), 7.54–7.63 (**3** + **4**, m, 3H), 7.63–7.73 (**3'**, m, 2H)

^{13}C{^{1}H} NMR (DMSO-d_6): 52.3 (P–<u>C</u>H$_2$–N, d, $^1J_{CP}$ 115.6), 58.5 (N–<u>C</u>H$_2$–Ph, d, $^3J_{CP}$ 8.1), 126.9 (*p*-Ph), 128.0 (**4'**, d, $^4J_{CP}$ 2.4), 128.1 (*o*-Ph), 128.2 (**4**), 128.6 (*m*-Ph), 130.5 (**3'**, d, $^3J_{CP}$ 9.7), 130.8 (**2'**, d, $^2J_{CP}$ 3.1), 131.3 (**3**, d, $^3J_{CP}$ 9.4), 131.5 (**2'**, d, $^2J_{CP}$ 2.9), 133.7 (**1'**, d, $^1J_{CP}$ 125.6), 134.7 (**1**, d, $^1J_{CP}$ 73.4), 138.2 (*i*-Ph)

^{31}P NMR (DMSO-d_6 / 85% H_3PO_4): 13.8 (HO–<u>P</u>–OH, m, 1P), 33.2 (C–<u>P</u>–C, m, 1P)

MS(+): 352 (352, [M+H]$^+$), 374 (374, [M+Na]$^+$)

MS(–): 350 (350, [M–H]$^-$)

HRMS(+) (found (*calc*)): 352.1467 (*352.1461*, $C_{21}H_{23}NO_2P$)

TLC: 0.84 (*i*PrOH:conc. aq. NH$_3$:water = 10:1:2), 0.52 (EtOH), 0.72 (MeOH:*i*PrOH = 1:1)

*(Phthalimido-methyl)[(*N,N*-dibenzyl)-aminomethyl]phosphinic acid* ***D****.*

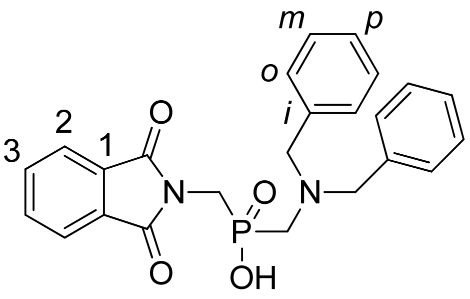

In 4-ml vial, *N*,*N*-dibenzylamine (106 µl, 0.55 mmol, 1.1 equiv.), paraformaldehyde (30 mg, 1.0 mmol, 2 equiv.), and (phtalimidomethyl)phosphinic acid (113 mg, 0.5 mmol, 1 equiv.) were mixed with glacial AcOH (2 ml). The suspension was heated up to 40 °C for 1 day and conversion was determined by ^{31}P NMR. Then, solvents were removed on rotary evaporator and water (~10 ml) was added to oily residue. Heterogenous mixture was triturated using ultrasound. Solid was filtered off and washed with water (2 ml), with Et_2O (2× 5 ml) and dried in oven (100 °C / 15 min). White powder, **D**·7/3H_2O (119 mg, 50 %).

A single crystal was prepared by slow cooling of hot aqueous solution of **D**.

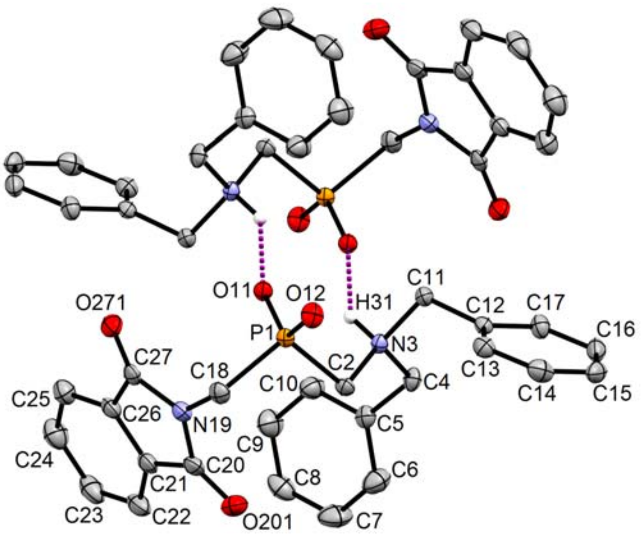

^{1}H NMR (DMSO-d_6): 2.81 (P–C<u>H</u>$_2$–N–Bn, d, $^{2}J_{HP}$ 8.3, 2H), 3.78 (PhtN–C<u>H</u>$_2$–P, d, $^{2}J_{HP}$ 7.6, 2H), 3.92 (N–C<u>H</u>$_2$–Ph, s, 4H), 7.22–7.52 (Ph, m, 10H), 7.78–7.93 (Phth, m, 4H)

^{13}C{^{1}H} NMR (DMSO-d_6): 37.2 (PhtN–<u>C</u>H$_2$–P, d, $^{1}J_{CP}$ 96.4), 51.4 (P–<u>C</u>H$_2$–N–Bn, d, $^{1}J_{CP}$ 100.8), 57.9 (N–<u>C</u>H$_2$–Ph, d, $^{3}J_{CP}$ 6.4), 123.1 (**2**), 127.8 (*p*-Ph), 128.4 (*m*-Ph), 129.8 (*o*-Ph), 131.6 (**1**), 134.5 (**3**), 135.8 (*i*-Ph), 167.2 (N–<u>C</u>=O)

^{31}P NMR (DMSO-d_6): 30.3–31.2 (m)

MS(+): 457 (457, $[M+Na]^{+}$)

MS(–): 433 (433, $[M–H]^{-}$)

HRMS(+) (found (*calc*)): 435.1477 (*435.1468*, $C_{24}H_{24}N_2O_4P$)

TLC: 0.72 (*i*PrOH:conc. aq. NH_3:water = 10:1:2), 0.45 (EtOH), 0.60 (MeOH:*i*PrOH = 1:1)

EA(found (*calc* M · 7/3H$_2$O)): C 60.48 (*60.50*), H 5.18 (*5.85*), N 5.75 (*5.88*), P 7.53 (*6.50*)

[(N,N-Dicyclohexyl)-aminomethyl][(N′,N′-dibenzyl)-aminomethyl]phosphinic acid **E**.

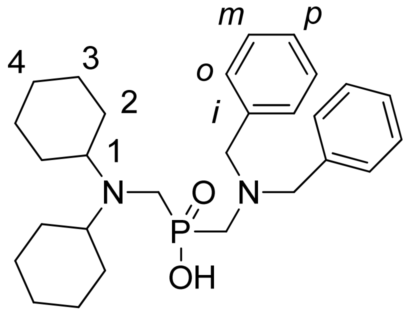

In 25-ml round-bottom flask, *N*,*N*-dibenzyl-amine (212 µl, 1.1 mmol, 1.1 equiv.), paraformaldehyde (60 mg, 2.0 mmol, 2 equiv.), and (*N*,*N*-dicyclohexyl)-aminomethyl-*H*-phosphinic acid **5** (260 mg, 1.0 mmol, 1 equiv.) were mixed with glacial AcOH (10 ml). The suspension was heated at 40 °C for 2 days and conversion was followed by ^{31}P NMR. Then, solvents were removed on rotary evaporator. The oily residue was dissolved aq. MeOH (~75 %, ~3 ml) and purified on flash silica column chromatography (C18, gradient from pure water to ACN:water:TFA = 9:1:0.01). Fractions containing pure product were combined and concentrated *in vacuo* to yield viscous oil of **E**·TFA (230 mg, 40 %).

^{1}H NMR ($CDCl_3$): 1.02–1.17 (**4**, m, 2H), 1.17–1.30 (**3**, m, 4H), 1.34–1.50 (**2**, m, 4H), 1.56–1.69 (**4**, m, 2H), 1.74–1.90 (**3**, m, 4H), 1.91–2.05 (**2**, m, 4H), 2.94 (P–C<u>H</u>$_2$–N–Bn, d, $^{2}J_{HP}$ 10.8, 2H), 3.11 (Cy–N–C<u>H</u>$_2$–P, d, $^{2}J_{HP}$ 8.7, 2H), 3.20–3.30 (**1**, m, 2H), 4.23 (N–C<u>H</u>$_2$–Ph, s, 4H), 7.29–7.41 (*m*-Ph + *p*-Ph, m, 6H), 7.41–7.52 (*o*-Ph, m, 4H)
^{13}C{^{1}H} NMR ($CDCl_3$): 24.7 (**4**), 25.1 (**3**), 27.9 (**2**), 63.9 (**1**, d, $^{3}J_{CP}$ 2.5), 47.0 (Cy–N–<u>C</u>H$_2$–P, d, $^{1}J_{CP}$ 86.2), 50.2 (P–<u>C</u>H$_2$–N–Bn, d, $^{1}J_{CP}$ 105.1), 58.5 (N–<u>C</u>H$_2$–Ph, d, $^{3}J_{CP}$ 5.7), 128.7 (*m*-Ph), 128.8 (*p*-Ph), 131.0 (*o*-Ph), 132.8 (*i*-Ph)
^{31}P NMR ($CDCl_3$): 16.3–19.0 (m)
MS(+): 469 (469, $[M+H]^+$), 491 (491, $[M+Na]^+$)
MS(–): 467 (467, $[M–H]^-$)
HRMS(+) (found (*calc*)): 429.2993 (*469.2984*, $C_{28}H_{42}N_2O_2P$), 937.5868 (*937.5884*, $C_{56}H_{83}N_4O_4P_2$)
TLC: 0.86 (*i*PrOH:conc. aq. NH_3:water = 10:1:2), 0.55 (EtOH), 0.56 (MeOH:*i*PrOH = 1:1)

*[(*N*-Benzyl)-aminomethyl](phthalimido-methyl)phosphinic acid* **D1**.

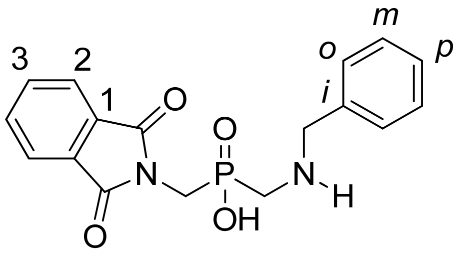

In 25-ml flask, phosphinic acid **D** (240 mg, 0.5 mmol, 1 equiv.) and Pd/C (25 mg, 10% w/w) was suspended in MeOH (~10 ml) and the flask was flushed with hydrogen. The mixture was heated at 50 °C under hydrogen atmosphere from balloon for 1 day. Then, solution was filtered through filtration paper. The filtered-off solid was suspended/dissolved in boiling water (~20 ml) and suspension was filtered. The filtrate was evaporated to dryness *in vacuo*. The solid residue was suspended in acetone (~20 ml) using ultrasound. Pure product was filtered off, washed with acetone (~10 ml), Et_2O (2× 5 ml) and dried in oven (30 min / 90 °C). White powder, **D1**·4/3H_2O (98 mg, 53 %).

^{1}H NMR (CD_3OD + a drop of conc. aq. HCl): 3.55 (P–C<u>H</u>$_2$–N–Bn, d, $^2J_{HP}$ 10.1, 2H), 4.24 (P–C<u>H</u>$_2$–N–Pht, d, $^2J_{HP}$ 9.0, 2H), 4.40 (N–C<u>H</u>$_2$–Ph, s, 2H), 7.44–7.54 (*m*-Ph + *p*-Ph, m, 3H), 7.56–7.63 (*o*-Ph), 7.83–7.90 (**3**, m, 2H), 7.90–7.96 (**2**, m, 2H)
^{13}C{^{1}H} NMR (CD_3OD + a drop of conc. aq. HCl): 38.1 (P–<u>C</u>H$_2$–N–Pht, d, $^1J_{CP}$ 105.4), 45.7 (P–<u>C</u>H$_2$–N–Bn, d, $^1J_{CP}$ 95.5), 54.3 (N–<u>C</u>H$_2$–Ph, d, $^3J_{CP}$ 6.5), 124.5 (**2**), 130.3 (*m*-Ph), 130.9 (*p*-Ph), 131.5 (*o*-Ph), 131.8 (*i*-Ph), 133.2 (**1**), 135.8 (**3**), 169.2 (N–<u>C</u>=O)
^{31}P NMR (CD_3OD + a drop of conc. aq. HCl / 85% aq H_3PO_4): 30.9 (p, $^2J_{PH}$ 8.9, $^2J_{PH}$ 9.9)
MS(+): 345 (345, $[M+H]^+$), 689 (689, $[2M+H]^+$)
MS(–): 343 (343, $[M-H]^-$), 687 (687, $[2M-H]^-$)
HRMS(+) (found (*calc*)): 345.1003 (*345.0999*, $C_{17}H_{18}N_2O_4P$), 689.1914 (*689.1925*, $C_{34}H_{35}N_4O_8P_2$)
TLC: 0.57 (*i*PrOH:conc. aq. NH_3:H_2O = 10:1:2), 0.29 (MeOH:*i*PrOH = 1:1), 0.29 (EtOH)
EA(found (*calc* M · 4/3H_2O)): C 55.78 (*55.44*), H 4.82 (*5.38*), N 7.55 (*7.61*), P 8.61 (*8.41*)

(Aminomethyl)(phthalimido-methyl)phosphinic acid ***D2***.

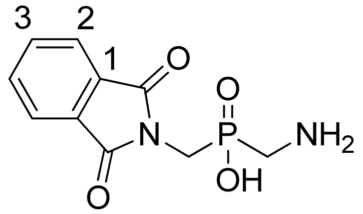

In 25-ml flask, phosphinic acid **D** (240 mg, 0.5 mmol, 1 equiv.) and Pd/C (25 mg, 10% w/w) was suspended in DMF : AcOH ~5:1 (~10 ml) and the flask was flushed with hydrogen. The mixture was heated at 50 °C under hydrogen atmosphere from balloon for 2 days. Then, the suspension was filtered through 0.22 μm PVDF filter. An excess of Et_2O (~25 ml) was added to the filtrate, and precipitate was filtered off and washed with Et_2O (3×5 ml). The powder was dried on air and then triturated in boiling MeOH (~10 ml). Part of the product was filtered off and filtrate was left to crystallize in fridge for 3 h. Then, precipitate was filtered off, washed with cold MeOH (~3 ml) and Et_2O (2×5 ml). Combined powdered product was dried in oven (15 min, 75 °C). White powder (55 mg, 43 %).

^{1}H NMR (D_2O + *t*BuOH, pD = 5.8 + 0.4): 3.17 (P–C<u>H</u>$_2$–NH_2, d, $^2J_{HP}$ 10.1, 2H), 3.98 (P–C<u>H</u>$_2$–N–Pht, d, $^2J_{HP}$ 8.8, 2H), 7.81–7.86 (**3**, m, 2H), 7.86–7.91 (**2**, m, 2H)
^{13}C{^{1}H} NMR (D_2O + *t*BuOH, pD = 5.8 + 0.4): 37.8 (P–<u>C</u>H_2–N–Pht, d, $^1J_{CP}$ 103.7), 38.7 (P–<u>C</u>H_2–NH_2, d, $^1J_{CP}$ 93.7), 124.3 (**2**), 131.9 (**1**), 135.5 (**3**), 170.5 (N–<u>C</u>=O)
^{31}P NMR (D_2O + *t*BuOH / 85% aq H_3PO_4, pD = 5.8 + 0.4): 24.8 (p, $^2J_{PH}$ 9.8, $^2J_{PH}$ 8.9)
MS(+): 255 (255, $[M+H]^+$), 509 (509, $[2M+H]^+$), 531 (531, $[2M+Na]^+$)
MS(–): 253 (253, $[M-H]^-$)
HRMS(+) (found (*calc*)): 255.0524 (*255.0529*, $C_{10}H_{12}N_2O_4P$), 509.0967 (*509.0986*, $C_{20}H_{23}N_4O_8P_2$)
TLC: 0.43 (*i*PrOH:conc. aq. NH_3:H_2O = 10:1:2), 0.16 (MeOH:*i*PrOH = 1:1), 0.19 (EtOH)

*(Aminomethyl)[(*N,N*-dibenzyl)-aminomethyl]phosphinic acid* ***D3****.*

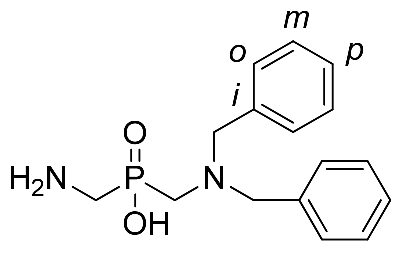

In 25-ml flask, phosphinic acid **D** (240 mg, 0.5 mmol, 1 equiv.) was dissolved in anhydrous EtOH (~15 ml) and $N_2H_4{\cdot}H_2O$ (37 mg, 0.8 mmol, 1.5 equiv.) was added. Solution was heated at 80 °C for 1 day. Then, the solution was cooled to room temperature and some precipitate was filtered off using 0.22 μm PVDF filter. The filtrate was evaporated to dryness *in vacuo*. The oily residue was suspended in $CHCl_3$ (~20 ml) and some more precipitate was filtered off using 0.22 μm PVDF filter. The filtrate was extracted by aq. HCl (1:1 dilution, 3×5 ml) and the amino-phosphinic acid was transferred into the aqueous phase. The solvents were removed *in vacuo* and the residue was once co-evaporated with MeOH (~5 ml). The oily residue was dissolved in MeOH (~5 ml) and product was precipitated by addition of Et_2O (~20 ml). Pure product was filtered off and washed with Et_2O (2×5 ml). Off-white hygroscopic powder **D3·3/2HCl·7/2H$_2$O** (105 mg, 50 %)).

^{1}H NMR (D_2O + *t*BuOH, pD = 1.2 + 0.4): 2.88 (P–C<u>H</u>$_2$–NH$_2$, d, $^2J_{HP}$ 9.9, 2H), 3.38 (P–C<u>H</u>$_2$–N–Bn, d, $^2J_{HP}$ 8.9, 2H), 4.57 (N–C<u>H</u>$_2$–Ph, s, 4H), 7.46–7.62 (Ph, m, 10H)
^{13}C{^{1}H} NMR (D_2O + *t*BuOH, pD = 1.2 + 0.4): 38.9 (P–<u>C</u>H$_2$–NH$_2$, d, $^1J_{CP}$ 101.1), 50.5 (P–<u>C</u>H$_2$–N–Bn, d, $^1J_{CP}$ 91.5), 60.1 (N–<u>C</u>H$_2$–Ph, d, $^3J_{CP}$ 3.6), 129.4 (*i*-Ph), 130.1 (*m*-Ph), 131.1 (*p*-Ph), 132.1 (*o*-Ph)
^{31}P NMR (D_2O + *t*BuOH / 85% aq H_3PO_4, pD = 1.2 + 0.4): 18.6 (p, $^2J_{PH}$ 9.7, $^2J_{PH}$ 8.9)
MS(+): 305 (305, $[M+H]^+$), 609 (609, $[2M+H]^+$)
MS(–): 303 (303, $[M–H]^-$), 607 (607, $[2M–H]^-$)
HRMS(+) (found (*calc*)): 305.1419 (*305.1413*, $C_{16}H_{22}N_2O_2P$), 609.2748 (*609.2754*, $C_{32}H_{43}N_4O_4P_2$)
TLC: 0.60 (*i*PrOH:conc. aq. NH_3:H_2O = 10:1:2), 0.17 (MeOH:*i*PrOH = 1:1), 0.15 (EtOH)
EA(found (*calc* M · 3/2HCl · 7/2H$_2$O)): C 45.39 (*45.53*), H 5.89 (*7.05*), N 6.92 (*6.64*), P 7.14 (*7.34*), Cl 12.17 (*12.60*)

4. X-ray Diffraction Experimental and Data

The diffraction data were collected at (*i*) 120 K: [H_3(*N*,*N″*-dibenzyl)-diethylene-triamine]Cl_3, **1**, **12**, **13**·0.25H_2O, **17**·2H_2O, BnNHCH_2PO_2H_2, **C**·PhPO_3H_2, **D**) or (*ii*) 150 K (all other structures). Data acquisition was carried out on (*i*) Nonius KappaCCD diffractometer equipped with Cryostream Cooler (Oxford Cryosystem) and with Bruker APEX-II CCD detector using monochromatized Mo-*K*α radiation (λ 0.71073 Å): **2**, **5**, **10**, **11**, $(AdNH_3)^+$**(18)**$^-$·H_2O, **22**·H_2O, Bn_2NCH_2PO_2H_2, **C**·PhPO_3H_2) or (*ii*) Bruker D8 VENTURE Kappa Duo PHOTON100 diffractometer with IµS micro-focus sealed tube: **12**, **13**·0.25H_2O, **17**·2H_2O, **D**) using Cu-*K*α (λ 1.54178 Å) radiation or [H_3(*N*,*N″*-dibenzyl)-diethylenetriamine]Cl_3, **1**, **4**·2H_2O, **8**·H_2O, **20**·MeOH, **25** using Mo-*K*α (λ 0.71073 Å) radiation

Data were analysed using the SAINT (Bruker AXS Inc.) software package. Data were corrected for absorption effects using the multi-scan method (SADABS). All structures were solved by direct methods (SHELXT2014)[12] and refined using full-matrix least-squares techniques (SHELXL2014).[13] All non-hydrogen atoms were refined anisotropically. All hydrogen atoms were found in the difference density map. However, the appropriate numbers of hydrogen atoms bound to carbon atoms were fixed in theoretical positions using $U_{eq}(H) = 1.2\ U_{eq}(C)$ to keep a number of parameters low, and only hydrogen atoms bound to heteroatoms (N, O, P) were fully refined.

In the crystal structures of **1**, **2**, **5**, **10**, **11**, **12**, **25**, BnNHCH_2PO_2H_2 and **D**, only aminophosphinate molecules are present. In the crystal structures of **4**·2H_2O, **8**·H_2O, **13**·0.25H_2O, **17**·2H_2O and **22**·H_2O, also water molecules of crystallization are present. In the case of **13**·0.25H_2O, the occupancy of water molecule was constrained to 0.25 to obtain reliable thermal factor. In the case of **17**·2H_2O, a hard-to-be-modelled disorder of water molecules was found. Therefore, appropriate solvate contribution was squeezed using PLATON.[14] In addition in this case, a planar-symmetry forced disorder of the phosphinate group with phosphorus atom disordered in two close positions sharing oxygen atoms which are positioned in the symmetry plane was found. The compound **18** was crystallized in form of adamantylammonium salt monohydrate, the compound **20** crystallizes as a MeOH solvate, and the compound **C** was isolated as an adduct with phenylphosphonic acid, **C**·PhPO_3H_2. In the case of adamantylammonium salt of **18** and Bn_2NCH_2PO_2H_2, two formula units form the structurally independent unit. For [H_3(*N*,*N″*-dibenzyl)-diethylenetriamine]Cl_3 and **17**·2H_2O, symmetric molecules were found and it leads to half-formula as the structurally independent part. For all compounds except the above-mentioned ones, one formula unit forms the independent part of the crystal structures. Except structure of **17**·2H_2O discussed above, no disorder was found in any other structure.

Table S5 contains selected experimental crystallographic parameters for the structures reported in this paper. Data for the structures have been deposited the Cambridge Crystallographic Data Centre (for CCDC reference numbers see also Table S5). Parameters of intramolecular and intermolecular hydrogen bonds are outlined in Tables S6 and S7. Molecular structures of the compounds those solid-state structures were determined by X-ray diffraction are shown together with other characterizations of the compounds (see above).

---

[12] *a*) G. M. Sheldrick, *SHELXT2014/5. Program for Crystal Structure Solution from Diffraction Data*, University of Göttingen, Göttingen, 2014; (*b*) G. M. Sheldrick, *Acta Crystallogr. Sect. A.*, **2008**, *A64*, 112–122.
[13] (*a*) C. B. Hübschle, G. M. Sheldrick and B. Dittrich, *ShelXle: a Qt graphical user interface for SHELXL*, University of Göttingen, Göttingen, 2014. (*b*) C. B. Hübschle, G. M. Sheldrick and B. Dittrich, *J. Appl. Crystallogr.*, **2011**, *44*, 1281–1284. (*c*) G. M. Sheldrick, *SHELXL-2014/7. Program for Crystal Structure Refinement from Diffraction Data*, University of Göttingen, Göttingen, 2017; (*d*) G. M. Sheldrick, *Acta Crystallogr. Sect. C*, **2015**, *C71*, 3–8.
[14] (*a*) A. L. Spek, *PLATON A Multipurpose Crystallographic Tool*, Utrecht University, Utrecht, The Netherlands, 2019. (*b*) A. L. Spek, *Acta Crystallogr.*, 2009, **D65**, 148–155.

Table S5. Experimental parameters of the reported crystal structures and their CCDC numbers.

Parameter	[H_3(*N*,*N″*-dibenzyl)-diethylene-triamine]Cl_3	**1**	**2**	**4**·$2H_2O$	**5**	**8**·H_2O	**10**	**11**	**12**	**13**·$0.25H_2O$
Formula	$C_{18}H_{28}Cl_3N_3$	$C_{15}H_{18}NO_2P$	$C_3H_{10}NO_2P$	$C_7H_{22}NO_4P$	$C_{13}H_{26}NO_2P$	$C_5H_{14}NO_4P$	$C_4H_{10}NO_4P$	$C_{10}H_{14}NO_4P$	$C_5H_{10}NO_6P$	$C_6H_{12.5}NO_{4.25}P$
M_r	392.78	275.27	123.09	215.22	259.32	183.14	167.10	243.19	211.11	197.64
Habit	plate	prism	prism	prism	prism	plate	prism	prism	prism	bar
Colour	colourless	colourless	colourless	colourless	colourless	colourless	colourless	colourless	colourless	colourless
Crystal system	monoclinic	monoclinic	orthorhombic	monoclinic	triclinic	orthorhombic	orthorhombic	triclinic	triclinic	triclinic
Space group	*C*2	$P2_1/n$	$Pna2_1$	$C2/c$	$P{-}1$	*Pbca*	$P2_12_12_1$	$P{-}1$	$P{-}1$	$P2_1/n$
a, Å	39.208(3)	10.2763(4)	9.7130(3)	13.3640(6)	8.5485(2)	10.3557(4)	7.9050(4)	5.6610(2)	5.1523(6)	5.5885(3)
b, Å	4.9186(3)	9.1700(3)	10.9592(3)	8.5851(4)	8.7247(2)	12.4193(5)	7.9314(4)	8.5501(3)	7.4078(9)	19.127(1)
c, Å	5.1714(4)	15.3112(6)	5.5558(2)	20.140(1)	10.3439(2)	13.5892(4)	11.8753(5)	12.0035(4)	11.850(1)	8.3332(5)
α, °	90	90	90	90	71.418(1)	90	90	78.854(1)	101.004(5)	90
β, °	90.465(3)	103.229(1)	90	91.384(2)	68.689(1)	90	90	79.171(1)	95.811(5)	103.684(3)
γ, °	90	90	90	90	81.280(1)	90	90	78.139(1)	103.446(5)	90
U, Å^3	997.26(12)	1404.54(9)	591.40(3)	2310.1(2)	680.70(3)	1747.7(1)	744.55(6)	551.19(3)	426.75(9)	865.48(9)
Z	2	4	4	8	2	8	4	2	2	4
D_{calc}, g cm^{-3}	1.308	1.302	1.382	1.238	1.265	1.392	1.491	1.465	1.643	1.517

μ, mm^{-1}	0.465	0.193	0.362	0.226	0.194	0.286	0.329	0.248	2.968	2.723
Unique refl.	2107	3210	1343	2663	3105	2003	1717	2526	1679	1684
Obsd. refl. (I>2σ(I))	2036	2966	1329	2425	2803	1750	1690	2333	1527	1545
R(I>2σ(I))	0.0376	0.0310	0.0181	0.0288	0.0312	0.0338	0.0197	0.0304	0.0309	0.0431
R'(all)	0.0399	0.0338	0.0183	0.0329	0.0353	0.0402	0.0200	0.0336	0.0345	0.0467
wR(I>2σ(I))	0.0911	0.0798	0.0520	0.0746	0.0821	0.0839	0.0552	0.0739	0.0821	0.1067
wR'(all)	0.0920	0.0817	0.0521	0.0767	0.0846	0.0879	0.0554	0.0759	0.0798	0.1090
**CCDC number**	**1984986**	**1984991**	**1984993**	**1984990**	**1985003**	**1984994**	**1984996**	**1984997**	**1984992**	**1985000**

Parameter	**17**·2H_2O	$(AdNH_3)^+$(**18**)$^-$·H_2O	**20**·MeOH	**22**·H_2O	**25**	$BnNHCH_2PO_2H_2$	**C**·$PhPO_3H_2$	**D**
Formula	$C_{21}H_{24}N_3O_8P$	$C_{26}H_{39}N_2O_3P$	$C_{23}H_{28}NO_3P$	$C_9H_{17}NO_5P_2$	$C_6H_{17}NO_4P_2$	$C_8H_{12}NO_2P$	$C_{27}H_{29}NO_5P_2$	$C_{24}H_{23}N_2O_4P$
M_r	477.40	458.56	397.43	281.17	229.14	185.16	509.45	434.41
Habit	prism	bar	prism	plate	prism	prism	prism	prism
Colour	colourless	colourless	colourless	colourless	colourless	colourless	colourless	colourless
Crystal system	monoclinic	triclinic	triclinic	monoclinic	monoclinic	orthorhombic	monoclinic	triclinic
Space group	$P2_1/m$	$P-1$	$P-1$	$P2_1$	$P2_1/c$	$Pca2_1$	$P2_1/c$	$P-1$
a, Å	5.6105(2)	6.4395(2)	9.7954(5)	7.0189(3)	8.6777(3)	10.4097(4)	9.4746(4)	9.9559(5)
b, Å	21.6543(9)	17.5869(5)	9.9645(5)	5.7569(2)	11.9197(3)	6.3878(3)	11.3717(5)	10.8993(5)
c, Å	8.9960(4)	22.0784(6)	11.1729(6)	15.9662(8)	10.7504(3)	27.1688(12)	23.1551(10)	11.2372(5)
α, °	90	89.251(2)	73.496(2)	90	90	90	90	110.848(1)
β, °	94.695(2)	89.612(1)	82.075(2)	98.694(2)	104.459(1)	90	91.990(1)	92.553(1)
γ, °	90	83.824(1)	87.525(2)	90	90	90	90	110.000(1)
U, Å^3	1089.27(8)	2485.6(1)	1035.62(9)	637.73(5)	1076.75(6)	1806.59(14)	2493.28(19)	1051.45(9)
Z	2	4	2	2	4	8	4	2
D_{calc}, g cm^{-3}	1.456	1.225	1.275	1.464	1.414	1.361	1.357	1.372
μ, mm^{-1}	1.604	0.140	0.156	0.350	0.390	0.263	0.213	1.448

Unique refl.	1961	10831	4763	2908	2457	3454	5741	3984
Obsd. refl. (I>2σ(I))	1822	7572	4190	2630	2387	3301	4966	3629
R(I>2σ(I))	0.0428	0.0506	0.0345	0.0308	0.0238	0.0415	0.0337	0.0333
R'(all)	0.0451	0.0858	0.0415	0.0375	0.0243	0.0433	0.0420	0.0373
wR(I>2σ(I))	0.1050	0.1032	0.0857	0.0734	0.0666	0.1078	0.0787	0.0810
wR'(all)	0.1064	0.1164	0.0902	0.0761	0.0670	0.1090	0.0840	0.0834
**CCDC number**	**1984995**	**1984988**	**1984987**	**1984999**	**1985002**	**1984998**	**1984989**	**1985001**

Table S6. Parameters of intramolecular hydrogen bonds found in the solid state structures of the prepared compounds.

Compound	Distance, Å		Angle, °	
**2**	N3···O11	3.156(2)	N3–H31···O11	97(2)
**4**·2H_2O	N3···O11	3.205(1)	N3–H31···O11	109(1)
**5**	N3···O11	3.045(1)	N3–H31···O11	115(1)
**8**·H_2O	N3···O11	2.946(2)	N3–H31···O11	101(1)
**10**	N3···O52	2.761(2)	N3–H31···O52	94(1)
**11**	N3···O52	2.732(1)	N3–H31···O52	101(1)
**12**	N3···O11	2.823(2)	N3–H31···O11	120(2)
	N3···O52	2.729(2)	N3–H31···O52	104(2)
	N3···O72	2.698(2)	N3–H31···O72	110(2)
**13**·0.25H_2O	N3···O412	2.698(3)	N3–H31···O412	113(2)
**20**·MeOH	N3···O11	2.852(1)	N3–H31···O11	111(1)
**22**·H_2O	N3···O21	2.998(3)	N3–H31···O21	96(2)
**25**	N3···O22	3.181(1)	N3–H31···O22	111(1)
$BnNHCH_2PO_2H_2$	N3A···O11A	2.862(5)	N3A–H31A···O11A	79(4)
	N3X···O11X	2.875(5)	N3X–H31X···O11X	87(3)
**C**·$PhPO_3H_2$	N3···O12	3.184(2)	N3–H31···O12	95(1)
**D**	N3···O11	2.799(1)	N3–H31···O11	117(1)

Table S7. Parameters of intermolecular hydrogen bonds found in the solid state structures of the prepared compounds.

**Compound**	**D–H**	***d*(D–H), Å**	***d*(H···A), Å**	**<DHA, °**	***d*(D···A), Å**	**A [symmetry code]**
**1**	N3–H31	0.922	1.737	172.91	2.654	O11 [−*x*+½, *y*−½, −*z*+1½]
**2**	N3–H31	0.886	1.788	166.13	2.657	O11 [−*x*, −*y*, *z*−½]
**4**·$2H_2O$	N3–H31	0.886	1.842	168.12	2.714	O11 [−*x*+1, −*y*+1, −*z*+1]
	O1W–H11W[a]	0.863	1.899	172.29	2.757	O12
	O1W–H12W[a]	0.826	1.966	171.36	2.786	O12 [−*x*+1, *y*, −*z*+1½]
	O2W–H21W[a]	0.855	1.963	160.72	2.785	O1W[a]
	O2W–H22W[a]	0.863	2.062	154.48	2.864	O1W[a] [−*x*+½, *y*−½, −*z*+1½]
**5**	N3–H31	0.883	1.926	152.68	2.740	O11 [−*x*+1, −*y*+1, −*z*+1]
**8**·H_2O	N3–H31	0.911	1.819	160.06	2.693	O11 [−*x*+1, −*y*+1, −*z*+1]
	O1W–H11W[a]	0.847	1.938	161.27	2.754	O12
	O1W–H12W[a]	0.841	1.957	162.62	2.770	O11 [x−½, −y+½, −z+1]
**10**	N3–H31	0.895	1.765	169.89	2.650	O11 [−*x*+1, *y*+½, −*z*+½]
	O51–H511	0.859	1.661	171.75	2.514	O12 [*x*−½, −*y*+1½, −*z*+1]
**11**	N3–H31	0.887	1.861	158.02	2.704	O11 [*x*−1, *y*, *z*]
	O51–H511	0.865	1.671	170.74	2.529	O12 [*x*−1, *y*+1, *z*]
**12**	O51–H511	0.906	1.606	166.40	2.496	O12 [−*x*+1, −*y*+1, −*z*+1]
	O71–H711	0.847	1.685	161.14	2.502	O11 [−*x*+1, −*y*+1, −*z*]
**13**·$0.25H_2O$	N3–H31	0.916	1.959	143.68	2.751	O11 [*x*−½, −*y*+½, *z*−½]
	O411–H411	1.056	1.406	176.38	2.461	O12 [*x*−½, −*y*+½, *z*+½]
	O1W–H11W[a]	0.922	1.883	152.77	2.736	O12
**17**·$2H_2O$	N3–H31	0.966	1.678	163.56	2.619	O11 [*x*−1, *y*, *z*]
$(AdNH_3)^+$(**18**)$^-$·H_2O	N30A–H30A	0.872	2.037	170.52	2.901	O1W[a] [*x*+1, *y*, *z*]
	N30A–H30B	0.931	1.879	164.08	2.786	O12A [−*x*, −*y*+1, −*z*]
	N30A–H30C	1.014	1.797	170.76	2.802	O11A
	N30X–H30X	0.881	2.045	170.76	2.918	O2W[a] [*x*+1, *y*, *z*]
	N30X–H30Y	0.951	1.845	170.14	2.788	O12X [−*x*+1, −*y*+1, −*z*+1]
	N30X–H30Z	0.953	1.839	166.13	2.773	O11X
	O1W–H11W[a]	0.849	1.951	166.88	2.784	O11A

	O1W–H12W[a]	0.850	1.993	172.67	2.838	O12A [−*x*, −*y*+1, −*z*]
	O2W–H21W[a]	0.894	2.095	162.78	2.961	O11X
	O2W–H22W[a]	0.844	1.974	172.64	2.813	O12X [−*x*+1, −*y*+1, −*z*+1]
**20**·MeOH	N3–H31	0.910	1.871	157.75	2.736	O11 [−*x*+1, −*y*+1, −*z*+1]
	O1M–H1M[b]	0.835	1.806	176.62	2.640	O12
**22**·H_2O	O11–H11O	1.032	1.379	175.07	2.409	O21 [*x*+1, *y*+1, *z*]
	N3–H31	0.819	1.901	165.31	2.701	O12 [*x*, *y*−1, *z*]
	O1W–H11W[a]	0.804	2.069	159.17	2.835	O22 [−*x*, *y*+½, −*z*+1]
	O1W–H12W[a]	0.793	1.986	170.23	2.771	O22 [*x*+1, *y*, *z*]
**25**	O11–H11O	0.952	1.491	176.64	2.442	O21 [*x*, −*y*+1/2, *z*+½]
	N3–H31	0.868	1.881	161.80	2.719	O22 [−*x*+1, −*y*+1, −*z*+1]
$BnNHCH_2PO_2H_2$	N3A–H31A	1.050	1.704	160.58	2.717	O11X
	N3A–H32A	1.042	1.769	152.87	2.738	O12X [*x*, *y*+1, *z*]
	N3X–H31X	0.868	1.865	175.62	2.731	O11A
	N3X–H32X	0.920	1.889	152.69	2.739	O12A [*x*, *y*−1, *z*]
**C**·$PhPO_3H_2$	N3–H31	0.929	1.725	174.65	2.652	O23 [−*x*+1, *y*−½, −*z*+1½]
	O21–H21O	0.914	1.564	174.99	2.476	O11
	O22–H22O	0.896	1.620	177.98	2.516	O12 [−*x*+1, *y*+½, −*z*+1½]
**D**	N3–H31	0.912	1.977	140.20	2.740	O11 [−*x*+1, −*y*, −*z*+1]

[a]W – atom belonging to a water molecule. [b]M – atom belonging to a methanol molecule.
