# Supplementary material for: Selective and clean synthesis of aminoalkyl-H-phosphinic acids from hypophosphorous acid by phospha-Mannich reaction
Source: RSC Adv. 2020 Jun 4;10(36):21329–49. doi: 10.1039/d0ra03075a (PMC9059144; doi:10.1039/d0ra03075a)
Supplement: RA-010-D0RA03075A-s002 [file RA-010-D0RA03075A-s002.pdf]

Electronic Supplementary Material (ESI) for RSC Advances.
This journal is © The Royal Society of Chemistry 2020

Electronic Supplementary Information for:

Selective and clean synthesis of amino H-phosphinic acids from hypophosphorus acid by phospha-Mannich reaction

Peter Urbanovský, Jan Kotek, Ivana Císařová and Petr Hermann*

Department of Inorganic Chemistry, Faculty of Science, Universita Karlova (Charles University), Hlavova 8/2030, 12843 Prague 2, Czech Republic. Tel.: +420-22195-1263, fax: +420-22195-1253, e-mail: petrh@natur.cuni.cz

Content:

NMR spectra for compounds (**2–17**) in Table 1 2–18

NMR spectra for compounds (**18–21b**) in Table 2 19–25

NMR spectra for compounds (**22–27**) in Table 3 26–31

NMR spectra for compounds (**28a–30**) in Table 4 32–35

NMR spectra for compounds (**31–35**) in Table 5 36–41

NMR spectra for compounds (**31a–35b**) in Table 6 42–51

NMR spectra for compounds based on cyclic amines (**16a, 36, 37, 37-Me, 38-Me**) 52–56

NMR spectra for additional compounds (**28c, 25a, 19a, 19b, B–E, D1–D3**) 57–67

NMR spectra for (*N*-benzyl)-aminomethyl-*H*-phosphinic acid 68

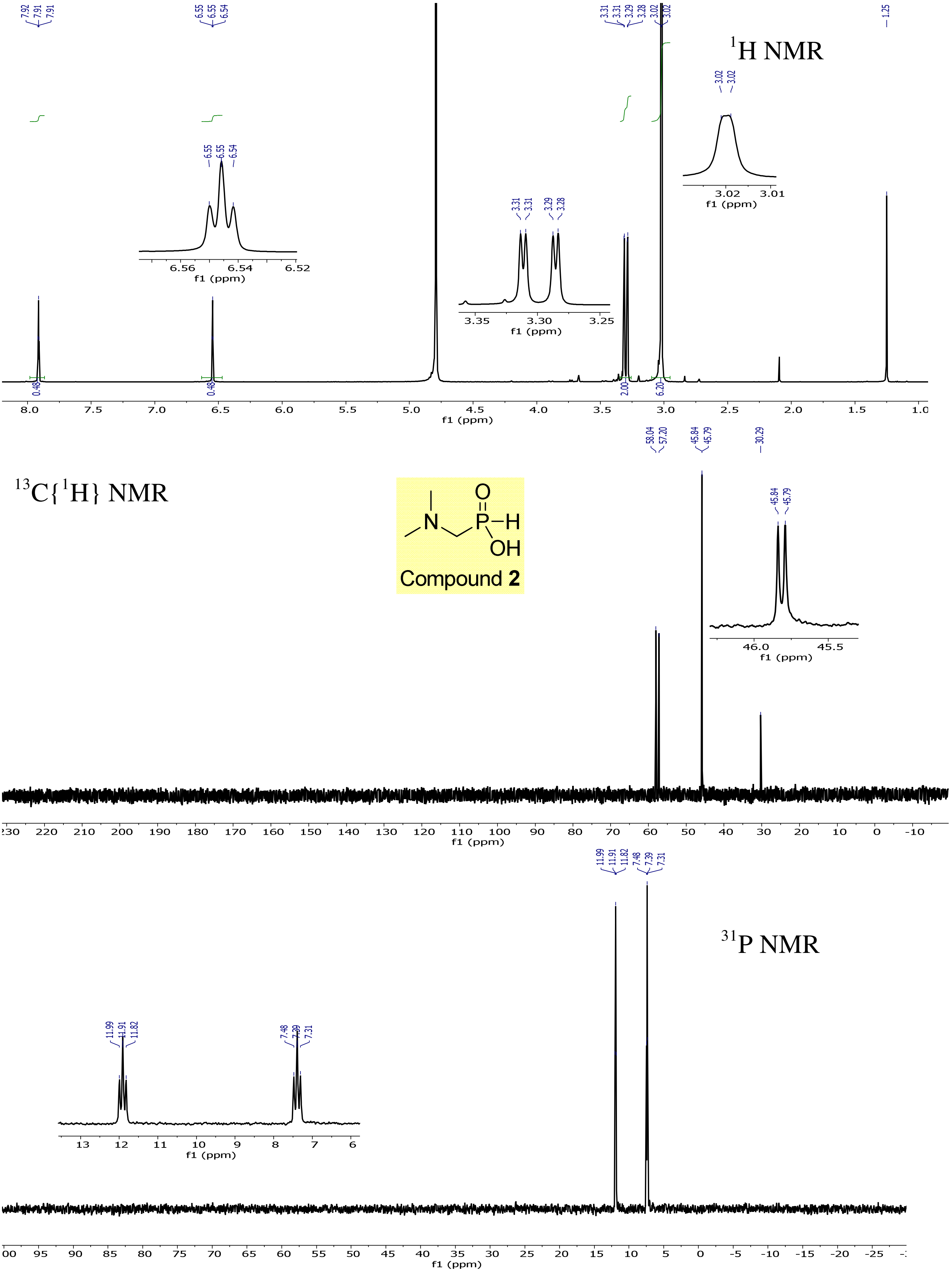

1H NMR
13C{1H} NMR
Compound 2
OH
31P NMR
f1 (ppm)

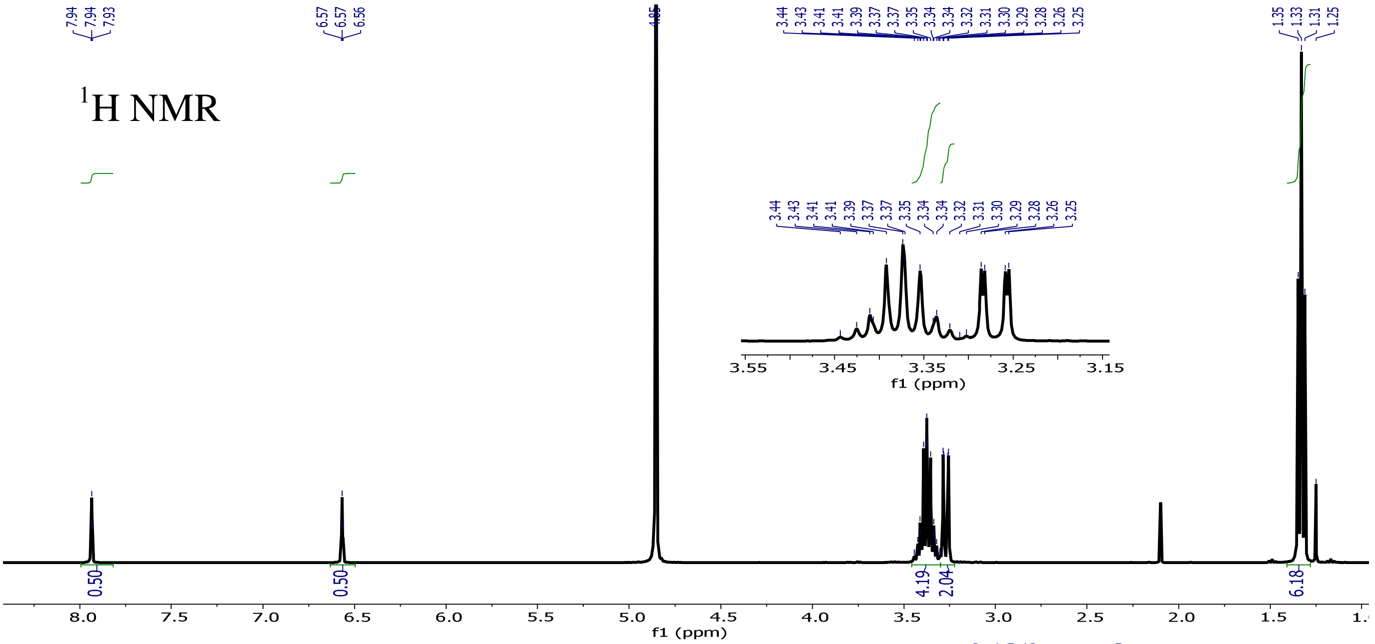
1H NMR
7.94 7.94 7.93
6.57 6.57 6.56
4.85
3.44 3.43 3.41 3.41 3.39 3.37 3.37 3.35 3.34 3.34 3.32 3.31 3.30 3.29 3.28 3.26 3.25
1.35 1.33 1.31 1.25
0.50
0.50
4.19
2.04
6.18
f1 (ppm)

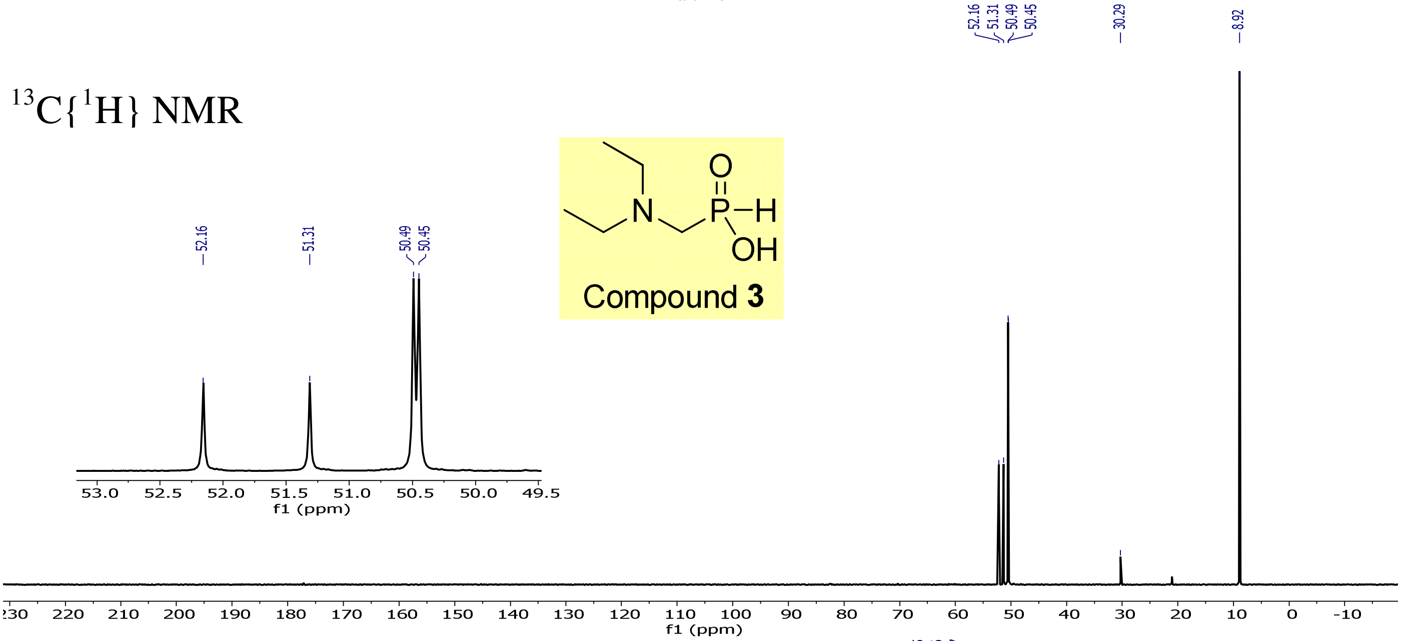
13C{1H} NMR
52.16 51.31 50.49 50.45
30.29
8.92
Compound 3
f1 (ppm)

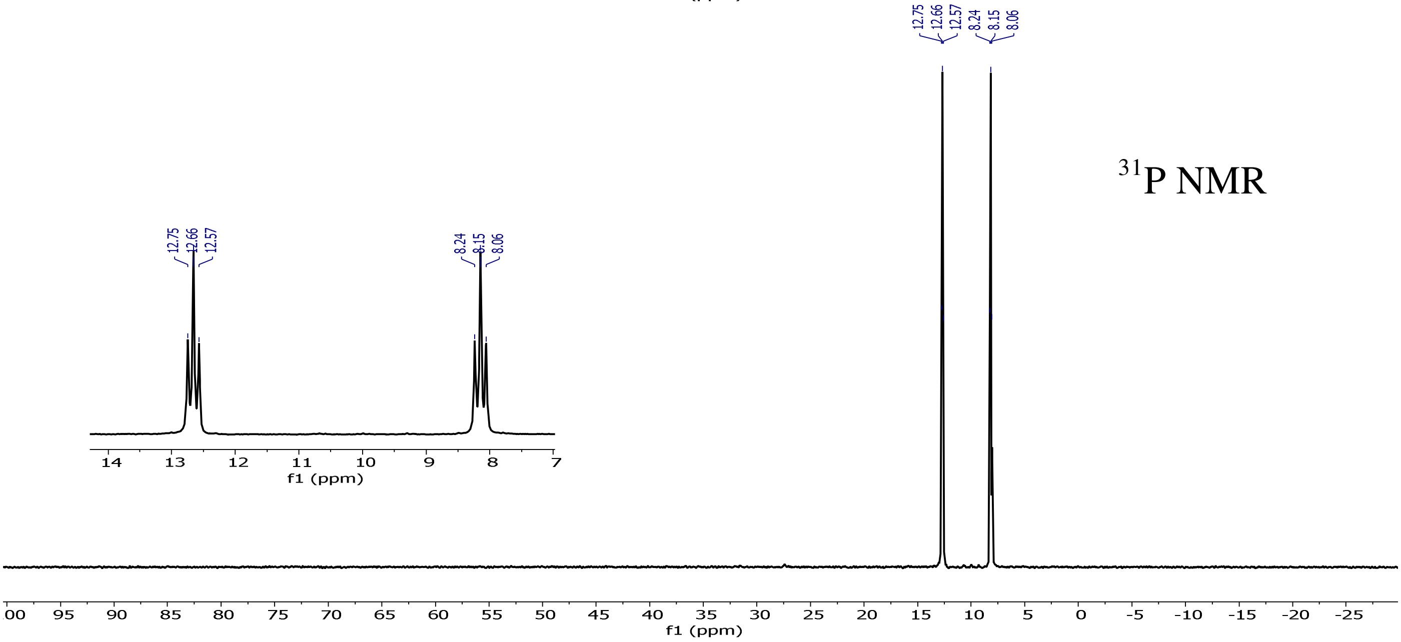
31P NMR
12.75 12.66 12.57 8.24 8.15 8.06
f1 (ppm)

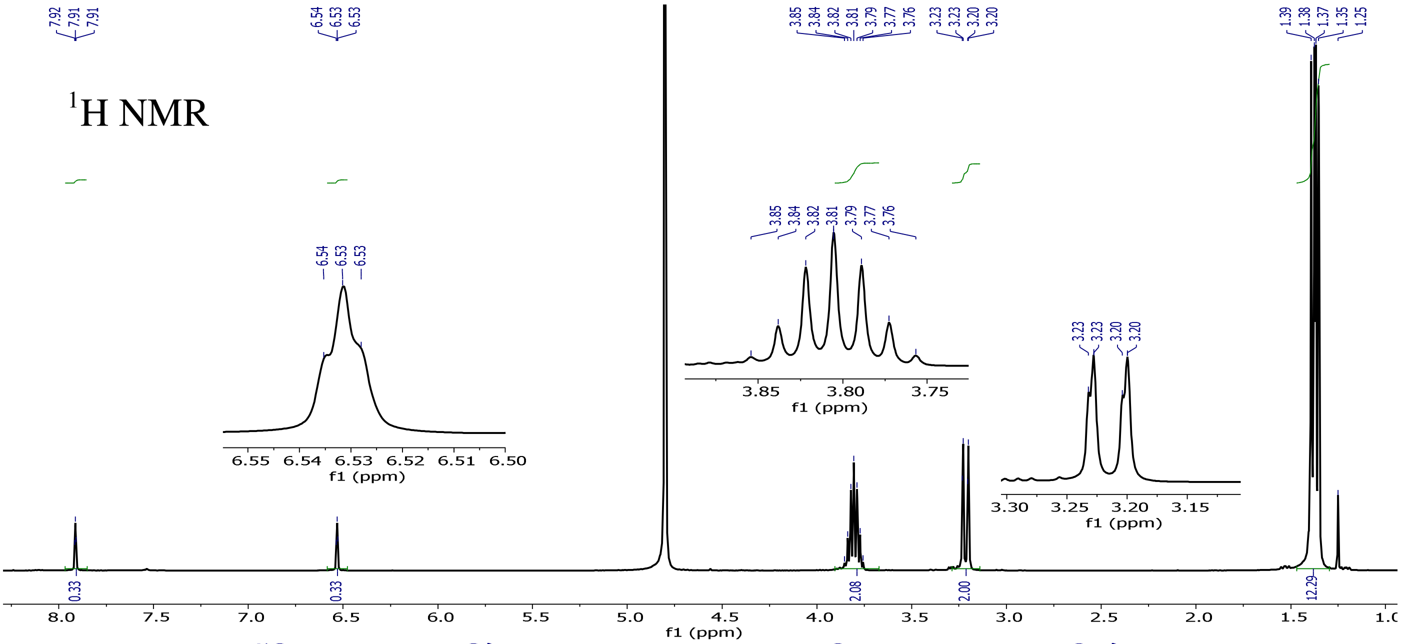

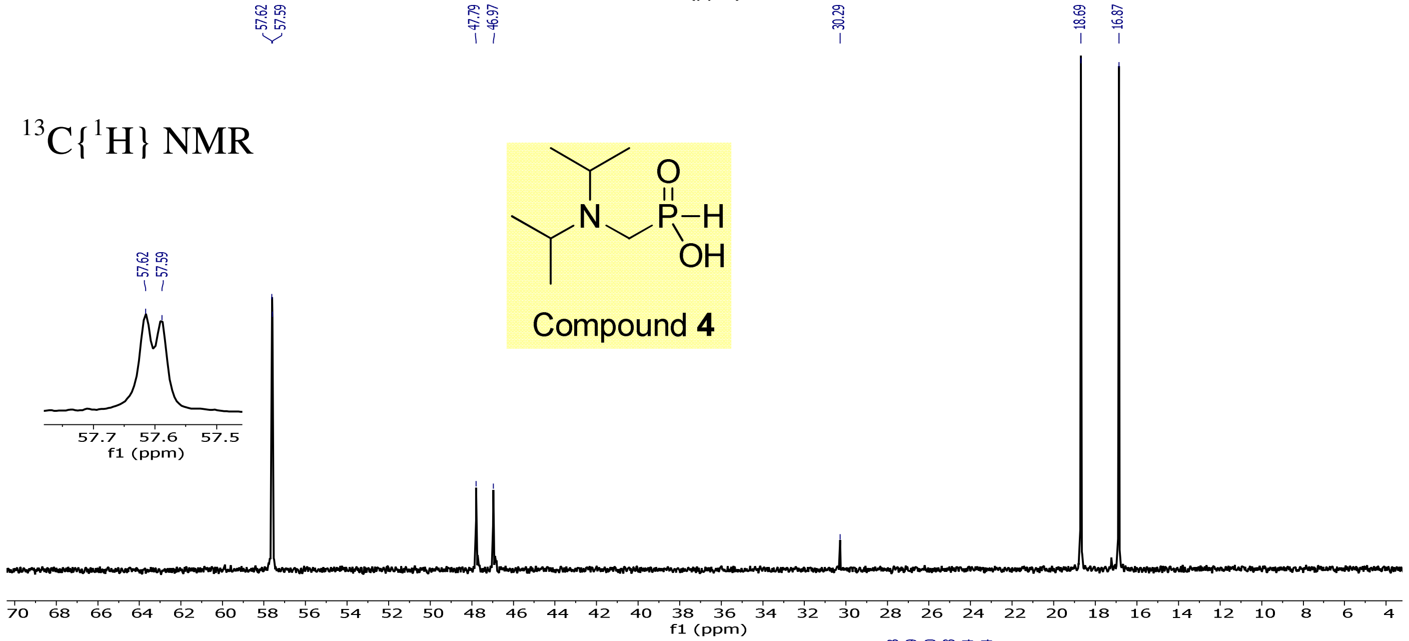

Compound **4**

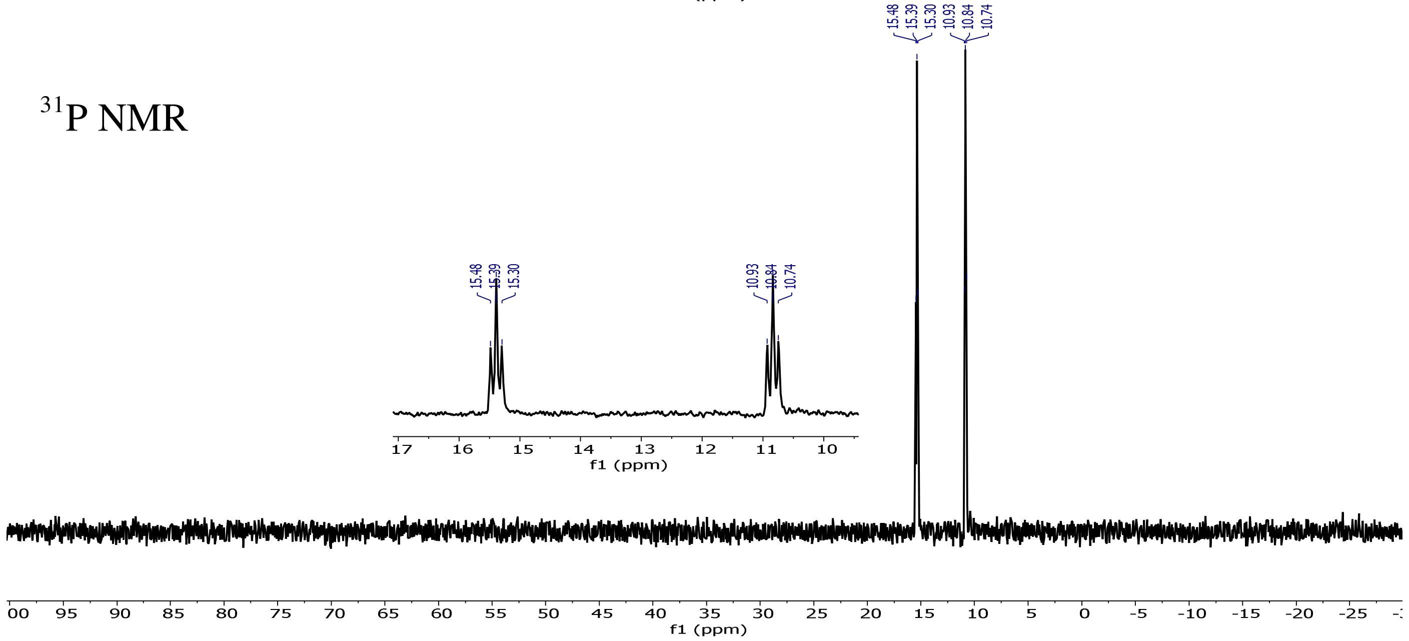

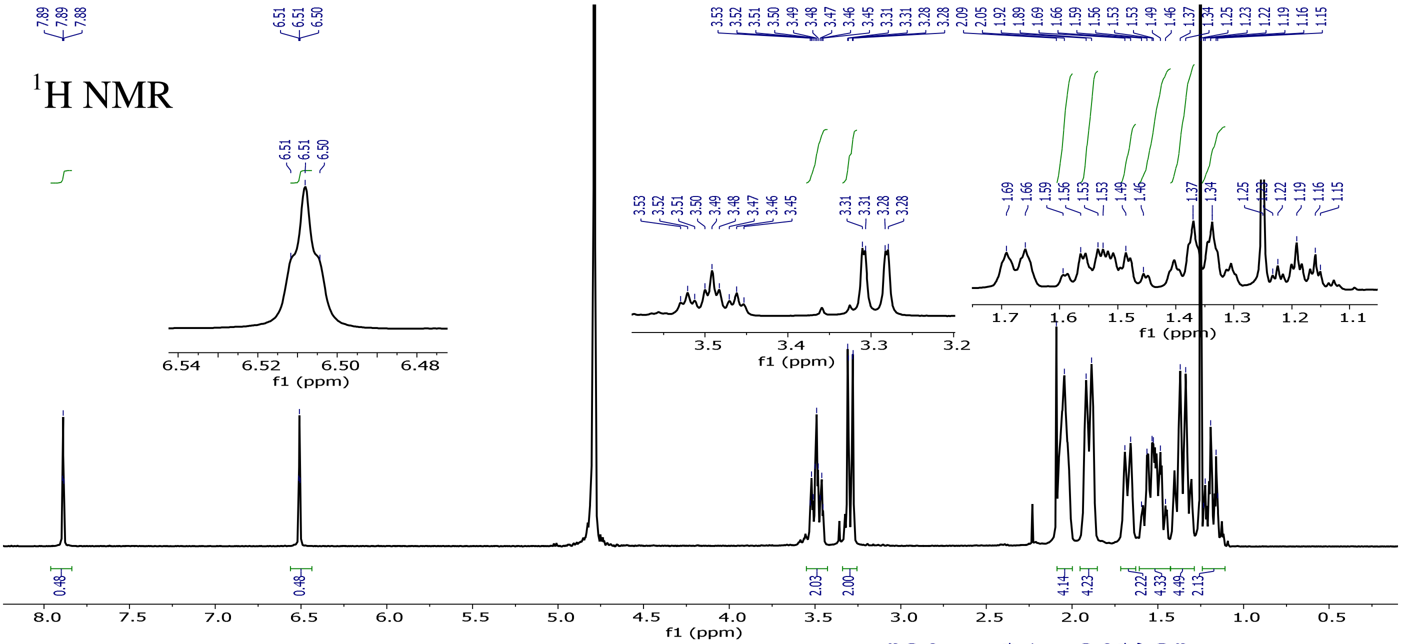

^{1}H NMR
f1 (ppm)

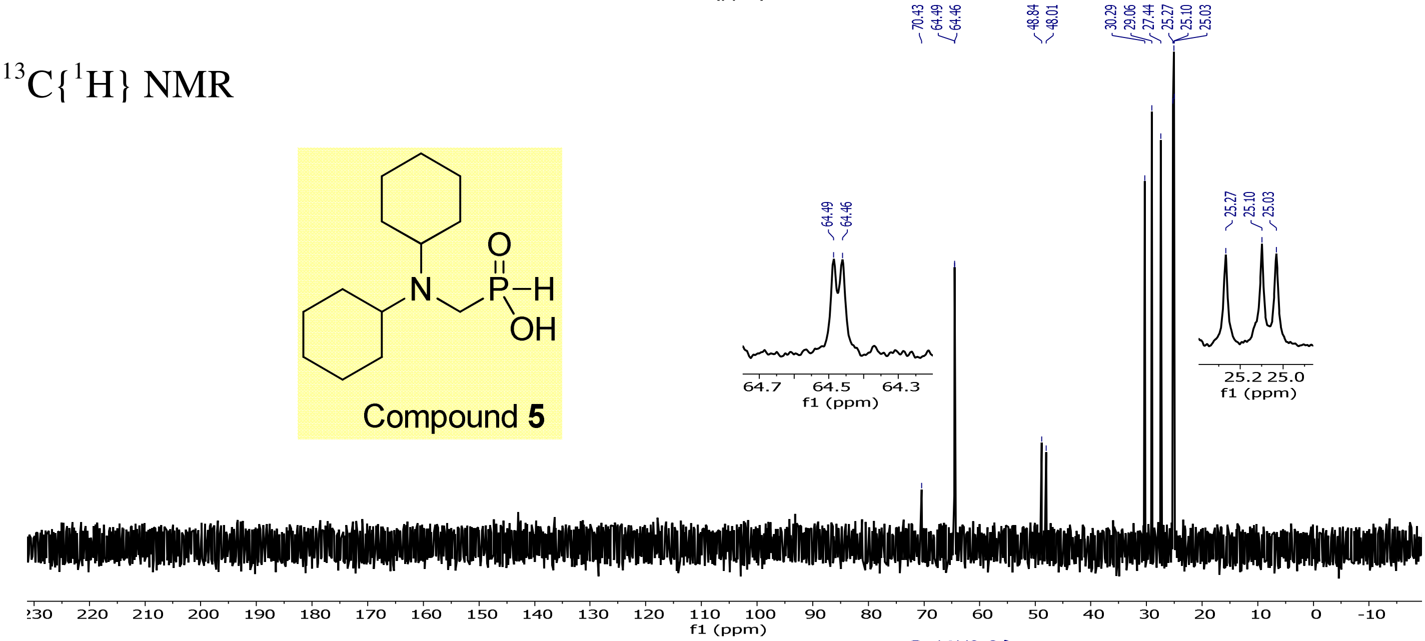

^{13}C{^{1}H} NMR
Compound **5**
f1 (ppm)

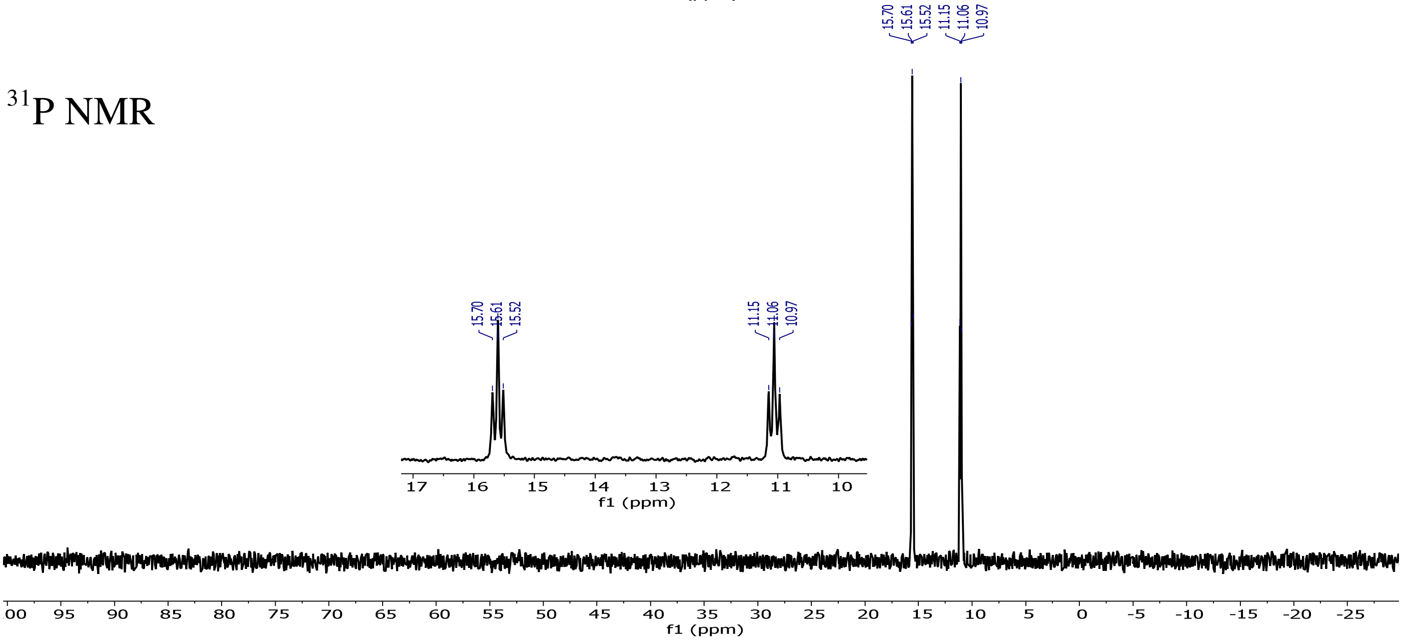

^{31}P NMR
f1 (ppm)

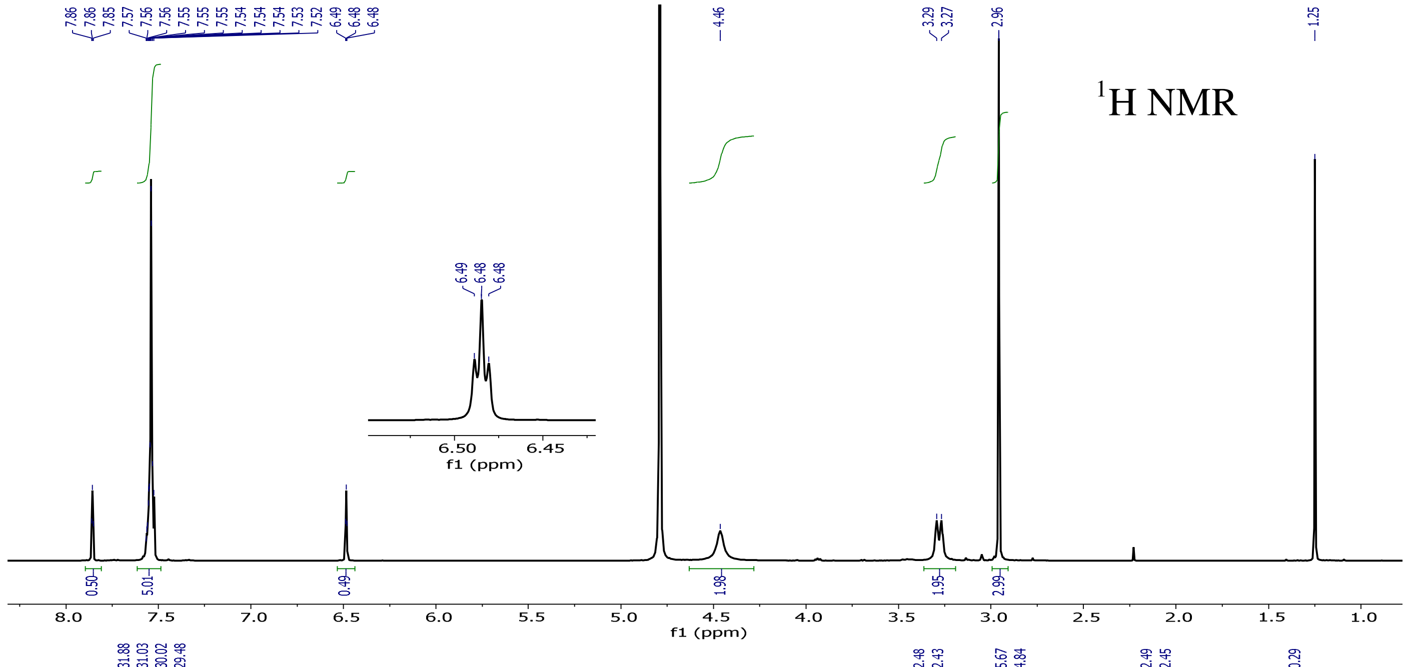

1H NMR
f1 (ppm)

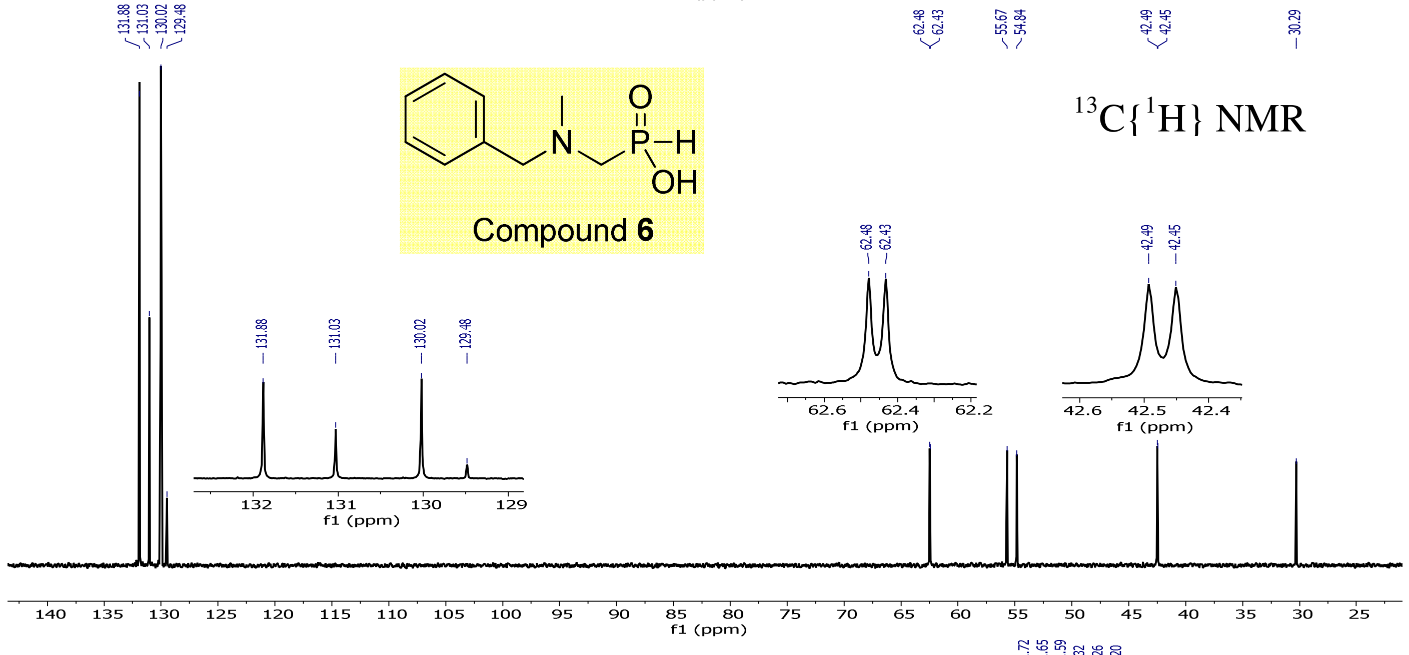

13C{1H} NMR
O
P–H
OH
N
Compound 6
f1 (ppm)

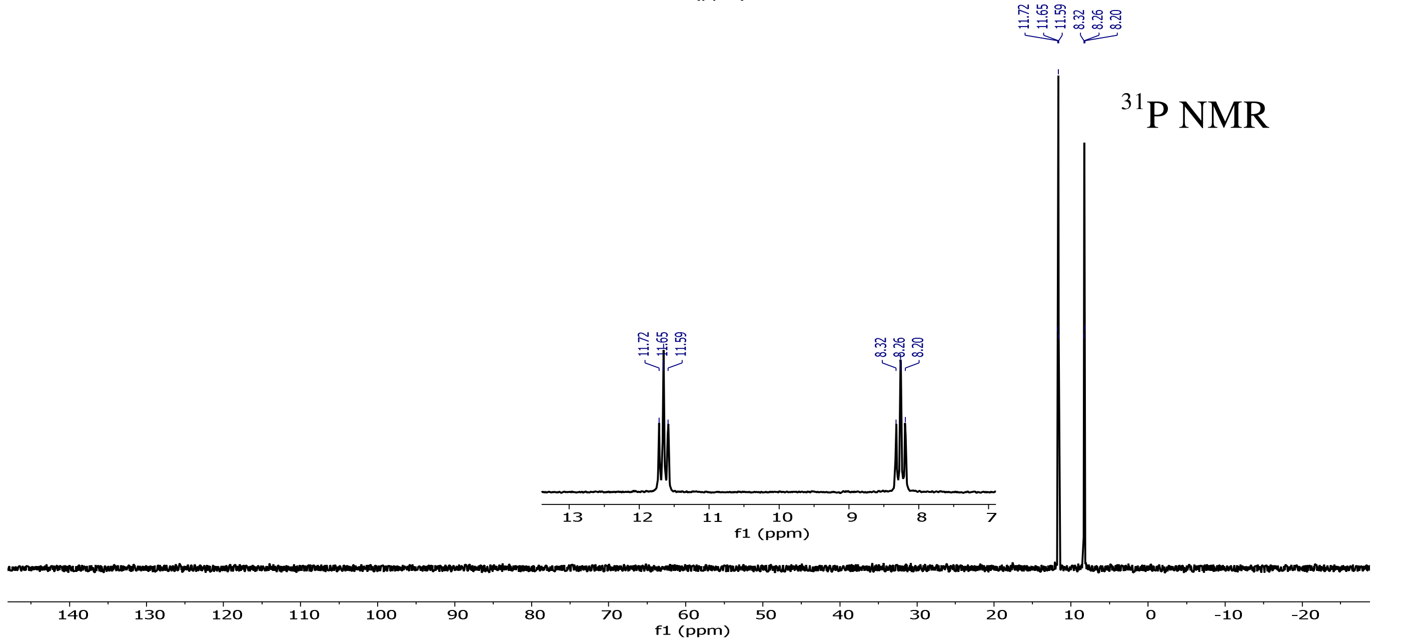

31P NMR
f1 (ppm)

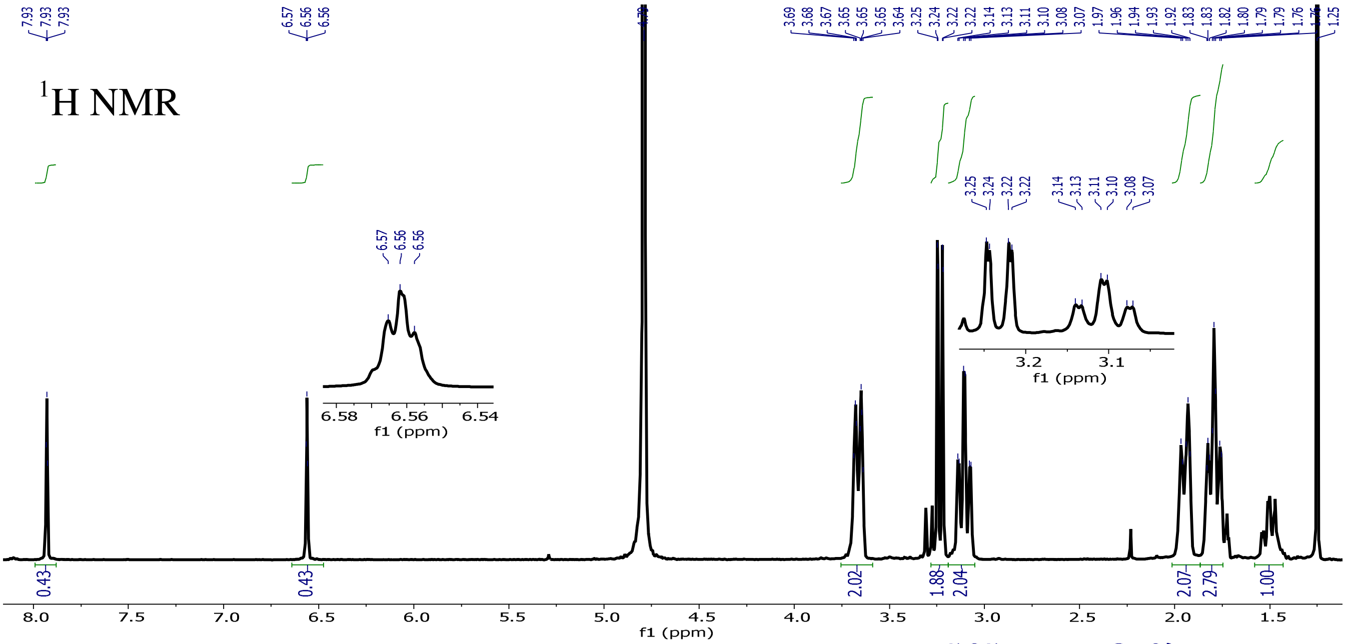
¹H NMR
f1 (ppm)

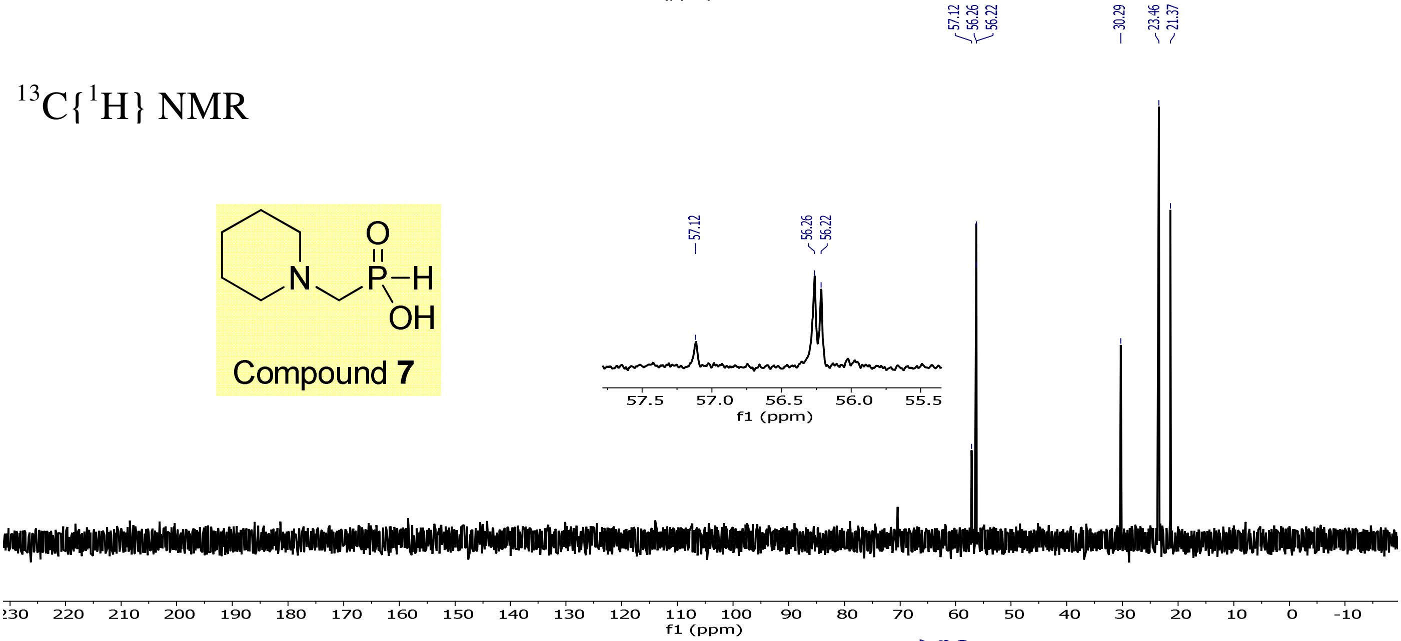
¹³C{¹H} NMR
N
P
O
H
OH
Compound 7
f1 (ppm)

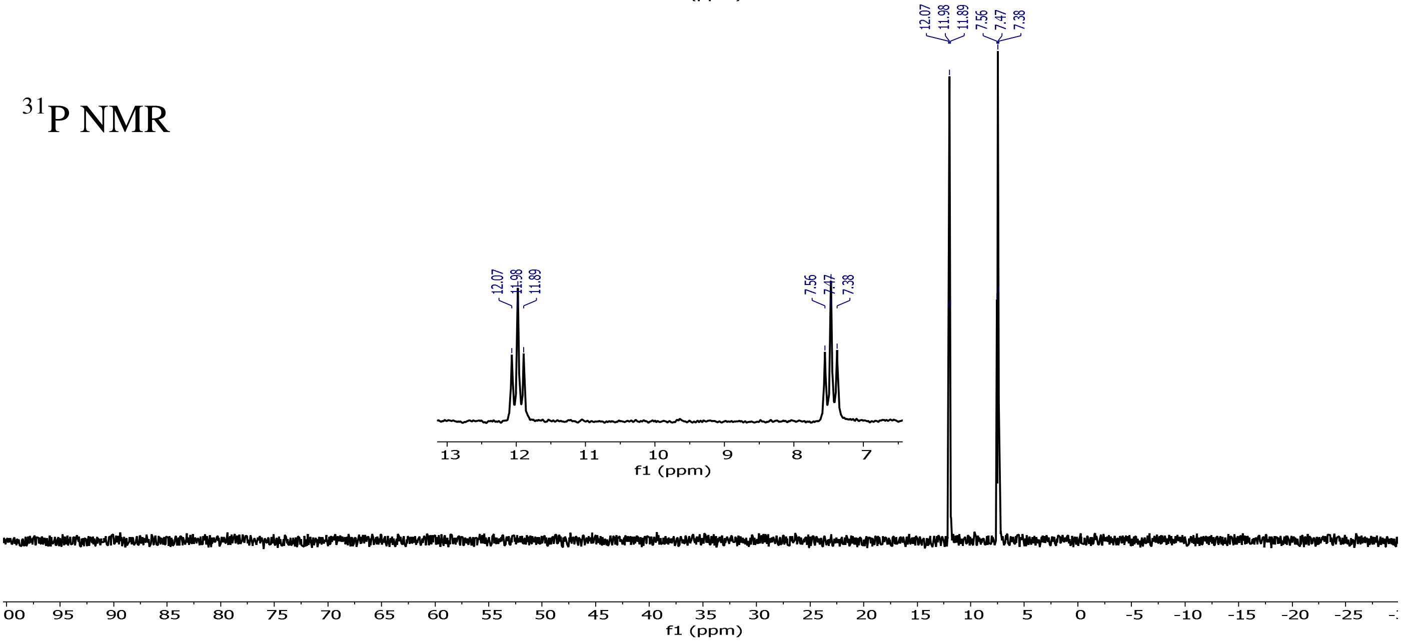
³¹P NMR
f1 (ppm)

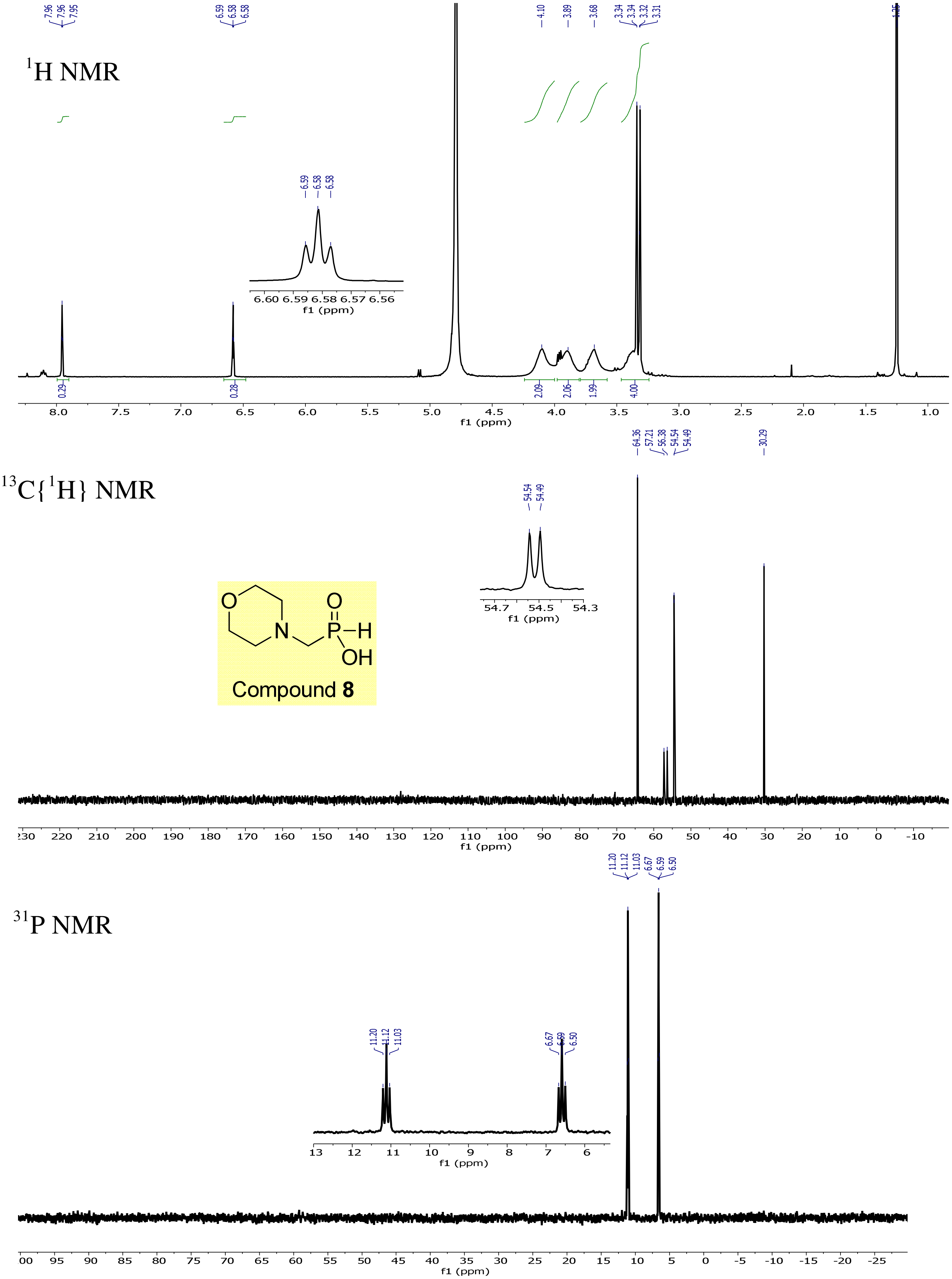
^{1}H NMR
^{13}C{^{1}H} NMR
Compound 8
^{31}P NMR
f1 (ppm)

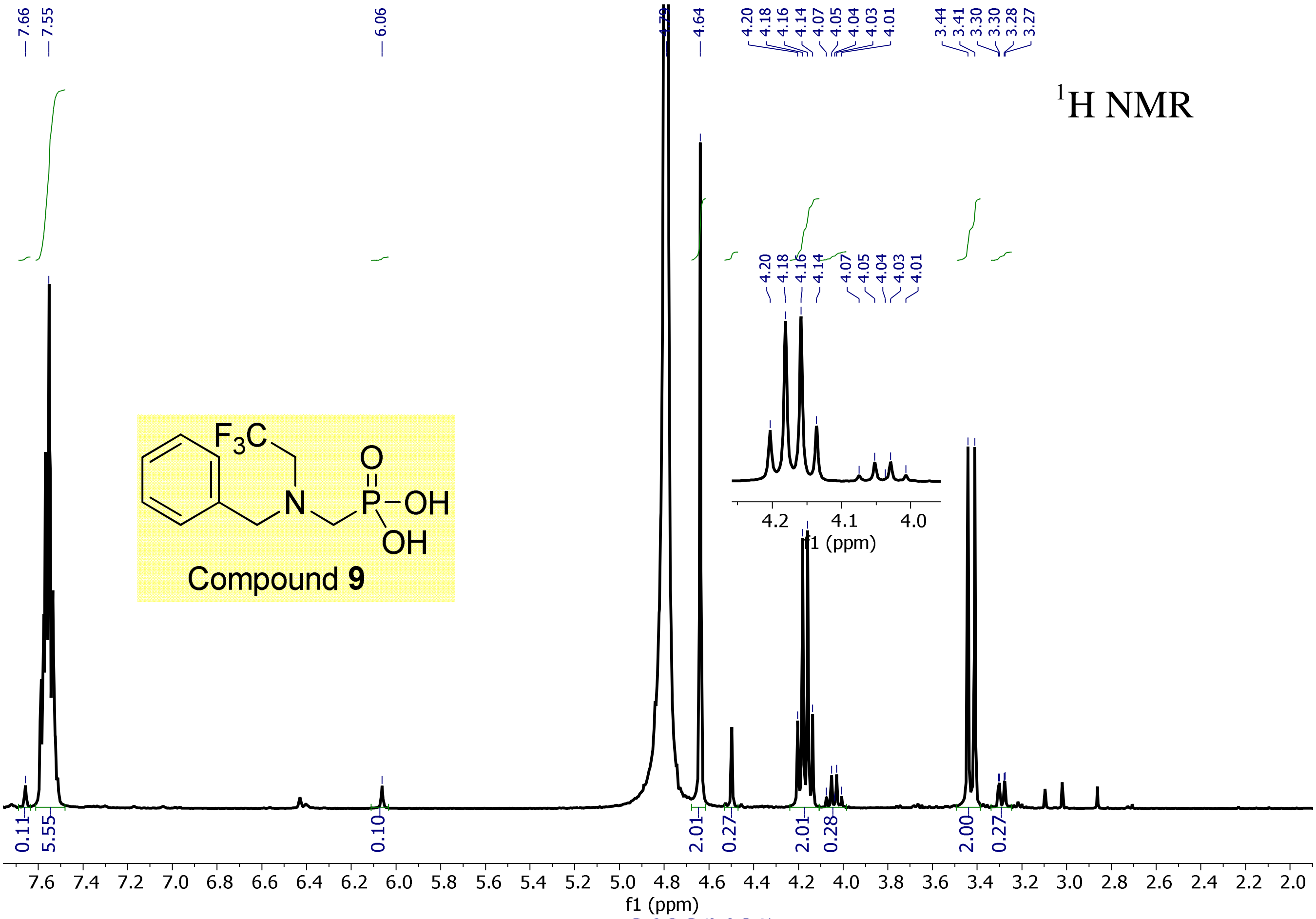

^{1}H NMR
Compound **9**
7.66
7.55
6.06
4.64
4.20
4.18
4.16
4.14
4.07
4.05
4.04
4.03
4.01
3.44
3.41
3.30
3.30
3.28
3.27
0.11
5.55
0.10
2.01
0.27
2.01
0.28
2.00
0.27
f1 (ppm)

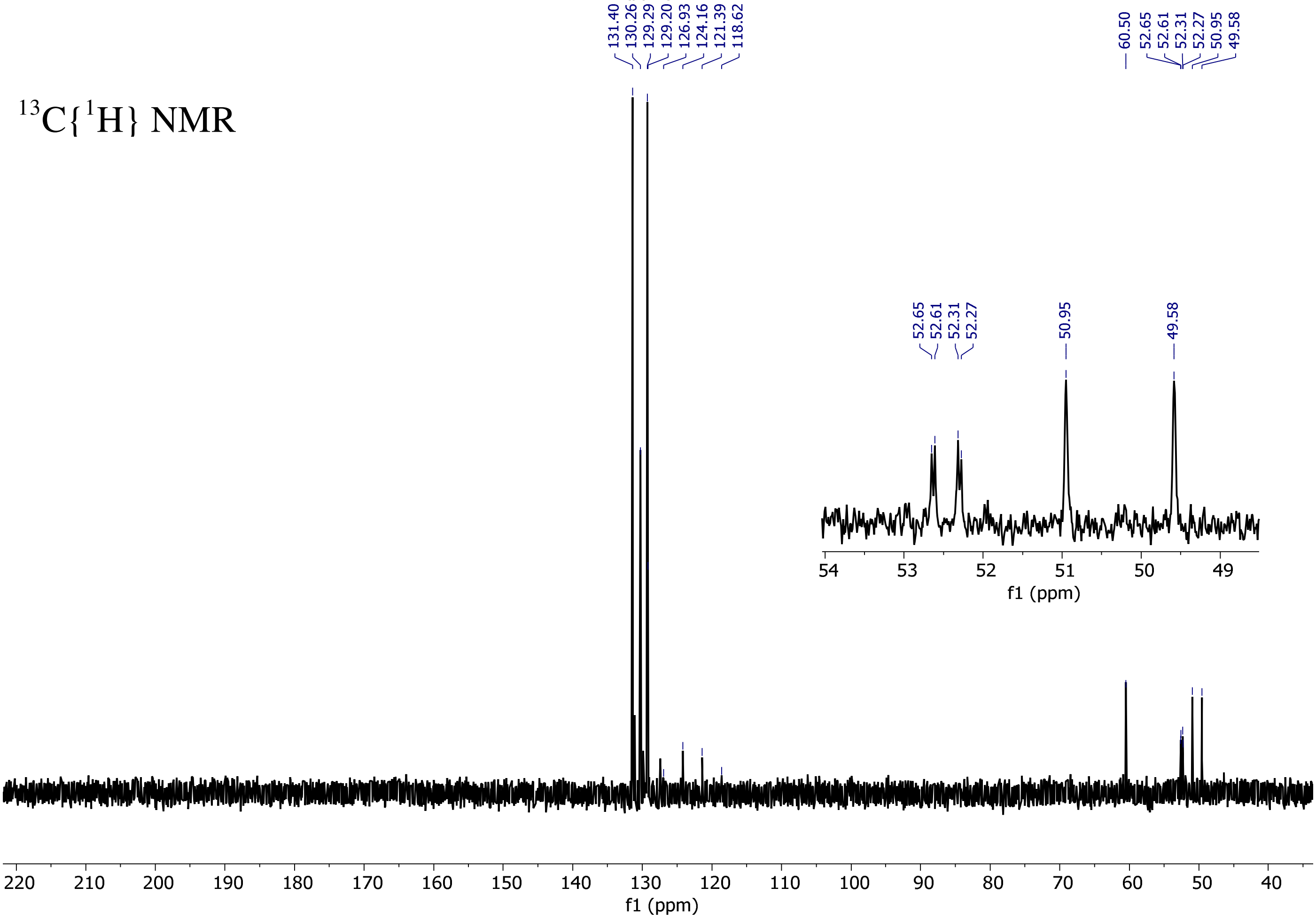

^{13}C{^{1}H} NMR
131.40
130.26
129.29
129.20
126.93
124.16
121.39
118.62
60.50
52.65
52.61
52.31
52.27
50.95
49.58
f1 (ppm)

^{31}P NMR

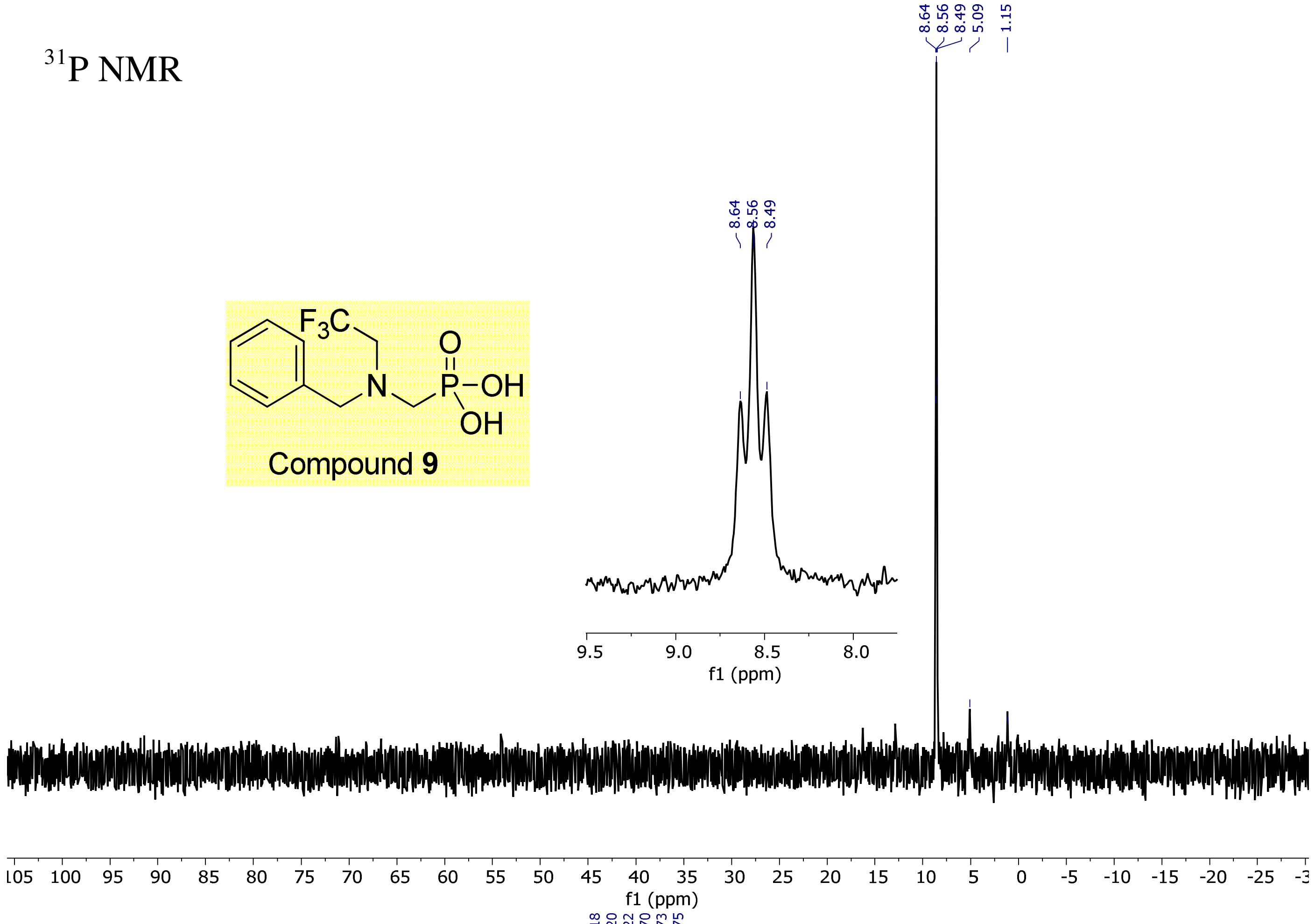

^{19}F NMR

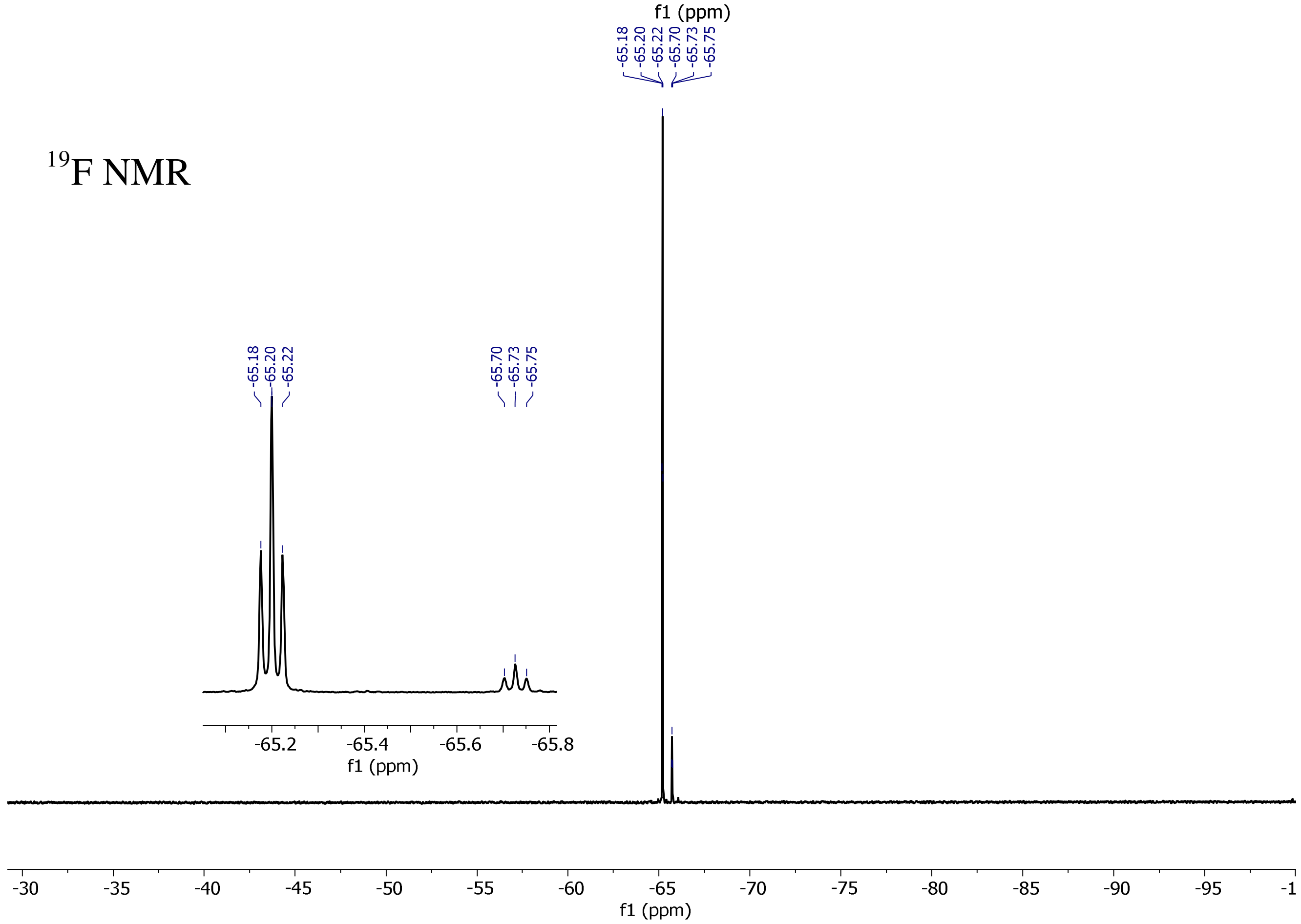

^{1}H NMR

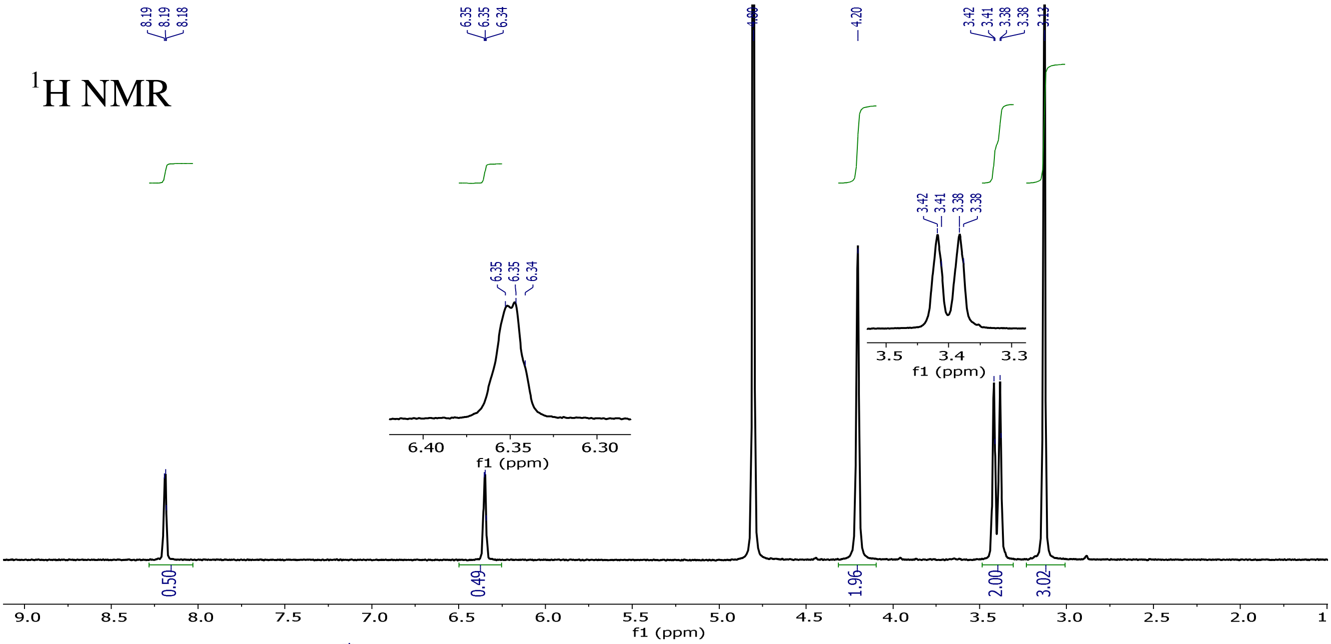

$^{13}C\{^{1}H\}$ NMR

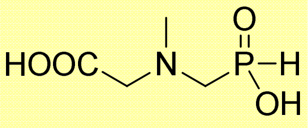

Compound **10**

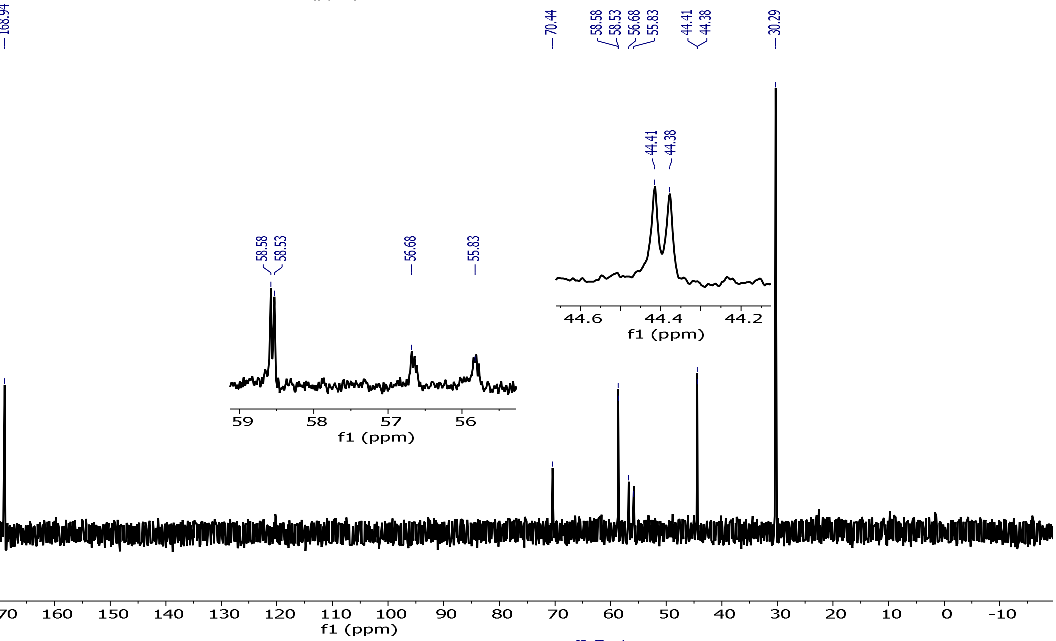

^{31}P NMR

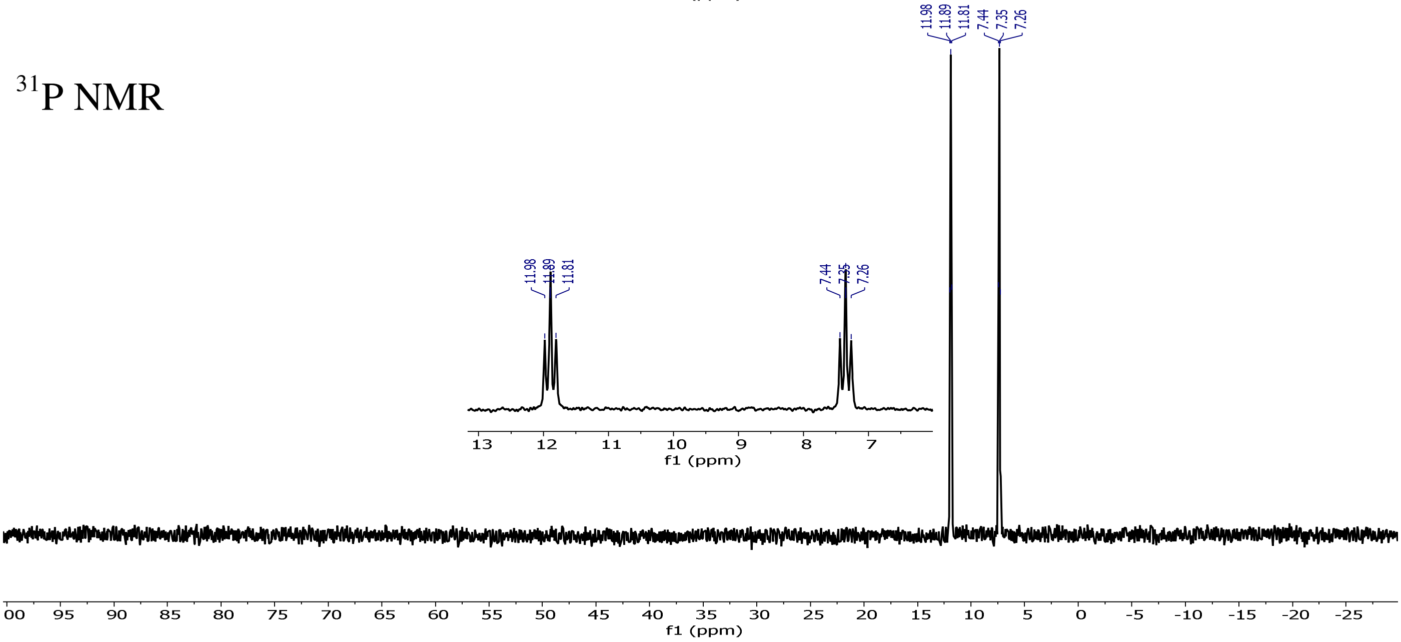

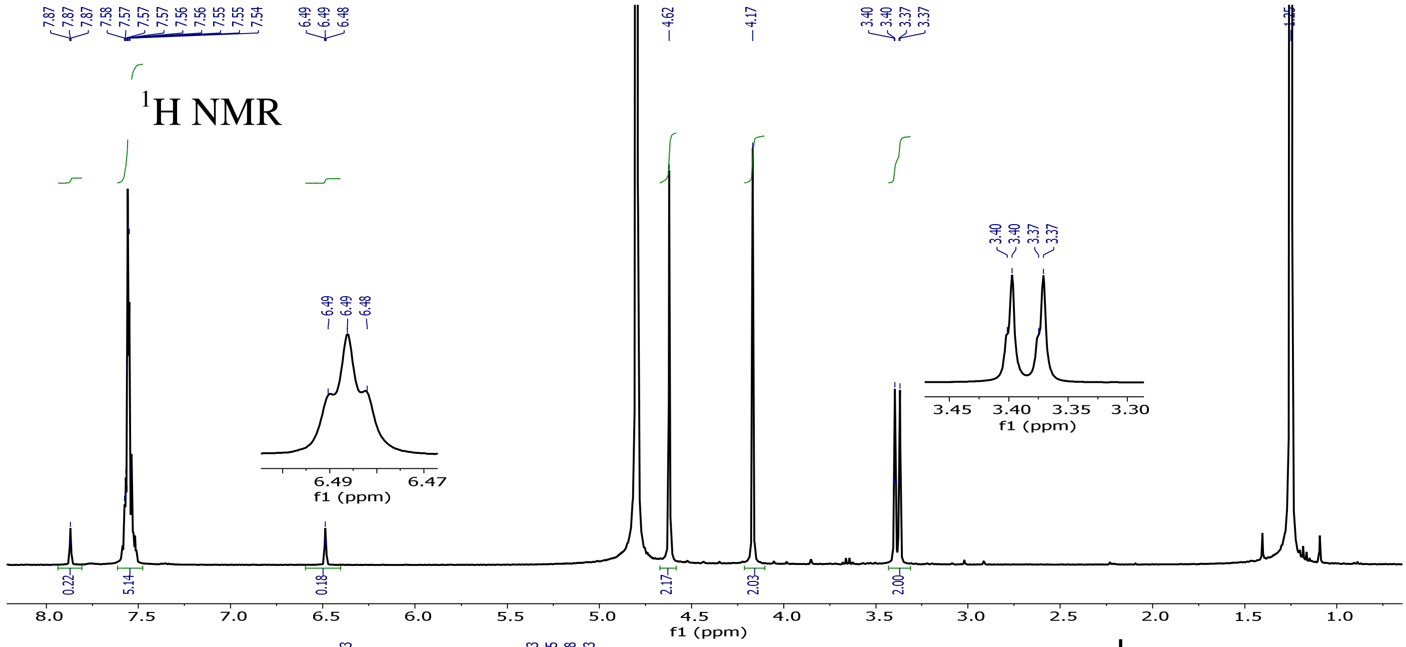

^{1}H NMR
7.87 7.87 7.87 7.58 7.57 7.57 7.57 7.56 7.56 7.55 7.55 7.54
6.49 6.49 6.48
4.62
4.17
3.40 3.40 3.37 3.37
1.26
0.22 5.14 0.18 2.17 2.03 2.00
f1 (ppm)

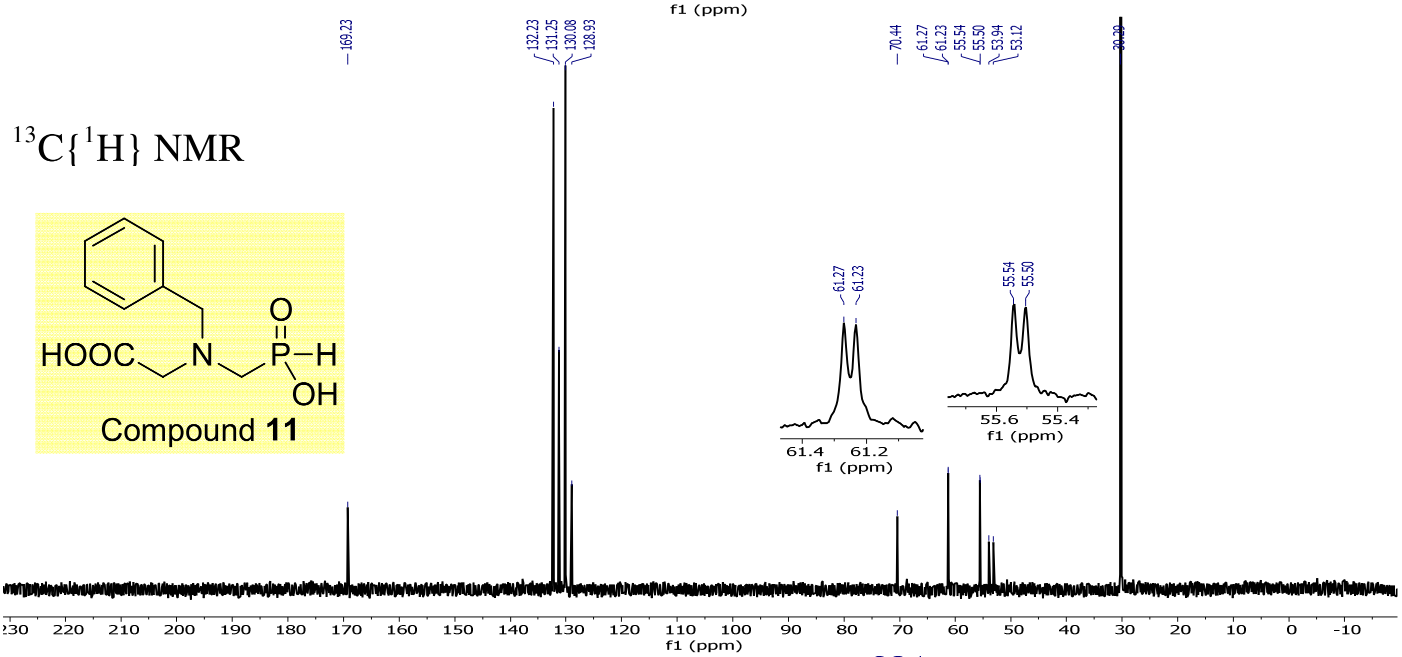

^{13}C{^{1}H} NMR
169.23
132.23 131.25 130.08 128.93
70.44
61.27 61.23 55.54 55.50 53.94 53.12
30.29
HOOC N P H O OH
Compound 11
f1 (ppm)

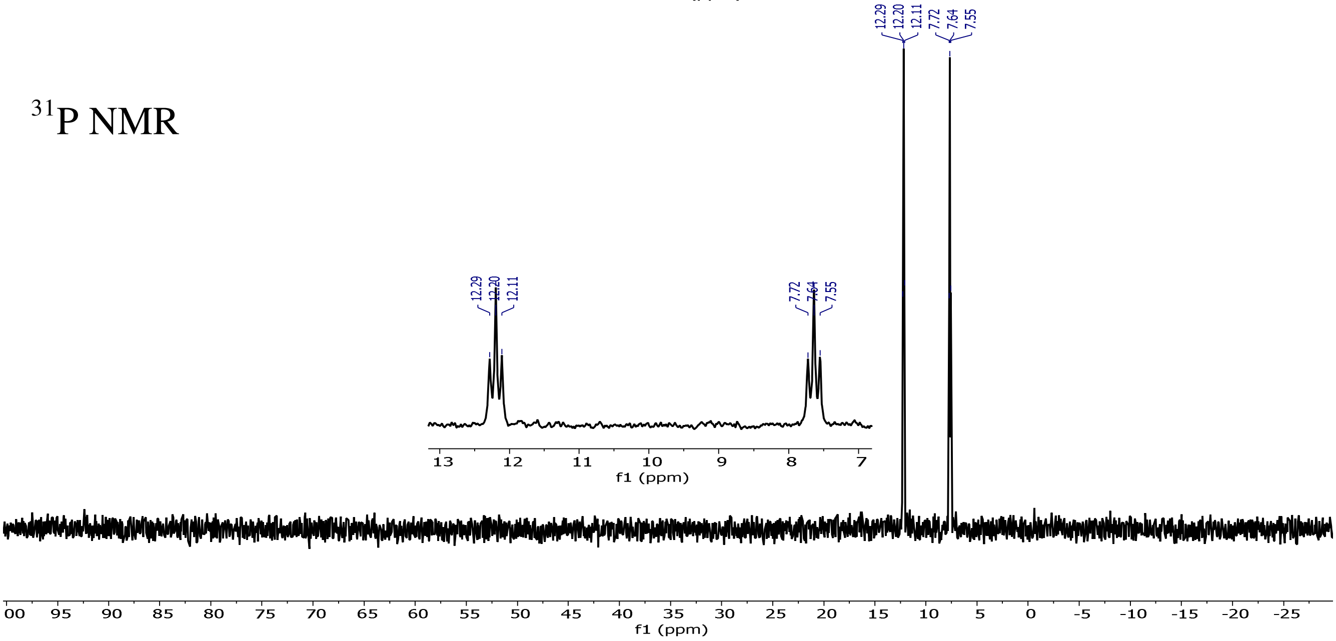

^{31}P NMR
12.29 12.20 12.11 7.72 7.64 7.55
f1 (ppm)

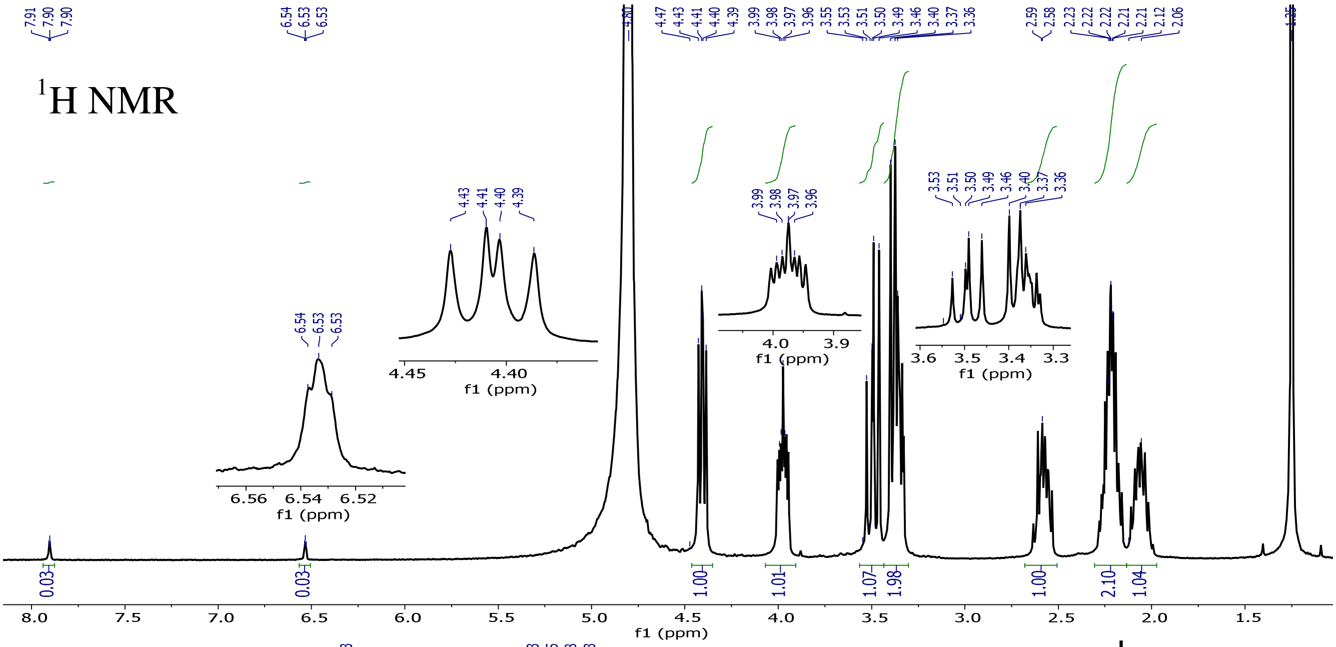

^{1}H NMR

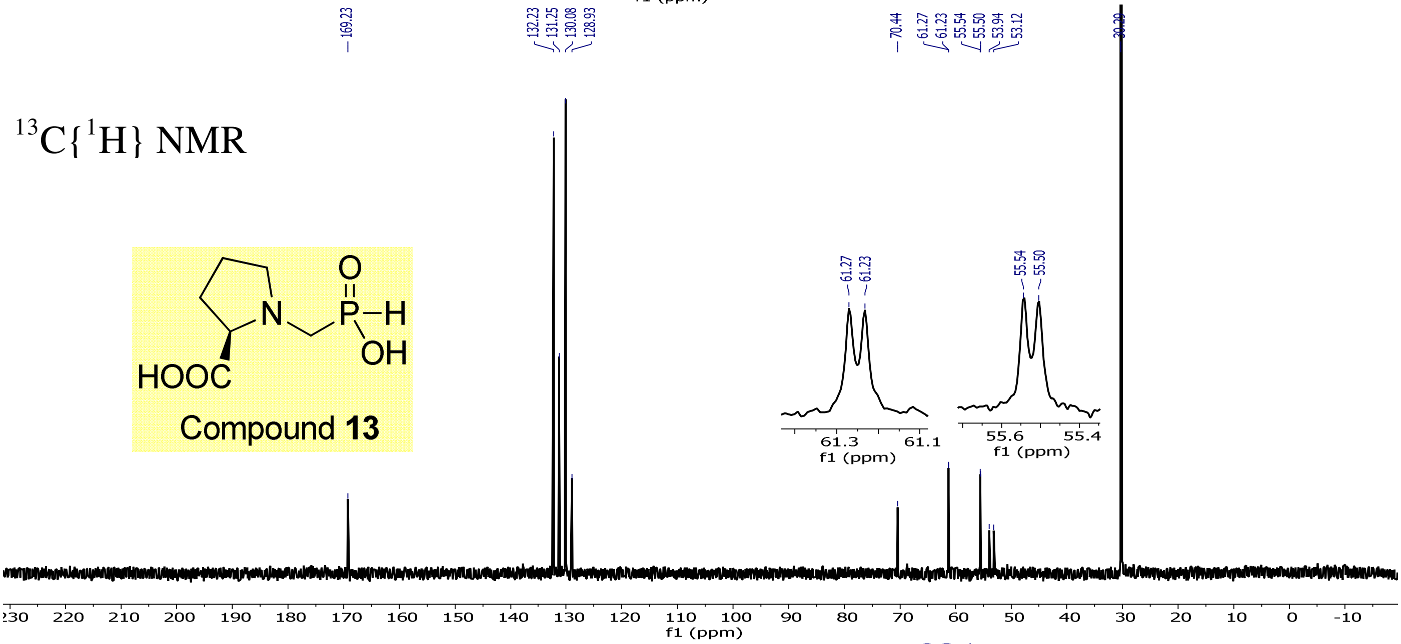

^{13}C{^{1}H} NMR
HOOC
OH
Compound **13**

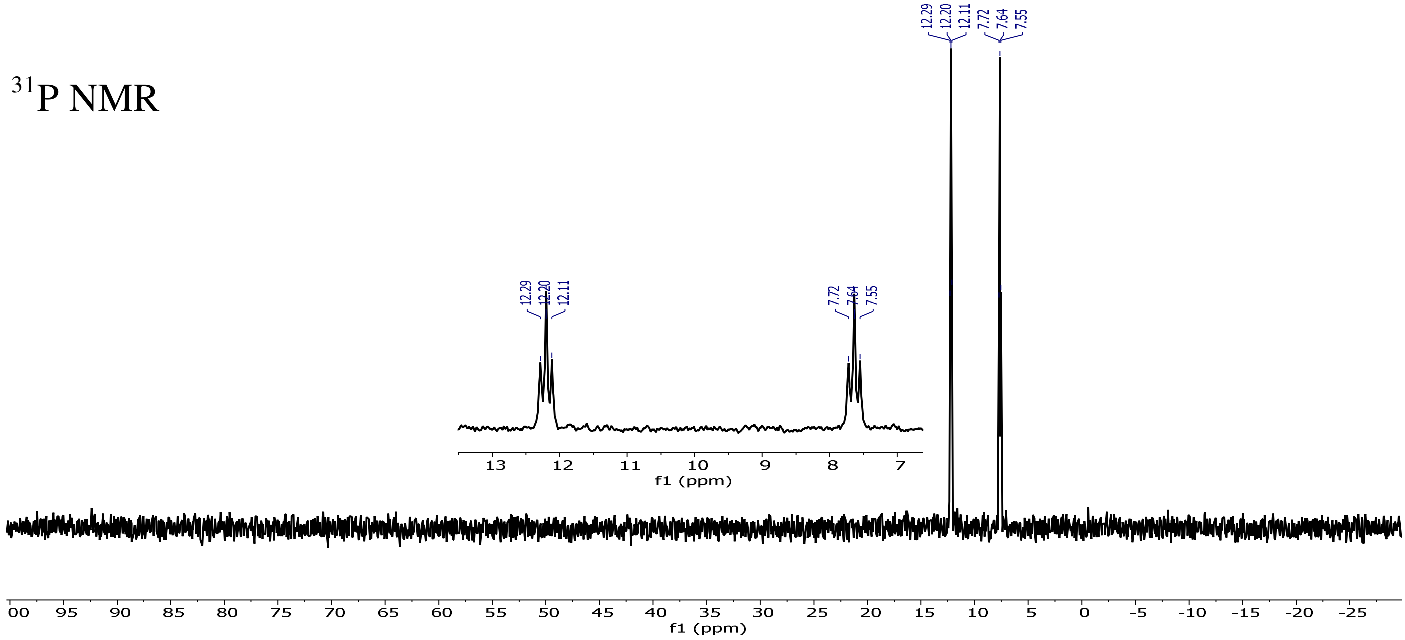

^{31}P NMR

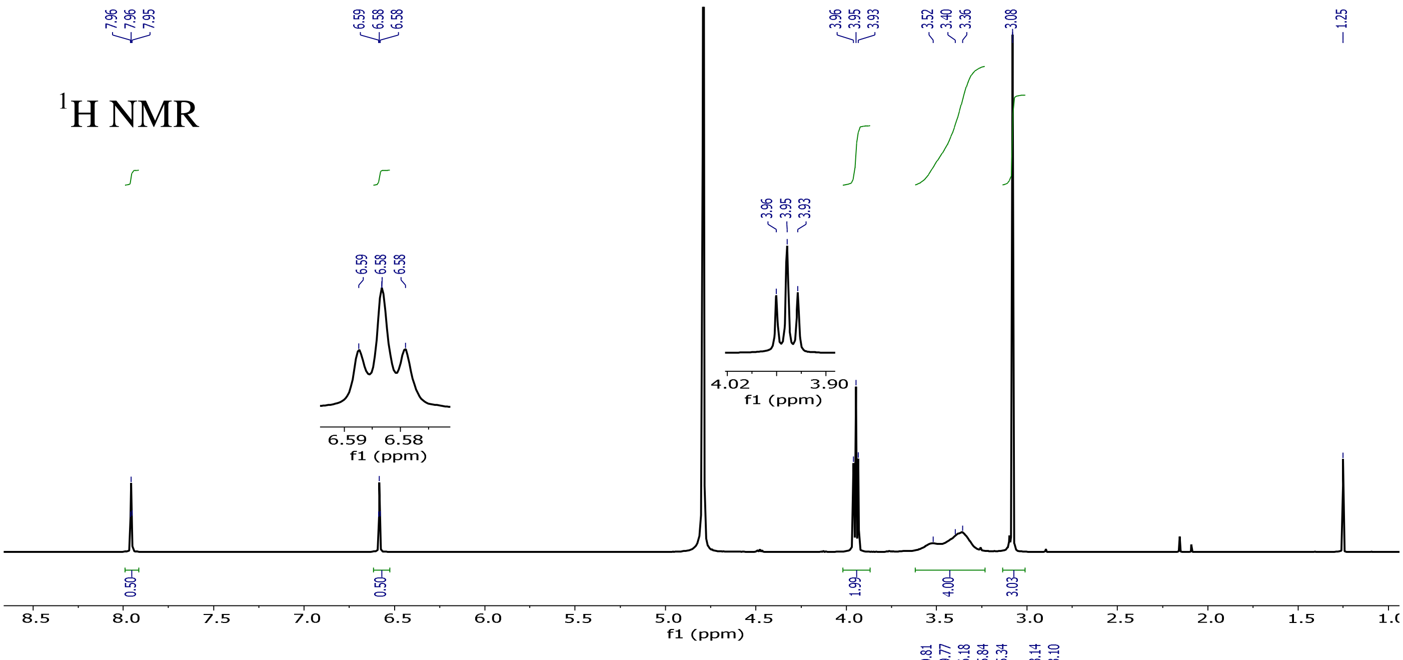
1H NMR
f1 (ppm)

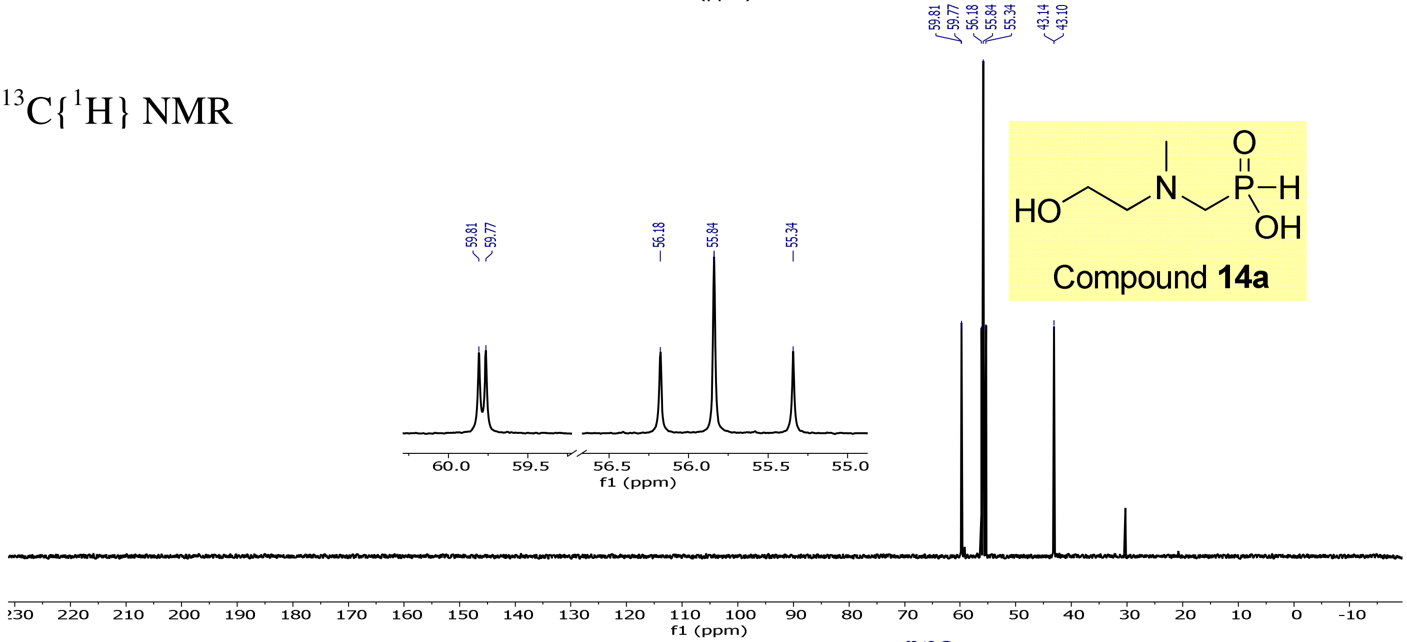
13C{1H} NMR
f1 (ppm)
HO
N
P
O
H
OH
Compound **14a**

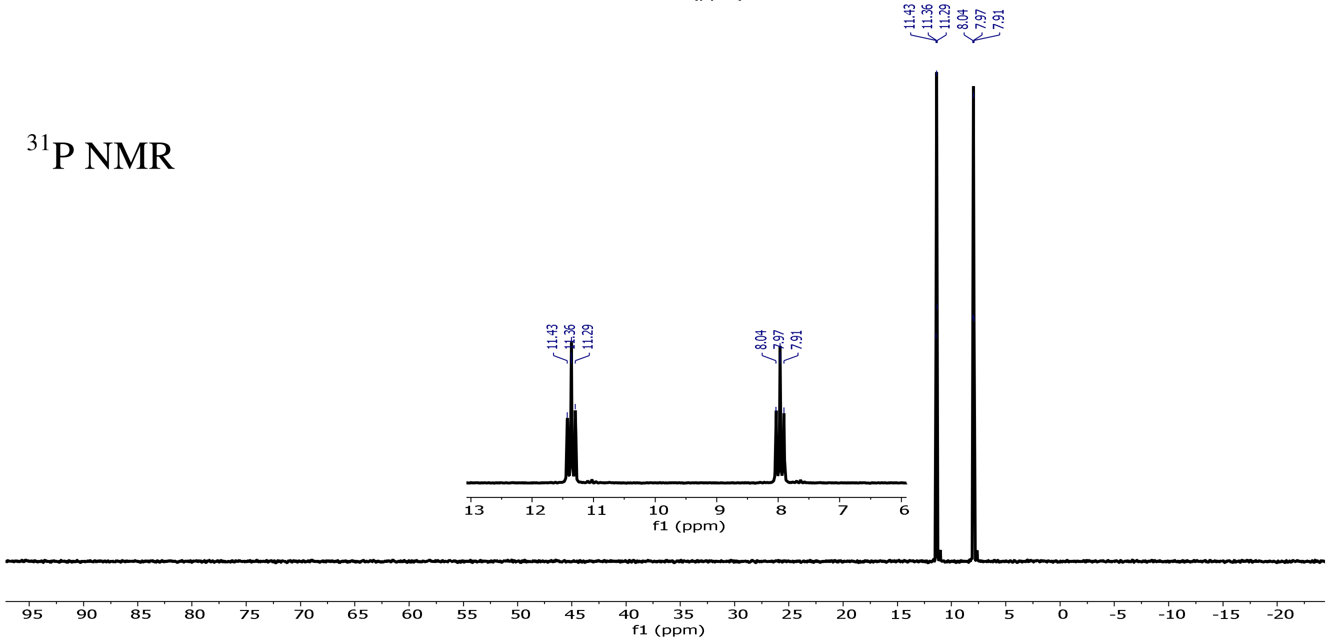
31P NMR
f1 (ppm)

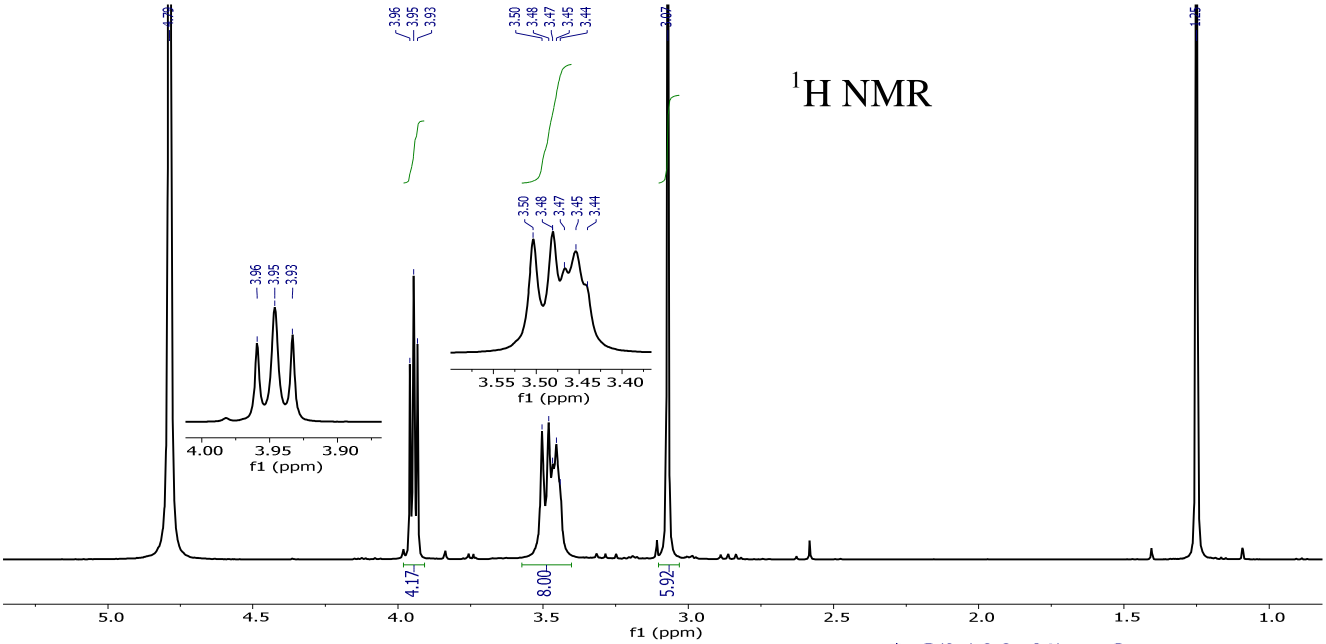

^{1}H NMR
4.79
3.96 3.95 3.93
3.50 3.48 3.47 3.45 3.44
3.07
1.25
4.17
8.00
5.92
f1 (ppm)

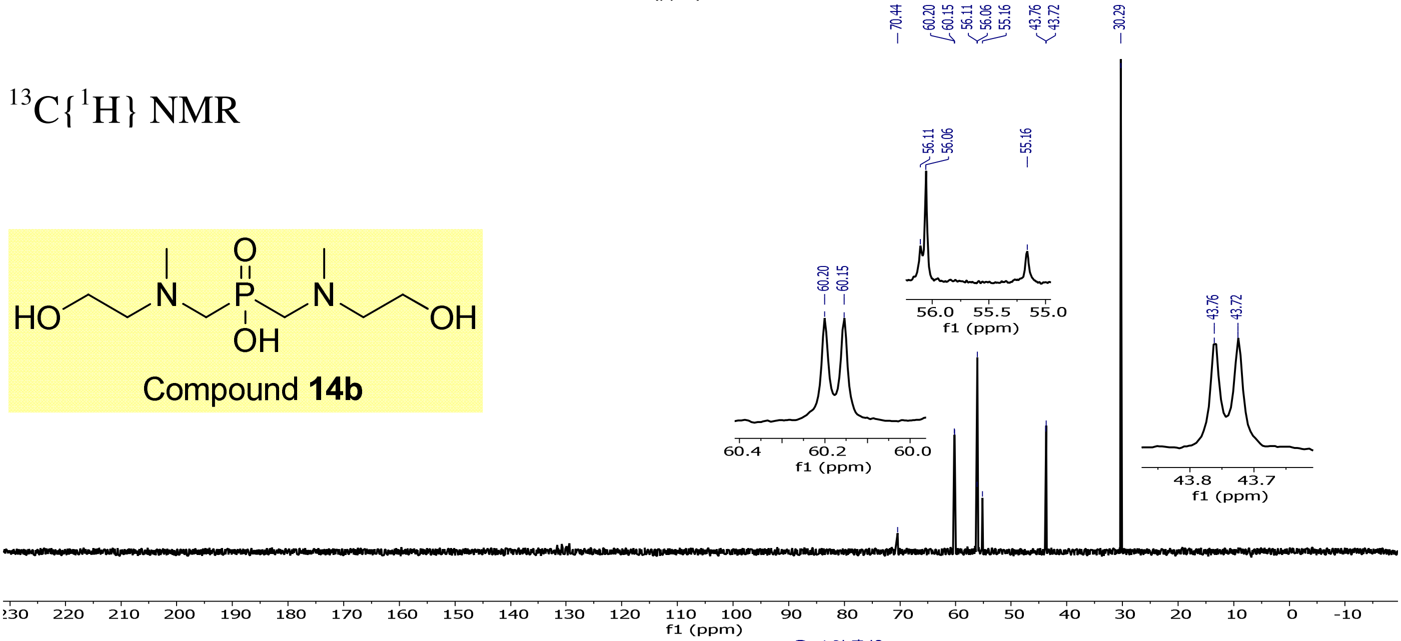

^{13}C{^{1}H} NMR
70.44
60.20 60.15
56.11 56.06 55.16
43.76 43.72
30.29
HO
N
P
O
OH
N
OH
Compound 14b
f1 (ppm)

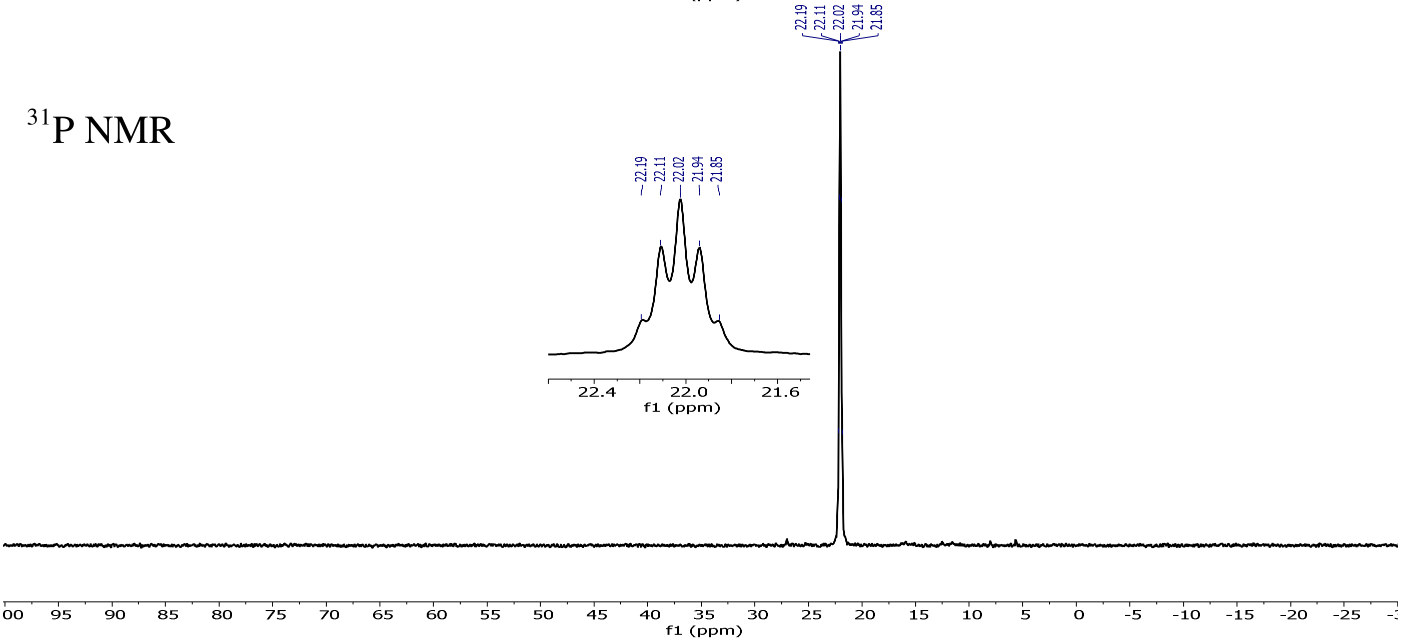

^{31}P NMR
22.19 22.11 22.02 21.94 21.85
f1 (ppm)

^{1}H NMR

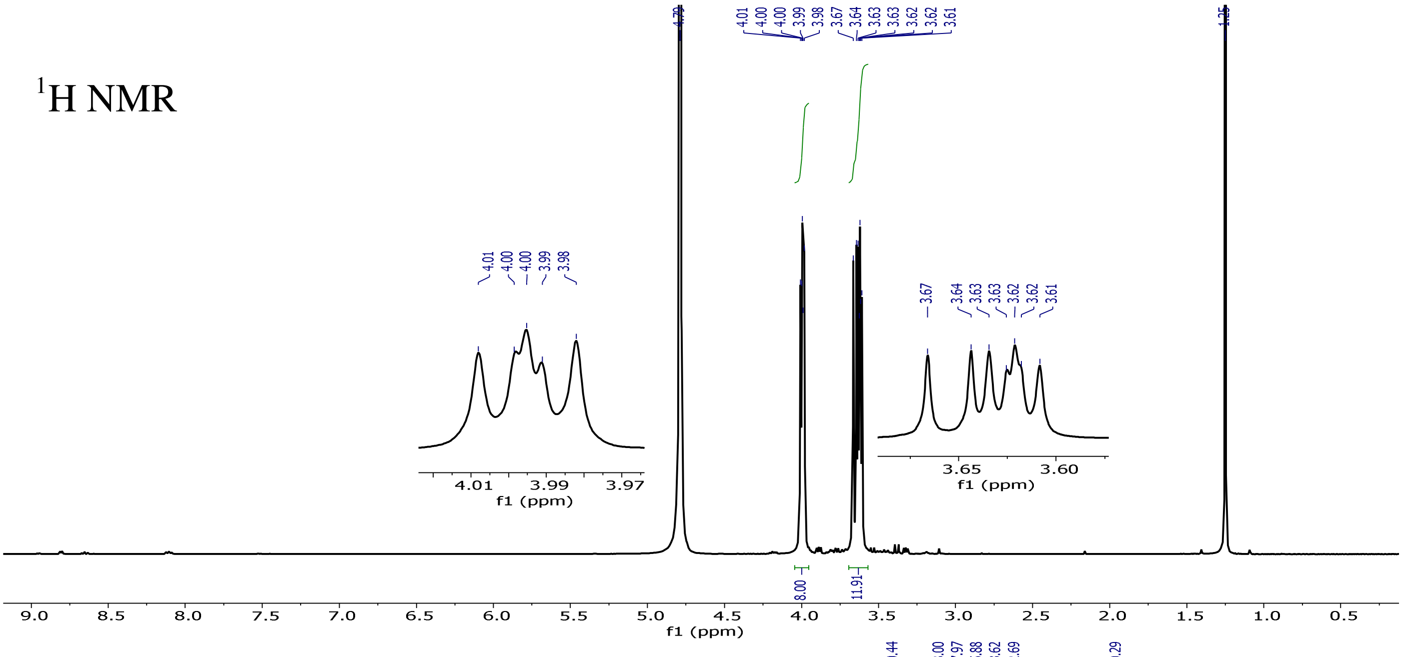

^{13}C{^{1}H} NMR

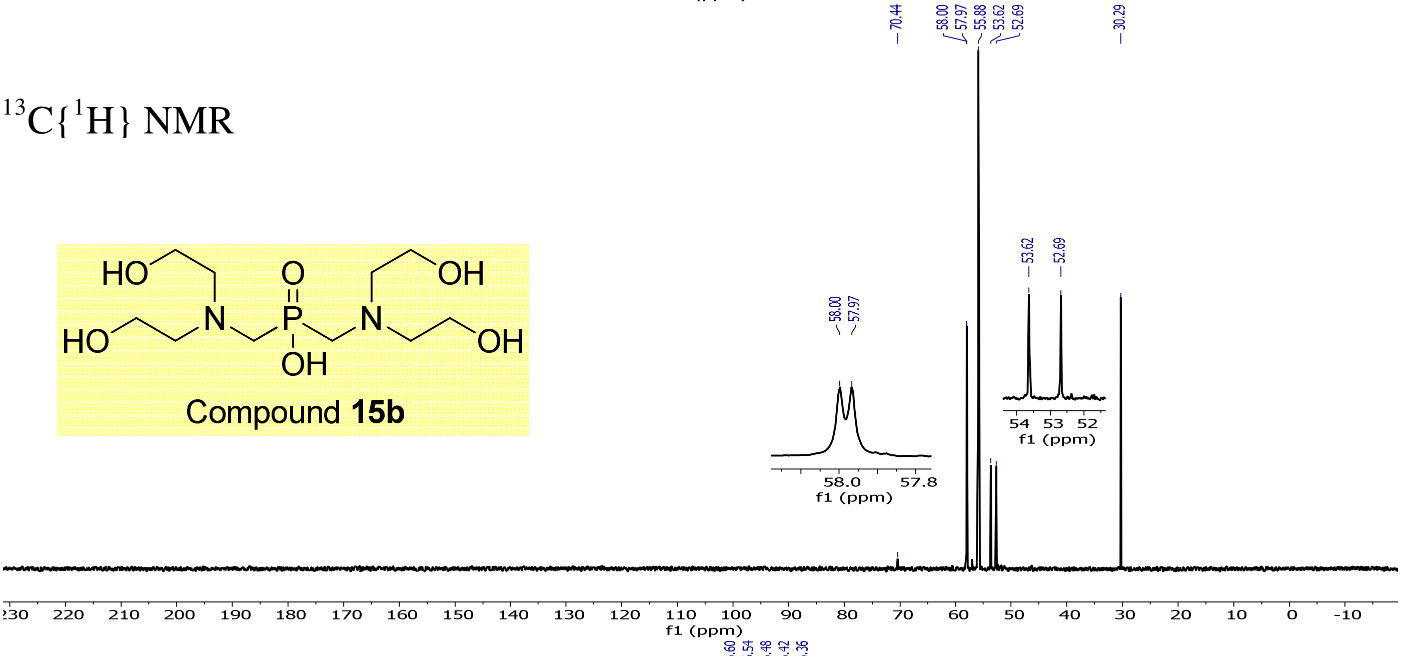

Compound **15b**

^{31}P NMR

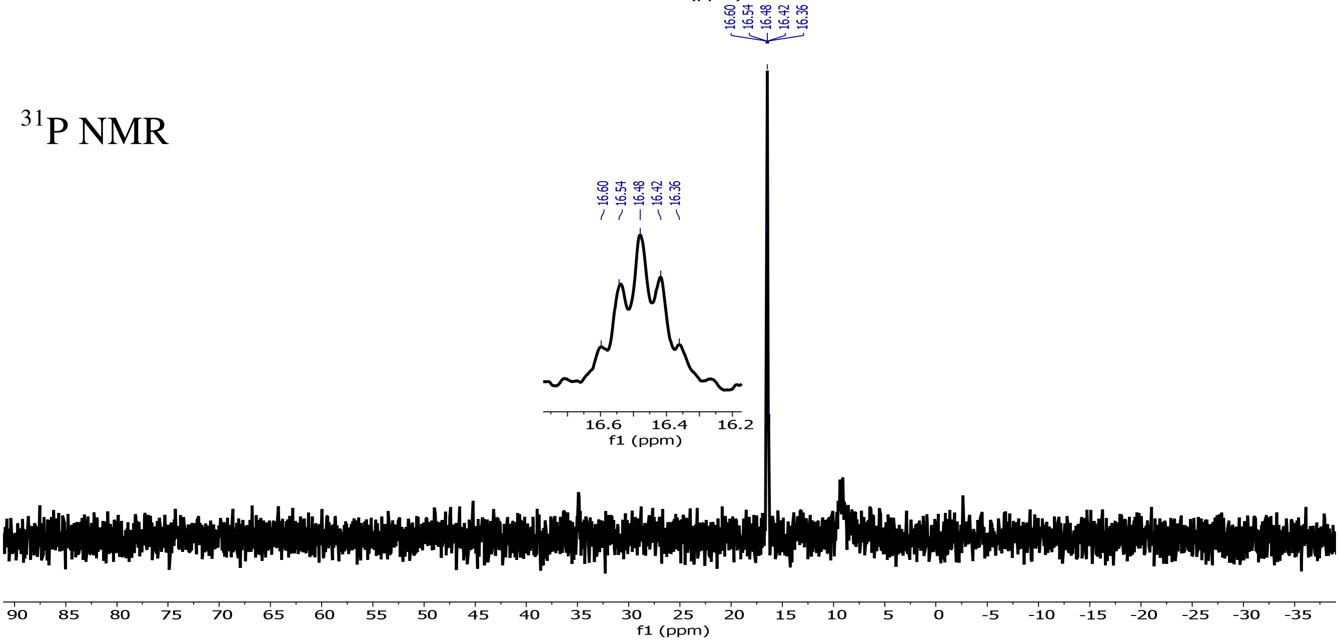

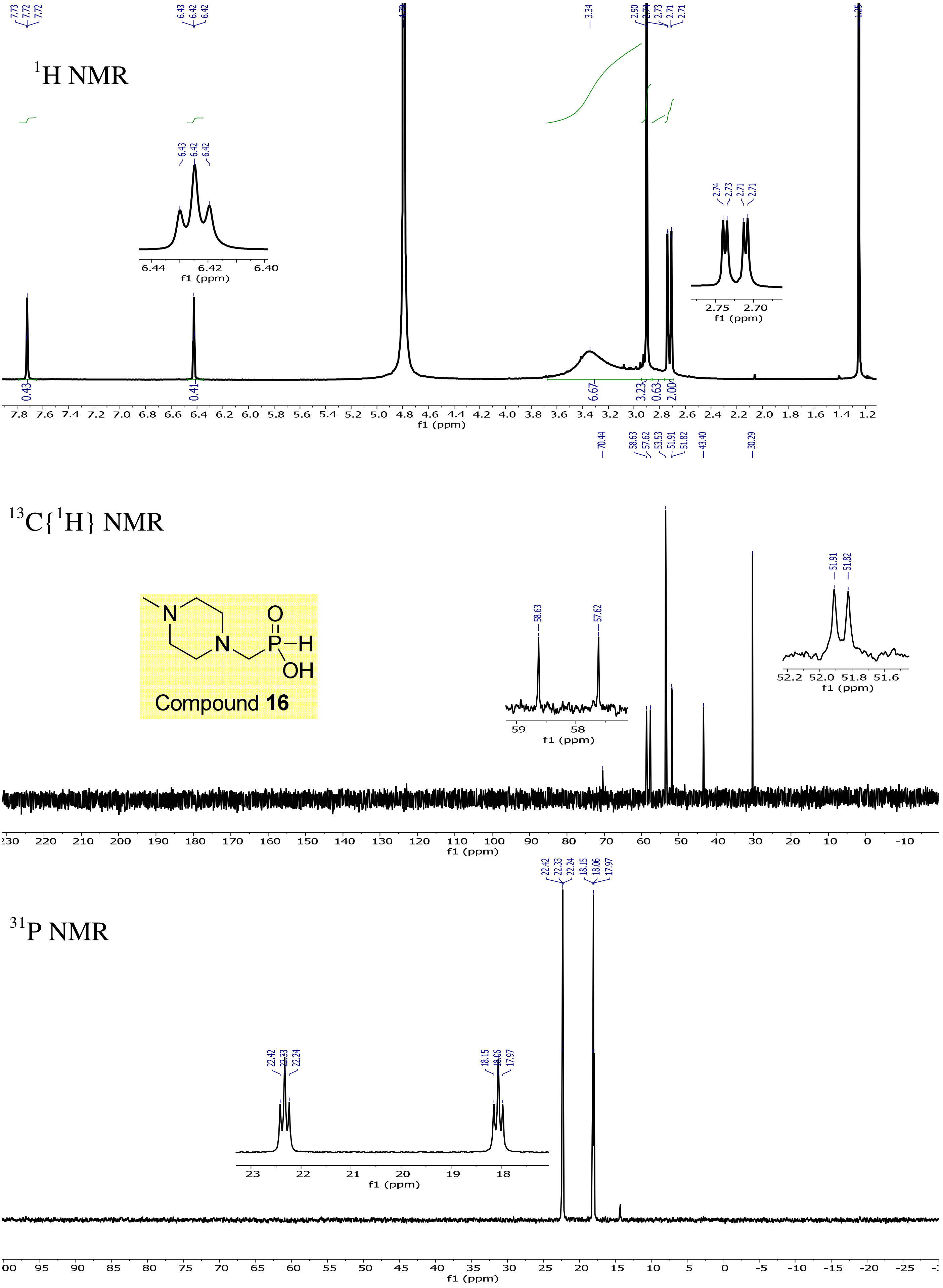

¹H NMR
¹³C{¹H} NMR
Compound 16
³¹P NMR
f1 (ppm)

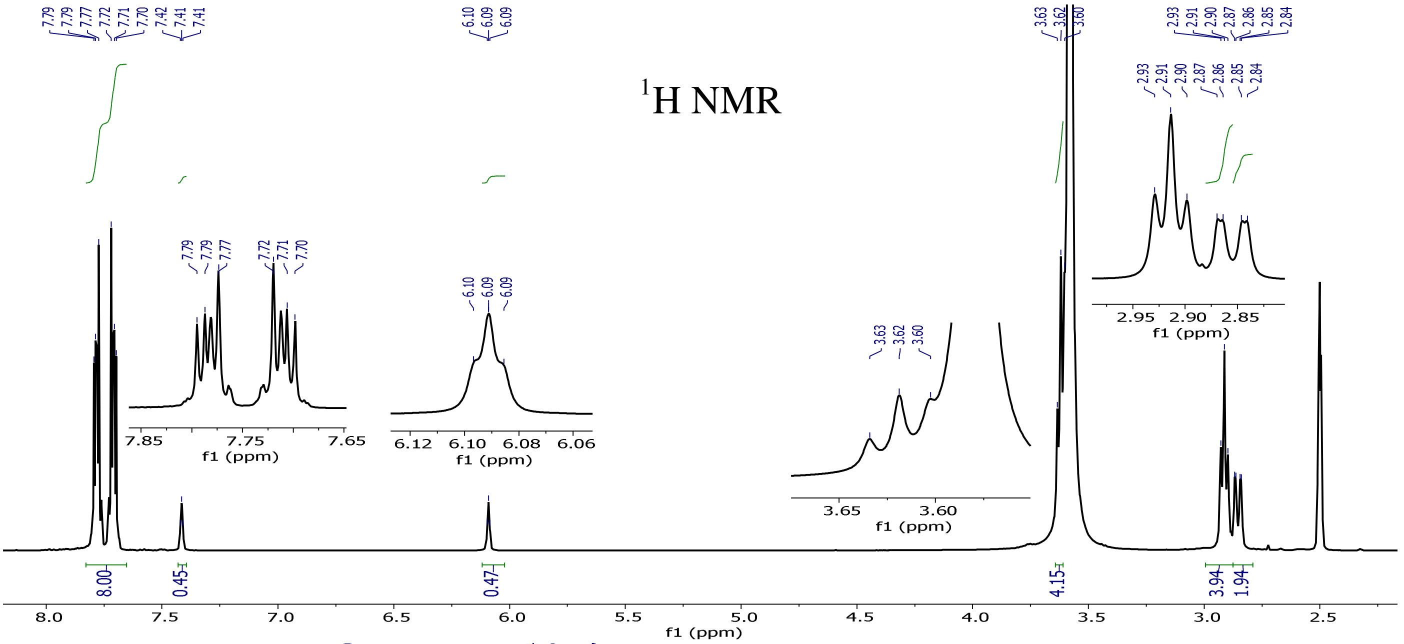
1H NMR
7.79 7.79 7.77 7.72 7.71 7.70 7.42 7.41 7.41
6.10 6.09 6.09
3.63 3.62 3.60
2.93 2.91 2.90 2.87 2.86 2.85 2.84
f1 (ppm)

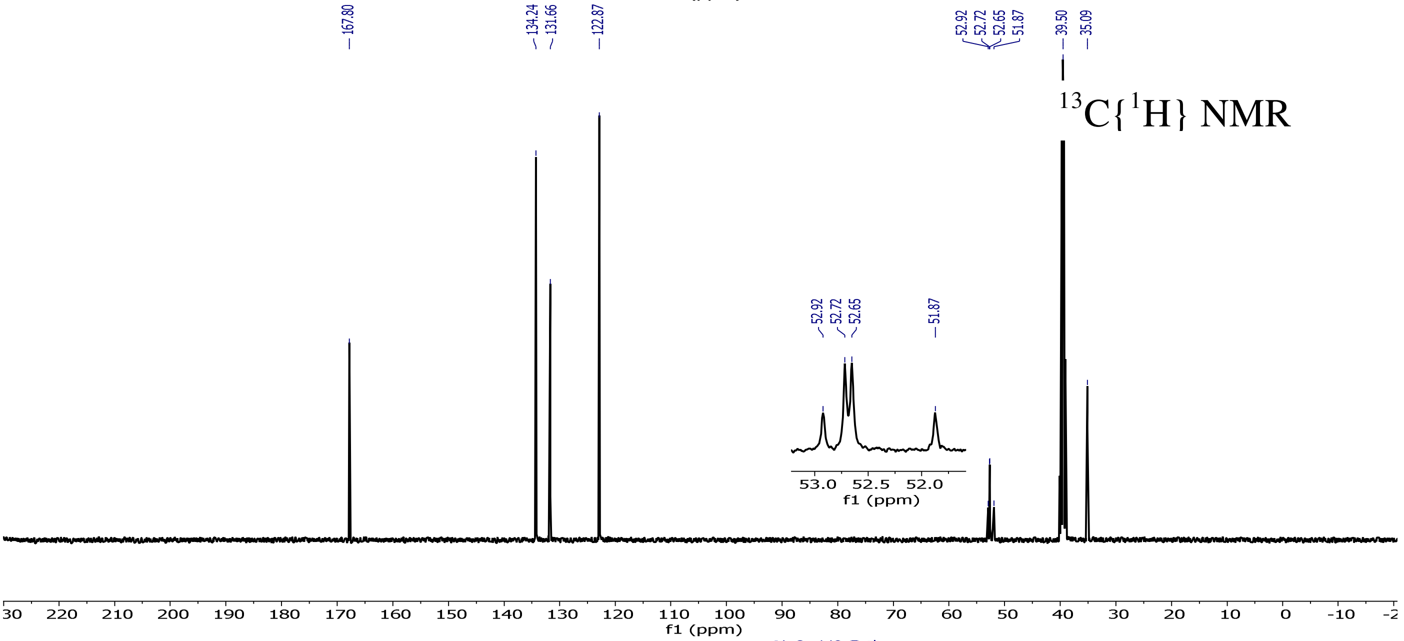
13C{1H} NMR
167.80
134.24 131.66
122.87
52.92 52.72 52.65 51.87
39.50
35.09
f1 (ppm)

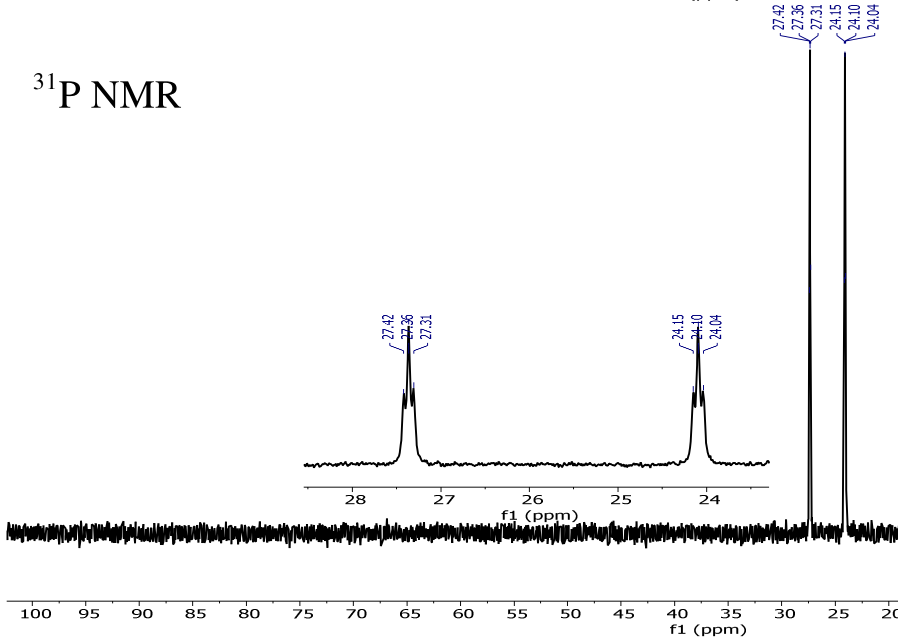
31P NMR
27.42 27.36 27.31 24.15 24.10 24.04
f1 (ppm)

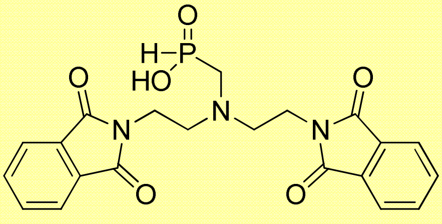

Compound **17**

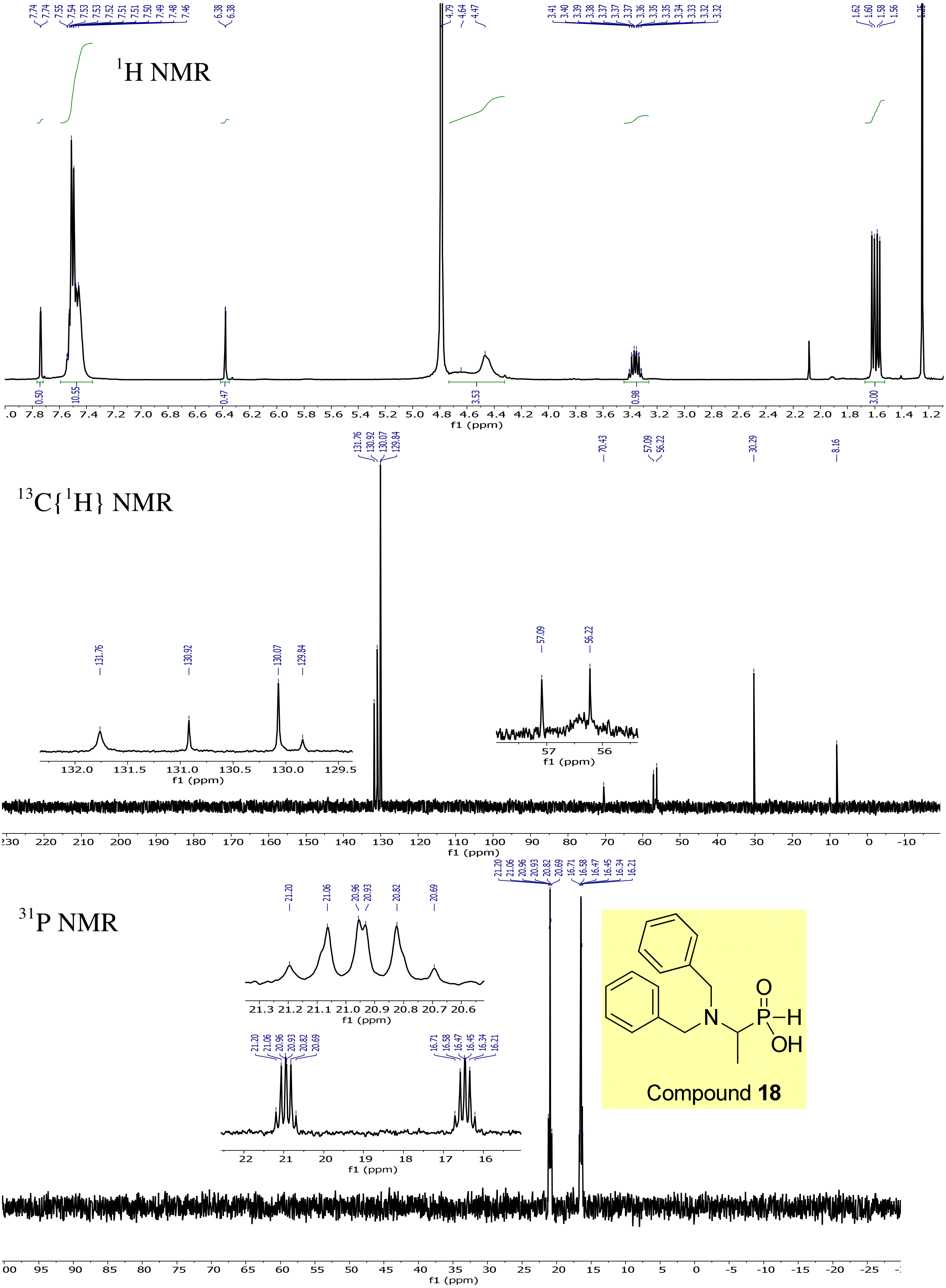

Compound **18**

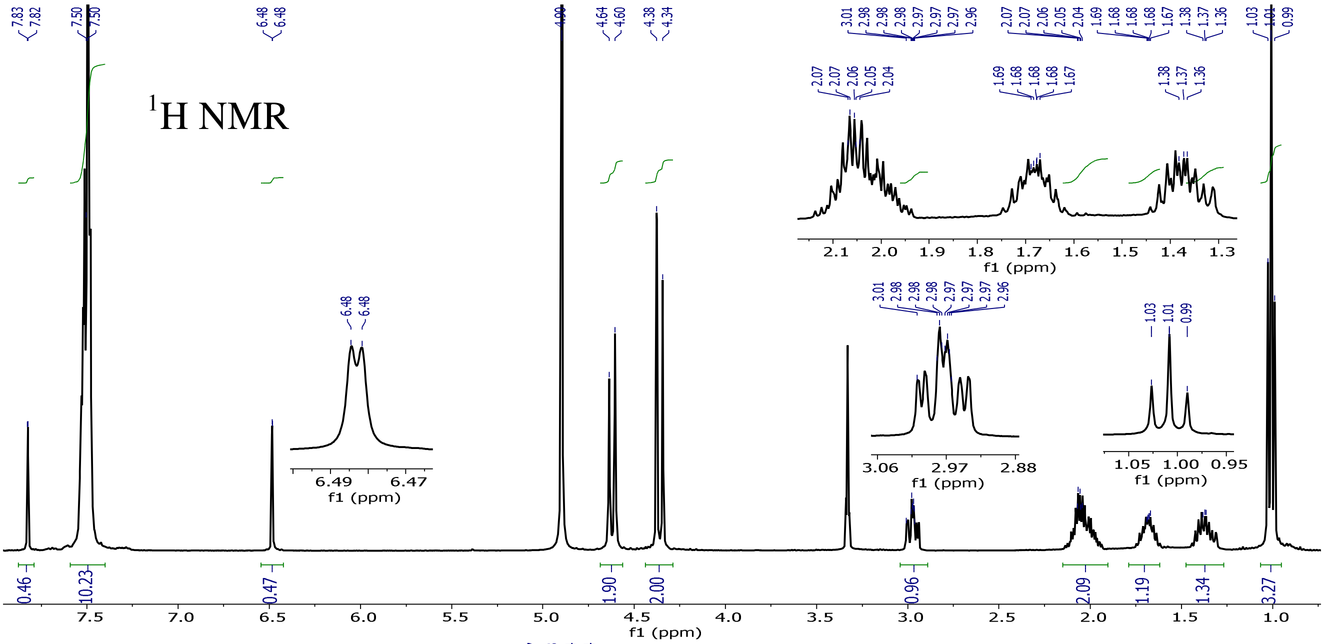

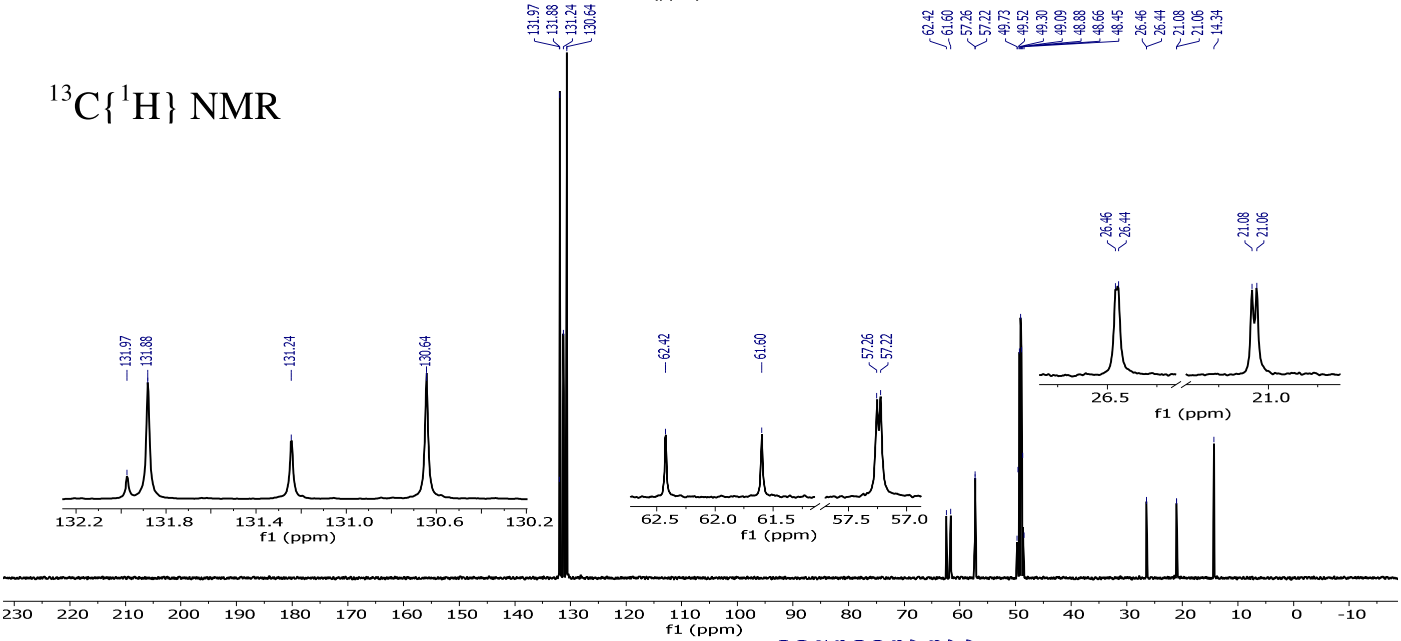

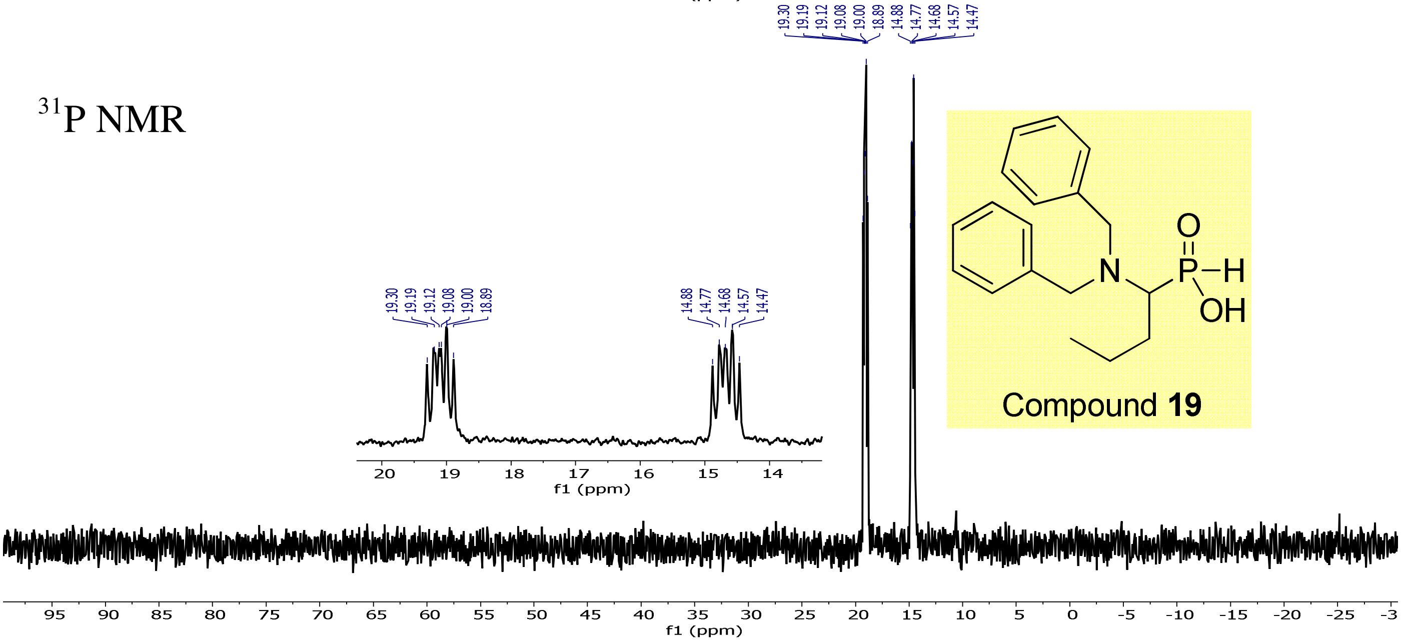

Compound **19**

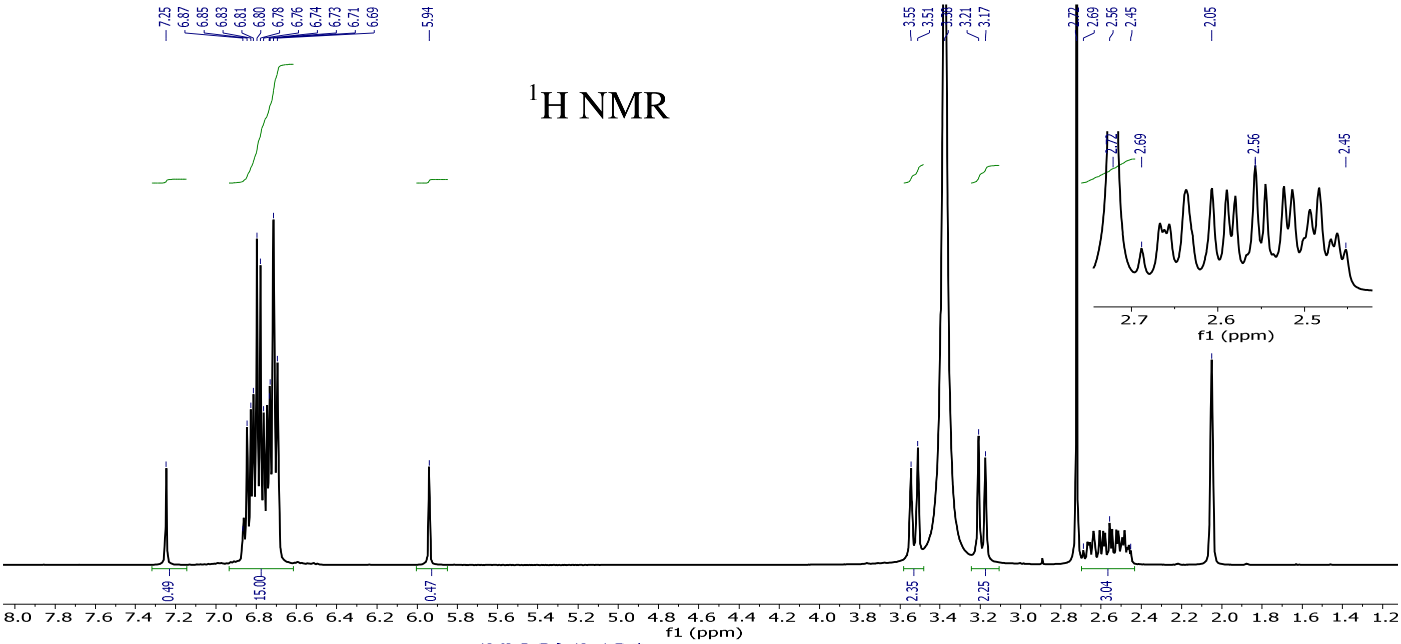

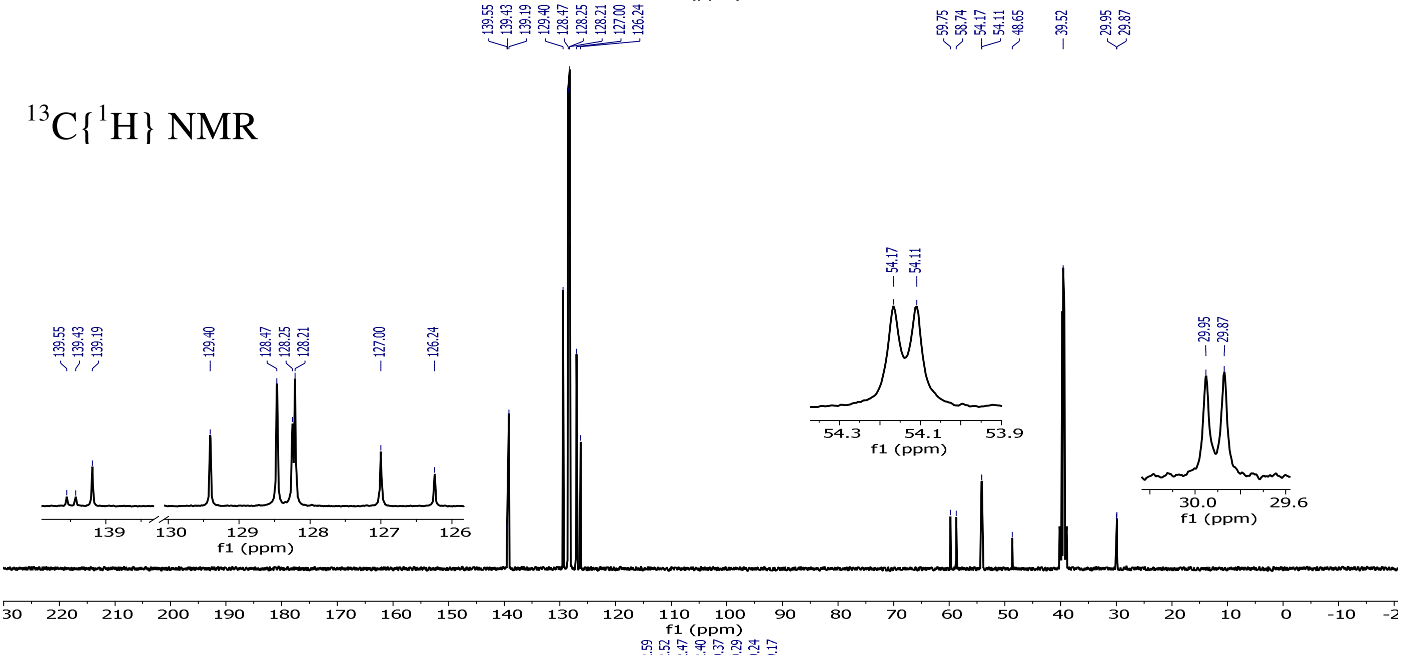

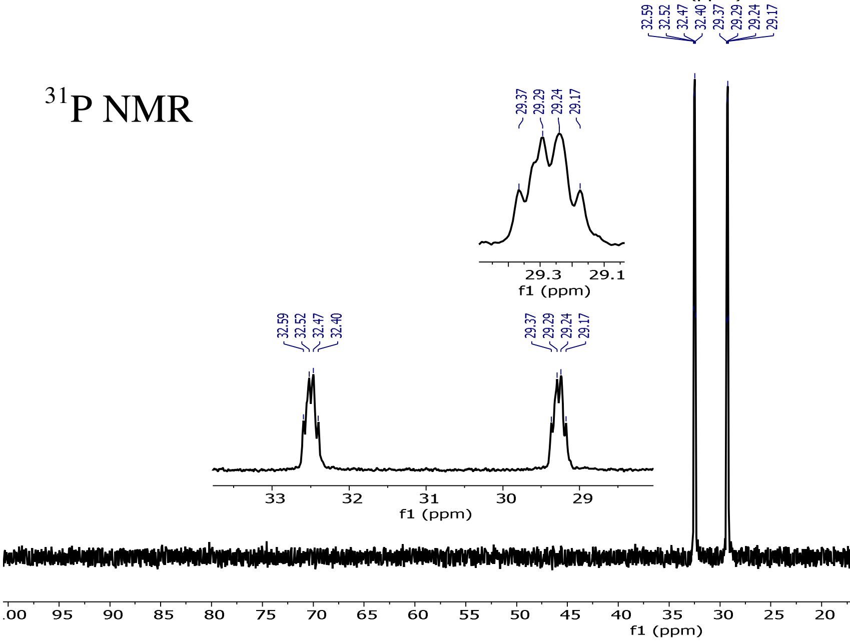

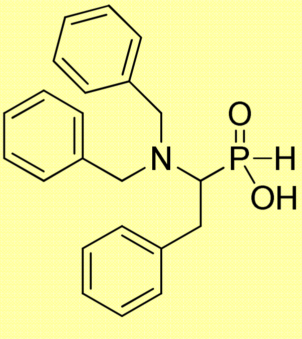

Compound **20**

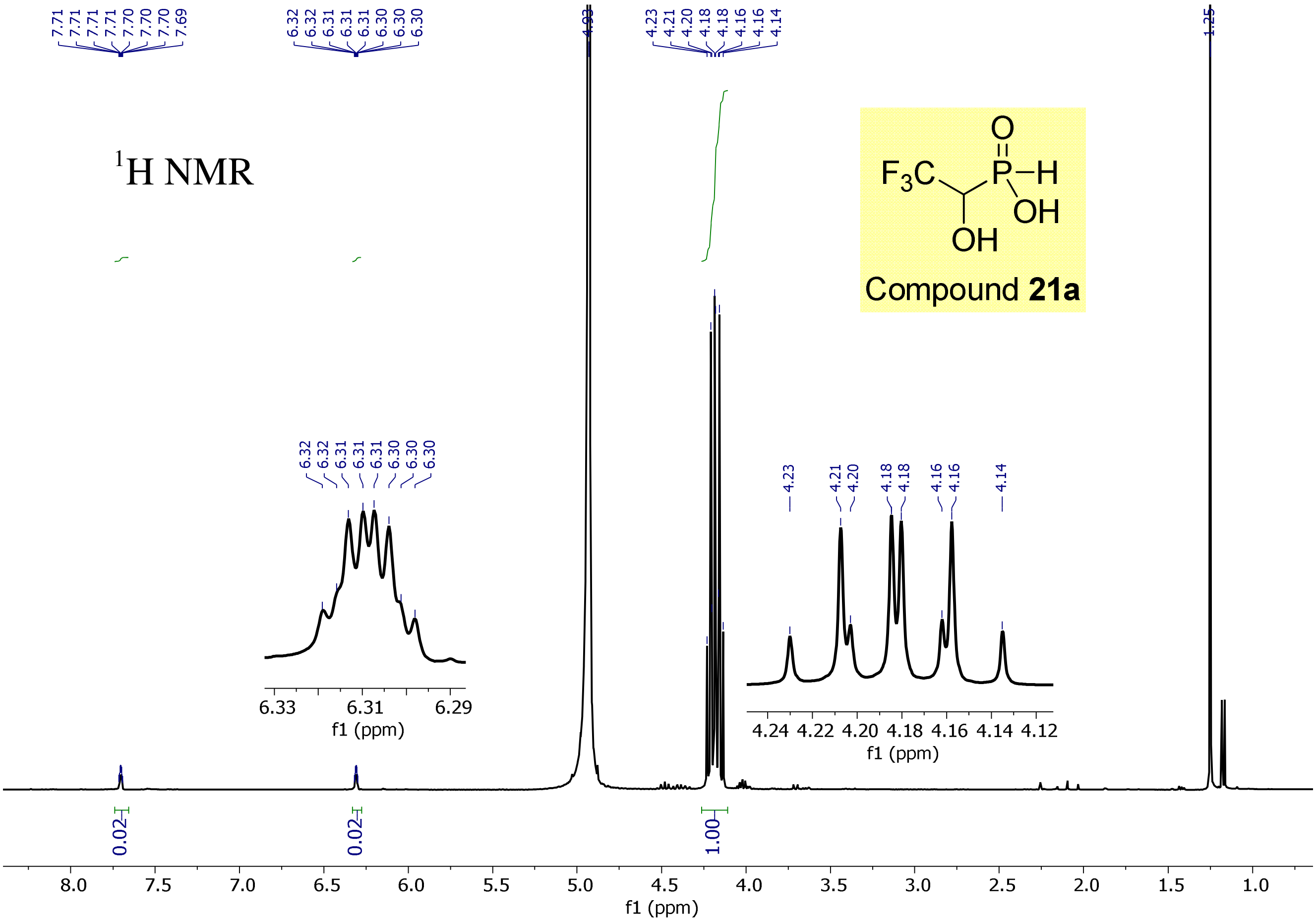
^{1}H NMR
Compound **21a**
F_3C
OH
O
P–H
OH
0.02
0.02
1.00
f1 (ppm)

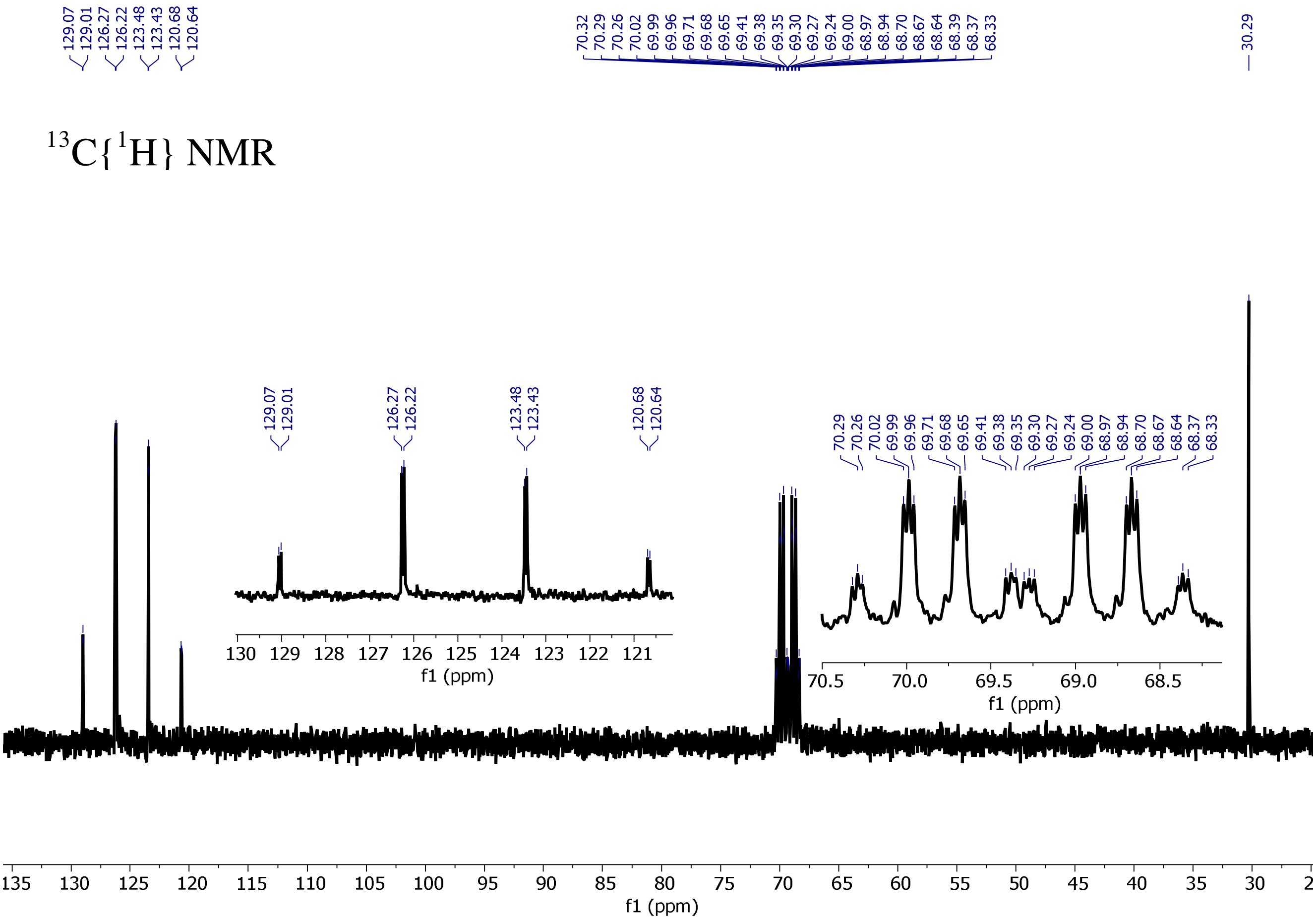
^{13}C{^{1}H} NMR
f1 (ppm)

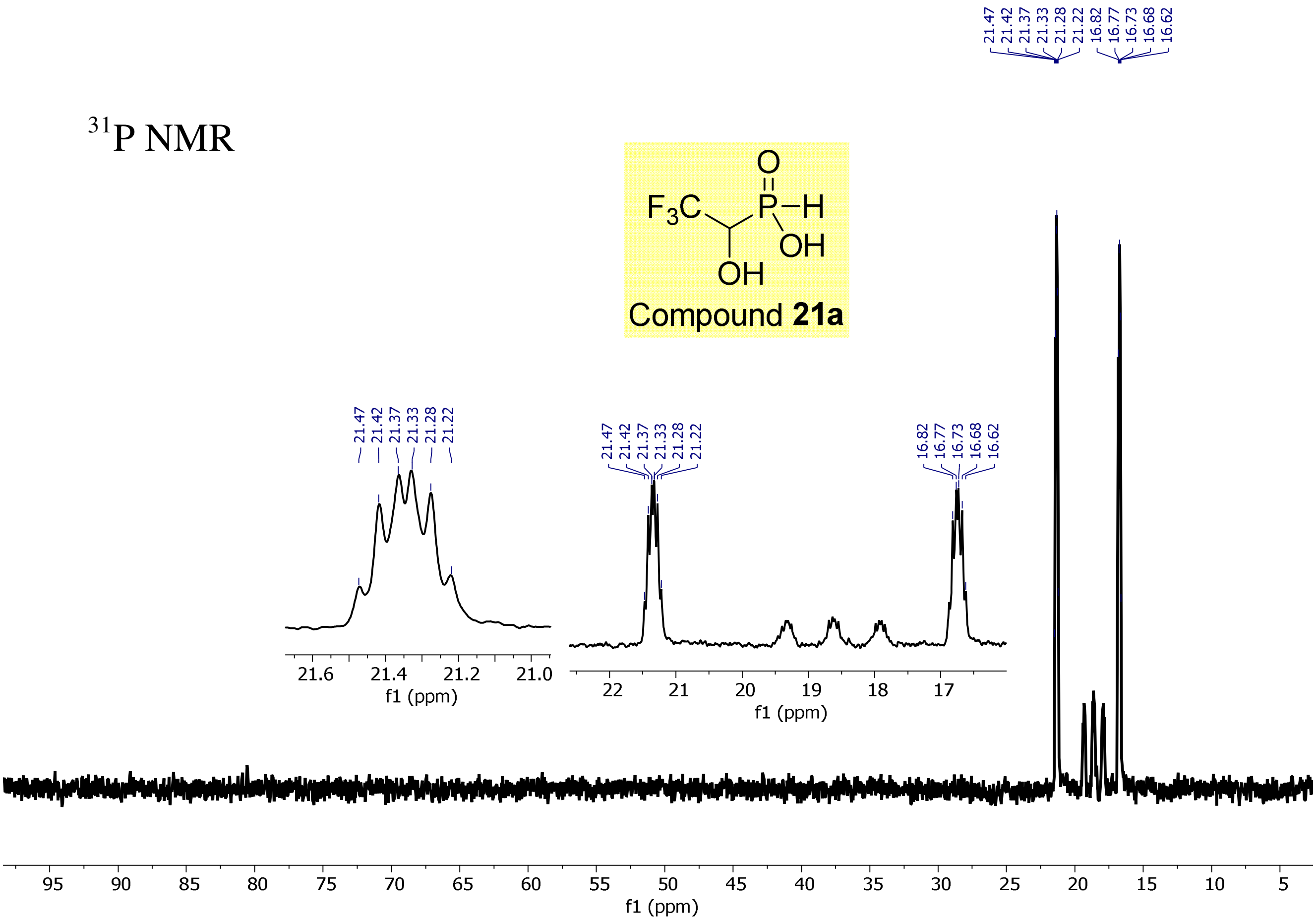

31P NMR
Compound 21a
21.47 21.42 21.37 21.33 21.28 21.22
16.82 16.77 16.73 16.68 16.62
f1 (ppm)

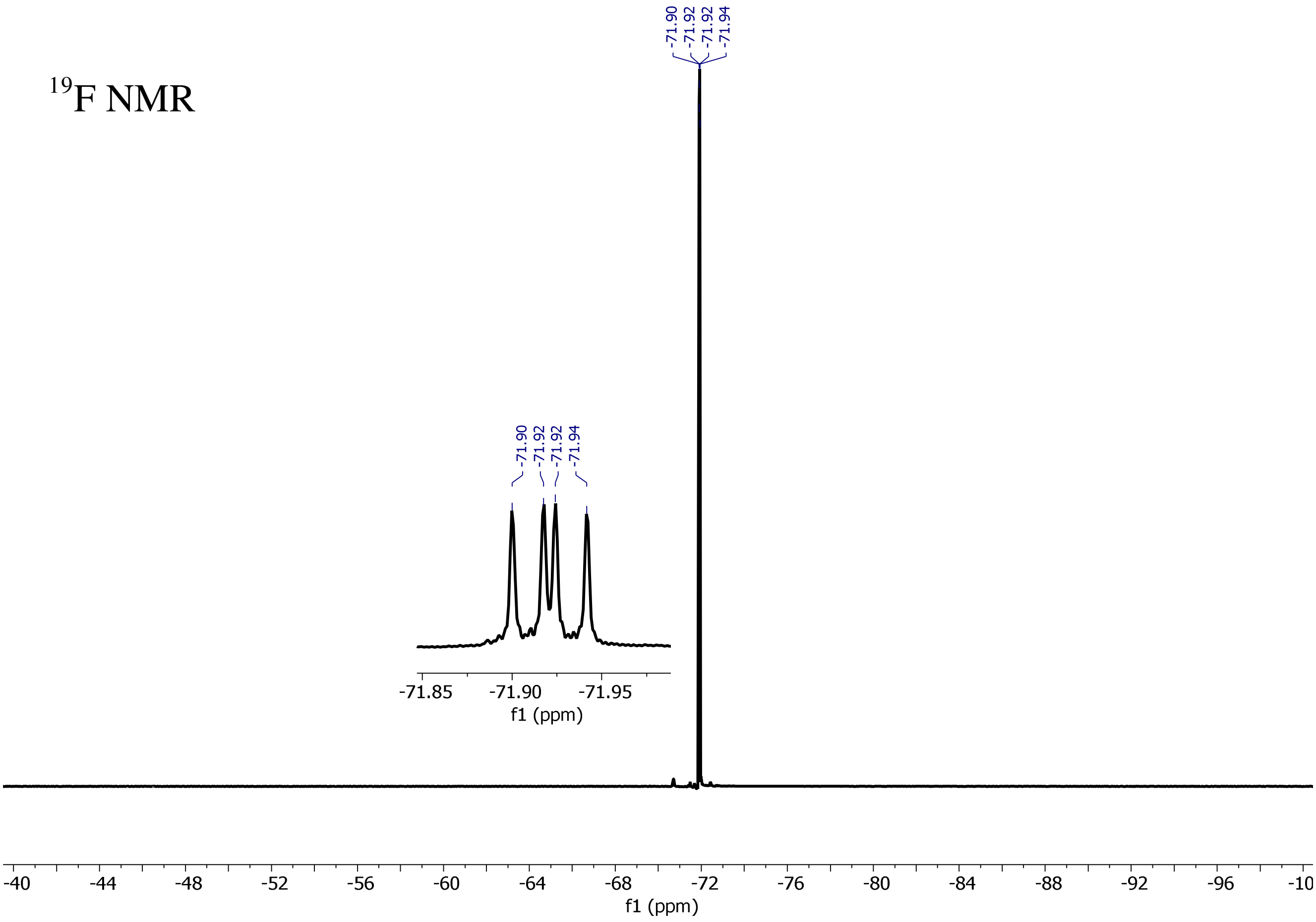

19F NMR
-71.90 -71.92 -71.92 -71.94
f1 (ppm)

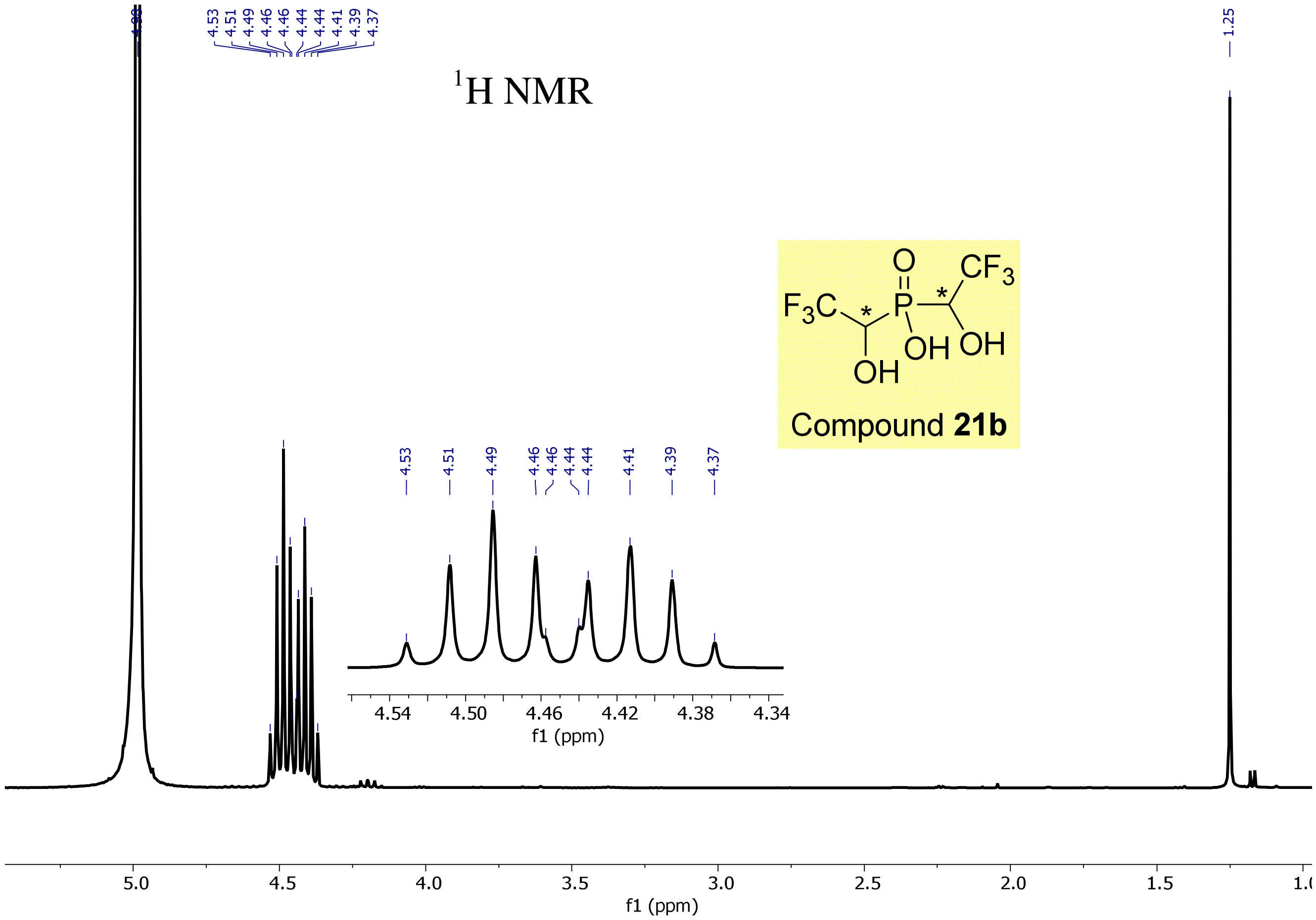

4.98
4.53 4.51 4.49 4.46 4.46 4.44 4.44 4.44 4.41 4.39 4.37
1.25
¹H NMR
Compound 21b
f1 (ppm)

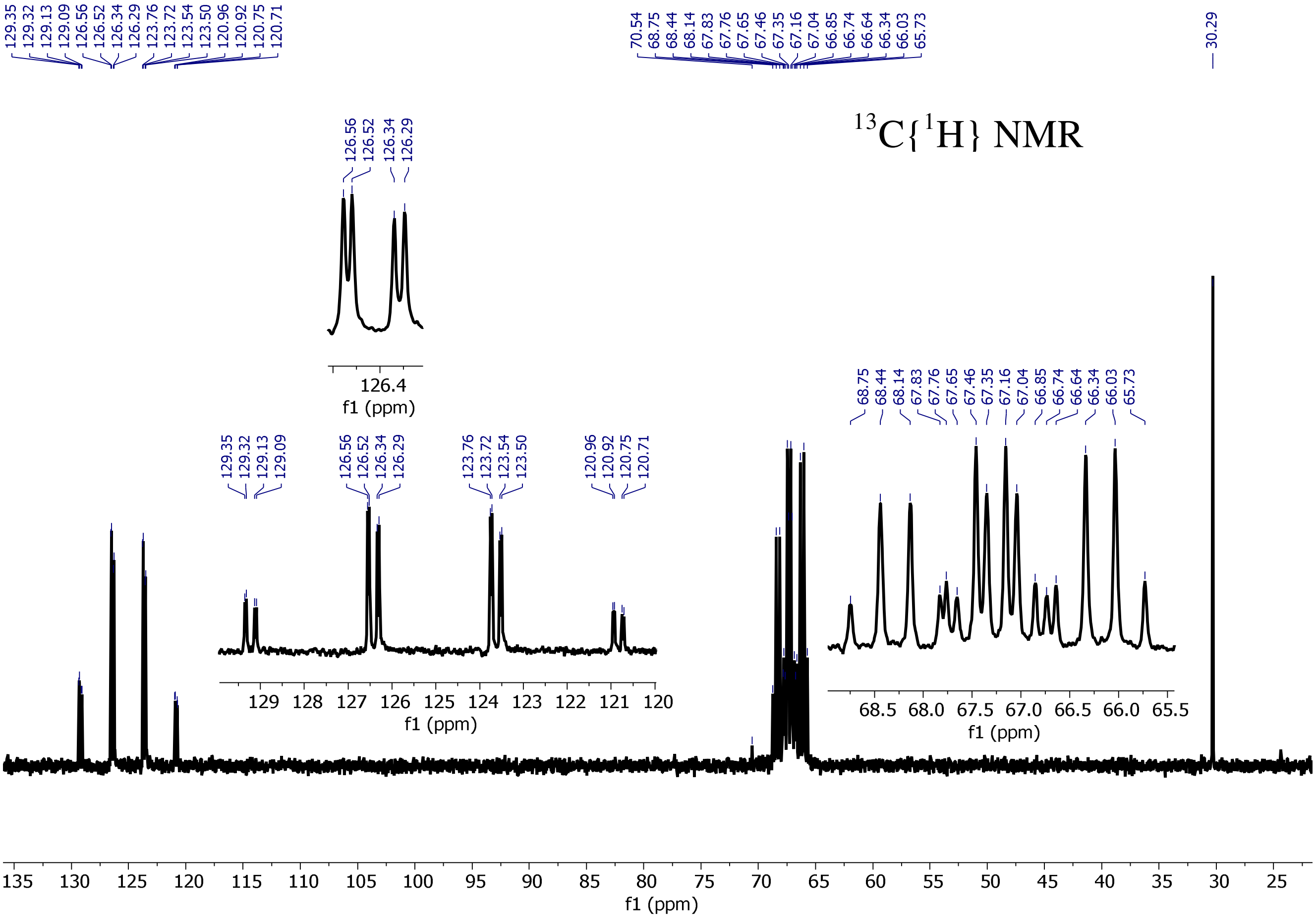

129.35 129.32 129.13 129.09 126.56 126.52 126.34 126.29 123.76 123.72 123.54 123.50 120.96 120.92 120.75 120.71
70.54 68.75 68.44 68.14 67.83 67.76 67.65 67.46 67.35 67.16 67.04 66.85 66.74 66.64 66.34 66.03 65.73
30.29
¹³C{¹H} NMR
f1 (ppm)

^{31}P NMR

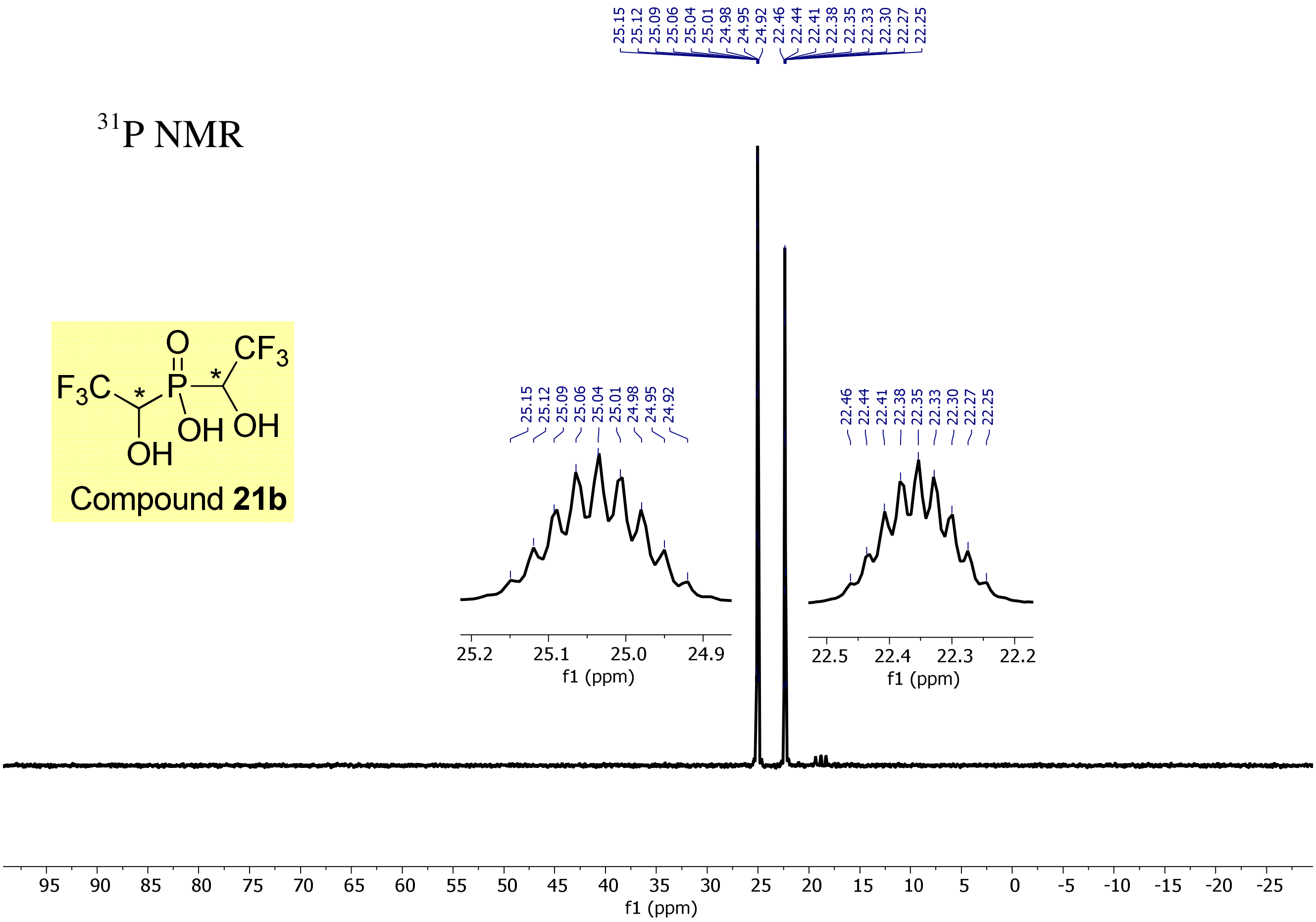

Compound **21b**

^{19}F NMR

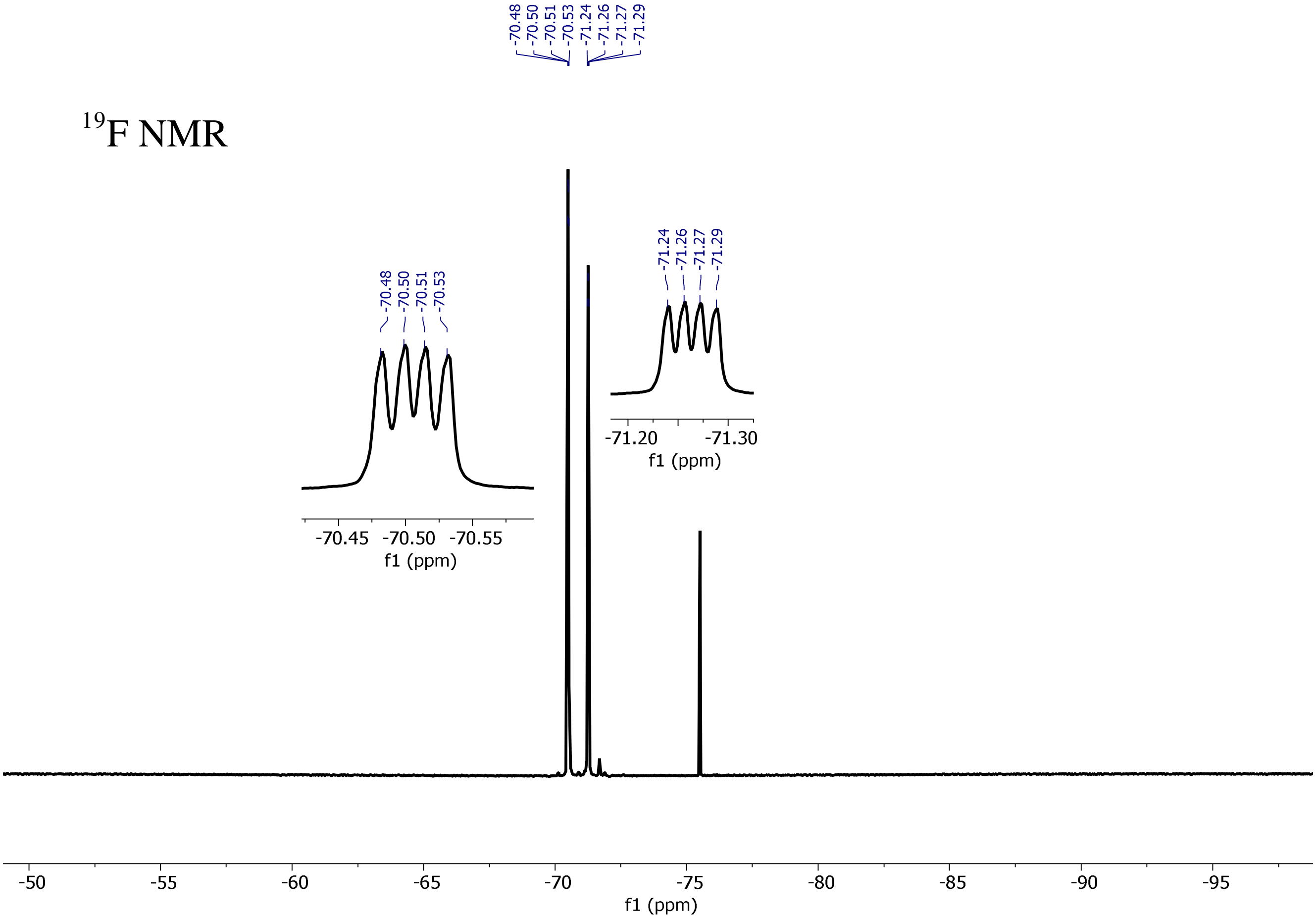

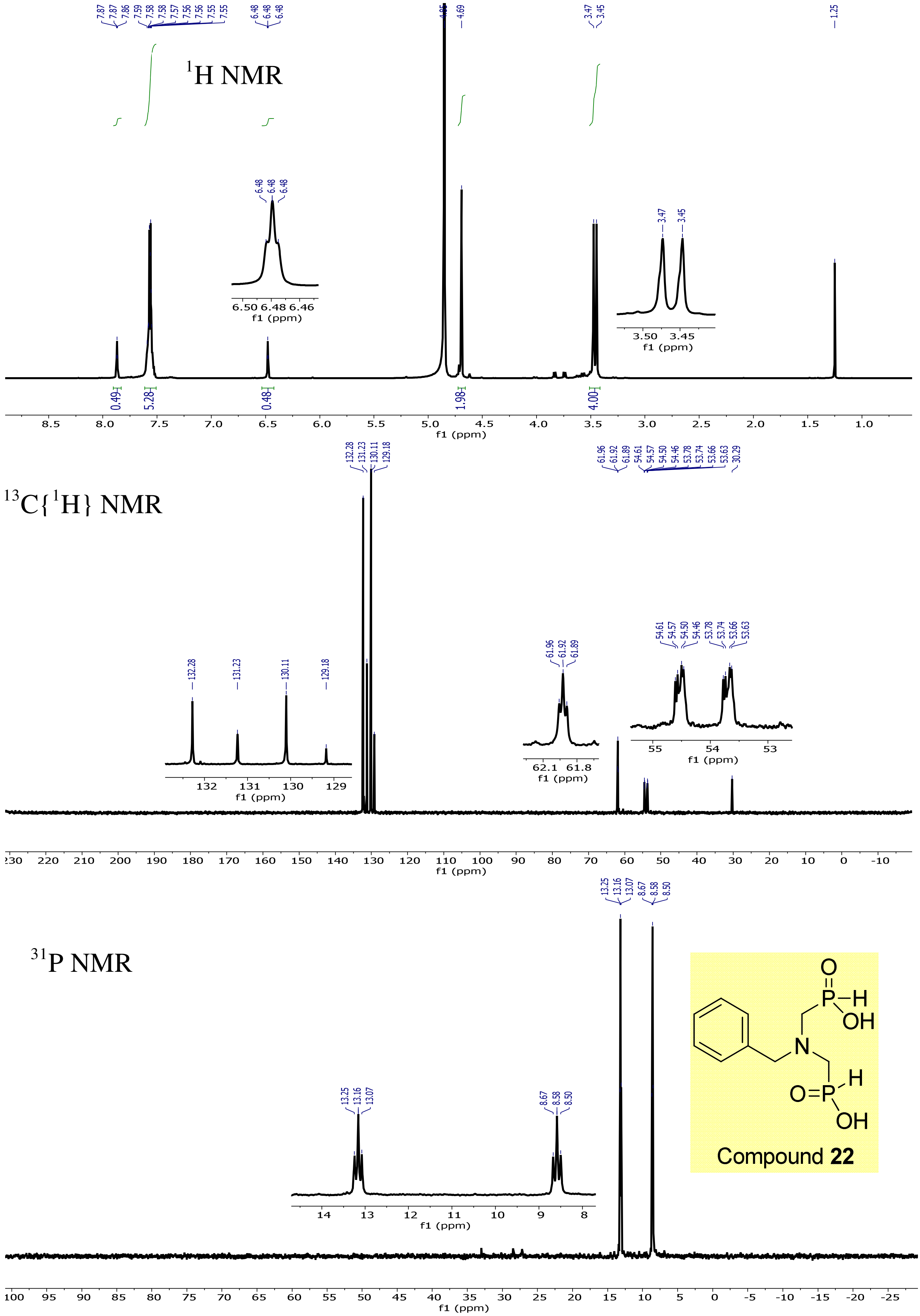

1H NMR
13C{1H} NMR
31P NMR
f1 (ppm)
Compound 22

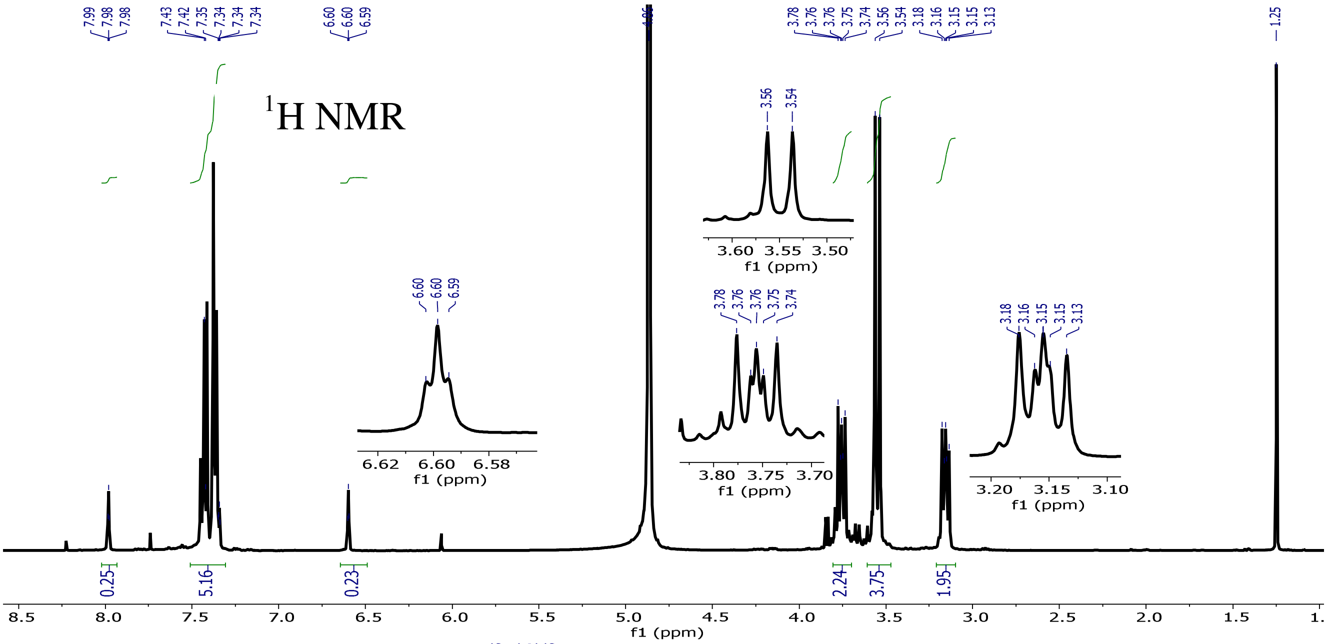

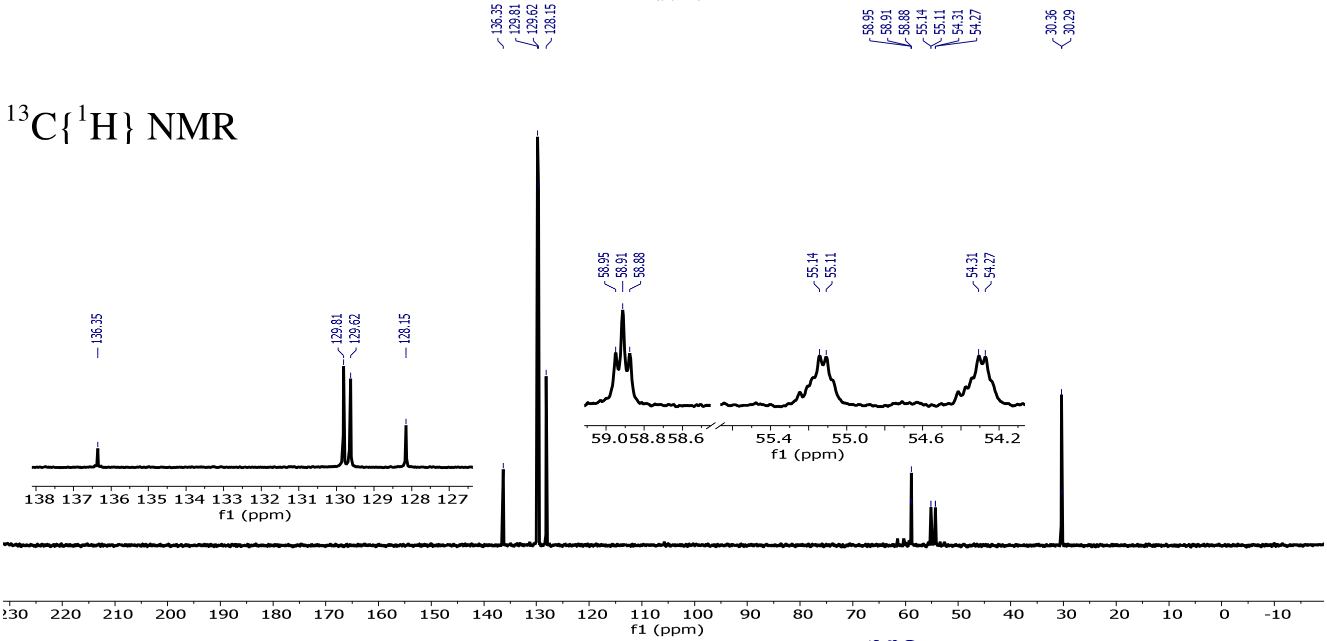

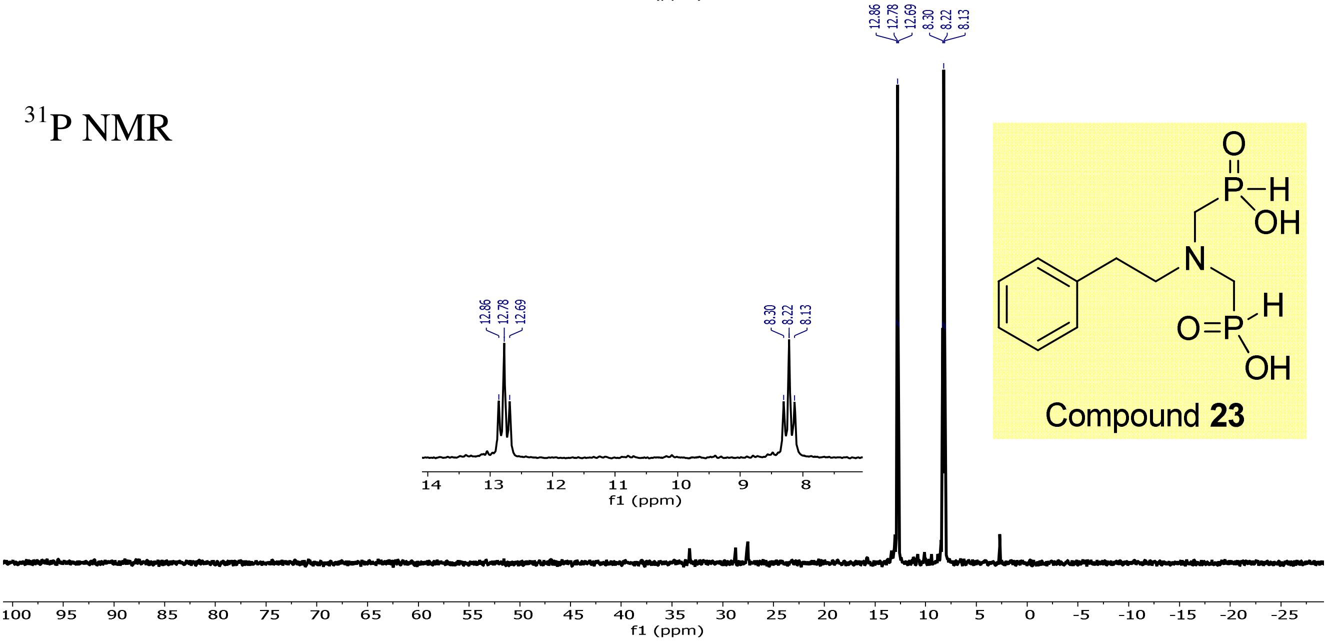

Compound **23**

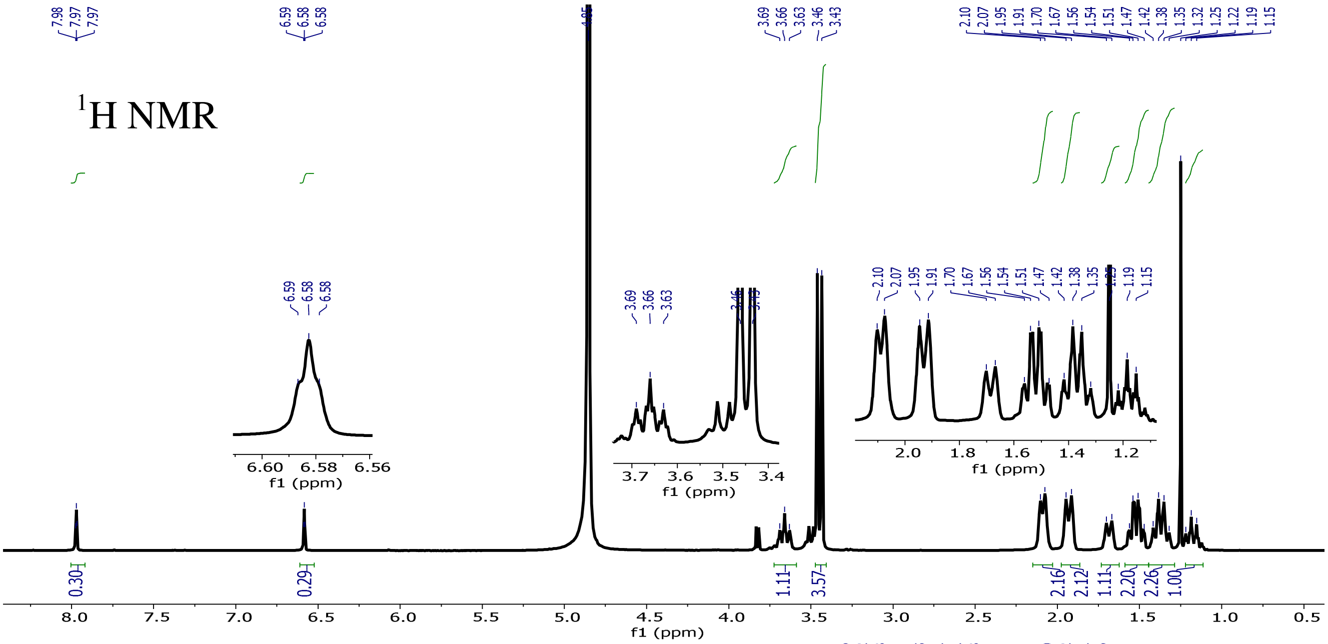
^{1}H NMR
f1 (ppm)

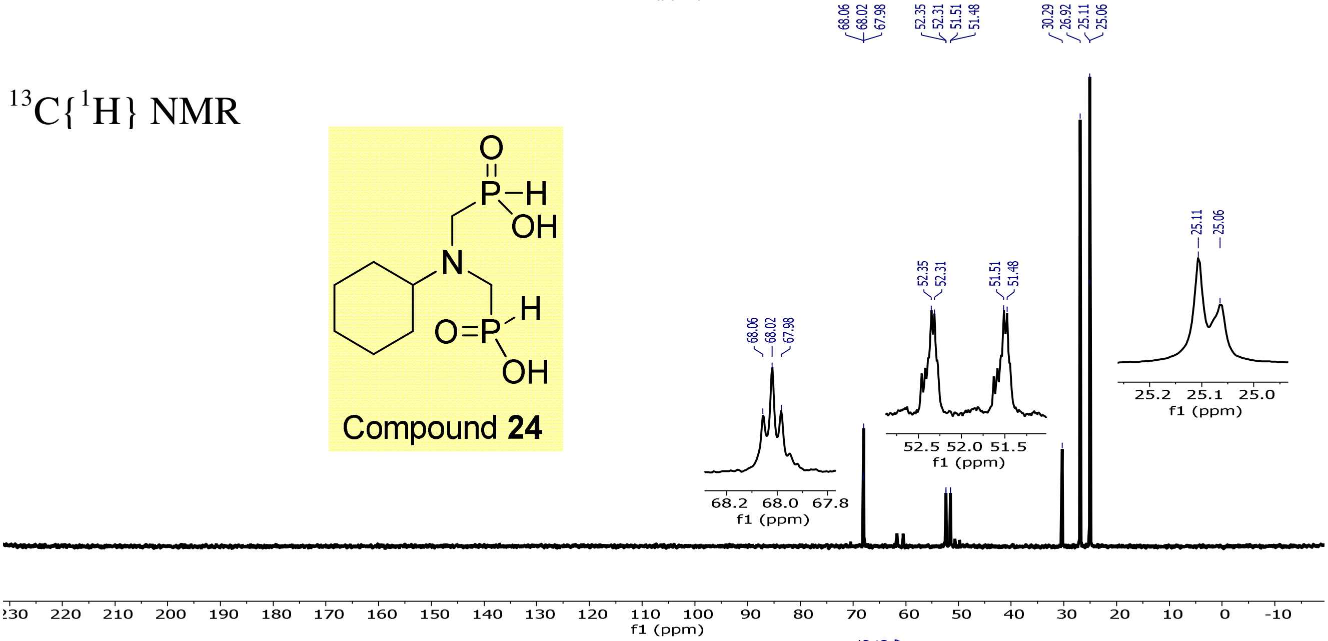
^{13}C{^{1}H} NMR
Compound 24
f1 (ppm)

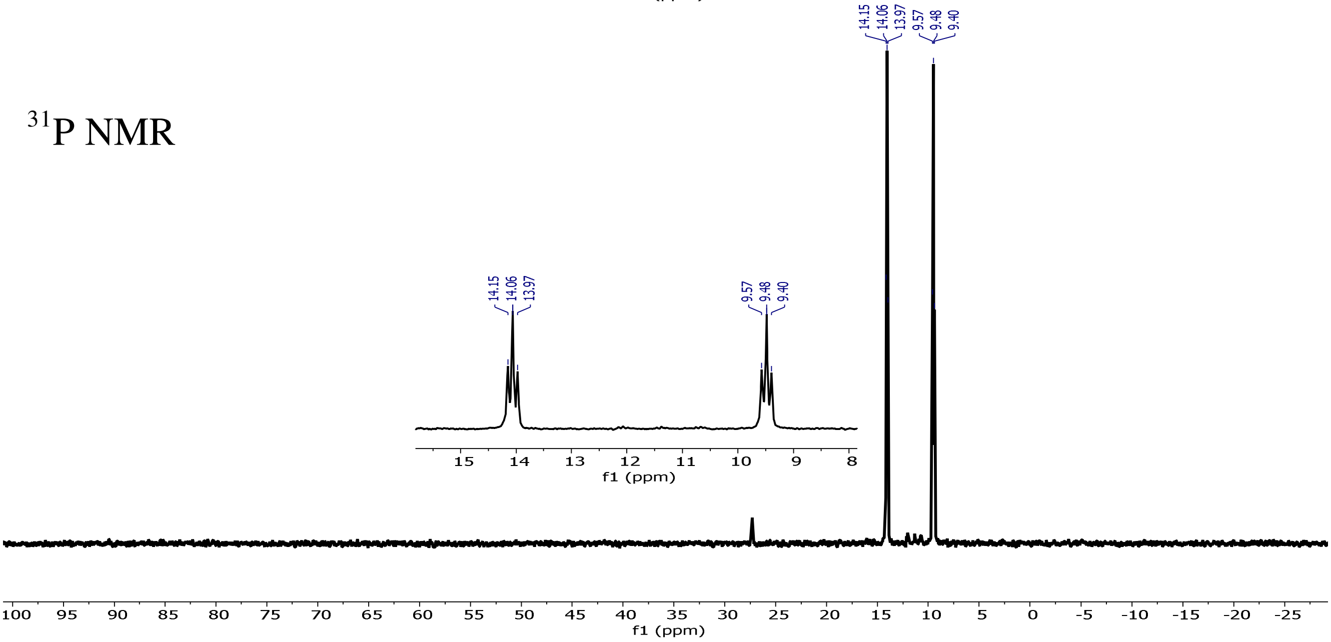
^{31}P NMR
f1 (ppm)

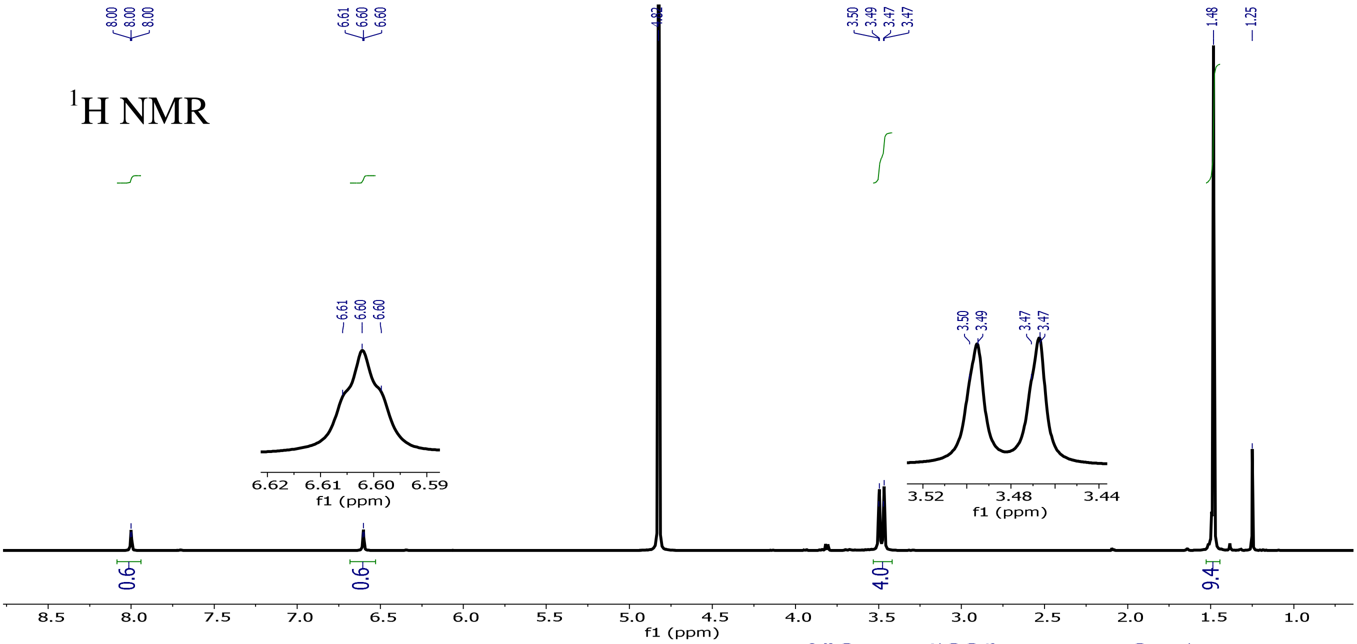

^{1}H NMR
8.00 8.00 8.00
6.61 6.60 6.60
4.82
3.50 3.49 3.47 3.47
1.48
1.25
0.6
0.6
4.0
9.4
6.62 6.61 6.60 6.59
3.52 3.48 3.44
f1 (ppm)

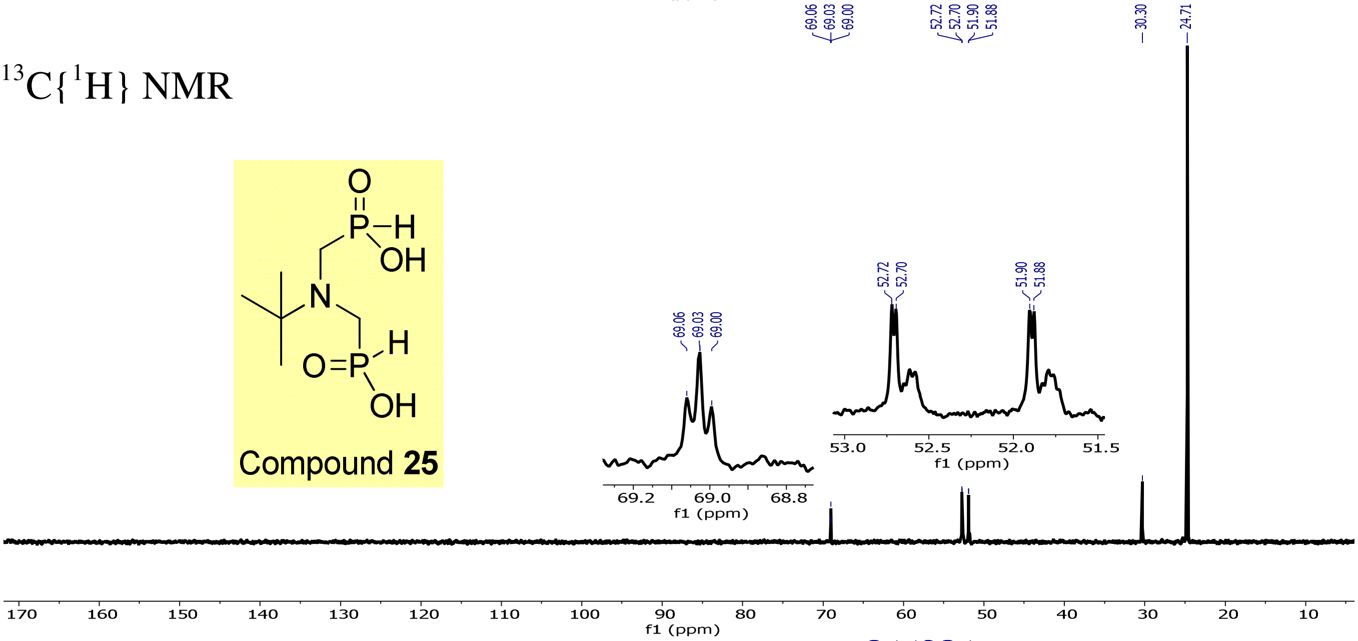

^{13}C{^{1}H} NMR
Compound **25**
69.06 69.03 69.00
52.72 52.70 51.90 51.88
30.30
24.71
69.2 69.0 68.8
53.0 52.5 52.0 51.5
f1 (ppm)

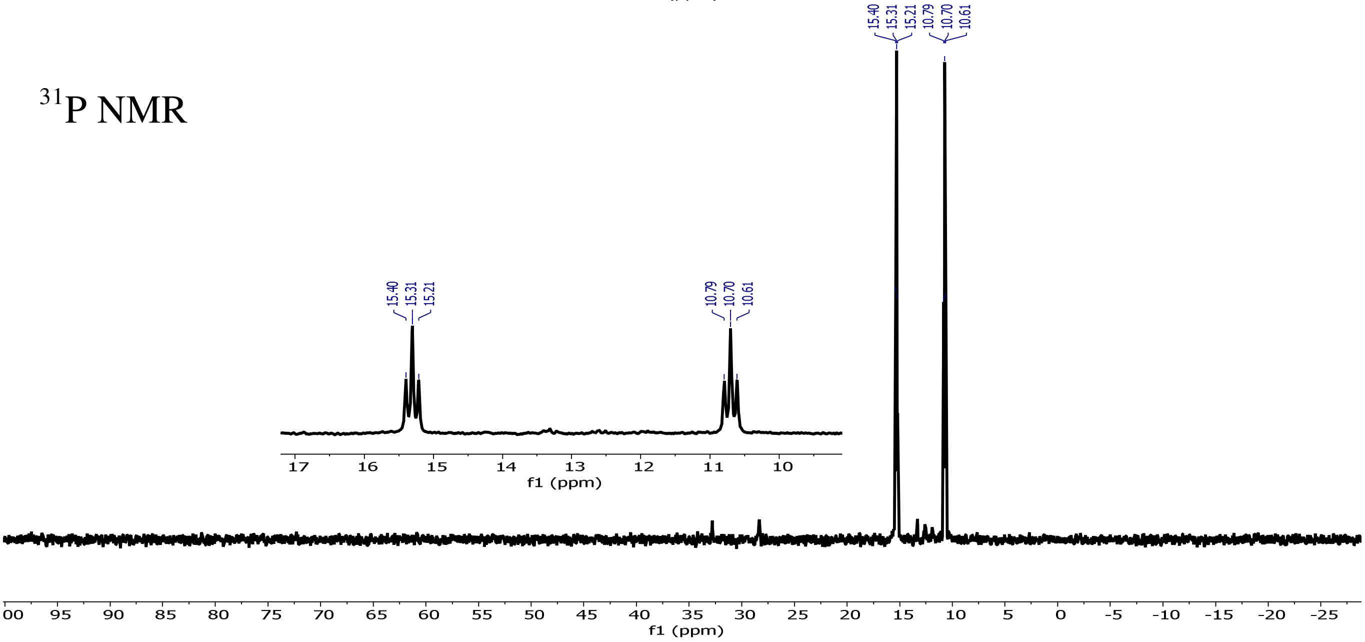

^{31}P NMR
15.40 15.31 15.21
10.79 10.70 10.61
17 16 15 14 13 12 11 10
f1 (ppm)

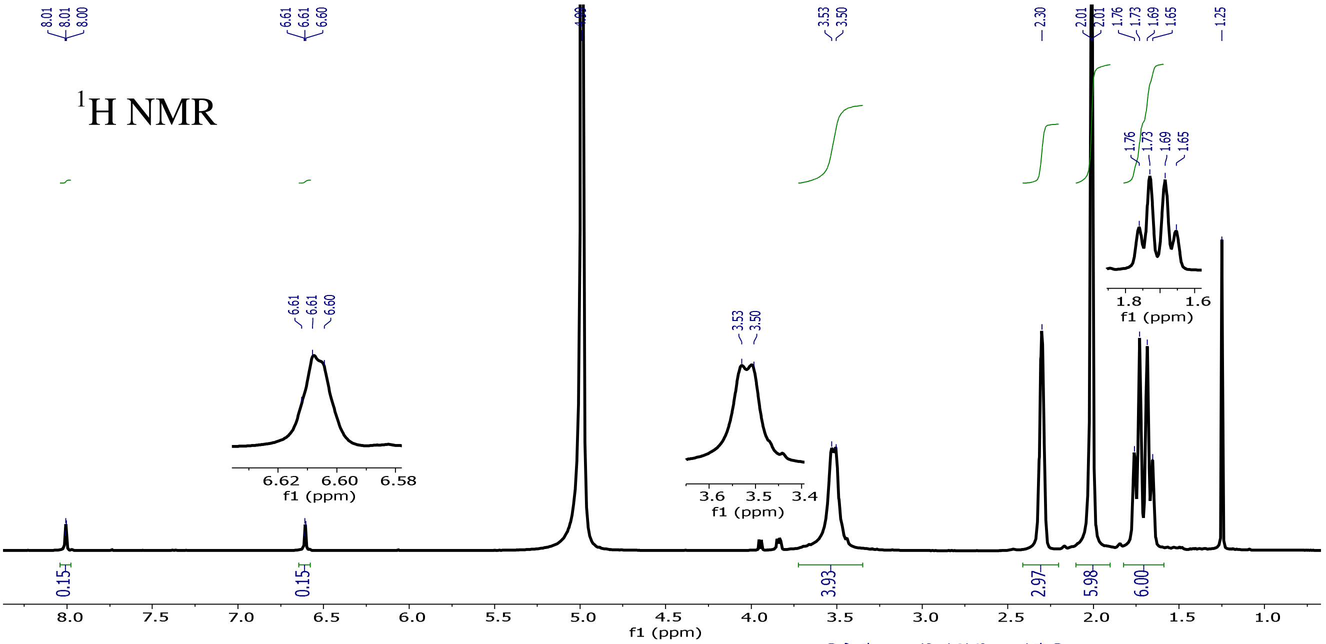

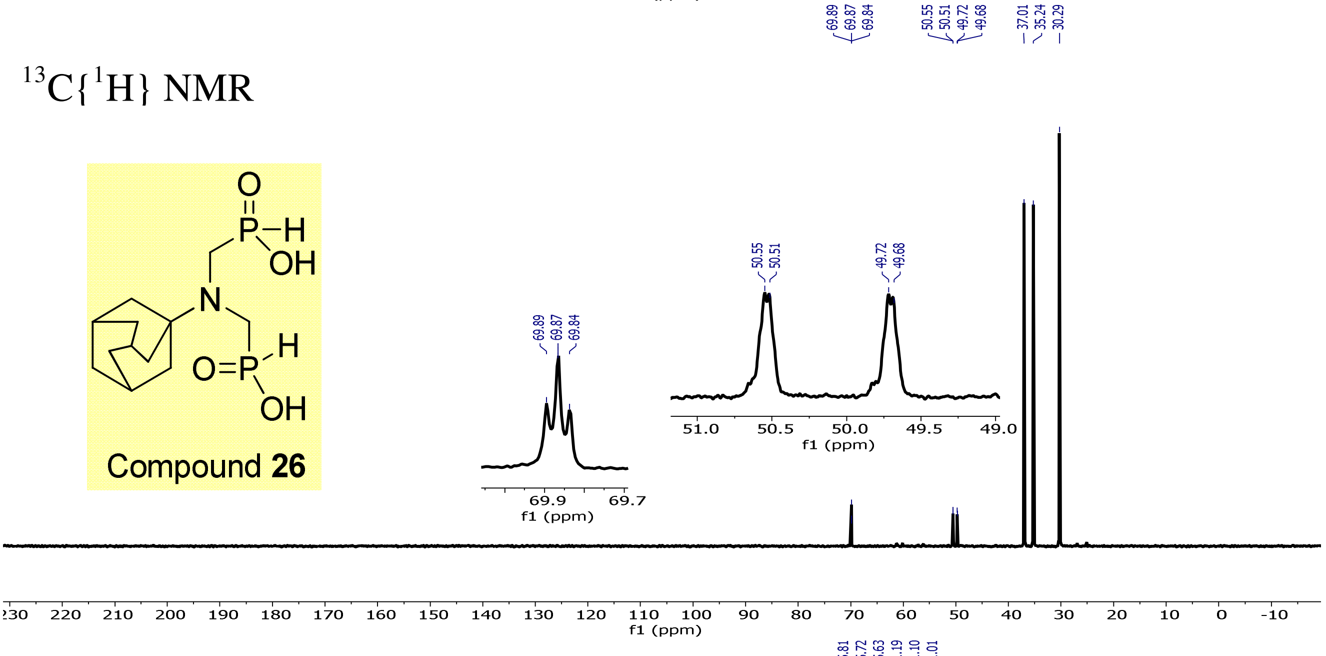

Compound **26**

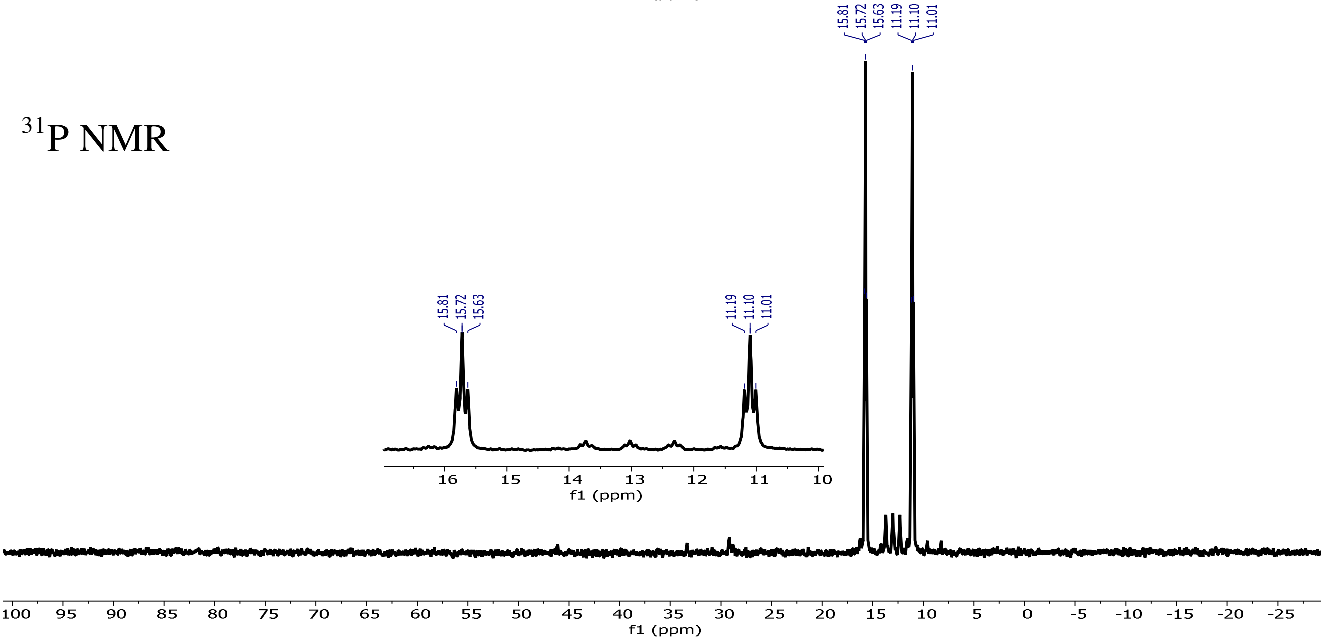

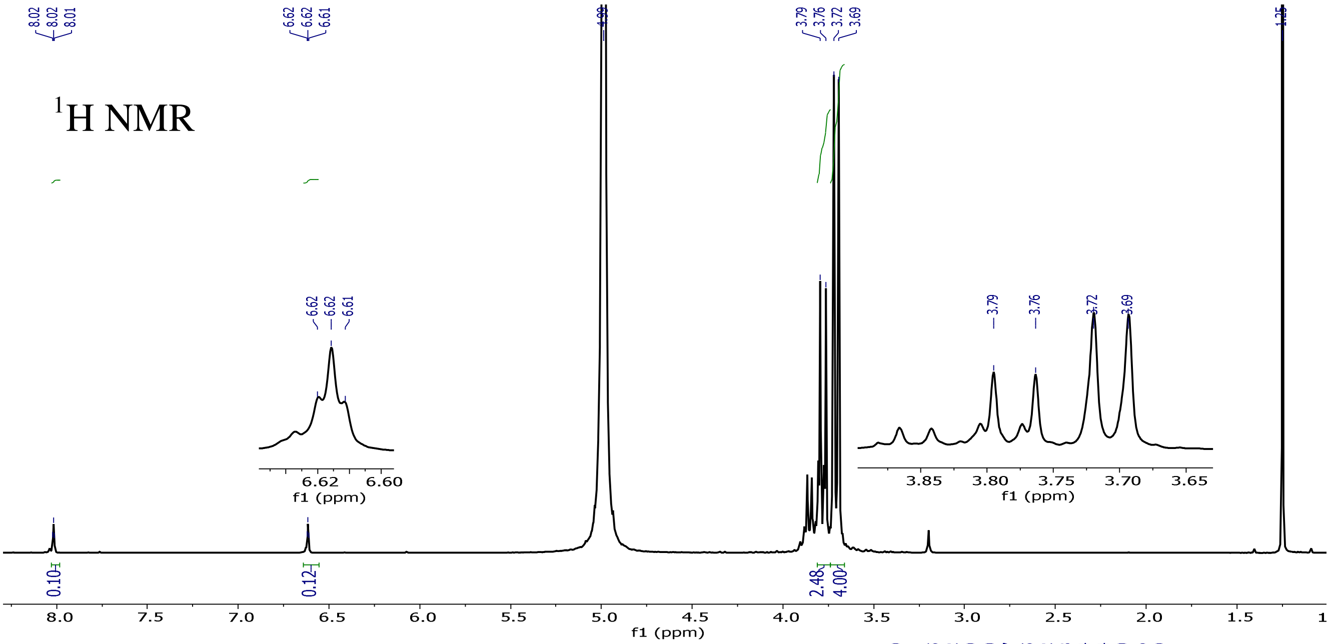
1H NMR
f1 (ppm)

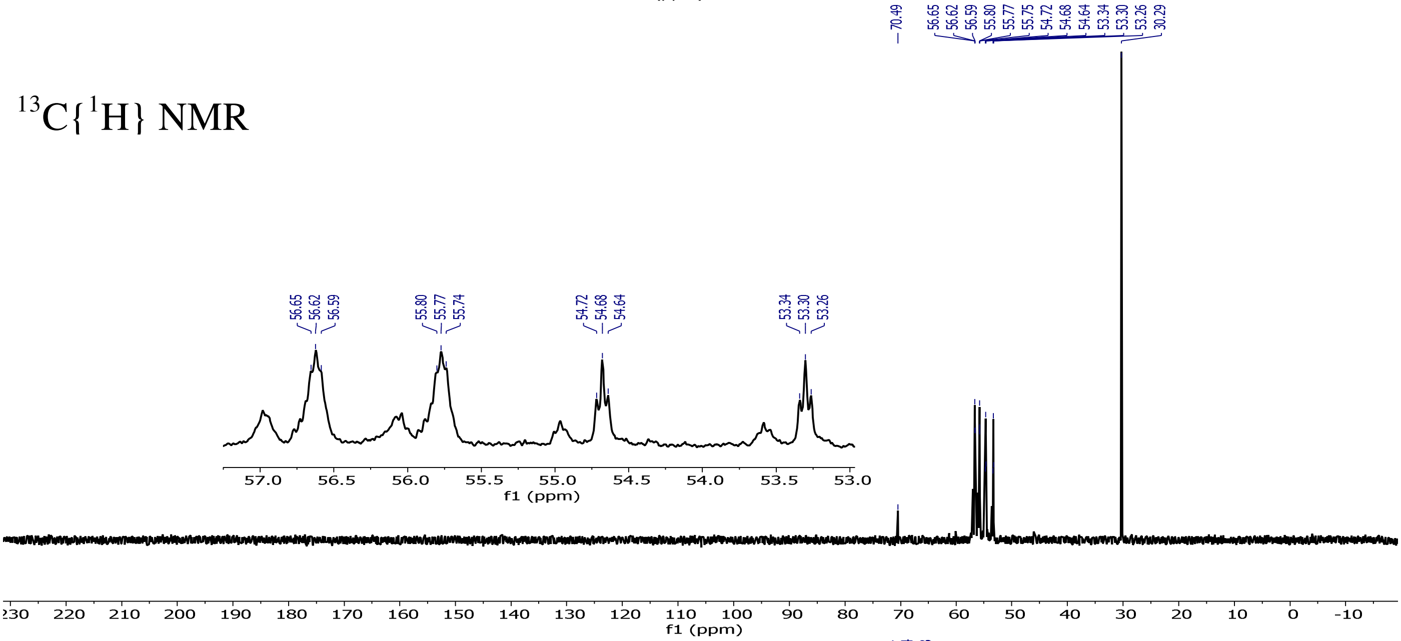
13C{1H} NMR
f1 (ppm)

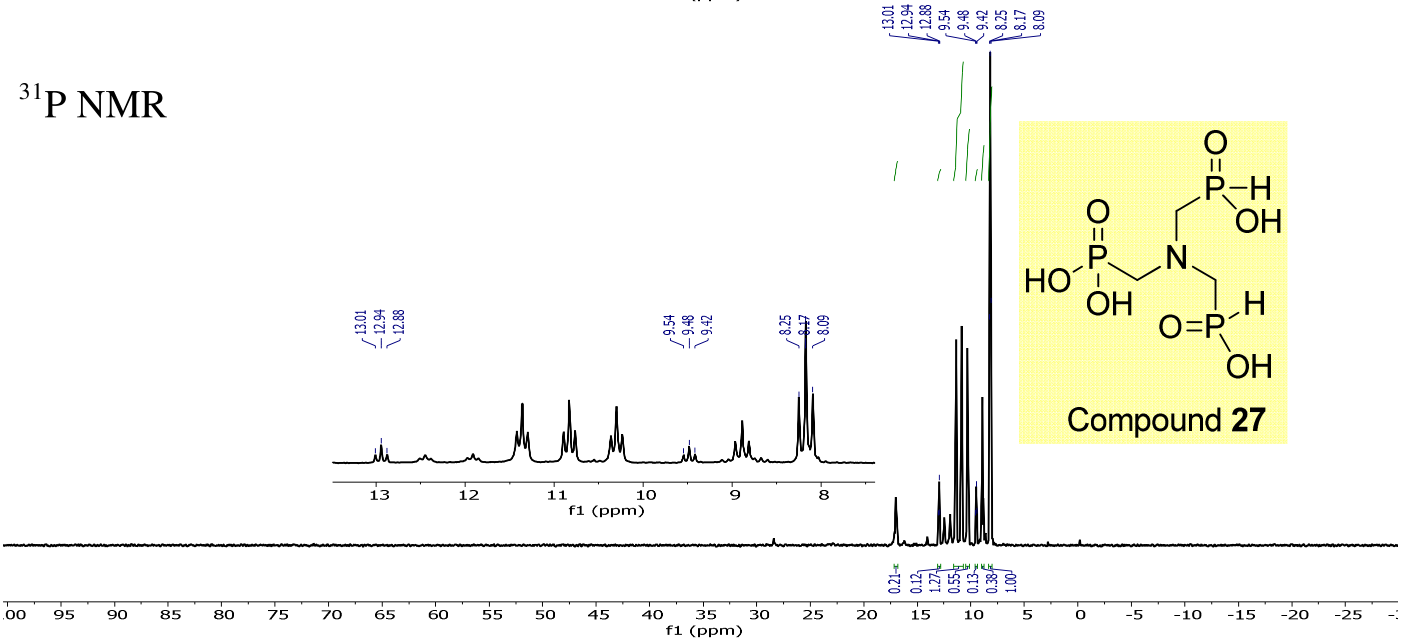
31P NMR
f1 (ppm)
Compound 27

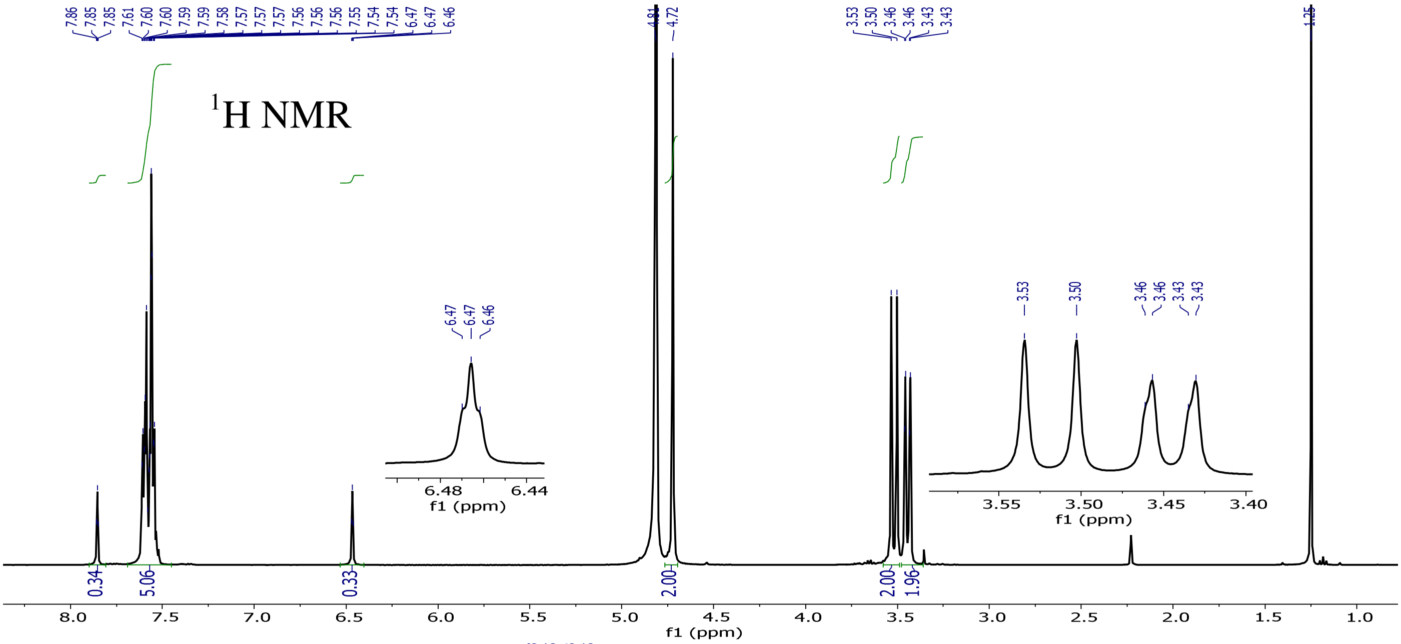

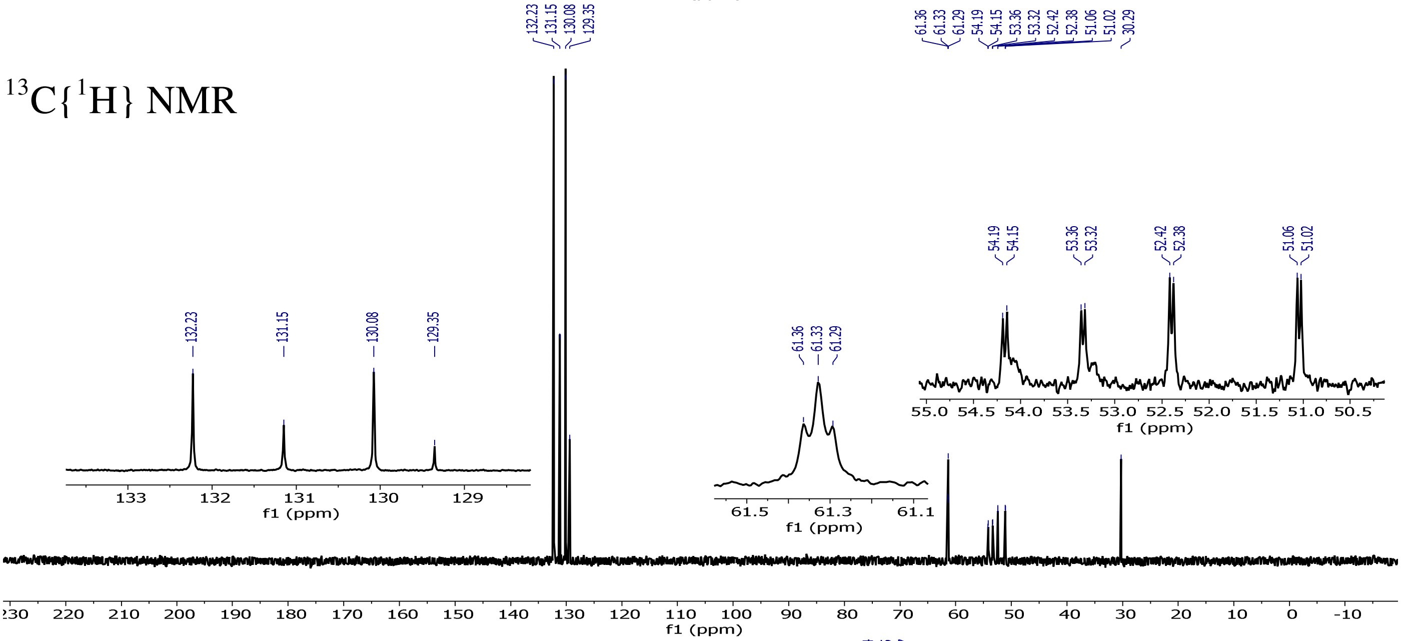

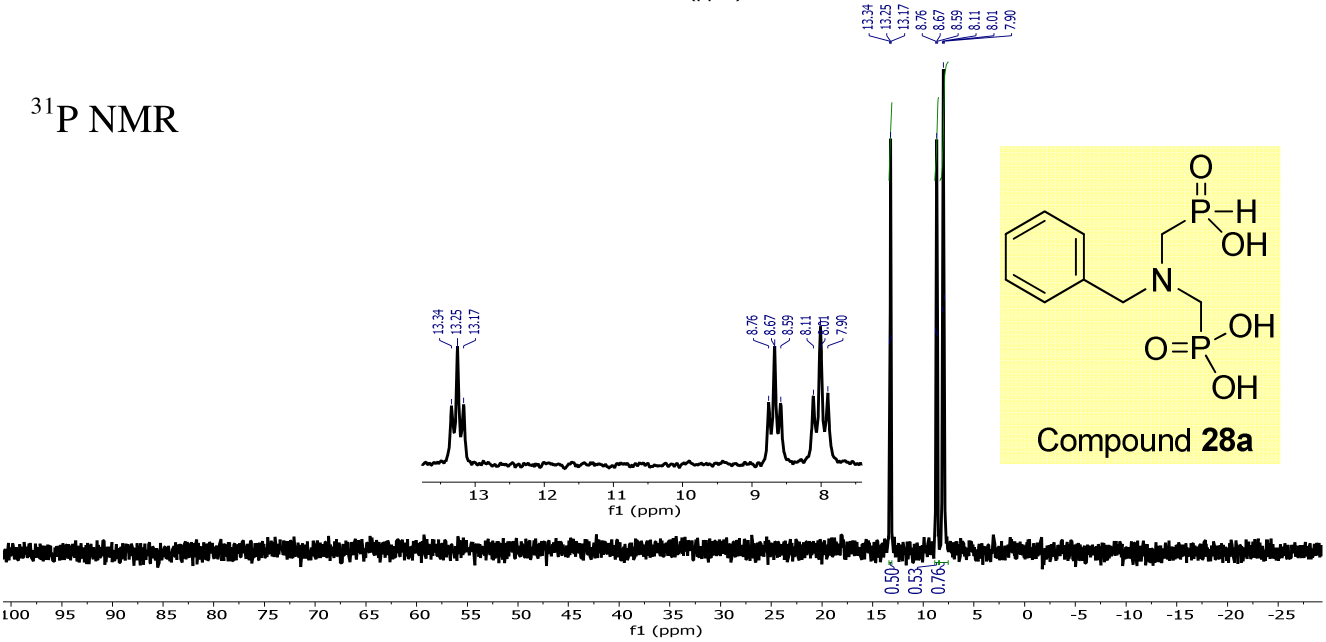

Compound **28a**

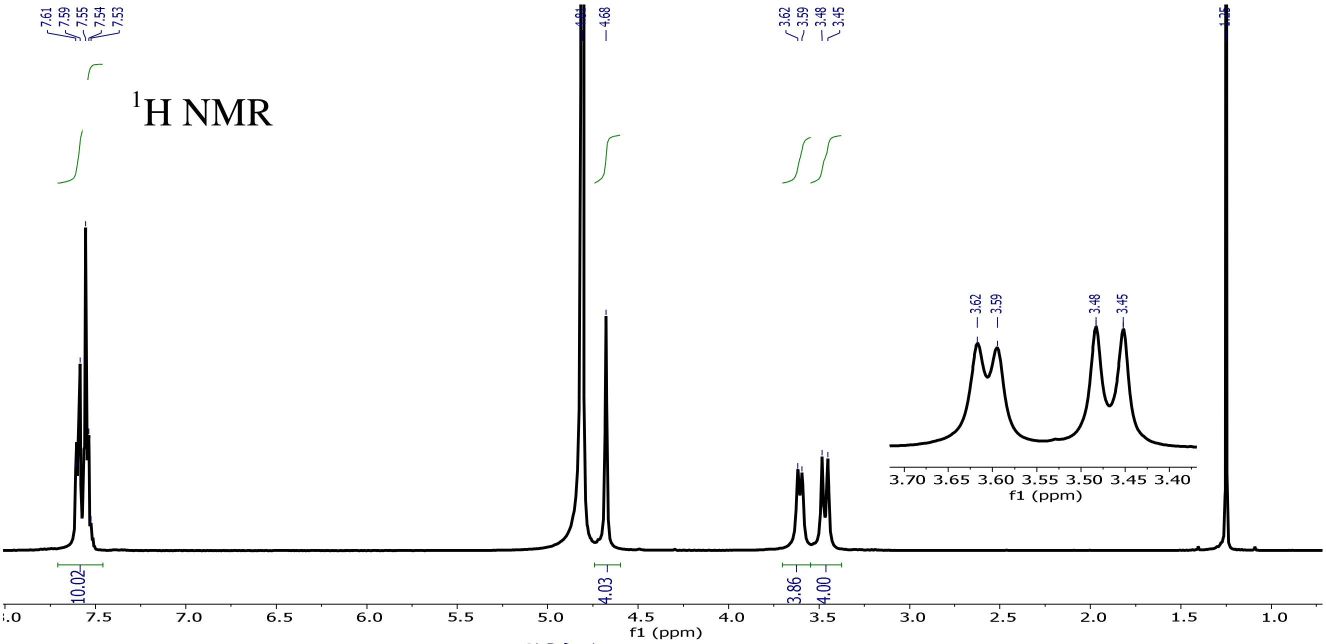

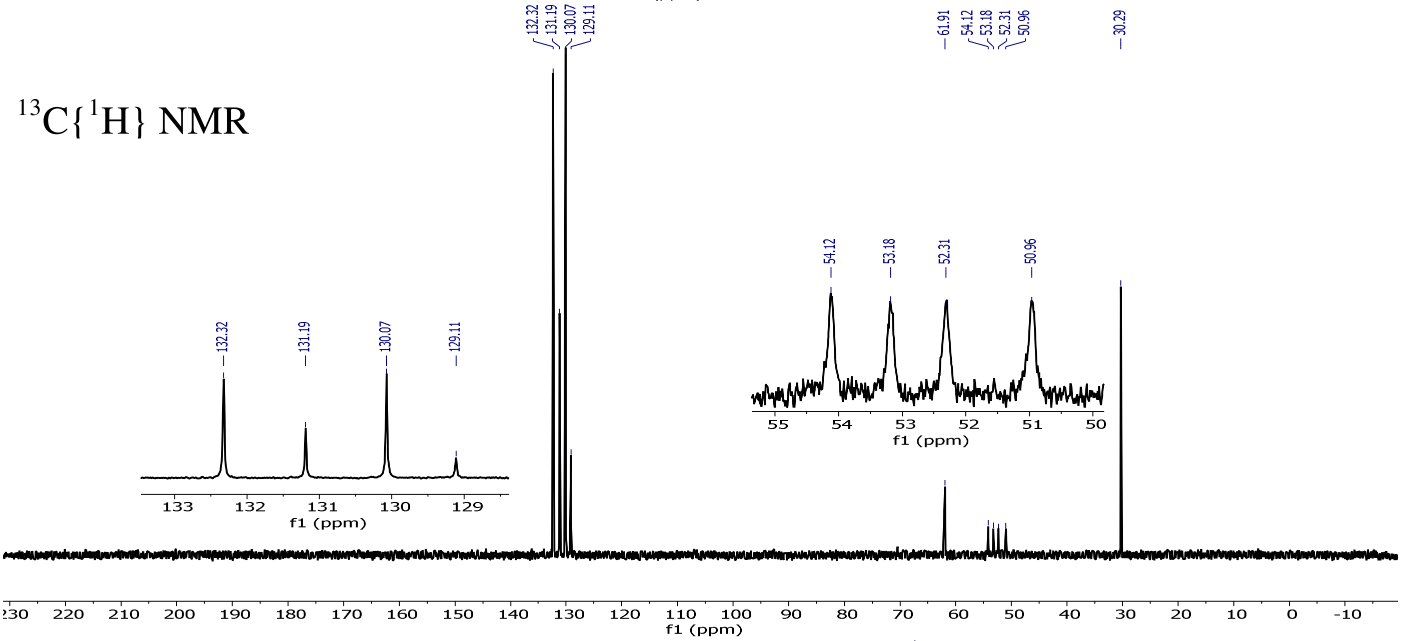

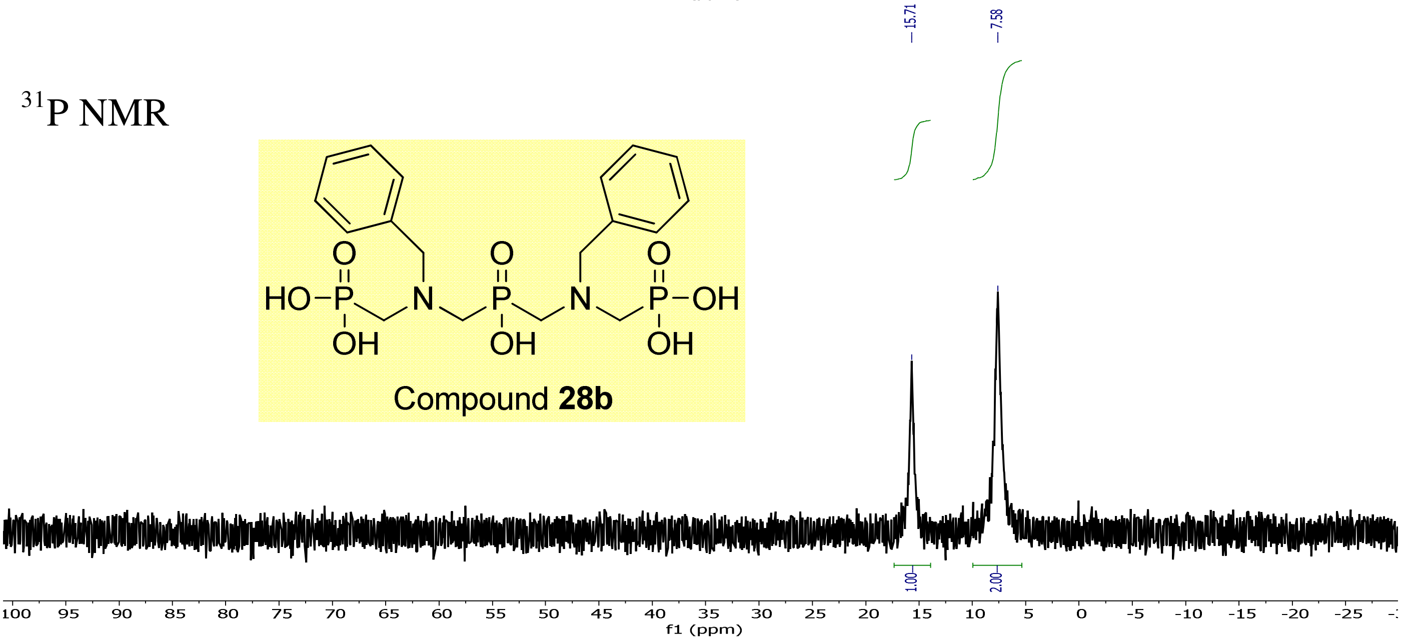

Compound **28b**

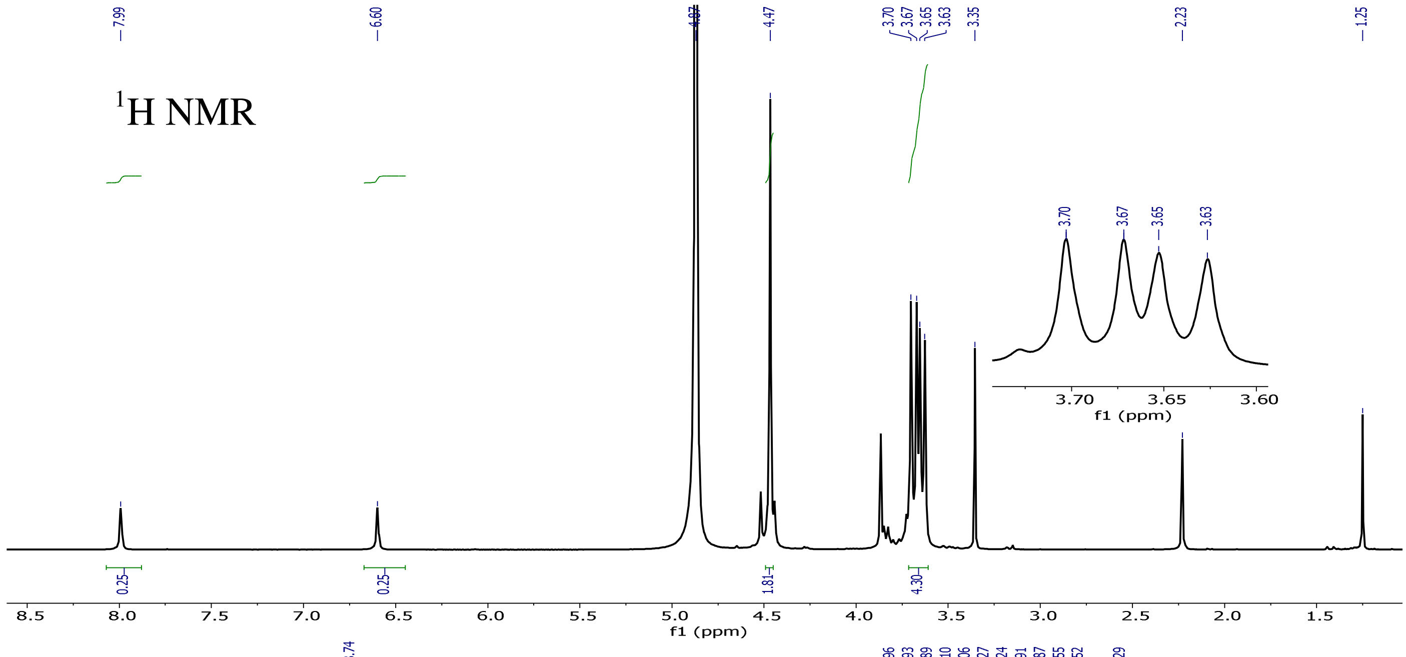

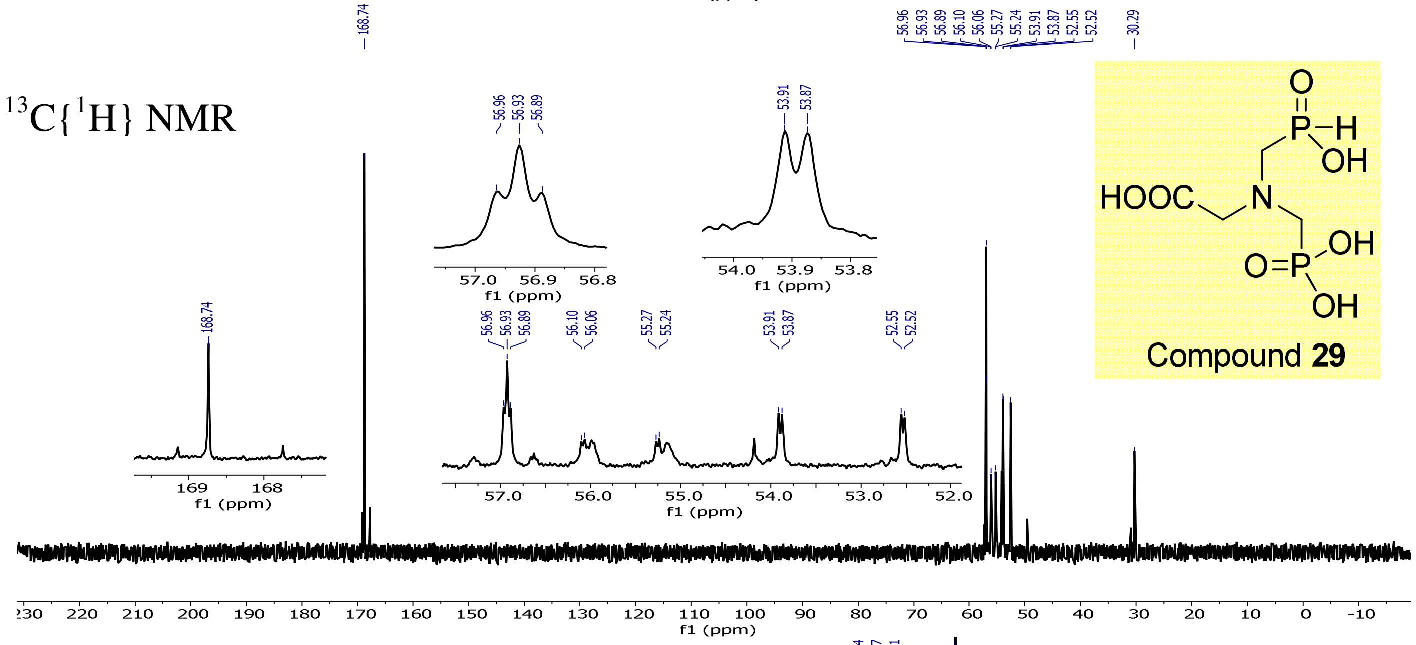

Compound **29**

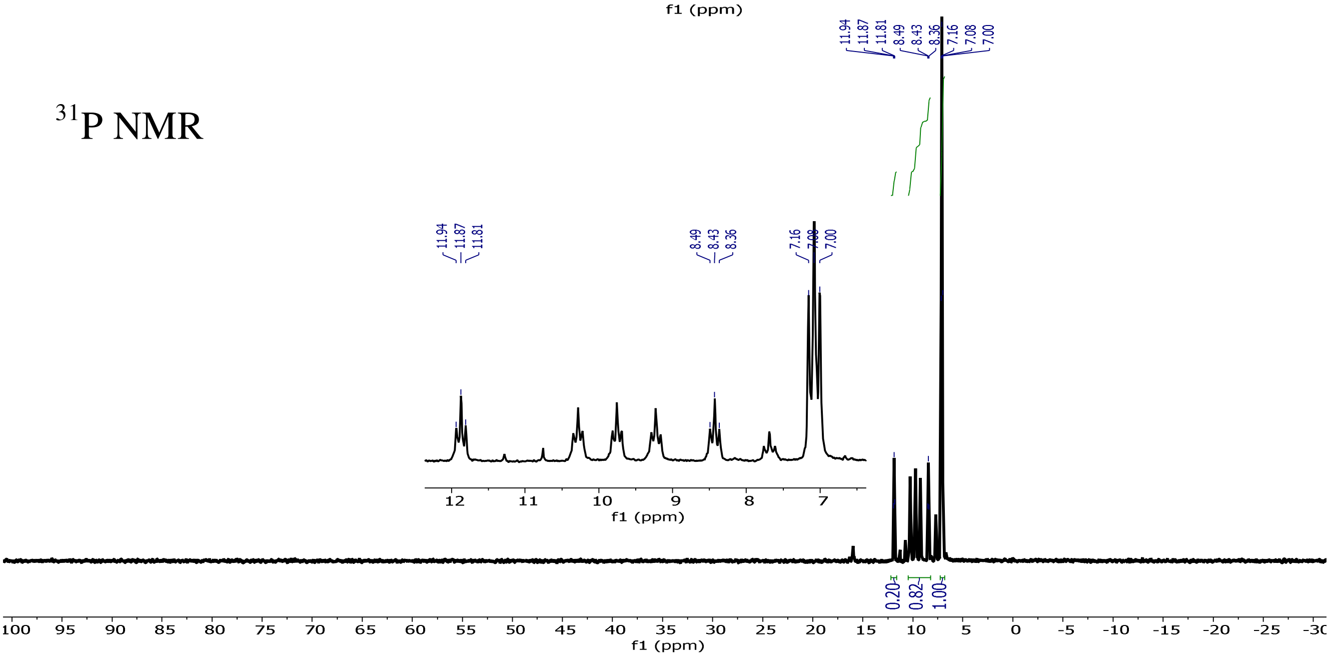

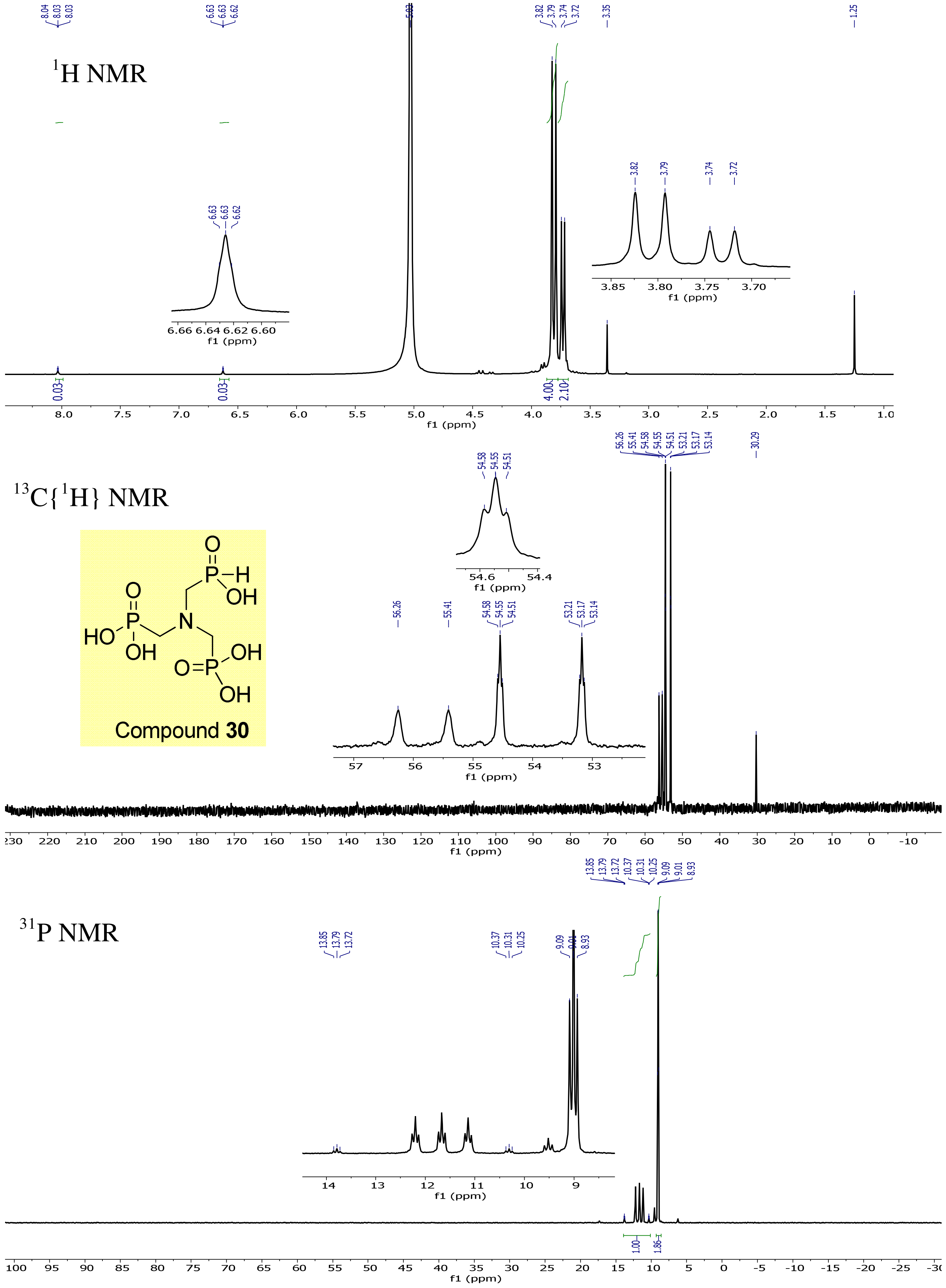

1H NMR
13C{1H} NMR
Compound 30
31P NMR
f1 (ppm)

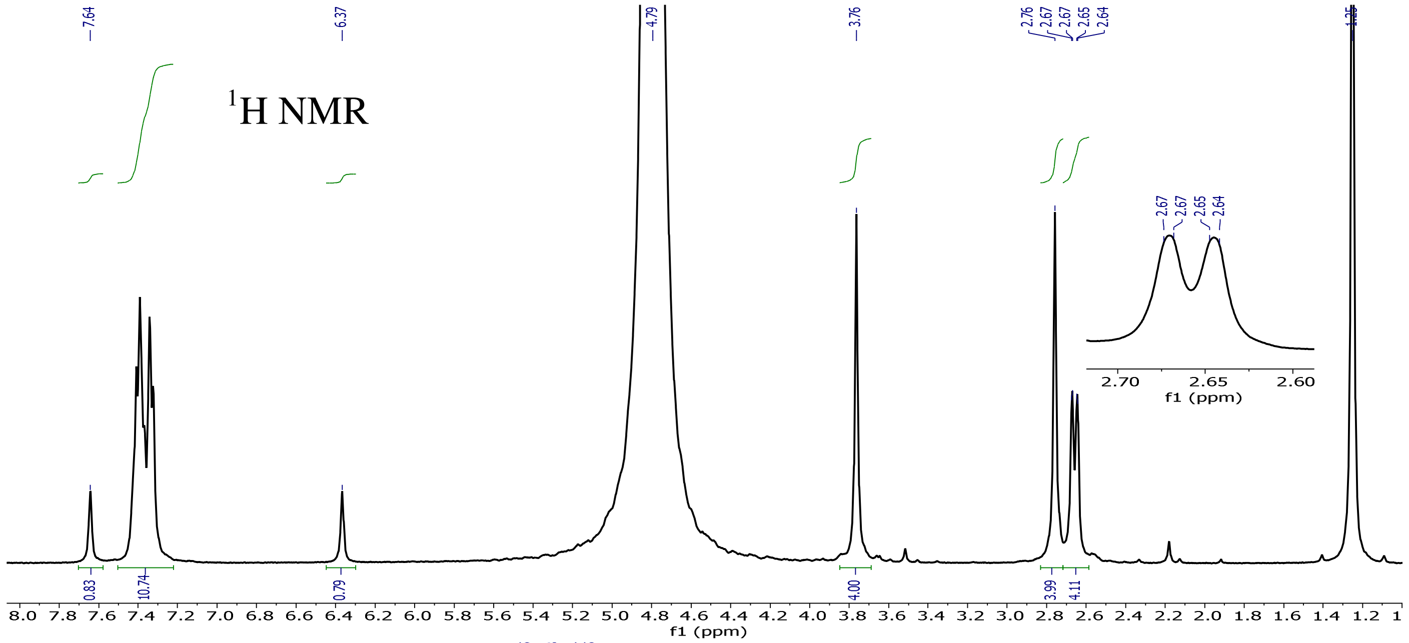
^{1}H NMR
7.64
6.37
4.79
3.76
2.76 2.67 2.67 2.65 2.64
1.25
0.83
10.74
0.79
4.00
3.99
4.11
f1 (ppm)
2.67 2.67 2.65 2.64
2.70 2.65 2.60
f1 (ppm)

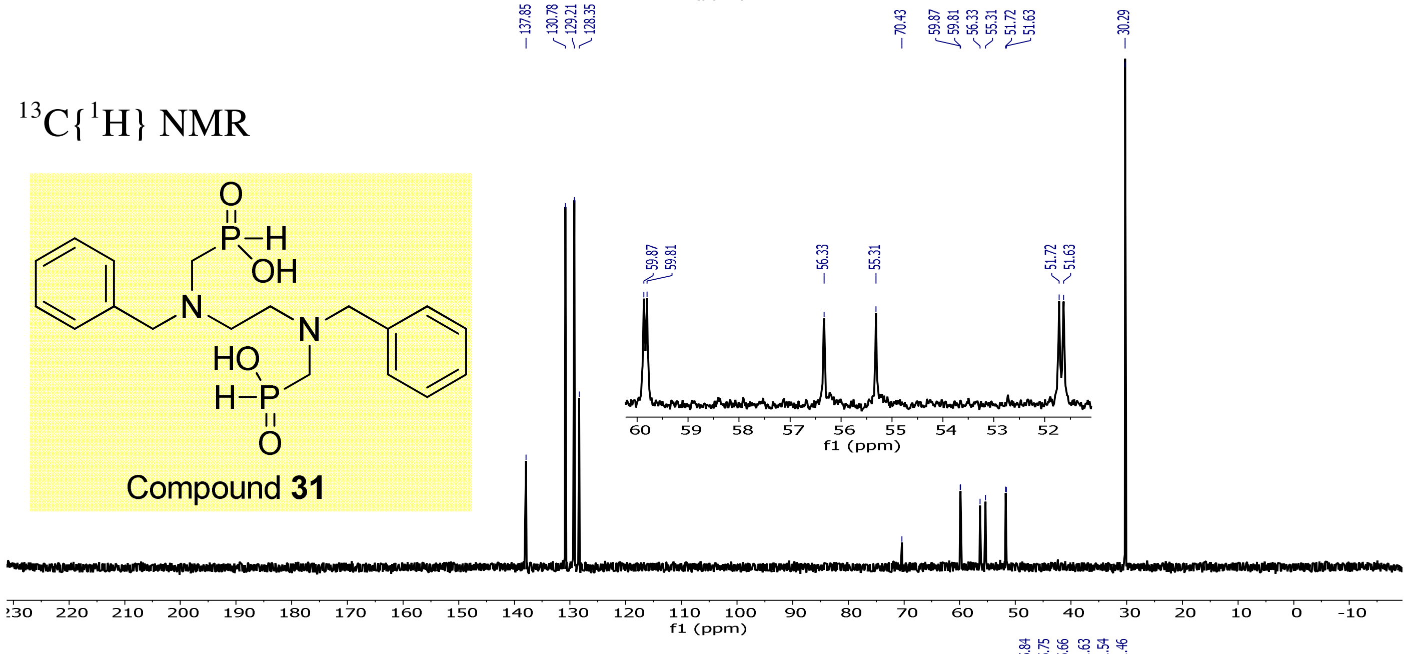
^{13}C{^{1}H} NMR
137.85 130.78 129.21 128.35
70.43
59.87 59.81 56.33 55.31 51.72 51.63
30.29
O
P–H
OH
N
N
HO
H–P
O
Compound 31
59.87 59.81
56.33
55.31
51.72 51.63
f1 (ppm)
f1 (ppm)

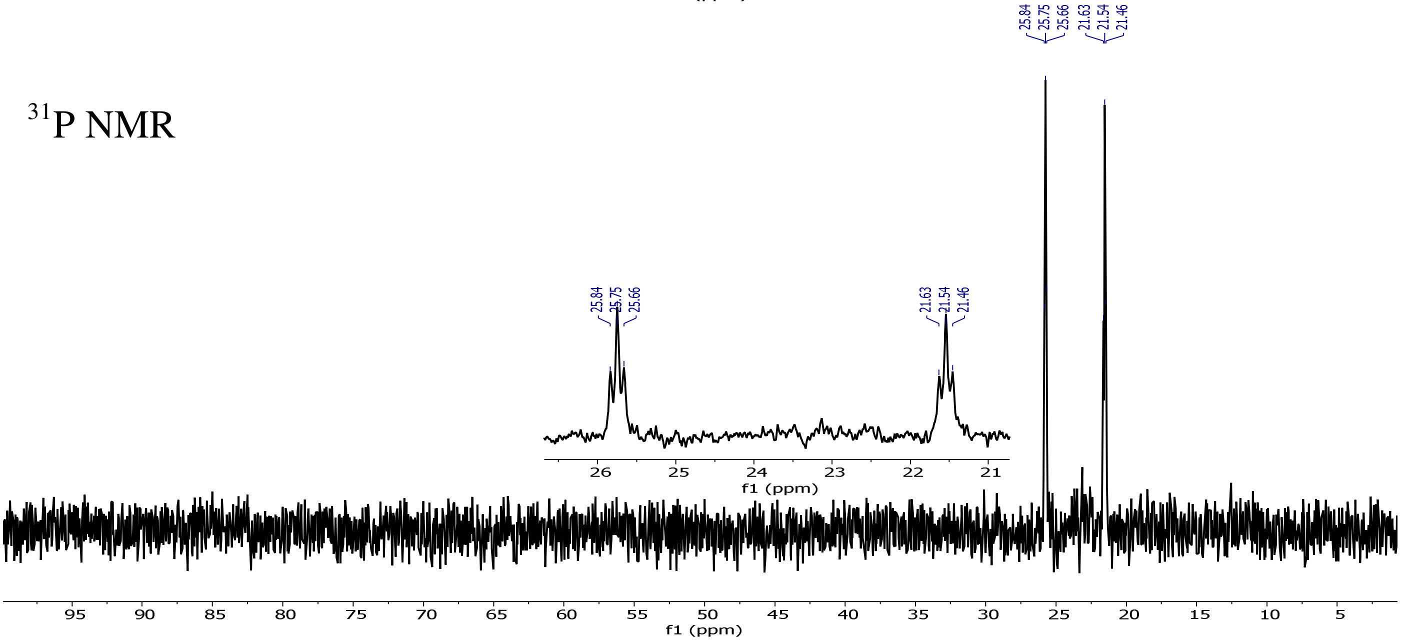
^{31}P NMR
25.84 25.75 25.66 21.63 21.54 21.46
f1 (ppm)
f1 (ppm)

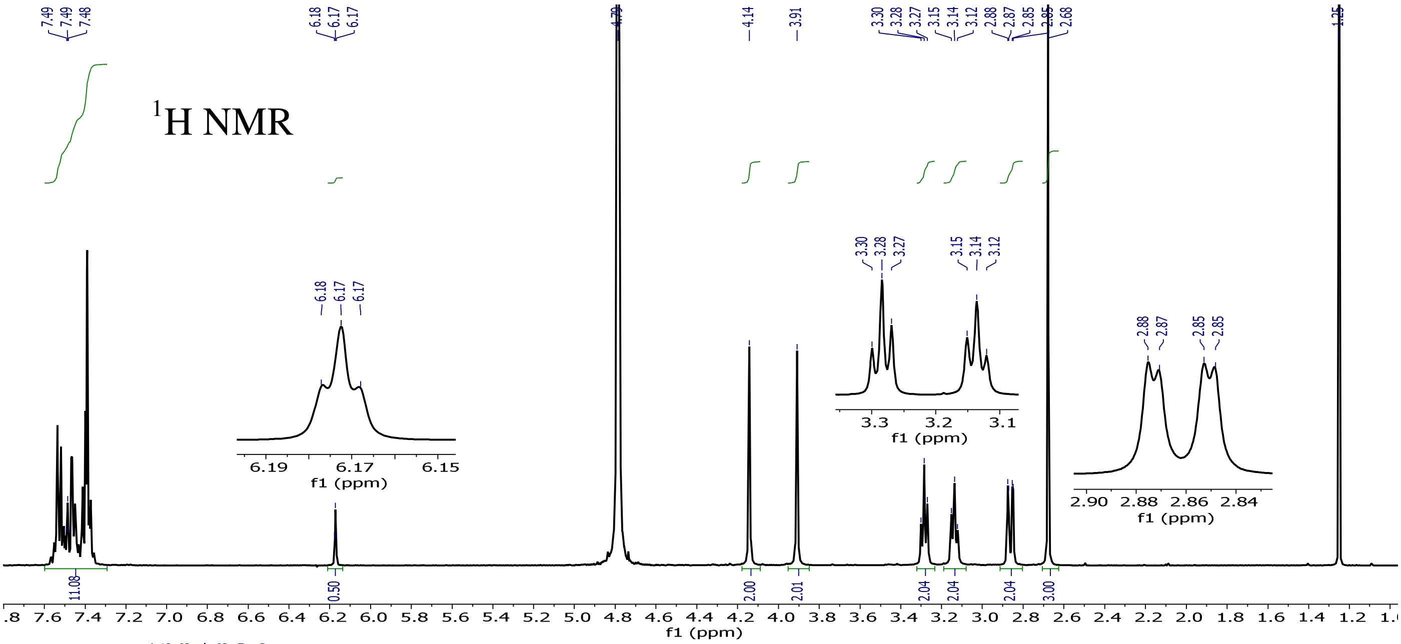
1H NMR
f1 (ppm)

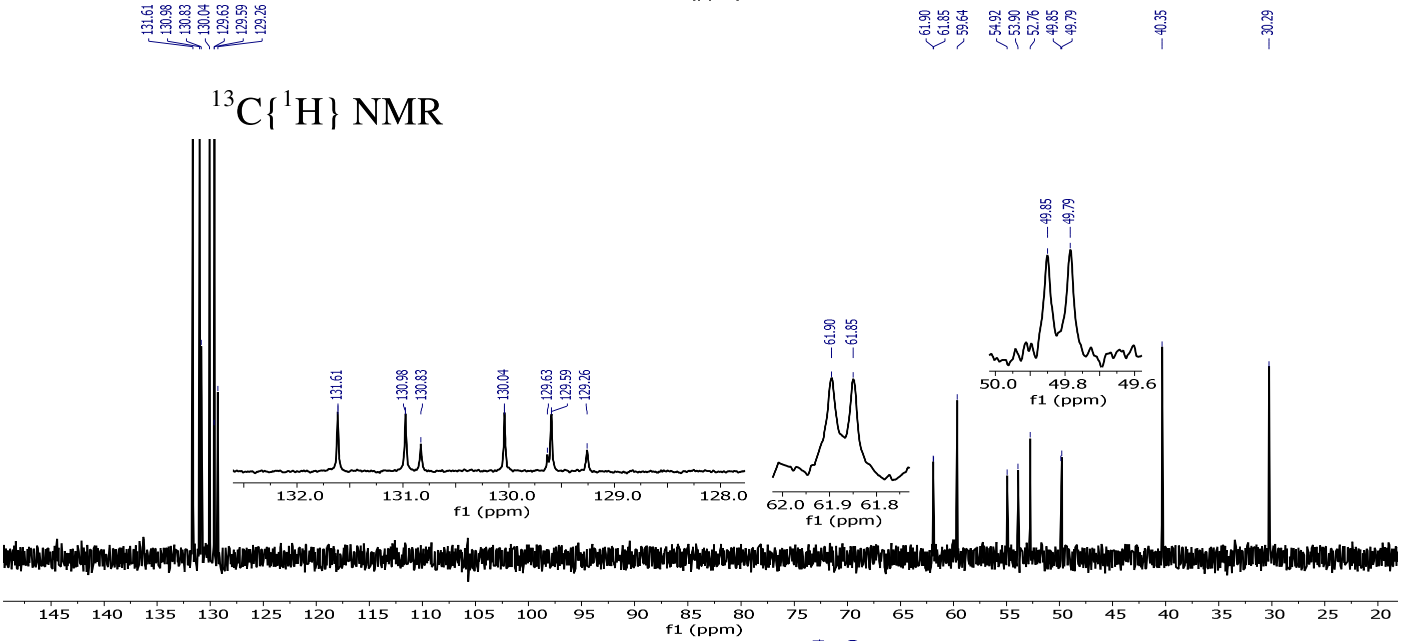
13C{1H} NMR
f1 (ppm)

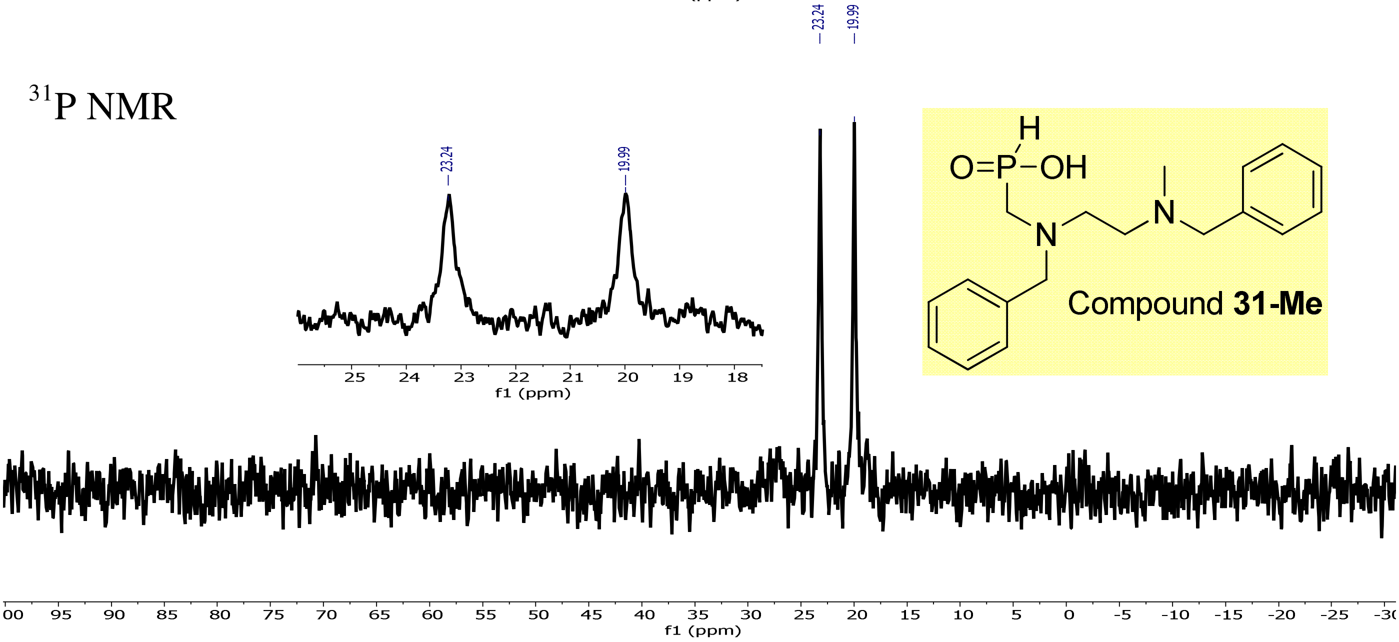
31P NMR
f1 (ppm)
Compound 31-Me

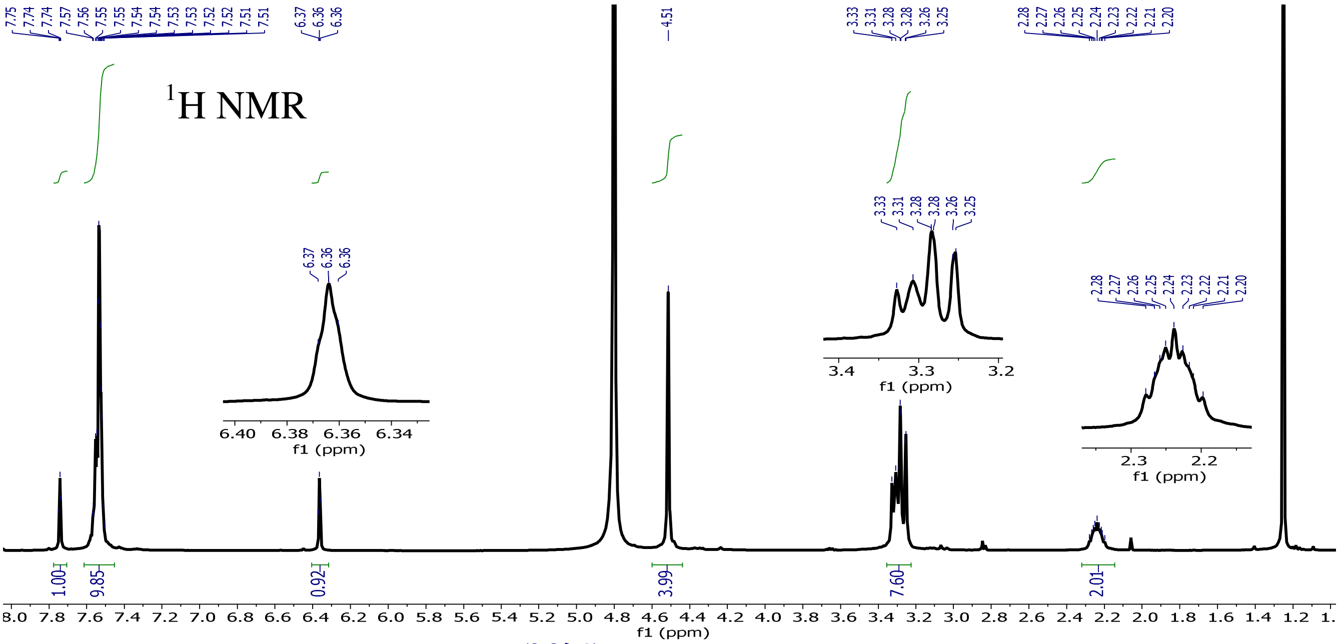

1H NMR
1.00
9.85
0.92
3.99
7.60
2.01
f1 (ppm)

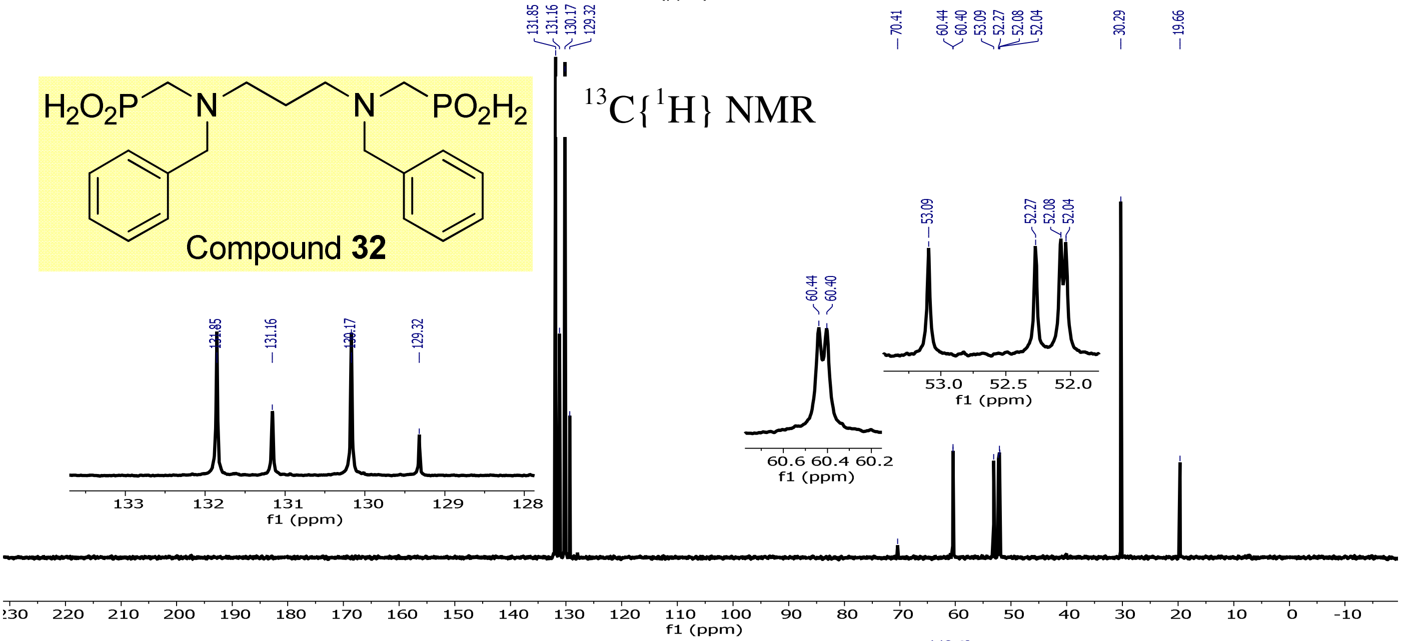

13C{1H} NMR
Compound 32
131.85
131.16
130.17
129.32
70.41
60.44
60.40
53.09
52.27
52.08
52.04
30.29
19.66
f1 (ppm)

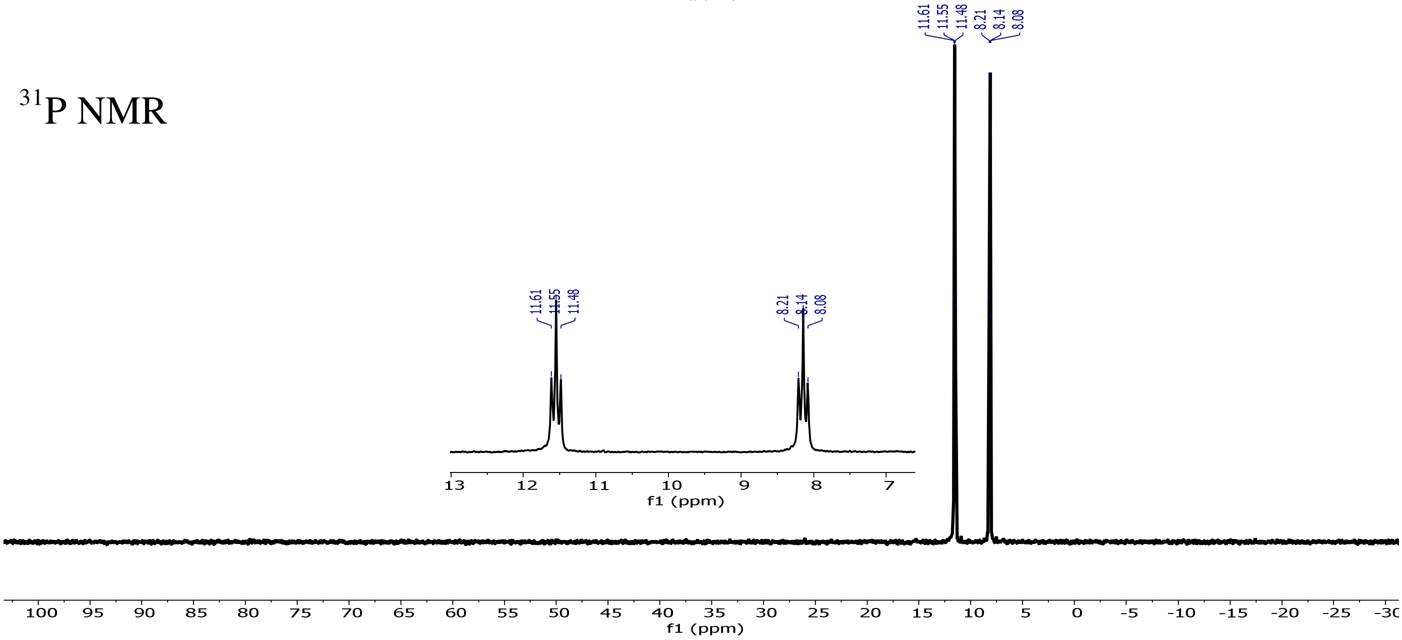

31P NMR
11.61
11.55
11.48
8.21
8.14
8.08
f1 (ppm)

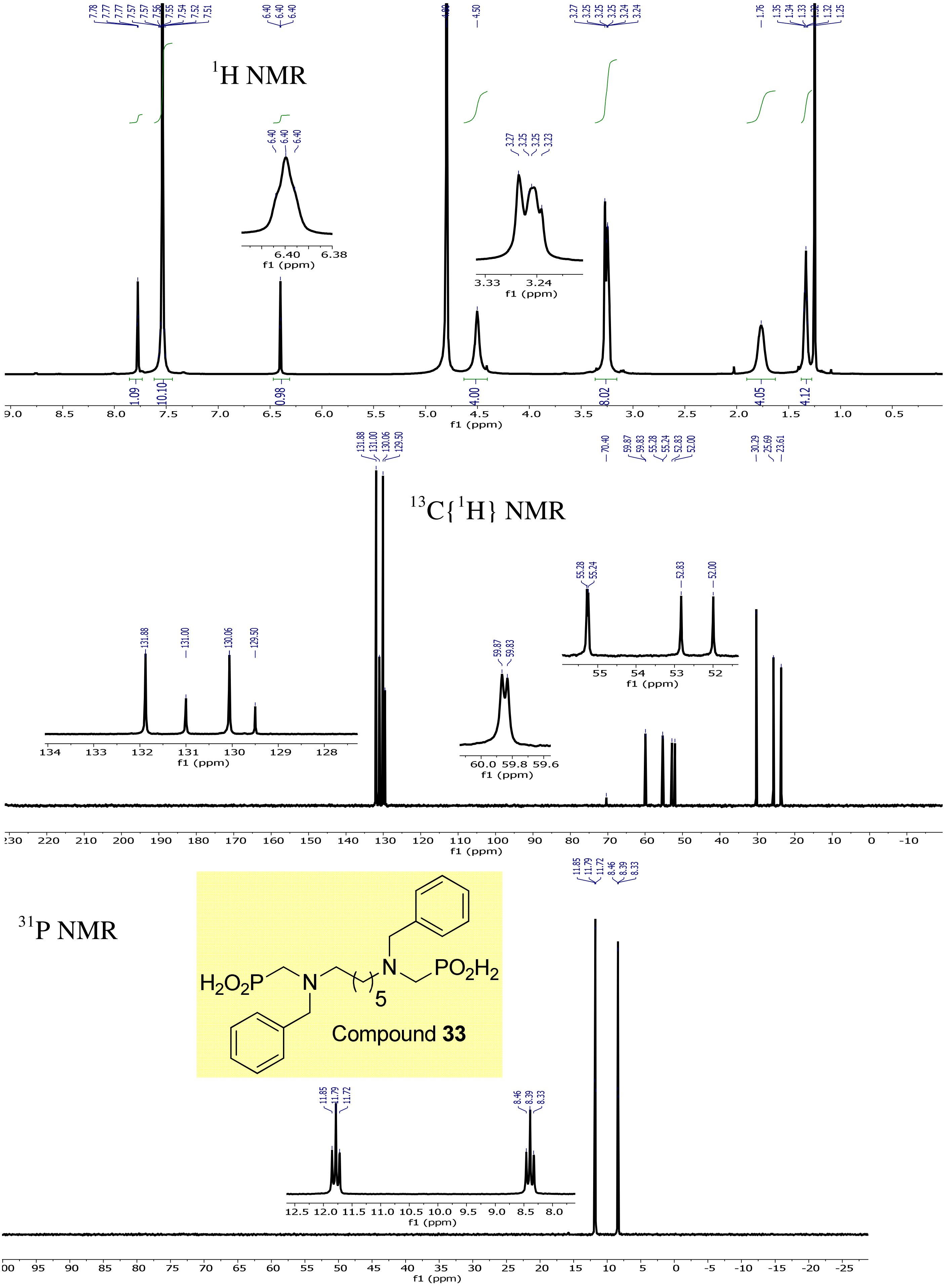

1H NMR
f1 (ppm)
13C{1H} NMR
f1 (ppm)
31P NMR
H2O2P
N
N
PO2H2
5
Compound 33
f1 (ppm)

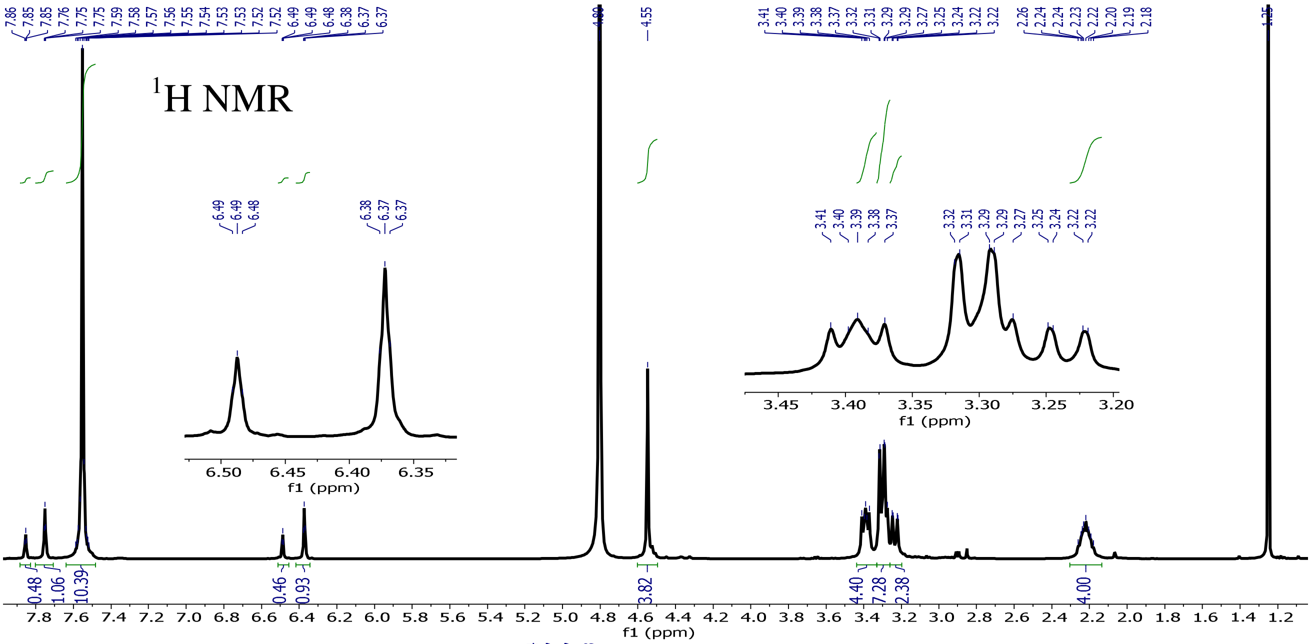
^{1}H NMR
f1 (ppm)

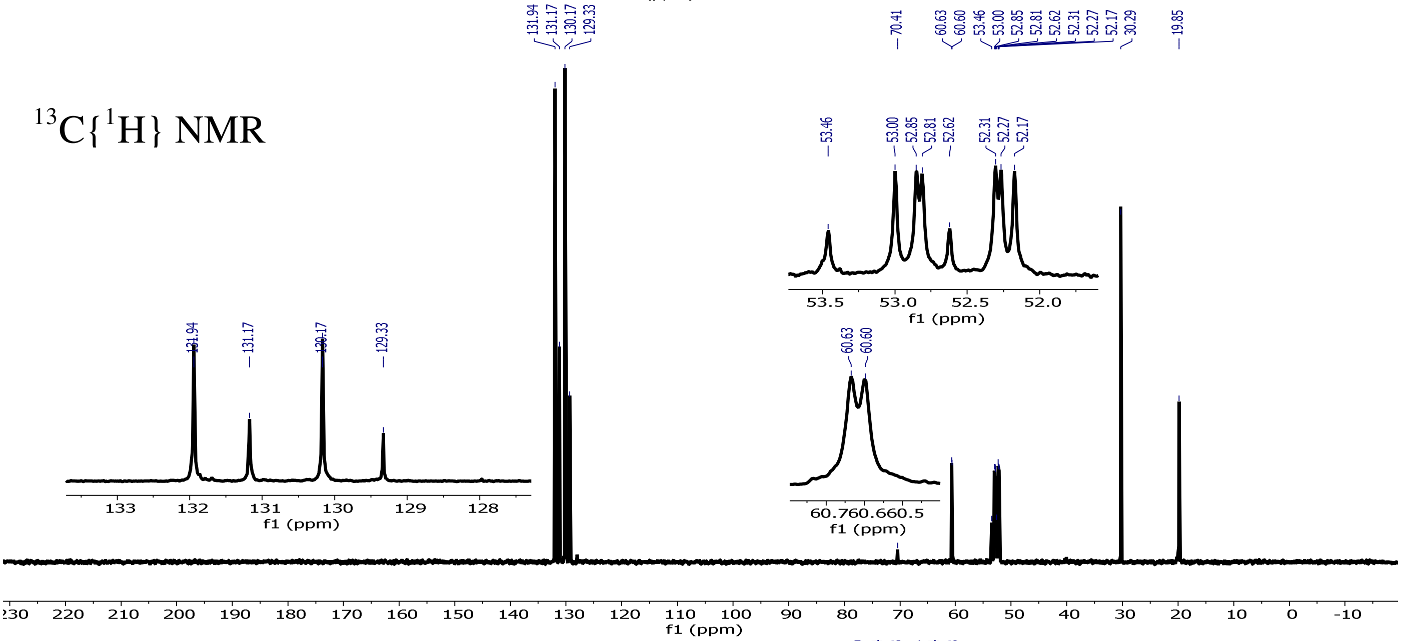
^{13}C{^{1}H} NMR
f1 (ppm)

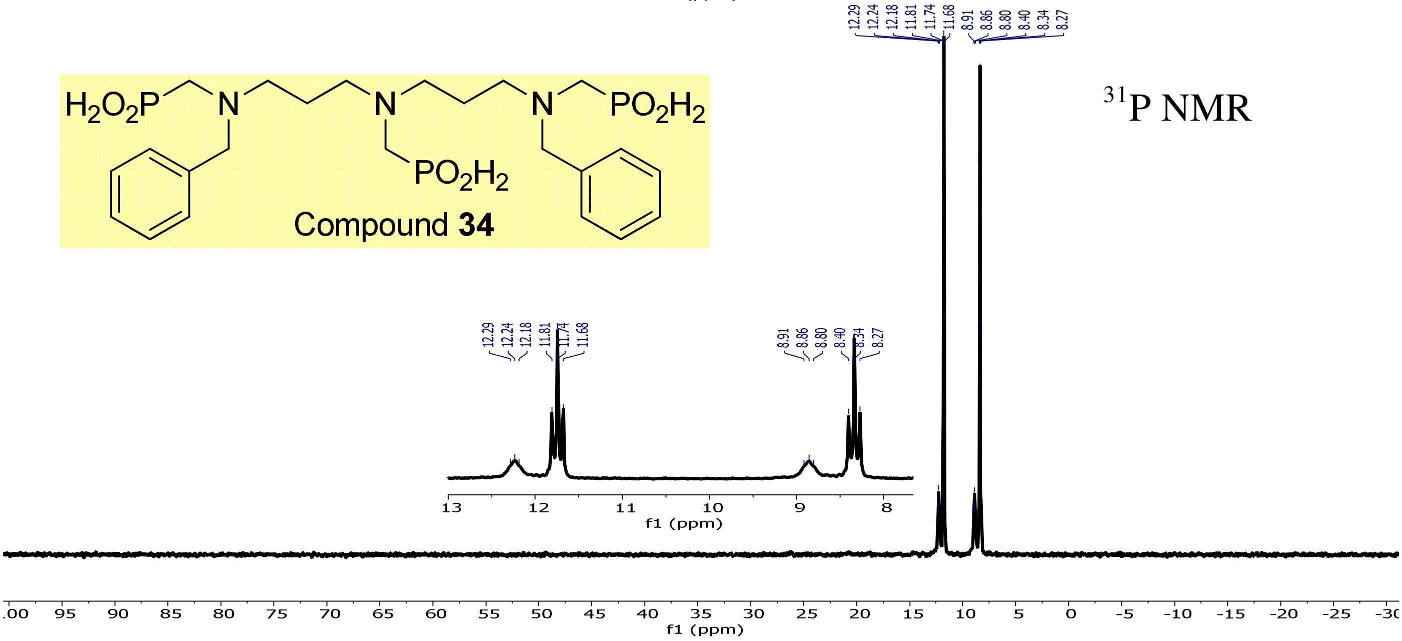
H_2O_2P
N
N
N
PO_2H_2
PO_2H_2
Compound **34**
^{31}P NMR
f1 (ppm)

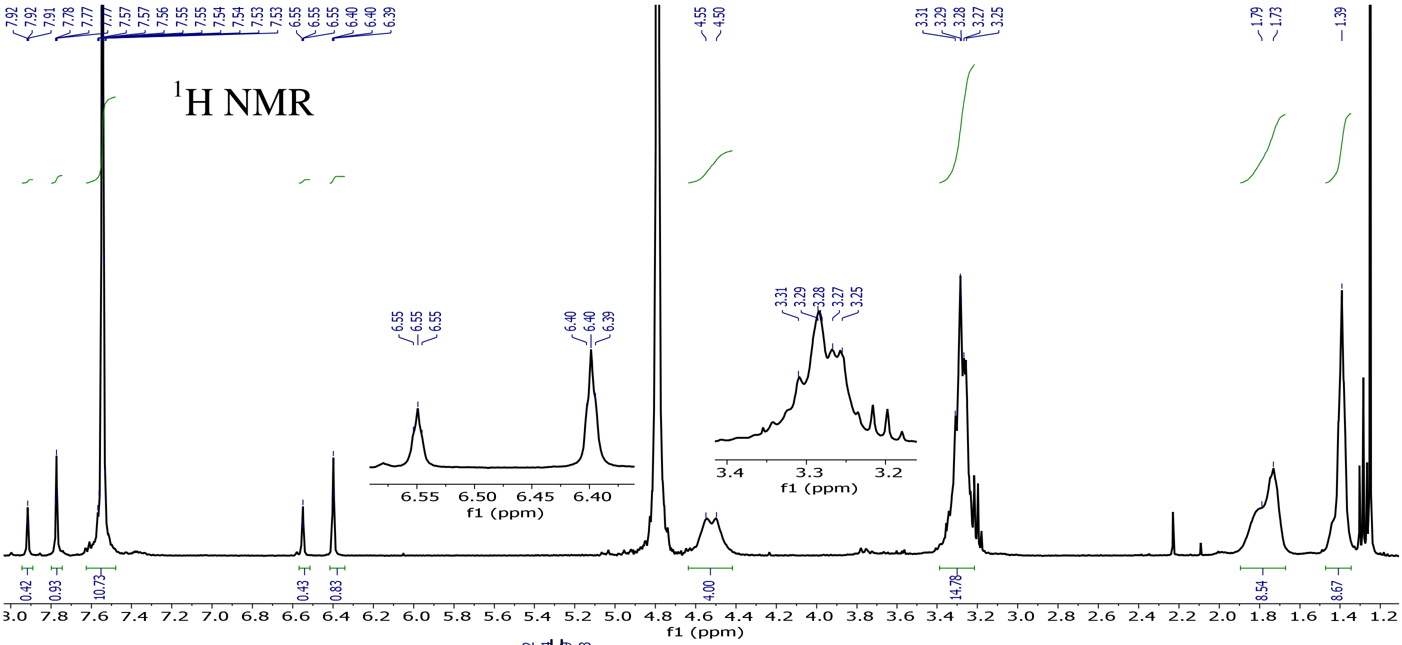
1H NMR
7.92 7.92 7.91 7.78 7.77 7.77 7.57 7.57 7.56 7.55 7.55 7.54 7.54 7.53 7.53 6.55 6.55 6.55 6.40 6.40 6.39 4.55 4.50 3.31 3.29 3.28 3.27 3.25 1.79 1.73 1.39
0.42 0.93 10.73 0.43 0.83 4.00 14.78 8.54 8.67
f1 (ppm)

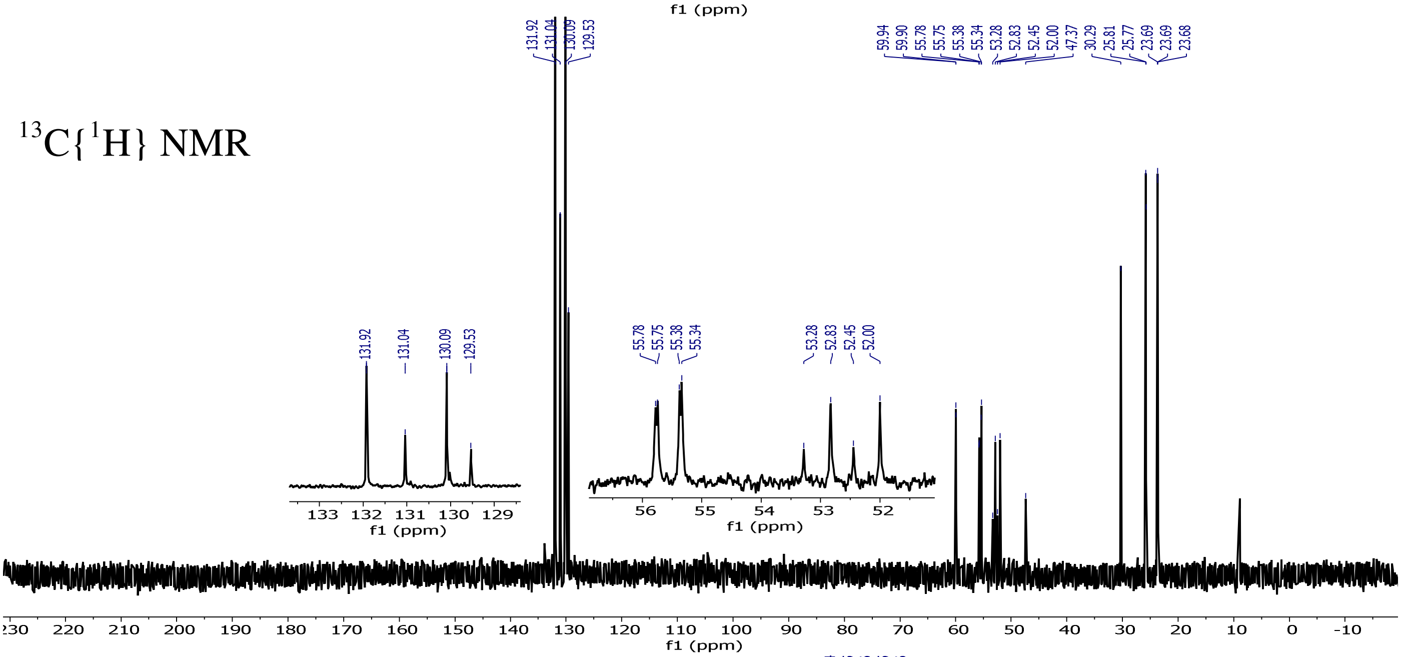
13C{1H} NMR
131.92 131.04 130.09 129.53
59.94 59.90 55.78 55.75 55.38 55.34 53.28 52.83 52.45 52.00 47.37 30.29 25.81 25.77 23.69 23.69 23.68
f1 (ppm)

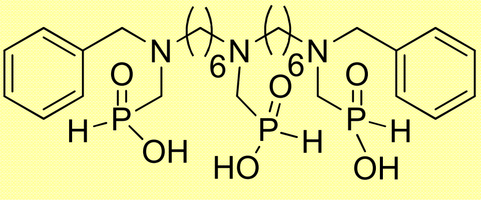
N
6
O
P
H
OH
HO

Compound **35**

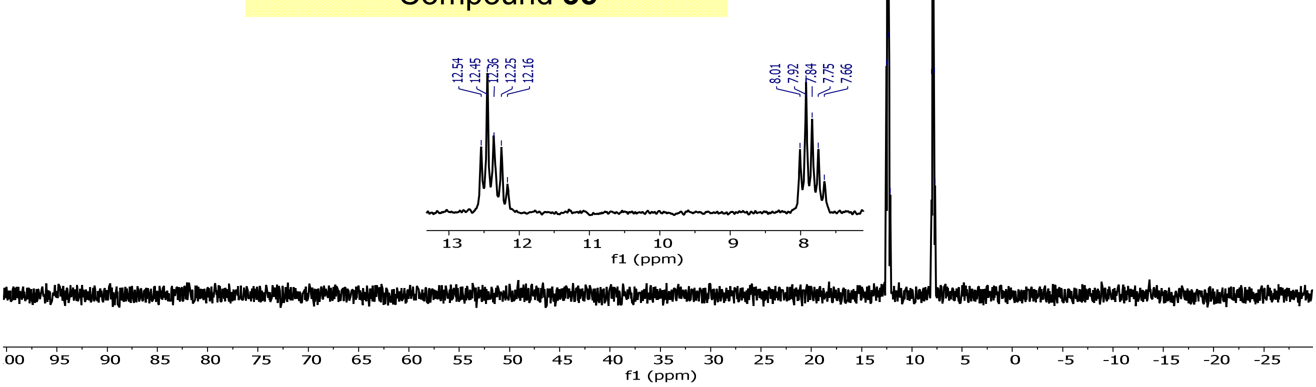
31P NMR
12.54 12.45 12.36 12.25 12.16 8.01 7.92 7.84 7.75 7.66
f1 (ppm)

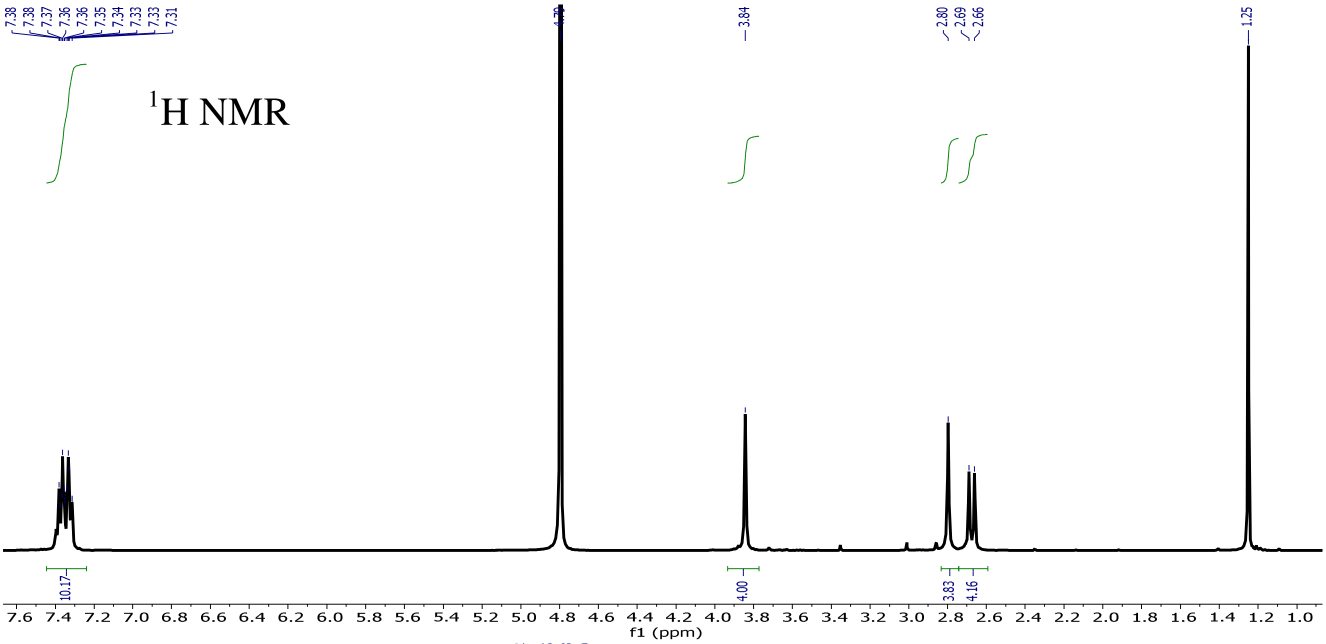
1H NMR
7.38
7.38
7.37
7.36
7.36
7.35
7.34
7.33
7.33
7.31
3.84
2.80
2.69
2.66
1.25
10.17
4.00
3.83
4.16
f1 (ppm)

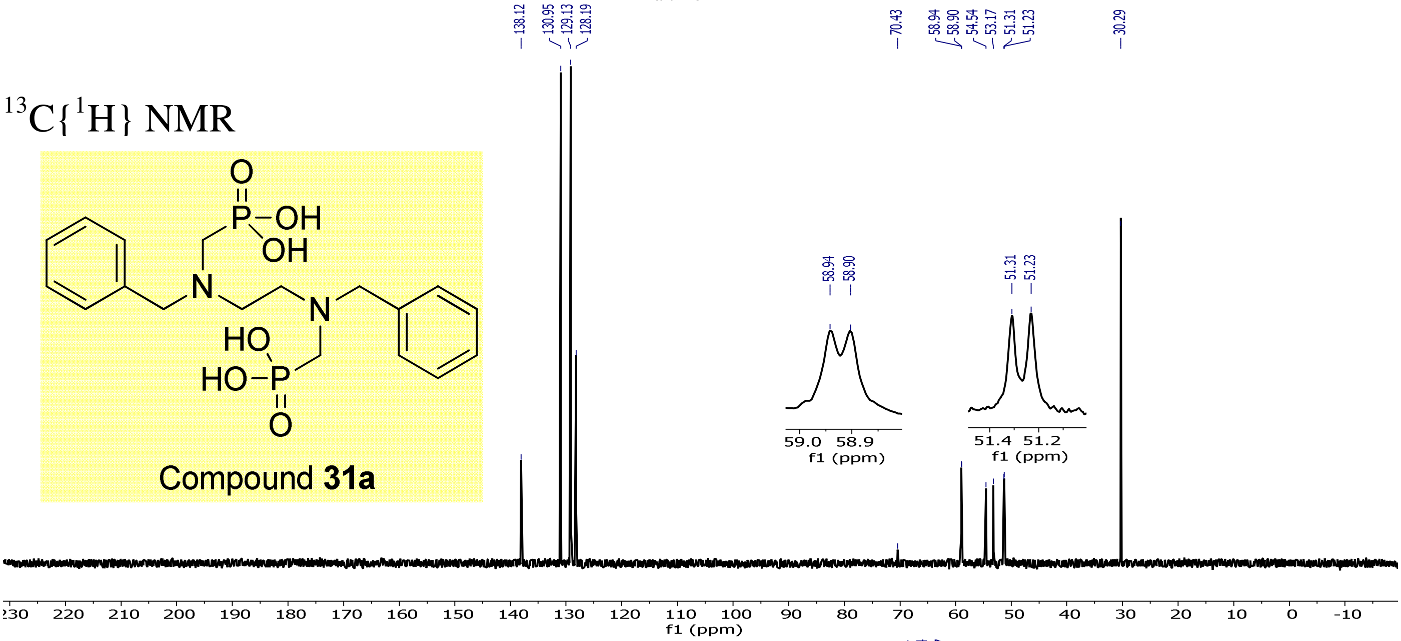
13C{1H} NMR
138.12
130.95
129.13
128.19
70.43
58.94
58.90
54.54
53.17
51.31
51.23
30.29
OH
OH
N
N
HO
HO
P
O
Compound 31a
f1 (ppm)

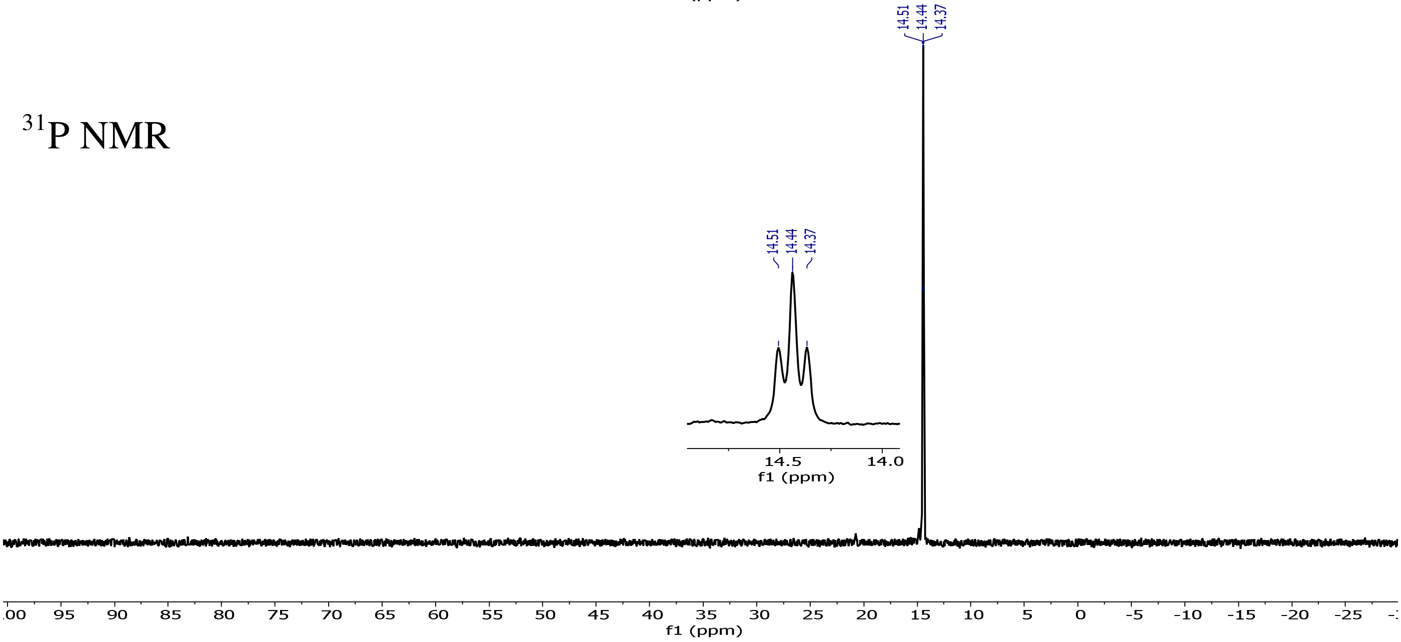
31P NMR
14.51
14.44
14.37
f1 (ppm)

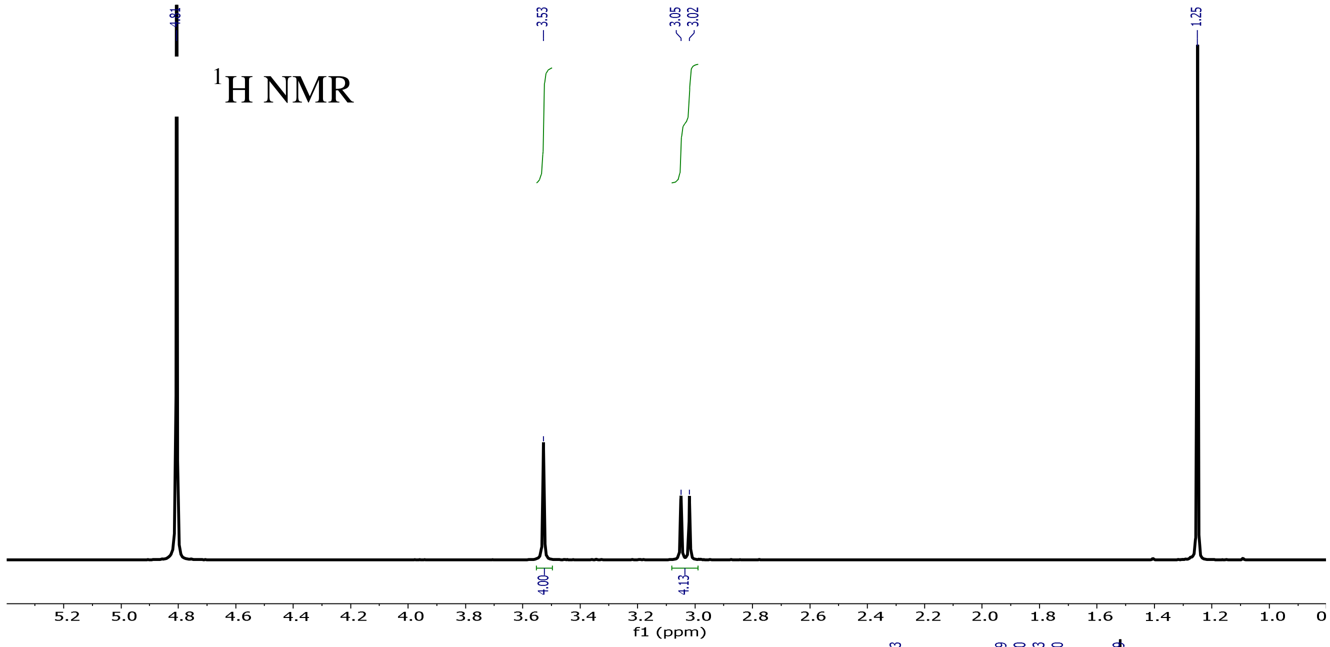

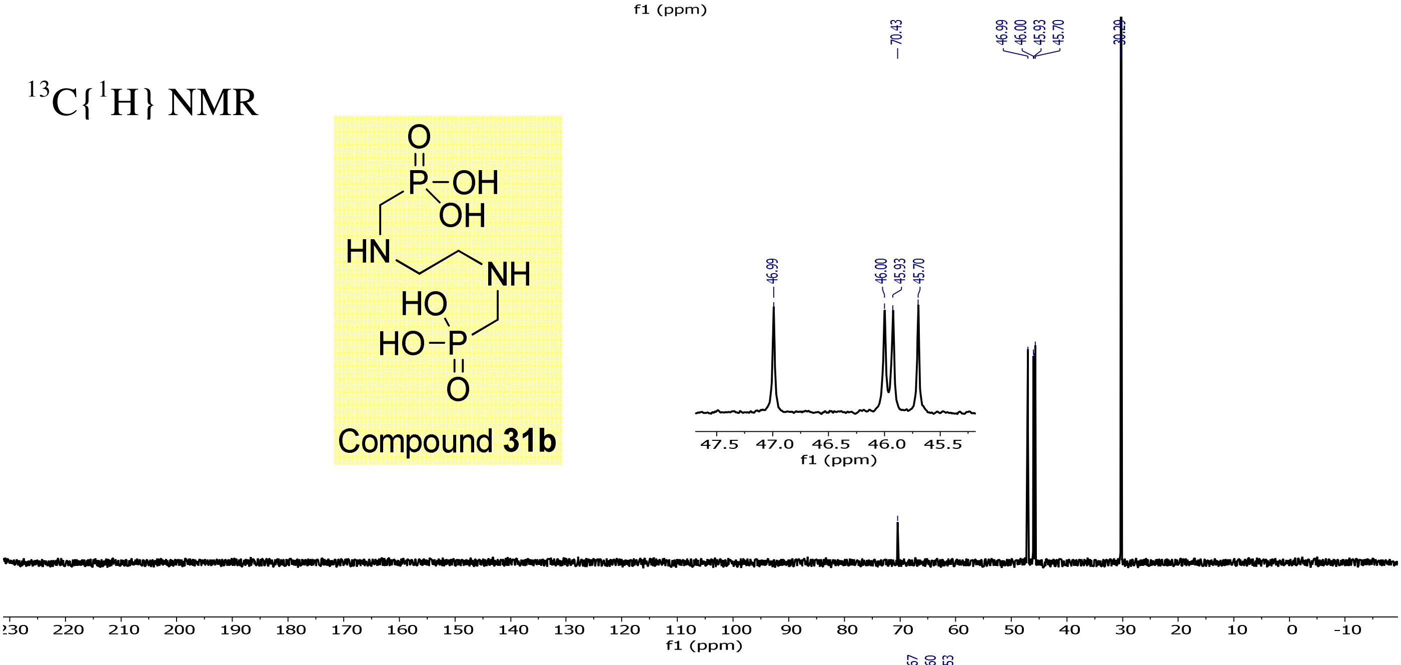

Compound **31b**

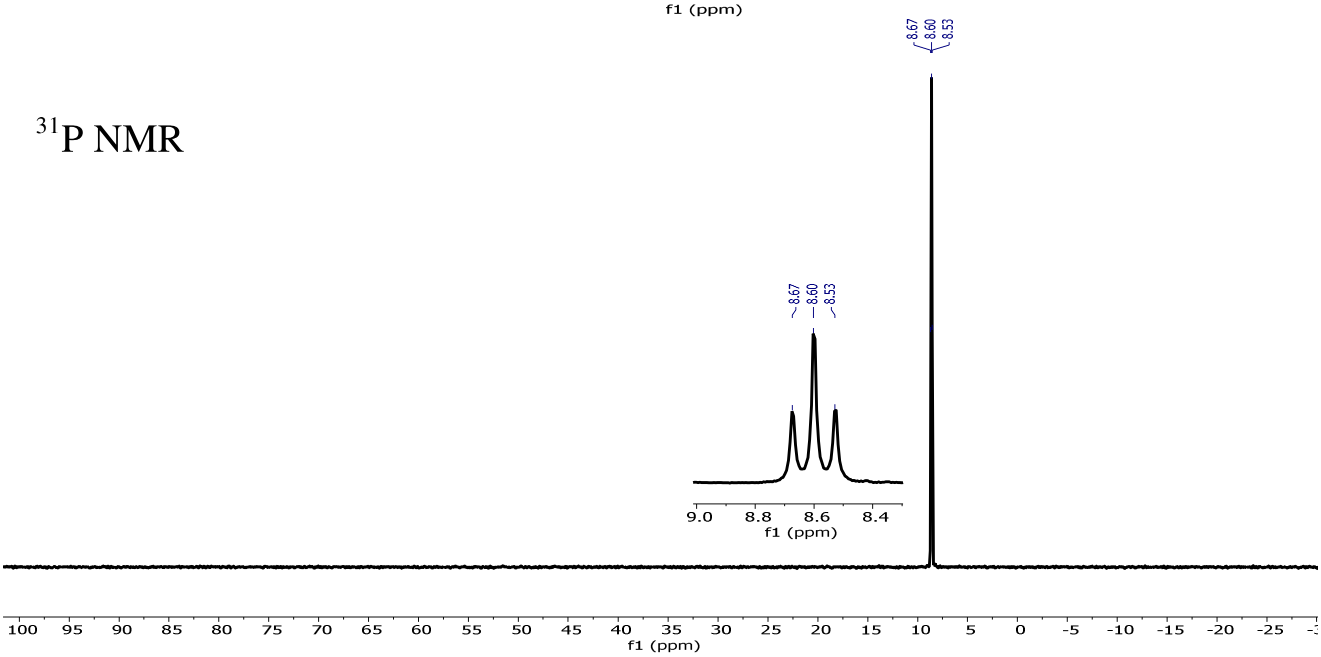

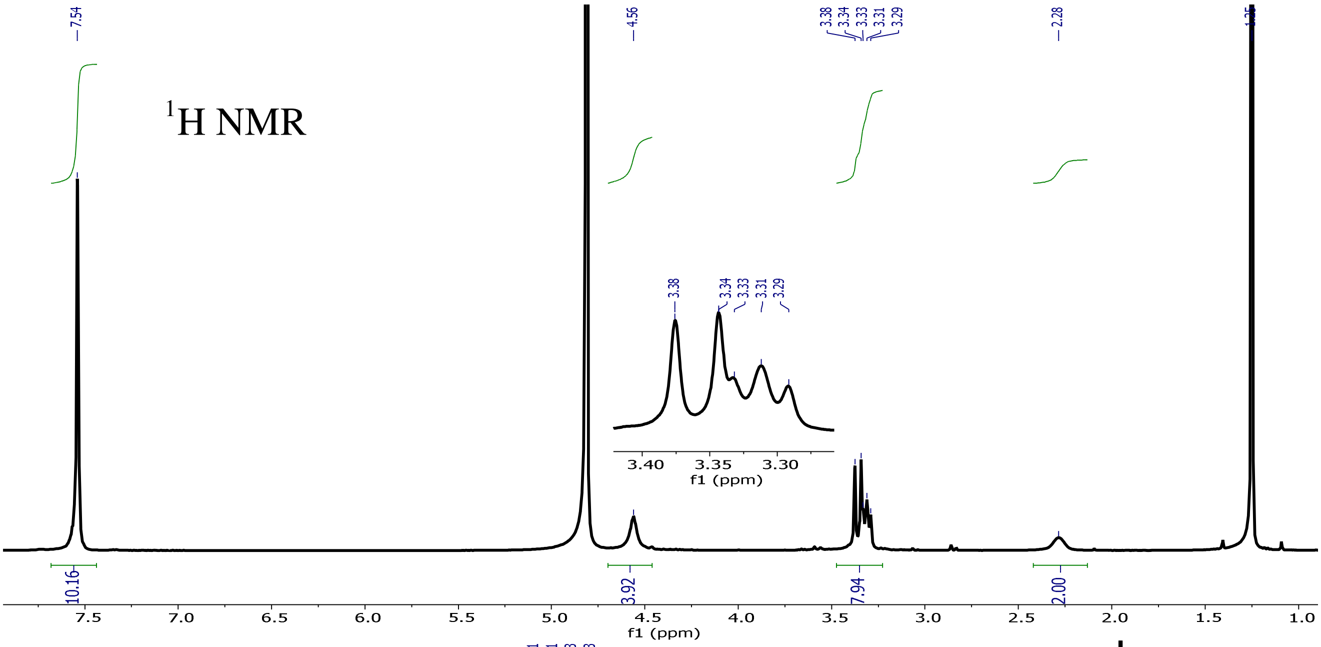

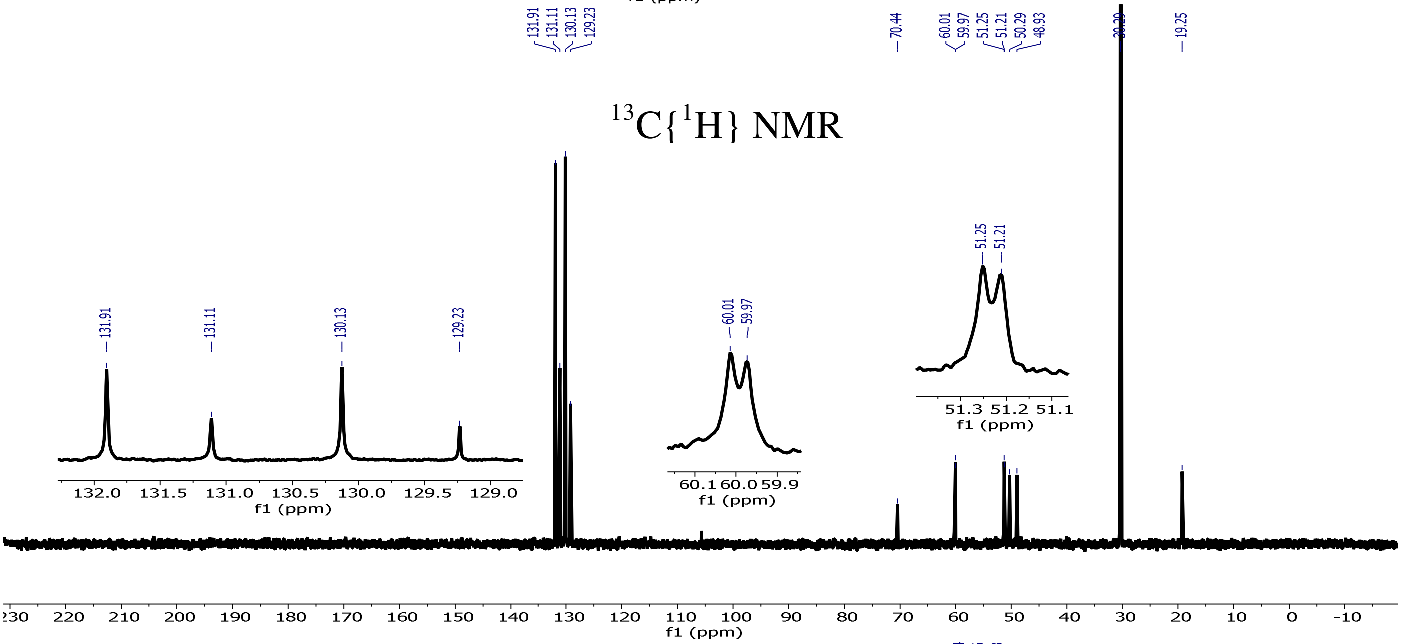

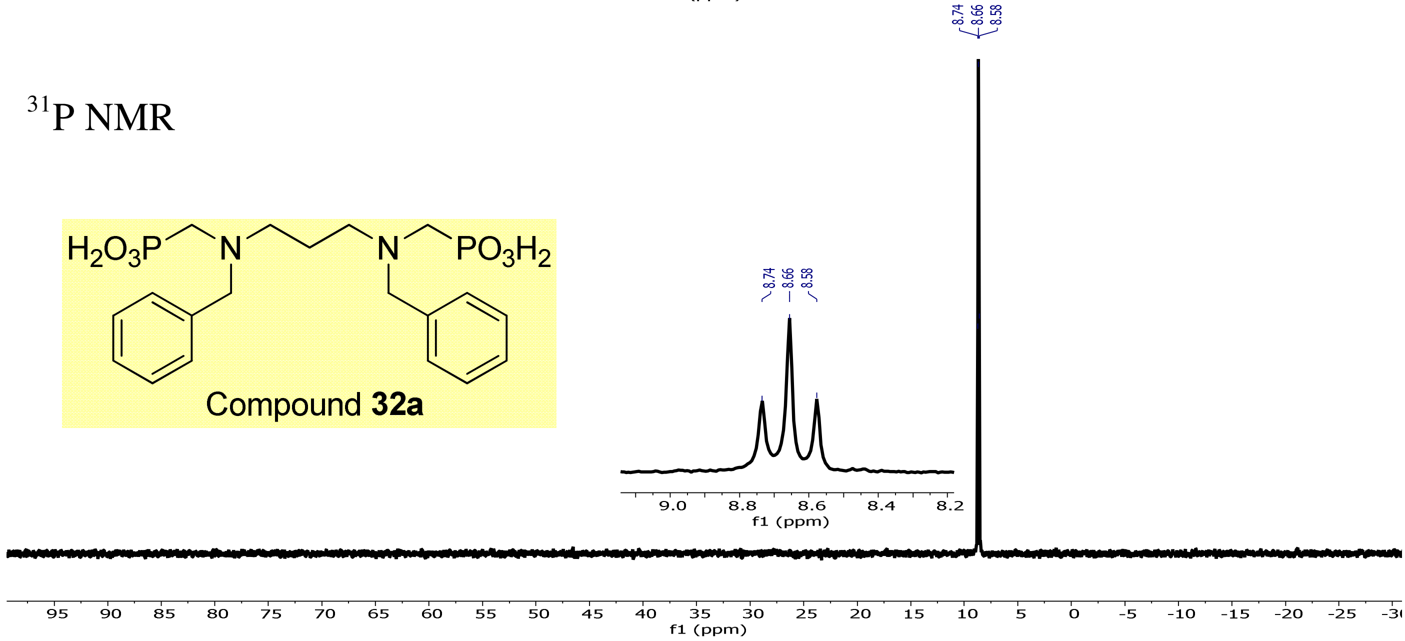

Compound **32a**

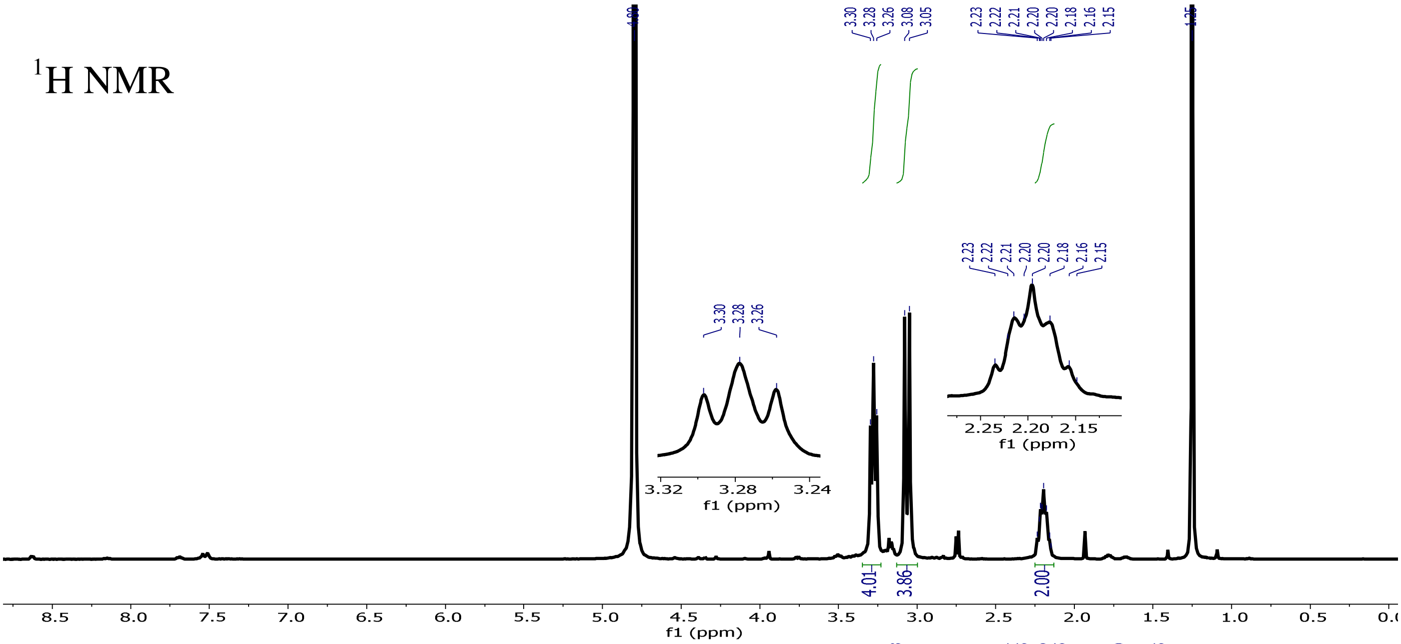

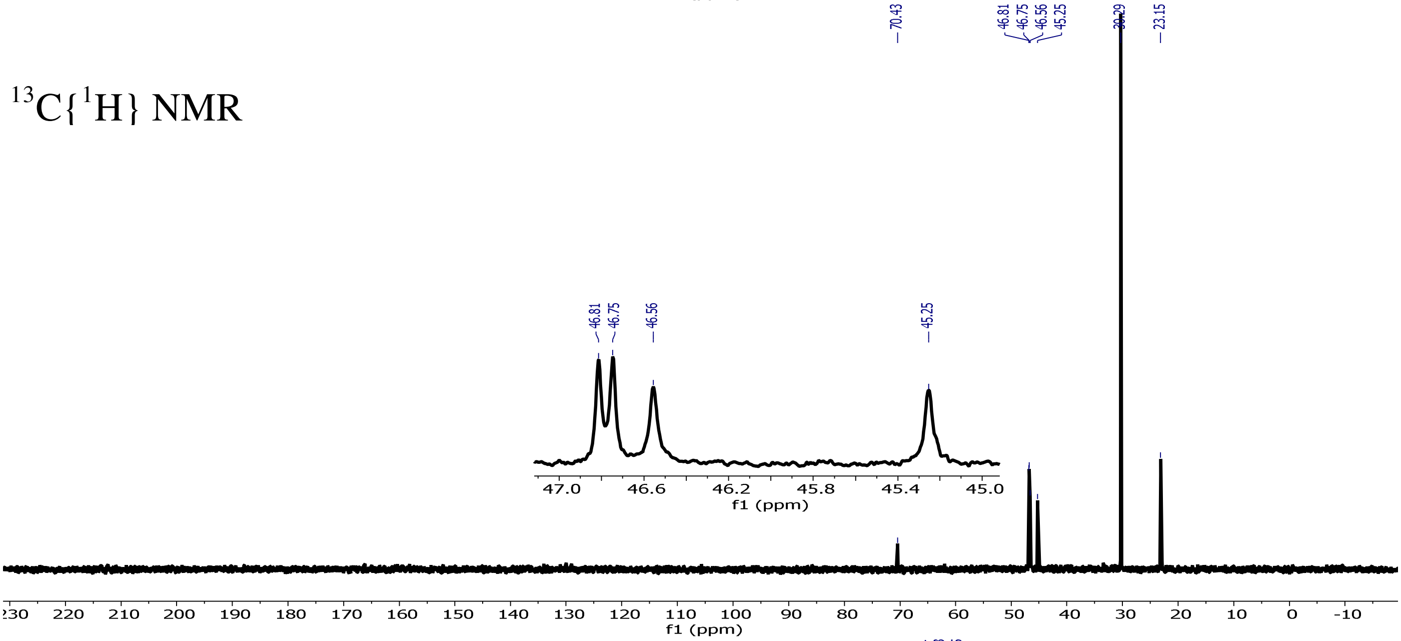

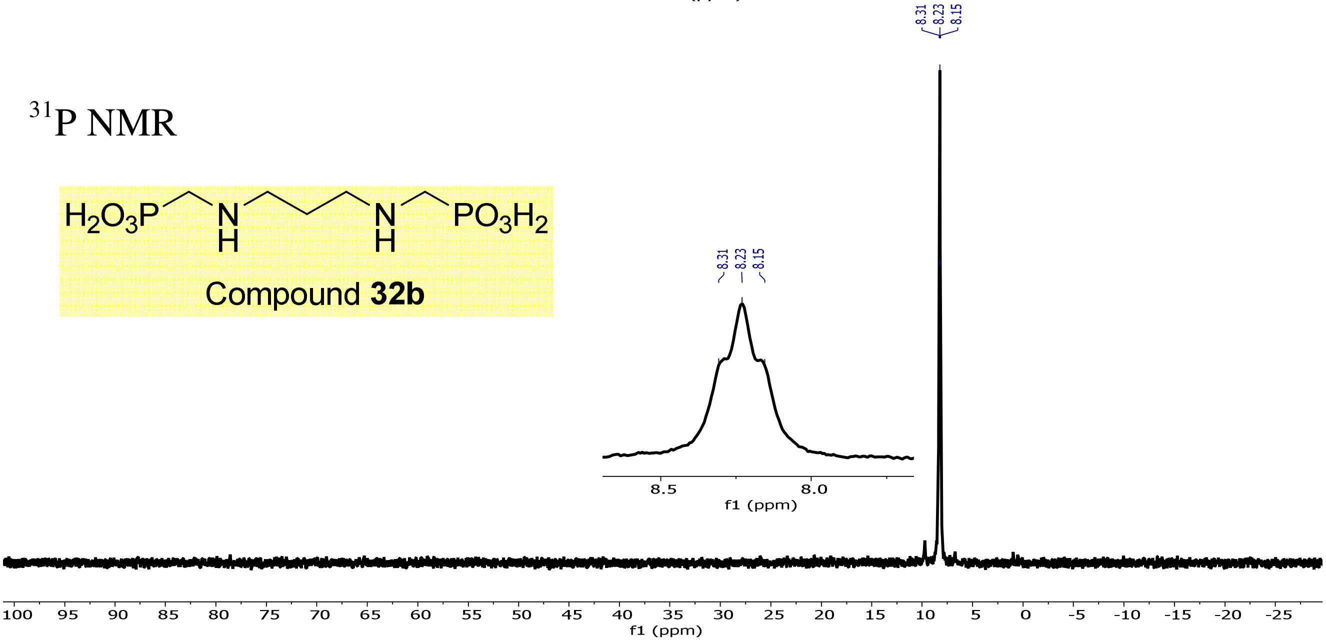

Compound **32b**

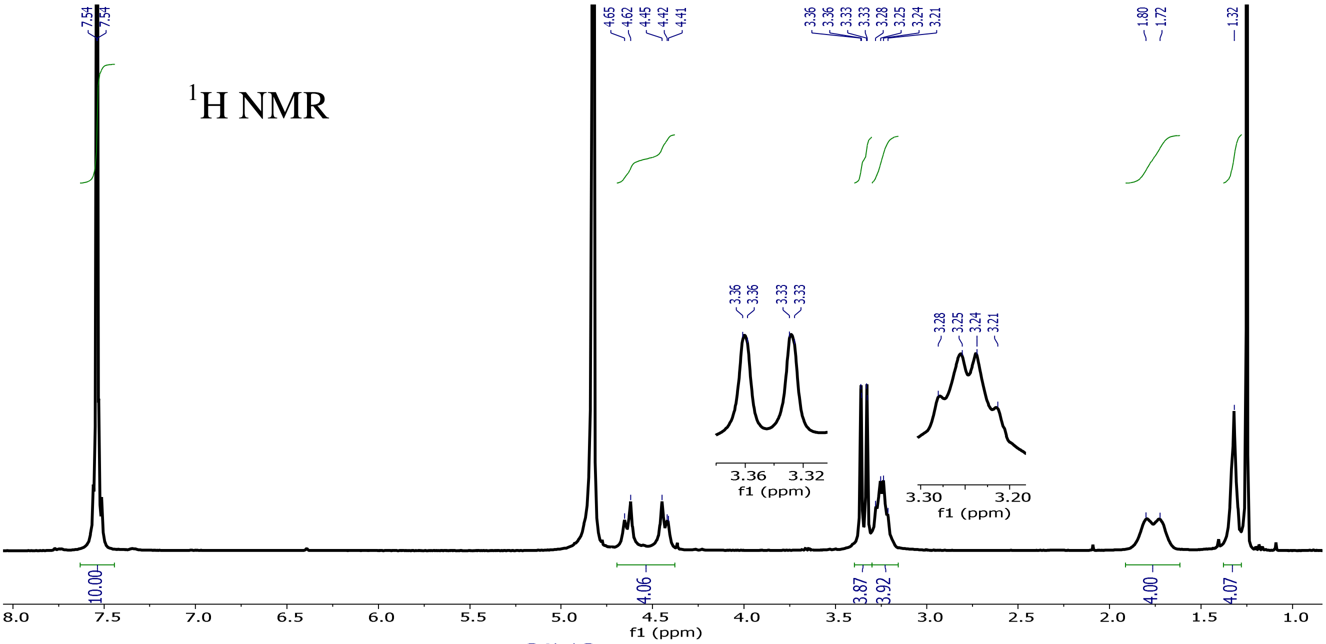

^{1}H NMR
7.54 7.54
4.65 4.62 4.45 4.42 4.41
3.36 3.36 3.33 3.33 3.28 3.25 3.24 3.21
1.80 1.72
1.32
10.00 4.06 3.87 3.92 4.00 4.07
f1 (ppm)

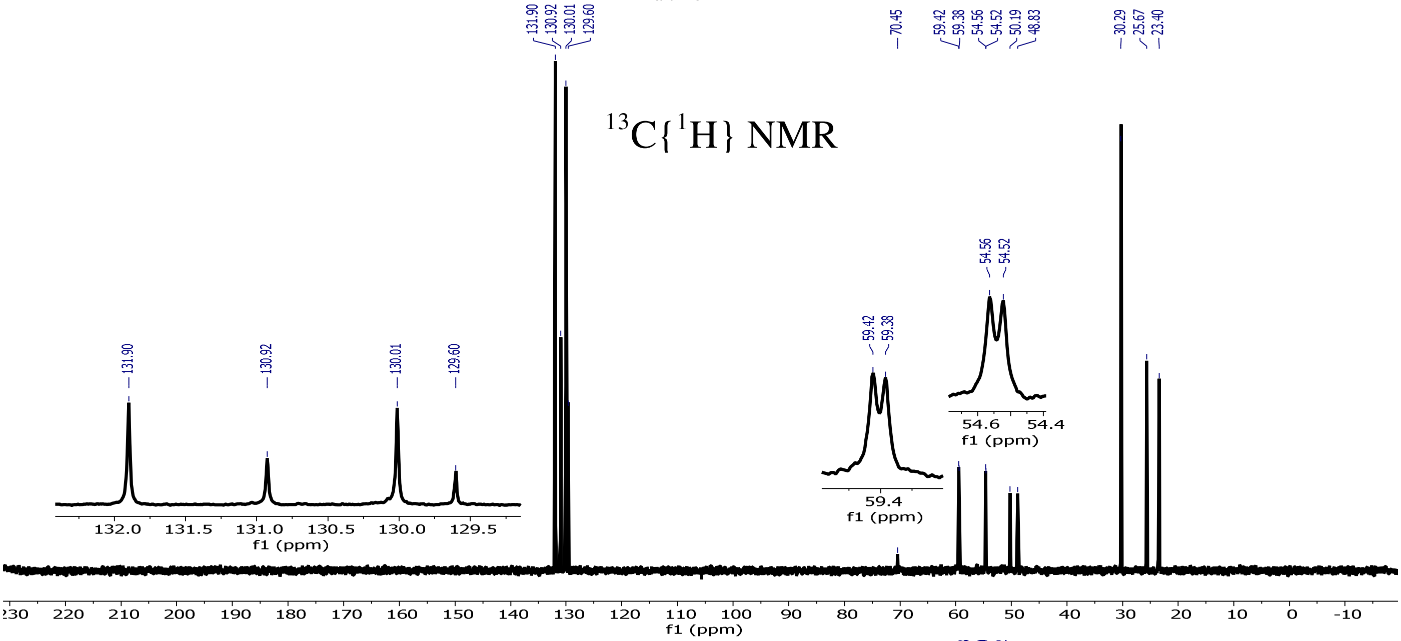

$^{13}C\{^{1}H\}$ NMR
131.90 130.92 130.01 129.60
70.45
59.42 59.38 54.56 54.52 50.19 48.83
30.29 25.67 23.40
f1 (ppm)

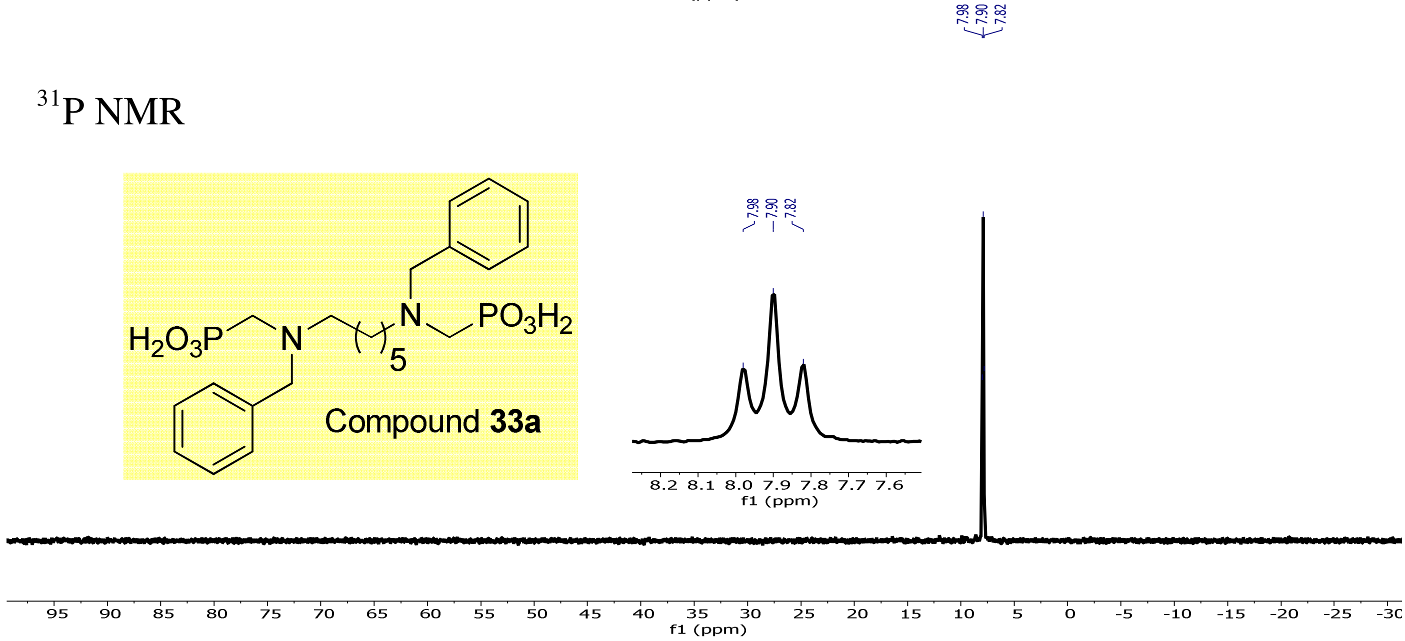

^{31}P NMR
7.98 7.90 7.82
H_2O_3P
PO_3H_2
N
5
Compound **33a**
f1 (ppm)

^{1}H NMR

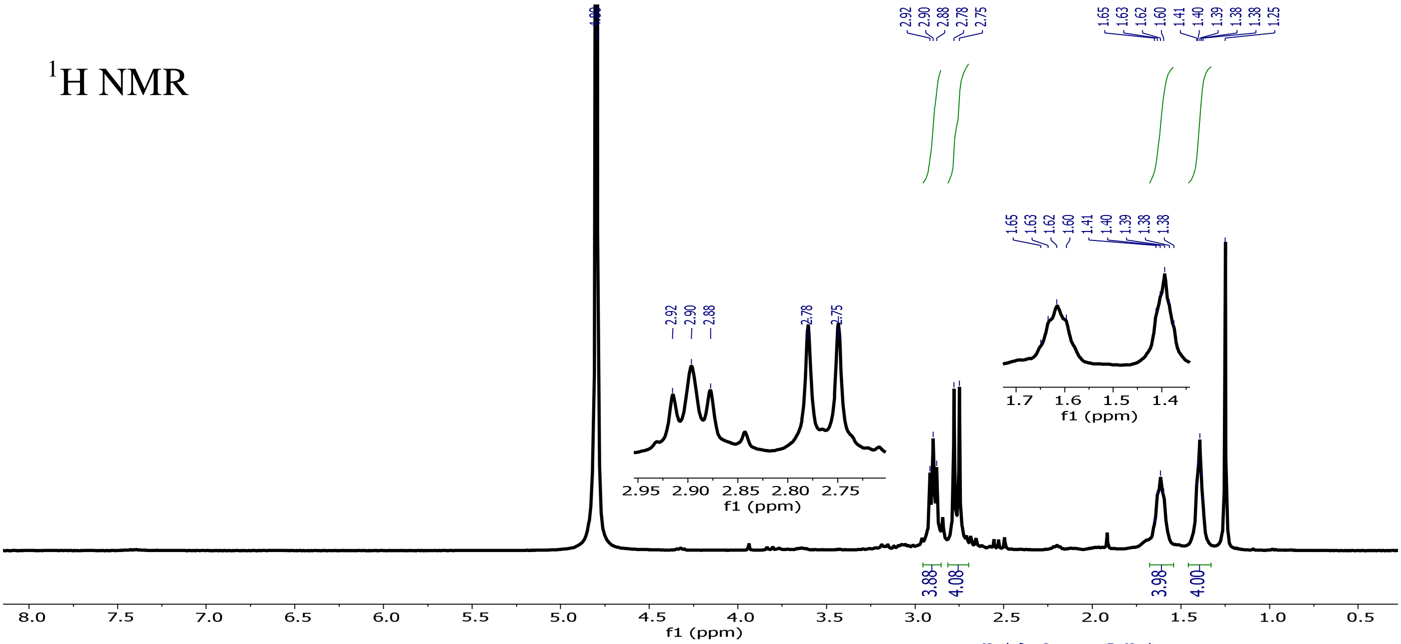

$^{13}C\{^{1}H\}$ NMR

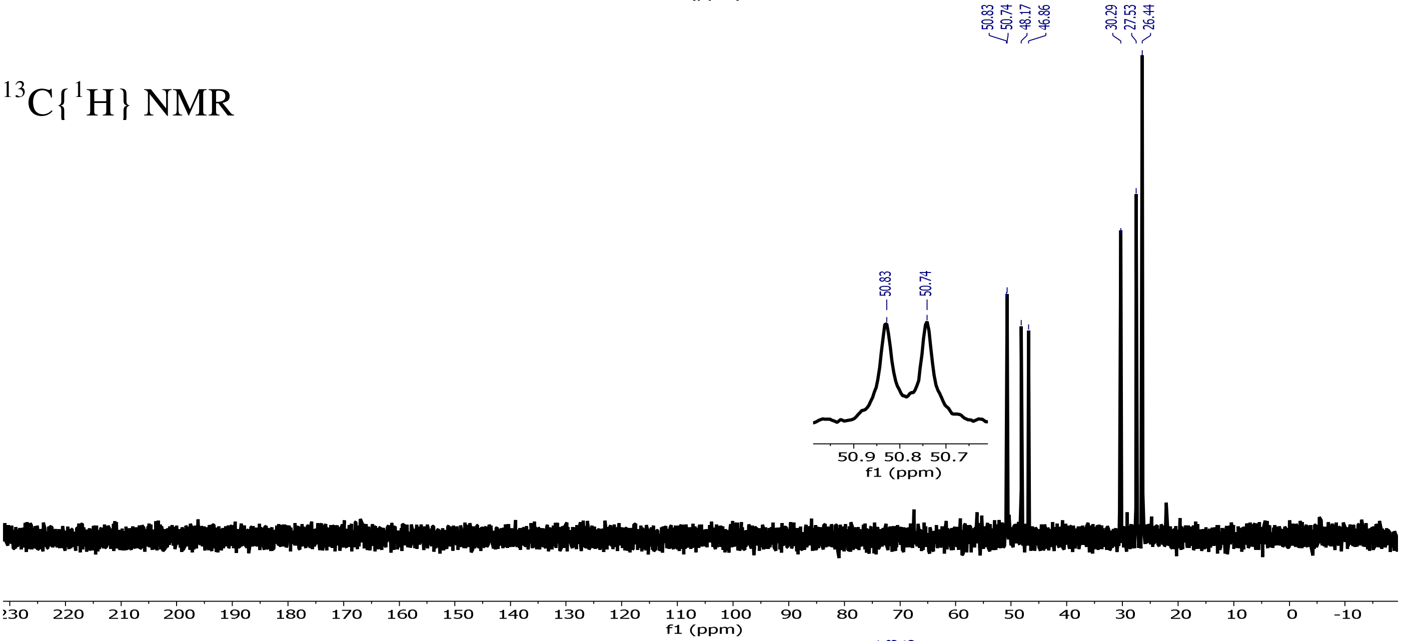

^{31}P NMR

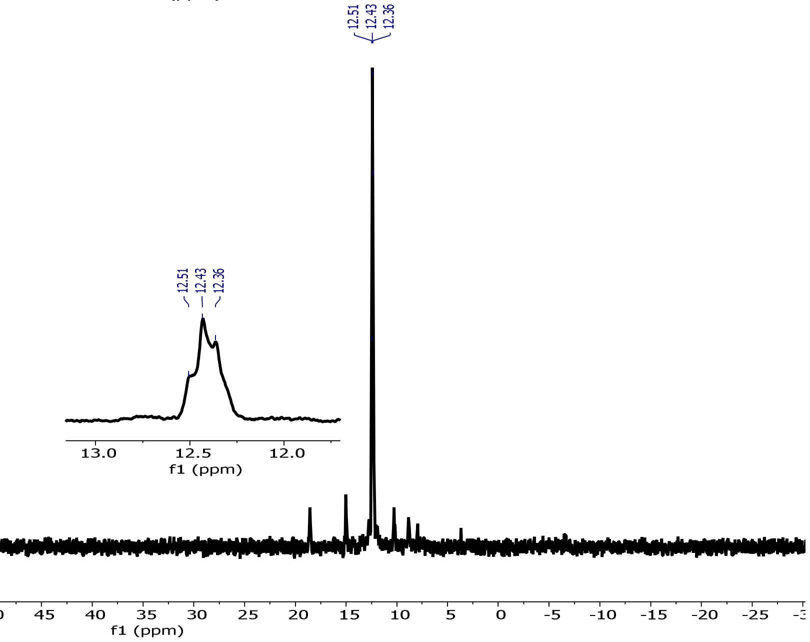

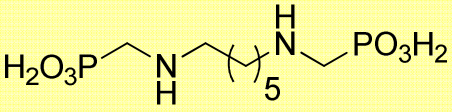

Compound **33b**

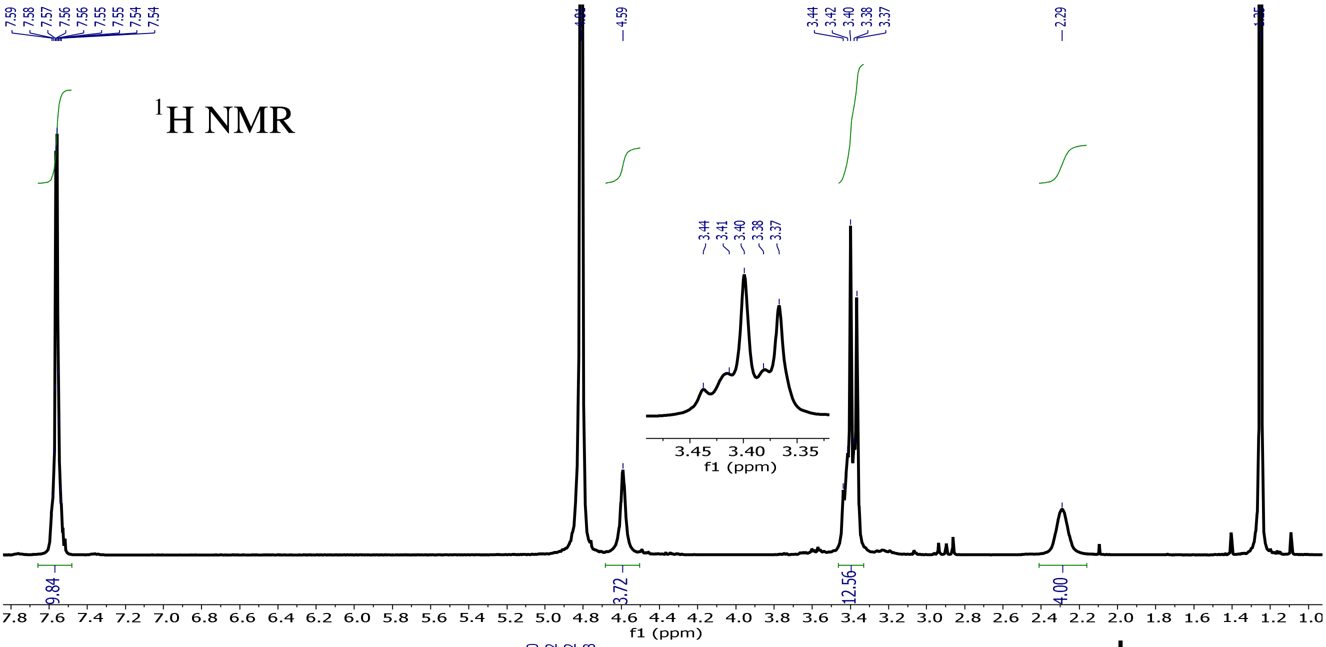

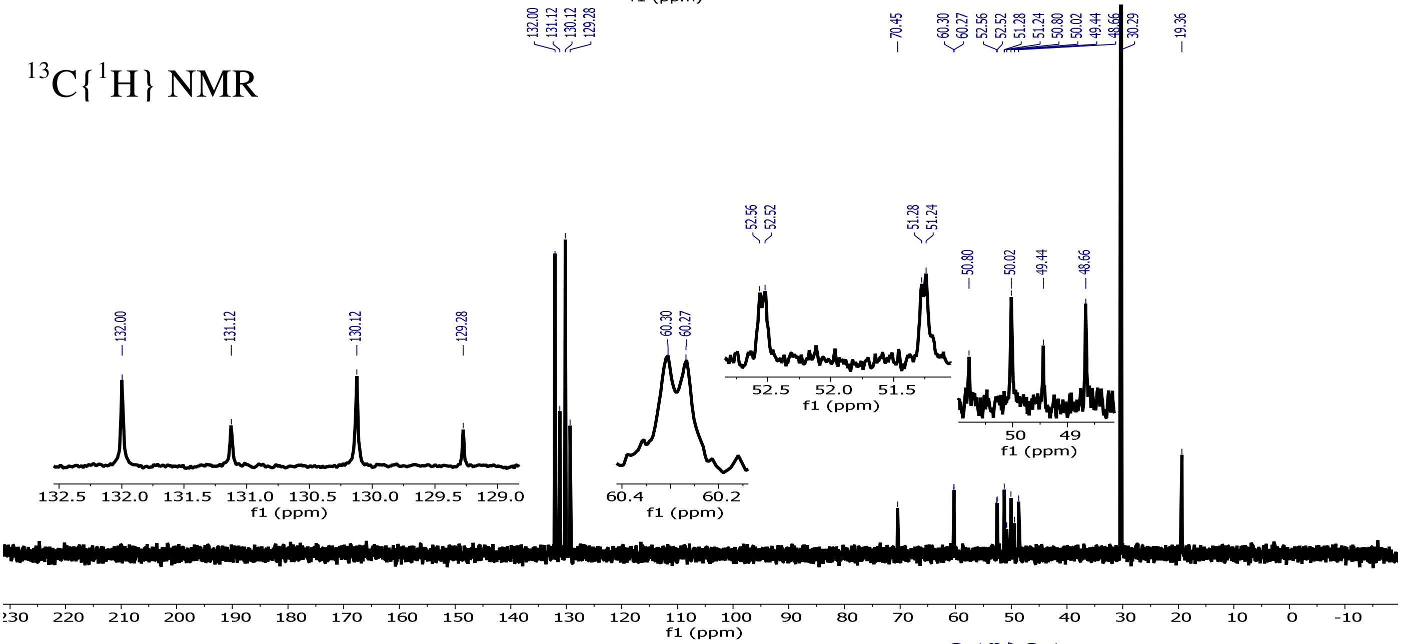

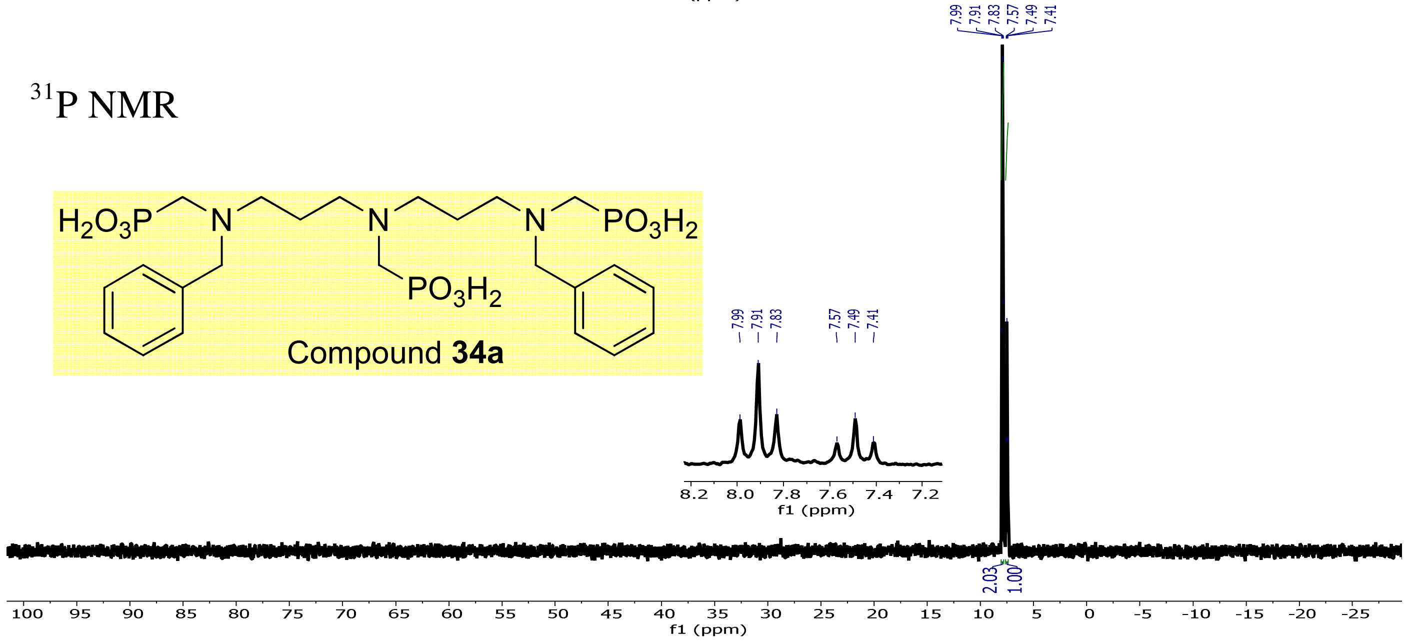

Compound **34a**

^{1}H NMR

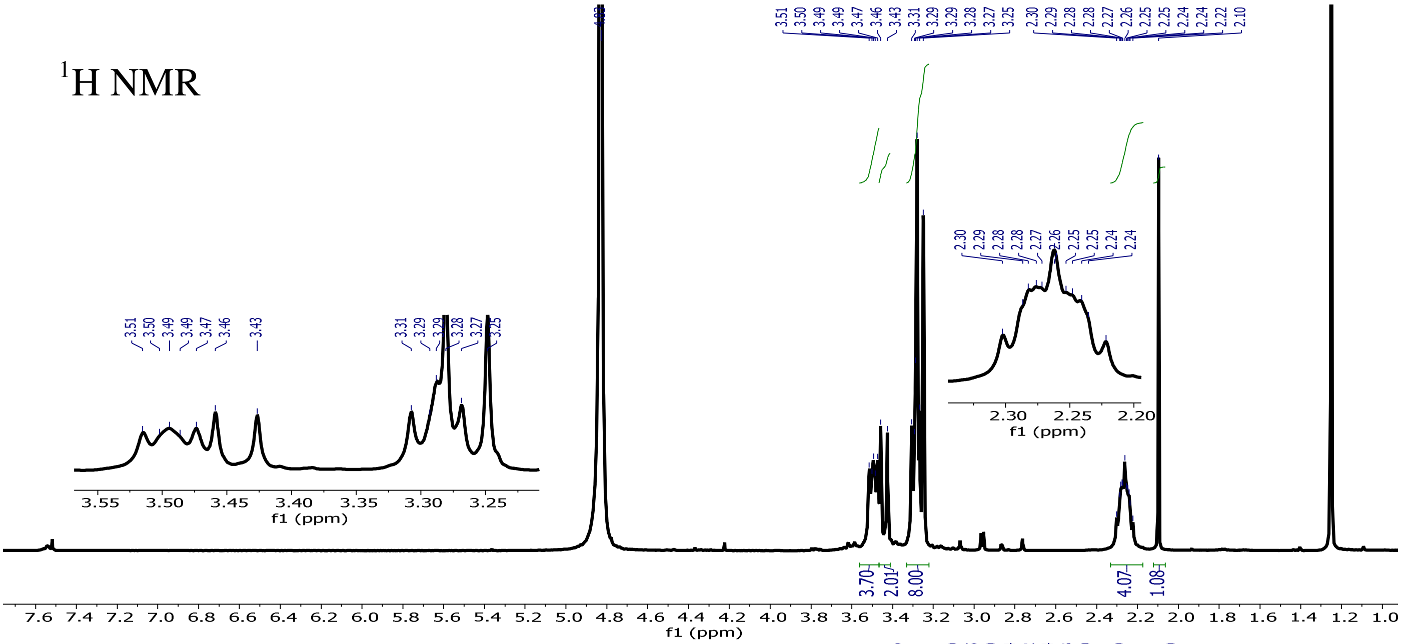

^{13}C{^{1}H} NMR

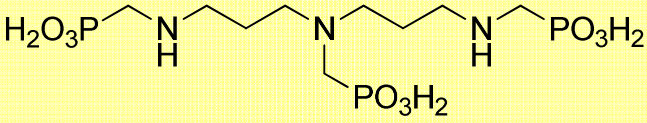

Compound **34b**

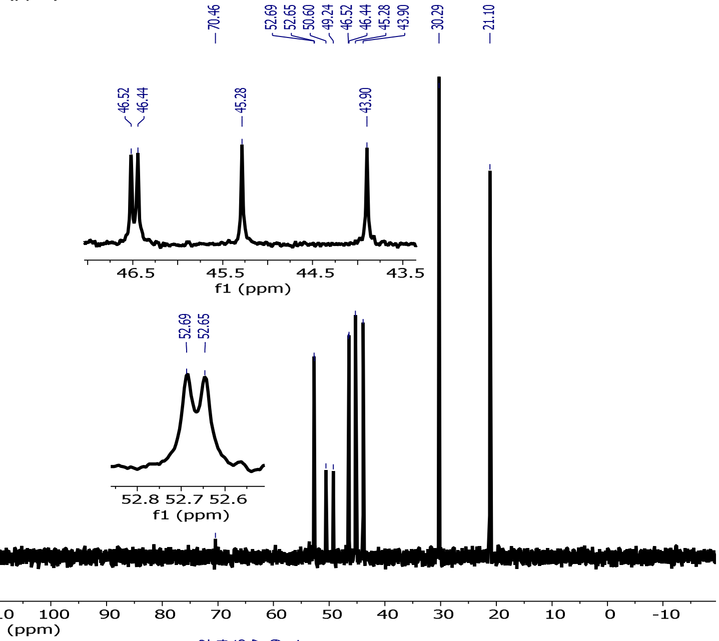

^{31}P NMR

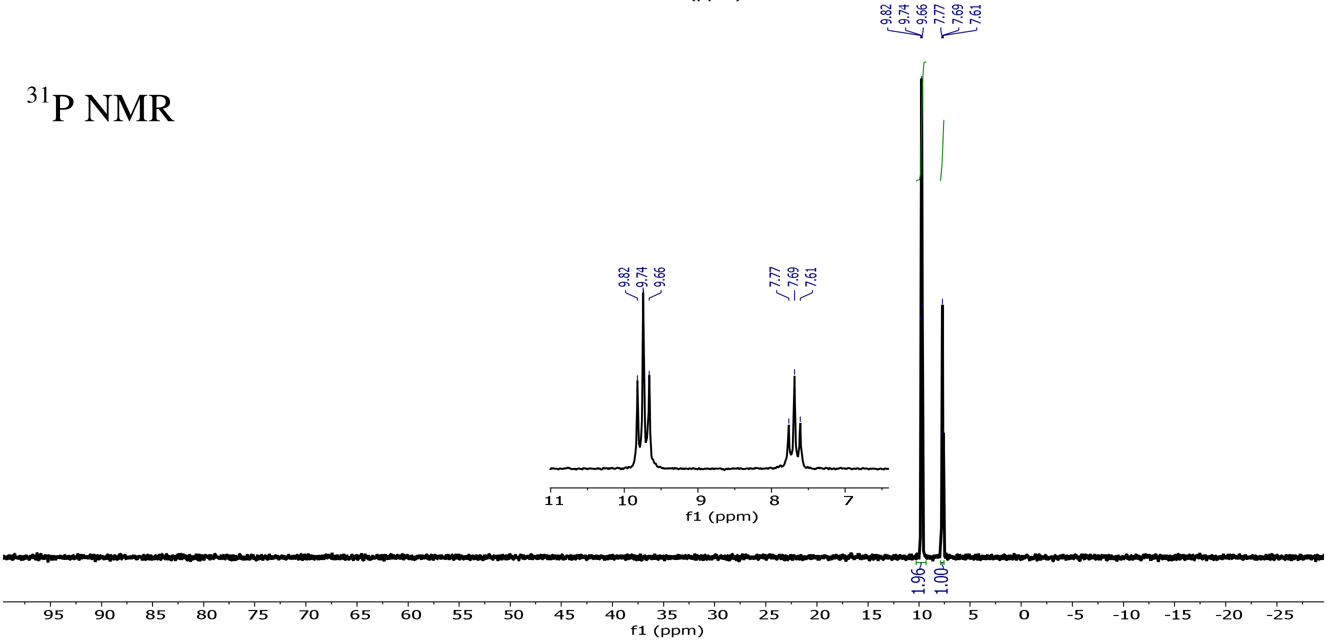

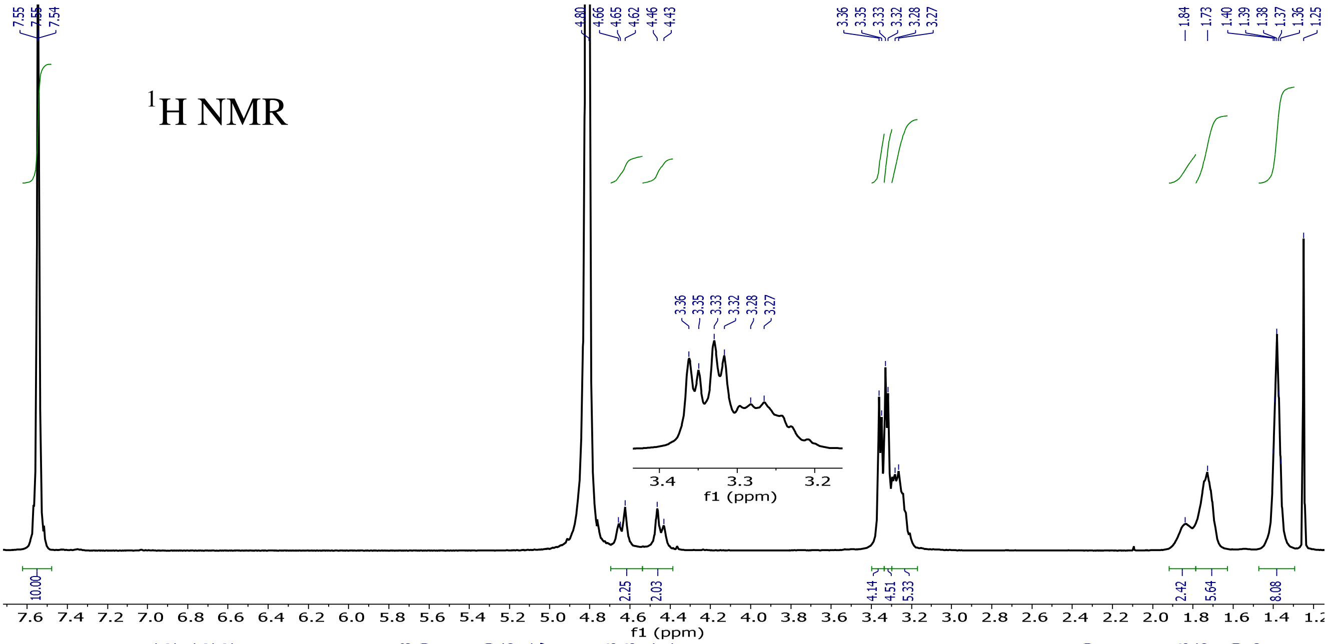

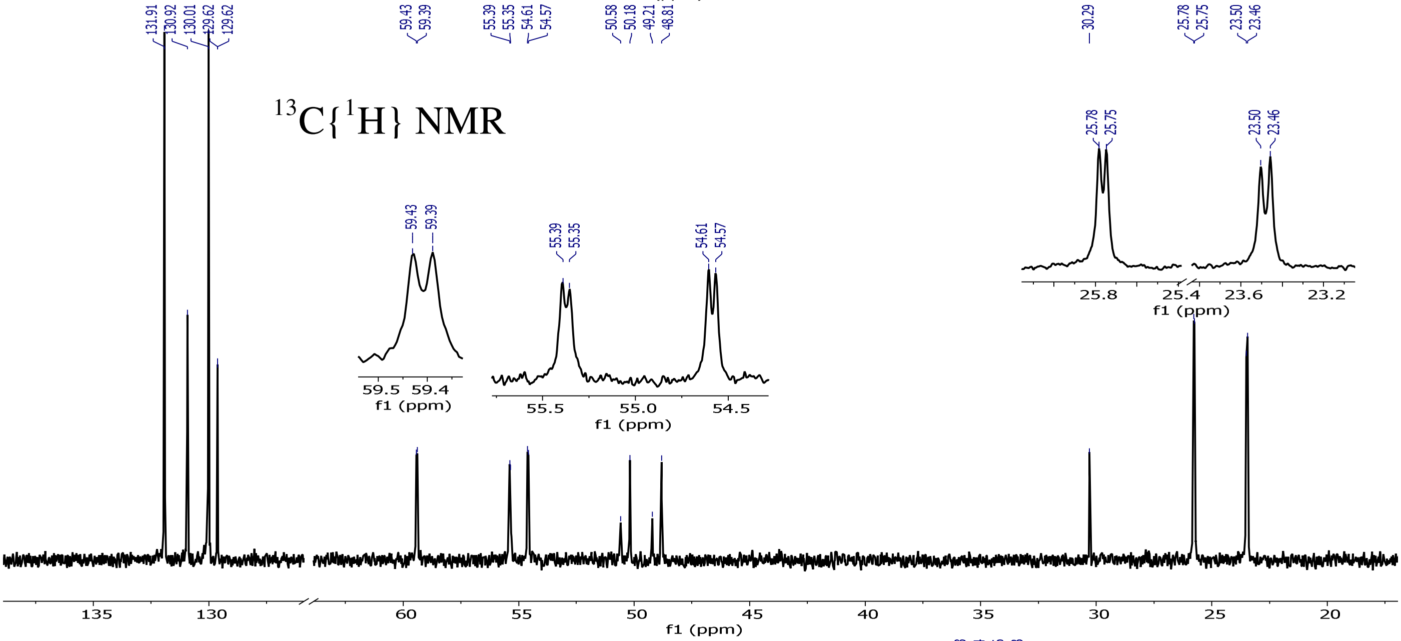

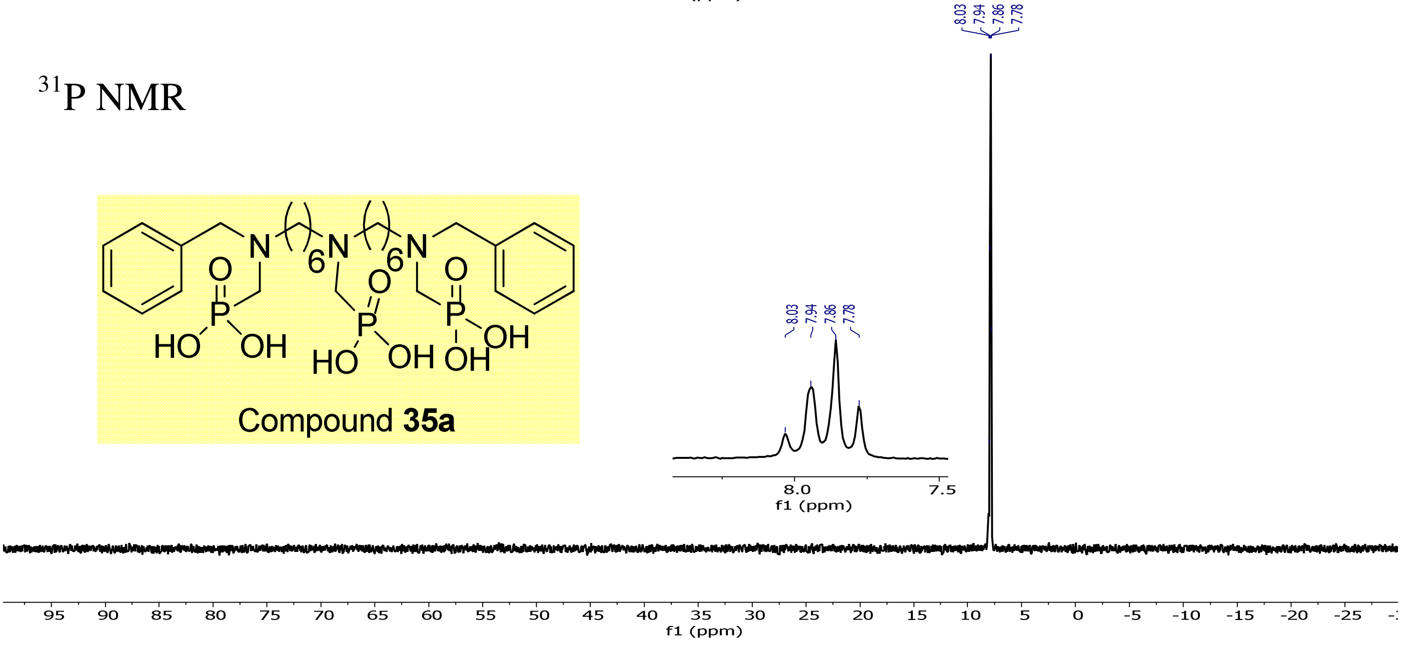

Compound **35a**

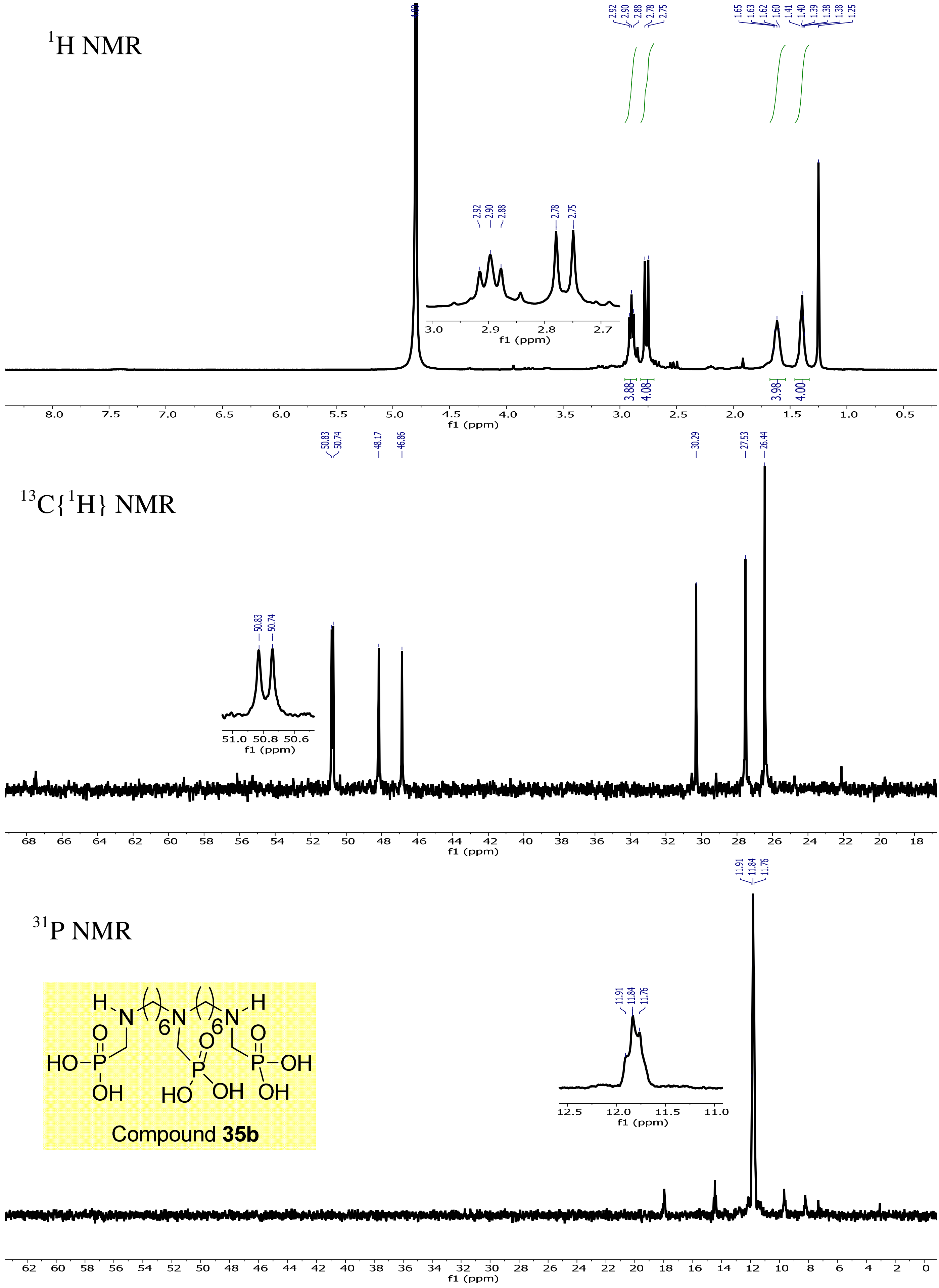
^{1}H NMR
2.92 2.90 2.88 2.78 2.75
1.65 1.63 1.62 1.60 1.41 1.40 1.39 1.38 1.38 1.25
3.88 4.08 3.98 4.00
f1 (ppm)
^{13}C{^{1}H} NMR
50.83 50.74 48.17 46.86 30.29 27.53 26.44
f1 (ppm)
^{31}P NMR
11.91 11.84 11.76
f1 (ppm)
Compound **35b**

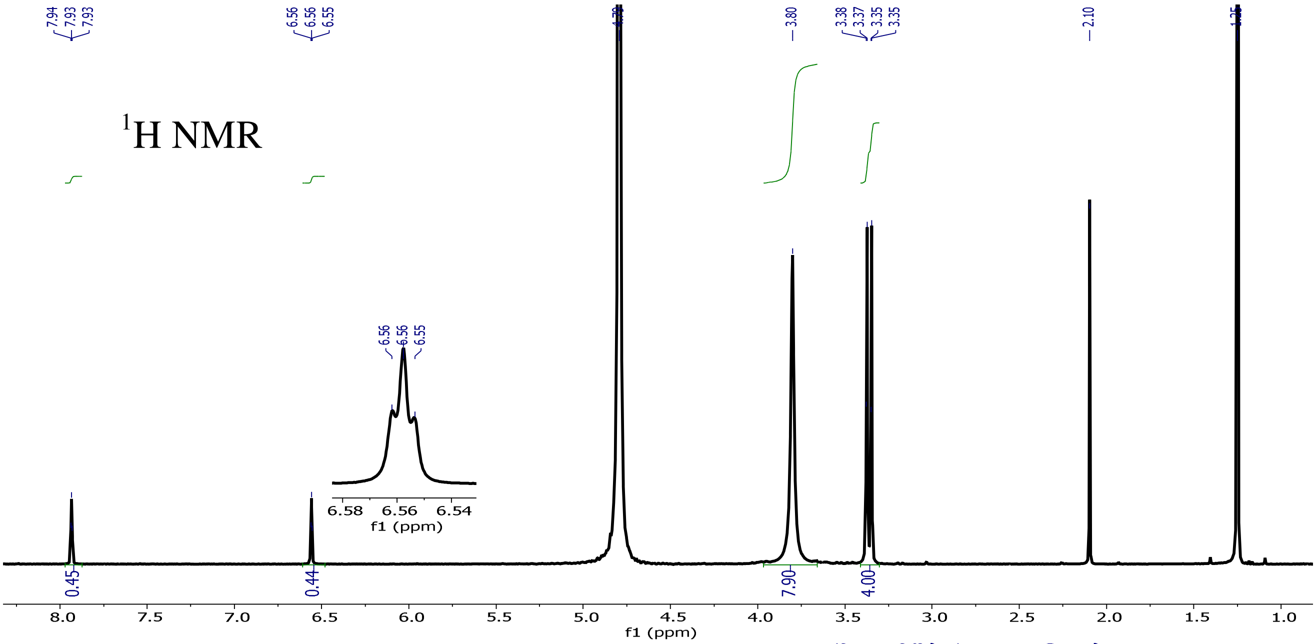
1H NMR
7.94 7.93 7.93
6.56 6.56 6.55
3.80
3.38 3.37 3.35 3.35
2.10
0.45
0.44
7.90
4.00
f1 (ppm)

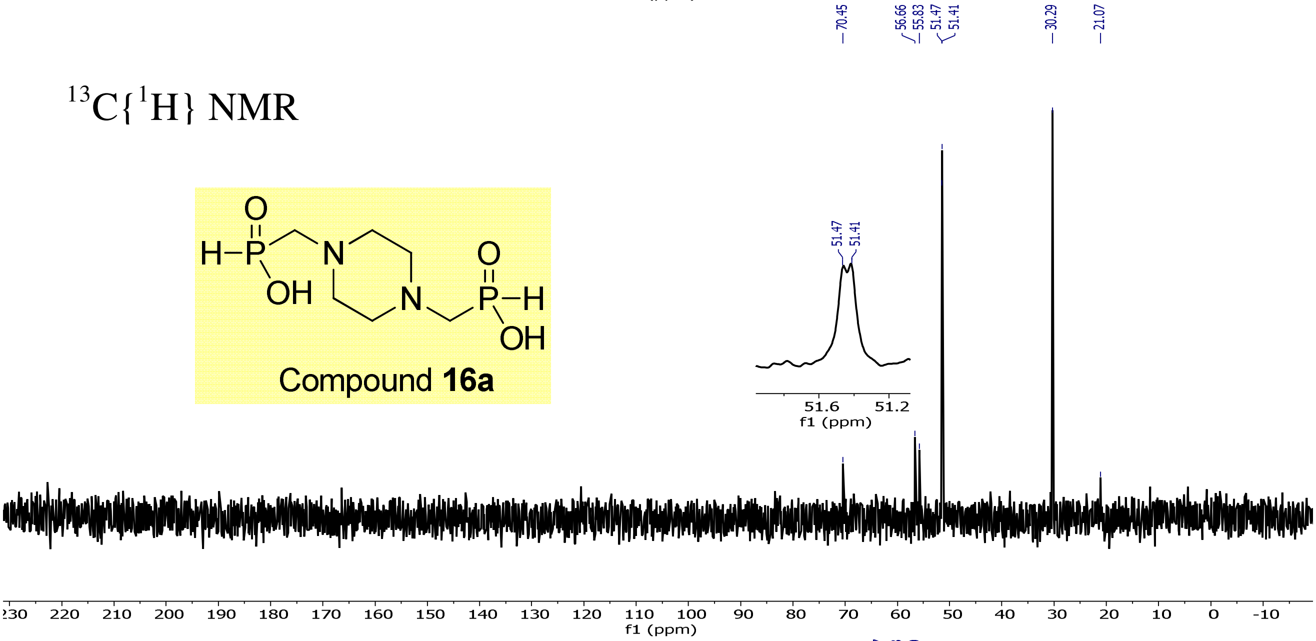
13C{1H} NMR
70.45
56.66 55.83 51.47 51.41
30.29
21.07
Compound 16a
f1 (ppm)

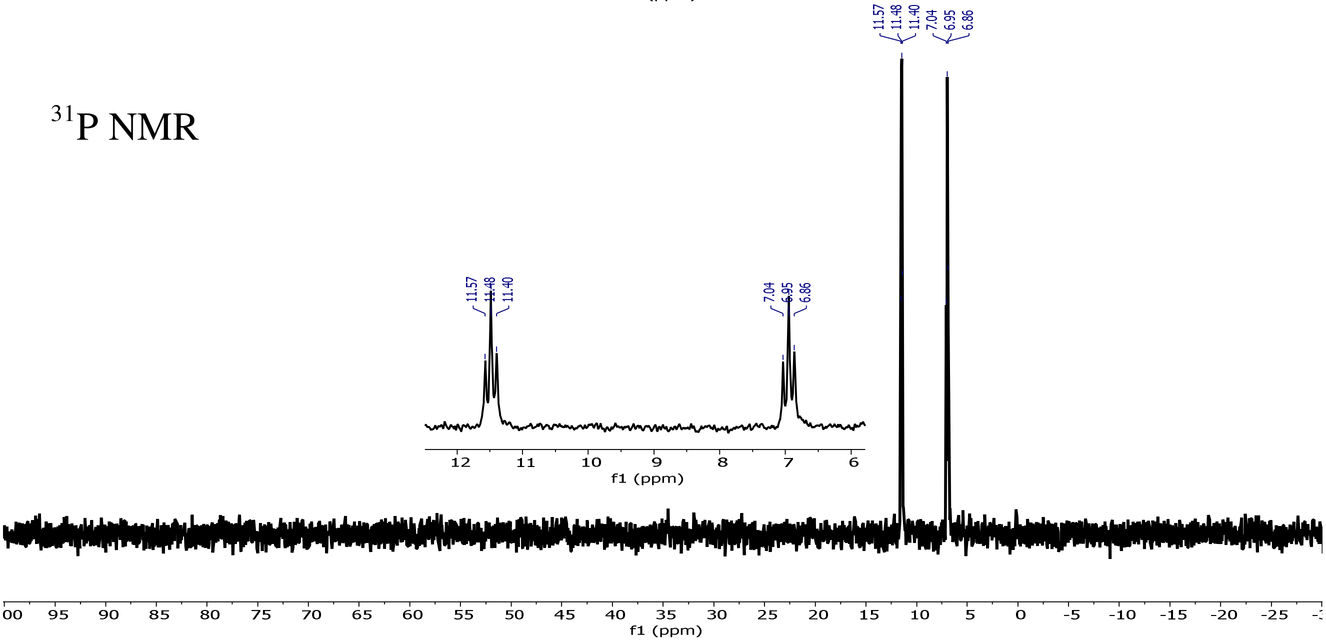
31P NMR
11.57 11.48 11.40 7.04 6.95 6.86
f1 (ppm)

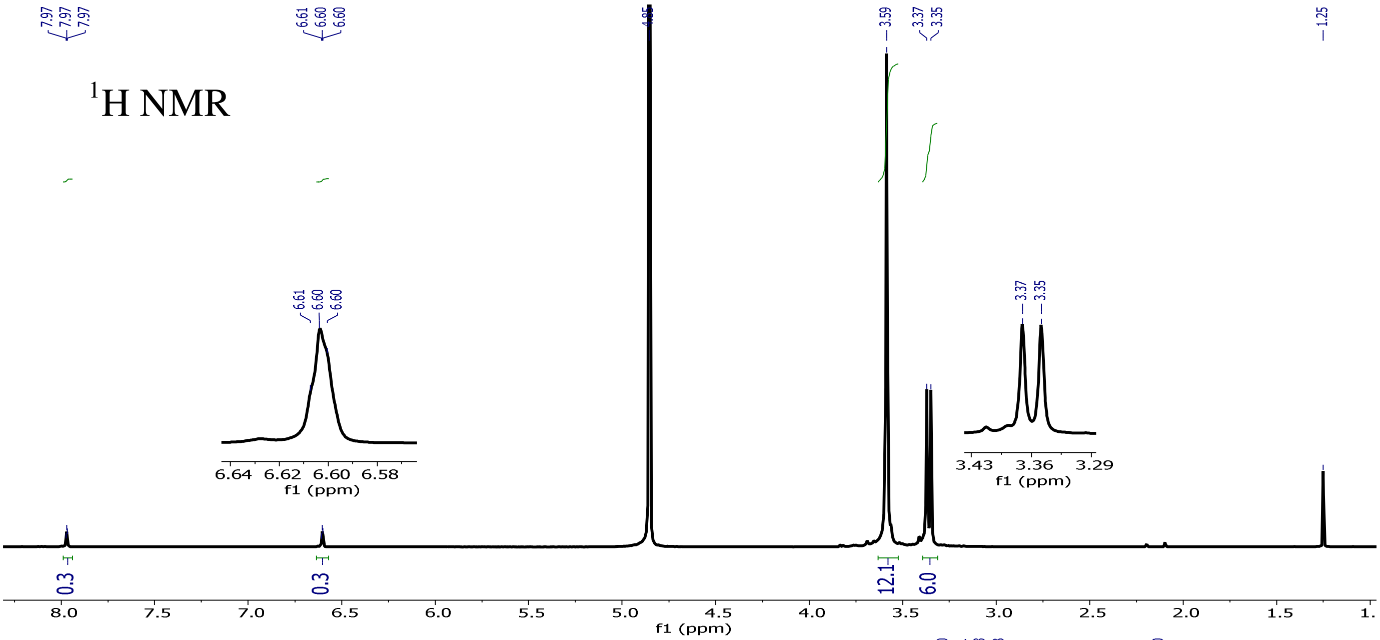

^{1}H NMR
f1 (ppm)

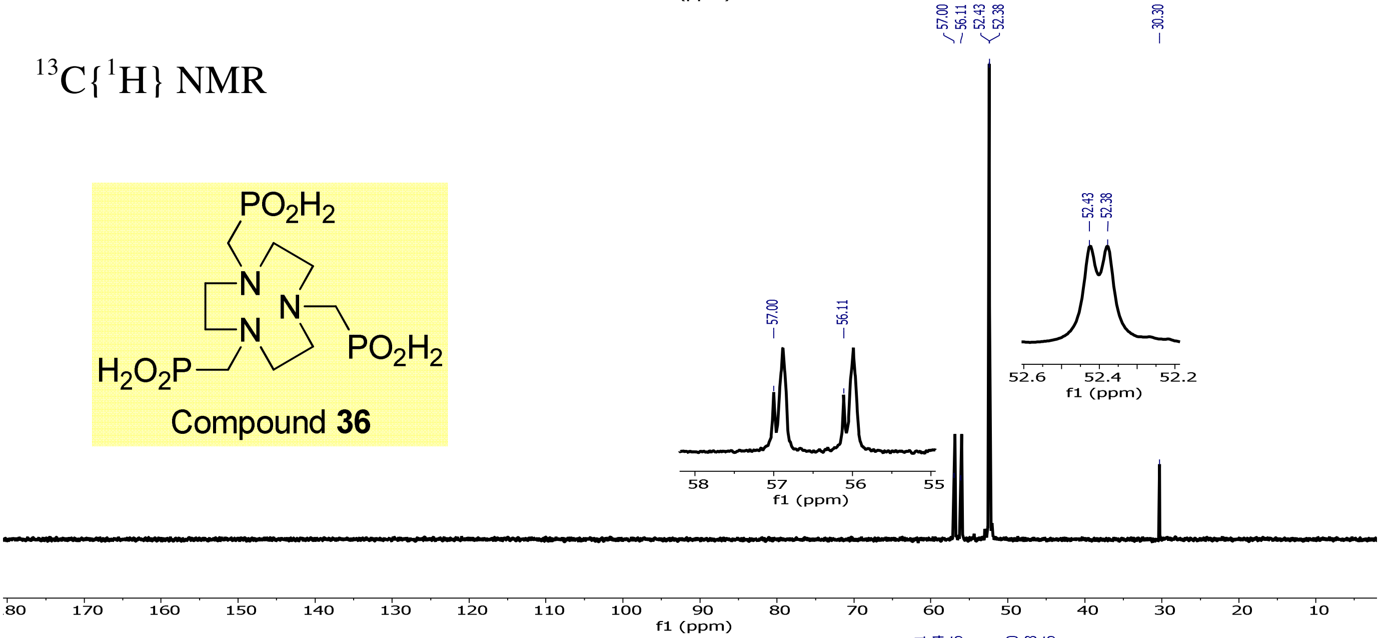

$^{13}C\{^{1}H\}$ NMR
PO_2H_2
H_2O_2P
Compound 36
f1 (ppm)

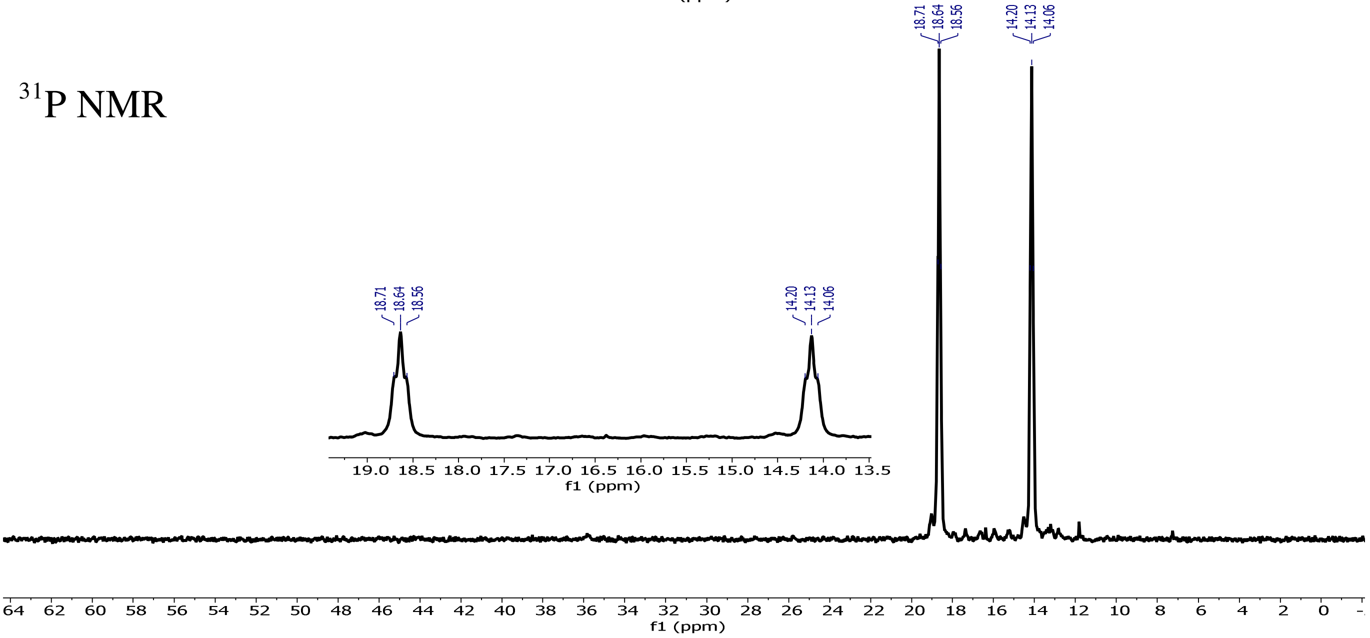

^{31}P NMR
f1 (ppm)

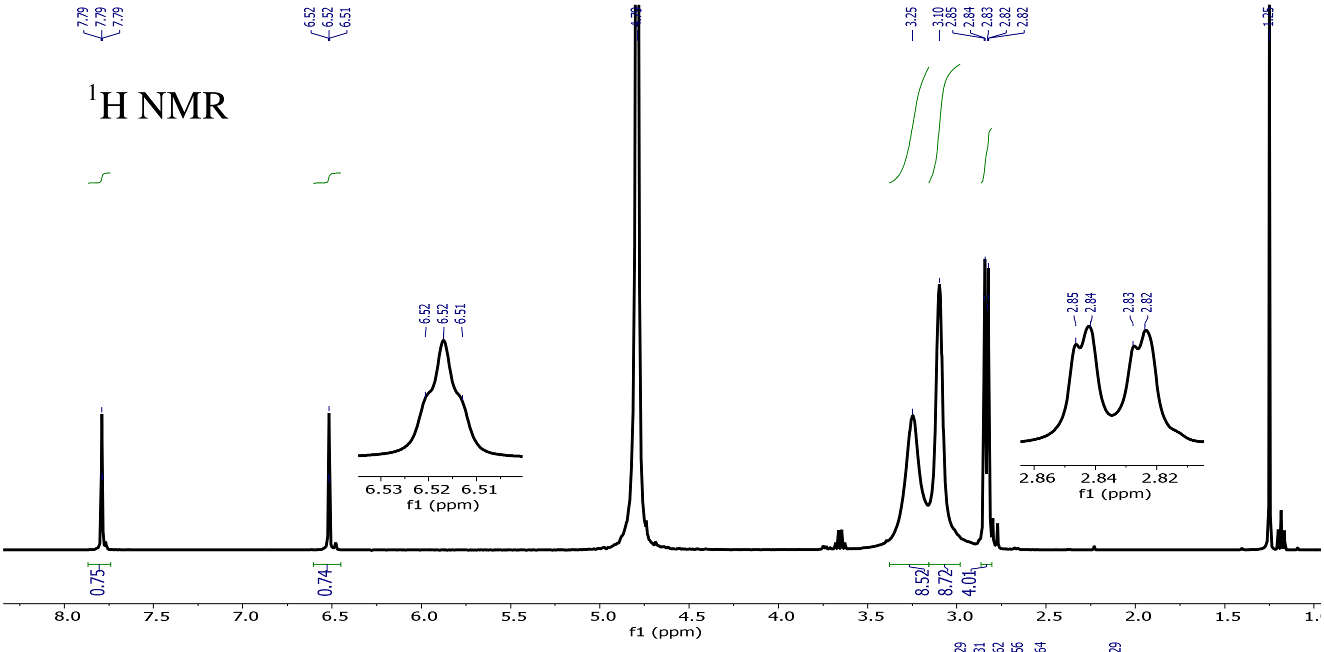

^{1}H NMR
7.79
7.79
7.79
6.52
6.52
6.51
3.25
3.10
2.85
2.84
2.83
2.82
2.82
1.25
0.75
0.74
8.52
8.72
4.01
6.53 6.52 6.51
2.86 2.84 2.82
f1 (ppm)

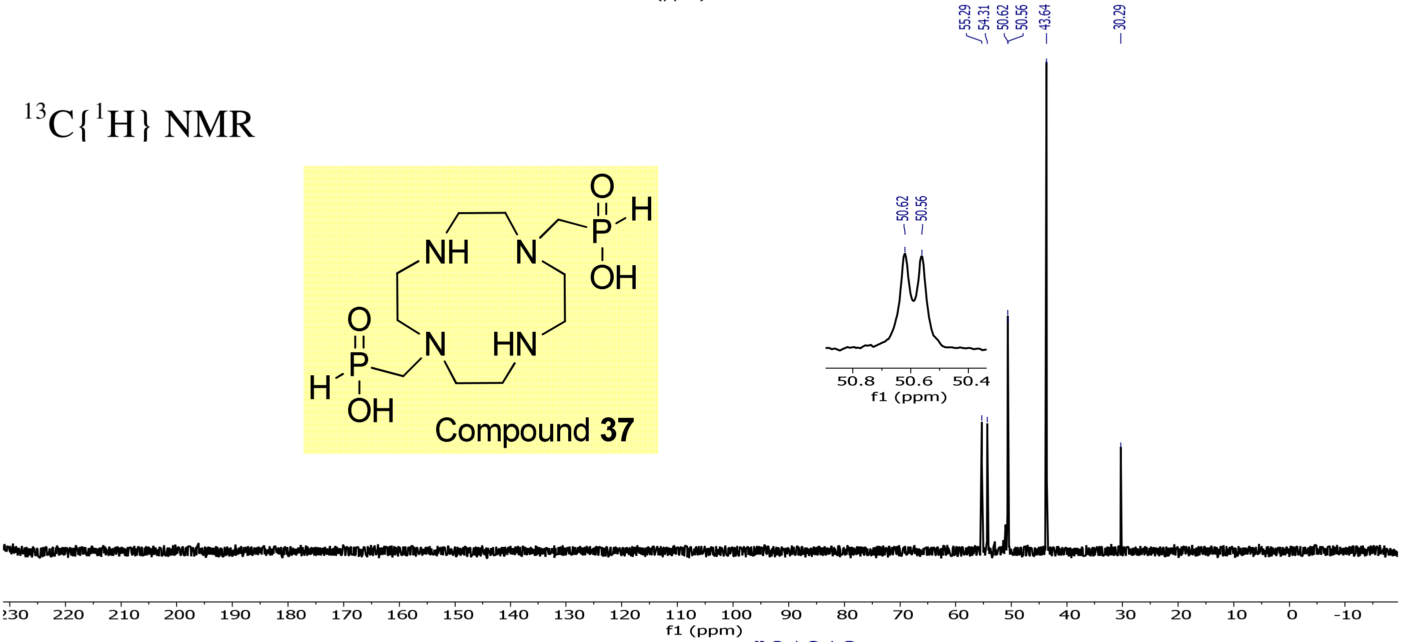

$^{13}C\{^{1}H\}$ NMR
55.29
54.31
50.62
50.56
43.64
30.29
50.8 50.6 50.4
f1 (ppm)
Compound **37**

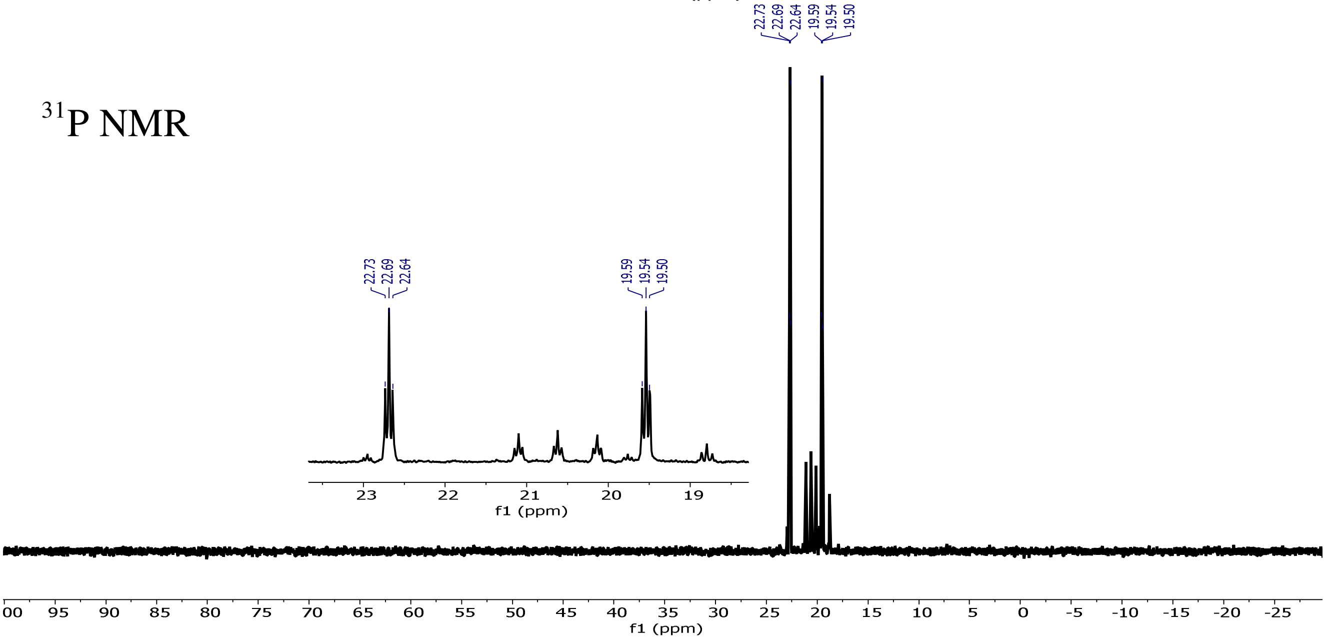

^{31}P NMR
22.73
22.69
22.64
19.59
19.54
19.50
23 22 21 20 19
f1 (ppm)

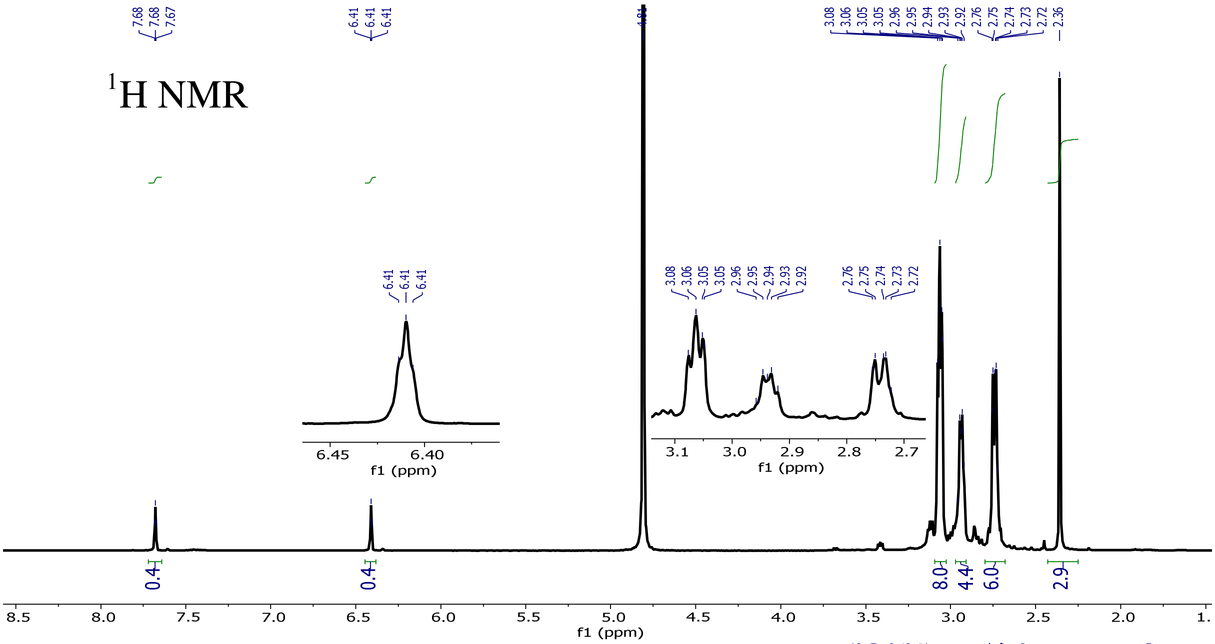
^{1}H NMR
f1 (ppm)

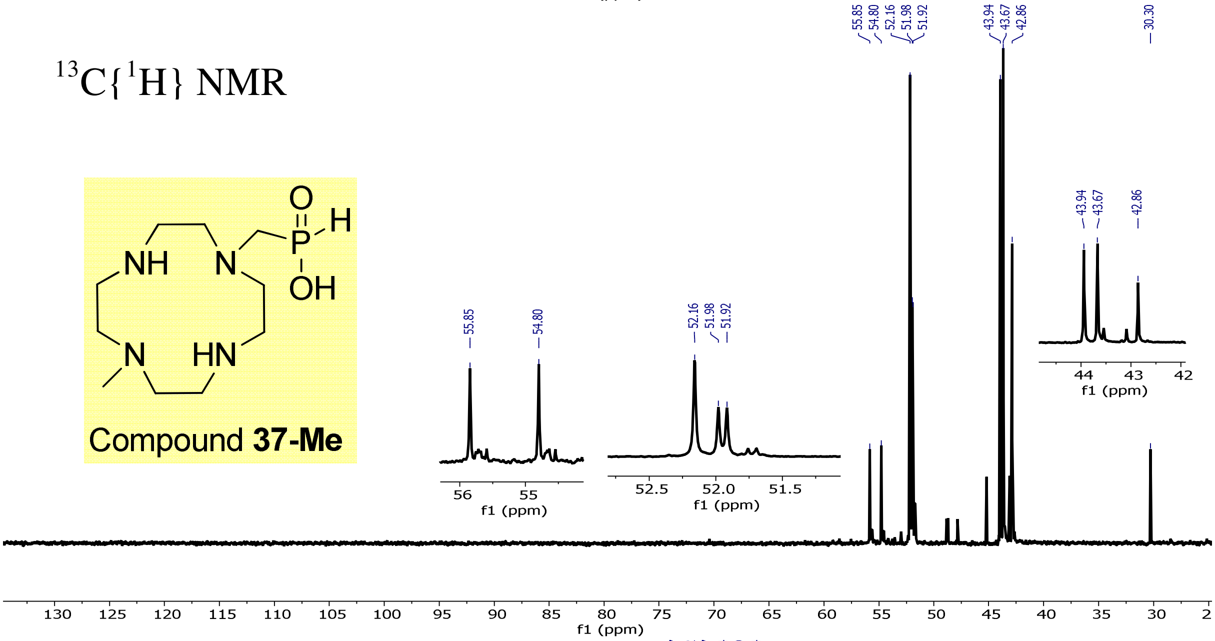
^{13}C{^{1}H} NMR
Compound **37-Me**
f1 (ppm)

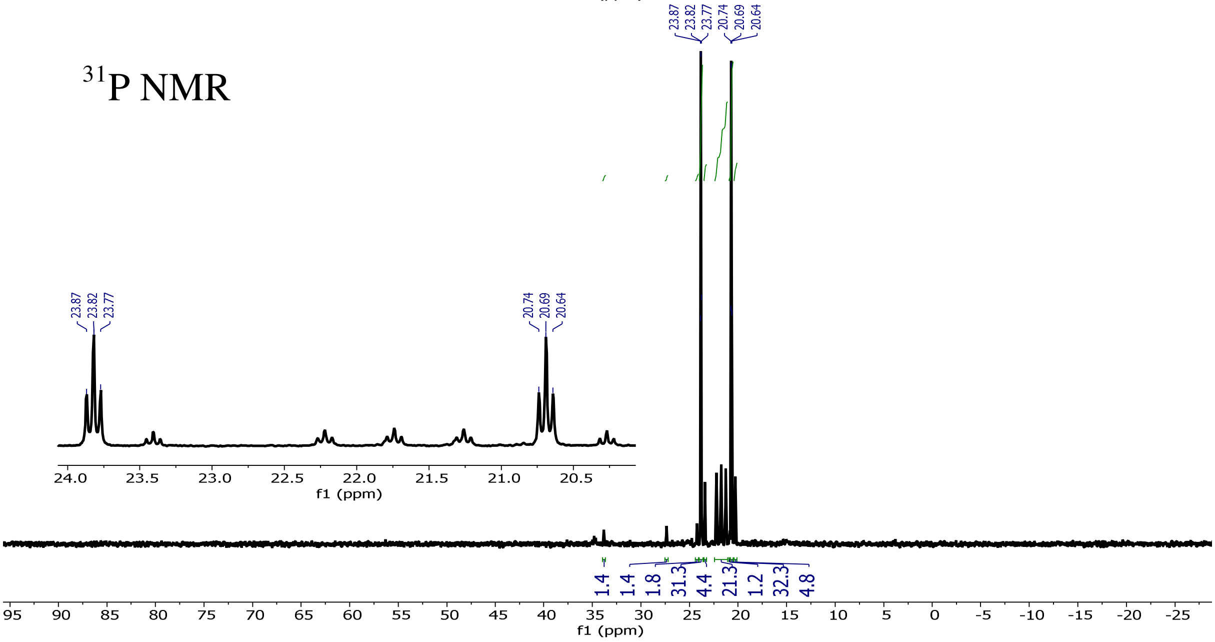
^{31}P NMR
f1 (ppm)

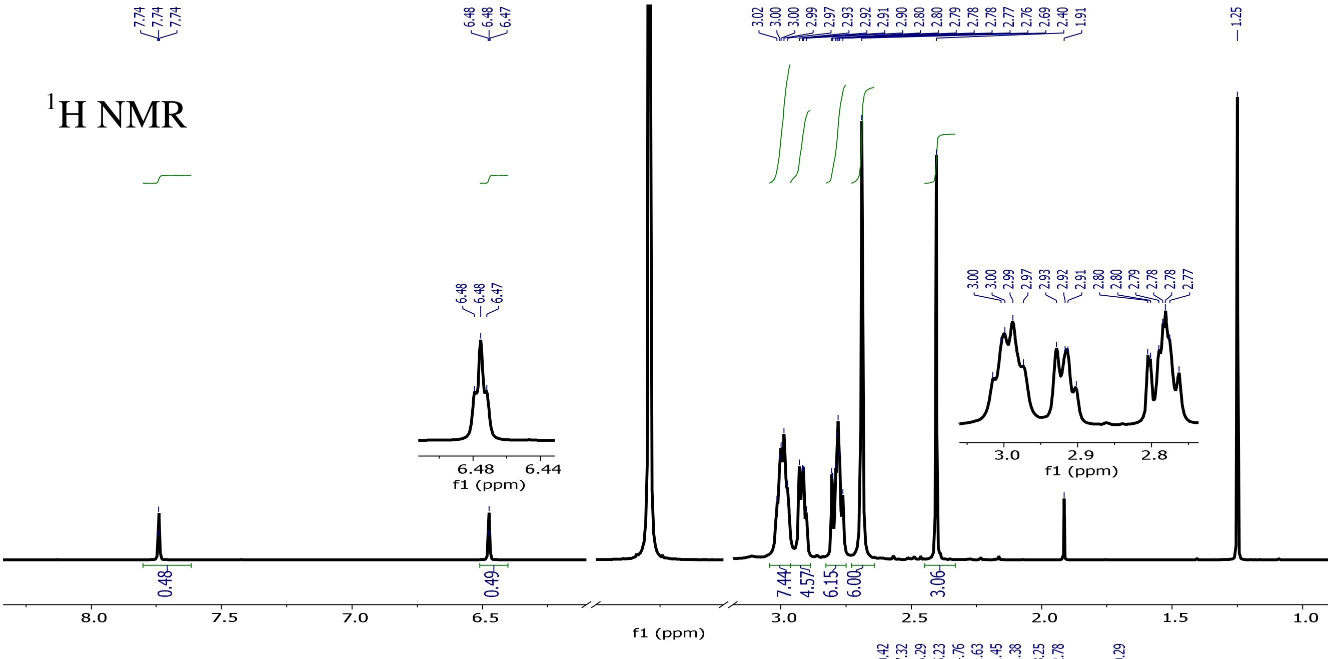

^{1}H NMR
f1 (ppm)

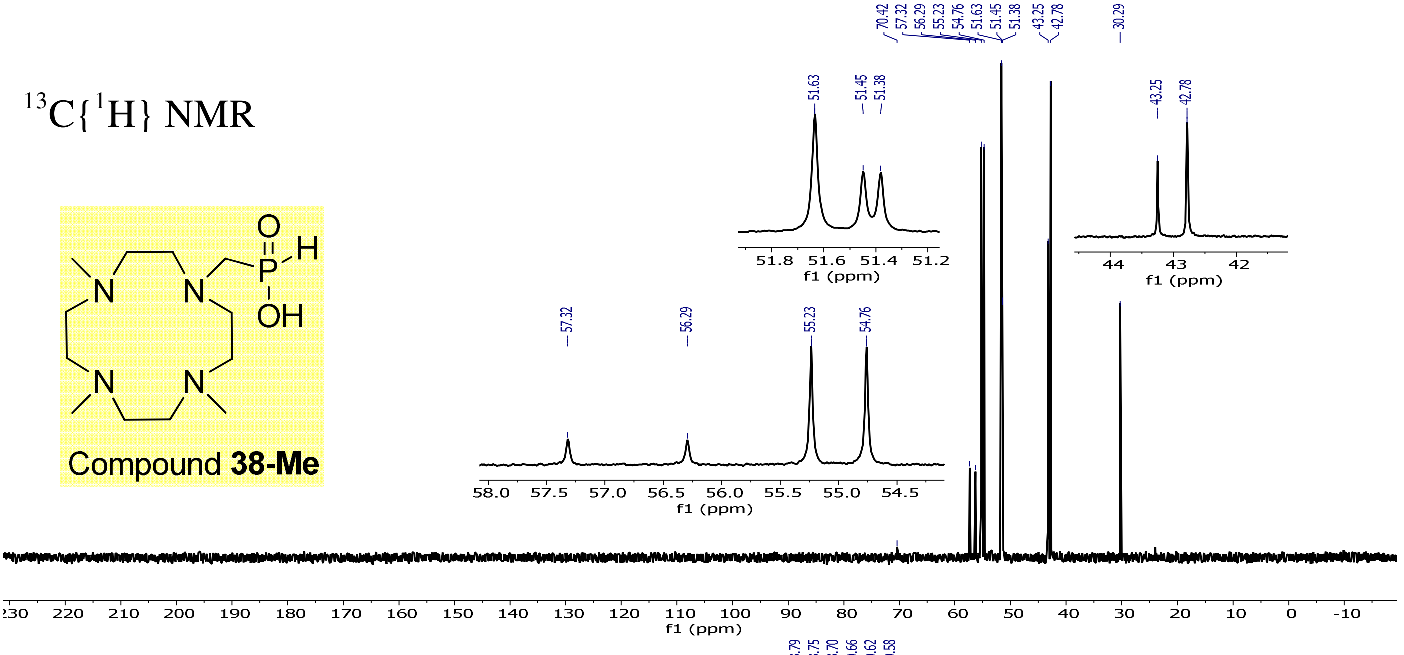

^{13}C{^{1}H} NMR
Compound 38-Me
f1 (ppm)

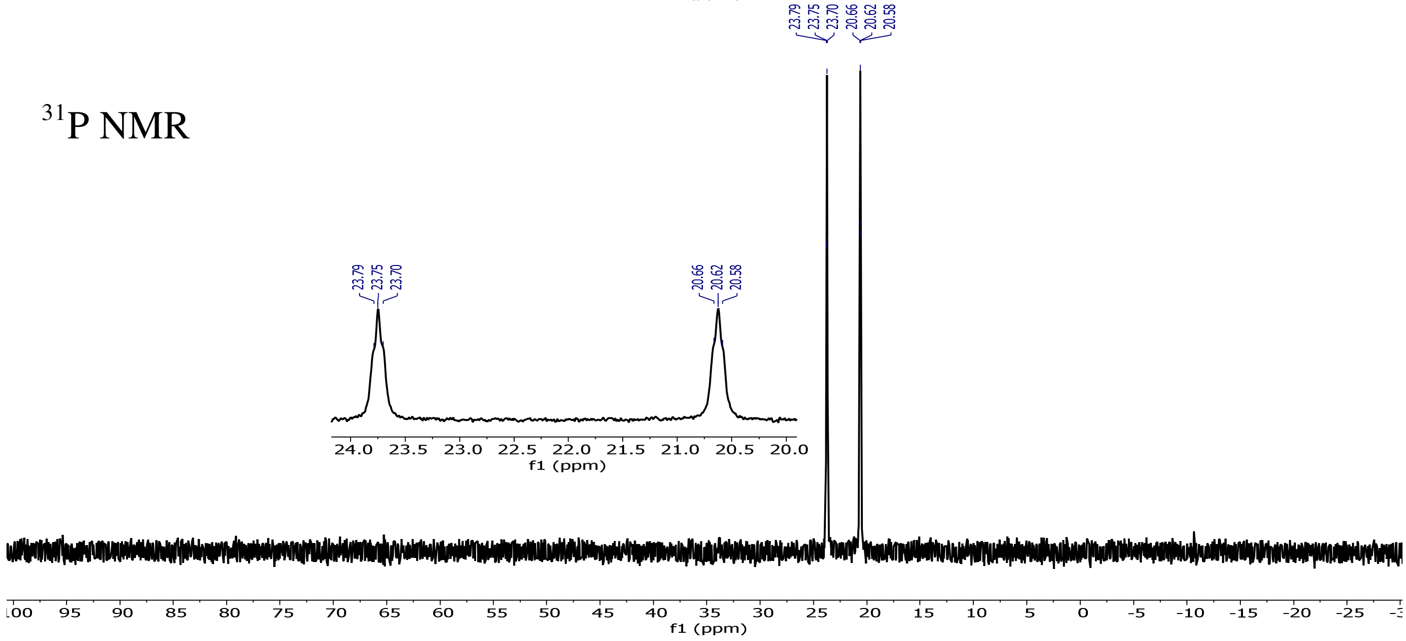

^{31}P NMR
f1 (ppm)

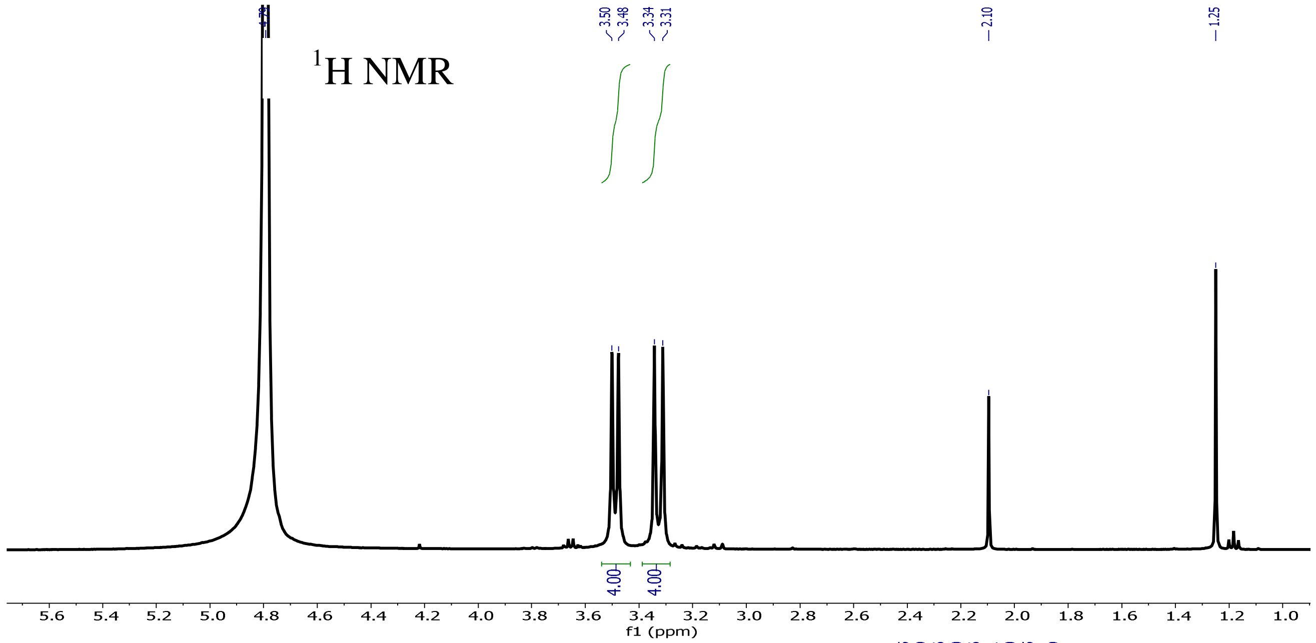
1H NMR
f1 (ppm)

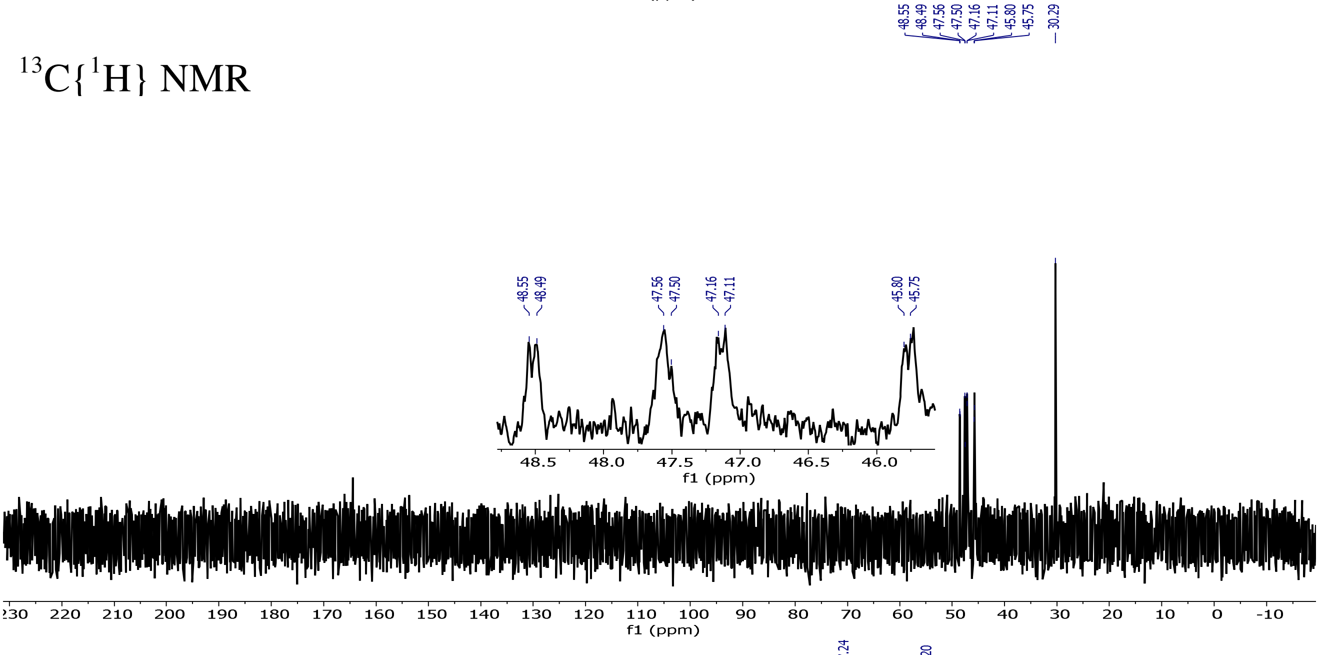
13C{1H} NMR
f1 (ppm)

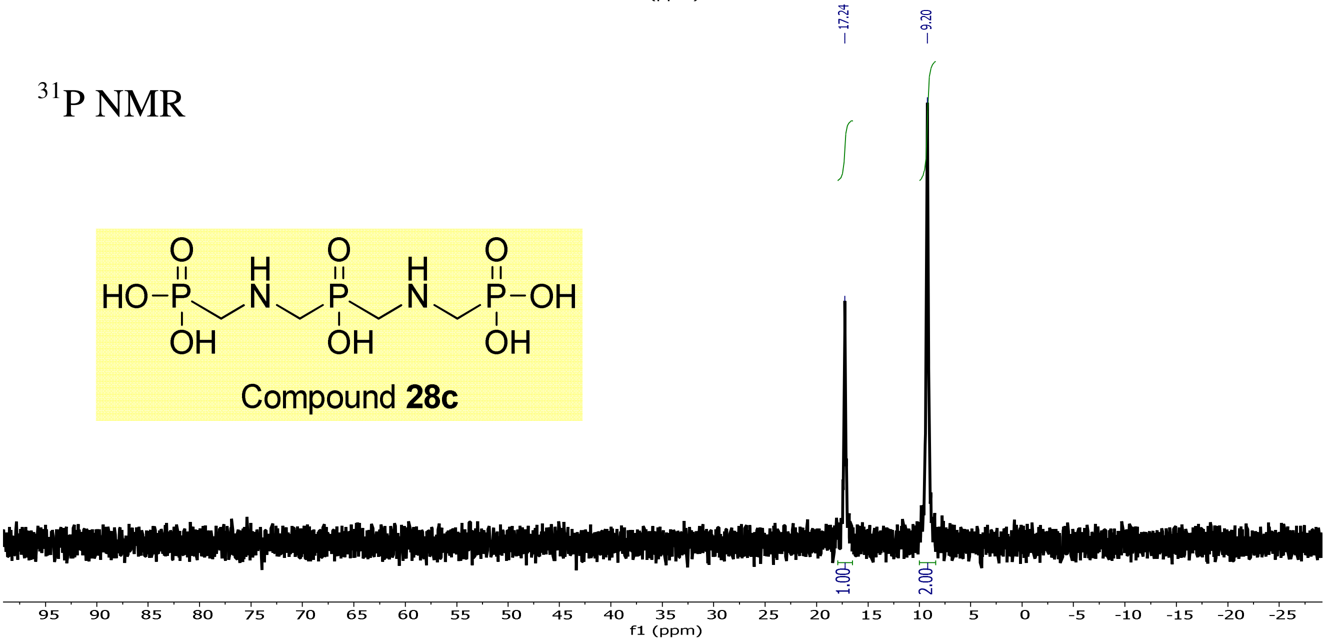
31P NMR
Compound 28c
f1 (ppm)

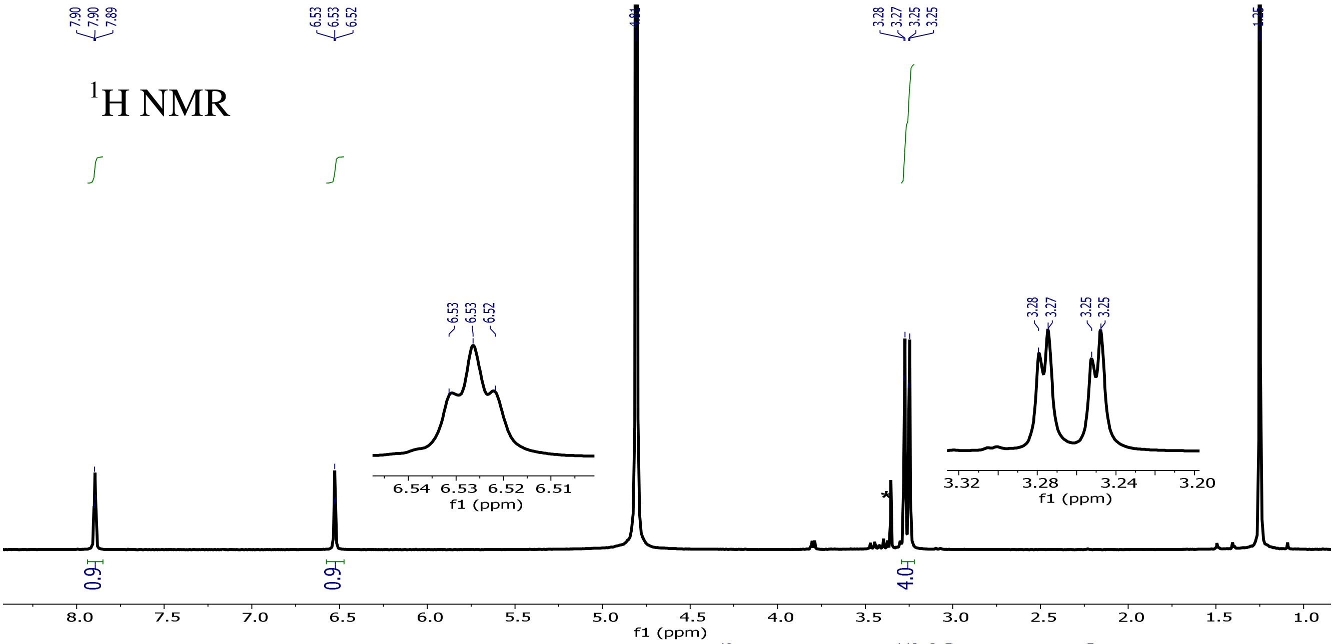

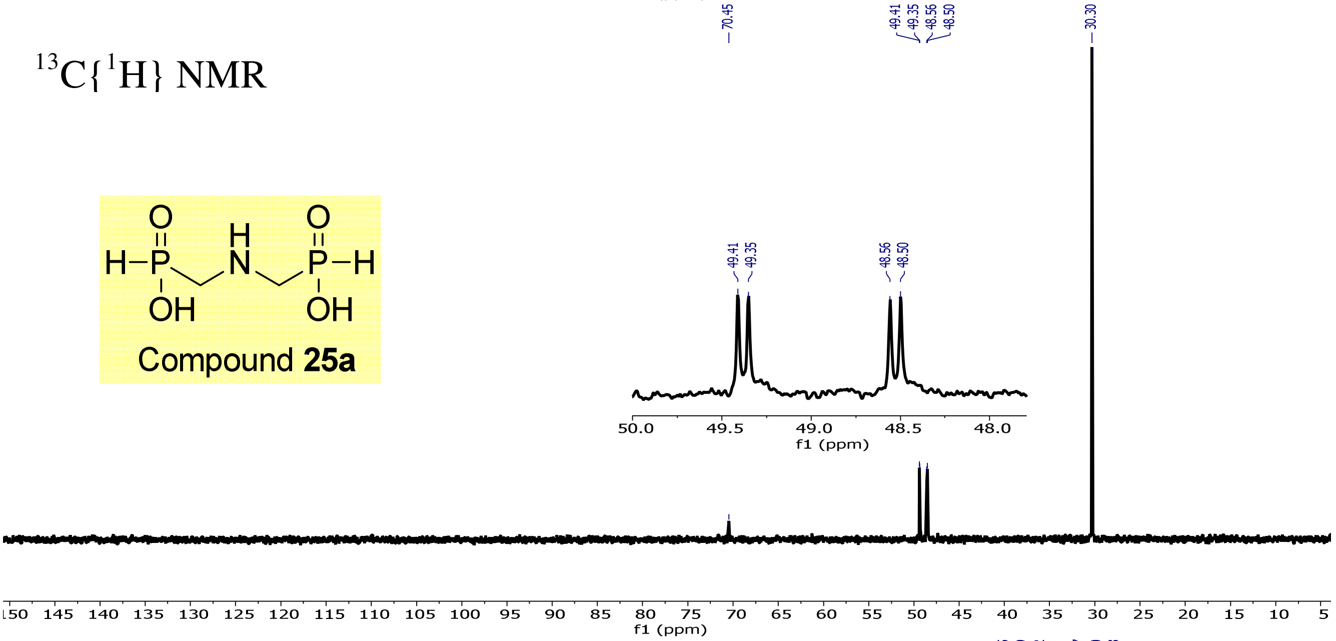

Compound **25a**

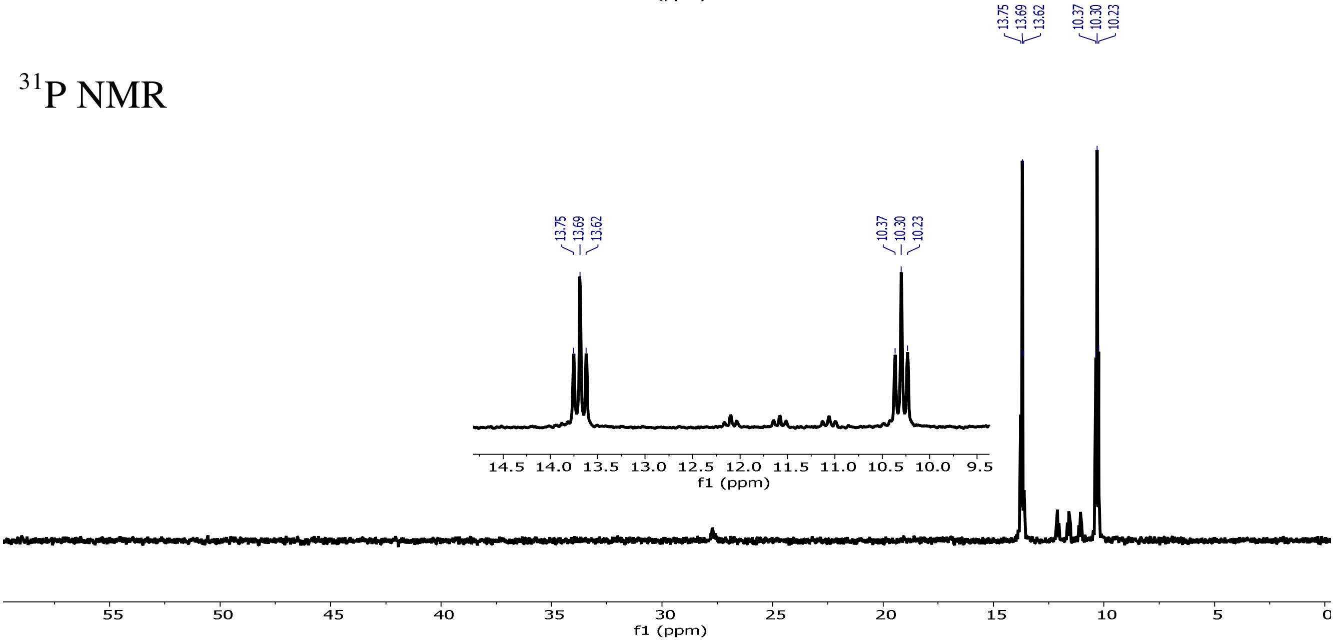

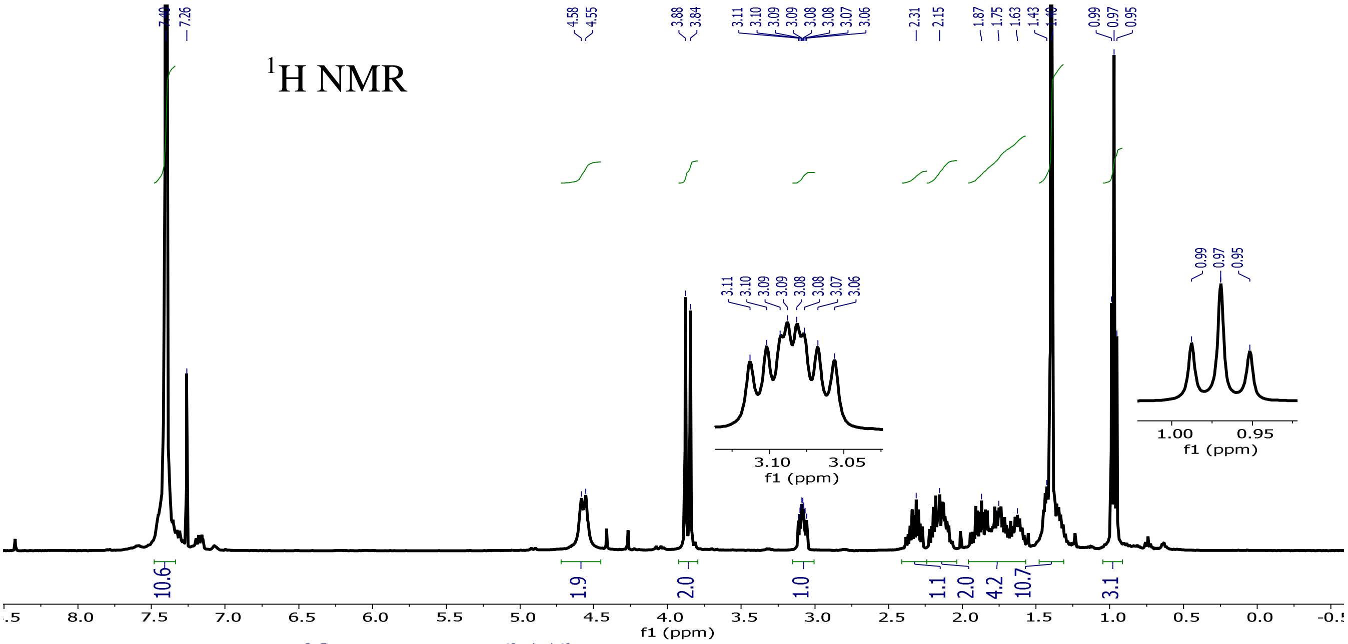
1H NMR
f1 (ppm)

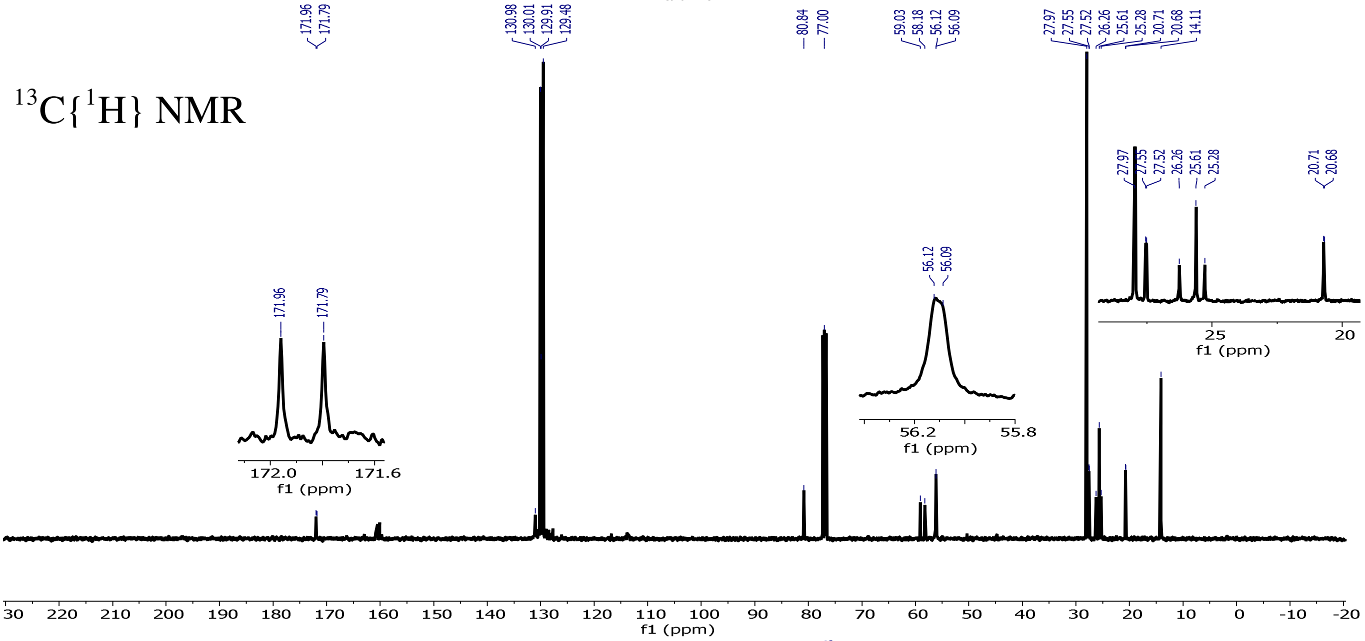
13C{1H} NMR
f1 (ppm)

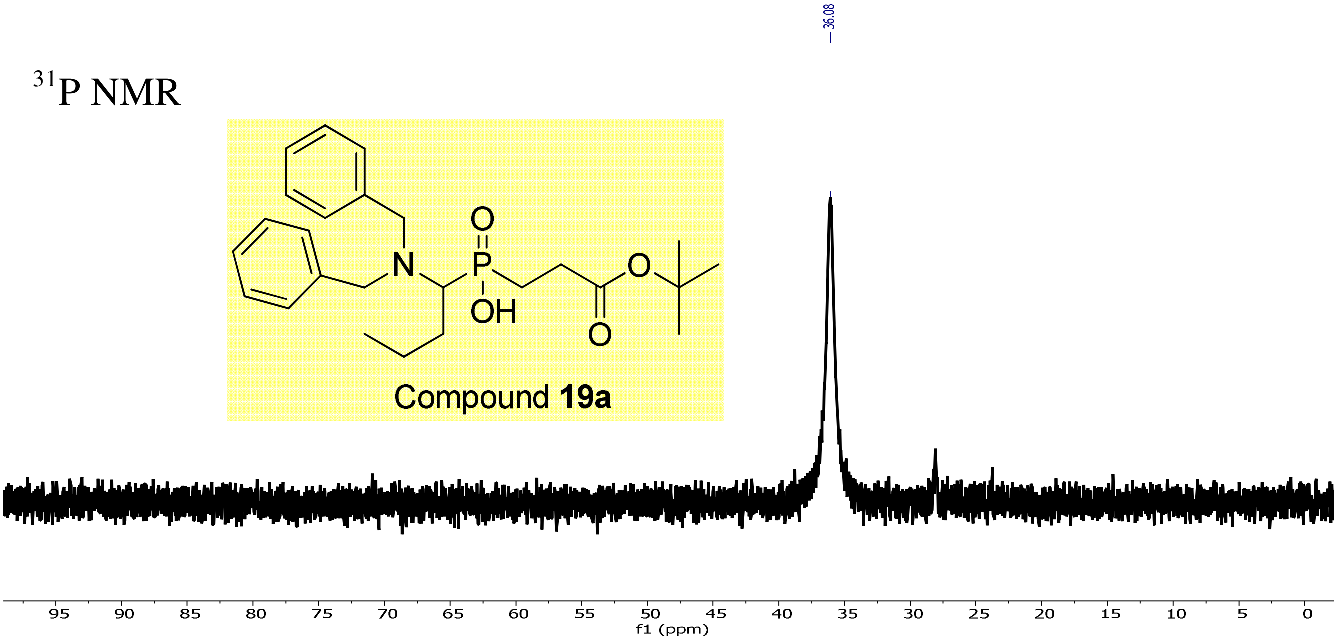
31P NMR
Compound 19a
f1 (ppm)

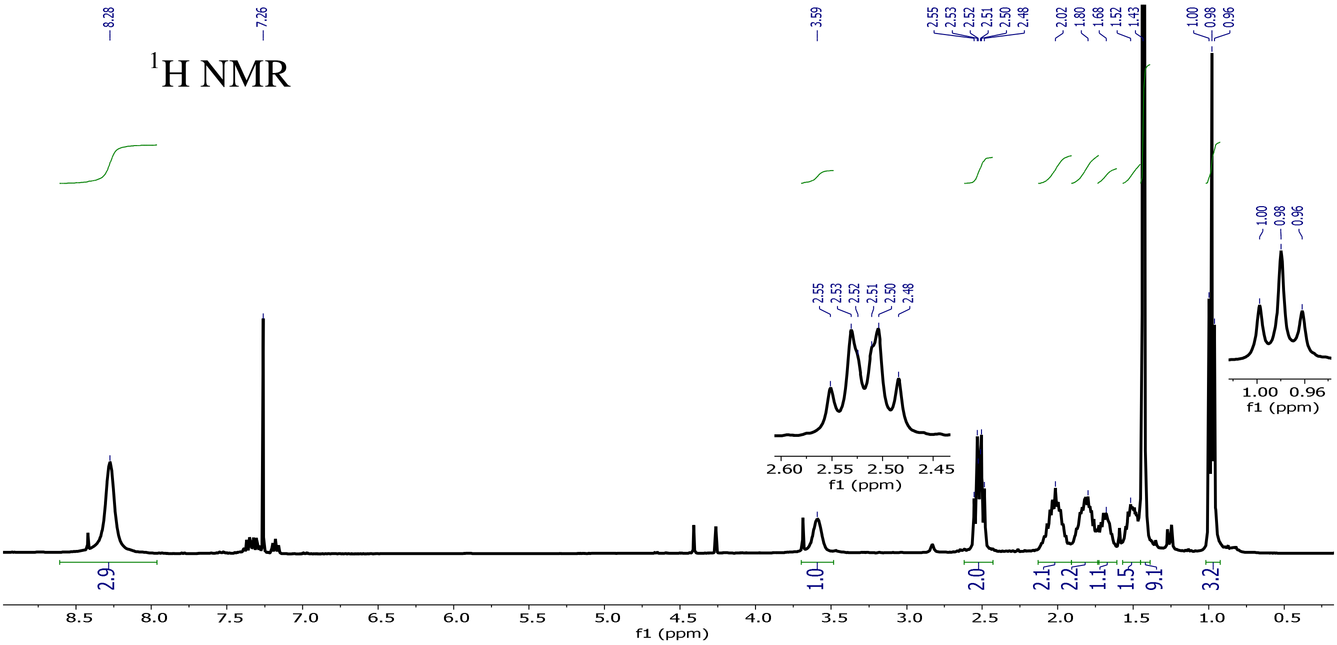

8.28
7.26
3.59
2.55 2.53 2.52 2.51 2.50 2.48
2.02 1.80 1.68 1.52 1.43
1.00 0.98 0.96
^{1}H NMR
2.60 2.55 2.50 2.45
f1 (ppm)
1.00 0.96
f1 (ppm)
2.9
1.0
2.0
2.1 2.2 1.1 1.5 9.1
3.2
8.5 8.0 7.5 7.0 6.5 6.0 5.5 5.0 4.5 4.0 3.5 3.0 2.5 2.0 1.5 1.0 0.5
f1 (ppm)

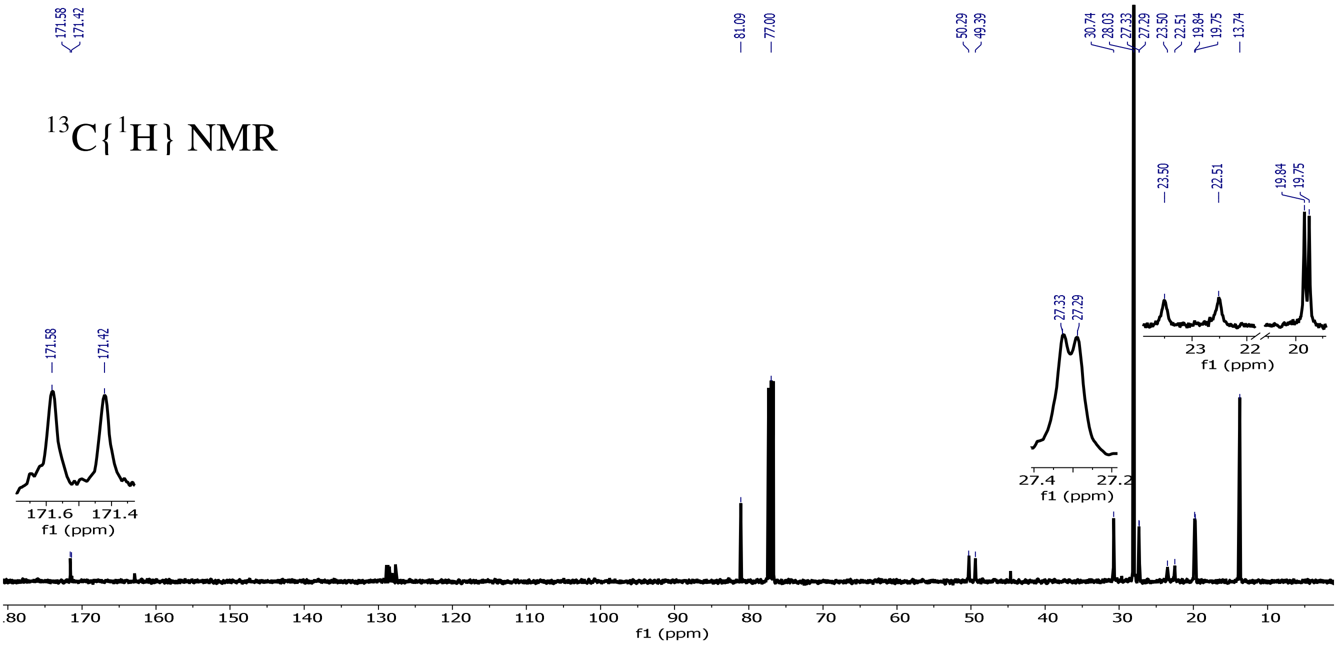

171.58 171.42
81.09 77.00
50.29 49.39
30.74 28.03 27.33 27.29 23.50 22.51 19.84 19.75 13.74
^{13}C{^{1}H} NMR
23.50 22.51 19.84 19.75
23 22 20
f1 (ppm)
27.33 27.29
27.4 27.2
f1 (ppm)
171.58 171.42
171.6 171.4
f1 (ppm)
180 170 160 150 140 130 120 110 100 90 80 70 60 50 40 30 20 10
f1 (ppm)

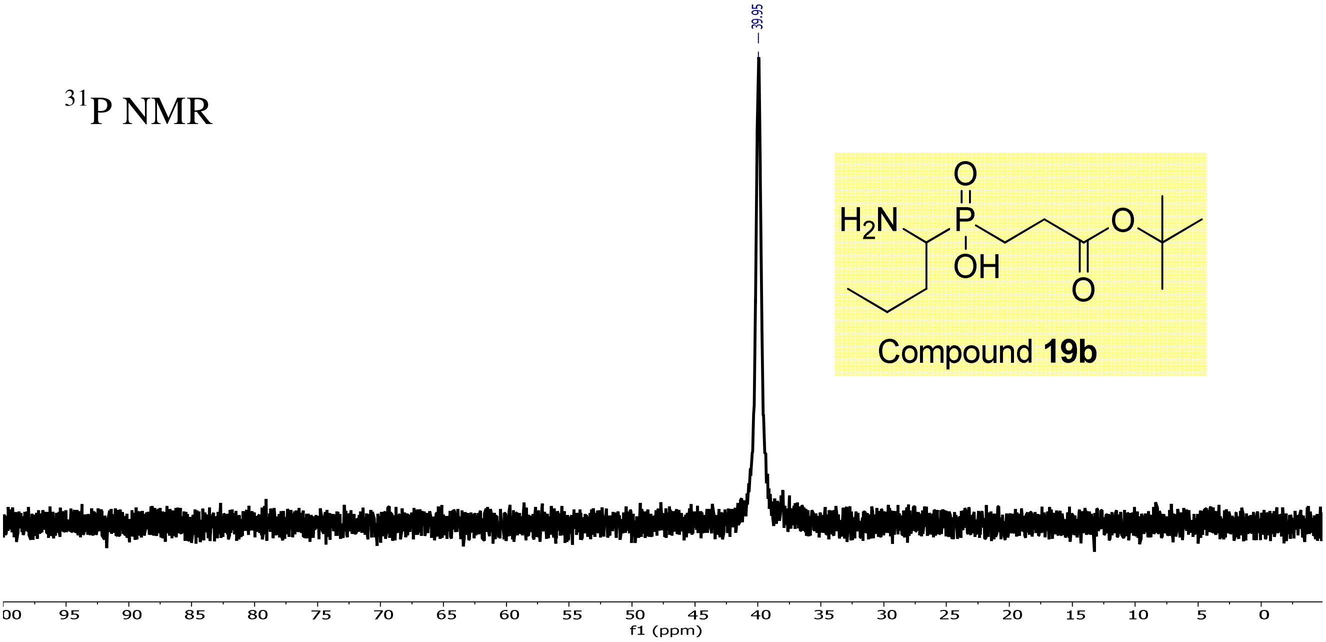

39.95
^{31}P NMR
H_2N
O
P
OH
O
O
Compound **19b**
100 95 90 85 80 75 70 65 60 55 50 45 40 35 30 25 20 15 10 5 0
f1 (ppm)

^{1}H NMR

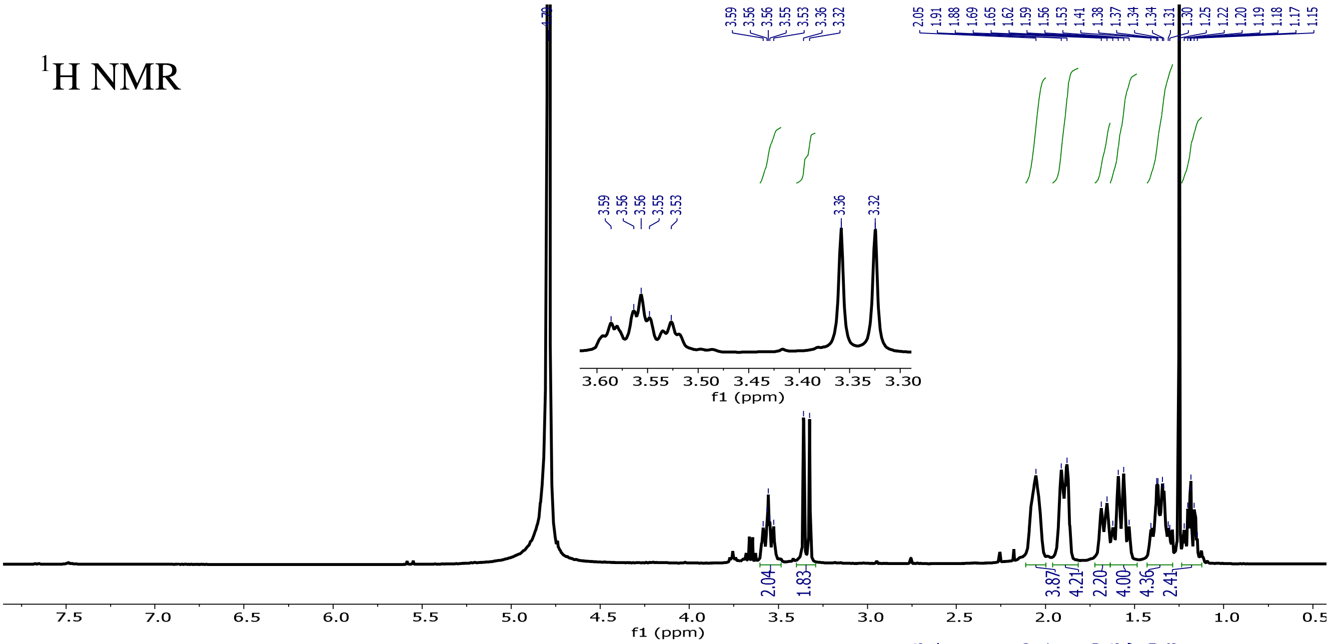

^{13}C{^{1}H} NMR

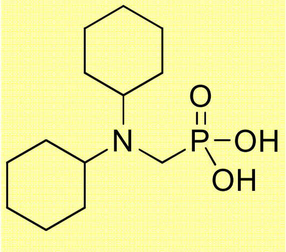

Compound **B**

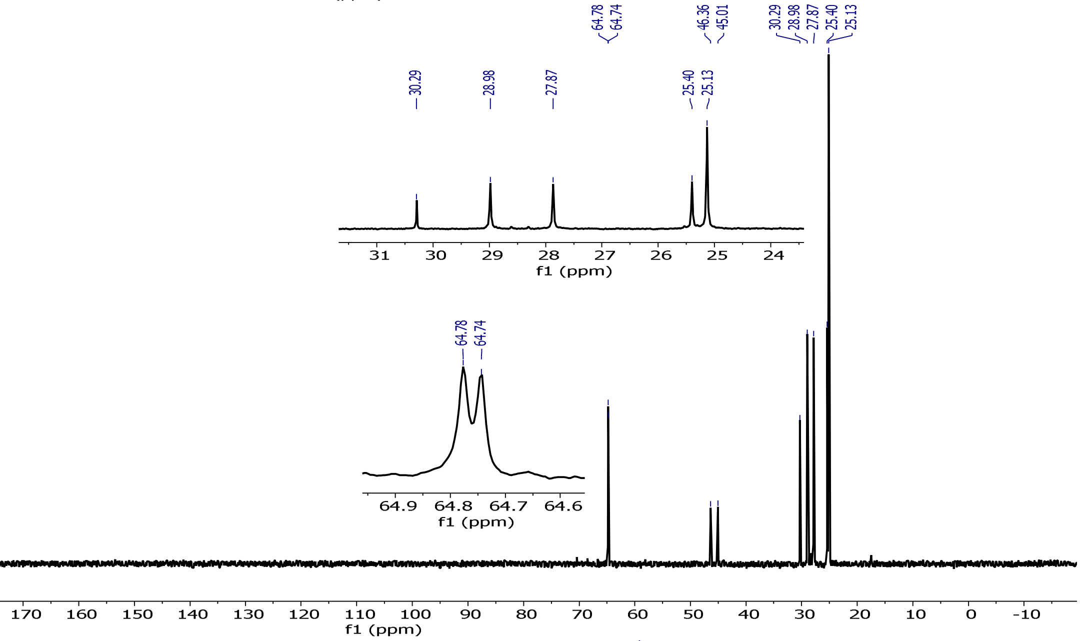

^{31}P NMR

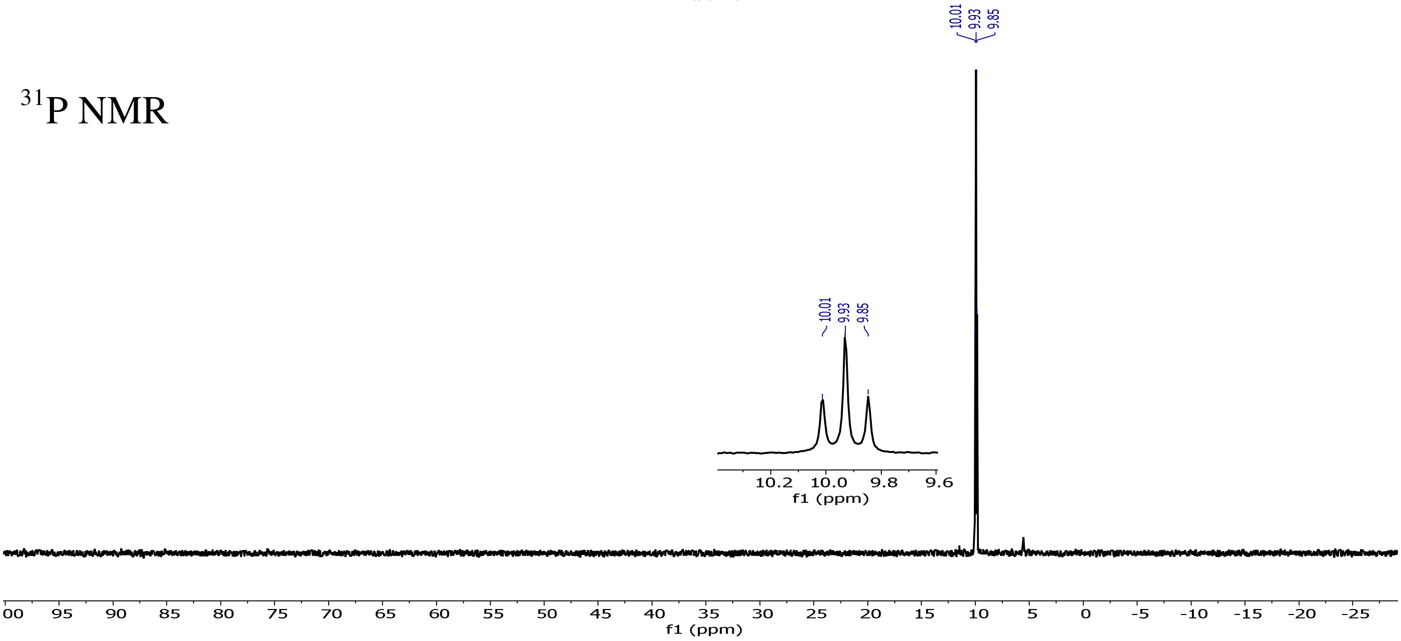

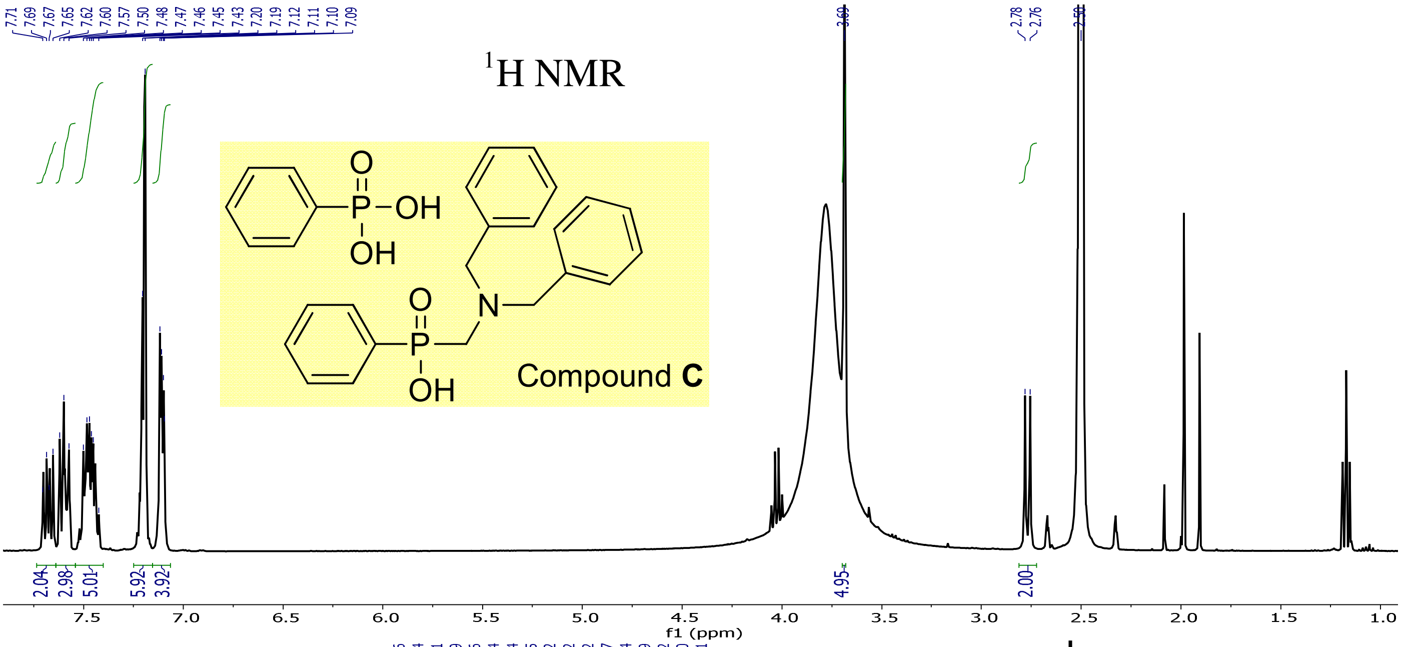
1H NMR
O
P
OH
OH
N
Compound C
f1 (ppm)

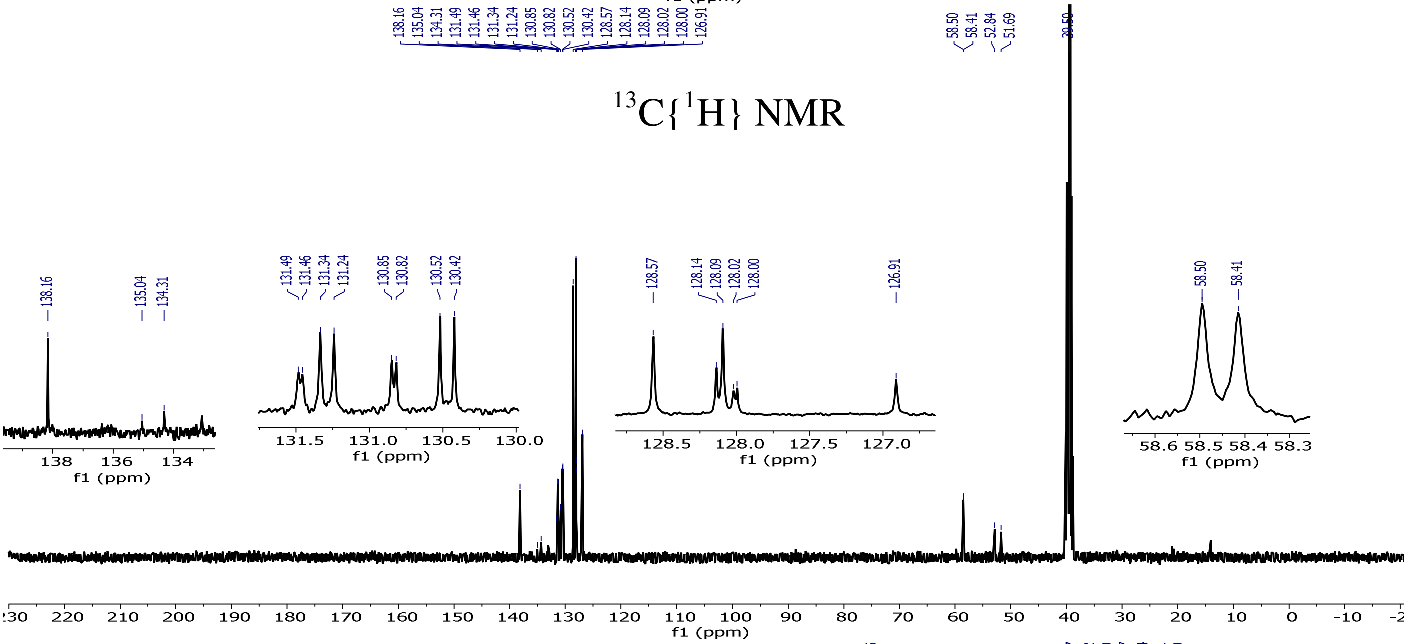
13C{1H} NMR
f1 (ppm)

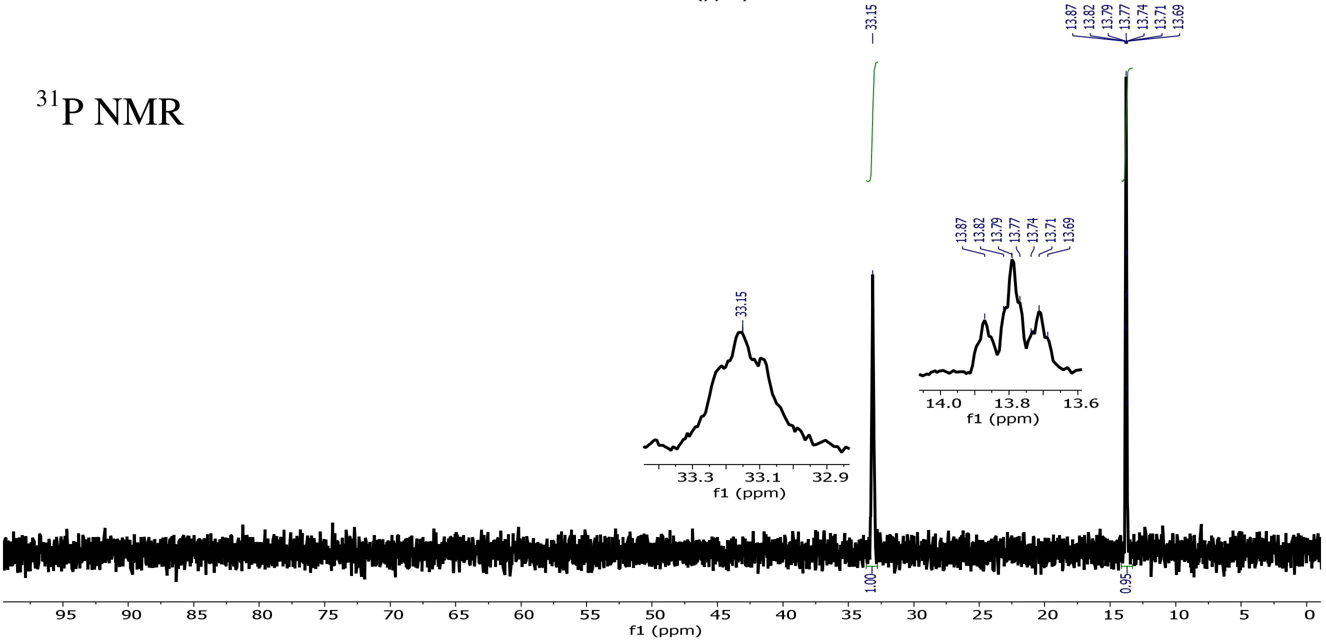
31P NMR
f1 (ppm)

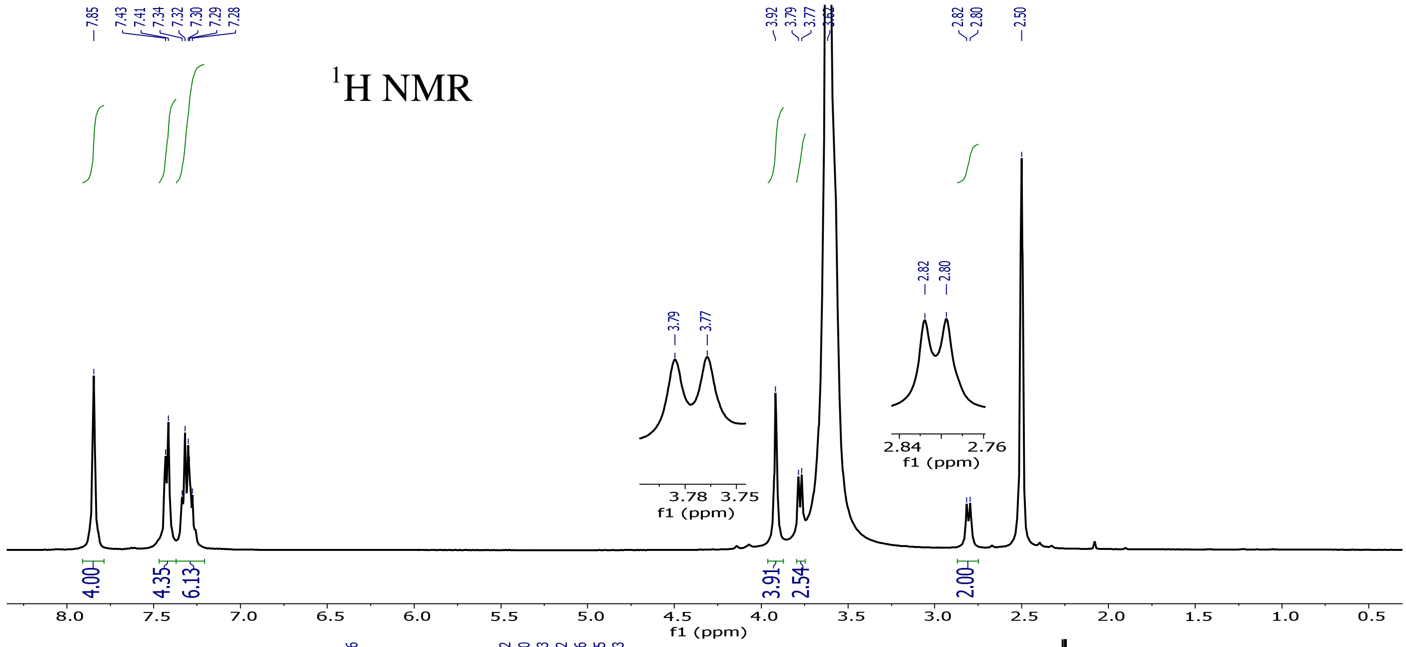

1H NMR
7.85
7.43
7.41
7.34
7.32
7.30
7.29
7.28
3.92
3.79
3.77
2.82
2.80
2.50
4.00
4.35
6.13
3.91
2.54
2.00
f1 (ppm)

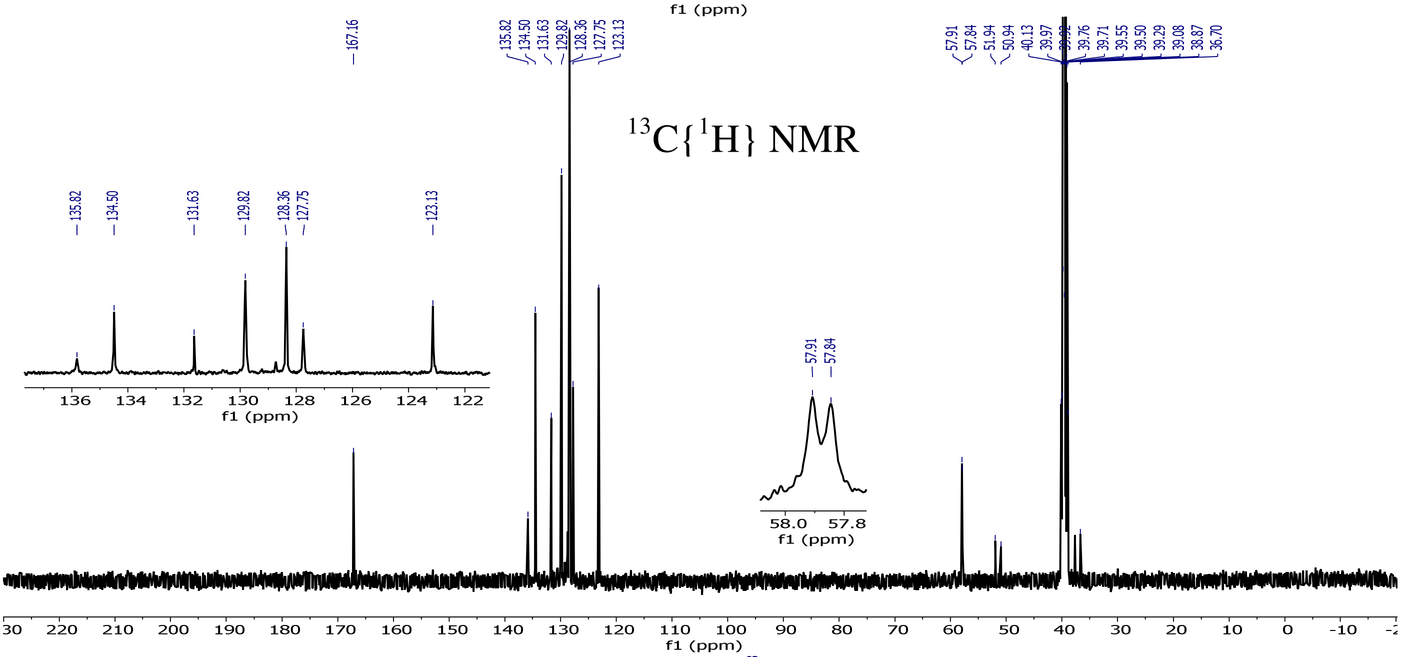

13C{1H} NMR
167.16
135.82
134.50
131.63
129.82
128.36
127.75
123.13
57.91
57.84
51.94
50.94
40.13
39.97
39.76
39.71
39.55
39.50
39.29
39.08
38.87
36.70
f1 (ppm)

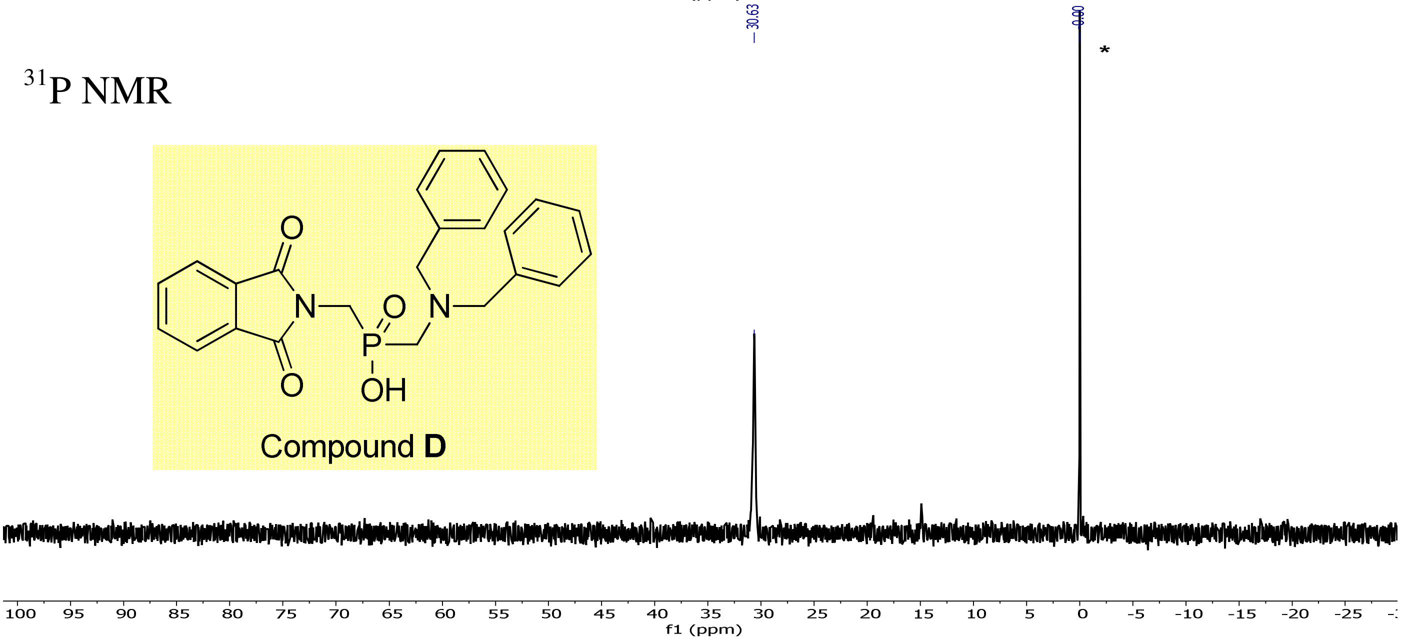

31P NMR
30.63
0.00
*
O
N
O
P
OH
N
O
Compound D
f1 (ppm)

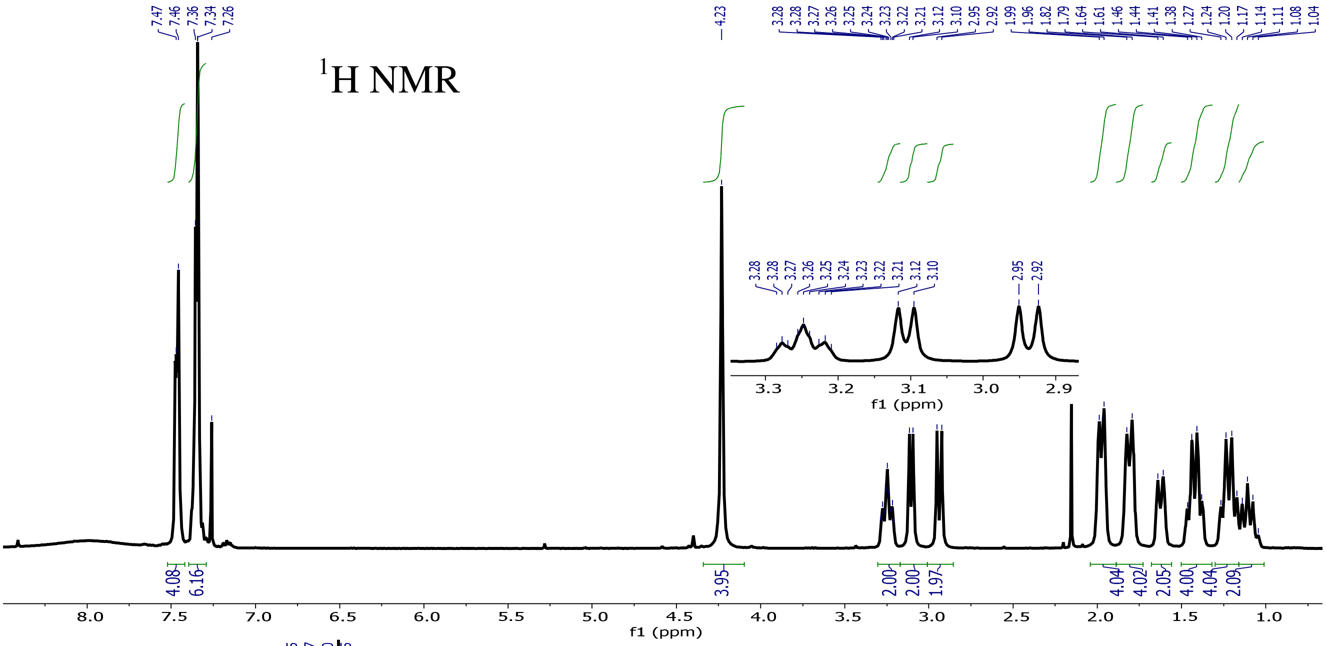
^{1}H NMR
f1 (ppm)

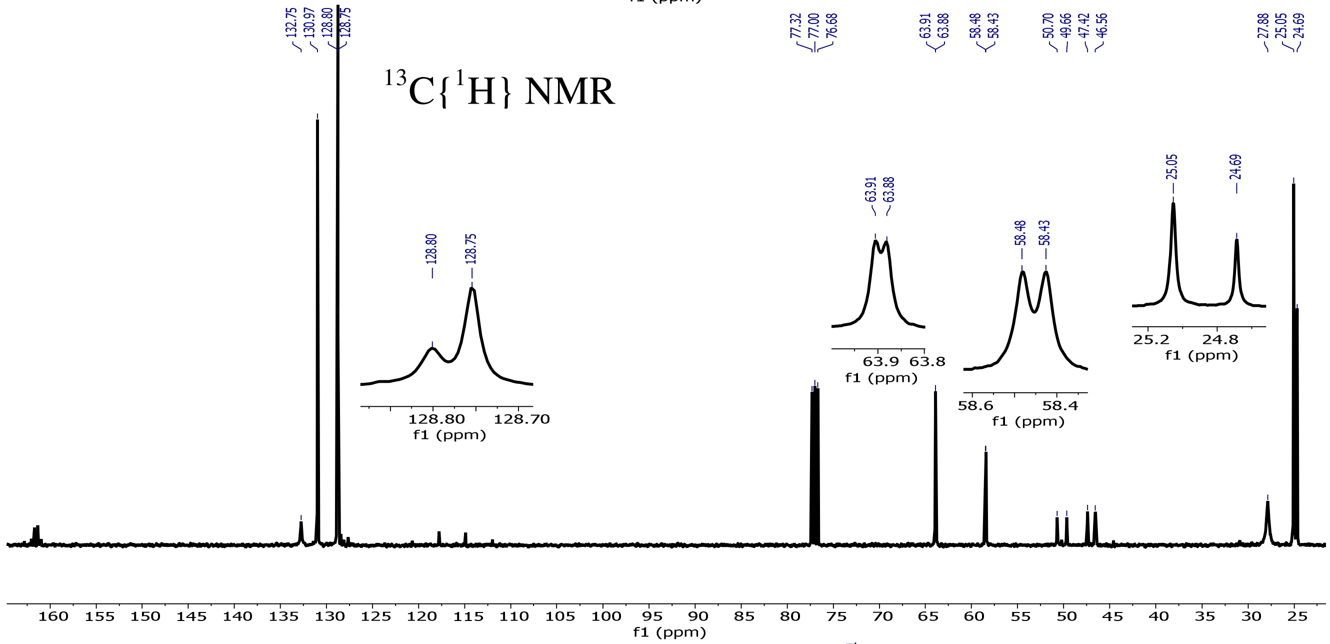
^{13}C{^{1}H} NMR
f1 (ppm)

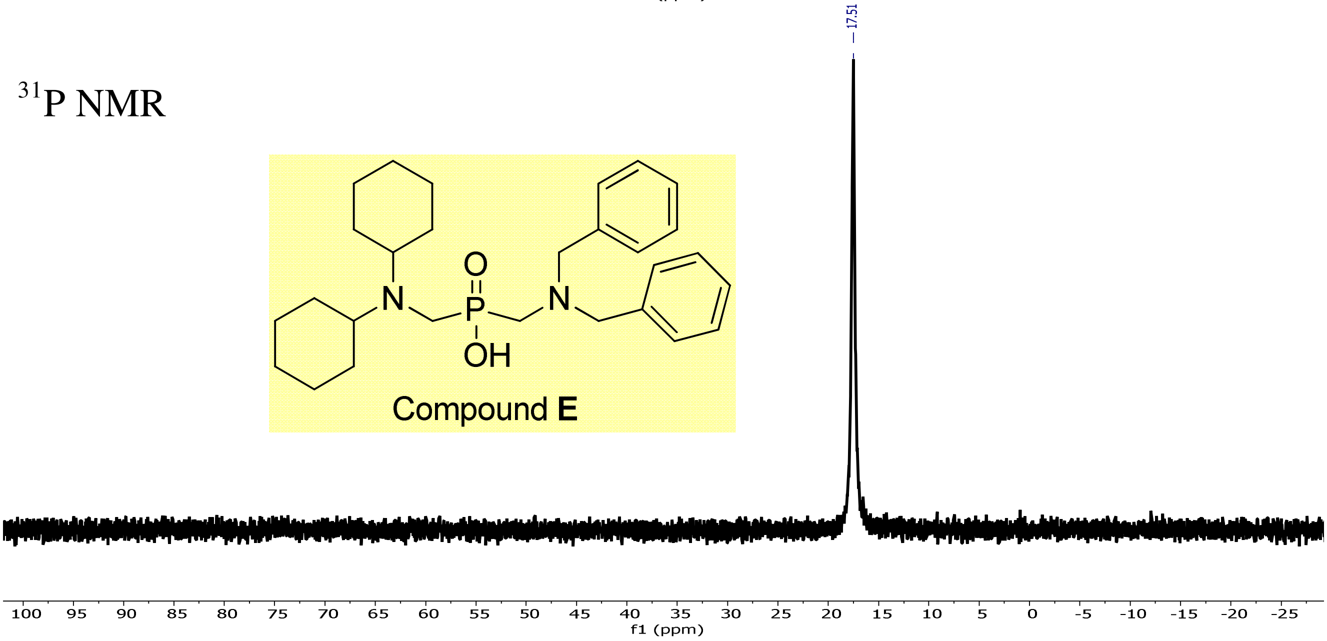
^{31}P NMR
17.51
Compound **E**
f1 (ppm)

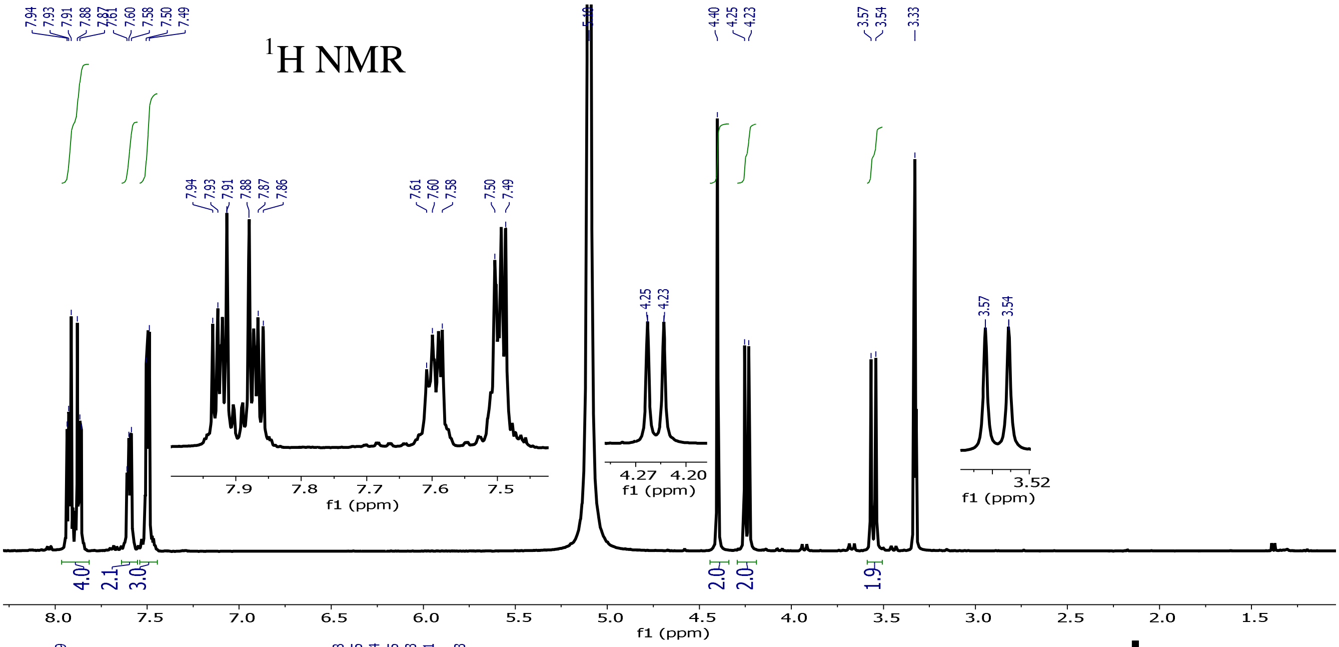

¹H NMR
7.94 7.93 7.91 7.88 7.87 7.61 7.60 7.58 7.50 7.49
4.40 4.25 4.23
3.57 3.54
3.33
4.0 2.1 3.0 2.0 2.0 1.9
f1 (ppm)

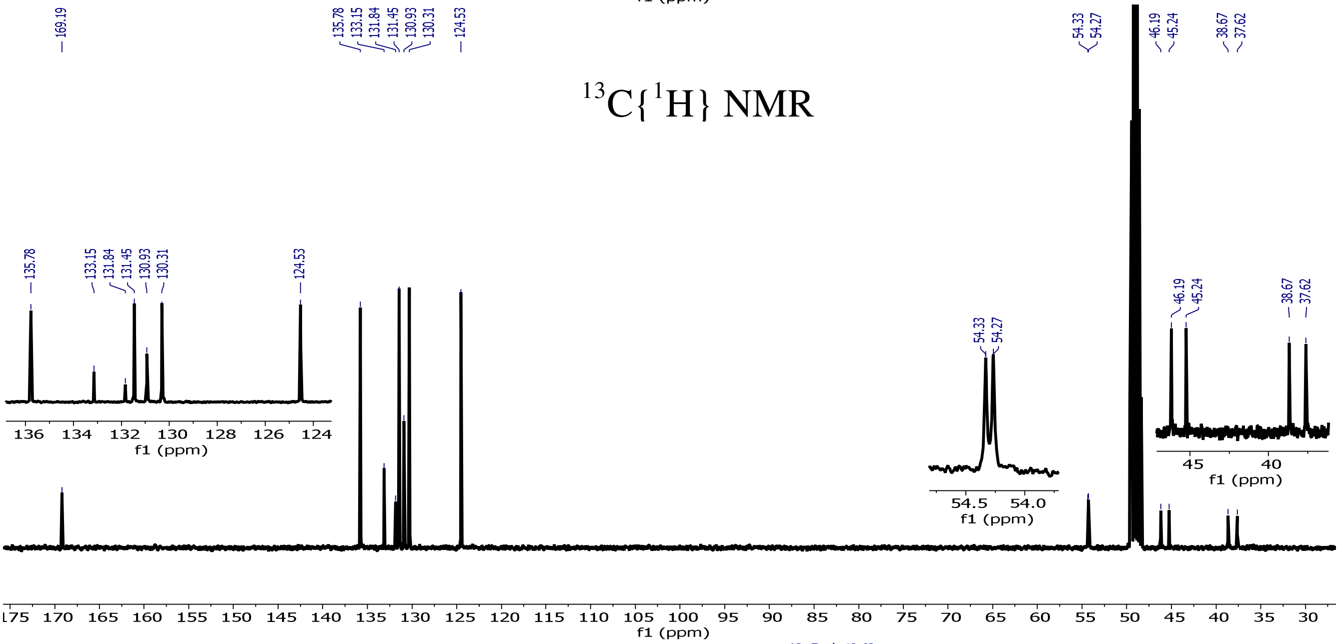

¹³C{¹H} NMR
169.19
135.78 133.15 131.84 131.45 130.93 130.31
124.53
54.33 54.27
46.19 45.24
38.67 37.62
f1 (ppm)

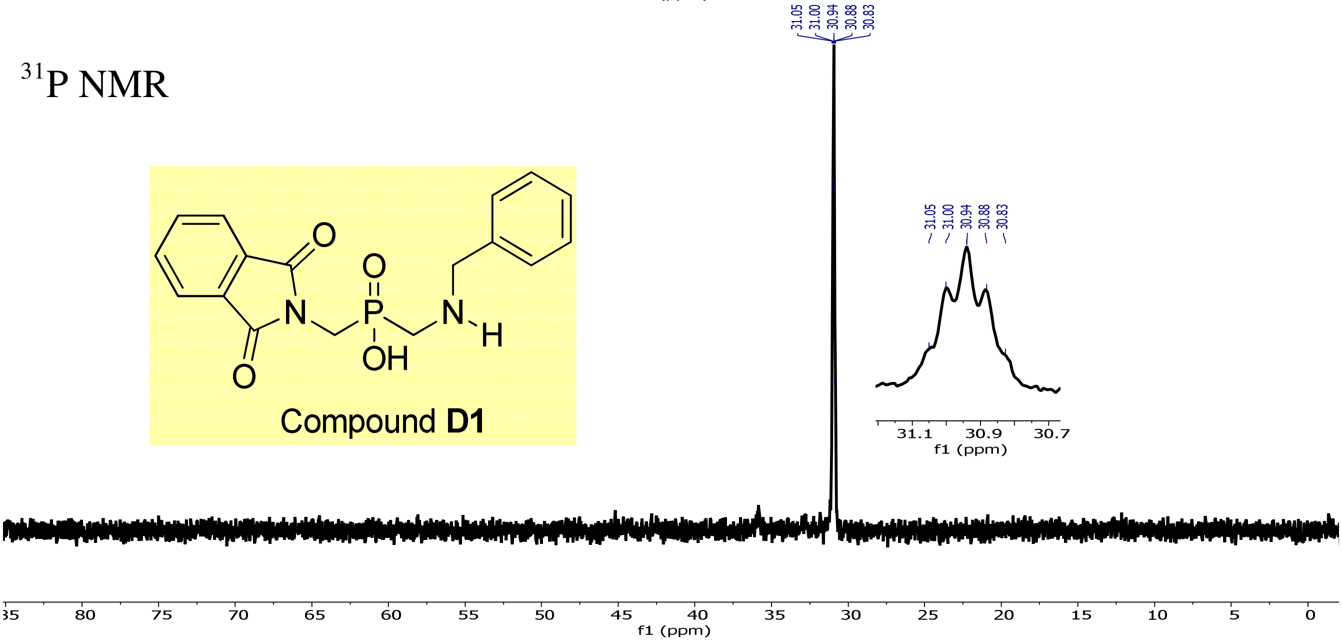

³¹P NMR
31.05 31.00 30.94 30.88 30.83
f1 (ppm)

Compound **D1**

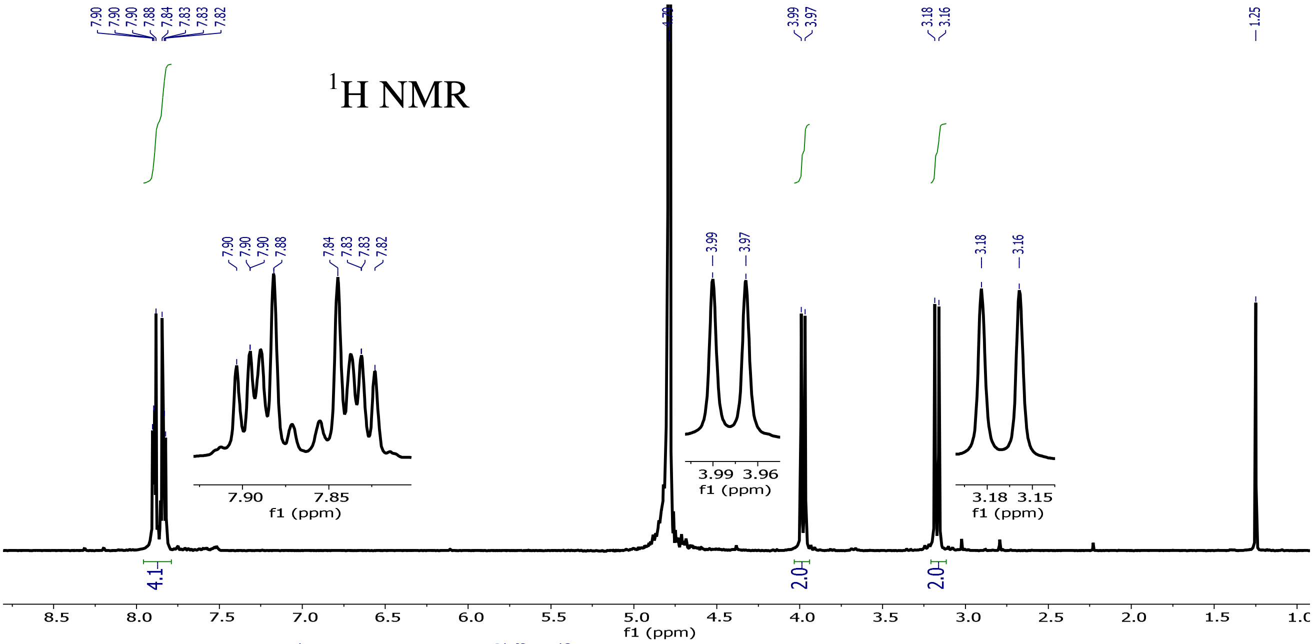
1H NMR
f1 (ppm)

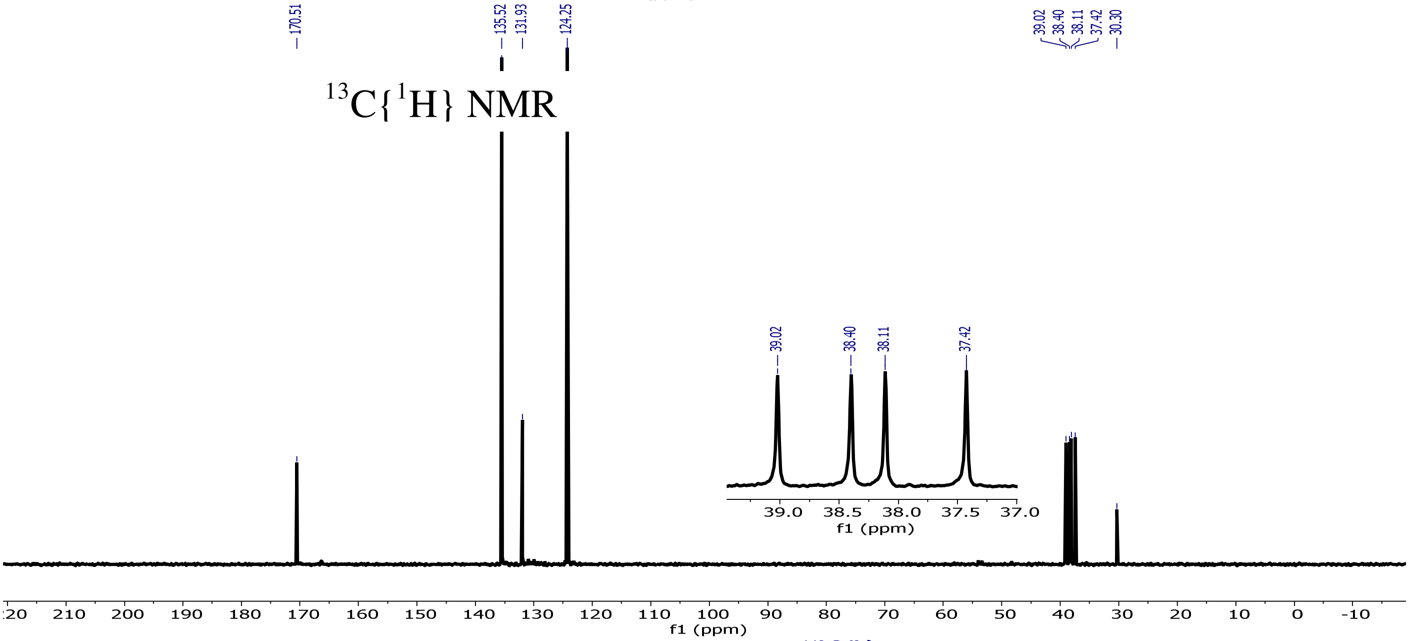
13C{1H} NMR
f1 (ppm)

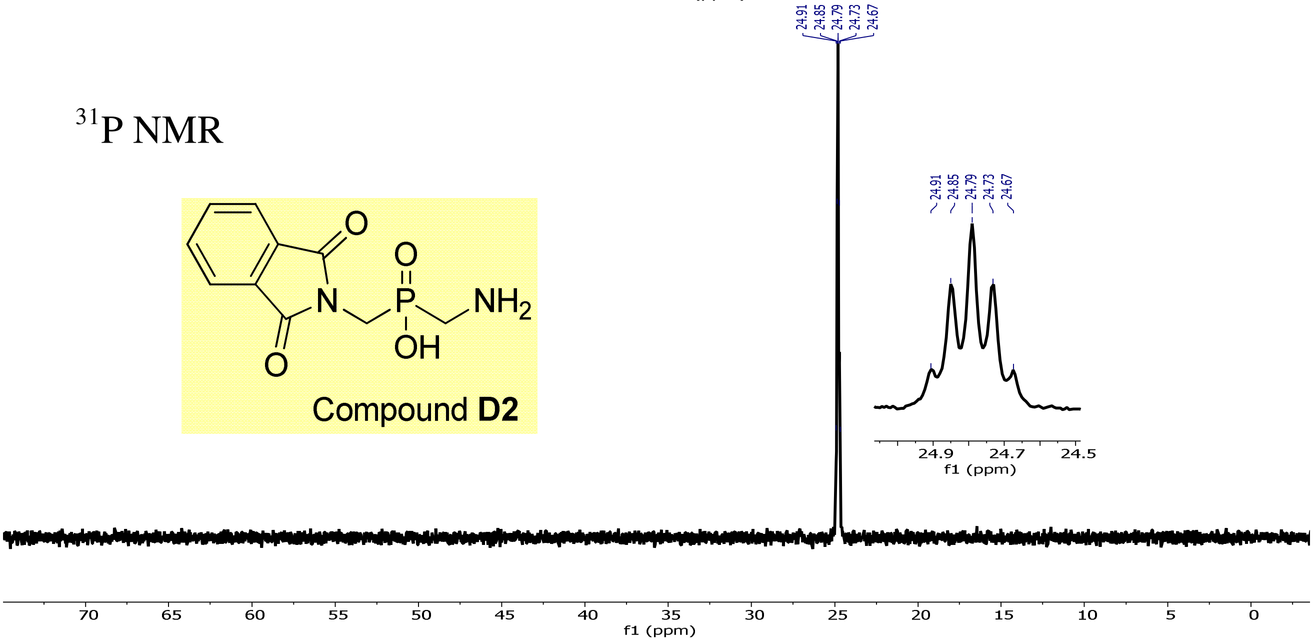
31P NMR
Compound D2
f1 (ppm)

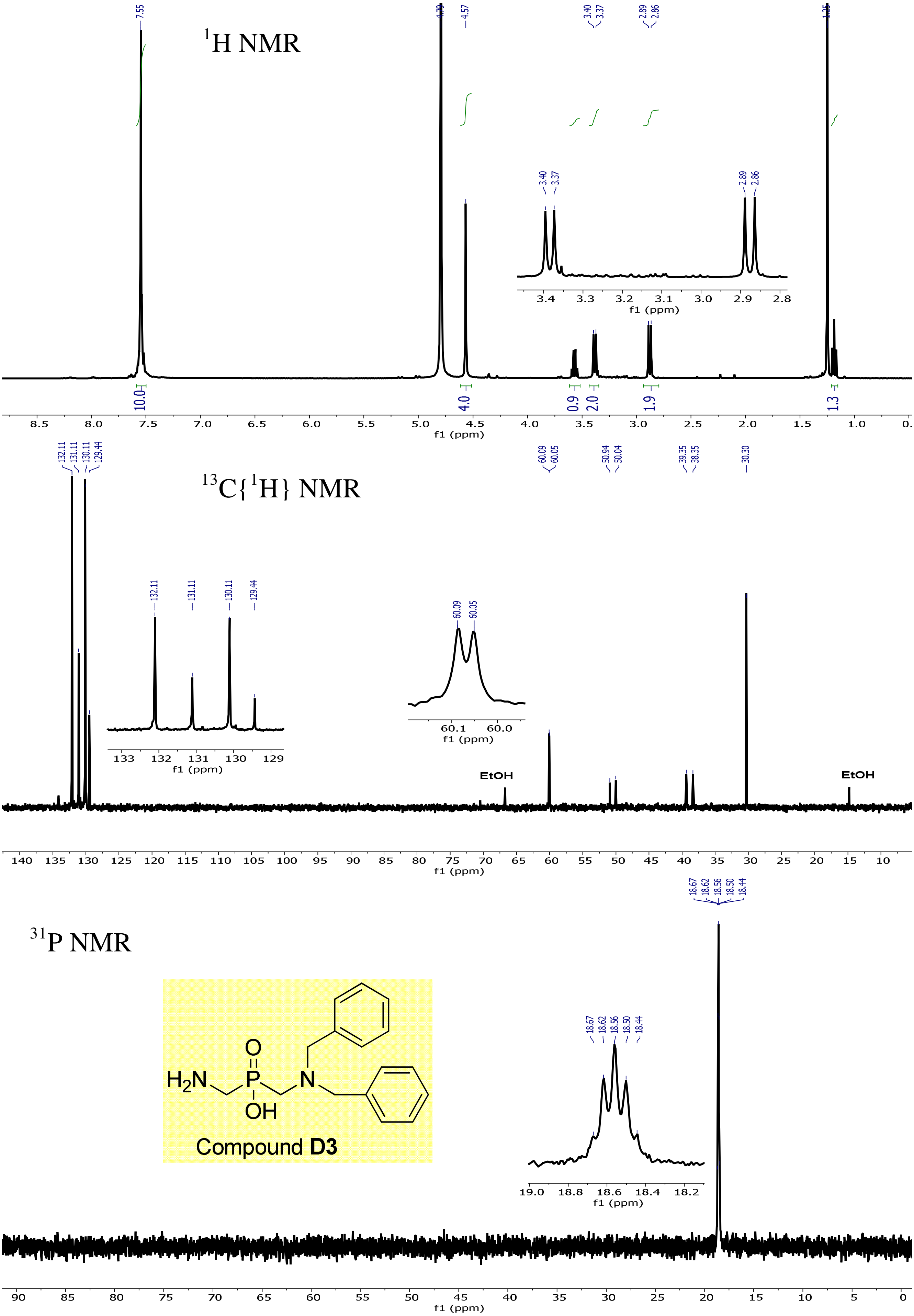

1H NMR
13C{1H} NMR
31P NMR
H2N
O
P
OH
N
Compound D3
EtOH
EtOH
f1 (ppm)

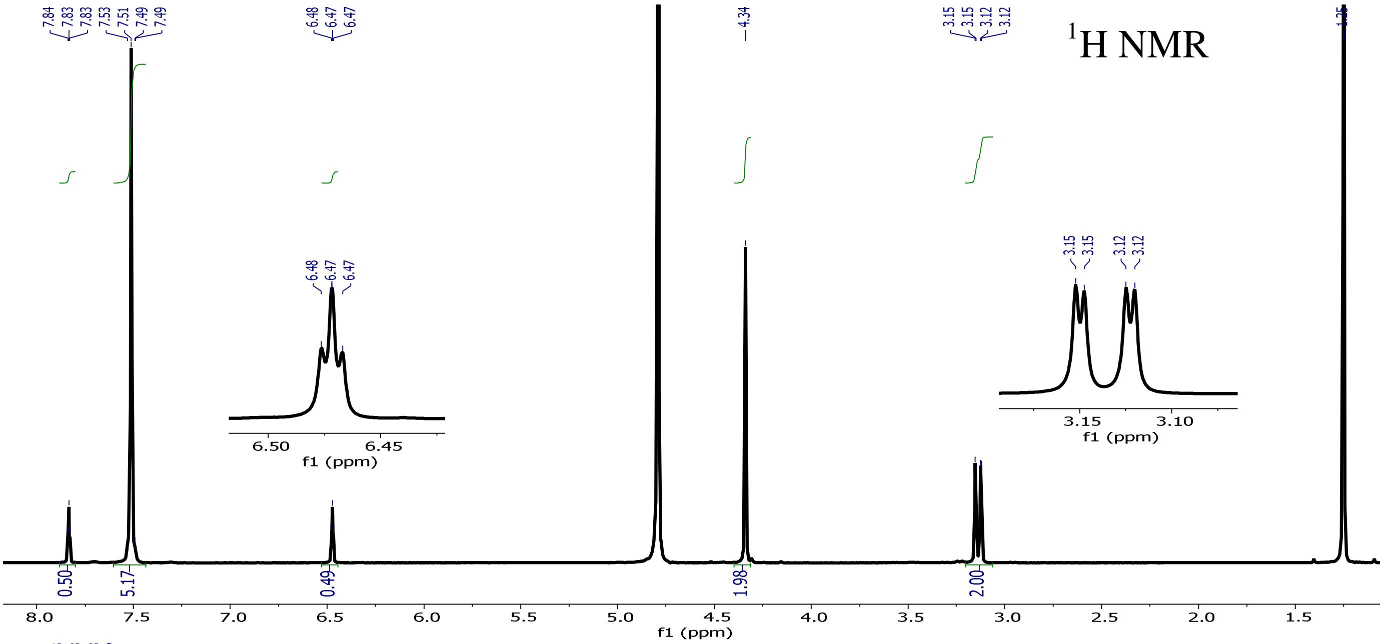

^{1}H NMR
f1 (ppm)

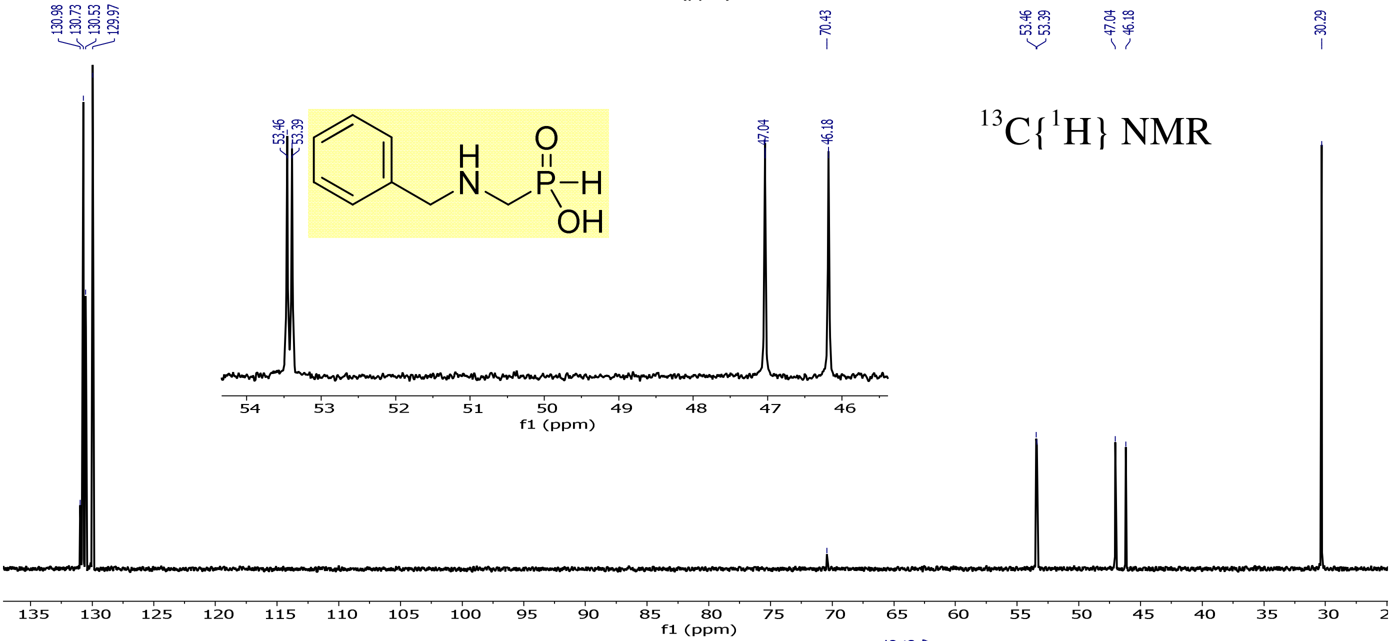

^{13}C{^{1}H} NMR
H
N
O
P
H
OH
f1 (ppm)

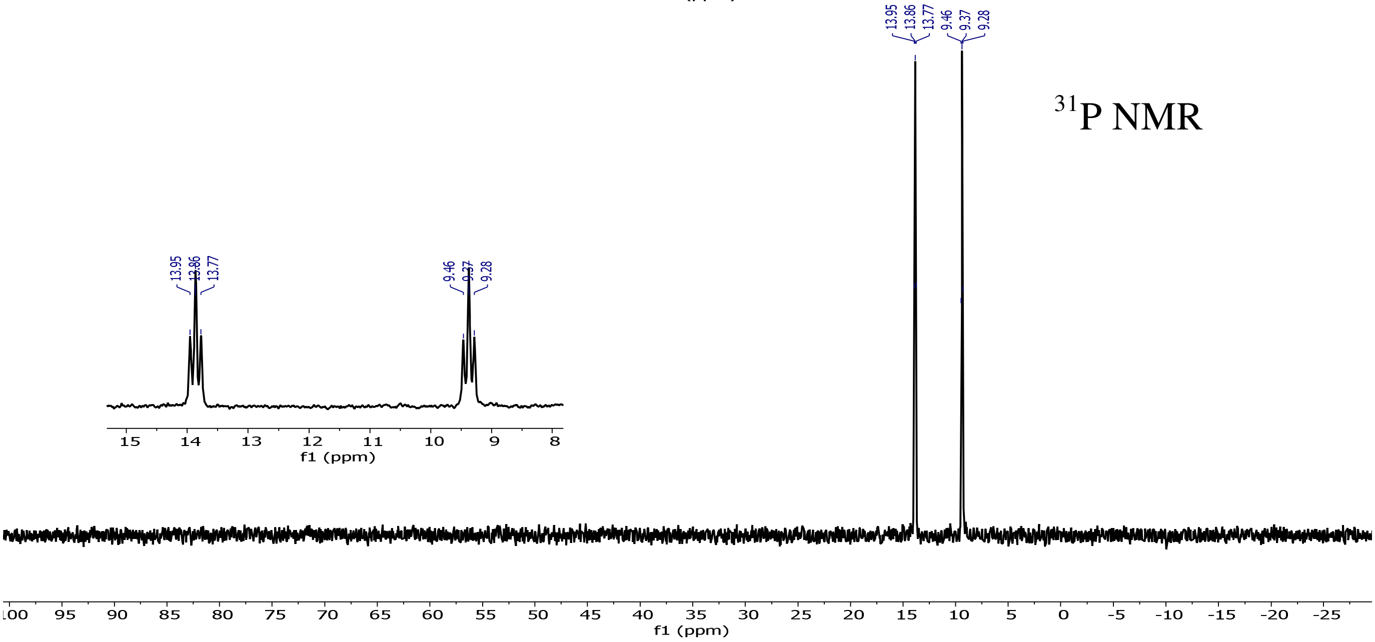

^{31}P NMR
f1 (ppm)
